# Proteins other than the locus of enterocyte effacement-encoded proteins contribute to *Escherichia coli* O157:H7 adherence to bovine rectoanal junction stratified squamous epithelial cells

**DOI:** 10.1186/1471-2180-12-103

**Published:** 2012-06-12

**Authors:** Indira T Kudva, Robert W Griffin, Bryan Krastins, David A Sarracino, Stephen B Calderwood, Manohar John

**Affiliations:** 1 Food Safety and Enteric Pathogens Research Unit, National Animal Disease Center, Agricultural Research Service, U.S. Department of Agriculture, Ames, Iowa, 50010, USA; 2 Division of Infectious Diseases, Massachusetts General Hospital, Boston, Massachusetts, 02114, USA; 3 Harvard Partners Center for Genetics and Genomics, 65 Landsdowne Street, Cambridge, Massachusetts, 02139, USA; 4 Department of Medicine, Harvard Medical School, Boston, Massachusetts, 02114, USA; 5 Department of Microbiology and Molecular Genetics, Harvard Medical School, Boston, Massachusetts, 02114, USA; 6 Present Address: Thermo-Fisher Scientific, Cambridge, Massachusetts, 02139, USA; 7 Present Address: Pathovacs Inc., Ames, Iowa, 50010, USA

**Keywords:** O157, Rectoanal junction, LEE, Adherence, DMEM, GeLC-MS/MS

## Abstract

**Background:**

In this study, we present evidence that proteins encoded by the Locus of Enterocyte Effacement (LEE), considered critical for *Escherichia coli* O157 (O157) adherence to follicle-associated epithelial (FAE) cells at the bovine recto-anal junction (RAJ), do not appear to contribute to O157 adherence to squamous epithelial (RSE) cells also constituting this primary site of O157 colonization in cattle.

**Results:**

Antisera targeting intimin-γ, the primary O157 adhesin, and other essential LEE proteins failed to block O157 adherence to RSE cells, when this pathogen was grown in DMEM, a culture medium that enhances expression of LEE proteins. In addition, RSE adherence of a DMEM-grown-O157 mutant lacking the intimin protein was comparable to that seen with its wild-type parent O157 strain grown in the same media. These adherence patterns were in complete contrast to that observed with HEp-2 cells (the adherence to which is mediated by intimin-γ), assayed under same conditions. This suggested that proteins other than intimin-γ that contribute to adherence to RSE cells are expressed by this pathogen during growth in DMEM. To identify such proteins, we defined the proteome of DMEM-grown-O157 (DMEM-proteome). GeLC-MS/MS revealed that the O157 DMEM-proteome comprised 684 proteins including several components of the cattle and human O157 immunome, orthologs of adhesins, hypothetical secreted and outer membrane proteins, in addition to the known virulence and LEE proteins. Bioinformatics-based analysis of the components of the O157 DMEM proteome revealed several new O157-specific proteins with adhesin potential.

**Conclusion:**

Proteins other than LEE and intimin-γ proteins are involved in O157 adherence to RSE cells at the bovine RAJ. Such proteins, with adhesin potential, are expressed by this human pathogen during growth in DMEM. Ongoing experiments to evaluate their role in RSE adherence should provide both valuable insights into the O157-RSE interactions and new targets for more efficacious anti-adhesion O157 vaccines.

## Background

*Escherichia coli* (*E. coli*) O157 (O157) was first identified as a human enteric pathogen in 1982 and has since been implicated in several outbreaks and sporadic infections [1,2]. Currently, this human pathogen ranks fourth after *Campylobacter, Salmonella*, and *Shigella* among the etiologic agents causing diarrhea in North America [3,4]. Cattle are the primary reservoirs for O157, with the bovine recto-anal junction (RAJ) serving as the primary colonization site for O157. Humans acquire infection by consumption of undercooked beef products such as ground meat or foods contaminated with manure [1,2].

The bovine RAJ comprises of two cell types, the follicle associated epithelium (FAE) towards the distal colon and the stratified squamous epithelium (RSE) closer to the anal canal [5]. Thus far, studies analyzing O157 persistence at the RAJ have focused primarily on its interactions with the FAE cells [6,7]. Proteins encoded on the O157 pathogenicity island, Locus of Enterocyte Effacement (LEE), have been shown to play a critical role in O157 adherence to FAE cells. These include the *E. coli* secreted proteins EspA and EspB, the adhesin Intimin, and the translocated receptor for Intimin, Tir which is secreted via the LEE-encoded type III secretion system (TTSS) [6-8]. Hence, several pre-harvest control measures being evaluated in cattle to control or eliminate O157 from entering the food chain [9-14], include vaccines targeting these LEE-encoded proteins. For instance, Potter et al. developed a vaccine comprising wild-type O157 culture supernatants that contain the TTSS proteins, Tir and Esps [15]; however, similar protection was noted in animals inoculated with the culture supernatant from a mutant strain of O157 lacking the *tir* gene. In addition, the immune response of the vaccinated animals was not merely to the TTSS proteins but also against a number of other proteins that were present in the supernatant. Interestingly, although the vaccine decreased both the number of *E. coli* O157 shed in the feces of vaccinated animals, and those colonizing the terminal rectum, it did not reduce the duration of shedding despite the subcutaneous administration of three doses of the vaccine [15,16]; http://www.bioniche.com. Similar results were also observed with another vaccine that targets the O157 siderophore receptor and porin (SRP) proteins [17,18]; https://animalhealth.pfizer.com. This clearly suggests that unidentified proteins other than those constituting the TTSS or SRP may play a crucial role in bovine colonization, and that the identification and inclusion of such proteins is likely to increase the efficacy of vaccines for elimination of O157 from the gastrointestinal tracts of cattle. Further supporting this inference are reported observations that the TTSS proteins do not significantly contribute to the persistence of O157 in feedlot cattle [19], or to O157 invasion of crypt cells at the RAJ [20].

In a previous study, O157 was observed to adhere to RSE cells in vivo and in vitro, besides the FAE cells [5] and this observation was used to develop a unique in vitro adherence assay for O157 with RSE cells [5]. In this study, we decided to (i) evaluate if the LEE-encoded proteins would also be critical for O157 adherence to RSE cells, as for FAE cells, and (ii) in the event that these proteins would not play a significant role in RSE cell adherence, define the proteome of O157 as expressed when grown in the adherence assay media, DMEM, to assemble targets for future evaluation in RSE adherence. Experimental and bioinformatic evaluation of such targets could in fact help identify a subset of novel adhesins that may have excellent potential to increase the efficacy of the anti-adhesion, cattle O157 vaccines, by eliminating O157 from both FAE and RSE cells at the RAJ.

## Methods

### Bacterial strains and culture conditions

The wild-type O157 strain EDL933 (O157), a sequenced isolate implicated in human disease [21], was used in this study. We cultured O157 in Dulbecco Modified Eagle Medium-Low Glucose (DMEM; Gibco/lnvitrogen Corporation, Grand Island, NY), for the cell adherence assays described below. The rationale for the use of this culture medium was (i) to reflect the growth conditions used in the eukaryotic cell adherence assays; and (ii) to closely parallel the in vivo nutrient-limiting conditions, and conditions used to prepare the cattle-use approved, LEE protein based, anti-adhesion O157 vaccine. In addition, another wild-type O157 strain 86–24 (86–24), its isogenic mutant (86-24*eae* Δ10) negative for Intimin, and this mutant complemented with the plasmid pEB310 (86-24*eae* Δ10(pEB310)) expressing Intimin, were also tested in the adherence assay [22]. The 86–24 strain and its derivatives were obtained from Dr. A. D. O’Brien, Uniformed Services University of the Health Sciences, Bethesda, MD.

We also cultured O157 in DMEM for proteomic analysis. Specifically, an overnight culture of the wild-type O157 strain in Luria-Bertani (LB) broth was pelleted and washed with sterile phosphate buffered saline (PBS; pH 7.4), and subcultured to an initial OD_600_ of 0.05 in fresh DMEM. After incubation at 37 °C with shaking at 250 rpm to an OD_600_ of 0.8 to 1.0, cells were harvested by centrifugation at 7,000 rpm, 15 min at 4 °C. Cells were washed three times with an equal volume of sterile PBS (pH 7.4), and processed to obtain cell lysate and pellet fractions for proteomic analysis as previously described [23].

### O157-RSE cell adherence inhibition assay: (i) in the presence of pooled anti-LEE proteins, anti-intimin and anti-H7 antisera

Adherence of O157 to the RSE cells was previously demonstrated and developed into an adherence assay in our laboratory [5]. In this study, the ability of pooled, rabbit polyclonal antisera to interfere with and inhibit O157 adherence to RSE cells was evaluated. Specifically, antisera generated against the recombinant LEE-encoded proteins, Tir, EspA and EspB, and Intimin, in rabbits (National Animal Disease Center Stocks), was pooled. Rabbit antisera targeting the O157 flagellar antigen H7 (Difco Laboratories, Inc., Detroit, MI) was also mixed into the pooled antisera, which was then tested at 1:5 and 1:10 dilutions. Specificity was confirmed by reacting each antiserum against both O157 cell lysates and the cognate protein in western blotting experiments (data not shown). Rabbit sera (Sigma-Aldrich, St. Louis, MO) from healthy animals (normal rabbit sera), at a 1:5 dilution, was used as a control.

### (ii) In the presence of anti-Intimin antisera alone

To specifically evaluate the role of intimin, the rabbit anti-Intimin antisera was evaluated separately for its ability to prevent O157 adherence to RSE cells at 1:5 and 1:10 dilutions.

Each of the RSE adherence assays was conducted in 8 technical and 2 biological replicates as described previously [5], with minor modifications, as follows. RSE cells were washed and resuspended in 1 ml Dulbecco Modified Eagle Medium – No Glucose (DMEM-NG) ± 2.5% D + Mannose, in 16 x 100 mm glass tubes, to a final concentration of 10^5^ cells/ml. Although Type 1 fimbriae are not expressed by O157, we included D + Mannose in parallel assays to cover any hitherto unknown transient expression especially in mutant strains. Bacterial pellets from overnight cultures in DMEM, incubated at 37°C without aeration, were resuspended in sterile saline with or without antisera (‘no sera’ control), and incubated at 37°C for 30 min. The bacteria-antibody mix was then added to the RSE cells suspension to final bacteria:cell ratio of 10:1, and the mixture incubated with aeration (37°C, 110 rpm, for 4 h). At the end of 4 h, the mixture was pelleted and washed thoroughly, once with 14 ml DMEM-NG, and twice with 14 mls of sterile, distilled water (dH_2_O) before reconstituting in 100 μl dH_2_O. Eight 2 μl drops of this suspension were placed on Polysine (Thermo Scientific Pierce) slides and dried overnight under direct light to quench non-specific fluorescence, before fixing in cold 95% ethanol for 10 min. The slides were then stained with 1% toluidine blue, or with fluorescence-tagged antibodies that specifically target O157 and the RSE cell cytokeratins as described previously [5]. Each experiment was then done in duplicate.

O157 adherence patterns on RSE cells were recorded as diffuse, or aggregative (clumps) for all positive interactions that involved direct association with the cells [5]. Scattered bacteria and bacterial micro-colonies not adhering to cell membranes were considered to be negative for adherence to the epithelial cells [5]. A total of 100–160 well dispersed RSE cells (10–20 cells per drop or chamber) were analyzed per slide as described previously [5]. The percent RSE cells with and without bacteria adhering to them were determined [5]. If more than 50% of RSE cells had >10 bacteria attached, the adherence was recorded as strongly positive. For >50% RSE cells with 1–10 adherent bacteria, the adherence was recorded as moderately positive. For less than 50% RSE cells with 1–5 adherent bacteria, the result was recorded as non-adherent.

### O157-HEp-2 cell adherence inhibition assay

The role played by LEE-encoded proteins and Intimin in the adherence of O157 to HEp-2 cells, has already been defined previously [22] and hence, this assay was used for comparative reasons. The assay was conducted as described previously [5] except that, the washed bacterial pellets were incubated with or without antisera (‘no sera’ control), at 37°C for 30 min, prior to addition to the HEp-2 cells. Both the pooled antisera and anti-intimin antisera, as described above, were used at dilutions ranging from 1:5 to 1:100 in these assays. Each assay was conducted in duplicate, and in 3–6 chambers of the chamber slides per run. Slides were stained with 1% toluidine blue, or with fluorescence-tagged antibodies that specifically target O157 and the HEp-2 cell actin filaments as described previously [5] and adherence patterns recorded as for RSE cells (see above).

### Adherence of 86–24, 86-24*eae* Δ10, and 86-24*eae* Δ10(pEB310), to RSE and HEp-2 cells

Wild-type 86–24 and its mutant derivatives were used to verify the role of Intimin directly and compare the results with that of the O157 adherence inhibition assays. This assay was conducted, recorded, as previously described and done in the absence of any antisera [5].

### OneDimensional (1D) SDS-PAGE liquid chromatography tandem mass spectrometry (GeLC-MS/MS)

Top down proteomic analysis was done at the Harvard Partners Center for Genetics and Genomics, Cambridge, Massachusetts. O157 cell pellet and lysate fractions were concentrated using spin filters (MW cutoff 5000 Daltons; Vivascience Inc., Englewood, NY), fractionated on 1D SDS-PAGE, and digested in-gel with trypsin prior to tandem mass spectrometry (MS/MS) as described previously [23]. The rationale for incorporating a 1D SDS-PAGE fractionation step is that this modification reduces complexity of protein mixtures, permits a larger dynamic range of protein identification, and allows for significantly better reproducibility [24,25].

For mass spectrometry (MS), samples were subjected to three different runs on an LCQ DECA XP plus Proteome X workstation (LCQ) from Thermo Finnigan as described earlier [23,26]. For each run, 10 μL of each reconstituted sample was injected with a Famos Autosampler, and the separation was done on a 75 μm (inner diameter) x 20 cm column packed with C_18_ media running at a flow rate of 0.25 μl/min provided from a Surveyor MS pump with a flow splitter with a gradient of water, 0.1% formic acid and then 5% acetonitrile, 0.1% formic acid (5%-72%) over the course of 480 min (8.0 hour run). Between each set of samples, a standard of a 5 Angiotensin mix of peptides (Michrom BioResources) was run to ascertain column performance, and to observe any potential carryover that might have occurred. The LCQ was run in a top five configuration, with one MS scan and five MS/MS scans. Dynamic exclusion was set to 1 with a limit of 30 seconds.

Peptide identifications were made using SEQUEST (Thermo Finnigan) through the Bioworks Browser 3.2, as described previously [23]. Sequential database searches were performed using the O157 strains EDL933 and Sakai FASTA database from European Bioinformatics Institute http://www.ebi.ac.uk/newt/display using static carbamidomethyl-modified cysteines and differential oxidized methionines. These protein databases (*Escherichia coli* (strain Sakai/O157:H7/RIMD 0509952/EHEC) – Tax ID: 386585 and *Escherichia coli* (strain EDL933/ATCC 700927/O157:H7/EHEC) – Tax ID: 155864) have a total of 10,737entries. A reverse O157 strain EDL933 FASTA database was spiked in to provide noise and determine validity of the peptide hits, so that known and theoretical protein hits can be determined without compromising the statistical relevance of all the data [26]. The MS data was searched with a 2-Dalton window on the MS precursor with a 0.8 Dalton on the fragment ions. Peptide score cutoff values were chosen at cross-correlation values (Xcorr) of 1.8 for singly charged ions, 2.5 for doubly charged ions, and 3.0 for triply charged ions, along with delta rank scoring preliminary cutoff (deltaCN) values of 0.1, and cross-correlation normalized values (RSp) of 1. The cross-correlation values chosen for each peptide assured a high confidence match for the different charge states, while the deltaCN values ensured the uniqueness of the peptide hit. The RSp value of 1 ensured that the peptide matched the top hit in the preliminary scoring. At these peptide filter values, very few reverse database hits were observed, which permitted a higher confidence in the few single peptide protein identifications. Furthermore, single hit proteins were manually validated to ensure relevance.

### Bioinformatics

Cellular location of proteins was determined using amino acid sequences of cognate proteins in the O157 sequence databases at http://www.ncbi.nlm.nih.gov/protein. In addition, extracytoplasmic proteins were verified for the presence of signal sequences using the program SignalP 3.0 at http://www.cbs.dtu.dk/services/SignalP, and subcellular localization of other proteins confirmed using the PSORT/PSORT-B program (http://psort.nibb.ac.jp/). Putative functions were determined by querying the Conserved Domain Database (CDD) at http://www.ncbi.nlm.nih.gov/Structure/cdd/wrpsb.cgi Protein components of the O157 DMEM-proteome with adhesion potential were shortlisted using Vaxign, a reverse vaccinology based vaccine target prediction and analysis system at http://www.violinet.org that utilizes the SPAAN algorithm [27].

## Results and discussion

LEE- encoded proteins considered critical for O157 adherence to FAE cells at the RAJ did not appear to have a role in O157 adherence to RSE cells at this same site. Both O157 strains grown in DMEM and pre-incubated with pooled, polyclonal antisera generated against the LEE (Tir, EspA, EspB, and Intimin) and flagellar H7 proteins, or the anti-Intimin antisera alone, at 1:5 and 1:10 dilution, continued to adhere to the RSE cells, irrespective of the presence/absence of D + Mannose. Data is shown for one of the O157 strains in the presence of D + Mannose (Additional file 1, Figure 1, panel A, Figure 2). These results were consistent between all trials, irrespective of toluidine blue or immunofluorescent staining, and did not show any differences in the adherence patterns compared to the controls. The same O157-RSE cell-adherence pattern was observed in the controls with normal rabbit sera added at 1:5 dilution (data not shown), and in the absence of any sera (Additional file 1, Figure 1, panel B; Figure 2) [5], irrespective of the presence/absence of D + Mannose. The continued adherence of O157 to the RSE cells in the presence of antibodies to the LEE proteins may have been due to the masking of these antigens and the unmasking of other O157 adhesins targeting the receptors on the RSE cells. To that effect an increase in the total number of RSE cells with adherent bacteria and decrease in the total number of RSE cells with no adherent bacteria was observed, in the presence of pooled and anti-Intimin antisera (Figure 2). We intentionally included antisera targeting the flagellar antigen H7 as flagella have been demonstrated to play a role in initial adherence to plant cells and the FAE [28,29]. These results suggest that additional mechanisms of adherence, distinct from those attributable to LEE, Intimin and flagellar H7 proteins, are involved in O157 attachment to the RAJ squamous epithelial cells.

**Figure 1:**
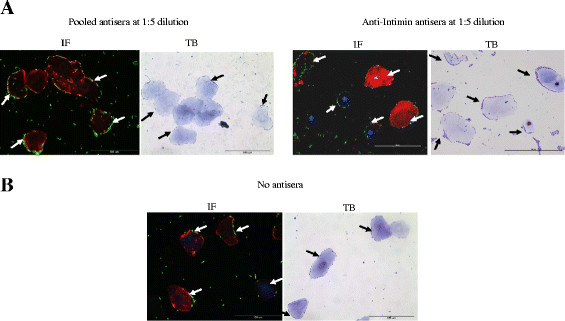
**Adherence patterns of O157 strain EDL 933 on RSE cells, in the presence of D + Mannose and +/− antisera.Panel A**, in the presence of “pooled antisera” against LEE, Intimin and flagellar H7 proteins, and the anti-Intimin antisera alone, at 1:5 dilutions. **Panel B**, in the absence of any sera (No sera). The immunofluorescence (IF) stained slides are shown at 40x magnification. O157 have green fluorescence, cytokeratins’ of RSE cells have orange-red fluorescence, and their nuclei have blue fluorescence. The arrows in the adjacent toluidine blue (TB) stained slides, at 40x magnification, point to RSE-adherent O157.

**Figure 2:**
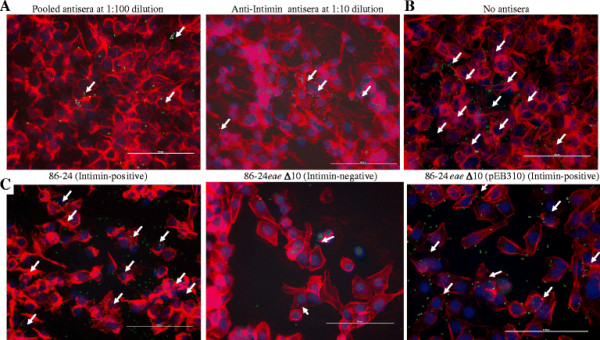
**Quantitative representation of the adherence patterns of O157 strains EDL 933, and 86–24 along with its mutant derivatives, on RSE and HEp-2 cells.** Percent mean ± standard error of mean of cells with adherent bacteria or no bacteria, in the ranges shown in the legend, are depicted in each graph.

**Figure 3:**
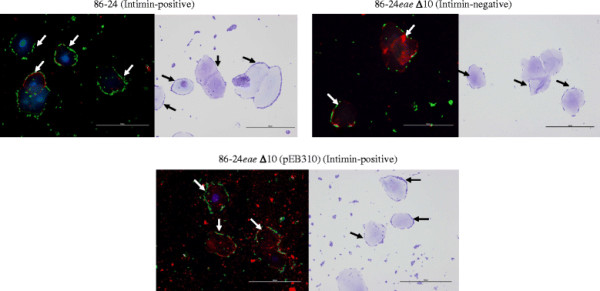
**Adherence patterns of O157 strains on HEp-2 cells, in the presence of D + Mannose and +/− antisera.Panel A**, O157 strain EDL933, in the presence of “pooled antisera” against LEE. Intimin and flagellar H7 proteins, and the anti-Intimin antisera alone, at 1:100 and 1:10 dilutions, respectively. **Panel B**, O157 strain EDL933, in the absence of any sera (No sera). **Panel C**, O157 strain 86–24 (Intimin-positive) and its mutant derivatives, 86-24*eae* Δ10 (Intimin-negative), and 86-24*eae* Δ10 (pEB310) (Initmin-positive) in the absence of any sera. The immunofluorescence (IF) stained slides are shown at 40x magnification. O157 have green fluorescence, actin filaments of HEp-2 cells have orange-red fluorescence, and their nuclei have blue fluorescence.

On the other hand, the LEE-encoded proteins were critical to O157 adherence to HEp-2 cells as demonstrated previously [22], with or without D + Mannose. As shown in Additional file 2 and Figure 3, panel A and Figure 2, preincubation with the pooled polyclonal antisera and the anti-Intimin antisera significantly interfered with and prevented O157 adherence to HEp-2 cells, at all dilutions tested. An increase in number of HEp-2 cells without any adhering bacteria was observed in the presence of either antiserum, accordingly (Figure 2). However, pre-incubation with normal rabbit sera at 1:5 dilution (data not shown) showed the same diffuse, moderate adherence as in the absence of any antisera (Additional file 2, Figure 3 panel B and Figure 2).

**Figure 4:**
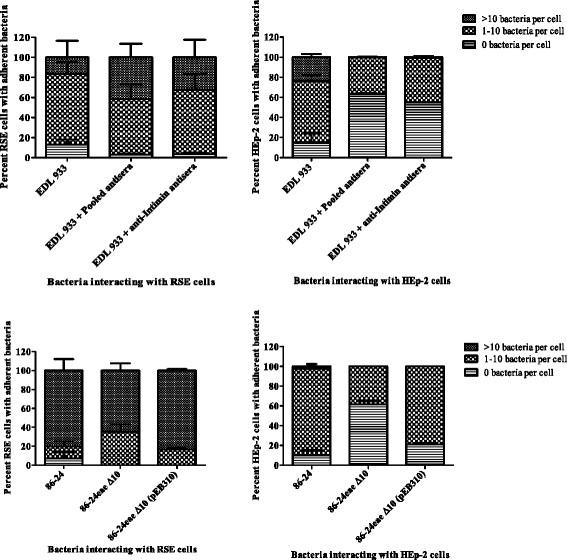
**Adherence patterns of O157 strain 86–24 (Intimin-positive) and its mutant derivatives, 86-24*eae*Δ10 (Intimin-negative) and 86-24*eae*Δ10 (pEB310) (Initmin-positive), on RSE cells, in the presence of D + Mannose.** The immunofluorescence (IF) stained slides are shown at 40x magnification. O157 have green fluorescence, cytokeratins’ of RSE cells have orange-red fluorescence, and their nuclei have blue fluorescence. The arrows in the adjacent toluidine blue (TB) stained slides, at 40x magnification, point to RSE-adherent O157.

The results observed with the adherence inhibition assays were further verified by the adherence patterns of O157 strain 86–24 (86–24) and its mutant derivatives on HEp-2 and RSE cells (Figure 3, panel C, Figures 4 and 2). The intimin-negative mutant 86-24*eae* Δ10 did not adhere well to the HEp-2 cells compared to the intimin-positive, wild-type 86–24 or complemented mutant, 86-24*eae* Δ10(pEB310) that demonstrated diffuse, moderate adherence (Figure 3, panel C, Figure 2, and Additional file 2). Actin accumulation observed in the majority of HEp-2 cells with 100x magnification only in the presence of 86–24 and 86-24*eae* Δ10(pEB310), along with an increase in the number of HEp-2 cells without adhering bacteria in the presence of 86-24*eae* Δ10, further verified these observations (data not shown). This confirmed the role of intimin in O157 adherence to HEp-2 cells. On the otherhand, 86–24 and all its mutant derivatives demonstrated diffuse, strong adherence to RSE cells, irrespective of intimin expression (Figures 4 and 2, and Additional file 1). Infact with 86-24*eae* Δ10, the number of RSE cells with adhering bacteria actually increased, which suggested that intimin did not have a role in the adherence of O157 to RSE cells.

In order to verify if proteins other than LEE proteins were being expressed by O157 upon growth in DMEM which could have a possible role in O157 adherence to RSE cells, we analyzed the O157 proteome as expressed in DMEM. While the proteome of O157 has been analyzed under various other growth conditions [30-33] we decided to evaluate the same following growth in DMEM for several reasons, such as (i) this was the media used to culture both bacteria and the RSE cells, separately, prior to the adherence assays, (ii) the media closely mimicked the nutrient-limiting conditions seen in vivo, and (iii) this media closely matched that used to develop a commercially available cattle, O157 vaccine [15, 16; http://www.bioniche.com. Our observations did not support a role for other host (RSE-cell)-derived factors in this adherence of O157 and hence, we did not evaluate RSE-cell adherence of O157 cultured in eukaryotic cell-conditioned media. This inference came from the fact that similar adherence results were obtained when DMEM was supplemented with norepinephrine (NE; DMEM-NE), a host neuroendocrine hormone that is encountered by O157 in vivo during the actual process of infection (data not shown). NE is reportedly a mimic of autoinduer 3 (AI-3), which regulates O157 virulence gene expression via quorum sensing [34]. Further, Intimin, its receptor, Tir, as well as EspB were expressed in equivalent amounts in both DMEM and DMEM-NE, as observed using western blotting by others [34], and by us, and also using top down proteomics by us (data not shown).

**Figure 5:**
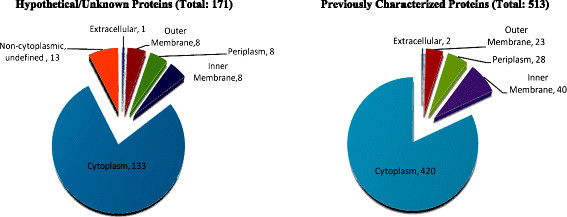
Bacterial cell localization of proteins comprising the O157 DMEM-proteome.

A total of 684 proteins were identified as being part of the O157 DMEM-proteome (13% of the O157 sequenced proteome), and these included several characterized and hypothetical/unknown proteins besides the TTSS proteins. While 171 of these proteins were uncharacterized with hypothetical functions assigned in the O157 genome [21; Figure 5, Additional files 3 and 5–12], the remaining 513 proteins localized to various bacterial cell compartments with functions including metabolic, cell division, regulatory, transport, environmental adaptation, and previously characterized O157 virulence factors [21]; Figure 5, Additional files 4 and 5,6,7,8,9,10,11,12. Proteins associated with O157 virulence or adherence in the DMEM-proteome included Tir, Intimin, EspB, LuxS, Iha, OmpA, KatP, ChuA, EspP, Stx1A, Stx1B, and Stx2B [20]; Additional files 4 and 5,6,7,8,9,10,11,12. Interestingly, 64 of the 684 (9.4%) proteins comprising the O157 DMEM-proteome were also part of the O157 immunoproteome in cattle, defined using the innovative proteome mining tool, Proteomics- based Expression Library Screening (PELS) [23]; Additional files 3 and 4. In addition, nine members of the DMEM-proteome were also part of the O157 immunome in humans [26].

Given that a subset of pathogen proteins targeted by the host immune system help pathogens overcome hostile in vivo environments and rapidly adapt to host niches, counter host defenses, survive, propagate, and establish infection, it is very likely that proteins other than LEE proteins in the O157 DMEM-proteome play a role in O157 adherence to the RSE cells at the RAJ. The identification and inclusion of such proteins in anti-adhesion vaccine preparations, and their optimal administration to the host immune system, may enhance the efficacy of such vaccines in reducing or eliminating O157 not only from the FAE cells but also from the RSE cells of the bovine RAJ. Our strategy for selecting proteins constituting the O157 DMEM proteome with potential to function as adhesins for further evaluation was to employ the SPAAN algorithm [27], which is part of the web-based vaccine target prediction and analysis system at http://www.violinet.org. In particular, we shortlisted proteins that were shared by four sequenced O157 strains, namely, strain EDL933, strain EC4115, strain Sakai, and strain TW14359. Our analysis identified 36/684 components of the O157 DMEM-proteome to have adhesin potential, including the extensively reported primary O157 adhesin, intimin-γ, and its protein receptor, Tir (Table 1). Three O157-specific proteins, namely ChuA, TerE, and a putative outermembrane porin protein on O-island #62 (Table 1), have been prioritized and are under experimental evaluation for a role in O157 adherence to RSE cells. Also, towards studies of common STEC adherence mechanisms, we have determined homologs of several of these putative adhesins including those encoded on O-islands such as, Tir, Intimin, ChuA, TerE, EspP, bioinformatically (psiBLAST, National Center for Biotechnology Information; http://www.ncbi.nih.gov), in sequenced non-O157 Shiga-toxin-producing *Escherichia coli* (STECs) of clinical importance. Experimental evaluation of the contribution of these homologs to adherence of these non-O157 STEC, *E. coli* O26:H11, O103:H2 and O111:NM, are underway.

**Table 1:** Bioinformatically determined putative adhesins in the O157 DMEM-Proteome

**Protein Identity: Sequences not homologous [O-island] OR homologous [Backbone] to*E. coli*K12 MG1655**	**Peptide Hits**	**Bacterial Cell Localization**	**Proteins identified by PELS**^**1**^	**Proteins identified by IVIAT**^**2**^	**Proteins associated with O157 viulence**
Tir: Translocated intimin receptor protein: **O-island#148**	3	Extracellular			**+**
Eae: Intimin: Adhesin; attaching and effacing protein: **O-island #148**	36	Outer Membrane			**+**
ChuA: Outer membrane heme/hemoglobin receptor; heme utilization/transport protein: **O-island #140**	13	Outer Membrane		**+**	**+**
EspP: Serine protease; secreted autotransporter: **pO157**	6	Outer Membrane	**+**		**+**
TerE: Putative tellurium resistance protein B (Putative phage inhibition, colicin resistance): **O-island #43**	35	Cytoplasm			
Z2239: Putative outer membrane porin protein: **O-island #62**	2	Outer Membrane			
FimG: Fimbrial Morphology: **Backbone**	1	Inner Membrane			
MsrA: Peptide methionine sulfoxide reductase : **Backbone**	1	Cytoplasm			
YjeI: Putative lipoprotein :**Backbone**	2	Non-cytoplasmic			
BtuB: Outer membrane receptor for transport of vitamin B12, E colicins, and bacteriophage: **Backbone**	3	Outer Membrane			
DsbA: Thiol:disulfide interchange protein precursor : **Backbone**	8	Periplasm			
PstS: High-affinity phosphate-specific transport system; periplasmic phosphate-binding protein: **Backbone**	3	Periplasm			
YiaF: Hypothetical protein: **Backbone**	2	Cytoplasm			
Slp: Outer membrane protein induced after carbon starvation: **Backbone**	17	Outer Membrane			
LivJ: Leu/Ile/Val-binding protein precursor; ABC transporter: **Backbone**	2	Periplasm			
PpiA: Peptidyl-prolyl cis-trans isomerase A precursor : **Backbone**	4	Cytoplasm			
YrbD: Hypothetical protein precursor; probable phospholipid ABC transporter-binding protein MlaD :**Backbone**	1	Periplasm			
YraP: Hypothetical protein; putative transport :**Backbone**	2	Periplasm			
TolC: Outer membrane channel; specific tolerance to colicin E1; segregation of daughter chromosome: **Backbone**	3	Outer Membrane	**+**		
Lpp: Murein-lipoprotein; major outer membrane lipoprotein precursor: **Backbone**	31	Outer Membrane	**+**	**MepA, MltB, Slt**	
NlpB: Lipoprotein-34: **Backbone**	3	Outer Membrane			
Z3508: Hypothetical protein :**Backbone**	1	Non-cytoplasmic			
OmpC: Outer membrane protein 1b: hyperosmotic shock: **Backbone**	123	Outer Membrane	**+**		
YehZ: Putative transport system permease protein :**Backbone**	1	Cytoplasm			
CspC: Cold shock-like protein : **Backbone**	25	Cytoplasm			
CspD: Cold shock-like protein : **Backbone**	1	Cytoplasm			
CspE: Cold shock-like protein : **Backbone**	9	Cytoplasm			
YeaF: Hypothetical protein yeaF; putative scaffolding protein in the formation of a murein :**Backbone**	4	Outer Membrane	**+**	**YeaA**	
OsmE: Osmotically inducible lipoprotein E precursor; activator of ntr-like gene : **Backbone**	9	Cytoplasm			
SlyB: Outer membrane lipoprotein slyB precursor : **Backbone**	3	Outer Membrane			
YciD: Putative outer membrane protein :**Backbone**	1	Outer Membrane			
YceI: Putative GTP binding :**Backbone**	2	Non-cytoplasmic			
ArtI: Arginine 3rd transport system periplasmic binding protein: **Backbone**	1	Periplasm			
YbiS: Putative transpeptidase :**Backbone**	5	Non-cytoplasmic			
OmpX: Outer membrane protein X precursor : **Backbone**	22	Outer Membrane			
FepA: Receptor for ferric enterobactin (Enterochelin) and colicins B and D: **Backbone**	29	Outer Membrane	**+**		

^1^PELS: Proteomics- based Expression Library Screening

^2^IVIAT:In Vivo-Induced Antigen Technology

## Conclusion

Proteins other than the LEE-encoded proteins are involved in O157 adherence to RSE cells at the bovine RAJ. Such proteins, with adhesin potential, are expressed by this human pathogen during growth in vitro, in DMEM. Three such putative adhesins, namely, ChuA, TerE, and a putative outermembrane porin protein on O-island #62, are currently under evaluation for their adherence potential. Such studies should provide both valuable insights into the O157-RSE interactions and new targets for more efficacious anti-adhesion O157 cattle vaccines.

## Competing interests

The authors declare no competing financial interests.

## Authors’ contributions

ITK was the project leader and designed, coordinated, obtained funding, conducted experiments, analyzed data and drafted the manuscript. RWG conducted experiments and tabulated data. BK and DAS performed proteomic analysis. SBC assisted in design and participated in helpful discussions. MJ was the co-project leader, and designed, coordinated, analyzed results and performed bioinformatic analysis. All authors read and approved the final manuscript.

## Supplementary Material

Additional file 1 http://www.biomedcentral.com/imedia/9899042126754199/supp1.pdf. TABLE A Quantitation of RSE cells with adherent bacteria in the presence of D + mannose. Click here for file

Additional file 2 http://www.biomedcentral.com/imedia/6766700936754199/supp2.pdf. TABLE B Quantitation of HEp-2 cells with adherent bacteria in the presence of D + mannose. Click here for file

Additional file 3 http://www.biomedcentral.com/imedia/1105071156754199/supp3.pdf. TABLE C Uncharacterized hypothetical proteins of the O157 DMEM-Proteome. Click here for file

Additional file 4 http://www.biomedcentral.com/imedia/1751063870675419/supp4.pdf. TABLE D Previously characterized proteins of the O157 DMEM-Proteome. Click here for file

Additional file 5 http://www.biomedcentral.com/imedia/1777785157675419/supp5.pdf. DATA SHEETS: O157-DMEM MS/MS data sheet 1. Click here for file

Additional file 6 http://www.biomedcentral.com/imedia/1707955235675419/supp6.pdf. DATA SHEETS: O157-DMEM MS/MS data sheet 2. Click here for file

Additional file 7 http://www.biomedcentral.com/imedia/1451425738675419/supp7.pdf. DATA SHEETS: O157-DMEM MS/MS data sheet 3. Click here for file

Additional file 8 http://www.biomedcentral.com/imedia/3116488396754199/supp8.pdf. DATA SHEETS: O157-DMEM MS/MS data sheet 4. Click here for file

Additional file 9 http://www.biomedcentral.com/imedia/1233524502675419/supp9.pdf. DATA SHEETS: O157-DMEM MS/MS data sheet 5. Click here for file

Additional file 10 http://www.biomedcentral.com/imedia/1610501146675419/supp10.pdf. DATA SHEETS: O157-DMEM MS/MS data sheet 6. Click here for file

Additional file 11 http://www.biomedcentral.com/imedia/1326109329675419/supp11.pdf. DATA SHEETS: O157-DMEM MS/MS data sheet 7. Click here for file

Additional file 12 http://www.biomedcentral.com/imedia/1285024576754199/supp12.pdf. DATA SHEETS: O157-DMEM MS/MS data sheet 8. Click here for file
