# Supplementary material for: Proteins other than the locus of enterocyte effacement-encoded proteins contribute to Escherichia coli O157:H7 adherence to bovine rectoanal junction stratified squamous epithelial cells
Source: BMC Microbiol. 2012 Jun 12;12:103. doi: 10.1186/1471-2180-12-103 (PMC3420319; doi:10.1186/1471-2180-12-103)
Supplement: Additional file 1 — http://www.biomedcentral.com/imedia/9899042126754199/supp1.pdf. TABLE A Quantitation of RSE cells with adherent bacteria in the presence of D + mannose. [file 1471-2180-12-103-S1.pdf]

Table A. Quantitation of RSE cells with adherent bacteria in the presence of D+mannose.

Bacteria Tested	Bacterial Adherence Pattern	RSE cells with adherent bacteria, in the ranges shown, for two different trials[1] (MOI[2] = 10^6 bacteria:10^5 RSE cells)				Percent Mean +/- standard error of mean, of RSE cells with adherent bacteria in the ranges shown[5]	
		Trial I		Trial II			
		**>10**	**1-10[3]**	**>10**	**1-10**	**>10**	**1-10**
O157 strain 933 + No antisera	Aggregative, Moderate	52 (156)[4]	92 (156)	0 (160)	131 (160)	16.5 ± 16.5	**70.5 ± 11.5**
O157 strain 933 + Pooled sera	Aggregative, Moderate	87 (159)	65 (159)	23 (83)	58 (83)	41.5 ± 13.5	**55.5 ± 14.5**
O157 strain 933 + Anti-Intimin antisera	Aggregative, Moderate	20 (133)	107 (133)	80 (160)	77 (160)	32.5 ± 17.5	**64 ± 16**
O157 strain 86-24 (Sm^r; Intimin-positive)	Diffuse, Strong	106 (155)	28 (155)	110 (120)	10 (120)	**80 ± 12**	13 ± 5
O157 strain 86-24*eae* Δ10 (Sm^r; Nal^r; Intimin-negative)	Diffuse, Strong	98 (137)	38 (137)	40 (70)	30 (70)	**64.5 ± 7.5**	35.5 ± 7.5
O157 86-24*eae* Δ10 (pEB310) (Amp^r; Cam^r; Intimin-positive)	Diffuse, Strong	120 (142)	22 (142)	90 (110)	20 (110)	**83.5 ± 1.5**	16.5 ± 1.5

[1] Each trial had one slide per bacterial group. Each slide in turn had 8 technical replicates spotted on it; 10-20 well dispersed RSE cells were evaluated per spot.

[2] MOI, multiplicity of infection.

3 Number of bacteria adhering to each RSE cell is shown as a range of >10, and 1-10. Number of RSE cells without bacteria is not shown.

[4]Total number of RSE cells evaluated in each trial is shown in parenthesis.

[5] Percent means for ranges used to determine “moderate or strong” adherence are in bold.
