# Supplementary material for: Proteins other than the locus of enterocyte effacement-encoded proteins contribute to Escherichia coli O157:H7 adherence to bovine rectoanal junction stratified squamous epithelial cells
Source: BMC Microbiol. 2012 Jun 12;12:103. doi: 10.1186/1471-2180-12-103 (PMC3420319; doi:10.1186/1471-2180-12-103)
Supplement: Additional file 2 — http://www.biomedcentral.com/imedia/6766700936754199/supp2.pdf. TABLE B Quantitation of HEp-2 cells with adherent bacteria in the presence of D + mannose. [file 1471-2180-12-103-S2.pdf]

Table B. Quantitation of HEp-2 cells with adherent bacteria in the presence of D+mannose.

**Bacteria Tested**	**Bacterial Adherence Pattern**	**HEp-2 cells with adherent bacteria, in the ranges shown, for two different trials[1] (MOI[2] = 10^6 bacteria:10^5 RSE cells)**				**Percent Mean +/- standard error of mean, of HEp-2 cells with adherent bacteria in the ranges shown[5]**	
		**Trial I**		**Trial II**			
		**>10**	**1-10[3]**	**>10**	**1-10**	**>10**	**1-10**
O157 strain 933 + No antisera	Diffuse, Moderate	27 (100)[4]	68 (100)	21 (100)	55 (100)	24 ±3.0	**61.5 ±6.5**
O157 strain 933 + Pooled sera	Non-adherent	0 (120)	44 (120)	0 (120)	43 (120)	0	**36. 5 ±0.5**
O157 strain 933 + Anti-Intimin antisera	Non-adherent	2 (120)	52 (120)	0 (40)	18 (40)	1 ±1.0	**44 ±1.0**
O157 strain 86-24 (Intimin-positive)	Diffuse, Moderate	5 (110)	88 (110)	0 (60)	57 (60)	2.5 ±2.5	**87.5 ±7.5**
O157 strain 86-24*eae* Δ10 (Intimin-negative)	Non-adherent	0 (120)	50 (120)	0 (60)	21 (60)	0	**38.5 ±3.5**
O157 86-24*eae* Δ10 (pEB310) (Intimin-positive)	Diffuse, Moderate	0 (120)	94 (120)	0 (60)	48 (60)	0	**79 ±1.0**

[1]Each trial had one slide per bacterial group. Each slide in turn had 3-6 technical replicates on it in separate chambers; 10-20 well dispersed HEp-2 cells were evaluated per chamber.
[2]MOI, multiplicity of infection.
[3] Number of bacteria adhering to each HEp-2 cell is shown as a range of >10, and 1-10. Number of HEp-2 cells without bacteria is not shown.
[4] Total number of HEp-2 cells evaluated in each trial is shown in parenthesis.
[5] Percent means for ranges used to determine "moderate or non-adherent" adherence are in bold.
