# Supplementary material for: Proteins other than the locus of enterocyte effacement-encoded proteins contribute to Escherichia coli O157:H7 adherence to bovine rectoanal junction stratified squamous epithelial cells
Source: BMC Microbiol. 2012 Jun 12;12:103. doi: 10.1186/1471-2180-12-103 (PMC3420319; doi:10.1186/1471-2180-12-103)
Supplement: Additional file 3 — http://www.biomedcentral.com/imedia/1105071156754199/supp3.pdf. TABLE C Uncharacterized hypothetical proteins of the O157 DMEM-Proteome. [file 1471-2180-12-103-S3.pdf]

Table C. Uncharacterized Hypothetical Proteins of the O157 DMEM-Proteome.

Protein: Sequences homologous [Backbone] or not homologous [O-island] to *E. coli* K12 MG1655	Peptide Hits	Bacterial Cell Localization	Proteins identified by PELS[1]	Proteins identified by IVIAT[2]
YbeF: Hypothetical protein ; signal peptide :**Backbone**	1	Extracellular		
YaeT: UP05_ECOLI (P39170) Putative Outer membrane protein assembly factor yaeT/ precursor :**Backbone**	15	Outer Membrane		
YeaF: Hypothetical protein yeaF; putative scaffolding protein in the formation of a murein :**Backbone**	4	Outer Membrane	+	**YeaA**
Z2239: Putative outer membrane porin protein : **O-island #62**	2	Outer Membrane		
YccZ: Putative polysaccharide export protein precursor :**Backbone**	2	Outer Membrane		
YfiO: Hypothetical lipoprotein precursor :**Backbone**	2	Outer Membrane		
Fiu: Putative outer membrane receptor for iron transport :**Backbone**	1	Outer Membrane		
YciD: Putative outer membrane protein :**Backbone**	1	Outer Membrane		
YiaD: Putative outer membrane protein :**Backbone**	1	Outer Membrane		
ChuT: Putative periplasmic hemin binding protein :**Backbone**	7	Periplasm		
YcdO: Hypothetical protein; putative iron transport :**Backbone**	7	Periplasm		
FliY: Putative periplasmic binding transport protein :**Backbone**	3	Periplasm		
YbhE: Hypothetical protein (6-phosphogluconolactone) :**Backbone**	2	Periplasm		
YraP: Hypothetical protein; putative transport :**Backbone**	2	Periplasm		
Z4846: Hypothetical lipoprotein :**Backbone**	1	Periplasm		
YggE: Hypothetical protein; putative actin :**Backbone**	1	Periplasm		
YrbD: Hypothetical protein precursor ; probable phospholipid ABC transporter-binding protein MlaD :**Backbone**	1	Periplasm		
MdtF: Putative transport system permease protein :**Backbone**	4	Inner Membrane		
MdtE: Putative membrane protein :**Backbone**	3	Inner Membrane		
YbaU: Putative protease maturation protein :**Backbone**	3	Inner Membrane		
YajC: Hypothetical protein; translocase :**Backbone**	3	Inner Membrane		
YjiY: Putative carbon starvation protein :**Backbone**	2	Inner Membrane		
YojN: Putative 2-component sensor protein :**Backbone**	1	Inner Membrane		
YagU: Hypothetical protein :**Backbone**	1	Inner Membrane		
YhcB: Hypothetical protein; cytochrome d ubiquinol oxidase subunit III :**Backbone**	1	Inner Membrane		
YncE: Hypothetical protein precursor; pre-mRNA processing and cytoskeleton assembly :**Backbone**	49	Non-cytoplasmic	+	
Z1498: Hypothetical protein H0152 :**Backbone**	7	Non-cytoplasmic		
Z2603: Hypothetical protein :**Backbone**	6	Non-cytoplasmic		
YbiS: Putative transpeptidase :**Backbone**	5	Non-cytoplasmic		
Z0955: Hypothetical protein; possible outer membrane :**Backbone**	3	Non-cytoplasmic	+	
YahO: Hypothetical protein :**Backbone**	2	Non-cytoplasmic		
Z0372: Hypothetical protein :**Backbone**	2	Non-cytoplasmic		
YrbC: Hypothetical protein in prophage CP-933K: **O-island #36**	2	Non-cytoplasmic		
YjeI: Putative lipoprotein :**Backbone**	2	Non-cytoplasmic		
YceI: Putative GTP binding :**Backbone**	2	Non-cytoplasmic		
Z2887: Hypothetical protein :**Backbone**	1	Non-cytoplasmic		
Z3508: Hypothetical protein :**Backbone**	1	Non-cytoplasmic		
YgiM: Putative signal transduction protein :**Backbone**	1	Non-cytoplasmic		
TerE: Putative tellurium resistance protein B (Putative phage inhibition, colicin resistance) :**O-island #43**	35	Cytoplasm		
TerD: Putative phage inhibition, colicin resistance and tellurite resistance protein TerD : **O-island #43**	25	Cytoplasm		
YfiD: Putative formate acetyltransferase :**Backbone**	22	Cytoplasm		
TerZ: Putative phage inhibition, colicin resistance and tellurite resistance protein TerZ: **O-island #43**	18	Cytoplasm		
ChuS: Putative heme/hemoglobin transport protein :**Backbone**	17	Cytoplasm		
YfbU: Hypothetical protein :**Backbone**	11	Cytoplasm		
Z3775: Putative dehydrogenase :**Backbone**	9	Cytoplasm		
YjjK: ABC transporter ATP-binding protein :**Backbone**	9	Cytoplasm		
YajQ: Hypothetical protein :**Backbone**	8	Cytoplasm		
YjgF: Putative endoribonuclease protein :**Backbone**	7	Cytoplasm		
YajG: Putative polymerase/proteinase :**Backbone**	7	Cytoplasm		
YcaC: Putative isochorismatase family protein :**Backbone**	7	Cytoplasm		
YqjD: Hypothetical protein :**Backbone**	7	Cytoplasm	+	
Z3719: Putative multimodular enzyme :**Backbone**	6	Cytoplasm		
YeeX: Hypothetical protein :**Backbone**	6	Cytoplasm		
Z3260: Hypothetical protein :**Backbone**	5	Cytoplasm		
YcfF: HIT-like protein :**Backbone**	5	Cytoplasm		

YihD: Putative fructose bisphosphate hydrolase :**Backbone**	5	Cytoplasm	
Ypt2: Hypothetical 31.7 kD protein in traX-fino intergenic region : **pO157**	4	Cytoplasm	
ChuY: Hypothetical protein chuY :**Backbone**	4	Cytoplasm	
YbjP: Putative enzyme :**Backbone**	4	Cytoplasm	
VacB: Putative enzyme :**Backbone**	4	Cytoplasm	
YeaD: Putative aldose 1-epimerase :**Backbone**	4	Cytoplasm	
YfiA: Putative ribosome-associated inhibitor A :**Backbone**	4	Cytoplasm	
YifE: Hypothetical protein :**Backbone**	4	Cytoplasm	
NifU: Z3796; Iron-sulfur cluster scaffold-like proteins; nifU-like : **Backbone**	3	Cytoplasm	
ElaB: ElaB protein : **Backbone**	3	Cytoplasm	
Bor: Bor protein of BP-933W : **Backbone**	3	Cytoplasm	
TerB: Putative phage inhibition, colicin resistance and tellurite resistance protein (TerB) : **O-island #43**	3	Cytoplasm	
Z3306: Hypothetical protein in prohage CP-933V : **O-island #93**	3	Cytoplasm	
Z3073: Hypothetical protein in prophage CP-933U: **O-island #79**	3	Cytoplasm	
Z2694: Hypothetical protein :**Backbone**	3	Cytoplasm	
Z2386: Hypothetical protein :**Backbone**	3	Cytoplasm	
Z5898: Hypothetical protein :**Backbone**	3	Cytoplasm	
YciL: Hypothetical protein :**Backbone**	3	Cytoplasm	
Z3606: Hypothetical protein :**Backbone**	3	Cytoplasm	
YggL: Hypothetical protein :**Backbone**	3	Cytoplasm	
Z1930: Putative protease encoded within prophage CP-933X :**Backbone**	3	Cytoplasm	
BolA: Possible regulator of murein genes (Putative regulator of murein genes) :**Backbone**	3	Cytoplasm	
YajO: Putative NAD(P)H-dependent xylose reductase :**Backbone**	3	Cytoplasm	
YaeH: Hypothetical protein :**Backbone**	3	Cytoplasm	
YbaB: Hypothetical protein :**Backbone**	3	Cytoplasm	
YbeD: Hypothetical protein :**Backbone**	3	Cytoplasm	
YchN: Hypothetical protein :**Backbone**	3	Cytoplasm	
YsgA: Putative carboxymethylenebutenolidase; dienelactone hydrolase : **Backbone**	2	Cytoplasm	
YGGX_ECOLI (P52065) UPF0269 protein yggX :**Backbone**	2	Cytoplasm	
YbbN: Putative thioredoxin-like protein :**Backbone**	2	Cytoplasm	
ChuX: Hypothetical protein (ShuX-like protein) :**Backbone**	2	Cytoplasm	
ChuW: Putative oxygen independent coproporphyrinogen III oxidase :**Backbone**	2	Cytoplasm	
YnhD: Putative ATP-binding component of a transport system :**Backbone**	2	Cytoplasm	
Z0377: Putative dehydrogenase : **O-island #12**	2	Cytoplasm	
YliJ: Putative transferase :**Backbone**	2	Cytoplasm	
YbhB: Hypothetical protein :**Backbone**	2	Cytoplasm	
YadF: Putative carbonic anhdrase :**Backbone**	2	Cytoplasm	
YcdY: Putative oxidoreductase component :**Backbone**	2	Cytoplasm	
Z3776: Hypothetical protein Z3776 :**Backbone**	2	Cytoplasm	
Z3696: Hypothetical protein :**Backbone**	2	Cytoplasm	
YqhD: Putative oxidoreductase :**Backbone**	2	Cytoplasm	
YecA: Hypothetical protein yecA :**Backbone**	2	Cytoplasm	
YgfZ: Hypothetical protein :**Backbone**	2	Cytoplasm	
Z1317: Hypothetical protein :**Backbone**	2	Cytoplasm	
YbeL: Hypothetical protein :**Backbone**	2	Cytoplasm	
YbeY: Hypothetical protein :**Backbone**	2	Cytoplasm	
YbgI: Hypothetical protein :**Backbone**	2	Cytoplasm	
YcgL: Hypothetical protein :**Backbone**	2	Cytoplasm	
YdcL: Hypothetical lipoprotein :**Backbone**	2	Cytoplasm	
YdhD: Probable monothiol glutaredoxin **:Backbone**	2	Cytoplasm	
YeaG: Hypothetical protein :**Backbone**	2	Cytoplasm	
YeeZ: Hypothetical protein :**Backbone**	2	Cytoplasm	
YfhP: Hypothetical protein :**Backbone**	2	Cytoplasm	
YiaF: Hypothetical protein :**Backbone**	2	Cytoplasm	
YibN: Hypothetical protein :**Backbone**	2	Cytoplasm	
YieF: Hypothetical protein :**Backbone**	2	Cytoplasm	**+**
YjbJ: Putative stress response protein :**Backbone**	1	Cytoplasm	
SbmC: Unknown function : **Backbone**	1	Cytoplasm	
PhnA: PhnA protein : **Backbone**	1	Cytoplasm	
CyaY: Hypothetical protein :**Backbone**	1	Cytoplasm	
DinI: DinI-like protein Z3305/ECs2939 in prophage CP-933V :**O-island #93**	1	Cytoplasm	
GloB: Probable hydroxyacylglutathione hydrolase :**Backbone**	1	Cytoplasm	
YbaS: Probable glutaminase :**Backbone**	1	Cytoplasm	
L7092: Hypothetical protein : **pO157**	1	Cytoplasm	

Z5104: Hypothetical protein :**Backbone**	1	Cytoplasm
YciE: Hypothetical protein :**Backbone**	1	Cytoplasm
YciF: Putative structural protein :**Backbone**	1	Cytoplasm
Z1903: Hypothetical protein encoded in prophage CP-933X : **O-island #52**	1	Cytoplasm
Z1456: Hypothetical protein in prohage BP-933W: **O-island #45**	1	Cytoplasm
Z3918: Chaperone-like protein :**Backbone**	1	Cytoplasm
Z1923: Hypothetical protein in CP-933X: **O-island #52**	1	Cytoplasm
YhiR: Hypothetical protein :**Backbone**	1	Cytoplasm
Z4875: Putative phosphotransferase system enzyme subunit :**Backbone**	1	Cytoplasm
YjbK: Putative regulator :**Backbone**	1	Cytoplasm
YehZ: Putative transport system permease protein :**Backbone**	1	Cytoplasm
Z1106: Putative enzyme (NADH oxidoreductase for the HCP) :**Backbone**	1	Cytoplasm
YhgF: Hypothetical protein :**Backbone**	1	Cytoplasm
YiiS: Hypothetical protein :**Backbone**	1	Cytoplasm
YciO: Hypothetical protein :**Backbone**	1	Cytoplasm
YheO: Hypothetical protein :**Backbone**	1	Cytoplasm
YgaU: Hypothetical protein :**Backbone**	1	Cytoplasm
Z1212: Hypothetical protein : **O-island #43**	1	Cytoplasm
Z1193: Hypothetical protein : **O-island #43**	1	Cytoplasm
TerC: Putative phage inhibition, colicin resistance and tellurite resistance protein : **O-island #43**	1	Cytoplasm
YncB: Putative oxidoreductase :**Backbone**	1	Cytoplasm
Z3787: Hypothetical protein :**Backbone**	1	Cytoplasm
YraM: Putative glycosylase :**Backbone**	1	Cytoplasm
YhaR: Hypothetical protein :**Backbone**	1	Cytoplasm
Z6025: Hypothetical protein in prophage CP-933P : **O-island #71**	1	Cytoplasm
WecE: Putative regulator :**Backbone**	1	Cytoplasm
Z3043: Hypothetical protein :**Backbone**	1	Cytoplasm
YjiC: Hypothetical protein :**Backbone**	1	Cytoplasm
Z2968: Hypothetical protein :**Backbone**	1	Cytoplasm
YbdB: Hypothetical protein :**Backbone**	1	Cytoplasm
YidB: Hypothetical protein :**Backbone**	1	Cytoplasm
Z2883: Hypothetical protein :**Backbone**	1	Cytoplasm
Z2879: Hypothetical protein :**Backbone**	1	Cytoplasm
Z2874: Putative regulator :**Backbone**	1	Cytoplasm
YfbT: Putative phosphatase :**Backbone**	1	Cytoplasm
YbaK: Hypothetical protein :**Backbone**	1	Cytoplasm
Z2852: Hypothetical protein :**Backbone**	1	Cytoplasm
YaeP: Hypothetical protein :**Backbone**	1	Cytoplasm
YbiC: Hypothetical oxidoreductase :**Backbone**	1	Cytoplasm
YbiT: Hypothetical ABC transporter ATP-binding protein :**Backbone**	1	Cytoplasm
YbjQ: Hypothetical protein :**Backbone**	1	Cytoplasm
YccJ: Hypothetical protein :**Backbone**	1	Cytoplasm
YciN: Hypothetical protein :**Backbone**	1	Cytoplasm
YdfZ: Hypothetical protein :**Backbone**	1	Cytoplasm
YdjA: Hypothetical protein :**Backbone**	1	Cytoplasm
YebC: Hypothetical protein :**Backbone**	1	Cytoplasm
YebG: Hypothetical protein :**Backbone**	1	Cytoplasm
YfhJ: Hypothetical protein :**Backbone**	1	Cytoplasm
YfiF: Hypothetical tRNA/rRNA methyltransferase :**Backbone**	1	Cytoplasm
YgaM: Hypothetical protein :**Backbone**	1	Cytoplasm
YggT: Hypothetical protein :**Backbone**	1	Cytoplasm
YqfB: Hypothetical protein :**Backbone**		

[1]PELS: Proteomics- based Expression Library Screening

[2]IVIAT:In Vivo-Induced Antigen Technology
