# Supplementary material for: Proteins other than the locus of enterocyte effacement-encoded proteins contribute to Escherichia coli O157:H7 adherence to bovine rectoanal junction stratified squamous epithelial cells
Source: BMC Microbiol. 2012 Jun 12;12:103. doi: 10.1186/1471-2180-12-103 (PMC3420319; doi:10.1186/1471-2180-12-103)
Supplement: Additional file 4 — http://www.biomedcentral.com/imedia/1751063870675419/supp4.pdf. TABLE D Previously characterized proteins of the O157 DMEM-Proteome. [file 1471-2180-12-103-S4.pdf]

Table D. Previoulsy characterized proteins of the O157 DMEM-Proteome.

**Protein: Sequences homologous [Backbone] or not homologous [O-island] to *E. coli* K12 MG1655**	**Peptide Hits**	**Bacterial Cell Localization**	**Proteins identified by PELS[1]**	**Proteins identified by IVIAT[2]**	**Proteins associated with O157 viulence**
EspB: Secreted protein EspB : **O-island #148**	5	Extracellular			+
Tir: Translocated intimin receptor protein: **O-island#148**	3	Extracellular			+
OmpC: Outer membrane protein 1b: hyperosmotic shock: **Backbone**	123	Outer Membrane	+		
OmpA: Outer membrane protein 3a: adhesin : **Backbone**	62	Outer Membrane	+		
Iha: Adhesin: **O-island # 43**	42	Outer Membrane	+		+
OmpF: Outer membrane protein 1a: **Backbone**	38	Outer Membrane			+
Eae: Intimin: Adhesin; attaching and effacing protein: **O-island #148**	36	Outer Membrane			+
Lpp: Murein-lipoprotein; major outer membrane lipoprotein precursor: **Backbone**	31	Outer Membrane	+	**MepA, MltB, Slt**	
FepA: Receptor for ferric enterobactin (Enterochelin) and colicins B and D: **Backbone**	29	Outer Membrane	+		
OmpT: Outer membrane protein 3B; protease VII precursor: **Backbone**	24	Outer Membrane	+		
OmpX: Outer membrane protein X precursor : **Backbone**	22	Outer Membrane			
CirA: Outer membrane receptor for iron-regulated colicin I receptor; porin; requires tonB: **Backbone**	20	Outer Membrane	+	+	
Slp: Outer membrane protein induced after carbon starvation: **Backbone**	17	Outer Membrane			
FhuE: Outer membrane receptor for ferric iron uptake: **Backbone**	15	Outer Membrane	+	**FhuA**	
ChuA: Outer membrane heme/hemoglobin receptor; heme utilization/transport protein: **O-island #140**	13	Outer Membrane		+	+
Pal: Peptidoglycan-associated lipoprotein precursor : **Backbone**	12	Outer Membrane			
EspP: Serine protease; secreted autotransporter: **pO157**	6	Outer Membrane	+		+
LptD: Organic solvent tolerance protein precursor : **Backbone**	4	Outer Membrane			
BtuB: Outer membrane receptor for transport of vitamin B12, E colicins, and bacteriophage: **Backbone**	3	Outer Membrane			
NlpB: Lipoprotein-34: **Backbone**	3	Outer Membrane			
TolC: Outer membrane channel; specific tolerance to colicin E1; segregation of daughter chromosome: **Backbone**	3	Outer Membrane	+		
SlyB: Outer membrane lipoprotein slyB precursor : **Backbone**	3	Outer Membrane			
Blc: Outer membrane lipoprotein precursor : **Backbone**	1	Outer Membrane			
LolB: Outer-membrane lipoprotein precursor : **Backbone**	1	Outer Membrane			
RcsF: Regulator in colanic acid synthesis; interacts with RcsB: **Backbone**	1	Outer Membrane			
MdoG: Glucans biosynthesis protein G precursor: Backbone	14	Periplasm			
HdeA: Protein hdeA precursor (10K-S protein): **Backbone**	11	Periplasm			
GlnBP: Glutamine-binding periplasmic protein precursor: **Backbone**	10	Periplasm			
DsbA: Thiol:disulfide interchange protein precursor : **Backbone**	8	Periplasm			
HlpA: Histone-like protein HLP-1 precursor (DNA-binding 17 kDa protein) : **Backbone**	8	Periplasm			
OsmY: Hyperosmotically inducible periplasmic protein: **Backbone**	8	Periplasm			
Stx1B: Shiga toxin 1 B subunit encoded within prophage CP-933V: **O-island # 93**	7	Periplasm	+		+
DegP: Periplasmic serine endoprotease; heat shock protein: **Backbone**	6	Periplasm			
FkpA: FKBP-type peptidyl-prolyl cis-trans isomerase fkpA precursor: **Backbone**	6	Periplasm			
MalE: Maltose-binding periplasmic protein precursor : **Backbone**	6	Periplasm			
HisJ: Histidine-binding periplasmic protein precursor : **Backbone**	4	Periplasm			
PotD: Spermidine/putrescine periplasmic transport protein : **Backbone**	4	Periplasm			
LolA:Outer-membrane lipoprotein carrier protein precursor : **Backbone**	3	Periplasm			
MdoG: Glucans biosynthesis protein G precursor: **Backbone**	3	Periplasm			
PstS: High-affinity phosphate-specific transport system; periplasmic phosphate-binding protein: **Backbone**	3	Periplasm			
LivJ: Leu/Ile/Val-binding protein precursor; ABC transporter: **Backbone**	2	Periplasm			
BtuE: Glutathione peroxidase : **Backbone**	2	Periplasm			
MglB: Galactose-binding transport protein; receptor for galactose: **Backbone**	2	Periplasm			
FepB: Ferric enterobactin (Enterochelin) binding protein; periplasmic component: **Backbone**	2	Periplasm			
Dut: Deoxyuridine 5'-triphosphate nucleotidohydrolase: **Backbone**	1	Periplasm			
Eco: Ecotin precursor : **Backbone**	1	Periplasm			
IVY: Inhibitor of vertebrate lysozyme precursor : **Backbone**	1	Periplasm			
Stx2B: Shiga toxin 2 subunit B encoded by bacteriophage BP-933W: **O-island # 45**	1	Periplasm			+
KatP: EHEC-catalase/peroxidase: **pO157**	1	Periplasm			+
BglX: Beta-D-glucoside glucohydrolase, periplasmic: **Backbone**	1	Periplasm	+		
ArtI: Arginine 3rd transport system periplasmic binding protein: **Backbone**	1	Periplasm			
RbsB: D-ribose periplasmic binding protein: **Backbone**	1	Periplasm			
AgP: Periplasmic glucose-1-phosphatase : **Backbone**	1	Periplasm			
AtpB: ATP synthase beta chain: **Backbone**	59	Inner Membrane	+		
AtpA: ATP synthase alpha chain: **Backbone**	35	Inner Membrane	+		
Dps: DNA protection during starvation protein : **Backbone**	30	Inner Membrane	+		
HtpG: High temperature protein G; heat shock; chaperone : **Backbone**	19	Inner Membrane			
ManX: PTS system, mannose-specific IIAB component (EIIAB-Man): **Backbone**	16	Inner Membrane			
FtsH: Cell division protease: **Backbone**	13	Inner Membrane			
GadC: Probable glutamate/gamma-aminobutyrate antiporter (Extreme acid sensitivity protein): **Backbone**	11	Inner Membrane			
MetQ: D-methionine-binding lipoprotein metQ precursor : **Backbone**	10	Inner Membrane	+		
AtpF: ATP synthase B chain: **Backbone**	8	Inner Membrane			
EcnB: Entericidin B precursor: **Backbone**	8	Inner Membrane			
ManZ: PTS system, mannose-specific IID component (EIID-Man): **Backbone**	8	Inner Membrane			
SecA: Preprotein translocase; secretion protein: **Backbone**	7	Inner Membrane			
AtpG: ATP synthase gamma chain **Backbone**	5	Inner Membrane			
AcrB: Acridine efflux pump; protection responses : **Backbone**	5	Inner Membrane			
AtpD: ATP synthase delta chain: **Backbone**	4	Inner Membrane			
MinD: Septum site-determining protein; cell division inhibitor : **Backbone**	4	Inner Membrane			

MscS: Small conductance mechanosensitive channel; osmotic regulation : **Backbone**	4	Inner Membrane	+	
AtpE: ATP synthase epsilon chain: **Backbone**	3	Inner Membrane		
ExbB: Biopolymer transport protein : **Backbone**	3	Inner Membrane		
AcrA: Acriflavine resistance protein A precursor : B**ackbone**	2	Inner Membrane		
AtcU: Copper-transporting P-type ATPase: **Backbone**	2	Inner Membrane		
Etk: Tyrosine-protein kinase: B**ackbone**	2	Inner Membrane		
PspA: Phage shock protein A : **Backbone**	2	Inner Membrane	+	
Stx1A: Shiga toxin 1 subunit A encoded within prophage CP-933V: **O-island # 93**	2	Inner Membrane	+	+
FtsY: Cell division membrane protein : **Backbone**	2	Inner Membrane		
WzzB: Regulator of length of O-antigen component of lipopolysaccharide chains: **Backbone**	2	Inner Membrane		
ArcB: Aerobic respiration control sensor protein: **Backbone**	1	Inner Membrane	+	
CysQ: CysQ protein (Sulfur metabolism) : **Backbone**	1	Inner Membrane		
DacA: Penicillin-binding protein 5 precursor; D-alanyl-D-alanine carboxypeptidase fraction A: **Backbone**	1	Inner Membrane		
FtsK: DNA translocase: **Backbone**	1	Inner Membrane		
HflD: High frequency lysogenization protein: **Backbone**	1	Inner Membrane		
LolD: Lipoprotein releasing system ATP-binding protein: **Backbone**	1	Inner Membrane		
MetN: D-methionine transport ATP-binding protein: **Backbone**	1	Inner Membrane		
OxaA: Inner membrane protein oxaA : **Backbone**	1	Inner Membrane		
ManY: PTS system, mannose-specific IIC component (EIIC-Man): B**ackbone**	1	Inner Membrane		
FimG: Fimbrial Morphology: **Backbone**	1	Inner Membrane		
DacC: D-alanyl-D-alanine carboxypeptidase; penicillin-binding protein 6: **Backbone**	1	Inner Membrane		
MrcB: Peptidoglycan synthetase; penicillin-binding protein 1B: **Backbone**	1	Inner Membrane		
SecY: Preprotein translocase secY subunit : **Backbone**	1	Inner Membrane		
TolQ: TolQ protein: **Backbone**	1	Inner Membrane		
TufA: Protein chain elongation factor Tu: **Backbone**	153	Cytoplasm	+	
AdhE: Aldehyde-alcohol dehydrogenase: **Backbone**	148	Cytoplasm		
PflB: Formate acetyltransferase 1: **Backbone**	123	Cytoplasm		
GapA: Glyceraldehyde-3-phosphate dehydrogenase A: **Backbone**	114	Cytoplasm		
GadA: Glutamate decarboxylase alpha: **Backbone**	110	Cytoplasm		
FusA: Elongation factor G (EF-G) : **Backbone**	102	Cytoplasm	+	
Eno: Enolase; 2-phospho-D-glycerate hydrolyase: B**ackbone**	88	Cytoplasm	+	
PgK: Phosphoglycerate kinase : **Backbone**	87	Cytoplasm	+	
MopA: 60 kDa chaperonin; protein Cpn60; groEL protein : **Backbone**	77	Cytoplasm	+	
AceE: Pyruvate dehydrogenase E1 component: **Backbone**	66	Cytoplasm		
RpsA: 30S ribosomal protein S1 : **Backbone**	65	Cytoplasm	+	
RpoB: DNA-directed RNA polymerase beta chain: **Backbone**	54	Cytoplasm	+	
AceF: Pyruvate dehydrogenase dihydrolipoyltransacetylase component: **Backbone**	50	Cytoplasm	+	
RpoC: DNA-directed RNA polymerase beta' chain : **Backbone**	47	Cytoplasm		
FbA: Fructose-bisphosphate aldolase class II: **Backbone**	46	Cytoplasm		
PtsI: PEP-protein phosphotransferase system enzyme I: **Backbone**	41	Cytoplasm		
DnaK: Chaperone protein dnaK; heat shock 70 kDa protein: **Backbone**	40	Cytoplasm		
RplL: 50S ribosomal protein L7/L12 (L8) : **Backbone**	39	Cytoplasm	+	
Crr: PTS system, glucose-specific IIA component: **Backbone**	38	Cytoplasm	+	
Tsf: Elongation factor Ts (EF-Ts) : **Backbone**	37	Cytoplasm		
RpsD: 30S ribosomal protein S4 : **Backbone**	37	Cytoplasm		
ClpB: Chaperone clpB :**Backbone**	31	Cytoplasm		
RplA: 50S ribosomal protein L1 : **Backbone**	31	Cytoplasm	+	
RplF: 50S ribosomal protein L6 : **Backbone**	31	Cytoplasm		
Tig: Trigger factor; cell division: **Backbone**	31	Cytoplasm	+	
AhpC: Alkyl hydroperoxide reductase subunit C: **Backbone**	30	Cytoplasm		
RplX: 50S ribosomal protein L24 : **Backbone**	27	Cytoplasm		
RpoA: DNA-directed RNA polymerase alpha chain: **Backbone**	27	Cytoplasm		
Pta: Phosphotransacetylase : **Backbone**	26	Cytoplasm		
TktA: Transketolase 1 isozyme : **Backbone**	26	Cytoplasm		
CarB: Carbamoyl-phosphate synthase large chain : **Backbone**	25	Cytoplasm		
CspC: Cold shock-like protein : **Backbone**	25	Cytoplasm		
RpsC: 30S ribosomal protein S3 : **Backbone**	25	Cytoplasm		
RpsE: 30S ribosomal protein S5 : **Backbone**	25	Cytoplasm	+	
PurH: Bifunctional purine biosynthesis protein: **Backbone**	24	Cytoplasm		
RplE: 50S ribosomal protein L5 : **Backbone**	24	Cytoplasm		
GpmA: 2,3-bisphosphoglycerate-dependent phosphoglycerate mutase : **Backbone**	23	Cytoplasm	+	
PgI: Glucose-6-phosphate isomerase : **Backbone**	22	Cytoplasm		
Pnp: Polynucleotide phosphorylase; cytidylate kinase activity : **Backbone**	22	Cytoplasm		
AlaS: Alanyl-tRNA synthetase : **Backbone**	22	Cytoplasm		
Tpx: Thiol peroxidase : **Backbone**	22	Cytoplasm	+	
PurL: Phosphoribosylformylglycinamidine synthase : **Backbone**	21	Cytoplasm		
RplI: 50S ribosomal protein L9 : **Backbone**	21	Cytoplasm	+	
RpsG: 30S ribosomal protein S7 : **Backbone**	21	Cytoplasm		
RpsB: 30S ribosomal protein S2 : **Backbone**	19	Cytoplasm		
RpmC: 50S ribosomal protein L29 : **Backbone**	18	Cytoplasm		
LpdA: Dihydrolipoyl dehydrogenase: **Backbone**	17	Cytoplasm	+	
InfB: Translation initiation factor IF-2 : **Backbone**	17	Cytoplasm		
GuaB: Inosine-5'-monophosphate dehydrogenase : **Backbone**	17	Cytoplasm		
Mdh: Malate dehydrogenase : **Backbone**	17	Cytoplasm		

RplB: 50S ribosomal protein L2 : **Backbone**	17	Cytoplasm			
RplV: 50S ribosomal protein L22 : **Backbone**	17	Cytoplasm			
EntF: Enterobactin synthetase component F : **Backbone**	16	Cytoplasm			
FtsZ: Cell division protein : **Backbone**	16	Cytoplasm			
GlyA: Serine hydroxymethyltransferase : **Backbone**	16	Cytoplasm			
PfkA: 6-phosphofructokinase isozyme I : **Backbone**	16	Cytoplasm			
RplY: 50S ribosomal subunit protein L25 : **Backbone**	16	Cytoplasm			
RpsM: 30S ribosomal protein S13 : **Backbone**	16	Cytoplasm			
Hns: DNA-binding protein H-NS; histone-like protein HLP-II : **Backbone**	15	Cytoplasm	**+**		
PykF: Pyruvate kinase I : **Backbone**	15	Cytoplasm			
RplM: 50S ribosomal protein L13 : **Backbone**	15	Cytoplasm			
PtsO: Phosphocarrier protein HPr; histidine-containing protein : **Backbone**	14	Cytoplasm			
RplC: 50S ribosomal protein L3 : **Backbone**	14	Cytoplasm	**+**		
RpsJ: 30S ribosomal protein S10 : **Backbone**	14	Cytoplasm			
RpsF: 30S ribosomal protein S6 : **Backbone**	14	Cytoplasm			
AcpP: Acyl carrier protein; cytosolic activating factor: **Backbone**	13	Cytoplasm			
GroeS: 10 kDa chaperonin : B**ackbone**	13	Cytoplasm			
HupA: DNA-binding protein HU-alpha : **Backbone**	13	Cytoplasm			
GcvP: Glycine dehydrogenase : **Backbone**	13	Cytoplasm			
Gnd: Gluconate-6-phosphate dehydrogenase; decarboxylating : **Backbone**	13	Cytoplasm	**+**		
FabG: 3-oxoacyl-[acyl-carrier-protein] reductase : **Backbone**	13	Cytoplasm			
Rne: RNase E, membrane attachment, mRNA turnover, maturation 5S RNA : **Backbone**	13	Cytoplasm			
DapD: 2,3,4,5-tetrahydropyridine-2-carboxylate N-succinyltransferase : **Backbone**	13	Cytoplasm			
IlvN: Isoleucine tRNA synthetase : **Backbone**	13	Cytoplasm			
RplJ: 50S ribosomal protein L10 (L8) : **Backbone**	13	Cytoplasm	**+**		
GadB: Glutamate decarboxylase beta : **Backbone**	12	Cytoplasm			
FabI: Enoyl-[acyl-carrier-protein] reductase [NADH] : **Backbone**	12	Cytoplasm			
IpyR: Inorganic pyrophosphatase : **Backbone**	12	Cytoplasm			
AdsS: Adenylosuccinate synthetase : **Backbone**	12	Cytoplasm			
Udp: Uridine phosphorylase : **Backbone**	12	Cytoplasm			
ValS: Valine tRNA synthetase : **Backbone**	12	Cytoplasm			
RplN: 50S ribosomal protein L14 : **Backbone**	12	Cytoplasm			
RplS: 50S ribosomal protein L19 : **Backbone**	12	Cytoplasm			
RpsK: 30S ribosomal protein S11 : **Backbone**	12	Cytoplasm			
SerC: Phosphoserine aminotransferase: **Backbone**	12	Cytoplasm			
TpiA: Triosephosphate isomerase : **Backbone**	12	Cytoplasm			
Ppc: Phosphoenolpyruvate carboxylase : **Backbone**	11	Cytoplasm			
Pnp: Purine nucleoside phosphorylase : **Backbone**	11	Cytoplasm			
Asd: Aspartate-semialdehyde dehydrogenase : **Backbone**	11	Cytoplasm			
InfC: Translation initiation factor IF-3 : **Backbone**	11	Cytoplasm	**+**		
AcnB: Aconitate hydrase B : **Backbone**	11	Cytoplasm			
AspC: Aspartate aminotransferase : **Backbone**	11	Cytoplasm			
TrxA: Thioredoxin 1 : **Backbone**	11	Cytoplasm	**+**	**Ggt**	
AckA: Acetate kinase : **Backbone**	10	Cytoplasm			
CysK: Cysteine synthase A : **Backbone**	10	Cytoplasm			
PrsA: Ribose-phosphate pyrophosphokinase : **Backbone**	10	Cytoplasm			
MetE: 5-methyltetrahydropteroyltriglutamate--homocysteine methyltransferase : **Backbone**	10	Cytoplasm			
PurM: Phosphoribosylformylglycinamidine cyclo-ligase : **Backbone**	10	Cytoplasm			
PurC: Phosphoribosylaminoimidazole-succinocarboxamide synthase : **Backbone**	10	Cytoplasm	**+**	**purD**	
Per: Perosamine synthetase : **Backbone**	10	Cytoplasm			
CydA: Cytochrome d terminal oxidase, polypeptide subunit I : **Backbone**	10	Cytoplasm			
Lon: DNA-binding, ATP-dependent protease La; heat shock K-protein : **Backbone**	10	Cytoplasm			
RplD: 50S ribosomal protein L4 : **Backbone**	10	Cytoplasm	**+**		
RpsT: 30S ribosomal protein S20 : **Backbone**	10	Cytoplasm			
AspS: Aspartyl-tRNA synthetase : **Backbone**	10	Cytoplasm			
TalB: Transaldolase B : Backbone	10	Cytoplasm			
Crp: Catabolite gene activator; cAMP receptor protein : B**ackbone**	9	Cytoplasm			
CspE: Cold shock-like protein : **Backbone**	9	Cytoplasm			
GyrB: DNA gyrase subunit B : **Backbone**	9	Cytoplasm			
InfA: Translation initiation factor IF-1 : **Backbone**	9	Cytoplasm			
LuxS: S-ribosylhomocysteinase; autoinducer-2 production protein : **Backbone**	9	Cytoplasm			**+**
OsmE: Osmotically inducible lipoprotein E precursor; activator of ntr-like gene : **Backbone**	9	Cytoplasm			
IcdA: Isocitrate dehydrogenase, specific for NADP+ : **Backbone**	9	Cytoplasm			
RibH: 6,7-dimethyl-8-ribityllumazine synthase : **Backbone**	9	Cytoplasm			
RplO: 50S ribosomal subunit protein L15 : **Backbone**	9	Cytoplasm			
RplQ: 50S ribosomal protein L17 : **Backbone**	9	Cytoplasm			
RPOZ_ECOLI (P08374) DNA-directed RNA polymerase omega chain (EC 2.7.7.6) (RNAP omega subunit) : **Backbone**	9	Cytoplasm			
RRF_ECOLI (P16174) Ribosome recycling factor (Ribosome releasing factor) (Frr) : **Backbone**	9	Cytoplasm	**+**		
RpsP: 30S ribosomal protein S16 : **Backbone**	9	Cytoplasm			
RpsR: 30S ribosomal protein S18 : **Backbone**	9	Cytoplasm			
RpsS: 30S ribosomal protein S19 : **Backbone**	9	Cytoplasm			
RpsH: 30S ribosomal protein S8 : **Backbone**	9	Cytoplasm			
SodA: Superoxide dismutase [Mn] : **Backbone**	9	Cytoplasm			
ThrS: Threonyl-tRNA synthetase : **Backbone**	9	Cytoplasm			
TypA: GTP-binding protein tyrosine phosphorylated protein A : **Backbone**	9	Cytoplasm			

DapA: Dihydrodipicolinate synthase :**Backbone**	8	Cytoplasm	
EntE: Enterobactin synthetase component E : **Backbone**	8	Cytoplasm	
FldA: Flavodoxin 1 : **Backbone**	8	Cytoplasm	
GlmS: Glucosamine--fructose-6-phosphate aminotransferase [isomerizing] : **Backbone**	8	Cytoplasm	
NusG: Transcription antitermination protein : **Backbone**	8	Cytoplasm	
Gmd: GDP-mannose dehydratase : **Backbone**	8	Cytoplasm	
PrlC: Oligopeptidase A : **Backbone**	8	Cytoplasm	
ThrA: Aspartokinase I, homoserine dehydrogenase I : **Backbone**	8	Cytoplasm	
PepN: Aminopeptidase N : **Backbone**	8	Cytoplasm	
GyrA: DNA gyrase, subunit A, type II topoisomerase : **Backbone**	8	Cytoplasm	+
PpsA: Phosphoenolpyruvate synthase : **Backbone**	8	Cytoplasm	
RplU: 50S ribosomal protein L21 : **Backbone**	8	Cytoplasm	
RpmE: 50S ribosomal protein L31 : **Backbone**	8	Cytoplasm	
SecB: Protein-export protein secB : **Backbone**	8	Cytoplasm	
PheT: Phenylalanyl-tRNA synthetase beta chain : **Backbone**	8	Cytoplasm	
LysS: Lysyl-tRNA synthetase : **Backbone**	8	Cytoplasm	
TalB: Transaldolase B : **Backbone**	8	Cytoplasm	
CesT: Tir chaperone : **O-island # 148**	7	Cytoplasm	
ClpP: ATP-dependent Clp protease proteolytic subunit : **Backbone**	7	Cytoplasm	
GuaA: GMP synthase ; glutamine amidotransferase : **Backbone**	7	Cytoplasm	
MetK: S-adenosylmethionine synthetase : **Backbone**	7	Cytoplasm	
PepD: Aminoacyl-histidine dipeptidase : **Backbone**	7	Cytoplasm	
ProS: Proline tRNA synthetase : **Backbone**	7	Cytoplasm	
TktB: Transketolase 2 isozyme : **Backbone**	7	Cytoplasm	
RplK: 50S ribosomal protein L11 : **Backbone**	7	Cytoplasm	+
RplP: 50S ribosomal protein L16 : **Backbone**	7	Cytoplasm	
RpmA: 50S ribosomal protein L27 : **Backbone**	7	Cytoplasm	
RpmF: 50S ribosomal protein L32 : **Backbone**	7	Cytoplasm	
RpsL: 30S ribosomal protein S12 : **Backbone**	7	Cytoplasm	
RpsU: 30S ribosomal protein S21 : **Backbone**	7	Cytoplasm	
SerA: D-3-phosphoglycerate dehydrogenase : **Backbone**	7	Cytoplasm	
SodF: Superoxide dismutase [Fe] : **Backbone**	7	Cytoplasm	
GlyS: Glycyl-tRNA synthetase beta chain : **Backbone**	7	Cytoplasm	
Eda: KHG/KDPG aldolase ; 4-hydroxy-2-oxoglutarate aldolase : **Backbone**	6	Cytoplasm	
AsnA: Aspartate--ammonia ligase; asparagine synthetase A : **Backbone**	6	Cytoplasm	
CarA: Carbamoyl-phosphate synthase small chain : **Backbone**	6	Cytoplasm	
EntB: Isochorismatase lyase; 2,3 dihydro-2,3 dihydroxybenzoate synthase: **Backbone**	6	Cytoplasm	
FabZ: (3R)-hydroxymyristoyl-[acyl carrier protein] dehydratase : **Backbone**	6	Cytoplasm	
GlnA: Glutamine synthetase : **Backbone**	6	Cytoplasm	
GrpE: GrpE protein; HSP-70 cofactor; heat shock protein 24 : **Backbone**	6	Cytoplasm	+
HldD: ADP-L-glycero-D-manno-heptose-6-epimerase : **Backbone**	6	Cytoplasm	
AdK: Adenylate kinase; ATP-AMP transphosphorylase : **Backbone**	6	Cytoplasm	
NadE: NH(3)-dependent NAD(+) synthetase : **Backbone**	6	Cytoplasm	
NusA: Transcription elongation protein; N utilization substance protein A : **Backbone**	6	Cytoplasm	
PntA: Pyridine nucleotide transhydrogenase, alpha subunit : **Backbone**	6	Cytoplasm	
RpsO: 30S ribosomal subunit protein S15 : **Backbone**	6	Cytoplasm	
OsmC: Osmotically inducible protein : **Backbone**	6	Cytoplasm	
NrdA: Ribonucleoside diphosphate reductase 1, alpha subunit, B1 : **Backbone**	6	Cytoplasm	
RecA: Recombinase A : **Backbone**	6	Cytoplasm	
SspA: Stringent starvation protein A : **Backbone**	6	Cytoplasm	
SucD: Succinyl-CoA synthetase alpha chain : **Backbone**	6	Cytoplasm	
LeuS: Leucyl-tRNA synthetase : **Backbone**	6	Cytoplasm	+
TrxB: Thioredoxin reductase : **Backbone**	6	Cytoplasm	
Bcp: Bacterioferritin comigratory protein : **Backbone**	5	Cytoplasm	
ClpX: ATP-dependent Clp protease ATP-binding subunit : **Backbone**	5	Cytoplasm	
EntC: Isochorismate synthase :**Backbone**	5	Cytoplasm	
GalU: UTP--glucose-1-phosphate uridylyltransferase : **Backbone**	5	Cytoplasm	
GcvT: Aminomethyltransferase; glycine cleavage system T protein : **Backbone**	5	Cytoplasm	
GmhA: Phosphoheptose isomerase; sedoheptulose 7-phosphate isomerase : **Backbone**	5	Cytoplasm	
GpmI: 2,3-bisphosphoglycerate-independent phosphoglycerate mutase : **Backbone**	5	Cytoplasm	
HflC: Protease specific for phage lambda cII repressor: **Backbone**	5	Cytoplasm	
Hfq: Host factor-I protein for bacteriophage Q beta replication, a growth-related protein : **Backbone**	5	Cytoplasm	
Lrp: Leucine-responsive regulatory protein : **Backbone**	5	Cytoplasm	
PolA: DNA polymerase I, 3'--> 5' polymerase, 5'--> 3' and 3'--> 5' exonuclease : **Backbone**	5	Cytoplasm	
FabD: Malonyl-CoA-transacylase : **Backbone**	5	Cytoplasm	
MreB: Regulator of ftsI, penicillin binding protein 3, septation function : **Backbone**	5	Cytoplasm	
FklB: FKBP-type 22KD peptidyl-prolyl cis-trans isomerase; rotamase : **Backbone**	5	Cytoplasm	
PurF: Amidophosphoribosyltransferase = PRPP amidotransferase : **Backbone**	5	Cytoplasm	
RpmG: 50S ribosomal protein L33 : **Backbone**	5	Cytoplasm	
RpsQ: 30S ribosomal protein S17 : **Backbone**	5	Cytoplasm	
AsnS: Asparaginyl-tRNA synthetase : **Backbone**	5	Cytoplasm	
TrpS: Tryptophanyl-tRNA synthetase : **Backbone**	5	Cytoplasm	
TyrS: Tyrosyl-tRNA synthetase : **Backbone**	5	Cytoplasm	
Upp: Uracil phosphoribosyltransferase : **Backbone**	5	Cytoplasm	
AroG: Phospho-2-dehydro-3-deoxyheptonate aldolase : **Backbone**	4	Cytoplasm	

DksA: DnaK suppressor protein : **Backbone**	4	Cytoplasm		
GreA: Transcription elongation factor : **Backbone**	4	Cytoplasm		
Gst: Glutathione S-transferase : **Backbone**	4	Cytoplasm		
IhfA: Integration host factor alpha-subunit (IHF-alpha) : **Backbone**	4	Cytoplasm		
MinE: Cell division topological specificity factor : **Backbone**	4	Cytoplasm		
MtnN: MTA/SAH nucleosidase (P46) (5'-methylthioadenosine nucleosidase) : **Backbone**	4	Cytoplasm		
YbeZ: PhoH-like protein : **Backbone**	4	Cytoplasm		
PckA: Phosphoenolpyruvate carboxykinase : **Backbone**	4	Cytoplasm		
PpiA: Peptidyl-prolyl cis-trans isomerase A precursor : **Backbone**	4	Cytoplasm		
Qor: Quinone oxidoreductase : **Backbone**	4	Cytoplasm		
RpoD: RNA polymerase, sigma(70) factor; regulation of proteins induced at high temperatures : **Backbone**	4	Cytoplasm		
NfnB: Oxygen-insensitive NAD(P)H nitroreductase : **Backbone**	4	Cytoplasm		
GltB: Glutamate synthase, large subunit : **Backbone**	4	Cytoplasm		
RpmE: 50S ribosomal protein L31 type B-2 : **Backbone**	4	Cytoplasm		
RplT: 50S ribosomal protein L20 : **Backbone**	4	Cytoplasm		
RpmB: 50S ribosomal protein L28 : **Backbone**	4	Cytoplasm		
SucC: Succinyl-CoA synthetase beta chain : **Backbone**	4	Cytoplasm		
SerS: Seryl-tRNA synthetase : **Backbone**	4	Cytoplasm		
UspA: Universal stress protein A : **Backbone**	4	Cytoplasm		
UspG: Universal stress protein G : **Backbone**	4	Cytoplasm		
AccA: Acetyl-coenzyme A carboxylase carboxyl transferase subunit alpha : **Backbone**	3	Cytoplasm		
PepA: Leucine aminopeptidase: **Backbone**	3	Cytoplasm		
AroB: 3-dehydroquinate synthase : **Backbone**	3	Cytoplasm		
BccP: Biotin carboxyl carrier protein of acetyl-CoA carboxylase : **Backbone**	3	Cytoplasm		
CpxR: Transcriptional regulatory protein : **Backbone**	3	Cytoplasm		
CsrA: Carbon storage regulator : **Backbone**	3	Cytoplasm		
DeaD: Cold-shock DEAD-box protein A; ATP-dependent RNA helicase :**Backbone**	3	Cytoplasm	+	
Def: Peptide deformylase : **Backbone**	3	Cytoplasm		
FabA: 3-hydroxydecanoyl-[acyl-carrier-protein] dehydratase : **Backbone**	3	Cytoplasm		
FtnA: Ferritin 1 : **Backbone**	3	Cytoplasm		
FolE: GTP cyclohydrolase I : **Backbone**	3	Cytoplasm		
Gmd: GDP-mannose 4,6-dehydratase : **Backbone**	3	Cytoplasm		
Gne: UDP-N-acetylglucosamine 4-epimerase : **Backbone**	3	Cytoplasm		
GntY: Protein gntY (Fe/S cluster biosynthesis) : **Backbone**	3	Cytoplasm		
GpdA: Glycerol-3-phosphate dehydrogenase [NAD(P)+] : **Backbone**	3	Cytoplasm		
HisG: ATP phosphoribosyltransferase : **Backbone**	3	Cytoplasm		
IlvE: Branched-chain-amino-acid aminotransferase; transaminase B: **Backbone**	3	Cytoplasm		
KdsA: 2-dehydro-3-deoxyphosphooctonate aldolase : **Backbone**	3	Cytoplasm		
EmrR: Transcriptional repressor : **Backbone**	3	Cytoplasm		
MurQ: N-acetylmuramic acid -6-phosphate esterase : **Backbone**	3	Cytoplasm		
Ndk: Nucleoside diphosphate kinase : **Backbone**	3	Cytoplasm		
SucA: 2-oxoglutarate dehydrogenase E1 component : **Backbone**	3	Cytoplasm		
PntB: NAD(P) transhydrogenase subunit beta : **Backbone**	3	Cytoplasm		
ProB: Glutamate 5-kinase : **Backbone**	3	Cytoplasm		
ProQ: Osmotic effector of ProP; transport : **Backbone**	3	Cytoplasm		
PyrB: Aspartate carbamoyltransferase catalytic chain : **Backbone**	3	Cytoplasm		
PyrE: Orotate phosphoribosyltransferase : **Backbone**	3	Cytoplasm		
PyrG: CTP synthase : **Backbone**	3	Cytoplasm	+	+
PyrH: Uridylate kinase : **Backbone**	3	Cytoplasm		
FcI: Fucose synthetase : **Backbone**	3	Cytoplasm		
EntA: 2,3-dihydro-2,3-dihydroxybenzoate dehydrogenase, enterochelin biosynthesis : **Backbone**	3	Cytoplasm		
KatG: Catalase; hydroperoxidase HPI(I) : **Backbone**	3	Cytoplasm	+	
GltA: Citrate synthase : **Backbone**	3	Cytoplasm		
NagE: PTS system, N-acetylglucosamine-specific enzyme IIABC : **Backbone**	3	Cytoplasm		
YhbL: Sigma cross-reacting protein 27A (SCRP-27A) : **Backbone**	3	Cytoplasm		
LdhA: Fermentative D-lactate dehydrogenase, NAD-dependent : **Backbone**	3	Cytoplasm		
AsnB: Asparagine synthetase B : **Backbone**	3	Cytoplasm		
Prc: Carboxy-terminal protease for penicillin-binding protein 3 : **Backbone**	3	Cytoplasm		
PpiB: Peptidyl-prolyl cis-trans isomerase B (Rotamase B) : **Backbone**	3	Cytoplasm		
PurU: Formyltetrahydrofolate deformylase; for purT-dependent FGAR synthesis : **Backbone**	3	Cytoplasm		
KatE: Catalase; hydroperoxidase HPII(III) : **Backbone**	3	Cytoplasm		
AdhP: Alcohol dehydrogenase : **Backbone**	3	Cytoplasm		
RpmD: 50S ribosomal protein L30 : **Backbone**	3	Cytoplasm		
RpiA: Ribose-5-phosphate isomerase A : **Backbone**	3	Cytoplasm		
RpsN: 30S ribosomal protein S14 : **Backbone**	3	Cytoplasm		
RpsI: 30S ribosomal protein S9 : **Backbone**	3	Cytoplasm	+	
SelD: Selenide,water dikinase; selenophosphate synthetase : **Backbone**	3	Cytoplasm		
PheS: Phenylalanyl-tRNA synthetase alpha chain : **Backbone**	3	Cytoplasm		
GlyQ: Glycyl-tRNA synthetase alpha chain : **Backbone**	3	Cytoplasm		
GlnS: Glutaminyl-tRNA synthetase : **Backbone**	3	Cytoplasm		
TkrA: 2-ketogluconate reductase : **Backbone**	3	Cytoplasm		
AccD: Acetyl-coenzyme A carboxylase carboxyl transferase subunit beta : **Backbone**	2	Cytoplasm		
Apt: Adenine phosphoribosyltransferase : **Backbone**	2	Cytoplasm		
ArcA: Aerobic respiration control protein arcA : **Backbone**	2	Cytoplasm		
DbhB: DNA-binding protein HU-beta (NS1) (HU-1) : **Backbone**	2	Cytoplasm		

UpD: Uroporphyrinogen decarboxylase : **Backbone**	2	Cytoplasm		
efP: Elongation factor P : **Backbone**	2	Cytoplasm		
FabF: 3-oxoacyl-[acyl-carrier-protein] synthase II : **Backbone**	2	Cytoplasm		
GlnB: Nitrogen regulatory protein P-II 1 : **Backbone**	2	Cytoplasm		
Grx2: Glutaredoxin 2 : **Backbone**	2	Cytoplasm		
Grx3: Glutaredoxin 3 : **Backbone**	2	Cytoplasm		
Gsa: Glutamate-1-semialdehyde 2,1-aminomutase : **Backbone**	2	Cytoplasm		
HisA: Phosphoribosylformimino-5-aminoimidazole carboxamide ribotide isomerase: **Backbone**	2	Cytoplasm		
HisC: Histidinol-phosphate aminotransferase : **Backbone**	2	Cytoplasm		
HslU: ATP-dependent hsl protease ATP-binding subunit; heat shock protein : **Backbone**	2	Cytoplasm		
HimD: Integration host factor beta-subunit : **Backbone**	2	Cytoplasm		
IscS: Cysteine desulfurase : **Backbone**	2	Cytoplasm		
LpxA: Acyl-[acyl-carrier-protein]--UDP-N-acetylglucosamine O-acyltransferase : **Backbone**	2	Cytoplasm		
ManC: Mannose-1-phosphate guanylyltransferase [GDP] : **Backbone**	2	Cytoplasm		
MukB: Chromosome partition protein; structural maintenance of chromosome related protein : **Backbone**	2	Cytoplasm		
NagB: Glucosamine-6-phosphate deaminase : **Backbone**	2	Cytoplasm		
NusB: N utilization substance protein B : **Backbone**	2	Cytoplasm		
SucB: Dihydrolipoyllysine-residue succinyltransferase component of 2-oxoglutarate dehydorgenase : **Backbone**	2	Cytoplasm		
PlsB: Glycerol-3-phosphate acyltransferase : **Backbone**	2	Cytoplasm		
PtsG: PTS system, glucose-specific IIBC component (EIIBC-Glc) : **Backbone**	2	Cytoplasm		
PurE: Phosphoribosylaminoimidazole carboxylase catalytic subunit : **Backbone**	2	Cytoplasm		
PyrI: Aspartate carbamoyltransferase regulatory chain : **Backbone**	2	Cytoplasm		
WbdR: Acetyl transferase; O-antigen biosynthesis : **Backbone**	2	Cytoplasm		
RibE: Riboflavin synthase, alpha chain : **Backbone**	2	Cytoplasm		
ManA: Mannose-6-phosphate isomerase : **Backbone**	2	Cytoplasm		
PoxB: Pyruvate oxidase : **Backbone**	2	Cytoplasm		
MalP: Maltodextrin phosphorylase : **Backbone**	2	Cytoplasm		
Ndh: Respiratory NADH dehydrogenase : **Backbone**	2	Cytoplasm		
Pgm: Phosphoglucomutase : **Backbone**	2	Cytoplasm		
Q8XBT4 (Q8XBT4) Alkyl hydroperoxide reductase, F52a subunit; detoxification of hydroperoxides : **Backbone**	2	Cytoplasm		
Fbp: Fructose-bisphosphatase : **Backbone**	2	Cytoplasm		
PykA: Pyruvate kinase II, glucose stimulated : **Backbone**	2	Cytoplasm		
BglA: 6-phospho-beta-glucosidase A; cryptic : **Backbone**	2	Cytoplasm		
YbaY: Glycoprotein/polysaccharide metabolism : **Backbone**	2	Cytoplasm		
RbfA: Ribosome-binding factor A (P15B protein) : **Backbone**	2	Cytoplasm		
RcsB: Capsular synthesis regulator component B : **Backbone**	2	Cytoplasm		
YhbH: Probable sigma(54) modulation protein : **Backbone**	2	Cytoplasm		
RraA: Regulator of ribonuclease activity A : **Backbone**	2	Cytoplasm		
RuvA: Holliday junction DNA helicase ruvA : **Backbone**	2	Cytoplasm		
Ssb: Single-strand binding protein; helix-destabilizing protein : **Backbone**	2	Cytoplasm		
StpA: DNA-binding protein; H-NS homolog : **Backbone**	2	Cytoplasm		
LysU: Lysyl-tRNA synthetase, heat inducible : **Backbone**	2	Cytoplasm		
MetG: Methionyl-tRNA synthetase(ligase) : **Backbone**	2	Cytoplasm		
ArgS: Arginyl-tRNA synthetase : **Backbone**	2	Cytoplasm		
Tdh: L-threonine 3-dehydrogenase : **Backbone**	2	Cytoplasm		
AroC: Chorismate synthase : **Backbone**	1	Cytoplasm		
AroK: Shikimate kinase I : **Backbone**	1	Cytoplasm		
SpeG: Spermidine N(1)-acetyltransferase : **Backbone**	1	Cytoplasm		
CbpA: Curved DNA-binding protein (chaperone under environmental stresses) : **Backbone**	1	Cytoplasm		
CcdA: CcdA protein; LetA protein; Protein H : **pO157**	1	Cytoplasm		
CspD: Cold shock-like protein : **Backbone**	1	Cytoplasm		
DdlA: D-alanine--D-alanine ligase A : **Backbone**	1	Cytoplasm		
DdlB: D-alanine--D-alanine ligase B : B**ackbone**	1	Cytoplasm		
DeoB: Phosphopentomutase; phosphodeoxyribomutase: **Backbone**	1	Cytoplasm		
DeoC: Deoxyribose-phosphate aldolase; phosphodeoxyriboaldolase : B**ackbone**	1	Cytoplasm		
YeiP: Elongation factor P-like protein : **Backbone**	1	Cytoplasm		
EngD: GTP-dependent nucleic acid-binding protein : **Backbone**	1	Cytoplasm		
ErpA: Iron-Sulfur Insertion protein: **Backbone**	1	Cytoplasm		
EvgA: Positive transcription regulator evgA : **Backbone**	1	Cytoplasm		
FabH: 3-oxoacyl-[acyl-carrier-protein] synthase III : B**ackbone**	1	Cytoplasm		
FeoA: Ferrous iron transport protein A: **Backbone**	1	Cytoplasm		
FiS: DNA-binding protein fis; factor-for-inversion stimulation protein : **Backbone**	1	Cytoplasm		
Fmt: Methionyl-tRNA formyltransferase : **Backbone**	1	Cytoplasm		
Fur: Ferric uptake regulation protein : **Backbone**	1	Cytoplasm		
GapC: Glyceraldehyde-3-phosphate dehydrogenase C : **Backbone**	1	Cytoplasm		
GalF: UTP--glucose-1-phosphate uridylyltransferase : **Backbone**	1	Cytoplasm		
GlgB: 1,4-alpha-glucan branching enzyme : **Backbone**	1	Cytoplasm		
GmhB: D,D-heptose 1,7-bisphosphate phosphatase : **Backbone**	1	Cytoplasm		
GshA: Glutamate--cysteine ligase : **Backbone**	1	Cytoplasm		
GshB: Glutathione synthetase : **Backbone**	1	Cytoplasm		
HdhA: 7-alpha-hydroxysteroid dehydrogenase : **Backbone**	1	Cytoplasm		
HflK: protease specific for phage lambda cII : **Backbone**	1	Cytoplasm	**+**	**HflX**
Hha: Haemolysin expression modulating protein : **Backbone**	1	Cytoplasm		
HisE: Histidine biosynthesis bifunctional protein : **Backbone**	1	Cytoplasm		
HisH: Imidazole glycerol phosphate synthase : **Backbone**	1	Cytoplasm		

HisF: Imidazole glycerol phosphate synthase subunit : **Backbone**	1	Cytoplasm	
HslO: 33 kDa chaperonin; heat shock protein: **Backbone**	1	Cytoplasm	
HslV: ATP-dependent protease; heat shock protein : **Backbone**	1	Cytoplasm	
IlvC: Ketol-acid reductoisomerase : **Backbone**	1	Cytoplasm	
IlvD: Dihydroxy-acid dehydratase : **Backbone**	1	Cytoplasm	
IspG: 4-hydroxy-3-methylbut-2-en-1-yl diphosphate synthase (EC 1.17.4.3)	1	Cytoplasm	
Z5044: 2-amino-3-ketobutyrate coenzyme A ligase : **Backbone**	1	Cytoplasm	
KsgA: Dimethyladenosine transferase : **Backbone**	1	Cytoplasm	
LeuC: 3-isopropylmalate dehydratase large subunit : **Backbone**	1	Cytoplasm	
LeuD: 3-isopropylmalate dehydratase small subunit : **Backbone**	1	Cytoplasm	
YhbG: Lipopolysaccharide export system; ABC transporter ATP-binding protein : **Backbone**	1	Cytoplasm	
MdaB: Modulator of drug activity B : **Backbone**	1	Cytoplasm	
MoaB: Molybdenum cofactor biosynthesis protein B : **Backbone**	1	Cytoplasm	
MsrA: Peptide methionine sulfoxide reductase : **Backbone**	1	Cytoplasm	
Mug: Mismatch specific uracil DNA glycosylase : **Backbone**	1	Cytoplasm	
NdpA: Nucleoid-associated protein ndpA : **Backbone**	1	Cytoplasm	
NuoG: NADH-quinone oxidoreductase chain 3; NADH dehydrogenase I, chain G : **Backbone**	1	Cytoplasm	
PanB: 3-methyl-2-oxobutanoate hydroxymethyltransferase : **Backbone**	1	Cytoplasm	
ParC: Topoisomerase IV subunit A : **Backbone**	1	Cytoplasm	
PepB: Peptidase B : **Backbone**	1	Cytoplasm	
ChbB: PTS system, N,N'-diacetylchitobiose-specific IIB component (EIIB-Chb) : **Backbone**	1	Cytoplasm	
PurD: Phosphoribosylamine--glycine ligase : **Backbone**	1	Cytoplasm	
PurR: HTH-type transcriptional repressor; purine nucleotide synthesis repressor : **Backbone**	1	Cytoplasm	
PyrD: Dihydroorotate dehydrogenase : **Backbone**	1	Cytoplasm	
FrmA: Alcohol dehydrogenase class III; formaldehyde dehydrogenase, glutathione-dependent : **Backbone**	1	Cytoplasm	
YaiL: Nucleoprotein/polynucleotide-associated enzyme : **Backbone**	1	Cytoplasm	
KdgK: Ketodeoxygluconokinase : **Backbone**	1	Cytoplasm	
PhnN: ATP-binding component of phosphonate transport : **Backbone**	1	Cytoplasm	
Cfa: Cyclopropane fatty acyl phospholipid synthase : **Backbone**	1	Cytoplasm	
TopA: DNA topoisomerase type I, omega protein : **Backbone**	1	Cytoplasm	
Crl: Transcriptional regulator of cryptic csgA gene for curli surface fibers : **Backbone**	1	Cytoplasm	
MoaD: Molybdopterin biosynthesis protein D chain : **Backbone**	1	Cytoplasm	+
GalE: UDP-galactose-4-epimerase : **Backbone**	1	Cytoplasm	
SeqA: Negative modulator of initiation of replication : **Backbone**	1	Cytoplasm	
RpoN: RNA polymerase, sigma(54 or 60) factor; nitrogen and fermentation regulation : **Backbone**	1	Cytoplasm	
GlmM: Phosphoglucosamine mutase : **Backbone**	1	Cytoplasm	
HrpA: Helicase, ATP-dependent : **Backbone**	1	Cytoplasm	
IlvH: Acetolactate synthase III, valine sensitive, small subunit : **Backbone**	1	Cytoplasm	
ThrC: Threonine synthase : **Backbone**	1	Cytoplasm	
Zwf: Glucose-6-phosphate dehydrogenase : **Backbone**	1	Cytoplasm	
FabB: 3-oxoacyl-[acyl-carrier-protein] synthase I : **Backbone**	1	Cytoplasm	
FolD: 5,10-methylene-tetrahydrofolate dehydrogenase; 5,10-methylene-tetrahydrofolate cyclohydorlase : **Backbone**	1	Cytoplasm	
NuoC: NADH dehydrogenase I chain C, D (NADH dehydrogenase I chain C/D) : **Backbone**	1	Cytoplasm	
MenB: Dihydroxynaphtoic acid synthetase : **Backbone**	1	Cytoplasm	
PfkB: 6-phosphofructokinase II; suppressor of pfkA (6-phosphofructokinase II) : **Backbone**	1	Cytoplasm	
CyoA: Cytochrome o ubiquinol oxidase subunit II : **Backbone**	1	Cytoplasm	
UbiC: Chorismate lyase : **Backbone**	1	Cytoplasm	
PutA: Proline dehydrogenase, P5C dehydrogenase : **Backbone**	1	Cytoplasm	
PrfC: Peptide chain release factor 3 (RF-3) : **Backbone**	1	Cytoplasm	
RhlB: ATP-dependent RNA helicase : **Backbone**	1	Cytoplasm	+
Rho: Transcription termination factor : **Backbone**	1	Cytoplasm	
RpmI: 50S ribosomal protein L35 : **Backbone**	1	Cytoplasm	
RlmB: 23S rRNA (guanosine-2'-O-)-methyltransferase : **Backbone**	1	Cytoplasm	
Rnc: Ribonuclease III : **Backbone**	1	Cytoplasm	
Rpe: Ribulose-phosphate 3-epimerase : **Backbone**	1	Cytoplasm	
RsuA: Ribosomal small subunit pseudouridine synthase A : **Backbone**	1	Cytoplasm	
SerB: Phosphoserine phosphatase : **Backbone**	1	Cytoplasm	
SlyA: Transcriptional regulator slyA : **Backbone**	1	Cytoplasm	
SlyD: FKBP-type peptidyl-prolyl cis-trans isomerase slyD : **Backbone**	1	Cytoplasm	
SpeA: Biosynthetic arginine decarboxylase : **Backbone**	1	Cytoplasm	+
SpeB: Agmatine ureohydrolase : **Backbone**	1	Cytoplasm	
SpeE: Spermidine synthase; putrescine aminopropyltransferase : **Backbone**	1	Cytoplasm	
SurA: Survival protein surA precursor (Peptidyl-prolyl cis-trans isomerase surA) : **Backbone**	1	Cytoplasm	
HisS: Histidyl-tRNA synthetase : **Backbone**	1	Cytoplasm	
TrpA: Tryptophan synthase alpha chain : **Backbone**	1	Cytoplasm	
ThyA: Thymidylate synthase : **Backbone**	1	Cytoplasm	
UbiE: Ubiquinone/menaquinone biosynthesis methyltransferase : **Backbone**	1	Cytoplasm	
UspD: Universal stress protein D : **Backbone**	1	Cytoplasm	
UvrRB: UvrABC system protein B : **Backbone**	1	Cytoplasm	
UvrY: Response regulator; transcritional regulator : **Backbone**	1	Cytoplasm	
WbdQ: GDP-mannose mannosyl hydrolase wbdQ/wbhG : **Backbone**	1	Cytoplasm	
ZapA: Cell division protein; ftz associated : **Backbone**	1	Cytoplasm	

[1]PELS: Proteomics- based Expression Library Screening

[2]IVIAT:In Vivo-Induced Antigen Technology
