# Supplementary material for: Proteins other than the locus of enterocyte effacement-encoded proteins contribute to Escherichia coli O157:H7 adherence to bovine rectoanal junction stratified squamous epithelial cells
Source: BMC Microbiol. 2012 Jun 12;12:103. doi: 10.1186/1471-2180-12-103 (PMC3420319; doi:10.1186/1471-2180-12-103)
Supplement: Additional file 5 — http://www.biomedcentral.com/imedia/1777785157675419/supp5.pdf. DATA SHEETS: O157-DMEM MS/MS data sheet 1. [file 1471-2180-12-103-S5.pdf]

DMEM-01 SequestReport

	Reference					Score	Accession		Peptides (Hits)		Area
	Time(s)	Sequence	MH+	Charge	XC	Delta Cn	Sp	RSp	Ions	Count	Peak Area
#1	ADHE_ECOLI (P17547					870.34			87 (87 0 0 0 0)		17.08
	8.67	-.AAALAAADAR.-	901.00	2	3.27	0.54	1329.5	1	15/18		1.60E9
	103.36 - 104.57	-.AADIVLQAAIAAGAPK.-	1480.74	2	5.84	0.62	2215.2	1	23/30		4.42E9
	103.50 - 104.09	-.AADIVLQAAIAAGAPK.-	1480.74	1	2.36	0.18	508.9	4	12/30		1.51E9
	92.72	-.AAYSSGKPAIGVGAGNTPVVIDETADIK.-	2703.00	3	3.64	0.49	861.0	1	32/108		1.28E9
	86.23 - 87.36	-.AAYSSGKPAIGVGAGNTPVVIDETADIKR.-	2859.19	3	5.50	0.62	1320.1	1	38/112		6.21E9
	86.52 - 87.10	-.AAYSSGKPAIGVGAGNTPVVIDETADIKR.-	2859.19	2	5.49	0.66	1157.4	1	23/56		1.68E9
	88.41	-.AAYSSGKPAIGVGAGNTPVVIDETADIKR.-	2859.19	3	4.91	0.58	1912.8	1	42/112		1.65E9
	9.40	-.AKDFEDAVEK.-	1152.24	1	2.70	0.38	650.7	1	12/18		8.10E8
	57.46	-.AKDFEDAVEKAEK.-	1480.60	3	3.90	0.51	1414.0	1	28/48		3.79E8
	57.26 - 57.84	-.AKDFEDAVEKAEK.-	1480.60	2	4.27	0.51	2007.7	1	20/24		7.28E8
	18.73 - 20.17	-.AVASVLM*SK.-	922.13	2	3.13	0.47	876.2	1	15/16		2.11E8
	20.83 - 22.37	-.AVASVLM*SK.-	922.13	2	2.82	0.39	728.8	1	14/16		4.16E8
	25.58 - 26.51	-.AVASVLM*SK.-	922.13	2	3.08	0.42	831.6	1	14/16		4.40E8
	22.96 - 24.60	-.AVASVLM*SK.-	922.13	2	3.12	0.43	877.4	1	15/16		5.89E8
	63.07 - 63.66	-.AVQDVILK.-	886.07	2	2.78	0.32	595.5	1	13/14		1.09E9
	98.99	-.AVTNVAELNALVER.-	1499.70	1	2.34	0.39	109.2	54	9/26		1.01E9
	99.18	-.AVTNVAELNALVER.-	1499.70	1	2.99	0.41	386.9	1	14/26		6.79E8
	47.21 - 48.82	-.DFEDAVEK.-	952.99	1	1.87	0.19	646.4	1	11/14		2.66E8
	94.20 - 94.83	-.EAGVQEADFLANVDK.-	1606.72	2	4.24	0.63	1105.3	1	18/28		3.62E9
	130.24 - 131.39	-.EAGVQEADFLANVDKLSEDAFDDQCTGANPR.-	3384.52	3	5.70	0.50	1225.1	1	36/120		4.30E9
	128.26 - 129.40	-.EAGVQEADFLANVDKLSEDAFDDQCTGANPR.-	3384.52	3	4.93	0.59	1305.2	1	32/120		1.19E10
	125.03	-.EAGVQEADFLANVDKLSEDAFDDQCTGANPR.-	3384.52	3	3.53	0.54	651.7	1	26/120		1.17E9
	126.78	-.EAGVQEADFLANVDKLSEDAFDDQCTGANPR.-	3384.52	3	6.04	0.63	1696.4	1	38/120		1.29E9
	118.42 - 119.19	-.EAGVQEADFLANVDKLSEDAFDDQCTGANPR.-	3384.52	3	5.49	0.57	980.9	1	29/120		1.19E9
	120.30	-.EAGVQEADFLANVDKLSEDAFDDQCTGANPR.-	3384.52	3	5.86	0.58	1430.1	1	34/120		1.05E9
	63.43 - 64.51	-.EYASFTQEQVDK.-	1445.51	2	3.65	0.61	971.5	1	15/22		5.68E9
	63.77	-.EYASFTQEQVDK.-	1445.51	1	2.87	0.44	283.5	1	13/22		1.14E9
	63.56 - 64.15	-.EYASFTQEQVDK.-	1445.51	1	1.81	0.13	302.1	1	14/22		1.84E9
	35.34 - 36.93	-.EYLPASYHEGSK.-	1381.47	2	2.80	0.41	536.2	1	16/22		4.75E8
	58.96	-.EYLPASYHEGSKNPVAR.-	1919.09	2	4.00	0.70	657.1	1	18/32		4.77E8
	66.29	-.FATHGGYLLQGK.-	1292.47	1	2.74	0.32	558.6	1	13/22		6.96E8
	66.61 - 67.32	-.FATHGGYLLQGK.-	1292.47	2	3.43	0.38	1013.2	1	18/22		2.83E9
	66.14	-.FATHGGYLLQGK.-	1292.47	3	3.02	0.30	1458.5	1	25/44		8.53E8
	127.11 - 127.94	-.FLFNNGYADQITSVLK.-	1831.06	2	4.85	0.50	1985.3	1	25/30		1.41E10
	125.35 - 126.80	-.FLFNNGYADQITSVLK.-	1831.06	2	5.08	0.55	1758.9	1	24/30		1.18E10
	132.56 - 133.88	-.FLFNNGYADQITSVLK.-	1831.06	2	3.18	0.50	1048.4	1	18/30		3.60E9
	111.51 - 112.49	-.GAELANSFKPDVIIALGGGSPM*DAAK.-	2546.88	3	3.46	0.45	580.4	1	32/100		4.41E9
	106.82	-.GSLPIALDEVITDGHK.-	1665.87	2	3.58	0.61	690.5	1	16/30		1.24E9
	99.55 - 100.72	-.GSLPIALDEVITDGHKR.-	1822.06	2	3.77	0.38	856.6	1	15/32		2.26E9
	96.40	-.GSLPIALDEVITDGHKR.-	1822.06	2	4.62	0.47	966.5	1	16/32		9.20E8
	91.10 - 92.45	-.IAELAGFSVPENTK.-	1476.66	2	4.53	0.50	855.3	1	17/26		1.11E10
	104.68 - 105.31	-.ILIGEVTVVDESEPFAHEK.-	2113.35	3	3.98	0.44	888.7	1	34/72		4.40E9
	104.70	-.ILIGEVTVVDESEPFAHEK.-	2113.35	2	5.18	0.65	1160.9	1	21/36		2.52E9
	109.67 - 111.01	-.ILINTPASQGGIGDLYNFK.-	2022.29	2	5.19	0.65	461.8	1	17/36		2.05E10
	113.40 - 114.69	-.ILINTPASQGGIGDLYNFK.-	2022.29	2	6.08	0.64	1405.0	1	25/36		1.13E10
	111.80 - 112.39	-.ILINTPASQGGIGDLYNFK.-	2022.29	3	6.08	0.63	1729.3	1	39/72		3.31E9
	111.55 - 112.80	-.ILINTPASQGGIGDLYNFK.-	2022.29	2	5.73	0.67	1418.1	1	26/36		3.04E10

	105.62 - 106.23	-.IM*WVM*YEHPETHFEELALR.-	2464.80	3	3.93	0.57	576.4	1	25/72	1.71E9
	105.15	-.KGAELANSFKPDVIIALGGGSPM*DAAK.-	2675.06	2	3.40	0.61	643.4	1	18/52	7.45E8
	104.84 - 105.39	-.KGAELANSFKPDVIIALGGGSPM*DAAK.-	2675.06	3	4.90	0.47	1352.6	1	37/104	4.09E9
	103.19	-.KGAELANSFKPDVIIALGGGSPM*DAAK.-	2675.06	3	3.59	0.42	873.4	1	32/104	1.98E9
	123.66	-.LGSQFHIPHGLANALLICNVIR.-	2444.86	3	4.61	0.46	2795.0	1	40/84	8.32E8
	119.12	-.LLAWLETLK.-	1087.34	2	3.07	0.37	559.0	1	13/16	8.88E8
	66.33	-.LSEDAFDDQCTGANPR.-	1796.82	2	2.87	0.40	936.7	1	16/30	6.60E8
	64.61 - 65.20	-.LSEDAFDDQCTGANPR.-	1796.82	2	4.02	0.50	1291.0	1	19/30	1.31E9
	70.27 - 70.83	-.LVAM*GGIGHTSCLYTDQDNQPAR.-	2521.74	2	3.97	0.63	635.0	1	19/44	1.61E9
	69.67 - 70.81	-.LVAM*GGIGHTSCLYTDQDNQPAR.-	2521.74	3	6.82	0.63	2008.4	1	39/88	4.20E9
	82.03	-.LVAMGGIGHTSCLYTDQDNQPAR.-	2505.74	2	2.95	0.53	690.2	1	17/44	5.69E8
	17.09 - 25.71	-.M*AVAESGM*GIVEDK.-	1469.66	2	3.70	0.50	842.4	1	22/26	1.19E9
	12.65 - 13.87	-.M*AVAESGM*GIVEDK.-	1469.66	2	4.36	0.51	1491.5	1	21/26	2.94E8
	28.10 - 29.87	-.M*AVAESGM*GIVEDK.-	1469.66	2	4.28	0.52	1799.0	1	23/26	9.95E8
	26.22 - 27.94	-.M*AVAESGM*GIVEDK.-	1469.66	2	4.20	0.53	1864.4	1	22/26	1.12E9
	14.72 - 16.50	-.M*AVAESGM*GIVEDK.-	1469.66	2	4.66	0.60	1956.5	1	23/26	1.86E8
	30.55 - 31.21	-.M*AVAESGM*GIVEDK.-	1469.66	2	3.50	0.46	1438.3	1	19/26	8.45E8
	10.81 - 11.99	-.M*AVAESGM*GIVEDK.-	1469.66	2	3.65	0.48	2088.4	1	21/26	5.59E8
	69.71 - 70.65	-.M*AVAESGMGIVEDK.-	1453.67	2	3.65	0.59	1365.9	1	20/26	1.12E9
	62.44 - 63.70	-.NAIIFSPHPR.-	1152.33	1	1.88	0.22	214.5	1	12/18	8.77E8
	81.62 - 82.77	-.NGALNAAIVGQPAYK.-	1487.69	2	3.66	0.61	1857.9	1	21/28	3.19E9
	81.85 - 81.87	-.NGALNAAIVGQPAYK.-	1487.69	1	2.76	0.48	247.2	1	12/28	9.05E8
	79.41 - 79.45	-.NGALNAAIVGQPAYK.-	1487.69	1	3.45	0.57	532.1	1	16/28	7.69E8
	79.83 - 80.30	-.NGALNAAIVGQPAYK.-	1487.69	2	3.58	0.59	1910.8	1	20/28	1.75E9
	76.40	-.NGALNAAIVGQPAYK.-	1487.69	2	3.10	0.50	891.5	1	16/28	1.14E9
	75.47 - 76.44	-.NGALNAAIVGQPAYK.-	1487.69	2	3.60	0.63	1441.1	1	18/28	1.07E9
	80.00	-.NHFASEYIYNAYK.-	1620.75	2	3.20	0.51	650.2	1	14/24	1.04E9
	72.10	-.NHFASEYIYNAYKDEK.-	1993.12	2	5.26	0.56	1992.0	1	21/30	4.30E8
	88.53 - 89.78	-.PAIGVGAGNTPVVIDETADIKR.-	2194.47	3	3.95	0.55	1147.4	1	34/84	2.69E9
	87.82 - 88.45	-.QILLDTYYGR.-	1242.41	2	2.89	0.45	527.7	2	12/18	2.52E9
	96.56 - 97.49	-.RGSLPIALDEVITDGHK.-	1822.06	3	3.34	0.42	1033.7	1	30/64	1.22E9
	138.81 - 140.06	-.TFDNGVICASEQSVVVVDSVYDAVR.-	2730.96	3	4.15	0.56	1359.2	1	30/96	1.17E9
	139.13 - 139.99	-.TFDNGVICASEQSVVVVDSVYDAVR.-	2730.96	2	4.97	0.67	953.9	1	19/48	8.99E8
	142.31 - 142.85	-.TFDNGVICASEQSVVVVDSVYDAVR.-	2730.96	2	2.67	0.12	376.2	1	15/48	9.17E8
	146.38	-.TFDNGVICASEQSVVVVDSVYDAVR.-	2730.96	2	2.61	0.53	584.3	1	16/48	2.76E8
	83.98	-.YAEIADHLGLSAPGDR.-	1685.82	1	2.11	0.39	420.6	1	15/30	6.99E8
	83.70 - 84.30	-.YAEIADHLGLSAPGDR.-	1685.82	2	5.17	0.61	2180.7	1	22/30	5.02E9
	121.45 - 122.05	-.YPLADYALTPDM*AIVDANLVM*DM*PK.-	2817.25	2	3.54	0.57	392.8	1	17/48	1.47E9
	122.40 - 123.08	-.YPLADYALTPDM*AIVDANLVM*DM*PK.-	2817.25	3	5.40	0.59	1056.6	1	30/96	5.59E9
	87.65 - 88.26	-.YPLISELK.-	963.15	2	2.91	0.26	1018.3	1	13/14	1.54E9
#2	EFG_ECOLI (P02996)					600.29			60 (60 0 0 0 0)	14.87
	87.72	-.AGDIAAAIGLK.-	1000.17	1	2.77	0.42	905.0	1	13/20	2.04E9
	87.63 - 88.17	-.AGDIAAAIGLK.-	1000.17	2	3.23	0.40	975.3	1	16/20	1.68E9
	87.57 - 88.15	-.AGDIAAAIGLK.-	1000.17	1	2.24	0.17	953.0	1	13/20	3.77E9
	90.87 - 91.64	-.AGPLAGYPVVDM*GIR.-	1532.79	2	3.74	0.67	582.0	1	17/28	4.89E9
	106.10	-.AGPLAGYPVVDMGIR.-	1516.79	2	3.15	0.56	858.0	1	19/28	2.27E9
	68.99 - 69.40	-.AKPVLLEPIM*K.-	1255.60	2	2.71	0.51	687.4	1	15/20	1.66E9
	69.37	-.AKPVLLEPIM*K.-	1255.60	3	3.26	0.41	1220.4	1	25/40	4.86E8
	75.53 - 76.09	-.ASYTM*EFLK.-	1106.27	2	2.56	0.50	612.8	1	13/16	1.51E9
	118.31	-.ASYTM*EFLKYDEAPSNVAQAVIEAR.-	2821.11	2	3.82	0.68	373.2	1	15/48	6.46E8
	100.80 - 101.94	-.DVTTGDTLCDPDAPIILER.-	2102.28	2	4.37	0.61	1604.6	1	23/36	1.30E10

	77.26 - 77.84	-.EFNVEANVGKPQVAYR.-	1822.01	2	4.08	0.59	1193.4	1	21/30	7.67E9
	79.56 - 80.85	-.EFNVEANVGKPQVAYR.-	1822.01	2	3.27	0.53	408.8	1	12/30	1.17E9
	90.83 - 91.99	-.GGVIPGEYIPAVDK.-	1415.62	2	3.55	0.55	638.8	1	20/26	6.46E9
	91.22 - 91.79	-.GGVIPGEYIPAVDK.-	1415.62	1	2.50	0.28	507.5	1	14/26	1.31E9
	109.65 - 110.20	-.GITITSAATTAFWSGM*AK.-	1831.08	2	3.48	0.59	666.3	1	15/34	2.82E9
	84.89 - 85.45	-.GQYGHVVIDM*YPLEPGSNPK.-	2218.48	3	3.84	0.52	1156.7	1	31/76	5.77E9
	85.00 - 85.57	-.GQYGHVVIDM*YPLEPGSNPK.-	2218.48	2	5.19	0.63	816.5	1	18/38	3.63E9
	98.45	-.GQYGHVVIDMYPLEPGSNPK.-	2202.48	2	4.65	0.62	631.3	1	15/38	6.41E8
	87.04 - 88.88	-.GYEFINDIK.-	1099.22	1	2.56	0.40	519.4	1	11/16	3.10E9
	88.34	-.GYEFINDIK.-	1099.22	2	2.89	0.52	917.9	1	13/16	1.87E9
	95.18	-.HASDDEPFSALAFK.-	1535.64	1	2.90	0.45	696.8	1	14/26	9.17E8
	94.63 - 95.26	-.HASDDEPFSALAFK.-	1535.64	3	4.34	0.62	1531.1	1	33/52	1.44E9
	94.55 - 95.74	-.HASDDEPFSALAFK.-	1535.64	2	4.48	0.48	1191.9	1	20/26	4.90E9
	125.72 - 127.23	-.IATDPFVGNLTFFR.-	1598.83	2	4.40	0.48	1007.1	1	19/26	1.72E10
	129.01	-.IATDPFVGNLTFFR.-	1598.83	2	4.02	0.50	1164.1	1	20/26	1.08E9
	70.03	-.IGEVHDGAATM*DWM*EQEQER.-	2365.50	3	4.35	0.51	1377.1	1	31/76	1.09E9
	99.71 - 100.30	-.IHAEVPLSEM*FGYATQLR.-	2079.37	3	4.73	0.57	1643.9	1	29/68	4.12E9
	98.04 - 99.13	-.IHAEVPLSEM*FGYATQLR.-	2079.37	3	4.85	0.62	1563.9	1	29/68	5.70E9
	99.25 - 99.83	-.IHAEVPLSEM*FGYATQLR.-	2079.37	2	4.73	0.65	614.7	1	22/34	2.35E9
	124.07	-.IHAEVPLSEMFGYATQLR.-	2063.37	2	4.41	0.55	665.0	1	21/34	7.30E8
	123.80	-.IHAEVPLSEMFGYATQLR.-	2063.37	3	4.94	0.47	1962.0	1	33/68	1.30E9
	72.70 - 73.24	-.ILFYTGVNHK.-	1192.39	2	3.24	0.32	935.0	1	15/18	1.31E9
	108.58 - 109.44	-.INIIDTPGHVDFTIEVER.-	2069.30	3	4.44	0.50	598.1	1	28/68	1.19E9
	108.68	-.INIIDTPGHVDFTIEVER.-	2069.30	2	4.60	0.66	590.4	1	19/34	1.05E9
	69.77	-.LAASIAFK.-	821.00	2	2.59	0.45	459.9	1	12/14	8.02E8
	152.61 - 154.32	-.LGANPVPLQLAIGAEEHFTGVVDLVK.-	2689.10	3	5.85	0.63	570.1	1	31/100	8.04E8
	177.11	-.LGANPVPLQLAIGAEEHFTGVVDLVK.-	2689.10	3	3.13	0.50	516.3	1	27/100	9.42E7
	89.60 - 90.14	-.LHFGSYHDVDSSELAFK.-	1953.10	3	4.02	0.58	963.4	1	30/64	2.21E9
	89.68 - 90.24	-.LHFGSYHDVDSSELAFK.-	1953.10	2	5.40	0.69	1777.3	1	21/32	3.12E9
	107.68 - 107.89	-.M*EFPEPVISIAVEPK.-	1702.99	1	2.50	0.27	124.1	1	13/28	1.18E9
	107.31 - 107.85	-.M*EFPEPVISIAVEPK.-	1702.99	2	3.98	0.67	599.2	1	19/28	1.25E10
	60.07 - 61.22	-.NIGISAHIDAGK.-	1196.34	2	3.64	0.46	1124.1	1	18/22	1.74E9
	62.92	-.NIGISAHIDAGK.-	1196.34	2	2.94	0.47	1216.3	1	18/22	9.69E8
	94.89 - 96.19	-.VEVETPEENTGDVIGDLSR.-	2060.16	2	4.97	0.61	737.0	1	21/36	1.11E10
	109.89 - 110.44	-.VLDGAVM*VYCAVGGVQPQSETVWR.-	2638.97	2	2.53	0.42	296.5	1	13/46	2.28E9
	112.89 - 113.42	-.VLNNEIILVTCGSAFK.-	1779.06	2	2.64	0.51	604.2	1	13/30	1.40E9
	110.17	-.VLNNEIILVTCGSAFK.-	1779.06	1	2.62	0.52	292.7	2	12/30	7.38E8
	109.73 - 111.10	-.VLNNEIILVTCGSAFK.-	1779.06	2	5.46	0.61	1666.4	1	22/30	6.46E9
	88.61 - 90.03	-.VYSGVVNSGDTVLNSVK.-	1738.92	2	4.77	0.57	1003.9	1	21/32	1.23E10
	88.84 - 88.86	-.VYSGVVNSGDTVLNSVK.-	1738.92	1	2.67	0.44	204.7	1	14/32	1.28E9
	80.34 - 81.05	-.VYSGVVNSGDTVLNSVK.-	1738.92	2	4.16	0.61	774.5	1	17/32	1.67E9
	79.60 - 80.14	-.VYSGVVNSGDTVLNSVK.-	1738.92	2	4.58	0.53	1850.7	1	24/32	2.41E9
	82.26 - 83.55	-.VYSGVVNSGDTVLNSVK.-	1738.92	2	4.06	0.58	896.7	1	20/32	1.63E9
	86.25 - 87.54	-.VYSGVVNSGDTVLNSVK.-	1738.92	2	4.34	0.58	907.2	1	21/32	3.55E9
	94.66	-.YDEAPSNVAQAVIEAR.-	1733.86	1	2.19	0.31	188.8	1	13/30	1.16E9
	94.78	-.YDEAPSNVAQAVIEAR.-	1733.86	3	4.84	0.46	2708.7	1	34/60	7.77E8
	95.41	-.YDEAPSNVAQAVIEAR.-	1733.86	2	3.86	0.57	771.8	1	19/30	1.10E10
	93.79 - 95.05	-.YDEAPSNVAQAVIEAR.-	1733.86	2	5.82	0.57	1889.5	1	26/30	1.17E10
	84.34 - 84.91	-.YLGGEELTEAEIK.-	1452.59	1	2.57	0.17	789.9	1	14/24	2.32E9
	84.28 - 85.71	-.YLGGEELTEAEIK.-	1452.59	2	4.36	0.49	1031.4	1	20/24	7.72E9
#3	Q8XEB4 (Q8XEB4) Fo					550.30			55 (55 0 0 0 0)	10.30

9.26	-.AGAPFGPGANPM*HGR.-	1453.61	2	2.93	0.40	590.6	2	14/28		1.14E9
120.16 - 120.99	-.DAIPTQSVLTITSNVVYGK.-	2007.27	2	5.21	0.74	1294.5	1	23/36		9.63E9
114.17	-.DAIPTQSVLTITSNVVYGKK.-	2135.45	2	3.75	0.24	593.3	2	17/38		1.04E9
136.15	-.DFIQKNYTPYEGDESFLAGATEATTTLWDK.-	3413.65	3	3.81	0.47	724.2	1	27/116		7.37E8
118.73 - 119.31	-.DGISYTFSIVPNALGK.-	1682.90	2	3.81	0.65	696.9	1	16/30		2.91E9
115.05	-.DGISYTFSIVPNALGKDDEVR.-	2297.51	3	3.72	0.53	1354.8	1	33/80		2.73E9
114.80	-.DGISYTFSIVPNALGKDDEVR.-	2297.51	2	3.81	0.60	441.3	1	18/40		1.37E9
126.09 - 127.29	-.DKYAQFTSLQADLENGVNLEQTIR.-	2754.99	3	3.12	0.47	1865.5	1	34/92		2.58E9
122.42	-.DKYAQFTSLQADLENGVNLEQTIR.-	2754.99	3	4.55	0.46	1152.3	1	29/92		1.16E9
93.75	-.EM*LLDAM*ENPEKYPQLTIR.-	2324.66	2	2.77	0.43	101.3	9	11/36		3.71E9
93.15 - 93.73	-.EM*LLDAM*ENPEKYPQLTIR.-	2324.66	3	4.10	0.38	844.1	1	30/72		7.10E9
91.95 - 93.17	-.EM*LLDAM*ENPEKYPQLTIR.-	2324.66	2	2.63	0.39	246.4	2	15/36		4.37E9
109.00 - 109.11	-.EM*LLDAMENPEKYPQLTIR.-	2308.66	2	2.80	0.07	176.8	3	13/36		1.35E9
113.56 - 114.12	-.FLNTLYTM*GPSPEPNM*TILWSEK.-	2703.08	2	3.18	0.47	289.0	1	13/44		2.49E9
130.29 - 130.82	-.FLNTLYTM*GPSPEPNM*TILWSEKLPLNFK.-	3415.97	3	4.47	0.43	725.8	1	30/112		9.86E8
62.31 - 62.84	-.GAVASLTSVAK.-	1004.16	2	3.60	0.49	1042.9	1	16/20	1	1.82E9
106.04 - 106.71	-.IFTEYRKTHNQGVFDVYTPDILR.-	2814.15	3	3.74	0.53	489.1	1	22/88		3.54E9
91.75 - 92.97	-.ITEQEAQEM*VDHLVM*K.-	1934.18	2	4.36	0.52	268.0	2	15/30		3.99E9
90.96 - 92.34	-.ITEQEAQEM*VDHLVM*K.-	1934.18	3	3.14	0.36	311.7	6	19/60		5.59E9
110.95	-.ITEQEAQEMVDHLVM*K.-	1918.18	3	3.05	0.19	317.1	4	19/60		1.30E9
99.49	-.KTHNQGVFDVYTPDILR.-	2004.24	3	4.49	0.48	1627.7	1	32/64		1.27E9
87.14	-.LATAWEGFTK.-	1124.27	1	1.92	0.35	511.0	2	11/18		2.02E9
87.30	-.LATAWEGFTK.-	1124.27	1	2.46	0.37	540.2	1	12/18		1.08E9
81.03 - 81.78	-.M*DHFM*DWLAK.-	1326.53	2	2.77	0.49	569.7	1	12/18		1.82E9
133.95 - 135.21	-.NYTPYEGDESFLAGATEATTTLWDK.-	2781.92	2	5.44	0.63	736.4	1	19/48		4.25E9
140.50	-.NYTPYEGDESFLAGATEATTTLWDK.-	2781.92	2	3.66	0.55	470.9	1	17/48		6.07E8
155.19 - 156.76	-.NYTPYEGDESFLAGATEATTTLWDKVM*EGVK.-	3441.72	3	4.15	0.52	506.3	1	35/120		1.60E9
83.76 - 85.04	-.SEPIKGDLLNYDEVM*ER.-	2025.23	3	3.80	0.65	1134.7	1	30/64		4.16E9
84.98 - 85.59	-.SEPIKGDLLNYDEVM*ER.-	2025.23	2	4.08	0.58	971.1	1	18/32		4.99E9
85.61	-.SEPIKGDLLNYDEVM*ER.-	2025.23	3	3.35	0.50	716.0	1	28/64		3.95E9
96.46	-.SEPIKGDLLNYDEVMER.-	2009.23	2	3.84	0.57	861.8	1	19/32		9.07E8
86.83 - 88.11	-.SGVLTGLPDAYGR.-	1306.45	2	3.61	0.48	779.2	1	17/24		5.49E9
98.71 - 100.10	-.THAPVDFDTAVASTITSHDAGYINK.-	2632.82	3	4.44	0.62	505.4	1	30/96		2.89E9
101.29	-.THAPVDFDTAVASTITSHDAGYINK.-	2632.82	2	5.12	0.68	1305.3	1	23/48		1.23E9
110.22	-.THNQGVFDVYTPDILR.-	1876.06	3	3.36	0.38	1113.8	2	26/60		9.88E8
106.04 - 106.71	-.THNQGVFDVYTPDILR.-	1876.06	2	5.93	0.67	1691.0	1	21/30		3.54E9
108.48	-.THNQGVFDVYTPDILR.-	1876.06	2	4.13	0.61	1148.4	1	18/30		7.75E8
114.48 - 115.62	-.TM*ACGIAGLSVAADSLSAIK.-	1953.24	2	4.82	0.70	1026.3	1	21/38		6.12E9
71.73 - 73.12	-.TM*LYAINGGVDEK.-	1427.61	2	3.65	0.19	1766.4	1	20/24		5.54E9
73.73 - 74.33	-.TM*LYAINGGVDEK.-	1427.61	2	3.54	0.51	1403.4	1	19/24		3.12E9
71.93	-.TM*LYAINGGVDEK.-	1427.61	1	1.93	0.45	185.5	1	12/24		1.02E9
73.69 - 74.28	-.TM*LYAINGGVDEK.-	1427.61	1	2.40	0.43	151.9	1	13/24		7.70E8
79.50	-.TM*LYAINGGVDEKLK.-	1668.94	2	3.77	0.59	1257.0	1	21/28		1.19E9
120.65 - 121.37	-.TMACGIAGLSVAADSLSAIK.-	1937.24	2	3.44	0.55	708.8	1	17/38		1.69E9
132.45 - 132.99	-.TPEYDELFSGDPIWATESIGGM*GLDGR.-	2931.14	2	3.56	0.62	574.2	1	18/52		2.31E9
132.41 - 133.11	-.TPEYDELFSGDPIWATESIGGM*GLDGR.-	2931.14	3	5.37	0.56	1055.1	1	33/104		4.81E9
104.74 - 105.29	-.TSTFLDVYIER.-	1344.50	2	3.08	0.48	941.5	1	15/20		2.65E9
105.00	-.TSTFLDVYIER.-	1344.50	1	1.82	0.35	203.4	1	10/20		7.95E8
100.06 - 100.66	-.VALYGIDYLM*K.-	1302.56	2	3.63	0.50	1228.2	1	17/20		4.24E9
92.93	-.VALYGIDYLM*KDK.-	1545.83	2	3.02	0.32	387.6	4	12/24		2.70E9
114.02	-.VALYGIDYLMK.-	1286.57	2	2.91	0.44	1088.3	1	16/20		6.66E8

	92.68 - 93.26	-.VDDLAVDLVER.-	1244.38	2	4.51	0.44	2126.3	1	19/20		6.07E9
	71.08 - 72.35	-.VVGLQTEAPLK.-	1155.37	2	2.84	0.49	897.3	1	16/20		9.43E8
	61.93 - 62.04	-.VVGLQTEAPLKR.-	1311.56	1	2.10	0.12	289.7	1	13/22		9.84E8
	80.44 - 81.32	-.YSYEASLM*ALHDR.-	1572.72	2	2.83	0.53	1195.4	1	16/24	1	1.20E9
#4	ODP1_ECOLI (P06958					490.35			49 (49 0 0 0 0)		6.20
	54.41 - 55.02	-.ALNVM*LK.-	805.02	2	2.54	0.33	410.6	3	11/12		3.03E8
	127.27	-.AQYLIDQLLAEAR.-	1504.71	1	2.58	0.39	386.4	1	12/24		5.68E8
	127.00 - 127.58	-.AQYLIDQLLAEAR.-	1504.71	2	5.05	0.46	2008.7	1	20/24		4.03E9
	92.61	-.ARNEQDGGDLVYFQGHISPGVYAR.-	2650.85	3	5.13	0.56	1996.3	1	37/92		1.54E9
	58.44	-.ATVILAHTIK.-	1067.31	1	1.81	0.29	336.7	2	11/18		5.34E8
	58.31 - 58.89	-.ATVILAHTIK.-	1067.31	2	2.78	0.39	948.1	1	15/18		1.16E9
	78.47	-.DRFNVPVSDADIEK.-	1605.73	2	4.01	0.45	1170.9	1	19/26		7.35E8
	152.90 - 153.45	-.DWLQAIESVIR.-	1330.51	2	4.70	0.47	2059.3	1	17/20		6.81E8
	149.05 - 150.29	-.DWLQAIESVIREEGVER.-	2030.23	2	3.41	0.33	221.4	2	14/32		7.80E8
	150.15	-.DWLQAIESVIREEGVER.-	2030.23	3	4.19	0.57	1620.7	1	32/64		3.43E8
	118.97 - 120.18	-.DYGVGSDVYSVTSFTELAR.-	2067.20	2	5.54	0.72	1374.9	1	22/36		4.56E9
	119.49 - 119.70	-.DYGVGSDVYSVTSFTELAR.-	2067.20	3	3.07	0.36	1176.5	1	30/72		6.52E8
	86.89	-.EISTTIAFVR.-	1137.31	2	2.58	0.49	1410.7	1	17/18		1.49E9
	113.87 - 114.96	-.EKLDNLVFVINCNLQR.-	1976.26	2	3.93	0.52	654.5	1	16/30		1.56E9
	62.08 - 62.61	-.FNIDADKVNPR.-	1289.42	2	2.96	0.34	1381.9	1	17/20		1.48E9
	82.60	-.FNVPVSDADIEK.-	1334.46	2	2.62	0.39	442.0	1	15/22		8.11E8
	76.46 - 77.10	-.FPNDVDPIETR.-	1303.40	2	3.46	0.45	1145.6	1	17/20		7.16E8
	23.23 - 24.65	-.GAITIATR.-	802.94	2	2.99	0.34	1185.2	1	13/14		3.59E8
	69.56 - 70.15	-.GFLIGGTSGR.-	965.09	2	2.52	0.48	1212.8	1	16/18		1.15E9
	80.38 - 81.48	-.IGDLCWAAGDQQAR.-	1561.68	2	3.77	0.47	1366.4	1	18/26		3.17E9
	143.38 - 144.52	-.IINELEGIFEGAGWNVIK.-	2003.29	2	5.78	0.64	1914.1	1	25/34		2.13E9
	143.48 - 144.01	-.IINELEGIFEGAGWNVIK.-	2003.29	3	7.07	0.52	2449.3	1	36/68		8.00E8
	48.75 - 49.37	-.IYAAFK.-	712.86	1	1.90	0.27	680.8	1	8/10		4.71E8
	60.89 - 60.94	-.KGIYKLETIEGSK.-	1466.71	2	3.10	0.58	1375.6	1	17/24		5.65E8
	161.38	-.LDGPVTGNGKIINELEGIFEGAGWNVIK.-	2942.32	3	5.65	0.64	1324.8	1	37/108		1.53E8
	145.06 - 146.68	-.LELPSLQDFGALLEEQSK.-	2018.25	2	5.67	0.67	1164.6	1	22/34		5.34E9
	145.53 - 146.17	-.LELPSLQDFGALLEEQSK.-	2018.25	3	6.12	0.58	2836.4	1	39/68		5.10E8
	92.22 - 93.38	-.LIQLM*NETVDGDYQTFK.-	2032.26	2	4.57	0.35	832.8	1	17/32		7.43E9
	90.59 - 91.68	-.LIQLM*NETVDGDYQTFK.-	2032.26	2	3.20	0.00	678.1	1	18/32		3.73E9
	96.00	-.LIQLM*NETVDGDYQTFK.-	2032.26	2	4.06	0.45	972.9	1	18/32		1.55E9
	93.97 - 95.09	-.LIQLM*NETVDGDYQTFK.-	2032.26	2	5.37	0.53	1224.9	1	20/32		6.94E9
	142.05 - 142.73	-.LM*PEFWQFPTVSM*GLGPIGAIYQAK.-	2815.30	3	5.78	0.64	1502.6	1	36/96		4.16E9
	142.25 - 142.87	-.LM*PEFWQFPTVSM*GLGPIGAIYQAK.-	2815.30	2	3.25	0.49	348.6	1	18/48		1.37E9
	96.64	-.LPYITFPEGSEEHTYLHAQR.-	2389.61	3	3.38	0.51	857.9	1	28/76		9.50E8
	71.85	-.LTQEQLDNFR.-	1264.37	2	3.94	0.54	1375.6	2	15/18		1.10E9
	79.08 - 79.71	-.LVPIIADEAR.-	1097.29	2	2.51	0.55	438.8	1	16/18		3.12E9
	98.51	-.NEQDGGDLVYFQGHISPGVYAR.-	2423.58	2	3.72	0.51	720.8	1	18/42		9.66E8
	98.35 - 100.34	-.NEQDGGDLVYFQGHISPGVYAR.-	2423.58	3	3.86	0.38	761.3	1	28/84		2.24E9
	113.07	-.QENVYYYITTLNENYHM*PAM*PEGAEEGIR.-	3466.76	3	3.57	0.48	1060.1	1	31/112		1.22E9
	77.41 - 78.80	-.QIGIYSPNGQQYTPQDR.-	1966.10	2	4.00	0.59	1014.0	1	21/32		3.89E9
	141.56 - 142.97	-.QPNFTEKLELPSLQDFGALLEEQSK.-	2863.17	3	4.00	0.48	552.6	2	26/96		1.48E9
	75.29 - 76.70	-.SERFPNDVDPIETR.-	1675.78	2	3.18	0.35	979.4	1	18/26		1.72E9
	83.03 - 84.17	-.TFGM*EGLFR.-	1074.24	2	2.66	0.34	499.4	1	14/16		1.52E9
	86.79	-.VPYIAQVM*NDAPAVASTDYM*K.-	2317.63	2	5.29	0.62	2137.3	1	25/40		1.26E9
	90.56 - 91.89	-.VPYIAQVM*NDAPAVASTDYM*K.-	2317.63	2	6.26	0.55	1345.2	1	22/40		2.51E9
	92.51	-.VPYIAQVM*NDAPAVASTDYM*K.-	2317.63	2	4.58	0.59	1050.9	1	22/40		1.43E9

	92.53 - 92.59	-.VQLLGSGSILR.-	1143.36	1	1.93	0.29	131.6	6	10/20	1.26E9
	92.49	-.VQLLGSGSILR.-	1143.36	2	3.19	0.44	1524.8	1	19/20	1.79E9
	93.23	-.WNAIM*TVLR.-	1120.35	2	2.96	0.54	782.4	1	13/16	8.67E8
#5	Q8X966 (Q8X966) Pyr					400.32			40 (40 0 0 0 0)	4.97
	100.60 - 101.14	-.AEAAPAATGGGIPGM*LPWPK.-	1909.20	2	4.46	0.67	526.1	1	19/38	1.78E9
	82.83	-.AEGKSEFAENDAYVHATPLIR.-	2319.52	2	5.83	0.72	1170.5	1	21/40	4.31E8
	82.71 - 84.02	-.AEGKSEFAENDAYVHATPLIR.-	2319.52	3	6.33	0.67	2831.2	1	37/80	1.34E9
	40.18 - 40.94	-.AVAAALEQM*PR.-	1173.37	2	3.73	0.43	1609.1	1	17/20	2.46E8
	31.81 - 33.16	-.AVAAALEQM*PR.-	1173.37	2	3.80	0.51	1566.4	1	17/20	5.73E8
	38.05 - 39.44	-.AVAAALEQM*PR.-	1173.37	2	3.74	0.48	1413.5	1	17/20	4.89E8
	36.06 - 37.49	-.AVAAALEQM*PR.-	1173.37	2	3.90	0.51	1439.2	1	17/20	8.08E8
	33.83 - 35.62	-.AVAAALEQM*PR.-	1173.37	2	4.01	0.52	1499.3	1	17/20	9.00E8
	74.53	-.AVAAALEQMPR.-	1157.37	2	3.78	0.48	1661.7	1	18/20	6.82E8
	129.44 - 130.31	-.DVNVPDIGSDEVEVTEILVK.-	2171.39	2	3.91	0.48	498.1	1	20/38	1.17E9
	127.46 - 128.75	-.DVNVPDIGSDEVEVTEILVK.-	2171.39	2	4.38	0.53	397.4	1	19/38	7.08E9
	9.63 - 11.04	-.EAAPAAVPEAAAAK.-	1267.41	2	2.64	0.52	779.3	1	20/26	1.24E9
	100.32 - 101.50	-.EVNVPDIGGDEVEVTEVM*VK.-	2175.40	2	4.96	0.58	521.5	1	20/38	4.12E9
	112.41	-.EVNVPDIGGDEVEVTEVMVK.-	2159.40	2	3.27	0.56	312.1	1	17/38	1.69E9
	87.80 - 88.43	-.FGEIEEVELGR.-	1278.39	2	3.98	0.57	1678.6	1	18/20	3.25E9
	116.15 - 117.56	-.FITIINNTLSDIR.-	1520.76	2	4.65	0.57	1803.7	1	20/24	2.65E9
	9.18 - 9.89	-.FNSSLSEDGQR.-	1240.26	2	3.35	0.46	941.5	1	17/20	8.86E8
	51.09	-.KGIIELSR.-	916.10	1	2.33	0.28	237.8	2	9/14	2.43E8
	50.76 - 51.54	-.KGIIELSR.-	916.10	2	2.74	0.33	1119.5	1	14/14	2.77E8
	109.63	-.LM*LPISLSFDHR.-	1445.71	2	2.87	0.36	1064.0	1	18/22	1.31E9
	109.77	-.LM*LPISLSFDHR.-	1445.71	3	3.52	0.52	1233.9	1	28/44	1.02E9
	8.80	-.QEAAPAAAPAPAAGVK.-	1420.60	2	3.09	0.58	465.7	1	18/30	1.33E9
	93.63 - 94.24	-.RAEAAPAATGGGIPGM*LPWPK.-	2065.39	2	2.72	0.46	125.8	7	12/40	1.53E9
	84.67 - 85.38	-.SAM*EPVWNGKEFVPR.-	1764.00	2	3.54	0.53	887.8	1	17/28	1.25E9
	88.32 - 89.07	-.SEFAENDAYVHATPLIR.-	1934.10	2	4.71	0.62	1435.7	1	20/32	2.18E9
	89.23	-.SEFAENDAYVHATPLIR.-	1934.10	3	3.22	0.44	852.1	1	30/64	1.30E9
	87.74	-.SEFAENDAYVHATPLIR.-	1934.10	3	3.38	0.49	814.2	1	28/64	2.02E9
	86.54 - 87.84	-.SEFAENDAYVHATPLIR.-	1934.10	2	3.07	0.51	763.4	1	16/32	2.55E9
	119.60 - 120.13	-.TGSLIM*IFEVEGAAPAAAPAK.-	2061.39	2	6.05	0.73	1795.4	1	23/40	3.47E9
	112.03 - 113.46	-.TGSLIM*IFEVEGAAPAAAPAKQEAAAPAPAAK.-	3067.51	3	5.16	0.63	884.6	1	35/124	3.52E9
	117.07 - 117.71	-.TQTGALIM*IFDSADGAADAAPAQAEEK.-	2709.93	2	3.28	0.58	296.1	1	14/52	9.78E8
	111.70 - 112.73	-.TQTGALIM*IFDSADGAADAAPAQAEEKK.-	2838.10	3	3.64	0.47	745.3	1	33/108	1.90E9
	119.97 - 121.07	-.VNTGDKVSTGSLIM*VFEVAGEAGAAAPAAK.-	2879.24	3	3.96	0.43	489.8	2	29/116	3.24E9
	133.07 - 134.32	-.VPDIGADEVEITEILVK.-	1841.09	2	3.36	0.54	579.8	1	15/32	1.63E9
	134.90 - 135.10	-.VPDIGADEVEITEILVK.-	1841.09	2	3.65	0.55	888.8	1	16/32	8.04E8
	131.22	-.VPDIGADEVEITEILVK.-	1841.09	3	5.23	0.42	1466.7	1	31/64	4.95E8
	130.86 - 132.05	-.VPDIGADEVEITEILVK.-	1841.09	2	5.94	0.55	1633.6	1	24/32	5.62E9
	126.36 - 127.64	-.VSTGSLIM*VFEVAGEAGAAAPAAK.-	2264.59	2	5.86	0.67	1922.3	1	26/46	2.51E9
	114.63 - 115.87	-.VSVGDKTQTGALIM*IFDSADGAADAAPAQAEEK.-	3295.58	3	5.68	0.40	748.7	1	36/128	1.96E9
	130.13 - 130.76	-.YINIGVAVDTPNGLVVPVFK.-	2116.49	2	4.28	0.62	1416.9	1	22/38	9.44E8
#6	RPOB_ECOLI (P00575					340.35			34 (34 0 0 0 0)	3.46
	71.55 - 72.80	-.ALEIEEM*QLK.-	1220.42	2	3.37	0.34	988.2	1	14/18	1.44E9
	151.84	-.ALNYTTEQILDLFFEK.-	1946.19	3	5.40	0.54	2118.3	1	32/60	1.04E8
	55.70 - 56.86	-.AVAVDSGVTAVAK.-	1188.36	2	3.38	0.50	1546.4	1	20/24	1.16E9
	71.19	-.AVLVAGGVEAEKLDK.-	1499.74	2	3.88	0.60	1357.7	1	22/28	1.36E9
	61.26	-.AYDLGADVR.-	980.06	2	2.65	0.32	1202.7	1	14/16	4.65E8
	156.69 - 158.16	-.DLSEELQILEAGLFSR.-	1821.02	2	4.13	0.53	1385.1	1	17/30	3.56E8

	158.73 - 158.75	-.DLSEELQILEAGLFSR.-	1821.02	2	5.68	0.56	2225.5	1	20/30		3.18E8
	158.29	-.DLSEELQILEAGLFSR.-	1821.02	3	5.46	0.39	2598.5	1	32/60		8.77E7
	141.40 - 142.81	-.DQVDYM*DVSTQQVVSVGASLIPFLEHDDANR.-	3466.73	3	6.98	0.62	1410.2	1	38/120		1.51E9
	103.48	-.FIEQDPEGQYGLEAAFR.-	1971.12	2	4.50	0.62	1416.5	1	24/32		2.61E9
	93.91 - 94.14	-.FTTIHIQELACVSR.-	1675.90	2	2.60	0.39	507.7	3	13/26		1.18E9
	106.51 - 107.06	-.GDVLADGPSTDLGELALGQNM*R.-	2246.44	2	5.32	0.48	2043.7	1	25/42		2.34E9
	119.83 - 120.24	-.GSWLDFEFDPK.-	1341.45	2	2.92	0.34	555.4	1	15/20		1.85E9
	112.85	-.IETLFTNDLDHGPYISETLR.-	2335.56	3	3.58	0.59	1008.9	1	28/76		1.88E9
	130.70 - 131.75	-.INPIEDM*PYDENGTPVDIVLNPLGVPSR.-	3082.43	3	4.47	0.51	1039.6	1	32/108		3.10E9
	130.11	-.INPIEDM*PYDENGTPVDIVLNPLGVPSR.-	3082.43	2	4.18	0.53	959.5	1	21/54		7.12E8
	75.07 - 75.67	-.ITQGDDLAPGVLK.-	1327.51	2	3.36	0.51	1167.2	1	17/24		1.39E9
	140.78	-.KDLSEELQILEAGLFSR.-	1949.19	3	4.51	0.52	2012.8	1	31/64		2.43E8
	69.18	-.LGDLPTSGQIR.-	1157.30	2	3.21	0.46	890.3	1	19/20		1.01E9
	93.50 - 94.26	-.LGEPVFDVQECQIR.-	1690.87	2	3.82	0.52	1345.1	1	19/26		2.35E9
	102.94 - 103.54	-.LIEVPVEYIAGK.-	1331.58	2	3.00	0.44	720.5	1	17/22		2.37E9
	89.85	-.LSALVEIYR.-	1064.26	2	2.73	0.45	1423.6	1	15/16		5.76E8
	108.29 - 109.58	-.LSLGDLDTLM*PQDM*INAKPISAAVK.-	2675.12	3	3.98	0.50	417.7	8	24/96		5.77E9
	78.70	-.QKVDLSTFSDEEVM*R.-	1800.97	2	3.47	0.53	868.7	1	19/28		7.28E8
	53.90 - 54.51	-.RGGVVQYVDASR.-	1307.44	2	3.24	0.42	420.1	2	14/22		6.11E8
	108.17	-.RIETLFTNDLDHGPYISETLR.-	2491.74	3	3.01	0.48	824.1	1	26/80		1.01E9
	21.70 - 23.28	-.SKGESSLFSR.-	1098.19	2	3.02	0.60	686.4	1	16/18		2.05E8
	75.55 - 76.91	-.STGSYSLVTQQPLGGK.-	1623.79	2	3.66	0.27	676.0	1	16/30		3.96E9
	109.26 - 109.85	-.SVFPIQSYSGNSELQYVSYR.-	2325.52	2	5.51	0.64	2314.9	1	27/38		3.31E9
	42.94 - 44.73	-.SVGEM*AENQFR.-	1284.38	2	2.85	0.48	815.2	1	14/20		3.49E8
	41.05 - 42.13	-.SVGEM*AENQFR.-	1284.38	2	2.68	0.49	581.8	3	12/20		2.61E8
	86.46 - 87.06	-.TGEQFERPVTVGYM*YM*LK.-	2182.51	3	4.42	0.62	1468.7	1	30/68		1.69E9
	87.12	-.TGEQFERPVTVGYM*YM*LK.-	2182.51	2	2.57	0.40	128.5	4	12/34		9.62E8
	107.35 - 108.01	-.VNEDEM*YPGEAGIDIYNLTK.-	2288.47	2	5.48	0.68	649.4	1	20/38		2.41E9
#7	RPOC_ECOLI (P0057					340.31			34 (34 0 0 0 0)		3.19
	147.09 - 148.78	-.AAGEAPAAPQVTAEDASASLAELLNAGLGGSDNE	3169.31	3	3.73	0.48	357.6	9	24/132		1.68E9
	149.31 - 150.81	-.AAGEAPAAPQVTAEDASASLAELLNAGLGGSDNE	3169.31	3	5.53	0.65	771.7	1	33/132		1.75E9
	97.63 - 99.08	-.AIVQLEDGVQISSGDTLAR.-	1973.17	2	4.27	0.35	832.7	1	18/36		2.49E9
	64.13 - 65.58	-.AM*M*DNLQTETVINR.-	1668.88	2	3.87	0.46	956.2	1	15/26		1.14E9
	106.80 - 107.76	-.ASLATESFISAASFQETTR.-	2018.17	2	3.13	0.34	405.7	1	13/36		2.48E9
	96.31	-.ATIVNAGSSDFLEGEQVEYSR.-	2273.40	2	5.90	0.73	1646.3	1	22/40		1.25E9
	58.00 - 58.56	-.DITGGLPR.-	828.94	1	1.81	0.29	214.4	1	9/14		4.81E8
	74.80	-.DLLGITK.-	759.91	1	1.87	0.32	616.2	1	10/12		7.43E8
	88.69 - 89.56	-.FATSDLNDLYR.-	1315.41	2	2.75	0.56	1136.8	1	15/20		2.09E9
	36.87 - 38.09	-.FRQNLLGK.-	976.16	2	2.54	0.13	723.5	1	12/14		1.90E8
	71.67 - 72.47	-.FTDM*IDGQTITR.-	1414.57	2	3.49	0.53	646.6	1	14/22		1.58E9
	114.82 - 115.75	-.GDGEQVAGGETVANWDPHTM*PVITEVSGFVR.-	3273.54	3	5.26	0.50	1014.1	1	34/120		2.27E9
	79.75	-.GDVISDGPEAPHDILR.-	1691.82	2	3.28	0.48	768.2	1	17/30		1.05E9
	101.61	-.GEAIGVIAAQSIGEPGTQLTM*R.-	2216.50	2	2.83	0.52	189.9	5	13/42		1.36E9
	34.62 - 35.88	-.GLKENVIVGR.-	1085.28	2	3.14	0.39	662.9	2	13/18		2.60E8
	36.62 - 37.61	-.GLKENVIVGR.-	1085.28	2	2.81	0.31	592.4	2	13/18		2.03E8
	100.00	-.GLM*AKPDGSIIETPITANFR.-	2148.47	2	3.96	0.53	255.5	1	15/38		1.18E9
	145.22 - 145.74	-.HEIISEAEAEVAEIQEQFQSGLVTAGER.-	3072.29	3	5.86	0.60	852.4	1	32/108		9.41E8
	70.89 - 71.87	-.IALASPDM*IR.-	1103.32	2	2.64	0.28	561.0	2	12/18		8.76E8
	85.82	-.IFGPVKDYECLCGK.-	1686.92	2	3.92	0.53	934.8	1	16/26		1.56E9
	63.39	-.ITEYEKDANGELVAK.-	1680.84	1	1.86	0.32	292.7	8	10/28		2.13E8
	56.38 - 57.62	-.ITEYEKDANGELVAK.-	1680.84	2	4.39	0.63	1525.3	1	18/28		1.66E9

	117.21 - 117.76	-.LGIQAFEPVLIEGK.-	1514.79	2	3.58	0.58	713.9	1	16/26	3.87E9
	60.54 - 61.10	-.LIPAGTGYAYHQDR.-	1562.71	2	3.39	0.51	561.3	1	19/26	8.31E8
	94.47 - 95.01	-.LVITPVDGSDPYEEM*IPK.-	2020.29	2	4.13	0.55	614.6	1	17/34	1.96E9
	92.42	-.M*GAEAIQALLK.-	1161.40	2	3.05	0.41	2148.9	1	18/20	1.06E9
	120.42 - 120.83	-.QTDELTGLSSLVVLDSAER.-	2034.21	2	5.12	0.65	1488.5	1	21/36	1.99E9
	141.38 - 141.91	-.RAAGEAPAAPQVTAEDASASLAELLNAGLGGSDN	3325.50	3	6.29	0.70	1874.6	1	39/136	8.17E8
	67.53	-.SGASVGIDDM*VIPEKK.-	1662.89	2	3.47	0.48	788.3	1	17/30	1.19E9
	101.09	-.SM*DLEQECEQLREELNETNSETK.-	2829.94	3	3.16	0.57	928.5	1	27/88	9.82E8
	72.57	-.VADLFEAR.-	921.03	2	2.77	0.24	1154.5	1	13/14	3.90E8
	96.66	-.VIDIWAAANDR.-	1244.38	2	3.94	0.50	1796.5	1	18/20	9.52E8
	101.65 - 101.67	-.VTAEDVLKPGTADILVPR.-	1895.19	2	4.38	0.50	461.1	1	19/34	2.09E9
	101.52 - 102.36	-.VTAEDVLKPGTADILVPR.-	1895.19	3	4.22	0.49	1480.3	1	33/68	2.34E9
#8	Q8XBW7 (Q8XBW7) C					250.28			25 (25 0 0 0 0)	1.69
	94.04	-.AGNAQTTGDLAGANYIAGAGAYTYNEPGR.-	2845.98	2	4.43	0.50	1096.2	1	22/56	8.34E8
	94.01	-.AGNAQTTGDLAGANYIAGAGAYTYNEPGR.-	2845.98	3	5.63	0.56	1625.6	1	36/112	1.39E9
	51.66 - 52.71	-.AGTYATTLPAGR.-	1179.31	2	2.53	0.48	952.7	1	16/22	6.36E8
	95.51	-.APSLYQTNPNYILYSK.-	1873.10	2	4.41	0.61	1017.8	1	19/30	2.32E9
	59.07 - 60.38	-.DVSEIIR.-	831.94	1	1.82	0.17	133.3	13	6/12	9.60E8
	128.54 - 129.42	-.EISPYSIVGLSATWDVTK.-	1967.21	2	4.17	0.53	848.6	1	19/34	1.58E9
	85.47	-.GM*GPENTLILIDGKPVSSR.-	2001.29	2	3.10	0.43	344.5	2	13/36	9.39E8
	81.64	-.GQGCYASAGGCYLQGNDDLKAETSINK.-	2879.03	3	4.44	0.48	1456.8	1	36/104	7.40E8
	28.83 - 30.05	-.IPEGLAGGTEGK.-	1129.25	2	2.83	0.57	1149.2	1	18/22	3.25E8
	30.64 - 31.94	-.IPEGLAGGTEGK.-	1129.25	2	3.37	0.49	1509.6	1	18/22	4.08E8
	123.56 - 124.75	-.LSIIPEYTLNSTLSWQAR.-	2093.37	2	4.58	0.58	455.5	1	15/34	1.28E9
	72.45	-.M*KDLSSNTQALTGTNTGGAIDGVSATDR.-	2798.98	3	5.04	0.58	1116.4	1	32/108	7.33E8
	75.99	-.M*KDLSSNTQALTGTNTGGAIDGVSATDR.-	2798.98	2	3.22	0.47	505.9	1	18/54	4.76E8
	75.92 - 76.87	-.M*KDLSSNTQALTGTNTGGAIDGVSATDR.-	2798.98	3	4.70	0.50	1329.5	1	34/108	1.70E9
	76.11 - 77.18	-.MKDLSSNTQALTGTNTGGAIDGVSATDR.-	2782.98	3	3.10	0.21	522.0	13	23/108	1.46E9
	96.58 - 97.59	-.NVSLTGGVDNLFDKR.-	1635.80	2	3.83	0.59	1205.0	1	19/28	1.71E9
	57.71	-.QGNLYAGDTQNTNSDAYTR.-	2090.11	2	4.08	0.67	384.5	1	13/36	5.07E8
	40.42 - 42.15	-.TM*PGVNLTGNSTSGQR.-	1636.77	2	2.92	0.61	400.8	1	16/30	2.60E8
	38.60 - 39.46	-.TM*PGVNLTGNSTSGQR.-	1636.77	2	3.09	0.54	321.3	1	16/30	1.63E8
	36.74	-.TM*PGVNLTGNSTSGQR.-	1636.77	2	3.22	0.63	352.2	1	18/30	1.79E8
	44.75	-.TM*PGVNLTGNSTSGQR.-	1636.77	2	2.68	0.56	775.5	1	18/30	2.60E8
	42.68 - 43.82	-.TM*PGVNLTGNSTSGQR.-	1636.77	2	3.62	0.66	496.4	1	20/30	2.45E8
	124.01 - 124.61	-.TNFSLTGPLGDEFSFR.-	1788.94	2	4.35	0.62	794.0	1	19/30	2.73E9
	55.45 - 55.94	-.TQADAWDINQGHQSAR.-	1798.86	2	3.11	0.22	378.0	2	12/30	3.88E8
	88.76	-.YGNGAAGGVVNIITK.-	1434.62	2	3.66	0.51	1161.8	1	20/28	1.98E9
#9	EAE_ECO57 (P43261)					240.36			24 (24 0 0 0 0)	3.68
	76.60 - 77.28	-.AAPGQQIILPLKK.-	1377.70	2	2.54	0.32	309.7	1	14/24	1.33E9
	94.97	-.ALNYAAQQAASLGSQLQSR.-	1978.16	2	4.24	0.57	1129.1	1	18/36	1.38E9
	95.14	-.ALNYAAQQAASLGSQLQSR.-	1978.16	3	4.78	0.54	1836.2	1	32/72	1.20E9
	29.85 - 30.62	-.ATITLTSSSAGK.-	1137.27	2	3.21	0.53	1026.1	1	18/22	5.62E8
	27.63 - 29.33	-.ATITLTSSSAGK.-	1137.27	2	3.34	0.59	1333.0	1	20/22	6.52E8
	9.36	-.ATSGDKQTVSYTIK.-	1499.65	2	3.53	0.45	798.5	1	20/26	1.09E9
	29.48 - 30.77	-.ATVSATVSDGAEVK.-	1335.44	2	2.60	0.50	873.7	1	18/26	4.28E8
	54.53 - 55.17	-.ATVSATVSDGAEVK.-	1335.44	2	2.90	0.11	660.9	1	16/26	4.10E8
	134.34 - 135.88	-.FFLPANM*LGYNVFIDQDFSGDNTR.-	2799.07	2	3.52	0.48	522.0	1	15/46	9.39E9
	99.85 - 100.91	-.FNGYLPSYPALGAK.-	1498.71	2	2.53	0.45	270.8	1	15/26	9.08E9
	37.51 - 39.37	-.FTANLGAGQR.-	1035.14	2	2.85	0.32	905.1	1	15/18	3.29E8
	121.24 - 122.16	-.GELPNIWLQYGQFK.-	1693.93	2	3.29	0.55	814.5	1	17/26	2.36E9

	122.76	-.GELPNIWLQYGQFK.-	1693.93	2	3.64	0.64	777.5	1	16/26	2.29E9
	77.51	-.HGTGNENDLLYSM*QFR.-	1899.03	2	2.89	0.51	320.1	1	13/30	1.01E9
	105.82	-.KNGVAQANVPVSFNIVSGTATLGANSAK.-	2717.03	2	4.37	0.57	611.4	1	21/54	1.06E9
	134.20 - 135.39	-.LPFEYSALPLLGSAPLVAAGGVAGHTNK.-	2752.16	3	7.10	0.51	2136.6	1	40/108	5.08E9
	119.61 - 120.79	-.LQSNPGAATVGVNYTPIPLVTM*GIDYR.-	2865.25	3	5.43	0.50	2055.1	1	38/104	2.20E9
	119.80	-.LQSNPGAATVGVNYTPIPLVTM*GIDYR.-	2865.25	2	3.66	0.54	224.1	1	12/52	8.18E8
	74.30 - 75.65	-.NNNIILEYK.-	1121.27	2	2.60	0.32	884.5	1	12/16	1.35E9
	128.42	-.SGVSSTYNLITQNPLPGVNVNTPNVYAVCVE.-	3308.64	3	3.99	0.40	868.7	1	31/120	1.13E9
	116.27 - 116.88	-.SQDINLSTIWSLNK.-	1619.80	2	3.89	0.60	1512.1	1	19/26	1.69E9
	100.54 - 101.12	-.SQGGQIQHSGSQSAQDYQAILPAYVQGGSNIYK.-	3482.72	3	4.70	0.55	530.7	1	33/128	3.86E9
	96.60	-.SWSQQIEPQYVNELR.-	1878.04	2	4.18	0.48	525.5	1	15/28	2.39E9
	115.66 - 116.86	-.TAEM*TSALNASAVIFFDQTK.-	2162.41	2	4.47	0.61	1601.9	1	23/38	1.74E9
#10	Q8XCU8 (Q8XCU8) Pl					230.34			23 (23 0 0 0 0)	1.82
	119.45 - 120.05	-.ATVFIFPDLNTGNTTYK.-	1903.12	2	4.21	0.60	620.3	1	19/32	2.26E9
	117.64 - 118.23	-.ATVFIFPDLNTGNTTYK.-	1903.12	2	3.99	0.48	782.3	1	20/32	3.33E9
	102.03	-.DAEVVLVEGLVPTRK.-	1625.89	2	3.31	0.33	1575.0	1	18/28	1.06E9
	169.71 - 169.86	-.GALVDDIVYTIALTAIQSAQQQ.-	2319.60	3	6.75	0.57	3825.7	1	39/84	4.75E8
	97.71 - 98.31	-.GIATCVLLGNPAEINR.-	1698.94	2	4.09	0.38	1174.1	1	19/30	2.06E9
	73.67 - 74.22	-.HLNATIINEGDINTR.-	1681.83	2	4.22	0.55	1294.4	1	20/28	1.65E9
	74.91	-.HQFAQSLNYEIAK.-	1549.71	2	3.27	0.41	1183.5	1	19/24	9.07E8
	132.60 - 133.83	-.IIM*LIPTGTSVGLTSVSLGVIR.-	2244.72	2	5.41	0.68	691.0	1	23/42	1.05E9
	65.64 - 65.83	-.IVLPEGDEPR.-	1125.26	2	2.60	0.48	620.6	2	15/18	1.01E9
	67.98 - 68.19	-.KHQFAQSLNYEIAK.-	1677.89	2	3.69	0.49	1177.9	1	19/26	5.27E8
	68.06	-.KHQFAQSLNYEIAK.-	1677.89	3	3.37	0.38	1085.6	1	26/52	5.21E8
	154.52 - 155.05	-.LQESSPLPVLGAVPWSFDLIATR.-	2497.87	3	5.64	0.53	1326.5	1	37/88	6.77E8
	74.49 - 75.09	-.LSVFKPIAQPR.-	1256.52	2	2.68	0.58	452.4	1	15/20	1.08E9
	66.43 - 66.96	-.NTNITGVIVNK.-	1173.35	2	3.15	0.28	1414.3	1	17/20	1.24E9
	12.29 - 13.55	-.TGGDAPDQTTTIVR.-	1432.52	2	3.22	0.45	563.9	1	18/26	4.37E8
	14.28 - 14.86	-.TGGDAPDQTTTIVR.-	1432.52	2	2.82	0.40	713.1	1	18/26	1.49E8
	10.53 - 11.71	-.TGGDAPDQTTTIVR.-	1432.52	2	3.54	0.50	680.9	1	19/26	7.94E8
	90.73 - 90.75	-.TRPDLSEIFDDSSK.-	1610.70	2	3.17	0.06	819.6	2	16/26	1.67E9
	113.71	-.VAASQGVELGAGIEIVDPEVVR.-	2209.49	2	2.55	0.37	435.7	5	14/42	1.25E9
	115.68	-.VAASQGVELGAGIEIVDPEVVR.-	2209.49	2	3.65	0.57	518.8	1	14/42	1.16E9
	63.19 - 64.45	-.VAM*LSYSTGTSGAGSDVEK.-	1877.02	2	5.47	0.59	1207.8	1	20/36	1.59E9
	74.68	-.VAMLSYSTGTSGAGSDVEK.-	1861.02	2	2.73	0.46	390.1	2	13/36	5.82E8
	73.63	-.YQLTELAR.-	994.13	2	2.72	0.32	667.8	1	13/14	7.21E8
#11	CARB_ECO57 (P6373					210.28			21 (21 0 0 0 0)	1.98
	95.16 - 95.70	-.ALEAAGVPVIGTSPDAIDR.-	1853.07	2	3.64	0.61	294.4	1	16/36	2.51E9
	83.47 - 84.81	-.EIGVETGGSNVQFAVNPK.-	1847.02	2	4.09	0.65	1147.4	1	19/34	2.30E9
	104.82 - 105.98	-.ERPDAVLPTM*GGQTALNCALELER.-	2658.96	3	4.60	0.61	1105.0	1	29/92	2.23E9
	76.76	-.GLM*NVQFAVK.-	1123.35	2	3.00	0.32	809.9	1	13/18	1.06E9
	77.30	-.GVIVQYGGQTPLK.-	1360.58	1	2.41	0.35	396.9	1	14/24	8.45E8
	77.02 - 78.45	-.GVIVQYGGQTPLK.-	1360.58	2	3.83	0.60	1615.0	1	19/24	2.10E9
	82.93	-.IKNGEYTYIINTTSGR.-	1831.02	3	3.14	0.30	1364.5	1	27/60	7.79E8
	82.91	-.IKNGEYTYIINTTSGR.-	1831.02	2	3.88	0.59	1078.0	1	19/30	8.77E8
	49.04 - 50.27	-.IM*VLGGGPNR.-	1030.23	2	2.72	0.40	854.2	3	14/18	5.24E8
	116.39	-.IWYIADAFR.-	1155.33	2	2.56	0.36	677.1	1	14/16	6.69E8
	125.58 - 126.42	-.LAVGYTLDELM*NDITGGR.-	1955.18	2	5.15	0.56	1576.8	1	21/34	1.83E9
	105.09	-.LAVGYTLDELM*NDITGGR.-	1955.18	2	4.88	0.59	1760.5	1	22/34	8.63E8
	151.77	-.LAVGYTLDELMNDITGGR.-	1939.18	2	5.52	0.68	1700.4	1	21/34	2.06E8
	99.87	-.LKQPANATVTAIEM*AVEK.-	1931.24	2	4.43	0.66	865.6	1	19/34	1.08E9

	100.02	-.LKQPANATVTAIEM*AVEK.-	1931.24	3	4.14	0.45	1140.4	1	30/68	9.96E8
	160.56 - 161.09	-.LYFEPVTLEDVLEIVR.-	1936.24	2	4.64	0.51	1015.2	1	22/30	1.54E8
	119.89 - 120.49	-.QGVLEEFGVTM*IGATADAIDKAEDR.-	2653.90	3	3.01	0.38	497.2	7	22/96	1.76E9
	30.32 - 31.53	-.SVGEVM*AIGR.-	1035.20	2	3.04	0.61	750.7	1	15/18	3.52E8
	95.64 - 96.52	-.TPASFEPSIDYVVTK.-	1654.84	2	4.31	0.65	587.3	1	18/28	3.39E9
	110.80 - 111.43	-.VAEVGITGLNAEFLR.-	1589.82	2	2.94	0.63	1008.8	1	18/28	2.63E9
	76.93	-.VSLDDPEALTK.-	1188.31	2	3.35	0.50	1264.9	1	17/20	1.35E9
#12	Q8X9M3 (Q8X9M3) Pc					210.26			21 (21 0 0 0 0)	1.90
	99.73 - 100.08	-.AKPGQDFFPLTVNYQER.-	2011.23	3	4.65	0.50	1869.5	1	31/64	1.67E9
	99.98 - 100.62	-.AKPGQDFFPLTVNYQER.-	2011.23	2	3.26	0.47	419.0	1	14/32	1.31E9
	81.97 - 83.38	-.ALTEETGTTIEIEDDGTVK.-	2023.14	2	4.45	0.58	833.7	1	16/36	1.71E9
	82.67 - 83.53	-.DGISALQM*DIK.-	1207.38	2	2.60	0.45	924.0	1	14/20	1.16E9
	70.71 - 71.79	-.EGLVHISQIADK.-	1310.48	2	3.05	0.49	1145.8	1	17/22	1.23E9
	65.92 - 66.69	-.EGLVHISQIADKR.-	1466.67	2	2.72	0.42	991.8	1	15/24	8.55E8
	34.07 - 35.50	-.EGRPSEGETLIAR.-	1415.54	2	3.49	0.39	646.5	1	16/24	3.75E8
	36.08 - 37.76	-.EGRPSEGETLIAR.-	1415.54	2	3.07	0.35	747.2	1	16/24	2.96E8
	31.68 - 33.52	-.EGRPSEGETLIAR.-	1415.54	2	3.33	0.42	769.7	1	16/24	6.29E8
	47.76 - 49.22	-.EIM*QVALNQAK.-	1261.47	2	2.84	0.43	542.6	1	14/20	8.25E8
	49.75 - 50.99	-.EIM*QVALNQAK.-	1261.47	2	3.11	0.44	454.6	1	13/20	6.72E8
	75.94	-.EIMQVALNQAK.-	1245.48	2	3.26	0.33	1206.9	1	17/20	6.66E8
	87.59	-.GETQALVTATLGTAR.-	1489.66	2	3.57	0.54	1146.6	1	17/28	2.05E9
	67.71 - 68.60	-.INPDKIKDVIGK.-	1340.59	2	2.65	0.49	641.3	1	16/22	9.65E8
	115.64 - 116.70	-.TDTFLFHYNFPPYSVGETGM*VGSPK.-	2809.10	3	5.29	0.57	1369.6	1	33/96	2.05E9
	100.68 - 101.56	-.VGYINDQYVLNPTQDELK.-	2110.31	2	4.74	0.60	798.8	1	19/34	2.25E9
	92.70 - 93.61	-.VGYINDQYVLNPTQDELKESK.-	2454.68	3	4.07	0.50	766.8	1	30/80	1.71E9
	75.19 - 75.86	-.VTDYLQM*GQEVPVK.-	1623.85	2	4.21	0.60	841.6	1	18/26	2.30E9
	108.03 - 109.19	-.VVSEITESNGSSSM*ASVCGASLALM*DAGVPIK.-	3201.56	3	5.22	0.54	578.4	1	32/124	2.45E9
	106.00	-.WDWQPEPVNEALNAR.-	1825.96	2	3.08	0.54	430.5	1	17/28	1.51E9
	56.68 - 57.22	-.YAQVDVIK.-	936.09	1	2.09	0.27	642.6	2	10/14	6.25E8
#13	SYA_ECO57 (Q8X3W8					200.25			20 (20 0 0 0 0)	1.84
	84.54 - 85.12	-.AGELIGM*VAQQVGGK.-	1474.71	2	2.64	0.39	402.6	2	13/28	2.42E9
	79.89	-.AVEDLVNAQIR.-	1228.38	2	3.96	0.47	1564.1	1	17/20	9.48E8
	67.44	-.FDFSHNEAM*KPEEIR.-	1867.03	2	2.72	0.48	660.6	1	15/28	7.21E8
	89.27	-.GGGRPDM*AQAGGTDAAALPAALASVK.-	2370.63	3	3.11	0.37	1484.6	1	37/100	1.31E9
	100.95	-.GLALLDEELAK.-	1172.35	2	3.03	0.39	957.2	1	15/20	1.15E9
	110.24	-.IAAVLQHVNSNYDIDLFR.-	2089.34	2	4.90	0.52	1150.0	1	20/34	1.23E9
	110.28	-.IAAVLQHVNSNYDIDLFR.-	2089.34	3	3.18	0.56	1366.5	1	25/68	9.03E8
	71.21	-.IEAVTGEGAITTVHADSDR.-	1943.06	2	3.34	0.39	494.0	1	18/36	5.87E8
	90.61 - 91.45	-.LLVSELSGVEPK.-	1271.49	2	3.27	0.01	1630.9	2	18/22	1.97E9
	76.38 - 77.32	-.LSGDTLDGETAFR.-	1382.46	2	3.09	0.46	977.2	1	18/24	1.61E9
	118.40 - 119.74	-.LYDTYGFPVDLTADVCR.-	2006.20	2	4.26	0.52	464.7	1	18/32	6.87E9
	105.80 - 106.94	-.NLPIETNIM*DLEAAK.-	1688.93	2	3.16	0.53	172.7	6	12/28	2.16E9
	103.01	-.QQAQVEQVLKTEEEQFAR.-	2162.35	2	3.14	0.53	237.1	1	13/34	6.81E8
	67.27	-.RIEAVTGEGAITTVHADSDR.-	2099.25	2	4.65	0.50	642.4	1	20/38	5.26E8
	55.72	-.TLIQAVAK.-	844.03	2	2.53	0.25	680.2	1	13/14	2.23E8
	149.66 - 150.27	-.TM*VDDLKNQLGSTIIVLATVAEGK.-	2533.92	3	4.21	0.54	1625.2	1	34/92	4.51E8
	60.47 - 61.06	-.VDEAGFEAAM*EEQR.-	1598.67	2	3.92	0.54	1691.5	1	20/26	6.75E8
	53.38	-.VGDAVQADVDEAR.-	1345.40	2	3.88	0.22	1365.6	1	20/24	6.29E8
	51.56 - 52.73	-.VGDAVQADVDEAR.-	1345.40	2	3.76	0.58	1162.9	1	19/24	7.76E8
	70.69	-.VTALFVDGK.-	950.11	2	2.84	0.51	372.3	3	13/16	6.09E8
#14	CLPB_ECO57 (P63285					180.25			18 (18 0 0 0 0)	1.92

	82.34 - 82.89	-.AAGATTANITQAIEQM*R.-	1763.96	2	5.08	0.65	1377.0	1	20/32	1.32E9
	120.67 - 121.73	-.AGLADPNRPIGSFLFLGPTGVGK.-	2285.63	2	3.22	0.57	178.2	4	12/44	1.30E9
	109.23	-.AIDLIDEAASSIR.-	1374.52	2	4.56	0.53	1874.6	1	19/24	1.33E9
	92.82	-.ALANFM*FDSDEAM*VR.-	1749.95	2	3.32	0.52	746.6	2	15/28	1.83E9
	74.16	-.GELHCVGATTLDEYR.-	1721.84	2	4.19	0.63	1745.3	1	21/28	9.91E8
	80.22	-.GYEIHISDEALK.-	1375.51	2	4.05	0.56	1549.7	1	18/22	1.12E9
	72.58	-.IINGEVPEGLK.-	1169.35	2	2.56	0.24	325.1	2	13/20	9.96E8
	79.25 - 80.53	-.LPQVEGTGGDVQPSQDLVR.-	1996.17	2	3.32	0.46	385.6	1	13/36	2.69E9
	81.12	-.LPQVEGTGGDVQPSQDLVR.-	1996.17	2	4.35	0.59	858.2	1	18/36	1.97E9
	105.86	-.LVGAPPGYVGYEEGGYLTEAVR.-	2298.54	2	3.45	0.47	332.0	1	20/42	1.87E9
	72.24	-.NNPVLIGEPGVGK.-	1294.48	2	3.24	0.65	1094.0	1	17/24	1.35E9
	99.35 - 100.58	-.NTVVIM*TSNLGSDLIQER.-	2007.26	2	3.63	0.07	484.3	2	14/34	1.64E9
	64.18	-.TAIVEGLAQR.-	1058.21	2	3.77	0.37	1886.6	1	17/18	5.94E8
	30.13 - 31.29	-.TDINQALNR.-	1045.13	2	2.74	0.41	1117.0	1	14/16	3.94E8
	121.61 - 122.18	-.VFVAEPSVEDTIAILR.-	1760.02	2	4.24	0.39	763.2	1	22/30	2.47E9
	93.99 - 95.20	-.VIGQNEAVDAVSNAIR.-	1656.82	2	4.73	0.53	1833.8	1	21/30	3.82E9
	87.28	-.VLALDM*GALVAGAK.-	1345.63	2	3.59	0.60	872.1	1	17/26	1.06E9
	97.65	-.VTDAEIAEVLAR.-	1287.45	2	3.88	0.48	2358.4	1	19/22	7.93E8
#15	PUR4_ECO57 (Q8XA4					170.30			17 (17 0 0 0 0)	1.22
	99.63	-.AGLVGFSVSNLR.-	1220.40	2	3.56	0.58	1219.8	1	18/22	1.05E9
	110.49 - 110.69	-.APVALLDFAASAR.-	1302.50	2	4.30	0.69	2182.0	1	20/24	1.33E9
	113.89 - 114.52	-.AVGEELCPALGLTIPVGK.-	1825.13	2	3.95	0.50	1004.5	1	20/34	2.31E9
	106.53	-.EAVESVLAQHGLADCVHYVGQAVSGDR.-	2869.09	3	3.33	0.33	332.0	111	21/104	7.58E8
	150.19 - 150.85	-.EM*TSPLSLVISAFAR.-	1638.91	2	3.67	0.49	929.7	1	16/28	2.19E8
	128.24	-.GFYDAIQALVAQR.-	1452.64	2	3.67	0.40	1805.0	1	20/24	9.71E8
	134.82 - 135.55	-.HFDNQPIDLPLDVLLGK.-	1935.21	2	4.28	0.63	948.1	1	19/32	6.72E8
	134.74 - 135.29	-.HFDNQPIDLPLDVLLGK.-	1935.21	3	4.15	0.52	1845.3	1	36/64	5.86E8
	104.45	-.IPGFEQPWEEDFGKPER.-	2062.23	3	3.66	0.49	920.6	1	28/64	8.99E8
	123.88	-.LAVGEALTNIAATQIGDIK.-	1899.18	2	4.30	0.53	1428.1	1	19/36	1.03E9
	117.13 - 117.84	-.LAVGEALTNIAATQIGDIKR.-	2055.37	2	4.14	0.54	916.5	1	17/38	1.03E9
	149.07 - 150.59	-.LGLALAEDEIDYLQDAFTK.-	2126.35	2	6.03	0.60	1892.7	1	24/36	1.70E9
	106.96 - 108.21	-.LSFDINEDVAAPYIATGARPK.-	2249.51	3	3.54	0.53	653.5	2	26/80	1.75E9
	91.37	-.NTFETTPDHVLSAYKDNAAVM*EGSEVGR.-	3056.27	3	3.99	0.41	1028.4	1	31/108	1.27E9
	87.50	-.TFLVTIGDR.-	1022.18	2	2.59	0.36	1289.7	1	14/16	6.41E8
	138.29	-.YVLAVAADQLPLFDELCK.-	2066.38	2	4.40	0.48	1128.6	1	22/34	5.42E8
	130.84	-.YVLAVAADQLPLFDELCKR.-	2222.56	3	4.43	0.60	707.9	1	26/72	8.31E8
#16	IF2_ECOLI (P02995) T					160.25			16 (16 0 0 0 0)	1.15
	45.95 - 47.56	-.AAM*SGM*LSPELK.-	1267.50	2	3.24	0.49	603.2	1	13/22	4.64E8
	48.08 - 49.51	-.AAM*SGM*LSPELK.-	1267.50	2	3.51	0.53	554.9	2	13/22	5.99E8
	69.95	-.AAQVPVVVAVNK.-	1195.44	1	1.82	0.31	708.3	1	12/22	8.58E8
	69.93	-.AAQVPVVVAVNK.-	1195.44	2	2.73	0.34	644.6	1	17/22	9.49E8
	115.44	-.ADVQGSVEAISDSLLK.-	1632.80	2	4.16	0.54	1118.4	1	18/30	2.00E9
	80.20 - 81.36	-.FGAIAGCM*VTEGVVK.-	1555.82	2	3.49	0.31	1130.9	1	19/28	1.87E9
	64.73 - 64.85	-.GPVATVLVR.-	912.11	2	3.40	0.46	1159.4	1	14/16	4.28E8
	59.95 - 61.35	-.GSSLQQGFQKPAQAVNR.-	1817.00	2	3.91	0.52	724.6	1	17/32	8.09E8
	153.54 - 154.84	-.IIGSGVGGITETDATLAAASNAILVGFNVR.-	2889.25	3	5.09	0.59	1097.8	1	34/116	4.05E8
	39.10	-.KGSSLQQGFQKPAQAVNR.-	1945.17	3	3.03	0.43	479.7	3	24/68	1.63E8
	69.69 - 70.48	-.KVIEAESLDLR.-	1273.46	2	2.92	0.51	1119.9	1	17/20	8.94E8
	126.17 - 127.34	-.LGAM*ATINQVIDQETAQLVAEEM*GHK.-	2831.17	3	4.85	0.51	1195.5	1	35/100	3.68E9
	138.79 - 140.10	-.LGAMATINQVIDQETAQLVAEEM*GHK.-	2815.17	3	3.93	0.39	486.5	2	27/100	7.05E8
	100.45 - 101.05	-.SKLENM*FANM*TEGEVHEVNIVLK.-	2666.03	3	4.34	0.51	1097.3	1	29/88	1.21E9

	105.51	-.TSLLDYIR.-	981.13	2	2.58	0.43	968.0	1	13/14	5.88E8
	133.58	-.YYSVIYNLIDEVK.-	1619.84	2	3.84	0.57	1596.1	1	21/24	8.99E8
#17	UP05_ECOLI (P39170					140.26			14 (14 0 0 0 0)	1.08
	93.85	-.ALFATGNFEDVR.-	1340.47	2	3.27	0.62	1015.8	1	15/22	1.05E9
	88.20	-.AVYFPHQASNYDPDYDYECATQDGAK.-	3027.11	3	5.28	0.55	1784.7	1	35/100	9.37E8
	76.48	-.DGDTLLVQVK.-	1088.24	1	1.80	0.02	528.6	1	11/18	4.28E8
	77.69	-.ERPTIASITFSGNK.-	1521.70	2	3.99	0.60	1132.5	1	18/26	7.56E8
	81.72	-.FNIDSTQVSLTPDKK.-	1693.88	3	3.09	0.26	581.7	2	26/56	5.89E8
	105.43	-.LFYNDFQADDADLSDYTNK.-	2256.32	2	4.23	0.59	1097.3	1	21/36	9.50E8
	107.08	-.LFYNDFQADDADLSDYTNK.-	2256.32	2	4.72	0.49	955.3	1	18/36	8.24E8
	114.00 - 115.42	-.SYGTDVTLGFPINEYNSLR.-	2147.33	2	3.13	0.43	675.8	1	17/36	2.17E9
	118.03 - 118.61	-.SYGTDVTLGFPINEYNSLR.-	2147.33	2	3.60	0.55	410.8	1	16/36	1.85E9
	68.89 - 69.65	-.TGDTVNDEDISNTIR.-	1650.68	2	2.81	0.27	961.6	1	17/28	1.88E9
	70.40 - 71.10	-.TGDTVNDEDISNTIR.-	1650.68	2	3.43	0.46	668.8	1	17/28	1.83E9
	87.40	-.VAVGAALLSM*PVR.-	1300.60	2	2.93	0.43	754.5	1	13/24	1.09E9
	70.63	-.VPGSPDQVDVVYK.-	1403.56	2	3.56	0.64	1133.0	1	18/24	7.86E8
	64.07	-.YLYSM*GEHPSTSDQDNSFK.-	2223.32	2	2.81	0.51	425.9	1	16/36	4.06E8
#18	ENTF_ECO57 (Q8XBV					140.26			14 (14 0 0 0 0)	0.84
	114.23	-.AAGDEPLFGPVLNIK.-	1541.77	2	3.59	0.43	776.5	1	18/28	1.35E9
	78.57	-.AVVAGLAQADTLR.-	1285.48	2	3.80	0.54	1991.2	1	19/24	1.02E9
	65.81	-.EHGVKPGDSVAVALPR.-	1632.85	2	3.11	0.59	421.9	1	17/30	8.59E8
	138.99 - 139.61	-.GEQTPASPFTPFADVVEEYQQYR.-	2660.83	3	5.26	0.54	1351.6	1	31/88	7.97E8
	139.15	-.GEQTPASPFTPFADVVEEYQQYR.-	2660.83	2	3.68	0.50	609.1	1	18/44	7.26E8
	56.04 - 57.40	-.GVM*VGQTAIVNR.-	1261.48	2	3.61	0.42	1575.0	1	17/22	4.63E8
	57.96	-.GVM*VGQTAIVNR.-	1261.48	2	3.86	0.44	2008.7	1	17/22	5.28E8
	149.83	-.LGSAALTATGPVLNVLPLGIHIAAQETLPELATR.-	3409.96	3	3.72	0.36	325.8	3	24/132	2.69E8
	96.23 - 97.51	-.M*M*LEDARPSLLITTDDQLPR.-	2348.68	3	3.76	0.52	1238.8	1	28/76	1.68E9
	89.13	-.QVFCSGEALPADLCR.-	1723.90	2	2.68	0.33	539.4	1	14/28	1.29E9
	90.65 - 91.24	-.TNIDPHGTAQALM*QADLQQDLR.-	2453.68	3	4.98	0.58	2235.9	1	35/84	1.02E9
	51.80 - 52.96	-.TPDAPALADAR.-	1098.19	2	3.28	0.46	813.7	1	16/20	4.81E8
	102.38 - 103.44	-.VM*QALPDVEQAVTHACVINQAAATGGDAR.-	3011.31	3	3.32	0.20	221.0	10	24/112	1.30E9
	56.34	-.YDAEQIVR.-	994.08	2	2.57	0.40	1155.7	1	14/14	3.45E8
#19	Q8XA49 (Q8XA49) Iso					130.25			13 (13 0 0 0 0)	1.00
	111.86 - 112.60	-.ANDIVVALLQEK.-	1313.53	2	3.19	0.48	913.8	1	16/22	1.86E9
	78.90	-.AYEAYDFHEVVQR.-	1627.74	2	5.00	0.56	2340.4	1	21/24	1.17E9
	78.67	-.EILGDEADQYVK.-	1380.48	2	3.18	0.46	1562.8	1	18/22	1.28E9
	101.89	-.IGVTDYTILGTVK.-	1380.61	2	3.90	0.59	1348.6	1	18/24	1.16E9
	72.26	-.LTALGDELR.-	988.12	2	2.56	0.28	911.5	1	14/16	4.64E8
	66.41	-.QVLTHGFTVDGQGR.-	1515.66	2	2.92	0.57	466.8	1	14/26	8.12E8
	117.48	-.SCQTALYHIAEALVR.-	1732.96	2	3.22	0.57	921.1	1	15/28	8.82E8
	46.09 - 47.69	-.SIGNTVSPQDVM*NK.-	1506.66	2	2.86	0.45	232.4	1	11/26	3.36E8
	67.73	-.TLELM*EEVAK.-	1179.37	2	2.83	0.38	632.4	1	13/18	6.31E8
	111.06	-.TSPSIDVAFQAVDQDALK.-	1906.08	2	4.54	0.65	929.3	1	19/34	2.11E9
	127.03 - 127.60	-.YGLETANPVGPDGTYLPGTYPTLDGVNVFK.-	3157.48	3	4.26	0.36	649.1	1	29/116	1.99E9
	127.19	-.YGLETANPVGPDGTYLPGTYPTLDGVNVFK.-	3157.48	2	3.65	0.51	265.7	1	17/58	7.36E8
	124.95	-.YGLETANPVGPDGTYLPGTYPTLDGVNVFK.-	3157.48	3	3.62	0.54	611.9	4	28/116	9.81E8
#20	GCSP_ECO57 (Q8XD:					120.21			12 (12 0 0 0 0)	1.06
	52.48	-.AEAAEINLR.-	987.09	2	2.76	0.31	1360.8	1	14/16	4.06E8
	91.47 - 91.66	-.ASQVAILNANYIASR.-	1591.79	2	4.24	0.48	1013.6	1	17/28	1.18E9
	95.66 - 96.68	-.FFVASDVHPQTLDVVR.-	1831.06	2	3.39	0.53	758.6	1	19/30	3.26E9
	116.17 - 117.44	-.HIGPDAAQQQEM*LNAVGAQSLNALTGQIVPK.-	3217.60	3	4.14	0.51	632.7	1	35/120	2.73E9

	111.08	-.LIDYGFHAPTM*SFPVAGTLM*VEPTESESK.-	3188.57	3	3.98	0.50	971.8	1	32/112	1.69E9
	99.89	-.LQDAFPVLYTGR.-	1380.57	2	2.70	0.34	776.7	1	15/22	8.94E8
	108.94	-.LTGYDAVCM*QPNSGAQGEYAGLLAIR.-	2773.07	2	3.67	0.51	235.5	1	14/50	4.01E8
	104.33	-.NM*LENPGWYTAYTPYQPEVSQGR.-	2718.94	2	3.69	0.47	653.7	1	17/44	9.06E8
	137.65	-.QGAVSAAPFGSASILPISWM*YIR.-	2439.82	3	3.99	0.44	895.6	1	32/88	5.36E8
	120.61 - 121.26	-.SDILNAVGITLDETTTR.-	1819.99	2	3.26	0.54	664.2	1	16/32	1.36E9
	80.57	-.TFCIPHGGGGPGM*GPIGVK.-	1856.13	2	2.97	0.53	419.6	1	15/36	7.13E8
	95.35	-.TQTLSQLENSGAFIER.-	1794.94	2	3.50	0.56	989.8	1	17/30	1.14E9
#21	Q8X957 (Q8X957) Acc					110.26			11 (11 0 0 0 0)	0.80
	114.54	-.AAEGIAPKPLDANQM*AALVELLK.-	2380.79	3	3.54	0.54	956.1	1	30/88	1.65E9
	108.73	-.DLVHAIPLYAIK.-	1353.63	2	2.95	0.46	965.7	1	15/22	6.58E8
	77.86	-.EGIEPDQPGVVGPIK.-	1535.72	2	2.83	0.55	335.9	1	15/28	1.20E9
	112.95 - 113.28	-.GFPLAYVGDVVGTGSSR.-	1682.86	2	3.76	0.58	1236.9	1	19/32	1.80E9
	166.78	-.LGTGANVFLASAELAAVAALIGK.-	2158.53	2	3.99	0.59	489.3	1	14/44	1.44E8
	98.33	-.LPTPEEYQTYVAQVDK.-	1882.06	2	3.22	0.25	554.1	1	14/30	1.17E9
	111.20	-.M*DAAQLTEEGYYSVFGK.-	1926.09	2	5.22	0.60	1612.8	1	23/32	1.45E9
	141.52 - 142.19	-.NPPAGEEEFLLDLLTNR.-	1929.12	2	5.16	0.52	821.6	1	19/32	6.33E8
	30.57 - 32.08	-.SPLLTPEK.-	885.04	2	2.58	0.21	836.5	1	12/14	2.69E8
	76.06	-.TDVLIDEVR.-	1060.18	2	2.64	0.20	1415.2	1	15/16	7.69E8
	102.13 - 102.67	-.YLNFNQLSQYTEK.-	1648.80	2	3.98	0.60	1700.5	1	19/24	1.76E9
#22	Q8X8J5 (Q8X8J5) RNa					110.25			11 (11 0 0 0 0)	1.14
	72.84 - 73.49	-.AAPATPATPAQPGLLSR.-	1619.85	2	2.67	0.52	325.3	1	13/32	3.15E9
	59.73 - 60.27	-.ALFSGGEETKPTEQPAPK.-	1888.07	2	4.87	0.58	778.1	1	19/34	7.42E8
	71.34	-.ALNVEEQSVQETEQEER.-	2019.07	2	5.02	0.62	1502.6	1	21/32	1.88E9
	65.44 - 66.53	-.APAPEYVPEAPR.-	1297.44	2	2.61	0.47	480.6	1	13/22	1.96E9
	106.45	-.GGDIEETAFNTNLEAADEIAR.-	2237.32	2	4.96	0.56	1214.6	1	21/40	1.32E9
	76.74	-.LHEEAM*ALPSEEEFAER.-	2005.15	2	4.77	0.61	1069.5	1	21/32	9.73E8
	128.92 - 130.19	-.LIEEEALKENTQEVHAIVPVPIASYLLNEK.-	3391.86	3	3.25	0.40	573.1	1	29/116	2.50E9
	136.06	-.LYTGEIPLFSHYQIESQIESAFQR.-	2858.15	3	3.33	0.41	724.8	1	29/92	3.70E8
	68.43 - 68.64	-.M*LINATQQEELR.-	1462.66	2	3.93	0.46	1174.9	1	17/22	1.17E9
	90.67	-.QDIGEILIDNPK.-	1355.52	2	2.57	0.23	771.9	1	15/22	1.07E9
	76.23 - 76.79	-.VPLPVVAQTAPEQQEENNADNRDNGGM*PR.-	3164.37	3	4.13	0.50	1077.2	1	32/112	1.19E9
#23	Q8XCB3 (Q8XCB3) Va					110.24			11 (11 0 0 0 0)	0.79
	98.63	-.ADVLAKPAVEAVENGDIQFVPK.-	2311.62	2	3.77	0.56	711.2	1	21/42	6.86E8
	110.61	-.GGVVIEPM*LTDQWYVR.-	1880.16	2	2.92	0.45	253.8	1	13/30	1.79E9
	88.67 - 89.21	-.GNESDVYSSEIPAEFQK.-	1900.98	2	3.88	0.23	478.8	2	14/32	1.73E9
	137.11	-.GNVIDPLDM*VDGISLPELLEK.-	2284.61	2	3.10	0.59	508.5	1	15/40	5.09E8
	139.40 - 139.87	-.GNVIDPLDM*VDGISLPELLEK.-	2284.61	2	4.80	0.63	1262.8	1	21/40	5.89E8
	142.39	-.IIDGAELLIPMAGLINKEDELAR.-	2495.92	3	3.88	0.54	966.1	1	33/88	4.35E8
	100.19	-.IPAWYDEAGNVYVGR.-	1710.87	2	3.82	0.63	1354.2	1	21/28	1.38E9
	86.56	-.LESITVLPADDKGPVSVTK.-	1970.25	3	3.42	0.45	793.5	1	29/72	1.22E9
	131.81	-.SKGNVIDPLDM*VDGISLPELLEK.-	2499.86	3	4.10	0.54	1016.1	1	31/88	5.97E8
	111.31	-.TADGKDYLVVATTRPETLLGDTGVAVNPEDPR.-	3372.69	3	4.46	0.47	909.4	1	38/124	1.28E9
	71.04	-.TAISDLEVENR.-	1247.34	2	3.03	0.25	1341.1	1	16/20	1.19E9
#24	CAPP_ECO57 (Q8X74					110.21			11 (11 0 0 0 0)	0.74
	131.19 - 131.85	-.AGNDANRQELLTTLQNLSNDELLPVAR.-	2967.24	3	3.05	0.43	456.3	1	30/104	1.60E9
	138.00	-.AIPWIFAWTQNR.-	1503.73	2	3.15	0.53	718.9	1	17/22	3.70E8
	104.64	-.DAGVM*AASWAQYQAQDALIK.-	2154.39	2	3.79	0.01	423.5	5	13/38	1.30E9
	84.69 - 84.93	-.ELNEQLEENLGYK.-	1579.69	2	3.15	0.55	602.9	1	13/24	1.29E9
	9.57	-.GEAASNPEVIAR.-	1214.31	2	3.54	0.51	1071.3	1	19/22	9.41E8
	70.94 - 71.49	-.GGAPAHAALLSQPPGSLK.-	1672.91	2	2.84	0.10	577.8	1	14/34	1.22E9

	121.20	-.LM*LPAWLGAGTALQK.-	1586.92	2	4.20	0.55	1131.1	1	22/28	9.48E8
	135.94	-.NIYTDPLNVLQAELLHR.-	2010.28	2	3.93	0.54	516.4	1	16/32	4.95E8
	8.92	-.NLQEEDIK.-	989.06	1	1.98	0.06	210.0	7	9/14	8.64E8
	134.60 - 135.51	-.QELLTTLQNLSNDELLPVAR.-	2268.55	2	2.72	0.28	143.4	128	8/38	1.06E9
	80.32	-.SATPEQELGKLPLGSRPAK.-	1980.26	2	2.61	0.38	336.1	3	13/36	5.44E8
#25	METE_ECO57 (Q8X8L					100.24			10 (10 0 0 0 0)	0.54
	72.20 - 72.78	-.AALANM*VQAAQNLR.-	1487.71	2	4.50	0.55	1585.1	1	20/26	1.45E9
	136.35	-.CHELALLCDALNSGDTAALAEWSAPIQAR.-	3155.45	3	3.78	0.56	868.6	1	32/112	5.65E8
	122.07 - 122.63	-.FKLPAWPTTTIGSFPQTTEIR.-	2392.74	3	4.88	0.53	948.4	1	32/80	1.97E9
	9.14	-.LAAITAQDSQR.-	1174.29	2	3.57	0.51	1166.3	1	18/20	1.19E9
	116.48	-.LPAWPTTTIGSFPQTTEIR.-	2117.39	2	2.86	0.42	429.2	1	16/36	1.17E9
	127.84	-.LPSDWLLSAGLINGR.-	1612.86	2	4.31	0.54	2461.2	1	22/28	9.07E8
	162.34 - 162.98	-.LSLLNDILPVYQQVLAELAK.-	2241.66	2	4.35	0.54	768.2	1	23/38	1.43E8
	168.81	-.LSLLNDILPVYQQVLAELAK.-	2241.66	3	4.24	0.47	1007.8	1	28/76	1.13E8
	168.83	-.LSLLNDILPVYQQVLAELAK.-	2241.66	2	4.32	0.53	200.3	1	13/38	1.58E8
	159.50	-.LTWTQLLDEVDEALALGHK.-	2153.42	3	3.45	0.37	1002.0	1	32/72	8.62E7
#26	Q8XE60 (Q8XE60) DN					100.22			10 (10 0 0 0 0)	0.92
	69.26 - 70.05	-.ADVAM*TGEITLR.-	1293.47	2	2.70	0.43	1196.3	1	17/22	1.05E9
	90.77	-.AEYLESPTIDEREQEVLVR.-	2277.47	2	2.82	0.57	304.2	3	13/36	1.07E9
	160.24 - 162.00	-.EASTDEPGVNDLFTVGTVASILQM*LK.-	2753.08	2	3.11	0.59	239.9	1	14/50	4.58E8
	162.32 - 164.07	-.EASTDEPGVNDLFTVGTVASILQM*LK.-	2753.08	3	4.37	0.53	857.5	1	28/100	8.12E8
	163.14 - 163.76	-.EASTDEPGVNDLFTVGTVASILQM*LK.-	2753.08	2	3.80	0.64	423.4	1	17/50	4.67E8
	83.94	-.ILEYLAVQSR.-	1192.39	2	3.56	0.38	2037.2	1	17/18	7.24E8
	108.86 - 109.56	-.KGELTVDDSAIIGIIR.-	1700.96	2	2.82	0.41	181.9	16	12/30	6.79E9
	68.25	-.LSGYTEDEKLNIAK.-	1581.75	2	3.49	0.46	915.3	1	16/26	1.01E9
	124.54	-.NPLFLLDEIDK.-	1317.51	2	4.08	0.52	1222.0	1	17/20	5.24E8
	44.34 - 46.30	-.QLLLDK.-	729.89	1	1.86	0.16	178.7	2	8/10	2.86E8
#27	GYRB_ECOLI (P06982					90.25			9 (9 0 0 0 0)	0.49
	124.27	-.DAIAADQLFTTLM*GDAVEPR.-	2151.38	2	3.02	0.50	473.4	1	16/38	1.11E9
	146.28 - 147.37	-.DAIAADQLFTTLMGDAVEPR.-	2135.38	2	2.58	0.29	481.8	1	16/38	4.14E8
	103.87	-.FDVHTNAEQNLFEPIVR.-	2030.23	2	3.60	0.47	850.1	1	15/32	8.10E8
	97.28	-.GLLEEDAFIER.-	1292.42	2	2.80	0.40	1005.4	1	14/20	7.88E8
	104.21	-.LADCQERDPALSELYLVEGDSAGGSAK.-	2853.04	3	3.82	0.46	1331.1	1	28/104	1.14E9
	139.54 - 140.40	-.M*LSSQEVATLITALGCGIGR.-	2094.41	2	4.95	0.60	1718.5	1	21/38	6.27E8
	79.16	-.QIYEHGVPQAPLAVTGETEK.-	2168.39	2	2.83	0.49	268.9	1	16/38	5.92E8
	63.72	-.RAFIEENALK.-	1191.36	2	2.74	0.29	1018.6	1	15/18	5.82E8
	118.63	-.SAVEQQM*NELLAEYLLENPTDAK.-	2623.88	2	3.71	0.56	786.4	1	18/44	9.38E8
#28	Q8XEC4 (Q8XEC4) Ph					80.28			8 (8 0 0 0 0)	0.64
	110.05 - 111.45	-.AFDFACLPNEGVGLAR.-	1737.93	2	2.78	0.58	549.5	1	16/30	2.27E9
	106.02	-.ALLEFDDQEPQLQNEIR.-	2059.22	2	4.84	0.58	855.8	1	20/32	1.40E9
	128.22 - 128.44	-.DIFSLTNEEVQELAK.-	1736.90	2	5.64	0.57	1635.0	1	20/28	1.90E9
	16.74 - 17.99	-.GVALSAGVQR.-	958.10	2	2.81	0.32	1216.5	1	15/18	1.40E8
	18.58 - 19.88	-.GVALSAGVQR.-	958.10	2	2.75	0.31	1674.0	1	16/18	2.03E8
	112.71	-.IYELLDKTDIDDVTQLAK.-	2094.35	2	4.09	0.59	806.3	1	17/34	1.30E9
	131.68 - 132.29	-.QWIIDTPFQPELENAIR.-	2071.32	2	4.16	0.45	397.7	1	14/32	1.41E9
	97.74	-.SNEYANLVGGERYEPDEENPM*LGFR.-	2904.08	3	3.16	0.29	625.1	2	26/96	5.83E8
#29	Q8X979 (Q8X979) Cyt					80.28			8 (8 0 0 0 0)	0.66
	96.29	-.AYSLLEQLR.-	1093.26	2	2.88	0.25	1227.9	1	14/16	8.92E8
	139.75	-.FAIQIPYALGIIATR.-	1647.99	2	3.51	0.30	947.4	1	20/28	5.14E8
	119.95	-.M*EM*VSFSELVLNPVAQVK.-	2054.42	3	3.74	0.46	2040.9	1	33/68	6.45E8
	119.78	-.M*EM*VSFSELVLNPVAQVK.-	2054.42	2	5.59	0.62	1468.7	1	21/34	1.93E9

	83.26	-.RYTPNVADATEAQIQQATK.-	2106.28	2	4.80	0.62	890.0	1	20/36		5.40E8
	74.82 - 75.43	-.SVDTPVIGLK.-	1029.21	1	1.85	0.46	271.5	2	9/18		1.07E9
	89.42 - 90.01	-.YTPNVADATEAQIQQATK.-	1950.10	2	3.64	0.52	738.6	1	17/34		3.41E9
	73.96	-.YTPNVADATEAQIQQATK.-	1950.10	2	2.95	0.49	780.8	1	18/34		4.34E8
#30	Q8XA84 (Q8XA84) As					80.27			8 (8 0 0 0 0)		0.67
	122.20 - 122.30	-.ALLTNVHGLNLENWQEELAQAK.-	2492.77	3	3.69	0.44	827.5	2	26/84		1.20E9
	128.36	-.EYHLLNPVIVDCTSSQAVADQYADFLR.-	3126.42	3	5.40	0.59	1074.8	1	32/104		8.05E8
	135.43 - 136.41	-.GYGAGNDVTAAGVFADLLR.-	1868.04	2	5.03	0.57	1570.9	1	23/36		1.80E9
	133.62 - 135.04	-.GYGAGNDVTAAGVFADLLR.-	1868.04	2	3.14	0.50	1040.5	1	18/36		2.10E9
	131.87 - 133.05	-.GYGAGNDVTAAGVFADLLR.-	1868.04	2	2.59	0.38	894.1	1	16/36		1.53E9
	82.20	-.LDEGM*SFSEATTLAR.-	1644.79	2	2.66	0.52	943.2	1	17/28		6.38E8
	90.48	-.SM*SYQEAM*ELSYFGAK.-	1875.07	2	3.75	0.63	770.1	1	15/30		8.14E8
	127.17 - 127.88	-.TITPIAQFQIPCLIK.-	1744.11	2	2.66	0.12	525.6	2	14/28		7.52E8
#31	SYFB_ECO57 (Q8XE3					80.25			8 (8 0 0 0 0)		0.66
	85.63	-.ADCLGIIGVAR.-	1145.33	2	3.62	0.34	1926.6	1	18/20		8.18E8
	77.76	-.AEANPALHPGQSAAIYLK.-	1852.08	3	3.04	0.49	542.0	1	26/68		8.91E8
	77.67	-.AEANPALHPGQSAAIYLK.-	1852.08	2	3.72	0.54	618.2	1	18/34		1.24E9
	118.27 - 118.81	-.FDM*EIEEDLVEEVAR.-	1840.99	2	4.58	0.44	1221.7	1	19/28		1.01E9
	83.18 - 84.32	-.FVPDTQAPLGIR.-	1314.52	2	3.07	0.47	570.4	1	17/22		1.29E9
	81.95	-.LDDNTIEISVTPNR.-	1587.71	2	2.79	0.36	1038.2	1	16/26		9.37E8
	82.69 - 83.09	-.TLEEEEIAATVAK.-	1404.55	2	3.68	0.48	1516.6	1	17/24		1.50E9
	114.73 - 115.29	-.VQQM*IHPGVEALLLPSPISVEM*SAM*R.-	2883.40	3	4.92	0.38	856.1	1	32/100		1.83E9
#32	Q8XE30 (Q8XE30) DN					80.22			8 (8 0 0 0 0)		0.51
	102.48	-.AGDDAARPEWLEPEFGVR.-	2016.16	2	3.18	0.42	586.9	1	15/34		1.03E9
	102.42	-.AGDDAARPEWLEPEFGVR.-	2016.16	3	3.80	0.33	1132.9	1	28/68		1.03E9
	59.99	-.GDGAILTATQNGYGKR.-	1622.77	2	3.25	0.52	614.6	1	14/30		5.44E8
	116.21 - 116.80	-.ITAILPVTEFEEGVK.-	1646.91	2	3.60	0.41	355.5	1	16/28		2.29E9
	42.59	-.RVEGISALR.-	1001.17	2	2.51	0.24	1026.2	2	13/16		1.82E8
	56.42 - 57.05	-.TAEDENVVGLQR.-	1331.41	2	3.67	0.41	1198.4	1	16/22		5.26E8
	126.44 - 127.42	-.TALVANPWQLGNVAAM*LER.-	2071.39	2	4.37	0.63	1055.0	1	20/36		1.34E9
	65.87	-.VSEISIVGR.-	960.11	2	3.01	0.36	1211.8	1	15/16		3.79E8
#33	TYPA_ECOL6 (Q9EXN					70.22			7 (7 0 0 0 0)		0.29
	118.36	-.AVAFALFGLQDR.-	1308.51	2	3.97	0.49	1588.0	1	18/22		5.53E8
	82.30	-.INIVDTPGHADFGGEVER.-	1927.06	3	3.30	0.54	641.2	1	25/68		7.71E8
	82.22	-.INIVDTPGHADFGGEVER.-	1927.06	2	4.48	0.63	927.8	1	19/34		7.80E8
	53.27 - 54.39	-.LLQQSGTFDSR.-	1252.36	2	2.81	0.50	1239.7	1	15/20		4.24E8
	143.23 - 144.27	-.M*TLEQALEFIDDDELVEVTPTSIR.-	2782.07	2	3.09	0.51	475.5	1	16/46		5.92E8
	57.60	-.NIAIIAHVDHGK.-	1288.48	2	2.90	0.40	1115.0	1	15/22		5.38E8
	55.92 - 56.50	-.VKPNQQVTIIDSEGK.-	1656.86	2	3.80	0.54	496.0	1	16/28		4.44E8
#34	Q8X996 (Q8X996) Pre					70.20			7 (7 0 0 0 0)		0.41
	102.01 - 102.62	-.FHANEAAIVAQAGYPAAVTIATNM*AGR.-	2733.06	3	3.05	0.43	1531.5	1	36/104		1.61E9
	140.12	-.GEVLENLIPEAFAVVR.-	1757.02	2	2.88	0.50	336.2	1	15/30		4.97E8
	78.55	-.ILAQSIEVYQR.-	1320.52	2	3.47	0.52	1421.4	1	17/20		1.10E9
	77.08	-.IQAIIEDIKER.-	1328.54	2	3.00	0.39	972.8	1	15/20		1.05E9
	153.58 - 154.82	-.NELLDVSDVSETINSIREDVFK.-	2523.74	3	4.09	0.53	455.8	1	26/84		4.24E8
	75.11	-.TPLIISGPAEDSSEM*YKR.-	2011.24	3	3.37	0.40	527.3	1	24/68		6.15E8
	74.95	-.TPLIISGPAEDSSEM*YKR.-	2011.24	2	3.44	0.46	511.0	1	15/34		5.80E8
#35	Q8XDE6 (Q8XDE6) Ar					70.20			7 (7 0 0 0 0)		0.43
	56.82 - 57.38	-.DQEFSSDLGSR.-	1241.25	2	2.62	0.43	1396.4	1	16/20		5.68E8
	115.31	-.FLAFGETHLADVLVSK.-	1748.02	2	2.58	0.56	693.6	1	17/30		7.51E8
	82.56	-.HITYYLDRPDVLAR.-	1732.96	2	2.87	0.45	380.1	2	11/26		8.92E8

	97.12	-.HQQGQPLSLPVHVADAFR.-	2001.24	3	4.01	0.50	2470.9	1	35/68	1.34E9
	72.02	-.TVVTAVSQAVR.-	1131.31	2	3.72	0.50	1551.7	1	17/20	7.60E8
	122.22	-.WDAAQSLLATYIK.-	1480.69	2	3.87	0.50	1492.9	1	18/24	6.64E8
	133.15 - 134.02	-.WFILQATSPAANVLETVR.-	2017.32	2	3.01	0.34	539.0	1	15/34	1.17E9
#36	Q8XBF4 (Q8XBF4) Pu					60.24			6 (6 0 0 0 0)	0.33
	85.65	-.AGVDFEIVNNESDPR.-	1662.74	2	2.56	0.35	610.7	1	15/28	1.01E9
	71.23	-.APECFYIEQK.-	1285.42	2	3.09	0.40	1307.4	1	15/18	6.15E8
	123.68 - 124.25	-.APM*ILALANPEPEILPPLAK.-	2115.57	2	4.61	0.42	791.8	1	19/38	1.76E9
	49.90 - 51.25	-.IQVSPTKPLATQR.-	1439.69	2	3.02	0.50	751.2	1	16/24	3.47E8
	11.78 - 13.29	-.RVVLPEGEEAR.-	1255.41	2	3.06	0.48	784.2	1	15/20	2.17E8
	133.76	-.TLDDVIEGADIFLGCSGPK.-	2008.21	2	4.81	0.63	1313.9	1	20/36	7.81E8
#37	Q8XE26 (Q8XE26) Rib					60.22			6 (6 0 0 0 0)	0.75
	117.80 - 118.60	-.ALDALLDYQDYPIPAAK.-	1878.12	2	2.91	0.40	856.1	1	16/32	6.33E9
	73.20 - 74.37	-.DAPDYQYLAAR.-	1283.37	2	2.94	0.51	481.2	1	16/20	1.18E9
	89.89 - 90.26	-.EQGACPWFNETTYAK.-	1802.92	2	3.16	0.57	346.8	1	13/28	1.76E9
	76.34	-.FIDQSISANTNYDPSR.-	1828.92	2	4.45	0.54	958.3	1	18/30	8.04E8
	140.85	-.TLGIGVINFAYYLAK.-	1643.95	2	3.01	0.56	837.6	1	17/28	3.78E8
	35.25 - 36.51	-.TSDIHETIIK.-	1157.30	2	2.63	0.45	1063.2	1	15/18	3.01E8
#38	Q8X8H1 (Q8X8H1) DN					50.29			5 (5 0 0 0 0)	0.24
	146.22 - 146.81	-.AM*GLPLLAVSGVEADDVIGTLAR.-	2285.65	2	5.73	0.65	1706.3	1	24/44	4.38E8
	119.25 - 120.32	-.ATAAEVFGLPLETVTSEQR.-	2020.23	2	3.15	0.47	296.8	1	14/36	1.21E9
	145.13	-.GIAFDTM*LESYILNSVAGR.-	2074.34	2	5.83	0.54	1618.7	1	21/36	3.71E8
	139.79	-.GPLNVFENIEM*PLVPVLSR.-	2141.52	2	3.13	0.44	163.6	9	10/36	4.54E8
	125.12	-.TAQALLQGLGGLDTLYAEPEK.-	2189.45	2	5.38	0.59	1572.7	1	25/40	9.25E8
#39	THIO_ECOLI (P00274)					50.23			5 (5 0 0 0 0)	0.21
	40.85 - 42.24	-.LNIDQNPGTAPK.-	1268.40	2	3.96	0.37	1736.3	1	19/22	3.19E8
	41.51 - 42.82	-.LNIDQNPGTAPK.-	1268.40	2	3.85	0.37	1494.6	1	18/22	3.17E8
	43.57 - 44.66	-.LNIDQNPGTAPK.-	1268.40	2	3.95	0.36	1381.9	1	18/22	2.93E8
	38.70 - 40.20	-.LNIDQNPGTAPK.-	1268.40	2	3.29	0.35	878.4	1	16/22	2.41E8
	121.15 - 122.65	-.M*IAPILDEIADEYQGK.-	1823.06	2	4.52	0.67	966.3	1	20/30	1.86E9
#40	SYT_ECO57 (Q8XE27					50.22			5 (5 0 0 0 0)	0.27
	65.24	-.ALNAYLQR.-	949.09	2	2.63	0.32	1240.7	1	13/14	4.92E8
	76.56	-.DLGSM*DVNEVIEK.-	1465.61	2	3.16	0.33	1045.3	1	16/24	9.36E8
	65.22	-.GKDLGSM*DVNEVIEK.-	1650.83	2	3.60	0.59	885.9	1	16/28	6.92E8
	26.81 - 28.52	-.LSASYVGEDNER.-	1340.38	2	2.89	0.49	736.4	1	16/22	4.11E8
	116.37	-.M*AIGPVIDNGFYYDVDLDR.-	2190.42	2	4.40	0.51	1339.0	1	22/36	1.32E9
#41	Q8XD55 (Q8XD55) Ac					50.17			5 (5 0 0 0 0)	0.30
	64.16 - 65.02	-.AQNAQVAAGQLGGTPPVK.-	1707.91	2	3.16	0.44	990.1	1	19/34	6.73E8
	125.28	-.FQLTPVDVITAIK.-	1445.73	2	3.21	0.25	612.1	2	17/24	6.23E8
	81.60 - 81.74	-.LATGANALDTAAAIR.-	1429.60	2	2.82	0.37	1344.8	1	19/28	1.08E9
	92.24	-.LPTGVGYDWTGM*SYQER.-	1977.14	2	3.23	0.47	383.0	1	13/32	8.91E8
	93.05	-.TSGVGDVQLFGSQYAM*R.-	1833.02	2	3.32	0.45	1078.0	1	19/32	1.01E9
#42	Q8X3J5 (Q8X3J5) Puta					40.28			4 (4 0 0 0 0)	0.22
	150.17 - 151.12	-.AEAM*GVALSDINQTISTAFGSSYVNDFLNQGR.-	3394.67	3	5.56	0.60	469.1	6	24/124	6.08E8
	27.89 - 29.35	-.LATGANALDTSR.-	1190.29	2	2.50	0.37	776.0	1	15/22	3.77E8
	108.36	-.NELLSLAAQSPNQVTGVRPNGLEDTPM*FK.-	3144.51	3	3.63	0.50	1194.1	1	34/112	1.14E9
	110.36	-.TPTSFLPEEDQGVFM*TTAQLPSGATM*VNTTK.-	3333.69	3	3.43	0.44	351.7	2	29/120	1.08E9
#43	Q8XAN5 (Q8XAN5) RN					40.27			4 (4 0 0 0 0)	0.32
	75.03	-.AATHDVLAGLTAR.-	1296.46	2	3.31	0.65	1537.3	1	19/24	9.89E8
	81.50	-.FGIDM*NTDHTLEEVGK.-	1822.98	2	3.69	0.53	541.3	1	15/30	7.93E8
	133.81 - 134.49	-.GLQFLDLIQEGNIGLM*K.-	1906.24	2	5.47	0.55	2213.2	1	25/32	1.11E9

	91.31 - 92.30	-.LQQIEEETGLTIEQVK.-	1859.07	2	4.52	0.62	2408.6	1	22/30	1.69E9
#44	SYL_ECO57 (Q8XBN8					40.24			4 (4 0 0 0 0)	0.37
	90.71	-.GDVAALNVDALTEDQK.-	1659.78	2	4.26	0.60	1025.3	1	18/30	1.39E9
	128.09	-.NVLQPIGWDAFGLPAEGAAVK.-	2154.45	2	4.77	0.63	645.7	1	19/40	1.15E9
	83.59	-.NYTIGDVIAR.-	1122.26	1	2.02	0.21	257.2	1	12/18	5.39E8
	113.05 - 113.63	-.YGLNIKPVILAADGSEPDLSQQALTEK.-	2872.22	3	4.76	0.50	872.5	1	38/104	2.25E9
#45	Q8XDL9 (Q8XDL9) Pu					40.24			4 (4 0 0 0 0)	0.21
	127.40	-.DYAELLESVADRPDAEM*LQTM*LLR.-	2813.16	3	4.74	0.44	1301.0	1	31/92	1.06E9
	141.06 - 142.49	-.GTSVYFPSQVIPM*LPEVLSNGLCSLNPQVDR.-	3435.89	3	3.56	0.32	572.3	1	32/120	8.53E8
	61.53	-.HLEELHNLYK.-	1296.46	2	2.79	0.60	683.8	1	14/18	6.15E8
	73.05	-.TCIHGDQVLAQPLGADR.-	1852.04	2	2.79	0.44	511.5	1	20/32	5.41E8
#46	Q8X774 (Q8X774) Pyr					40.22			4 (4 0 0 0 0)	0.46
	88.95 - 89.44	-.AQSLDALSSM*ANIAGYR.-	1784.97	2	2.63	0.31	459.6	1	14/32	2.34E9
	120.95 - 121.13	-.LPTQSSQLYGTNLVNLLK.-	1990.29	2	4.50	0.68	934.9	1	18/34	1.54E9
	52.85	-.NVTVM*AM*DSVPR.-	1352.56	2	2.75	0.43	557.1	2	13/22	4.73E8
	128.18 - 129.13	-.VIGYTDLPGRLPTQSSQLYGTNLVNLLK.-	3062.51	3	3.85	0.46	982.0	1	30/108	2.25E9
#47	EFTU_ECOLI (P02990					40.19			4 (4 0 0 0 0)	0.21
	141.54	-.AIDKPFLLPIEDVFSISGR.-	2118.46	3	3.80	0.45	1542.5	1	32/72	4.54E8
	107.57	-.ELLSQYDFPGDDTPIVR.-	1966.14	2	3.90	0.50	521.2	1	16/32	1.08E9
	72.66	-.GITINTSHVEYDTPTR.-	1804.94	2	3.78	0.62	776.2	1	16/30	7.56E8
	142.66 - 142.77	-.ILELAGFLDSYIPEPER.-	1963.22	2	3.54	0.45	636.2	1	17/32	7.85E8
#48	Q8X9H9 (Q8X9H9) PT					30.26			3 (3 0 0 0 0)	0.03
	168.24	-.ALQLPIAVLPVAALLLR.-	1772.25	2	3.79	0.38	644.0	1	17/32	2.22E8
	168.33	-.ALQLPIAVLPVAALLLR.-	1772.25	3	5.26	0.67	1996.0	1	32/64	1.00E8
	158.21 - 158.88	-.AM*VTINPEINM*GVLAGIITGLVGGAAYNR.-	2949.44	3	3.30	0.36	826.6	2	28/112	1.30E8
#49	Q8XEF0 (Q8XEF0) Glu					30.24			3 (3 0 0 0 0)	0.30
	126.00 - 126.70	-.DVPTNEGVLGEIALSSLPR.-	1968.20	2	2.69	0.42	253.0	12	12/36	1.32E9
	121.35 - 122.78	-.FGVTPAYLVNADVIQIK.-	1849.16	2	3.93	0.57	1029.3	1	20/32	1.37E9
	134.08 - 135.35	-.IGGASFEDFQQDLLNLSK.-	1983.17	2	4.77	0.62	1327.3	1	20/34	1.58E9
#50	ODO1_ECOLI (P07015					30.23			3 (3 0 0 0 0)	0.26
	140.76	-.HPLAVSSLEELANGTFLPAIGEIDELDPK.-	3077.43	3	3.65	0.44	814.1	1	29/112	6.28E8
	98.08 - 98.67	-.IVINNQVGFTTSNPLDAR.-	1960.18	2	4.32	0.45	734.7	1	16/34	1.61E9
	109.46	-.M*VQAPIFHVNADDPEAVAFVTR.-	2444.75	3	4.53	0.65	1072.7	1	31/84	1.48E9
#51	Q8XCM5 (Q8XCM5) C					30.22			3 (3 0 0 0 0)	0.12
	123.26	-.LAQTNSEDVFSLAM*TAFAR.-	2089.32	2	4.02	0.70	337.0	1	12/36	6.18E8
	138.04 - 138.76	-.VGVLDIPGFYVGLTDDVK.-	1908.18	2	2.71	0.45	267.7	1	16/34	7.13E8
	137.56	-.YLNLLDYSHNVLLASDVEQFAK.-	2553.85	3	4.41	0.49	1020.7	1	28/84	4.57E8
#52	Q8XC75 (Q8XC75) Z5					30.20			3 (3 0 0 0 0)	0.11
	139.69	-.LISEAFLPGHGFPVGVVNFNYLTADELEK.-	3178.58	3	3.92	0.54	667.3	3	25/112	4.19E8
	141.58 - 142.45	-.LLGEILTELVEQGM*AER.-	1918.20	2	3.44	0.51	448.3	1	13/32	5.79E8
	123.02	-.TFSGQTLSAEDLLPALPTGAVR.-	2245.52	2	2.94	0.40	313.4	1	15/42	5.20E8
#53	GADC_ECO57 (P5822					30.19			3 (3 0 0 0 0)	0.24
	73.01 - 73.65	-.ANTGVTLEPINSQNAPK.-	1754.92	2	3.80	0.51	460.5	1	17/32	2.30E9
	74.58	-.ANTGVTLEPINSQNAPK.-	1754.92	2	3.68	0.48	443.2	1	15/32	8.64E8
	41.19 - 41.92	-.GHFFLHPR.-	1011.16	2	2.92	0.53	703.9	1	12/14	2.69E8
#54	OSTA_ECO57 (Q8XA1					30.19			3 (3 0 0 0 0)	0.22
	52.13	-.FNVDYTK.-	886.97	1	1.90	0.07	548.9	3	9/12	2.34E8
	104.76 - 105.90	-.FSVGYAVQNFNATVSTK.-	1834.02	2	2.65	0.43	1088.9	1	17/32	1.49E9
	114.25 - 114.98	-.VHLEPTINLPLSNNWGSINTEAK.-	2548.84	3	3.75	0.45	1299.7	1	33/88	1.39E9
#55	Q8XE12 (Q8XE12) Ca					30.18			3 (3 0 0 0 0)	0.29
	132.13	-.GPTLLEDFILR.-	1274.49	2	3.63	0.58	1018.2	1	16/20	3.42E8

	77.63 - 78.78	-.LFSYTDTQISR.-	1331.46	2	2.77	0.38	644.2	1	14/20	2.62E9
	122.09	-.LFWLSQTPFEQR.-	1552.76	2	2.97	0.24	601.1	1	13/22	1.16E9
#56	PLSB_ECO57 (P5813(					20.29			2 (2 0 0 0 0)	0.06
	142.15	-.ESIDPIEAVRPAWLTPTVNNIAADLM*VR.-	3109.55	3	5.81	0.51	2024.8	1	38/108	4.74E8
	53.42	-.LYQGINVHNAER.-	1414.55	2	2.74	0.38	977.9	1	15/22	3.60E8
#57	PNTB_ECOLI (P07002					20.27			2 (2 0 0 0 0)	0.16
	102.19	-.NSHSVIITPGYGM*AVAQAQYPVAEITEK.-	2992.35	3	5.47	0.58	1557.4	1	31/108	1.20E9
	101.30	-.SM*NTGYAGVQNPLFFK.-	1791.02	2	3.37	0.56	449.5	1	14/30	1.04E9
#58	MUKB_ECO57 (Q8XD					20.26			2 (2 0 0 0 0)	0.11
	128.20	-.AEAAELEVDELKSQLADYQQALDVQQTR.-	3163.40	3	5.26	0.63	1569.5	1	35/108	9.89E8
	138.93 - 139.46	-.TFDLDELVTTLSGGNGAGK.-	1896.05	2	2.66	0.44	491.7	1	14/36	5.90E8
#59	Q8X765 (Q8X765) Cat					20.25			2 (2 0 0 0 0)	0.17
	102.08 - 102.15	-.FLNDPQAFNEAFAR.-	1640.78	2	4.28	0.45	931.3	1	18/26	1.45E9
	142.79 - 143.56	-.VGVLSNDFFVNLLDM*R.-	1856.14	2	5.08	0.59	1860.1	1	20/30	1.03E9
#60	ATCU_ECO57 (Q8XD2					20.23			2 (2 0 0 0 0)	0.10
	100.78	-.TALVM*GSASPQDLVQAVEK.-	1961.23	2	4.70	0.55	886.6	1	20/36	1.06E9
	54.43 - 55.04	-.TGTLTEGKPQVVAVK.-	1528.78	2	2.86	0.45	844.0	1	16/28	3.10E8
#61	ETK_ECO57 (Q8XC28					20.23			2 (2 0 0 0 0)	0.13
	133.60	-.AVLEQIVNVDNQLNELTFR.-	2216.48	2	4.58	0.59	1652.8	1	24/36	7.81E8
	97.57	-.ILNSIANNYLQQNIAR.-	1846.08	2	3.34	0.54	884.3	1	17/30	1.03E9
#62	Q8X6R2 (Q8X6R2) Ce					20.21			2 (2 0 0 0 0)	0.23
	110.13 - 110.82	-.EQTPEKETEVQNEQTVVEEIVQAQEPVK.-	3240.48	3	4.27	0.38	1202.9	1	30/108	2.31E9
	124.97	-.NNIPVIAQHTGADSASVIFDAIQAAK.-	2652.94	3	3.67	0.56	1247.1	1	34/100	1.04E9
#63	Q8X708 (Q8X708) Mal					20.20			2 (2 0 0 0 0)	0.07
	150.23 - 151.23	-.AYDINLTDLLEEEIDPALGNGGLGR.-	2659.89	2	4.07	0.53	437.3	1	16/48	2.78E8
	134.14	-.LTGNNLLNLGWYQDVQDSLK.-	2292.53	2	4.06	0.46	449.9	1	14/38	6.61E8
#64	FTSH_ECO57 (Q8X9L					20.16			2 (2 0 0 0 0)	0.10
	124.03 - 124.65	-.ALGVTFFLPEGDAISASR.-	1852.08	2	3.27	0.42	440.3	1	16/34	8.07E8
	150.17 - 151.12	-.GTPGFSGADLANLVNEAALFAAR.-	2263.50	2	3.19	0.45	347.5	2	14/44	6.08E8
#65	PARC_ECOLI (P20082					10.25			1 (1 0 0 0 0)	0.03
	146.49 - 147.29	-.ALITLPENAHVM*PPVVIEDASDM*LLAITQAGR.-	3419.96	3	4.90	0.59	685.8	1	29/124	4.08E8
#66	ARCB_ECO57 (P5836					10.24			1 (1 0 0 0 0)	0.01
	161.07	-.VQLDNQPVDFTSFLADLENLSALQAQQK.-	3134.44	3	4.73	0.56	756.8	1	29/108	1.10E8
#67	FTSK_ECO57 (Q8X5H					10.23			1 (1 0 0 0 0)	0.07
	93.67	-.YGEQYQHDVPVNAEDADAAAEAELAR.-	2833.92	3	4.64	0.50	1356.4	1	34/100	9.89E8
#68	Q8X728 (Q8X728) Hyp					10.22			1 (1 0 0 0 0)	0.05
	75.15	-.SIGVGQYQHDVSQTQLAR.-	1988.15	2	4.36	0.65	915.2	1	17/34	6.73E8
#69	CLPA_ECOLI (P15716					10.21			1 (1 0 0 0 0)	0.04
	137.60	-.IVQGDVPEVM*ADCTIYSLDIGSLLAGTK.-	2983.37	3	4.19	0.29	542.5	6	23/108	6.38E8
#70	NUOG_ECO57 (Q8XC					10.21			1 (1 0 0 0 0)	0.06
	125.24	-.EIESYDAVLVLGEDVTQTGAR.-	2266.45	2	4.14	0.54	930.0	1	20/40	8.32E8
#71	Q8X668 (Q8X668) Bet					10.21			1 (1 0 0 0 0)	0.03
	131.43 - 131.57	-.GIIDFLNQYEEAVK.-	1639.83	2	4.11	0.54	895.1	1	17/26	4.19E8
#72	Q8XA93 (Q8XA93) Hy					10.20			1 (1 0 0 0 0)	0.06
	125.26	-.LPALDLAEFNIAGAPGYSK.-	1948.21	2	4.09	0.60	1281.1	1	19/36	8.61E8
#73	PTGB_ECOLI (P05053					10.19			1 (1 0 0 0 0)	0.12
	95.62 - 96.17	-.ATGTSEM*APALVAAFGGK.-	1695.92	2	3.84	0.60	678.2	1	19/34	1.71E9
#74	ENO_ECOLI (P08324)					10.18			1 (1 0 0 0 0)	0.05
	124.15 - 124.85	-.AKGM*NTAVGDEGGYAPNLGSNAEALAVIAEAVK.-	3206.53	3	3.65	0.43	471.7	13	26/128	7.17E8
#75	Q8XAV7 (Q8XAV7) Pu					10.18			1 (1 0 0 0 0)	0.11
	130.07 - 131.30	-.NLDREPIGLM*DTVATVTPAQLLQFYQR.-	3107.53	3	3.59	0.30	712.2	2	27/104	1.56E9

#76	Q8X903 (Q8X903) Pep					10.18			1 (1 0 0 0 0)	0.02
	150.97	-.SGFPDLLVDTLLATEDR.-	1863.06	2	3.55	0.63	1009.7	1	17/32	2.47E8
#77	Q8X7C5 (Q8X7C5) DN					10.18			1 (1 0 0 0 0)	0.06
	128.46 - 128.48	-.AVLDHFFSDFTQQLDK.-	1912.09	2	3.52	0.53	682.6	1	15/30	9.05E8
#78	Q8XEG0 (Q8XEG0) Pr					10.18			1 (1 0 0 0 0)	0.06
	124.73	-.IADTSLPLDELVADPVTAVEK.-	2197.47	2	3.50	0.67	341.9	1	14/40	8.49E8
#79	Q8X9L3 (Q8X9L3) Hyp					10.17			1 (1 0 0 0 0)	0.04
	78.92	-.LATPAGQQVAQSLQQTGDTR.-	2071.24	2	3.40	0.58	326.0	1	13/38	5.89E8
#80	RvrsDB|00002799					10.16			1 (1 0 0 0 0)	0.60
	96.89 - 97.86	-.LDDM*ALNNC#VLSYDIPRK.-	2211.45	3	3.19	0.16	1224.1	2	26/68	8.66E9
#81	Q8XBP5 (Q8XBP5) Pe					10.16			1 (1 0 0 0 0)	0.12
	77.34 - 78.68	-.LVSNEFVAM*M*DLQK.-	1657.93	2	3.14	0.30	352.6	5	13/26	1.71E9
#82	UVRB_ECOLI (P07025					10.15			1 (1 0 0 0 0)	0.11
	134.58 - 135.57	-.LSLFDPLTGQIVSTIPR.-	1858.17	2	3.05	0.54	842.4	1	16/32	1.57E9
#83	RvrsDB|00004199					10.15			1 (1 0 0 0 0)	0.21
	97.82 - 98.43	-.TSQEDTAEQRSIVESFLQR.-	2225.36	2	2.93	0.12	297.7	9	12/36	2.97E9
#84	DEAD_ECO57 (Q8XA8					10.15			1 (1 0 0 0 0)	0.05
	71.98	-.VQQQLESSDLDQYR.-	1709.80	2	2.92	0.34	878.2	1	16/26	7.40E8
#85	OMPA_ECOLI (P0293					10.14			1 (1 0 0 0 0)	0.13
	110.77	-.LGYPITDDLDIYTR.-	1655.83	2	2.83	0.52	641.5	1	14/26	1.81E9
#86	CYOB_ECOLI (P1840					10.14			1 (1 0 0 0 0)	0.03
	52.77 - 53.65	-.LENQHFDEITK.-	1374.48	2	2.82	0.38	677.4	2	13/20	4.25E8
#87	Q8XE40 (Q8XE40) Put					10.14			1 (1 0 0 0 0)	0.02
	135.82	-.ADPLAFWLSDQLAR.-	1603.80	2	2.74	0.50	796.1	1	15/26	3.54E8
#88	GLGB_ECO57 (Q8X6X					10.13			1 (1 0 0 0 0)	0.03
	144.48	-.EVSNFLVGNALYWIER.-	1911.15	2	2.66	0.46	306.4	1	12/30	4.22E8
#89	RS3_ECOLI (P02352)					10.13			1 (1 0 0 0 0)	0.08
	85.18 - 85.69	-.LVADSITSQLER.-	1332.49	2	2.66	0.47	921.3	1	16/22	1.11E9
#90	Q8X7W7 (Q8X7W7) P					10.13			1 (1 0 0 0 0)	0.07
	89.62	-.RGTLDVNQVIGDTTAVR.-	1816.01	2	2.52	0.35	438.9	1	14/32	1.01E9
#91	Q8X6U9 (Q8X6U9) Ga					10.11			1 (1 0 0 0 0)	0.07
	69.13	-.EDLAAYK.-	809.89	1	2.16	0.07	647.8	4	10/12	1.03E9
#92	RvrsDB|00003401					10.10			1 (1 0 0 0 0)	0.07
	63.33	-.AITLLNLK.-	886.11	1	2.03	0.02	623.9	2	10/14	1.07E9
#93	RvrsDB|00002450					10.10			1 (1 0 0 0 0)	0.03
	75.69	-.DLLNAGINGSM*.-	1121.25	1	1.95	0.05	246.5	4	10/20	4.94E8
#94	NARX_ECOLI (P10956					10.09			1 (1 0 0 0 0)	0.03
	10.94	-.EALSNALK.-	845.96	1	1.87	0.00	255.2	7	9/14	4.86E8
#95	Q8X639 (Q8X639) Put					10.09			1 (1 0 0 0 0)	0.04
	127.82	-.DNQLVNAGDLLLTIDK.-	1742.95	1	1.84	0.04	263.2	8	12/30	5.79E8
