# Supplementary material for: Proteins other than the locus of enterocyte effacement-encoded proteins contribute to Escherichia coli O157:H7 adherence to bovine rectoanal junction stratified squamous epithelial cells
Source: BMC Microbiol. 2012 Jun 12;12:103. doi: 10.1186/1471-2180-12-103 (PMC3420319; doi:10.1186/1471-2180-12-103)
Supplement: Additional file 6 — http://www.biomedcentral.com/imedia/1707955235675419/supp6.pdf. DATA SHEETS: O157-DMEM MS/MS data sheet 2. [file 1471-2180-12-103-S6.pdf]

DMEM-02 SequestReport

	Reference<br>Time(s)	Sequence	MH+	Charge	XC	Score<br>Delta Cn	Accession<br>Sp	RSp	Peptides (Hits)<br>Ions	Count	Area<br>Peak Area
#1	CH60_ECOLI (P06139					630.30			63 (63 0 0 0 0)		12.46
	204.81 - 205.51	-.AAVEEGVVAGGGVALIR.-	1568.80	2	3.16	0.52	1344.2	1	19/32		1.23E8
	73.38 - 74.18	-.AIAQVGTISANSDETVGK.-	1761.91	2	4.09	0.59	807.7	1	20/34		4.44E9
	71.49 - 72.74	-.AIAQVGTISANSDETVGK.-	1761.91	2	5.27	0.58	924.9	1	21/34		1.40E10
	70.55	-.AIAQVGTISANSDETVGK.-	1761.91	1	3.51	0.54	466.7	1	16/34		2.44E9
	70.22 - 72.43	-.AIAQVGTISANSDETVGK.-	1761.91	1	3.84	0.51	408.1	1	16/34		4.43E9
	69.93 - 70.89	-.AIAQVGTISANSDETVGK.-	1761.91	2	4.82	0.64	1043.1	1	22/34		1.25E10
	67.69	-.AIAQVGTISANSDETVGK.-	1761.91	2	3.38	0.57	680.6	1	17/34		1.33E9
	137.95 - 139.17	-.AM*LQDIATLTGGTVISEEIGM*ELEK.-	2683.05	3	6.00	0.61	1189.9	1	32/96		5.75E9
	137.64 - 138.89	-.AM*LQDIATLTGGTVISEEIGM*ELEK.-	2683.05	2	2.92	0.33	367.7	1	14/48		4.16E9
	135.60 - 136.77	-.ANDAAGDGTTTATVLAQAIITEGLK.-	2403.63	3	5.37	0.55	2007.4	1	37/96		9.86E9
	137.34 - 138.50	-.ANDAAGDGTTTATVLAQAIITEGLK.-	2403.63	3	6.04	0.54	2751.7	1	41/96		7.18E9
	43.08 - 44.69	-.ATLEDLGQAK.-	1046.16	2	2.76	0.45	1203.7	1	16/18		1.79E9
	41.38 - 42.43	-.ATLEDLGQAK.-	1046.16	2	2.96	0.34	1319.1	1	17/18		1.70E9
	35.15 - 36.76	-.ATLEDLGQAK.-	1046.16	2	2.74	0.36	1197.5	1	16/18		5.55E8
	39.52 - 44.88	-.ATLEDLGQAK.-	1046.16	1	2.07	0.32	281.5	1	10/18		9.46E8
	45.33 - 45.87	-.ATLEDLGQAK.-	1046.16	2	2.57	0.40	1177.7	2	16/18		7.86E8
	37.32 - 39.00	-.ATLEDLGQAK.-	1046.16	2	3.04	0.41	1247.2	1	16/18		1.11E9
	39.51 - 40.84	-.ATLEDLGQAK.-	1046.16	2	2.99	0.44	1196.3	1	16/18		1.37E9
	11.13	-.AVAAGM*NPM*DLKR.-	1406.66	2	2.57	0.52	307.3	1	14/24		3.18E9
	61.19 - 62.97	-.AVTAAVEELK.-	1031.19	1	1.95	0.43	487.5	1	10/18		6.05E9
	61.21 - 62.40	-.AVTAAVEELK.-	1031.19	2	2.80	0.55	785.3	1	15/18		5.16E9
	61.48	-.AVTAAVEELK.-	1031.19	1	1.90	0.36	393.3	2	10/18		3.77E9
	204.03 - 205.34	-.DTTTIIDGVGEEAAIQGR.-	1846.97	2	3.38	0.39	927.8	1	17/34		2.62E8
	85.06 - 86.31	-.DTTTIIDGVGEEAAIQGR.-	1846.97	2	4.54	0.26	629.8	2	18/34		5.81E9
	95.91 - 97.11	-.DTTTIIDGVGEEAAIQGR.-	1846.97	2	4.57	0.50	1521.7	1	22/34		9.90E9
	96.28	-.DTTTIIDGVGEEAAIQGR.-	1846.97	3	4.11	0.57	2540.1	1	34/68		1.68E9
	90.96 - 92.33	-.DTTTIIDGVGEEAAIQGR.-	1846.97	2	3.31	0.42	829.4	1	18/34		2.31E9
	123.46 - 124.62	-.EGVITVEDGTGLQDELDVVEGM*QFDR.-	2869.07	3	5.89	0.62	2464.1	1	42/100		5.74E9
	123.67 - 124.92	-.EGVITVEDGTGLQDELDVVEGM*QFDR.-	2869.07	2	4.24	0.60	750.3	1	17/50		7.32E9
	125.61	-.EGVITVEDGTGLQDELDVVEGM*QFDR.-	2869.07	2	2.85	0.51	311.2	1	13/50		3.63E9
	78.12 - 79.36	-.EIELEDKFENM*GAQM*VK.-	2044.29	2	3.01	0.38	509.4	1	15/32		6.01E9
	80.21 - 81.52	-.EIELEDKFENM*GAQM*VK.-	2044.29	2	2.86	0.46	527.6	1	14/32		6.29E9
	90.50 - 91.17	-.EIELEDKFENM*GAQMVK.-	2028.29	2	3.24	0.25	528.4	2	15/32		3.67E9
	90.48 - 91.35	-.EIELEDKFENMGAQM*VK.-	2028.29	3	4.54	0.17	1457.7	1	29/64		2.60E9
	105.48 - 106.10	-.EIELEDKFENMGAQMVK.-	2012.29	2	3.76	0.50	597.0	1	16/32		2.93E9
	92.47 - 93.60	-.EM*LPVLEAVAK.-	1216.47	2	2.55	0.33	781.0	1	16/20		4.04E9
	106.21 - 107.49	-.GGDGNYGYNAATEEYGNM*IDM*GILDPTK.-	3000.18	3	5.27	0.57	1019.4	1	31/108		4.88E9
	99.89	-.GGDGNYGYNAATEEYGNM*IDM*GILDPTK.-	3000.18	2	3.76	0.58	727.8	1	18/54		1.77E9
	104.64 - 105.71	-.GGDGNYGYNAATEEYGNM*IDM*GILDPTK.-	3000.18	2	4.03	0.58	429.6	1	15/54		5.33E9
	104.45 - 105.67	-.GGDGNYGYNAATEEYGNM*IDM*GILDPTK.-	3000.18	3	5.58	0.58	1075.5	1	29/108		5.82E9
	106.70 - 107.40	-.GGDGNYGYNAATEEYGNM*IDM*GILDPTK.-	3000.18	2	3.55	0.47	273.8	1	13/54		5.35E9
	118.27	-.GGDGNYGYNAATEEYGNMIDM*GILDPTK.-	2984.18	2	3.35	0.22	164.1	10	10/54		2.09E9
	11.24	-.GQNEDQNVGIK.-	1202.26	2	2.95	0.39	666.6	1	14/20		3.07E9
	74.61 - 75.19	-.GVNVLADAVK.-	986.15	2	3.24	0.32	887.7	1	15/18		2.79E9
	74.49 - 76.37	-.GVNVLADAVK.-	986.15	1	1.99	0.43	487.7	1	12/18		1.12E10
	139.90 - 140.43	-.GYLSPYFINKPETGAVELESPFILLADK.-	3113.55	3	5.69	0.64	1707.7	1	37/108		1.56E10
	140.13	-.GYLSPYFINKPETGAVELESPFILLADK.-	3113.55	2	2.91	0.53	209.6	1	15/54		3.17E9
	132.49 - 133.84	-.GYLSPYFINKPETGAVELESPFILLADKK.-	3241.72	3	5.01	0.48	1155.9	1	34/112		2.26E10
	131.98 - 132.65	-.GYLSPYFINKPETGAVELESPFILLADKK.-	3241.72	2	4.42	0.57	427.1	1	16/56		3.13E9
	134.92 - 135.73	-.GYLSPYFINKPETGAVELESPFILLADKK.-	3241.72	3	4.53	0.50	632.4	1	27/112		2.69E9
	55.71 - 57.00	-.LADLRGQNEDQNVGIK.-	1770.93	2	4.06	0.50	692.5	1	17/30		1.12E9
	57.24 - 57.80	-.LADLRGQNEDQNVGIK.-	1770.93	2	3.60	0.57	678.9	1	15/30		9.43E8
	32.10 - 44.36	-.LAGGVAVIK.-	828.04	1	1.81	0.12	385.3	9	9/16		3.06E9

	32.50 - 33.76	-.LAGGVAVIK.-	828.04	2	3.11	0.29	551.9	1	15/16	6.37E8
	34.39 - 35.54	-.LAGGVAVIK.-	828.04	2	3.11	0.40	549.1	1	15/16	6.43E8
	36.08 - 37.51	-.LAGGVAVIK.-	828.04	2	3.00	0.26	576.6	1	15/16	3.54E8
	12.30	-.LIAEAMDK.-	891.07	1	1.88	0.26	1008.8	1	11/14	1.96E9
	11.32	-.NVVLDK.-	687.81	1	1.98	0.09	480.1	1	8/10	5.50E9
	84.81 - 85.41	-.QIVLNCGEEPSVVANTVK.-	1958.20	2	4.44	0.66	1604.6	1	22/34	2.74E9
	89.44 - 90.85	-.QIVLNCGEEPSVVANTVK.-	1958.20	2	5.09	0.68	1869.3	1	25/34	1.63E10
	91.45 - 91.98	-.QIVLNCGEEPSVVANTVK.-	1958.20	2	5.22	0.69	2087.4	1	25/34	6.17E9
	50.37 - 51.78	-.SFGAPTITK.-	922.06	1	1.86	0.13	674.8	1	12/16	1.37E9
	74.28 - 75.69	-.SFGAPTITKDGVSVAR.-	1606.81	2	3.61	0.63	946.3	1	18/30	3.01E9
#2	RS1_ECOLI (P02349)					520.31			52 (52 0 0 0 0)	9.67
	99.50 - 100.29	-.AFLPGSLVDVRPVR.-	1526.81	2	2.97	0.43	316.8	1	14/26	6.92E9
	92.75 - 94.11	-.AKDEADEKDAIATVNKQEDANFSNNAMAEA	3487.71	3	3.30	0.26	187.2	166	22/124	4.23E9
	80.81 - 81.33	-.ANPWQQFAETHNK.-	1571.68	2	3.49	0.48	939.8	1	17/24	4.03E9
	94.71	-.AVIESENSAERDQLLENLQEGM*EVK.-	2849.08	2	2.83	0.45	181.7	2	13/48	3.78E9
	94.63	-.AVIESENSAERDQLLENLQEGM*EVK.-	2849.08	3	5.62	0.56	1072.1	1	33/96	7.94E9
	109.89 - 110.61	-.AVIESENSAERDQLLENLQEGMEVK.-	2833.08	3	5.67	0.56	1326.6	1	33/96	4.74E9
	113.14	-.AVIESENSAERDQLLENLQEGMEVK.-	2833.08	2	2.98	0.47	279.8	1	15/48	1.51E9
	76.25 - 79.07	-.AYEDAETVTGVINGK.-	1567.68	1	2.24	0.40	758.1	1	16/28	4.05E9
	77.06 - 78.43	-.AYEDAETVTGVINGK.-	1567.68	2	3.68	0.63	1737.8	1	20/28	7.22E9
	84.93	-.AYEDAETVTGVINGK.-	1567.68	1	2.20	0.51	609.4	1	15/28	1.52E9
	86.95 - 87.95	-.DQLLENLQEGM*EVK.-	1662.84	2	3.15	0.46	1315.3	1	18/26	3.31E9
	88.52 - 89.29	-.DQLLENLQEGM*EVK.-	1662.84	2	3.39	0.50	1038.2	1	17/26	2.96E9
	102.42	-.DRVEDATLVLSVGDEVEAK.-	2046.22	2	4.33	0.68	1086.9	1	19/36	1.64E9
	204.74	-.DRVEDATLVLSVGDEVEAK.-	2046.22	2	3.59	0.33	466.1	1	14/36	1.09E8
	81.96 - 83.31	-.DTLHLEGKELEFK.-	1559.75	2	2.71	0.32	904.5	1	15/24	2.82E9
	11.02	-.GAIVTGK.-	645.77	1	1.87	0.36	537.2	1	8/12	2.48E9
	104.51 - 105.65	-.GATVELADGVEGYLR.-	1550.70	2	4.45	0.58	2107.9	1	20/28	5.07E9
	92.72 - 93.25	-.GGFTVELNGIR.-	1163.31	2	3.06	0.35	1046.1	1	16/20	4.44E9
	112.80 - 112.99	-.GVVVAIDKDVVLVDAGLK.-	1811.16	2	4.97	0.62	1060.8	1	24/34	5.42E9
	110.86 - 112.19	-.GVVVAIDKDVVLVDAGLK.-	1811.16	2	6.11	0.63	2166.4	1	25/34	5.73E9
	114.63 - 115.11	-.GVVVAIDKDVVLVDAGLK.-	1811.16	2	2.71	0.11	332.0	4	13/34	3.88E9
	111.89	-.GVVVAIDKDVVLVDAGLK.-	1811.16	3	4.60	0.49	2056.5	1	34/68	2.00E9
	120.95 - 121.83	-.GVVVAIDKDVVLVDAGLKSESAIPAEQFK.-	2999.45	3	5.77	0.60	1180.8	1	36/112	4.96E9
	72.98	-.HEAWITLEK.-	1127.28	1	1.92	0.28	569.4	1	11/16	2.01E9
	72.90 - 73.49	-.HEAWITLEK.-	1127.28	2	3.20	0.63	885.7	1	14/16	2.33E9
	81.24 - 81.80	-.HPSEIVNVGDEITVK.-	1637.82	2	3.97	0.55	565.7	1	17/28	3.48E9
	99.03 - 99.22	-.KGDEIAAVVLQVDAER.-	1713.91	2	4.84	0.57	1769.1	1	22/30	2.81E9
	92.98 - 94.40	-.KGDEIAAVVLQVDAERER.-	1999.22	3	3.91	0.55	1174.7	1	33/68	5.96E9
	93.94 - 94.52	-.KGDEIAAVVLQVDAERER.-	1999.22	2	2.84	0.25	398.3	2	14/34	3.63E9
	128.94 - 129.52	-.M*TESFAQLFEESLK.-	1676.87	2	3.81	0.55	1618.1	1	19/26	5.48E9
	148.57	-.M*TESFAQLFEESLKEIETR.-	2305.55	3	3.73	0.51	1308.0	1	32/72	1.89E9
	144.33 - 144.92	-.M*TESFAQLFEESLKEIETRPGSIVR.-	2915.27	3	3.96	0.46	794.9	1	28/96	1.09E10
	148.45 - 149.63	-.NLTDYGAFVDLGGVDGLLHITDM*AWK.-	2839.17	3	3.72	0.57	935.2	1	33/100	1.03E10
	148.59 - 149.18	-.NLTDYGAFVDLGGVDGLLHITDM*AWK.-	2839.17	2	2.74	0.36	252.4	1	13/50	4.19E9
	69.77	-.QEDANFSNNAM*AEAFK.-	1803.89	2	3.22	0.48	425.1	1	14/30	1.32E9
	111.01	-.QLAEDPFNNWVALNK.-	1759.94	2	2.52	0.43	519.4	1	15/28	2.51E9
	114.82 - 115.13	-.QLAEDPFNNWVALNK.-	1759.94	2	4.57	0.55	1032.7	1	21/28	4.33E9
	105.29	-.QLAEDPFNNWVALNKK.-	1888.12	2	2.70	0.43	297.3	1	13/30	2.95E9
	98.45 - 99.56	-.QLGEDPWVAIAK.-	1327.51	2	2.52	0.58	767.3	1	17/22	7.04E9
	96.89 - 97.87	-.QLGEDPWVAIAK.-	1327.51	2	3.16	0.30	597.4	1	16/22	7.18E9
	92.22 - 93.42	-.RAVIESENSAERDQLLENLQEGM*EVK.-	3005.27	3	3.35	0.40	534.1	7	28/100	5.16E9
	90.36	-.RAVIESENSAERDQLLENLQEGM*EVK.-	3005.27	3	3.08	0.42	290.2	8	24/100	4.39E9
	104.27	-.RAVIESENSAERDQLLENLQEGMEVK.-	2989.27	3	4.51	0.41	687.1	2	26/100	3.22E9
	62.85 - 64.08	-.RHEAWITLEK.-	1283.46	2	3.04	0.41	1485.8	1	16/18	1.67E9
	61.15	-.SESAIPAEQFK.-	1207.32	1	1.99	0.30	238.8	1	10/20	1.97E9
	60.84 - 62.03	-.SESAIPAEQFK.-	1207.32	2	3.28	0.50	512.1	1	16/20	2.42E9

	77.00 - 77.64	-.VKHPSEIVNVGDEITVK.-	1865.12	3	3.86	0.41	1125.3	1	32/64	2.95E9
	75.77 - 76.96	-.VKHPSEIVNVGDEITVK.-	1865.12	2	2.80	0.13	375.0	2	14/32	6.19E9
	77.50 - 78.14	-.VKHPSEIVNVGDEITVK.-	1865.12	2	3.90	0.46	892.2	1	20/32	5.48E9
	124.68 - 125.78	-.VVNVGDVVEVM*VLDIDEER.-	2146.41	2	5.67	0.62	1744.9	1	23/36	1.07E10
	126.37 - 127.56	-.VVNVGDVVEVM*VLDIDEER.-	2146.41	2	4.62	0.58	1007.0	1	19/36	5.48E9
	128.89 - 129.93	-.VVNVGDVVEVM*VLDIDEER.-	2146.41	2	4.68	0.65	600.6	1	15/36	4.92E9
#3	Q8XBL3 (Q8XBL3) PE					350.29			35 (35 0 0 0 0)	6.13
	93.71	-.ALLLKEDEIVIDR.-	1527.79	3	4.48	0.48	1214.2	1	25/48	1.18E9
	93.62	-.ALLLKEDEIVIDR.-	1527.79	2	4.21	0.58	1404.9	1	19/24	3.27E9
	85.16	-.ALLLKEDEIVIDRK.-	1655.96	2	3.75	0.60	1430.5	1	18/26	1.70E9
	86.89 - 87.53	-.DALPTEEEQFAAYK.-	1612.72	2	2.91	0.54	755.4	1	20/26	4.57E9
	87.01	-.DALPTEEEQFAAYK.-	1612.72	1	2.06	0.41	270.1	2	11/26	1.29E9
	117.61	-.DKHM*TADAAAHEVIEGQASALEELDDEYLK	3317.54	3	4.96	0.62	612.2	4	26/116	2.07E9
	64.48	-.DVEGAERNGAEGVGLYR.-	1792.89	2	3.44	0.52	445.9	1	15/32	3.39E9
	20.78 - 28.16	-.EIEIYK.-	794.92	1	2.07	0.22	230.0	1	8/10	5.55E8
	63.35 - 64.37	-.EIEIYKQELRDEGK.-	1750.93	2	2.83	0.36	306.7	1	11/26	2.43E9
	64.97 - 65.07	-.EIEIYKQELRDEGK.-	1750.93	2	3.39	0.56	618.2	1	15/26	1.58E9
	115.53 - 116.66	-.IM*FPM*IISVEEVR.-	1596.94	2	3.20	0.37	940.4	1	18/24	1.11E10
	117.23	-.IM*FPM*IISVEEVR.-	1596.94	2	3.83	0.53	836.8	1	16/24	8.62E9
	126.10	-.IM*FPMIISVEEVR.-	1580.94	2	3.21	0.42	1301.5	1	19/24	2.20E9
	64.06 - 64.54	-.ISADQVDQEVER.-	1389.45	1	2.30	0.22	515.7	1	13/22	1.61E9
	78.39	-.KVLGFITDAGGR.-	1234.43	2	3.08	0.46	1668.4	1	18/22	1.79E9
	104.79 - 105.79	-.LKDLPAITLDGHQVEVCANIGTVR.-	2620.98	3	5.00	0.61	1128.1	1	35/92	3.10E9
	105.35 - 105.86	-.M*ISGILASPGIAFGK.-	1478.78	2	5.13	0.56	1460.8	1	20/28	6.55E9
	120.61 - 120.67	-.NDDYLILDAVNNQVYVNPTNEVIDK.-	2880.11	2	5.75	0.63	1155.1	1	21/48	3.20E9
	123.12 - 123.21	-.NDDYLILDAVNNQVYVNPTNEVIDK.-	2880.11	2	4.04	0.57	1131.4	1	22/48	3.53E9
	67.92 - 68.48	-.NILGLK.-	657.83	1	1.91	0.00	308.9	1	7/10	2.47E9
	106.98 - 108.34	-.SLELPAIVGTGSVTSQVK.-	1787.05	2	4.75	0.65	1241.5	1	22/34	6.87E9
	105.56 - 106.35	-.SLELPAIVGTGSVTSQVK.-	1787.05	2	2.52	0.34	371.0	4	13/34	7.67E9
	111.85	-.TEFLFM*DRDALPTEEEQFAAYK.-	2668.92	3	5.61	0.55	1063.3	1	31/84	6.83E9
	111.66 - 111.73	-.TEFLFM*DRDALPTEEEQFAAYK.-	2668.92	2	3.32	0.46	319.9	1	15/42	2.63E9
	119.41	-.TEFLFMDRDALPTEEEQFAAYK.-	2652.92	3	3.30	0.35	582.0	7	24/84	3.55E9
	119.66	-.TEFLFMDRDALPTEEEQFAAYK.-	2652.92	2	2.83	0.32	254.4	2	12/42	1.48E9
	83.14 - 83.66	-.TM*DIGGDKELPYM*NFPK.-	1989.26	3	3.64	0.47	579.0	4	21/64	2.93E9
	83.10 - 84.24	-.TM*DIGGDKELPYM*NFPK.-	1989.26	2	3.03	0.50	264.5	1	13/32	3.43E9
	112.08 - 112.65	-.VLAEQALAQPTTDELM*TLVNK.-	2302.63	2	5.69	0.57	1003.5	1	24/40	1.16E10
	111.98 - 112.53	-.VLAEQALAQPTTDELM*TLVNK.-	2302.63	3	3.60	0.34	1803.1	1	33/80	8.68E9
	122.97	-.VLAEQALAQPTTDELMTLVNK.-	2286.63	2	2.67	0.31	286.9	1	15/40	6.72E9
	121.12 - 122.39	-.VLAEQALAQPTTDELMTLVNK.-	2286.63	2	3.56	0.38	373.1	1	16/40	6.50E9
	85.20 - 86.39	-.VLGFITDAGGR.-	1106.26	2	3.25	0.45	1196.9	1	17/20	2.92E9
	86.91	-.VLGFITDAGGR.-	1106.26	2	3.27	0.51	1534.2	1	19/20	2.79E9
	31.83 - 33.26	-.WTGM*CGELAGDER.-	1498.59	2	3.22	0.51	1256.2	1	17/24	8.99E8
#4	Q9LAP1 (Q9LAP1) Iha					340.37			34 (34 0 0 0 0)	5.24
	101.86 - 102.54	-.ASEQDVLWFDM*DTTR.-	1830.95	2	3.98	0.53	523.5	1	13/28	4.05E9
	73.18	-.DGQLGSLTGGYDR.-	1339.39	2	3.13	0.58	816.9	1	16/24	2.69E9
	73.16 - 73.70	-.DGQLGSLTGGYDR.-	1339.39	2	2.86	0.52	660.5	2	14/24	2.20E9
	79.65 - 80.79	-.DGVVLASTGETFR.-	1352.48	2	3.35	0.59	1754.0	1	18/24	3.64E9
	73.81 - 73.91	-.DYSDVSLYSAGK.-	1305.37	1	2.15	0.42	712.3	1	15/22	2.46E9
	73.95	-.DYSDVSLYSAGK.-	1305.37	2	3.30	0.65	1453.0	1	19/22	1.62E9
	145.65 - 146.47	-.ESNLILNSLLLTPLGESHLVTVGGEFQSSSM'	3418.86	3	4.66	0.54	1030.6	1	34/124	6.85E9
	135.54	-.ESNLILNSLLLTPLGESHLVTVGGEFQSSSM'	3418.86	3	3.31	0.47	583.5	1	32/124	1.83E9
	143.96 - 145.04	-.ESNLILNSLLLTPLGESHLVTVGGEFQSSSM'	3418.86	3	5.08	0.60	1440.7	1	41/124	6.32E9
	69.03 - 69.61	-.FTQNYSSLSAVQK.-	1473.61	2	3.47	0.27	2079.1	1	20/24	9.03E9
	69.19 - 69.81	-.FTQNYSSLSAVQK.-	1473.61	1	2.12	0.28	381.5	2	13/24	2.02E9
	115.34 - 116.16	-.GM*PASYTLILIDGVR.-	1622.91	2	3.22	0.37	991.1	1	17/28	4.57E9
	99.94 - 100.69	-.GPM*STLYGSDAM*GGVVNIITR.-	2172.47	2	5.14	0.56	1078.0	1	22/40	4.09E9
	109.59 - 110.20	-.GPMSTLYGSDAM*GGVVNIITR.-	2156.47	2	3.47	0.46	733.8	1	18/40	2.46E9

	80.05	-.IPYPTESQNYNLGAR.-	1723.87	1	1.85	0.27	116.1	7	10/28	1.58E9
	79.71 - 80.93	-.IPYPTESQNYNLGAR.-	1723.87	2	4.20	0.55	934.8	1	20/28	9.26E9
	99.39 - 100.71	-.ISAGYDHTFTFGTWK.-	1731.89	2	3.57	0.57	798.6	1	15/28	2.95E9
	79.13 - 80.37	-.IVSYSINDNTNSYVNSGK.-	1976.09	2	5.14	0.68	872.6	1	21/34	4.67E9
	77.31 - 78.54	-.IVSYSINDNTNSYVNSGK.-	1976.09	2	5.66	0.66	1266.9	1	22/34	4.85E9
	74.63 - 76.60	-.IVSYSINDNTNSYVNSGK.-	1976.09	2	3.35	0.58	313.2	2	13/34	3.88E9
	112.71 - 113.29	-.KLTNAAASVSVISQEELQSSQYHDLAEALR.-	3260.56	3	7.47	0.66	2389.0	1	39/116	4.76E9
	116.22	-.KLTNAAASVSVISQEELQSSQYHDLAEALR.-	3260.56	3	6.35	0.61	766.5	1	32/116	2.61E9
	148.35 - 149.48	-.LNWQITEEVASWLGAR.-	1874.09	2	4.99	0.52	1749.9	1	21/30	3.30E9
	117.71 - 118.65	-.LTNAAASVSVISQEELQSSQYHDLAEALR.-	3132.39	3	5.74	0.52	672.5	1	29/112	3.82E9
	119.13 - 120.44	-.M*TDALTLNAAVNNLLNK.-	1833.10	2	5.12	0.63	1792.5	1	23/32	4.62E9
	130.78	-.M*TDALTLNAAVNNLLNKDYSDVSLYSAGK.-	3119.45	3	4.98	0.64	770.9	1	32/112	3.31E9
	109.32 - 110.63	-.QGGSSDVTPNGFSAM*NTGFM*PPLAAIER.-	2886.17	2	2.68	0.58	138.0	16	12/54	3.17E9
	12.32	-.QGSSVTSLSDTAATR.-	1481.55	2	3.56	0.48	654.1	1	16/28	2.39E9
	11.61	-.SVEGVDVESGTGK.-	1264.32	2	3.29	0.54	1224.2	1	20/24	2.64E9
	77.62	-.SYLNWNETENK.-	1398.46	2	3.69	0.52	1527.3	1	17/20	2.06E9
	76.62	-.TGGLEISIR.-	946.08	2	2.89	0.39	1160.3	1	15/16	1.10E9
	94.02	-.WLSSVNAGLNLQESNK.-	1760.93	2	2.77	0.38	854.3	1	17/30	2.20E9
	73.10	-.YDNRDGQLGSLTGGYDR.-	1887.95	2	3.43	0.56	648.5	1	20/32	2.63E9
	74.80	-.YDNRDGQLGSLTGGYDR.-	1887.95	2	2.60	0.54	638.4	1	17/32	1.43E9
#5	DNAK_ECOLI (P04475					330.38			33 (33 0 0 0 0)	5.58
	112.23 - 113.52	-.AKLESLVEDLVNR.-	1486.70	2	3.55	0.59	1152.1	1	17/24	6.40E9
	112.34	-.AKLESLVEDLVNR.-	1486.70	3	4.09	0.26	1410.6	1	26/48	1.44E9
	88.55 - 89.09	-.DAEANAEADRKFEELVQTR.-	2193.32	2	2.85	0.30	377.0	2	15/36	1.63E9
	88.63 - 89.21	-.DAEANAEADRKFEELVQTR.-	2193.32	3	4.71	0.53	1030.2	1	26/72	3.01E9
	88.90	-.HSQVFSTAEDNQSAVTIHVLQGER.-	2654.83	3	4.01	0.49	1562.7	1	31/92	2.86E9
	86.68 - 88.05	-.HSQVFSTAEDNQSAVTIHVLQGER.-	2654.83	3	7.56	0.67	2468.2	1	40/92	5.68E9
	105.83 - 107.74	-.IELSSAQQTDVNLPYITADATGPK.-	2533.77	2	2.65	0.47	432.4	1	15/46	4.74E9
	89.73 - 89.81	-.IIAADNGDAWVEVK.-	1501.67	1	2.19	0.31	415.8	1	13/26	1.77E9
	89.32 - 89.90	-.IIAADNGDAWVEVK.-	1501.67	2	3.27	0.57	886.1	1	16/26	3.43E9
	102.32 - 103.46	-.IIGIDLGTTNSCVAIM*DGTTPR.-	2322.62	2	6.46	0.75	1971.0	1	25/42	4.91E9
	105.21	-.IIGIDLGTTNSCVAIM*DGTTPR.-	2322.62	2	3.87	0.66	920.3	1	19/42	2.73E9
	113.64 - 113.87	-.IIGIDLGTTNSCVAIMDGTTPR.-	2306.62	2	2.72	0.46	250.7	1	15/42	3.98E9
	115.19	-.IIGIDLGTTNSCVAIMDGTTPR.-	2306.62	2	4.00	0.59	1067.9	1	20/42	2.34E9
	101.52 - 102.03	-.IINEPTAAALAYGLDK.-	1660.89	1	3.31	0.49	816.4	1	17/30	1.72E9
	101.34 - 101.91	-.IINEPTAAALAYGLDK.-	1660.89	2	5.44	0.63	1254.8	1	24/30	1.03E10
	101.61	-.IINEPTAAALAYGLDK.-	1660.89	3	3.41	0.32	1573.5	1	29/60	1.06E9
	125.84 - 126.44	-.KTAEDYLGEPVTEAVITVPAYFNDAQR.-	2999.28	3	3.00	0.33	562.0	1	32/104	5.58E9
	107.53	-.LESLVEDLVNR.-	1287.45	2	3.33	0.30	1280.5	1	17/20	1.48E9
	98.86 - 98.95	-.LINYLVEEFKK.-	1396.66	1	2.43	0.23	441.5	10	11/20	9.76E8
	98.57 - 99.14	-.LINYLVEEFKK.-	1396.66	2	3.39	0.41	985.9	1	16/20	2.37E9
	68.01 - 69.22	-.M*APPQISAEVLKK.-	1428.72	2	2.78	0.53	704.4	1	19/24	2.23E9
	92.12 - 92.68	-.QAVTNPQNTLFAIK.-	1545.77	2	3.28	0.52	382.9	1	15/26	5.88E9
	94.13	-.RIINEPTAAALAYGLDK.-	1817.08	2	5.21	0.60	1595.2	1	20/32	2.08E9
	95.99	-.SLGQFNLDGINPAPR.-	1599.77	2	3.22	0.52	711.4	1	15/28	4.35E9
	100.13 - 101.46	-.SLGQFNLDGINPAPR.-	1599.77	2	4.46	0.58	1114.4	1	17/28	9.87E9
	130.76 - 131.77	-.TAEDYLGEPVTEAVITVPAYFNDAQR.-	2871.10	2	3.87	0.60	823.3	1	23/50	1.30E10
	143.47	-.TAIESALTALETALKGEDK.-	1962.19	3	3.02	0.44	511.9	1	30/72	1.59E9
	95.93 - 96.51	-.TFEVLATNGDTHLGGEDFDSR.-	2282.37	2	5.60	0.58	1793.0	1	27/40	6.00E9
	97.31 - 97.93	-.TTPSIIAYTQDGETLVGQPAK.-	2191.42	2	4.90	0.55	1039.9	1	19/40	2.71E9
	104.14 - 104.71	-.TTPSIIAYTQDGETLVGQPAK.-	2191.42	2	5.19	0.61	1378.6	1	22/40	7.24E9
	98.91 - 99.49	-.TTPSIIAYTQDGETLVGQPAKR.-	2347.61	2	4.17	0.56	553.8	1	18/42	2.35E9
	57.22 - 58.25	-.VAEFFGKEPR.-	1180.34	2	2.75	0.45	1046.3	1	16/18	1.01E9
	103.48	-.VLENAEGDRTTPSIIAYTQDGETLVGQPAK.-	3175.45	3	4.33	0.52	993.9	1	34/116	2.34E9
#6	Q8XEB4 (Q8XEB4) Fo					280.27			28 (28 0 0 0 0)	3.83
	121.24 - 121.81	-.DAIPTQSVLTITSNVVYGK.-	2007.27	2	3.94	0.63	757.9	1	19/36	5.36E9
	115.74	-.DGISYTFSIVPNALGKDDEVR.-	2297.51	2	3.29	0.55	287.3	1	14/40	2.14E9

	94.42	-.EM*LLDAM*ENPEKYPQLTIR.-	2324.66	2	2.88	0.51	140.4	2	12/36		2.49E9
	114.33 - 114.73	-.FLNTLYTM*GPSPEPNM*TILWSEK.-	2703.08	2	4.23	0.65	420.8	1	15/44		4.47E9
	59.66	-.GAVASLTSVAK.-	1004.16	2	2.68	0.40	1095.4	1	16/20	1	8.11E8
	92.28 - 93.39	-.ITEQEAQEM*VDHLVM*K.-	1934.18	2	4.01	0.54	312.5	1	15/30		3.60E9
	77.27	-.KSGVLTGLPDAYGR.-	1434.62	2	3.90	0.24	1228.6	1	18/26		2.09E9
	99.05	-.KTHNQGVFDVYTPDILR.-	2004.24	2	4.01	0.61	1061.5	1	17/32		2.23E9
	134.34 - 134.94	-.NYTPYEGDESFLAGATEATTTLWDK.-	2781.92	2	3.09	0.56	457.1	1	16/48		4.58E9
	151.59 - 152.47	-.NYTPYEGDESFLAGATEATTTLWDKVM*EG	3441.72	3	3.06	0.41	232.5	4	29/120		3.27E9
	84.99 - 86.20	-.SEPIKGDLLNYDEVM*ER.-	2025.23	2	4.36	0.61	824.7	1	18/32		5.10E9
	85.89 - 86.46	-.SEPIKGDLLNYDEVM*ER.-	2025.23	3	3.48	0.52	572.2	3	23/64		2.80E9
	96.74	-.SEPIKGDLLNYDEVMER.-	2009.23	3	3.32	0.52	649.6	1	27/64		1.71E9
	96.62	-.SEPIKGDLLNYDEVMER.-	2009.23	2	3.10	0.53	515.8	1	15/32		2.21E9
	87.88 - 88.40	-.SGVLTGLPDAYGR.-	1306.45	2	3.08	0.52	832.8	1	18/24		3.40E9
	87.97 - 88.17	-.SGVLTGLPDAYGR.-	1306.45	1	1.85	0.29	201.4	5	11/24		1.74E9
	101.01 - 101.57	-.THAPVDFDTAVASTITSHDAGYINK.-	2632.82	3	4.99	0.70	1135.4	1	42/96		4.26E9
	106.49 - 107.02	-.THNQGVFDVYTPDILR.-	1876.06	2	5.04	0.63	1786.9	1	19/30		3.76E9
	106.68	-.THNQGVFDVYTPDILR.-	1876.06	3	3.77	0.34	953.1	1	25/60		2.03E9
	72.18	-.TM*LYAINGGVDEK.-	1427.61	2	3.60	0.27	1502.0	1	19/24		1.84E9
	132.97 - 133.53	-.TPEYDELFSGDPIWATESIGGM*GLDGR.-	2931.14	3	5.35	0.56	887.8	1	30/104		6.58E9
	133.00 - 133.55	-.TPEYDELFSGDPIWATESIGGM*GLDGR.-	2931.14	2	2.62	0.42	212.3	2	13/52		4.81E9
	100.99	-.VALYGIDYLM*K.-	1302.56	2	3.49	0.54	1132.0	1	17/20		2.41E9
	93.56 - 94.17	-.VDDLAVDLVER.-	1244.38	2	3.84	0.43	2072.6	1	19/20		3.37E9
	55.02 - 56.21	-.VVGLQTEAPLKR.-	1311.56	2	2.97	0.44	966.5	1	16/22		1.84E9
	56.81 - 57.99	-.VVGLQTEAPLKR.-	1311.56	2	2.72	0.42	980.5	1	15/22		1.82E9
	148.41 - 148.93	-.YGYDISGPATNAQEAIQWTYFGYLAAVK.-	3099.40	3	5.28	0.52	1099.8	1	31/108		4.75E9
	148.39 - 149.55	-.YGYDISGPATNAQEAIQWTYFGYLAAVK.-	3099.40	2	3.73	0.62	472.1	1	15/54		3.17E9
#7	ADHE_ECOLI (P17547					270.31			27 (27 0 0 0 0)		4.51
	87.37 - 88.01	-.AAYSSGKPAIGVGAGNTPVVIDETADIKR.-	2859.19	3	5.46	0.56	1685.1	1	40/112		2.50E9
	128.69 - 129.27	-.EAGVQEADFLANVDKLSEDAFDDQCTGANF	3384.52	3	6.14	0.59	1804.3	1	39/120		7.66E9
	129.10	-.EAGVQEADFLANVDKLSEDAFDDQCTGANF	3384.52	2	2.53	0.55	339.0	1	16/60		1.21E9
	60.65	-.EYASFTQEQVDK.-	1445.51	1	2.30	0.43	222.2	1	13/22		6.58E8
	63.20 - 64.43	-.FATHGGYLLQGK.-	1292.47	2	3.43	0.33	1535.4	1	20/22		1.24E9
	126.39 - 127.40	-.FLFNNGYADQITSVLK.-	1831.06	2	4.09	0.47	1748.9	1	22/30		1.30E10
	128.00 - 128.72	-.FLFNNGYADQITSVLK.-	1831.06	2	4.69	0.54	1613.4	1	21/30		1.01E10
	99.25 - 100.37	-.GSLPIALDEVITDGHKR.-	1822.06	2	3.03	0.50	534.8	1	16/32		3.61E9
	92.93 - 93.48	-.IAELAGFSVPENTK.-	1476.66	2	2.72	0.46	596.6	1	15/26		4.10E9
	105.14	-.ILIGEVTVVDESEPFAHEK.-	2113.35	2	4.22	0.49	858.4	1	18/36		3.43E9
	105.08	-.ILIGEVTVVDESEPFAHEK.-	2113.35	3	3.13	0.22	1003.7	1	36/72		2.92E9
	110.90 - 111.64	-.ILINTPASQGGIGDLYNFK.-	2022.29	2	5.07	0.70	888.2	1	20/36		5.09E9
	113.03	-.ILINTPASQGGIGDLYNFK.-	2022.29	3	3.21	0.48	1553.2	1	34/72		2.03E9
	115.17	-.ILINTPASQGGIGDLYNFK.-	2022.29	2	4.50	0.59	1053.0	1	22/36		2.76E9
	113.22 - 114.61	-.ILINTPASQGGIGDLYNFK.-	2022.29	2	4.53	0.62	570.4	1	17/36		1.69E10
	110.49 - 111.43	-.KGAELANSFKPDVIIALGGGSPMDAAK.-	2659.06	3	3.52	0.56	877.9	1	30/104		2.55E9
	122.29 - 122.90	-.LGSQFHIPHGLANALLICNVIR.-	2444.86	3	4.49	0.56	2402.9	1	34/84		3.90E9
	62.44 - 63.90	-.LSEDAFDDQCTGANPR.-	1796.82	2	2.76	0.37	371.3	1	15/30		3.79E9
	107.48 - 107.65	-.M*IAVTTTSGTGSEVTPFAVVTDDATGQK.-	2802.06	2	5.03	0.48	686.9	1	22/54		2.25E9
	58.72	-.NAIIFSPHPR.-	1152.33	2	3.04	0.47	921.8	1	15/18		6.03E8
	83.58	-.NGALNAAIVGQPAYK.-	1487.69	1	2.13	0.50	162.3	19	11/28		6.99E8
	79.54 - 80.60	-.NHFASEYIYNAYKDEK.-	1993.12	2	4.92	0.55	1869.5	1	20/30		2.05E9
	79.63	-.NHFASEYIYNAYKDEK.-	1993.12	3	4.50	0.56	870.8	1	26/60		1.54E9
	90.06 - 90.87	-.PAIGVGAGNTPVVIDETADIKR.-	2194.47	3	4.56	0.55	1186.8	1	36/84		3.05E9
	89.00 - 89.13	-.QILLDTYYGR.-	1242.41	2	3.18	0.38	479.2	1	13/18		1.48E9
	84.74 - 85.31	-.YAEIADHLGLSAPGDR.-	1685.82	2	4.58	0.55	1735.7	1	22/30		1.85E9
	122.22 - 122.93	-.YPLADYALTPDM*AIVDANLVM*DM*PK.-	2817.25	3	4.12	0.44	948.8	1	32/96		3.24E9
#8	Q8XCY2 (Q8XCY2) Tra					230.36			23 (23 0 0 0 0)		2.89
	77.04 - 77.65	-.AGTHDSHGAPLGDAEIALTR.-	1990.12	2	5.09	0.64	968.8	1	22/38		3.48E9
	75.93 - 77.17	-.AGTHDSHGAPLGDAEIALTR.-	1990.12	3	4.13	0.57	1063.9	1	28/76		3.51E9

	77.81	-.AGTHDSHGAPLGDAEIALTR.-	1990.12	3	3.95	0.53	1552.6	1	31/76	2.48E9
	78.86 - 80.23	-.AINEDAAGNYIHYGVR.-	1763.89	2	4.26	0.51	1665.1	1	21/30	4.89E9
	79.87	-.AINEDAAGNYIHYGVR.-	1763.89	3	3.18	0.48	714.7	1	28/60	1.71E9
	65.70	-.AVTDKPSLLMCK.-	1363.64	2	2.52	0.34	390.2	2	13/22	8.26E8
	68.17	-.AYPQEAAEFTR.-	1283.37	2	3.11	0.51	381.8	1	15/20	2.70E9
	149.51 - 150.94	-.FVLSNGHGSM*LIYSLLHLTGYDLPM*EELK.-	3311.82	3	5.34	0.44	2343.9	1	37/112	4.84E9
	90.89	-.QVM*VYTHDSIGLGEDGPTHQPVEQVASLR.	3181.48	3	5.98	0.58	909.5	1	33/112	1.82E9
	100.08	-.QVMVYTHDSIGLGEDGPTHQPVEQVASLR.-	3165.48	3	3.66	0.41	1042.7	1	33/112	2.95E9
	100.19	-.SGHPGAPM*GM*ADIAEVLWR.-	2028.30	3	4.10	0.43	2395.9	1	32/72	2.08E9
	51.33 - 52.59	-.TEEQLANIAR.-	1145.25	2	3.50	0.19	1576.7	1	16/18	1.92E9
	53.19 - 54.31	-.TEEQLANIAR.-	1145.25	2	3.45	0.16	1737.3	1	16/18	1.51E9
	51.60 - 53.91	-.TEEQLANIAR.-	1145.25	1	2.07	0.37	233.2	1	9/18	8.51E8
	71.20	-.TIIGFGSPNK.-	1034.19	2	2.65	0.46	715.5	1	15/18	8.04E8
	70.86 - 71.47	-.TIIGFGSPNK.-	1034.19	1	2.11	0.33	303.0	1	11/18	2.30E9
	71.24	-.TIIGFGSPNK.-	1034.19	1	2.06	0.34	213.6	1	10/18	1.44E9
	127.69	-.TPGHPEVGYTAGVETTTGPLGQGIANAVGM	3412.77	3	4.24	0.50	740.6	1	32/136	3.20E9
	124.44 - 125.55	-.TPGHPEVGYTAGVETTTGPLGQGIANAVGM	3412.77	3	7.27	0.72	1805.0	1	38/136	8.29E9
	130.25 - 130.84	-.TPGHPEVGYTAGVETTTGPLGQGIANAVGM	3396.77	3	6.17	0.64	2203.3	1	43/136	3.70E9
	65.19 - 66.39	-.VVSM*PSTDAFDKQDAAYR.-	2018.19	2	3.94	0.60	586.5	1	20/34	2.11E9
	81.82 - 82.53	-.VVSMPSTDAFDKQDAAYR.-	2002.19	2	3.30	0.42	878.2	1	18/34	2.16E9
	132.76 - 134.01	-.YAPFEIPSEIYAQWDAK.-	2029.24	2	3.69	0.57	575.4	1	18/32	7.30E9
#9	PUR9_ECO57 (Q8X61					210.26			21 (21 0 0 0 0)	2.56
	103.60	-.AFEHTAAYDSM*IANYFGSM*VPAYHGESK.-	3128.40	3	4.73	0.51	1479.1	1	35/108	2.13E9
	110.95 - 111.45	-.AGIVEFAQALSAR.-	1333.52	2	4.26	0.59	1559.7	1	19/24	3.13E9
	97.66	-.ALSYNNIADTDAALECVK.-	1969.13	2	4.51	0.43	755.4	1	19/34	2.11E9
	131.25 - 132.61	-.ALSYNNIADTDAALECVKEFAEPACVIVK.-	3213.57	3	4.23	0.49	351.6	6	24/112	1.04E10
	73.36	-.DLGM*VGAEELR.-	1206.35	2	2.97	0.40	949.2	1	16/20	2.34E9
	110.09	-.EGCSLEDAVENIDIGGPTM*VR.-	2279.46	2	3.81	0.41	972.3	1	19/40	3.07E9
	116.58	-.EGCSLEDAVENIDIGGPTMVR.-	2263.46	2	3.90	0.41	844.4	1	19/40	1.93E9
	89.39	-.ELDAETAQAIISR.-	1417.55	1	2.02	0.37	202.8	1	12/24	7.48E8
	89.02 - 89.56	-.ELDAETAQAIISR.-	1417.55	2	3.16	0.46	771.4	1	14/24	3.35E9
	103.68	-.GLPVTEVSDYTGFPEM*M*DGR.-	2234.45	2	3.36	0.60	405.1	1	14/38	4.47E9
	101.99 - 103.08	-.GLPVTEVSDYTGFPEM*M*DGR.-	2234.45	2	4.88	0.69	565.7	1	17/38	5.48E9
	110.44 - 111.05	-.GLPVTEVSDYTGFPEMM*DGR.-	2218.45	2	3.62	0.03	585.4	1	16/38	2.09E9
	63.40 - 63.92	-.GVELLSTGGTAR.-	1161.29	1	1.84	0.36	346.7	1	14/22	1.66E9
	63.49 - 64.15	-.GVELLSTGGTAR.-	1161.29	2	3.51	0.52	1549.3	1	18/22	1.05E9
	96.99 - 97.83	-.HANPCGVAIGNSILDAYDR.-	2044.21	2	5.16	0.61	1909.5	1	25/36	2.62E9
	94.87 - 95.04	-.HANPCGVAIGNSILDAYDR.-	2044.21	2	3.28	0.51	832.5	1	17/36	1.96E9
	105.64 - 106.23	-.QFVEVIIAPSASEEALK.-	1832.09	2	3.30	0.14	530.9	1	15/32	3.48E9
	35.17 - 36.23	-.RVNGGLLVQDR.-	1227.40	2	2.59	0.30	535.6	2	14/20	3.74E8
	113.44 - 113.81	-.TDPTSAFGGIIAFNR.-	1567.73	2	4.75	0.48	1759.4	1	22/28	4.65E9
	60.73	-.VNGGLLVQDR.-	1071.21	2	2.62	0.39	992.2	1	15/18	6.47E8
	82.49	-.YGENSHQQAAFYIEENVK.-	2128.24	2	4.18	0.55	1817.2	1	22/34	1.42E9
#10	HTPG_ECOLI (P10413					190.31			19 (19 0 0 0 0)	1.70
	80.00 - 81.22	-.ALSNPDLYEGDGELR.-	1649.74	2	3.79	0.62	849.6	1	18/28	3.35E9
	138.10	-.DDAEKYQTFWQQFGLVLK.-	2217.47	2	2.53	0.43	434.6	1	13/34	2.59E9
	81.76	-.DEVIDHLGTIAK.-	1311.47	2	3.54	0.39	1207.7	1	17/22	1.71E9
	73.51 - 74.03	-.EGPAEDFANQEAIAK.-	1590.67	2	3.19	0.64	795.3	1	18/28	2.89E9
	73.12	-.ELISNASDAADKLR.-	1503.64	2	3.89	0.45	1495.8	1	19/26	1.58E9
	85.27	-.FASTHTDSSAQTVSLEDYVSR.-	2302.40	2	6.24	0.73	2021.9	1	24/40	1.96E9
	101.13 - 101.70	-.GLIDSSDLPLNVSR.-	1486.65	2	2.95	0.47	1083.9	1	19/26	3.59E9
	67.67 - 67.79	-.GTEITLHLR.-	1040.20	2	2.64	0.27	872.6	1	13/16	1.25E9
	70.91 - 71.51	-.LTDTPAIVSTDADEM*STQM*AK.-	2258.47	2	4.96	0.72	760.2	1	21/40	2.09E9
	72.59	-.LTDTPAIVSTDADEM*STQM*AK.-	2258.47	2	3.46	0.53	494.6	1	15/40	1.44E9
	84.60 - 85.66	-.LTDTPAIVSTDADEMSTQM*AK.-	2242.47	2	2.62	0.01	320.6	1	13/40	2.76E9
	72.08	-.NKSEITDEEYKEFYK.-	1924.05	2	4.61	0.39	1441.9	1	20/28	1.29E9
	72.10	-.NKSEITDEEYKEFYK.-	1924.05	3	4.54	0.27	1078.0	1	28/56	1.26E9

	84.89	-.SFLESLGSDQAK.-	1282.38	2	3.95	0.46	1322.4	1	18/22	1.27E9
	103.31 - 103.94	-.VFIM*DDAEQFM*PNYLR.-	2022.29	2	3.68	0.37	416.3	1	13/30	2.59E9
	114.86	-.VFIMDDAEQFM*PNYLR.-	2006.29	2	3.02	0.42	437.4	1	13/30	2.08E9
	106.00	-.YIFELNPDHVLVK.-	1587.84	2	3.02	0.51	1300.3	1	19/24	2.11E9
	90.21	-.YSDHIALPVEIEK.-	1514.71	2	3.93	0.40	1236.0	1	18/24	1.69E9
	82.65	-.YSDHIALPVEIEKR.-	1670.89	2	3.31	0.39	706.6	1	15/26	1.85E9
#11	G6PI_ECOLI (P11537)					190.30			19 (19 0 0 0 0)	2.59
	125.22	-.AITDVVNIGIGGSDLGPYM*VTEALRPYK.-	2967.39	2	2.72	0.56	122.8	3	13/54	3.02E9
	124.97 - 126.21	-.AITDVVNIGIGGSDLGPYM*VTEALRPYK.-	2967.39	3	5.99	0.58	1210.6	1	39/108	6.89E9
	97.33 - 98.49	-.DQGKDPATLDYVVPFK.-	1794.00	2	3.00	0.40	905.4	1	19/30	3.78E9
	144.01 - 144.94	-.EITPFSLGALIALYEHK.-	1903.21	2	2.51	0.43	446.1	1	13/32	3.17E9
	95.49 - 96.06	-.EVVEQEYRDQGKDPATLDYVVPFK.-	2827.10	3	5.67	0.58	1222.7	1	33/92	3.68E9
	96.64	-.FSATFDDQM*LVDYSK.-	1783.94	2	4.02	0.72	800.5	1	17/28	2.01E9
	11.49	-.HFAALSTNAK.-	1060.19	1	2.04	0.18	542.2	1	13/18	2.17E9
	101.40 - 102.52	-.HFDEM*KDVTIADLFAK.-	1897.14	3	3.11	0.46	1546.7	1	27/60	2.67E9
	101.48 - 102.18	-.HFDEM*KDVTIADLFAK.-	1897.14	2	4.55	0.64	1021.5	1	20/30	2.82E9
	79.40	-.ILPELKDDKEISSHDSSTNGLINR.-	2682.93	2	3.79	0.52	734.1	1	19/46	1.70E9
	81.10 - 82.11	-.ILPELKDDKEISSHDSSTNGLINR.-	2682.93	3	3.09	0.44	1163.4	1	32/92	3.74E9
	81.04	-.ILPELKDDKEISSHDSSTNGLINR.-	2682.93	2	3.89	0.58	542.4	1	17/46	1.32E9
	94.28	-.KVNPETTLFLVASK.-	1547.82	2	2.95	0.38	819.0	1	17/26	2.66E9
	139.52	-.LLSNFFAQTEALAFGK.-	1758.01	1	2.33	0.21	166.8	63	9/30	1.17E9
	139.40 - 140.74	-.LLSNFFAQTEALAFGK.-	1758.01	2	5.30	0.61	1055.4	1	20/30	8.22E9
	83.23 - 84.51	-.M*KNINPTQTAAWQALQK.-	1960.25	2	2.65	0.39	323.1	1	13/32	3.27E9
	92.85 - 93.04	-.NINPTQTAAWQALQK.-	1684.88	2	3.94	0.55	751.3	1	18/28	3.60E9
	91.29	-.SREVVEQEYRDQGKDPATLDYVVPFK.-	3070.36	3	3.83	0.48	628.1	1	27/100	9.25E8
	106.60 - 107.13	-.VNPETTLFLVASK.-	1419.65	2	2.72	0.47	319.0	1	14/24	3.09E9
#12	RPOB_ECOLI (P00575					170.31			17 (17 0 0 0 0)	1.86
	71.55	-.ALEIEEM*QLK.-	1220.42	2	3.04	0.47	877.3	1	14/18	8.68E8
	151.57	-.ALNYTTEQILDLFFEK.-	1946.19	2	4.68	0.54	2039.5	1	20/30	3.46E9
	70.72 - 71.26	-.AVLVAGGVEAEKLDK.-	1499.74	2	3.45	0.54	1087.6	1	20/28	1.10E9
	104.41	-.FIEQDPEGQYGLEAAFR.-	1971.12	2	4.33	0.65	612.1	1	17/32	3.67E9
	107.82	-.GDVLADGPSTDLGELALGQNM*R.-	2246.44	2	3.94	0.46	787.7	1	17/42	2.34E9
	130.67 - 131.42	-.INPIEDM*PYDENGTPVDIVLNPLGVPSR.-	3082.43	3	4.56	0.57	603.0	1	28/108	3.40E9
	130.80	-.INPIEDM*PYDENGTPVDIVLNPLGVPSR.-	3082.43	2	3.71	0.57	659.6	1	18/54	2.01E9
	140.28	-.KDLSEELQILEAGLFSR.-	1949.19	2	4.65	0.61	1473.2	1	21/32	3.17E9
	140.36	-.KDLSEELQILEAGLFSR.-	1949.19	3	3.74	0.44	830.5	1	25/64	8.19E8
	68.98 - 69.89	-.LGDLPTSGQIR.-	1157.30	2	2.88	0.34	769.7	1	18/20	1.39E9
	103.50 - 104.04	-.LIEVPVEYIAGK.-	1331.58	2	2.77	0.37	446.9	1	13/22	3.09E9
	90.66	-.LSALVEIYR.-	1064.26	2	3.04	0.43	1007.9	1	13/16	6.81E8
	109.94 - 111.30	-.LSLGDLDTLM*PQDM*INAKPISAAVK.-	2675.12	3	3.51	0.27	419.2	20	23/96	7.14E9
	77.75 - 77.97	-.STGSYSLVTQQPLGGK.-	1623.79	2	3.77	0.47	720.7	1	16/30	2.44E9
	78.02	-.STGSYSLVTQQPLGGK.-	1623.79	1	2.38	0.45	282.4	1	13/30	8.96E8
	110.17 - 110.84	-.SVFPIQSYSGNSELQYVSYR.-	2325.52	2	6.24	0.58	2136.7	1	27/38	4.08E9
	108.74	-.VNEDEM*YPGEAGIDIYNLTK.-	2288.47	2	3.41	0.51	246.0	1	14/38	2.38E9
#13	Q8X633 (Q8X633) Out					160.29			16 (16 0 0 0 0)	2.33
	72.92	-.AGVLNLGDKDLSR.-	1358.53	2	2.88	0.48	946.0	1	16/24	1.80E9
	93.52 - 94.05	-.DAPASISVITQEDLQR.-	1743.90	2	4.06	0.65	1190.1	1	18/30	2.73E9
	111.13	-.DGNVEFAWTPNQNHDFTAGYGFDR.-	2759.84	2	2.66	0.49	355.6	1	16/46	1.25E9
	122.35 - 123.58	-.DRGDTYNGQFFTSGPLIDGVLGM*K.-	2605.87	3	3.72	0.46	1403.4	1	33/92	6.18E9
	124.19 - 125.50	-.DRGDTYNGQFFTSGPLIDGVLGM*K.-	2605.87	3	3.44	0.51	1215.4	1	29/92	5.59E9
	63.67 - 64.89	-.EVPGVQLTNEGDNR.-	1528.61	2	3.16	0.42	322.0	1	17/26	2.02E9
	123.33 - 124.49	-.GPM*SSLYGSDALGGVVNIITK.-	2096.39	2	5.76	0.63	1708.9	1	24/40	5.29E9
	15.94 - 16.59	-.HDKLSDAVNLTGGTSSK.-	1730.86	2	2.51	0.34	276.0	1	13/32	3.66E8
	118.74	-.HNDFDLNWIPVDSIER.-	1971.12	2	4.26	0.67	917.3	1	19/30	2.85E9
	108.76 - 109.34	-.IFEPLALTTGVR.-	1317.56	2	3.57	0.28	1038.9	1	18/22	4.25E9
	46.63 - 48.31	-.LSDAVNLTGGTSSK.-	1350.46	2	3.64	0.63	1197.9	1	21/26	4.35E8
	50.75 - 52.07	-.LSDAVNLTGGTSSK.-	1350.46	2	4.17	0.60	1234.2	1	21/26	9.65E8

	48.82 - 50.15	-.LSDAVNLTGGTSSK.-	1350.46	2	4.24	0.65	1375.9	1	22/26	9.85E8
	110.01 - 111.20	-.TPGGYTIWNTGAAWQVTK.-	1952.16	2	4.75	0.55	902.8	1	21/34	4.19E9
	115.86	-.TSASQYALFVEDEWR.-	1802.92	2	5.21	0.62	1291.4	1	20/28	8.67E9
	130.31 - 131.49	-.TSDVNAAPGYQNFVGFTANGVPVFSYYNVN	3342.62	3	4.60	0.47	542.4	1	31/120	6.40E9
#14	TIG_ECOLI (P22257) T					160.23			16 (16 0 0 0 0)	1.63
	138.87	-.ANDIDVPAALIDSEIDVLR.-	2040.26	2	4.11	0.58	407.6	1	19/36	4.01E9
	140.91 - 141.97	-.ANDIDVPAALIDSEIDVLR.-	2040.26	2	4.51	0.61	557.1	1	21/36	2.93E9
	64.66 - 64.74	-.ASDFVLAM*GQGR.-	1268.43	2	2.95	0.54	642.0	1	14/22	1.12E9
	110.03	-.ELPELTAEFIK.-	1290.49	2	3.07	0.51	772.6	1	16/20	2.52E9
	75.26	-.FAINLK.-	705.87	1	1.83	0.21	547.6	4	8/10	1.40E9
	80.41 - 81.68	-.FGVEDGSVEGLR.-	1265.36	2	3.11	0.49	574.2	1	17/22	3.24E9
	138.85	-.GLIEEMASAYEDPKEVIEFYSK.-	2549.84	3	3.95	0.54	1606.6	1	33/84	2.01E9
	138.83	-.GLIEEMASAYEDPKEVIEFYSK.-	2549.84	2	3.35	0.56	443.7	1	18/42	2.42E9
	75.89 - 76.00	-.INPAGAPTYVPGEYK.-	1577.76	1	2.98	0.39	434.3	1	13/28	9.68E8
	75.53 - 76.18	-.INPAGAPTYVPGEYK.-	1577.76	2	2.93	0.51	537.5	1	16/28	3.93E9
	113.83	-.NVALEEQAVEAVLAK.-	1584.80	1	1.89	0.23	250.7	12	10/28	1.22E9
	113.56 - 114.88	-.NVALEEQAVEAVLAK.-	1584.80	2	4.03	0.56	2190.5	1	21/28	4.46E9
	94.58 - 95.72	-.RVTITIAADSIETAVK.-	1688.95	2	2.62	0.09	639.7	3	15/30	3.56E9
	57.06 - 57.68	-.SQAIEGLVK.-	945.10	2	2.60	0.33	419.4	3	12/16	4.92E8
	71.09 - 71.76	-.VTEKETTFNELM*NQQA.-	1900.06	2	2.72	0.27	404.0	5	11/30	1.24E9
	126.89	-.VVVGLLLGEVIR.-	1267.59	2	4.36	0.56	1673.8	1	20/22	2.06E9
#15	EFG_ECOLI (P02996)					140.26			14 (14 0 0 0 0)	2.15
	102.86 - 103.44	-.DVTTGDTLCDPDAPIILER.-	2102.28	2	5.15	0.48	1464.3	1	23/36	6.44E9
	78.24 - 78.82	-.EFNVEANVGKPQVAYR.-	1822.01	2	3.90	0.45	754.0	1	18/30	2.86E9
	85.97	-.GQYGHVVIDM*YPLEPGSNPK.-	2218.48	2	4.85	0.59	898.8	1	18/38	1.82E9
	95.12 - 95.68	-.HASDDEPFSALAFK.-	1535.64	2	4.49	0.59	1213.0	1	20/26	3.85E9
	126.61 - 127.18	-.IATDPFVGNLTFFR.-	1598.83	2	3.85	0.58	1372.4	1	21/26	6.66E9
	99.64	-.IHAEVPLSEM*FGYATQLR.-	2079.37	2	2.53	0.40	154.4	12	10/34	1.59E9
	149.89 - 150.64	-.LGANPVPLQLAIGAEEHFTGVVDLVK.-	2689.10	3	4.91	0.58	448.0	1	30/100	4.92E9
	90.40	-.LHFGSYHDVDSSELAFK.-	1953.10	2	4.30	0.64	1608.2	1	20/32	2.13E9
	107.84 - 109.13	-.M*EFPEPVISIAVEPK.-	1702.99	2	4.02	0.63	584.7	1	18/28	5.58E9
	49.77	-.NIGISAHIDAGK.-	1196.34	2	2.71	0.44	1110.8	1	16/22	3.20E8
	96.26 - 96.83	-.VEVETPEENTGDVIGDLSR.-	2060.16	2	3.79	0.56	503.3	1	18/36	3.68E9
	79.94 - 81.02	-.VYSGVVNSGDTVLNSVK.-	1738.92	2	2.98	0.33	463.7	1	15/32	3.41E9
	89.69 - 90.25	-.VYSGVVNSGDTVLNSVK.-	1738.92	2	4.85	0.61	1034.6	1	20/32	3.89E9
	85.56	-.YLGGEELTEAEIK.-	1452.59	2	4.48	0.49	1160.7	1	21/24	2.64E9
#16	Q8XDA4 (Q8XDA4) Ol					140.25			14 (14 0 0 0 0)	1.44
	104.33	-.AEQQLDKDSAIVPVYYYVNAR.-	2443.70	2	2.90	0.51	345.1	1	14/40	2.42E9
	105.75	-.AGWCADYNEPTSFLNTM*LSNSSM*NTAHYI	3344.58	3	3.57	0.30	701.2	1	29/112	2.78E9
	74.40	-.AQGDM*PAYGYTPPYTDGAK.-	2020.17	2	2.63	0.39	631.0	1	17/36	1.82E9
	72.67 - 73.83	-.AQGDM*PAYGYTPPYTDGAK.-	2020.17	2	4.12	0.59	668.1	1	16/36	1.68E9
	125.93	-.DLFEGLLVSDLDGHPAPGVAESWDNKDAK.-	3097.34	3	5.10	0.56	1074.5	1	34/112	2.52E9
	111.18 - 111.92	-.LLAEAGYTADKPLTINLLYNTSDLHK.-	2876.25	3	4.98	0.51	619.0	3	25/100	3.17E9
	91.31	-.LVKPWVGGYTGKDPLDNTYTR.-	2381.67	3	3.03	0.49	648.4	1	32/80	1.59E9
	82.67	-.NNGSEVQSLDPHKIEGVPESNISR.-	2607.78	2	2.83	0.32	163.5	2	14/46	9.45E8
	99.33	-.SGEIDM*TYNNM*PIELFQK.-	2163.41	2	4.04	0.60	543.7	1	15/34	1.65E9
	108.65	-.SGEIDMTYNNM*PIELFQK.-	2147.42	2	3.88	0.38	452.2	1	13/34	1.94E9
	85.62 - 85.70	-.SPAFDSIM*AETLK.-	1426.62	2	2.64	0.47	1125.1	1	18/24	2.14E9
	115.90 - 116.43	-.TVINQVTYLPIASEVTDVNR.-	2233.51	2	5.07	0.61	739.1	1	21/38	4.34E9
	115.97 - 117.15	-.WSDGTPVTAQDFVYSWQR.-	2144.29	2	3.43	0.61	838.0	1	18/34	4.32E9
	97.37	-.WTQPGNIVTNGAYTLK.-	1763.98	2	2.89	0.49	281.1	1	13/30	2.01E9
#17	Q7DB97 (Q7DB97) Ou					130.30			13 (13 0 0 0 0)	1.38
	77.56 - 78.68	-.DLLQEGQSSGFR.-	1337.42	2	3.20	0.50	1475.8	1	17/22	3.55E9
	78.89	-.DLLQEGQSSGFR.-	1337.42	1	2.50	0.42	308.4	1	14/22	1.43E9
	107.99 - 109.20	-.FDDLM*LSNDALEFK.-	1674.85	2	4.17	0.47	1050.6	1	19/26	4.93E9
	117.98	-.FYTNYWVPNPNLRPETNETQEYGFGLR.-	3307.58	3	3.40	0.55	473.1	1	26/104	3.97E9
	119.53 - 120.37	-.FYTNYWVPNPNLRPETNETQEYGFGLR.-	3307.58	3	3.10	0.42	449.2	1	28/104	4.10E9

	80.25	-.LAPQGNDWLNADAK.-	1513.64	1	2.05	0.41	531.6	1	14/26	1.27E9
	48.22	-.QSNGETAPNDESINNM*LAK.-	2050.15	2	2.84	0.48	231.7	1	14/36	1.70E8
	50.43 - 51.14	-.QSNGETAPNDESINNM*LAK.-	2050.15	2	2.51	0.43	353.2	1	16/36	3.32E8
	52.77 - 54.10	-.QSNGETAPNDESINNM*LAK.-	2050.15	2	2.90	0.41	295.3	1	16/36	3.77E8
	107.95 - 108.51	-.SSFEAPM*M*VSVIDTSAPENQTATSATDLLF	3203.50	3	4.66	0.56	1180.0	1	33/116	3.80E9
	108.28	-.SSFEAPM*M*VSVIDTSAPENQTATSATDLLF	3203.50	2	3.29	0.48	307.8	1	17/58	2.11E9
	91.43 - 91.52	-.VFGTGGTGDHSLGLGASAFGR.-	1965.12	2	5.95	0.73	2271.2	1	27/40	2.61E9
	113.48 - 114.25	-.YTTDLFSLDVAYNR.-	1678.82	2	2.99	0.28	1061.3	1	16/26	3.21E9
#18	RPOC_ECOLI (P0057					130.22			13 (13 0 0 0 0)	1.07
	99.87 - 100.48	-.AIVQLEDGVQISSGDTLAR.-	1973.17	2	3.91	0.48	865.4	1	17/36	1.81E9
	46.98 - 49.56	-.DITGGLPR.-	828.94	1	1.85	0.15	151.9	2	8/14	4.09E8
	72.22 - 73.06	-.FTDM*IDGQTITR.-	1414.57	2	3.39	0.55	912.9	1	15/22	1.86E9
	115.32	-.GDGEQVAGGETVANWDPHTM*PVITEVSGF	3273.54	3	3.69	0.39	514.8	1	27/120	2.61E9
	80.87	-.GDVISDGPEAPHDILR.-	1691.82	2	2.71	0.29	910.5	1	19/30	2.00E9
	102.68	-.GEAIGVIAAQSIGEPGTQLTM*R.-	2216.50	2	3.74	0.60	334.9	1	16/42	1.78E9
	86.74	-.IFGPVKDYECLCGK.-	1686.92	2	3.29	0.49	333.9	4	11/26	1.60E9
	118.04	-.LGIQAFEPVLIEGK.-	1514.79	2	3.54	0.53	735.7	1	16/26	3.64E9
	112.88	-.LLDLAAPDIIVR.-	1309.58	2	3.17	0.41	1092.5	1	18/22	1.41E9
	95.62	-.LVITPVDGSDPYEEM*IPK.-	2020.29	2	4.20	0.63	1022.6	1	20/34	2.07E9
	121.35	-.QTDELTGLSSLVVLDSAER.-	2034.21	2	4.32	0.65	1352.5	1	21/36	3.22E9
	62.54 - 63.47	-.VPYGAVLAK.-	918.12	1	2.00	0.20	569.6	1	10/16	8.23E8
	86.22	-.YIVNEVQDVYR.-	1398.55	2	3.76	0.54	1466.5	1	17/20	1.56E9
#19	ODP1_ECOLI (P0695					120.28			12 (12 0 0 0 0)	1.17
	127.42	-.AQYLIDQLLAEAR.-	1504.71	2	4.55	0.44	1909.4	1	19/24	2.04E9
	94.88	-.ARNEQDGGDLVYFQGHISPGVYAR.-	2650.85	3	3.07	0.33	797.5	2	26/92	2.22E9
	49.89	-.ATVILAHTIK.-	1067.31	2	2.72	0.55	1102.7	1	16/18	3.79E8
	48.18 - 49.22	-.ATVILAHTIK.-	1067.31	2	2.94	0.53	1004.2	1	15/18	4.64E8
	45.68 - 47.37	-.ATVILAHTIK.-	1067.31	2	3.34	0.47	1392.4	1	16/18	4.39E8
	149.95	-.DWLQAIESVIREEGVER.-	2030.23	2	3.20	0.41	278.9	1	15/32	2.18E9
	114.06	-.EKLDNLVFVINCNLQR.-	1976.26	2	2.97	0.55	567.0	1	15/30	2.23E9
	55.88 - 56.57	-.FNIDADKVNPR.-	1289.42	2	2.53	0.48	846.7	1	13/20	8.33E8
	143.72	-.IINELEGIFEGAGWNVIK.-	2003.29	2	2.64	0.44	772.2	1	16/34	3.25E9
	145.18 - 145.76	-.LELPSLQDFGALLEEQSK.-	2018.25	2	5.53	0.66	1773.6	1	23/34	6.73E9
	94.75	-.LIQLM*NETVDGDYQTFK.-	2032.26	2	3.27	0.43	826.2	1	16/32	3.74E9
	78.58 - 79.15	-.QIGIYSPNGQQYTPQDR.-	1966.10	2	4.20	0.60	885.5	1	20/32	2.53E9
#20	IMDH_ECOLI (P06981					120.25			12 (12 0 0 0 0)	1.07
	96.85	-.AKYPDLQIIGGNVATAAGAR.-	1987.25	2	4.53	0.58	1263.6	1	22/38	2.56E9
	131.73	-.EALTFDDVLLVPAHSTVLPNTADLSTQLTK.-	3211.61	2	2.74	0.37	52.0	37	11/58	1.71E9
	142.20 - 142.80	-.ELTERNGFAGYPVVTEENELVGIITGR.-	2965.26	3	4.98	0.59	1525.6	1	34/104	3.61E9
	90.27 - 90.83	-.FVTDLNQPVSVYM*TPK.-	1856.13	2	3.89	0.54	472.7	1	16/30	3.55E9
	101.97 - 103.26	-.FVTDLNQPVSVYMTPK.-	1840.13	2	3.02	0.30	446.2	1	15/30	2.87E9
	86.66	-.HESGVVTDPQTVLPTTTLR.-	2052.28	2	3.53	0.62	373.4	1	16/36	1.90E9
	15.74	-.ISGAGIQESHVHDVTITK.-	1893.09	2	2.91	0.47	809.2	1	16/34	2.65E8
	30.95 - 32.41	-.ISGAGIQESHVHDVTITK.-	1893.09	2	2.97	0.50	638.8	1	16/34	4.09E8
	81.16	-.KHESGVVTDPQTVLPTTTLR.-	2180.45	2	4.69	0.55	638.7	1	21/38	1.36E9
	97.95 - 99.16	-.LNIPM*LSAAM*DTVTEAR.-	1866.15	2	3.19	0.60	496.9	1	18/32	2.75E9
	80.47	-.SCM*GLTGCGTIDELR.-	1686.85	2	3.25	0.52	722.1	1	18/28	2.35E9
	88.98	-.YFQSDNAADKLVPEGIEGR.-	2110.27	2	4.71	0.57	1121.6	1	20/36	1.48E9
#21	Q8X8H4 (Q8X8H4) Ou					120.24			12 (12 0 0 0 0)	1.27
	121.64	-.AEQGSYALVDLFTR.-	1570.73	2	2.72	0.57	1361.5	1	17/26	3.31E9
	75.71 - 76.85	-.GATGLM*TGTGNPSAAINM*VR.-	1952.20	2	3.35	0.49	351.5	2	15/38	2.28E9
	120.91	-.GFQIDNYM*VDGIPTYFESR.-	2269.48	2	3.42	0.64	354.6	1	15/36	2.14E9
	73.23 - 74.44	-.IEQDNVAQSTGTPIPGSNGETAYK.-	2478.61	2	2.72	0.59	515.8	1	16/46	2.49E9
	84.47	-.IVGGYQNNDSWLDR.-	1637.74	2	2.81	0.55	1009.8	1	16/26	1.53E9
	135.77 - 136.40	-.KVDALDLFADGSYELFGR.-	2017.23	2	4.21	0.57	937.4	1	17/34	3.24E9
	66.76 - 67.30	-.STAPDWAYNDKEINK.-	1752.86	2	3.04	0.30	307.7	8	12/28	8.68E8
	126.25	-.VTLADPLHLILGAR.-	1489.79	2	3.48	0.52	775.4	1	17/26	2.13E9

	126.74 - 127.35	-.WNLGDALSDM*ALFER.-	1754.95	2	4.87	0.68	1483.7	1	22/28	2.04E9
	71.37 - 72.51	-.YIAEDNEGNAVNPNLPR.-	1887.00	2	4.83	0.61	1339.4	1	21/32	4.33E9
	99.08 - 99.60	-.YLAPITGNNYELGLK.-	1666.90	2	3.71	0.61	620.7	1	16/28	2.69E9
	83.47 - 84.02	-.YVADLQSPLTEDGK.-	1536.67	2	2.74	0.39	940.7	1	17/26	2.22E9
#22	CLPB_ECO57 (P6328!					110.27			11 (11 0 0 0 0)	0.99
	110.28	-.AIDLIDEAASSIR.-	1374.52	2	4.00	0.59	1635.1	1	19/24	1.85E9
	93.96	-.ALANFM*FDSDEAM*VR.-	1749.95	2	3.59	0.59	558.8	1	14/28	1.82E9
	74.42	-.GELHCVGATTLDEYR.-	1721.84	2	4.29	0.64	1467.7	1	21/28	1.18E9
	81.12	-.GYEIHISDEALK.-	1375.51	2	2.74	0.37	897.0	1	15/22	2.36E9
	82.03	-.LPQVEGTGGDVQPSQDLVR.-	1996.17	2	4.10	0.56	926.2	1	18/36	1.93E9
	106.94	-.LVGAPPGYVGYEEGGYLTEAVR.-	2298.54	2	3.76	0.54	458.4	1	22/42	1.67E9
	100.75	-.NTVVIM*TSNLGSDLIQER.-	2007.26	2	5.40	0.64	1562.4	1	21/34	1.92E9
	122.18 - 123.41	-.VFVAEPSVEDTIAILR.-	1760.02	2	3.62	0.47	595.1	1	20/30	3.58E9
	94.98	-.VIGQNEAVDAVSNAIR.-	1656.82	2	4.14	0.51	1218.9	1	18/30	3.01E9
	88.81	-.VLALDM*GALVAGAK.-	1345.63	2	3.07	0.48	1183.7	1	17/26	1.73E9
	97.91	-.VTDAEIAEVLAR.-	1287.45	2	2.96	0.37	900.6	1	18/22	1.88E9
#23	KPY1_ECOLI (P14178					100.26			10 (10 0 0 0 0)	1.79
	51.21	-.ELALQSGLAHK.-	1167.34	2	2.56	0.31	723.3	1	14/20	7.27E8
	48.00 - 49.27	-.ELALQSGLAHK.-	1167.34	2	2.58	0.55	1016.2	1	15/20	5.93E8
	108.06 - 108.98	-.GAVETAEKLDAPLIVVATQGGK.-	2168.48	2	4.72	0.60	713.2	1	17/42	5.22E9
	130.46 - 131.13	-.GDLGVEIPVEEVIFAQK.-	1844.10	2	4.08	0.63	802.2	1	22/32	1.64E10
	122.27 - 122.86	-.GVNLPGVSIALPALAEK.-	1649.96	2	4.35	0.62	825.4	1	20/32	7.04E9
	125.38	-.IENQEGLNNFDEILEASDGIM*VAR.-	2694.91	2	4.64	0.61	807.5	1	18/46	2.43E9
	105.17 - 106.18	-.KYFPDATILALTTNEK.-	1826.08	2	4.81	0.62	1370.7	1	23/30	4.09E9
	98.18 - 98.84	-.LDAPLIVVATQGGK.-	1382.63	2	5.26	0.60	1541.6	1	21/26	3.06E9
	12.38	-.RSDVIEIR.-	988.12	2	2.71	0.05	685.9	5	12/14	1.25E9
	83.02	-.TAAILLDTKGPEIR.-	1498.75	3	3.14	0.37	2062.5	1	35/52	6.80E8
#24	FTSH_ECO57 (Q8X9L					100.25			10 (10 0 0 0 0)	1.31
	124.14 - 125.30	-.ALGVTFFLPEGDAISASR.-	1852.08	2	3.71	0.48	514.4	1	19/34	6.05E9
	151.06 - 151.35	-.GTPGFSGADLANLVNEAALFAAR.-	2263.50	2	4.87	0.54	512.5	1	19/44	4.49E9
	87.30	-.LAEEIIYGPEHVSTGASNDIK.-	2244.44	3	3.54	0.36	1534.7	1	34/80	1.87E9
	86.42 - 87.82	-.LAEEIIYGPEHVSTGASNDIK.-	2244.44	2	2.80	0.43	507.8	1	16/40	3.70E9
	136.53 - 137.15	-.LGPLLYAEEEGEVFLGR.-	1893.13	2	5.01	0.68	1759.1	1	22/32	3.03E9
	77.52 - 77.67	-.QKLESQISTLYGGR.-	1580.77	2	2.84	0.55	409.2	1	14/26	1.73E9
	98.53 - 99.20	-.RVPLAPDIDAAIIAR.-	1591.88	2	3.52	0.48	804.3	1	17/28	3.15E9
	139.56	-.TTFADVAGCDEAKEEVAELVEYLR.-	2716.93	2	3.46	0.57	660.6	1	16/46	2.41E9
	135.20	-.TTFADVAGCDEAKEEVAELVEYLREPSR.-	3186.42	3	3.53	0.50	623.6	1	29/108	2.48E9
	73.93 - 74.53	-.YTTYIPVQDPK.-	1325.49	2	2.77	0.53	594.2	1	15/20	1.49E9
#25	SYD_ECO57 (Q8XCI7)					100.21			10 (10 0 0 0 0)	1.11
	71.62 - 72.82	-.ADVLPLDSNHVNTEEAR.-	1880.99	2	4.06	0.65	596.3	1	17/32	1.86E9
	118.11	-.DLGSLIFIDM*R.-	1296.52	2	3.27	0.63	924.3	1	15/20	1.65E9
	119.24 - 120.46	-.EGIVQVFFDPDRADALK.-	1921.14	2	3.12	0.51	349.5	1	13/32	4.47E9
	143.20 - 143.33	-.FLNAEIIEAILDR.-	1517.75	2	4.16	0.55	1769.9	1	18/24	2.82E9
	107.93	-.FYALPQSPQLFK.-	1439.68	2	2.53	0.50	431.5	1	15/22	1.60E9
	60.07	-.GLEGINSPVAK.-	1085.24	1	2.03	0.38	495.4	1	13/20	5.32E8
	114.96 - 116.20	-.GVDLGDFPVM*TFAEAER.-	1871.06	2	3.91	0.51	779.6	1	21/32	4.68E9
	129.66 - 130.48	-.GVDLGDFPVMTFAEAER.-	1855.06	2	2.58	0.44	467.1	1	15/32	3.02E9
	113.71	-.NPM*ELTDVADLLR.-	1503.70	2	4.29	0.42	1398.9	1	18/24	2.73E9
	84.35	-.VAALCVPGGASLTR.-	1372.59	2	3.04	0.49	661.6	1	18/26	2.21E9
#26	YJJK_ECOLI (P37797)					90.27			9 (9 0 0 0 0)	0.66
	54.75 - 56.13	-.FEELNSTEYQK.-	1388.46	2	3.28	0.22	1676.5	1	16/20	6.92E8
	103.62	-.FLHDFEGTVVAITHDR.-	1858.05	3	4.44	0.58	1907.8	1	32/60	1.59E9
	87.28	-.IGYLPQEPQLNPEHTVR.-	1992.22	2	2.99	0.50	318.8	2	14/32	1.53E9
	74.09	-.IM*AGIDKDIEGEARPQPDIK.-	2213.50	2	3.06	0.50	243.9	1	14/38	1.07E9
	88.36	-.LEEIIQAHDGHNLNVQLER.-	2229.44	2	3.20	0.48	331.4	3	13/36	1.09E9
	125.67	-.LLIDDLSFSIPK.-	1361.61	2	3.69	0.50	927.2	1	16/22	1.75E9
	125.82 - 126.27	-.LLLEKPDM*LLLDEPTNHLDAESVAWLER.-	3278.72	3	3.23	0.33	996.4	1	30/108	2.84E9

	78.84 - 79.38	-.M*ISGQEQPDSGTITLGETVK.-	2108.31	2	4.04	0.56	825.8	1	24/38		2.18E9
	113.62	-.RLDEVYALYADPDADFDKLAAEQGR.-	2843.05	3	5.46	0.57	883.2	1	33/96		2.49E9
#27	GLMS_ECO57 (Q8XE(					80.25			8 (8 0 0 0 0)		0.67
	122.63	-.ELGYLGSLAICNVPGSSLVR.-	2106.40	2	4.24	0.60	646.0	1	18/38		3.65E9
	96.55	-.GDQYPIALEGALK.-	1375.55	2	3.05	0.51	587.2	1	17/24		1.93E9
	118.30	-.GLDASIEHDIVHGLQALPSR.-	2129.36	3	5.02	0.54	2072.0	1	34/76		2.28E9
	118.40	-.GLDASIEHDIVHGLQALPSR.-	2129.36	2	3.55	0.56	985.0	1	18/38		2.32E9
	104.20	-.ISHGQVDLSELGPNADELLSK.-	2223.43	2	5.10	0.56	1151.0	1	21/40		2.53E9
	56.77 - 57.42	-.RQDIESNLQYDAGDK.-	1752.82	2	4.81	0.61	1139.6	1	19/28		7.76E8
	54.92 - 56.09	-.RQDIESNLQYDAGDK.-	1752.82	2	4.81	0.65	1498.9	1	20/28		6.52E8
	75.79	-.RQDIESNLQYDAGDKGIYR.-	2242.39	2	3.27	0.54	399.7	1	15/36		1.30E9
#28	ENTE_ECO57 (Q8XB\					80.25			8 (8 0 0 0 0)		0.95
	113.67 - 114.77	-.EQGIAEFKLPDRVECVDSLPLTAVGK.-	2873.25	3	3.34	0.45	416.4	2	24/100		3.47E9
	136.19 - 136.78	-.GYWQDLPLTDILTR.-	1691.91	2	2.79	0.40	506.8	1	16/26		3.26E9
	57.82 - 58.62	-.HAASDSIAVIDGER.-	1441.53	2	3.52	0.60	1886.1	1	22/26		7.27E8
	123.91	-.IAAEEIENLLLR.-	1384.60	2	3.78	0.44	1671.1	1	17/22		2.73E9
	146.49 - 147.05	-.IPAEIGCQLQQVFGM*AEGLVNYTR.-	2712.07	3	3.75	0.52	975.7	1	29/92		2.94E9
	121.51 - 122.13	-.SELNAYASQIEPALLIADR.-	2075.31	2	4.99	0.63	1131.4	1	21/36		4.53E9
	117.96	-.SELNAYASQIEPALLIADR.-	2075.31	2	4.11	0.62	1002.8	1	19/36		1.92E9
	76.98 - 77.44	-.SVEICQFTQQTR.-	1497.63	2	3.38	0.52	1485.1	1	17/22		2.40E9
#29	SYK1_ECO57 (Q8XD5					80.24			8 (8 0 0 0 0)		0.56
	70.43	-.ALRPLPDKFHGLQDQEAR.-	2092.35	2	3.52	0.49	370.0	1	15/34		1.17E9
	70.59	-.ALRPLPDKFHGLQDQEAR.-	2092.35	3	4.84	0.62	1662.3	1	31/68		8.67E8
	85.50 - 86.84	-.ASFVTLQDVGGR.-	1250.39	2	3.31	0.49	946.8	1	16/22	1	1.98E9
	91.66	-.EIGNGFSELNDAEDQAQR.-	1994.02	2	3.88	0.15	681.0	1	14/34		1.80E9
	81.86 - 82.38	-.GFM*EVETPM*M*QVIPGGAAAR.-	2141.48	2	4.32	0.52	706.8	1	18/38		1.49E9
	110.72 - 111.28	-.TEVTYGDVTLDFGKPFEK.-	2047.25	2	3.46	0.15	974.5	1	18/34		3.18E9
	65.31	-.TGELSIHCTELR.-	1416.56	2	2.69	0.41	730.3	1	14/22		1.04E9
	72.80 - 72.86	-.YRPETDM*ADLDNFDSAK.-	2005.11	2	3.38	0.47	634.1	1	18/32		1.44E9
#30	EAE_ECO57 (P43261)					70.32			7 (7 0 0 0 0)		1.07
	95.79	-.ALNYAAQQAASLGSQLQSR.-	1978.16	2	5.04	0.60	1052.2	1	18/36		1.72E9
	102.16	-.FNGYLPSYPALGAK.-	1498.71	2	2.68	0.69	392.3	1	17/26		1.97E9
	122.43 - 123.62	-.GELPNIWLQYGQFK.-	1693.93	2	3.72	0.61	681.6	1	16/26		3.97E9
	106.14 - 106.29	-.KNGVAQANVPVSFNIVSGTATLGANSAK.-	2717.03	3	3.99	0.52	1936.3	1	38/108		4.00E9
	132.91 - 134.11	-.LPFEYSALPLLGSAPLVAAGGVAGHTNK.-	2752.16	3	6.34	0.65	2360.0	1	43/108		5.45E9
	100.63 - 101.23	-.SQGGQIQHSGSQSAQDYQAILPAYVQGGSN	3482.72	3	4.12	0.51	754.9	1	37/128		4.25E9
	96.97 - 97.53	-.SWSQQIEPQYVNELR.-	1878.04	2	3.37	0.51	471.5	1	12/28		3.39E9
#31	Q8X8W2 (Q8X8W2) P					70.27			7 (7 0 0 0 0)		0.79
	100.00 - 100.65	-.AGPGSLGPVNM*PIPVVIDR.-	1906.24	2	4.69	0.63	984.3	1	19/36		2.52E9
	117.94 - 118.58	-.AGPGSLGPVNMPIPVVIDR.-	1890.24	2	3.02	0.52	470.5	1	16/36		2.61E9
	83.95	-.AQGIEVLLDDRK.-	1357.54	2	3.94	0.48	1329.1	1	17/22		1.20E9
	109.15 - 110.26	-.GERPFVLGPTHEEVITDLIR.-	2279.58	3	5.33	0.61	1479.6	1	30/76		3.63E9
	114.40 - 114.44	-.LPQVASPLTFATEEEIR.-	1902.14	2	4.64	0.63	429.7	1	15/32		3.92E9
	83.43 - 84.30	-.NVVAGDPSPDGQGTLLIK.-	1781.99	2	2.70	0.50	456.0	1	16/34		2.24E9
	122.99	-.TIAELVEQFNLPIKK.-	1744.07	2	3.88	0.65	816.9	1	16/28		2.08E9
#32	Q8X5Q2 (Q8X5Q2) Oli					70.25			7 (7 0 0 0 0)		1.21
	127.54 - 128.77	-.AEFGVDELQPWDIAYYSEK.-	2261.43	2	4.72	0.52	419.9	1	16/36		7.93E9
	136.71 - 137.24	-.EAYEQTLPLLSEYSTWVGQHEGLYK.-	2943.21	3	3.88	0.51	295.1	3	24/96		2.85E9
	101.63	-.FFELYDENNELR.-	1589.69	2	2.69	0.38	692.7	1	14/22		3.26E9
	68.40 - 69.32	-.ILPEHVVPAVTK.-	1303.58	2	2.94	0.56	440.8	1	15/22		1.29E9
	92.22 - 93.42	-.LVTDEAELAGM*PESALAAAK.-	2004.25	2	2.98	0.35	351.8	2	13/38		5.16E9
	146.00	-.M*AENPQQVLDFLTDLAK.-	1950.20	2	5.08	0.50	1036.9	1	21/32		3.21E9
	133.65 - 134.41	-.M*TNPLLTPFELPPFSK.-	1849.18	2	2.76	0.43	528.7	1	14/30		4.20E9
#33	SYGB_ECO57 (P6703					70.25			7 (7 0 0 0 0)		0.63
	126.33 - 127.75	-.FAGDDLPSNPVACALAIADK.-	2046.26	2	2.58	0.31	74.3	83	9/38		5.41E9
	85.58	-.HDGEAEDVAVALNEQYQPR.-	2142.23	2	4.93	0.58	1199.9	1	22/36		1.50E9
	136.82	-.IQALAGWIAEQIGADVNHATR.-	2235.49	3	3.31	0.41	551.4	4	27/80		1.34E9

	107.61	-.LQTVLFQQQLGTLR.-	1645.93	2	4.05	0.52	1551.3	1	19/26		2.29E9
	72.24	-.TLEAAAALAAANKR.-	1371.57	2	3.16	0.53	1829.9	1	20/26		1.72E9
	22.12 - 23.64	-.VANLAEAQPDREIEK.-	1683.85	2	2.74	0.51	220.9	3	13/28		1.75E8
	96.39	-.VIPATILGIQSDR.-	1383.62	2	3.43	0.50	801.0	1	18/24		2.24E9
#34	Q8XBF1 (Q8XBF1) Tra					70.21			7 (7 0 0 0 0)		0.73
	68.00	-.AGKEEAHGAPLGEEEVALAR.-	2035.20	2	4.26	0.52	516.1	1	18/38		1.10E9
	32.95 - 36.42	-.EAILEAQSVK.-	1088.24	1	2.29	0.29	261.9	1	12/18		5.27E8
	81.12	-.GSVSLKEDPAGNYIHYGVR.-	2063.26	3	4.21	0.48	2269.5	1	35/72		2.36E9
	140.68	-.LFPFFGFTAENIVAK.-	1701.99	2	2.79	0.60	614.7	1	18/28		2.53E9
	104.77	-.VAVEAGIADYWYK.-	1485.67	1	1.94	0.31	611.7	1	12/24		1.77E9
	104.68 - 105.60	-.VAVEAGIADYWYK.-	1485.67	2	3.28	0.54	946.5	1	16/24		4.33E9
	113.98	-.VVSLPSTDIFDAQDEEYRESVLPSNVAAR.-	3209.47	3	3.47	0.43	275.7	1	27/112		4.24E9
#35	GUAA_ECO57 (P6429					70.21			7 (7 0 0 0 0)		1.01
	77.08	-.FLSALAGENDPEAK.-	1462.59	2	3.81	0.51	1566.3	1	23/26		2.24E9
	77.19	-.FYGVQFHPEVTHTR.-	1718.90	2	3.99	0.63	1215.2	1	19/26		1.56E9
	120.84 - 122.16	-.IGLELGLPYDM*LYR.-	1669.97	2	3.00	0.49	453.0	1	15/26		3.86E9
	136.86 - 138.18	-.LNEAEQVLDM*FGDHFGLNIVHVPAEDR.-	3083.38	3	4.17	0.47	1143.4	1	31/104		3.17E9
	114.98	-.VSQAFTVFLPVR.-	1364.62	2	2.89	0.47	1184.1	1	15/22		2.38E9
	102.82 - 104.06	-.VVYDISGKPPATIEWE.-	1805.02	2	3.65	0.56	1036.5	1	19/30		3.41E9
	116.77	-.WLAQGTIYPDVIESAASATGK.-	2179.42	2	3.89	0.56	554.0	1	18/40		6.79E9
#36	NUSA_ECOLI (P0300З					60.27			6 (6 0 0 0 0)		0.64
	60.96	-.EHEGEIITGVVK.-	1311.47	2	2.58	0.44	626.8	1	14/22		1.19E9
	18.77 - 25.39	-.EITLEAAR.-	903.02	1	2.10	0.19	138.8	1	9/14		3.50E8
	118.67 - 119.22	-.ELLEIEGLDEPTVEALR.-	1927.14	2	5.09	0.47	1723.6	1	22/32		3.97E9
	123.95	-.NALATIAQAQEESLGDNKPADDLLNLEGVDF	3282.52	3	5.43	0.64	1063.8	1	37/120		3.86E9
	95.56 - 96.14	-.WLVVDEVTQPTK.-	1415.62	2	3.51	0.49	1150.3	1	17/22		2.67E9
	146.02 - 146.10	-.YEDESLNLGDYVEDQIESVTFDR.-	2737.82	2	4.87	0.50	1051.7	1	22/44		2.75E9
#37	GLNA_ECOLI (P06711					60.23			6 (6 0 0 0 0)		0.86
	33.42	-.AINALANPTTNSYKR.-	1634.82	2	3.88	0.52	1390.8	1	20/28		2.36E8
	150.04 - 151.17	-.EIPQVAGSLEEALNELDLDREFLK.-	2730.02	3	3.26	0.58	370.3	1	31/92		8.00E9
	150.68	-.EIPQVAGSLEEALNELDLDREFLK.-	2730.02	2	2.83	0.45	138.2	21	10/46		1.82E9
	110.93 - 111.56	-.FGSSISGSHVAIDDIEGAWNSSTQYEGGNK.	3115.23	3	4.59	0.44	1457.8	1	35/116		3.94E9
	98.07 - 99.31	-.GGYFPVPPVDSAQDIR.-	1718.89	2	3.21	0.44	492.5	1	18/30		3.76E9
	81.00	-.GKEQHVTIPAHQVNAEFFEEGK.-	2496.72	3	4.08	0.56	1431.9	1	30/84		2.22E9
#38	Q8X966 (Q8X966) Pyr					60.22			6 (6 0 0 0 0)		0.69
	128.43 - 128.66	-.DVNVPDIGSDEVEVTEILVK.-	2171.39	2	3.74	0.60	363.1	1	18/38		3.92E9
	88.19 - 89.27	-.FGEIEEVELGR.-	1278.39	2	2.84	0.06	1984.9	1	18/20		1.64E9
	71.43	-.ILREDVQAYVK.-	1334.55	2	2.68	0.19	571.9	1	13/20		1.14E9
	88.44	-.SEFAENDAYVHATPLIR.-	1934.10	3	3.55	0.54	1005.7	1	28/64		1.56E9
	119.72 - 120.35	-.TGSLIM*IFEVEGAAPAAAPAK.-	2061.39	2	4.02	0.65	589.7	1	15/40		2.87E9
	131.15 - 131.71	-.VPDIGADEVEITEILVK.-	1841.09	2	4.43	0.24	1298.7	1	20/32		4.72E9
#39	GPMI_ECO57 (Q8XDE					50.22			5 (5 0 0 0 0)		0.47
	103.79	-.AFFANPVLTGAVDK.-	1450.66	2	2.68	0.35	907.5	1	18/26		2.44E9
	73.97	-.AVEALDHCVEEVAK.-	1570.72	2	4.37	0.41	1642.0	1	19/26		1.43E9
	115.09 - 115.88	-.TPVM*DALWANRPHTLIDASGLEVGLPDR.-	3062.45	3	3.33	0.34	603.4	5	25/108		3.16E9
	80.33	-.VATYDLQPEM*SSAELTEK.-	2029.21	2	3.12	0.37	253.6	1	15/34		2.14E9
	92.02	-.VATYDLQPEMSSAELTEK.-	2013.21	2	2.74	0.34	327.4	1	16/34		1.72E9
#40	Q8XCR6 (Q8XCR6) Ar					50.19			5 (5 0 0 0 0)		0.45
	87.99	-.DKNVLLVDDSIVR.-	1486.70	2	3.86	0.48	1040.0	1	15/24		2.27E9
	134.45	-.DVDQGYLDFLDTLR.-	1670.80	2	3.09	0.49	916.4	1	15/26		2.61E9
	59.94 - 60.77	-.GTTSEQIIEM*AR.-	1352.50	2	3.82	0.51	668.5	1	13/22		8.58E8
	68.19	-.KVYLASAAPEIR.-	1318.55	2	3.41	0.49	730.0	1	16/22		1.57E9
	131.88	-.QCADNPVSNPCLFEYVYFARPDSFIDK.-	3254.54	3	3.17	0.38	417.0	8	23/104		3.03E9
#41	DCEA_ECO57 (P5822					50.17			5 (5 0 0 0 0)		0.29
	115.55	-.LGPYEFICTGRPDEGIPAVCFK.-	2527.84	3	3.31	0.53	1220.9	1	31/84	1	2.58E9
	46.96 - 47.83	-.LQGIAQQNSFK.-	1234.39	2	2.68	0.38	644.2	1	14/20	1	2.57E8
	45.12 - 46.31	-.LQGIAQQNSFK.-	1234.39	2	2.70	0.47	551.6	1	14/20	1	2.65E8

	42.65 - 44.03	-.LQGIAQQNSFK.-	1234.39	2	2.51	0.38	688.4	1	15/20	1	2.19E8
	119.81 - 121.18	-.VQNASYQVAAYLADEIAK.-	1955.16	2	2.76	0.53	565.5	1	14/34	1	3.45E9
#42	PPCK_ECO57 (Q8X73					40.29			4 (4 0 0 0 0)		0.57
	130.02 - 130.54	-.GVLTNLGAVAVDTGIFTGR.-	1862.12	2	3.45	0.63	1272.2	1	20/36		4.45E9
	118.15	-.LFIDNFDKYTDTPAGAALVAAGPK.-	2496.80	3	5.77	0.60	2548.8	1	38/92		2.74E9
	98.05	-.LTADQTQYHFLSGFTAK.-	1929.12	2	4.67	0.62	1066.4	1	18/32		2.13E9
	91.12 - 92.30	-.VSYPIYHIDNIVKPVSK.-	1973.30	2	2.71	0.24	242.7	5	12/32		3.76E9
#43	SYT_ECO57 (Q8XE27					40.22			4 (4 0 0 0 0)		0.32
	60.98 - 62.28	-.GKDLGSM*DVNEVIEK.-	1650.83	2	4.05	0.59	413.8	1	18/28		9.93E8
	117.81 - 119.01	-.M*AIGPVIDNGFYYDVDLDR.-	2190.42	2	4.49	0.55	1271.2	1	21/36		3.28E9
	78.08	-.TLTQEDVEALEK.-	1376.49	2	2.60	0.45	677.4	2	14/22		1.76E9
	74.78 - 74.90	-.TLTQEDVEALEKR.-	1532.68	2	2.75	0.48	1231.1	1	16/24		1.40E9
#44	Q8XE55 (Q8XE55) Put					30.31			3 (3 0 0 0 0)		0.21
	62.87	-.ISEHKPEAVKPLADVQEQVK.-	2246.55	3	3.62	0.52	1073.0	1	31/76		8.37E8
	62.73	-.ISEHKPEAVKPLADVQEQVK.-	2246.55	2	3.44	0.54	324.9	1	16/38		9.52E8
	79.29 - 80.64	-.VSDAASNDTESLAGAEQAAGVK.-	2092.17	2	6.11	0.63	1867.2	1	25/42		3.00E9
#45	Q7DB77 (Q7DB77) Pu					30.28			3 (3 0 0 0 0)		0.37
	71.18	-.GQLINSTGPLGSR.-	1300.45	2	3.90	0.56	1486.7	1	18/24		1.16E9
	77.38	-.LLGNPSAGIQSTYAR.-	1548.73	2	3.38	0.58	678.5	1	17/28		2.56E9
	126.91 - 128.14	-.VNIDELGNAIPSGVLKDDVVANIEEQAK.-	2952.26	3	5.57	0.52	1204.7	1	35/108		4.78E9
#46	PUR4_ECO57 (Q8XA4					30.28			3 (3 0 0 0 0)		0.30
	123.27	-.FSLVEVTQSPSLLLQGM*VGSQM*PIAVSHG	3388.86	3	4.43	0.52	555.5	1	30/124		2.18E9
	117.59	-.LAVGEALTNIAATQIGDIKR.-	2055.37	3	3.73	0.42	730.7	1	30/76		1.41E9
	150.02 - 150.77	-.LGLALAEDEIDYLQDAFTK.-	2126.35	2	5.53	0.58	2423.1	1	25/36		3.38E9
#47	Q8X714 (Q8X714) Out					30.22			3 (3 0 0 0 0)		0.33
	114.38	-.APNLGQLYGFYGNPNLDPEK.-	2208.42	2	4.38	0.68	820.5	1	19/38		2.39E9
	123.14	-.LNLAGGSGSADLSQFPIALVQR.-	2215.50	2	3.92	0.53	748.1	1	17/42		3.23E9
	119.91	-.LPGVDITQNGGSGQLSSIFIR.-	2160.42	2	3.27	0.60	571.1	1	15/40		2.01E9
#48	CARB_ECO57 (P6373					30.22			3 (3 0 0 0 0)		0.38
	126.78 - 126.87	-.LAVGYTLDELM*NDITGGR.-	1955.18	2	4.35	0.58	815.8	1	17/34		2.97E9
	96.60 - 97.01	-.TPASFEPSIDYVVTK.-	1654.84	2	2.82	0.49	361.6	2	15/28		3.23E9
	111.81	-.VAEVGITGLNAEFLR.-	1589.82	2	2.78	0.54	1047.9	1	17/28		2.56E9
#49	Q8XBJ9 (Q8XBJ9) Asp					30.21			3 (3 0 0 0 0)		0.27
	131.51	-.EIFEELFPLPSAAECVPGGPSVACSSAK.-	2951.26	2	2.77	0.47	348.5	1	17/54		2.03E9
	53.93 - 55.11	-.EVAAQQVSDQQLETAR.-	1773.88	2	4.29	0.39	944.1	1	19/30		1.05E9
	93.14 - 94.33	-.LSIVDVNAGAQPLYNQQK.-	1959.19	2	3.30	0.51	288.9	2	12/34		3.24E9
#50	Q8XBW7 (Q8XBW7) C					30.21			3 (3 0 0 0 0)		0.42
	96.08	-.APSLYQTNPNYILYSK.-	1873.10	2	4.11	0.58	1071.6	1	19/30		2.79E9
	123.25 - 124.64	-.TNFSLTGPLGDEFSFR.-	1788.94	2	4.12	0.47	870.8	1	18/30		4.75E9
	135.64	-.WDFAPLQSLELEAGYSR.-	1983.17	2	3.78	0.60	981.5	1	22/32		2.19E9
#51	SYQ_ECO57 (Q8X9H8					30.20			3 (3 0 0 0 0)		0.36
	152.88 - 153.39	-.LFSVPNPGAADDFLSVINPESLVIK.-	2644.02	2	2.95	0.23	443.5	1	16/48		4.11E9
	136.02	-.YSSDYFDQLHAYAIELINK.-	2291.50	2	4.05	0.59	1094.6	1	19/36		2.69E9
	135.94	-.YSSDYFDQLHAYAIELINK.-	2291.50	3	3.03	0.55	541.8	1	24/72		1.48E9
#52	Q8X8S8 (Q8X8S8) Hyp					30.19			3 (3 0 0 0 0)		0.19
	95.27	-.FHTQFIAISGVVR.-	1475.72	2	2.62	0.35	985.8	1	16/24		2.15E9
	46.18 - 46.90	-.NLEDDNDDVLEKR.-	1575.62	2	3.60	0.53	713.4	1	14/24		3.28E8
	80.68	-.SAQSHEAQSLFEITPQSTPEDKK.-	2559.73	3	3.89	0.41	828.5	1	29/88		1.84E9
#53	OPGG_ECO57 (P6755					30.18			3 (3 0 0 0 0)		0.18
	103.70	-.GLAIDTALPSGEEFPR.-	1673.85	2	3.58	0.56	605.2	1	18/30		1.65E9
	90.67	-.KLPEDTPVTAQTSIGDNGEIVESTVR.-	2757.99	2	3.64	0.57	525.1	1	19/50		1.03E9
	79.05	-.VIGAGQVYGLSAR.-	1291.48	2	3.06	0.61	1656.7	1	19/24		1.44E9
#54	Q8XDP4 (Q8XDP4) Pu					30.16			3 (3 0 0 0 0)		0.11
	59.51 - 60.79	-.HIGSNEAVYDIDR.-	1489.57	2	2.96	0.58	575.3	1	15/24		1.38E9
	53.69 - 54.54	-.INACIHNTGAEVIR.-	1568.75	2	3.15	0.53	411.9	1	15/26		5.70E8
	51.82 - 53.12	-.INACIHNTGAEVIR.-	1568.75	2	3.21	0.52	712.6	1	17/26		6.35E8
#55	YEAG_ECOLI (P77391					20.29			2 (2 0 0 0 0)		0.18

	84.26	-.LLNHSELTHAPCAPGTLETLSR.-	2418.69	3	3.29	0.28	1383.3	1	33/84	1.29E9
	153.82	-.VFNFDHVEVAANPVHLFYVLEQQIER.-	3115.49	3	5.88	0.60	2535.0	1	36/100	2.97E9
#56	Q8X774 (Q8X774) Pyri					20.27			2 (2 0 0 0 0)	0.32
	121.47 - 122.09	-.LPTQSSQLYGTNLVNLLK.-	1990.29	2	4.11	0.55	902.1	1	18/34	5.10E9
	128.51	-.VIGYTDLPGRLPTQSSQLYGTNLVNLLK.-	3062.51	3	5.48	0.55	1283.7	1	32/108	2.38E9
#57	SYM_ECO57 (Q8X7E7					20.26			2 (2 0 0 0 0)	0.25
	127.22	-.AAAAPVTGPLADDPIQETITFDDFAK.-	2675.93	2	2.75	0.51	446.8	1	18/50	2.64E9
	122.20 - 122.31	-.IDDIDLNLEDFVQR.-	1705.85	2	5.29	0.60	1976.7	1	21/26	3.22E9
#58	Q8XBT4 (Q8XBT4) Alk					20.25			2 (2 0 0 0 0)	0.17
	125.65 - 126.62	-.LIPAAVEGGLHQIETASGAVLK.-	2175.51	2	5.01	0.72	677.4	1	22/42	3.70E9
	49.15	-.M*GEIIIDAK.-	1006.20	1	1.85	0.28	324.0	1	12/16	1.56E8
#59	EFTU_ECOLI (P02990					20.23			2 (2 0 0 0 0)	0.23
	108.88	-.ELLSQYDFPGDDTPIVR.-	1966.14	2	4.62	0.65	572.4	1	18/32	3.26E9
	72.88	-.GITINTSHVEYDTPTR.-	1804.94	2	4.15	0.59	1080.2	1	19/30	1.96E9
#60	Q8X979 (Q8X979) Cyt					20.23			2 (2 0 0 0 0)	0.23
	120.56	-.M*EM*VSFSELVLNPVAQVK.-	2054.42	2	4.59	0.51	890.9	1	17/34	3.06E9
	89.94 - 90.62	-.YTPNVADATEAQIQQATK.-	1950.10	2	3.83	0.62	1057.7	1	19/34	2.33E9
#61	Q8X9G6 (Q8X9G6) Ph					20.22			2 (2 0 0 0 0)	0.29
	125.42	-.AGLTLGVDPLGGSGIEYWK.-	1934.18	2	4.42	0.62	1228.1	1	23/36	3.72E9
	91.91 - 93.08	-.DKFDLAFANDPDYDR.-	1802.88	2	3.27	0.57	945.5	1	16/28	3.03E9
#62	ENTF_ECO57 (Q8XBV					20.22			2 (2 0 0 0 0)	0.16
	79.59	-.AVVAGLAQADTLR.-	1285.48	2	3.03	0.45	1384.9	1	17/24	1.36E9
	149.10 - 149.42	-.LGSAALTATGPVLNVLPLGIHIAAQETLPELA	3409.96	3	4.42	0.52	458.5	2	29/132	2.27E9
#63	TYPA_ECOL6 (Q9EXN					20.22			2 (2 0 0 0 0)	0.12
	71.16 - 71.35	-.EGFELAVSRPK.-	1233.40	2	3.36	0.52	1047.4	1	17/20	1.07E9
	83.08	-.INIVDTPGHADFGGEVER.-	1927.06	2	4.40	0.67	916.6	1	21/34	1.65E9
#64	Q8X6L4 (Q8X6L4) Pyr					20.20			2 (2 0 0 0 0)	0.09
	82.96	-.AGGYLTDGTELHDTNFAR.-	1939.03	2	3.98	0.64	867.3	1	19/34	1.38E9
	60.75	-.ASEIDEALQR.-	1132.21	1	1.95	0.35	353.6	1	11/18	5.91E8
#65	Q8XD40 (Q8XD40) 6-p					20.19			2 (2 0 0 0 0)	0.09
	99.28	-.AEGGTGDAISGFEGSVPNPYVK.-	2153.29	2	3.83	0.57	616.4	1	18/42	1.50E9
	65.95	-.EVIASNGENL.-	1046.11	1	2.13	0.49	705.1	1	12/18	5.92E8
#66	SYR_ECO57 (Q8XCH2					20.19			2 (2 0 0 0 0)	0.23
	91.06 - 92.26	-.KAEIDEEQLAAAPVIIR.-	1867.14	2	3.32	0.44	431.1	2	16/32	3.98E9
	115.46	-.QQQENAGEM*ELADLEGFYR.-	2245.37	2	3.76	0.54	618.0	1	14/36	1.39E9
#67	Q8X8J5 (Q8X8J5) RNa					20.19			2 (2 0 0 0 0)	0.16
	107.88	-.GGDIEETAFNTNLEAADEIAR.-	2237.32	2	3.60	0.48	502.9	1	17/40	1.37E9
	135.48	-.LYTGEIPLFSHYQIESQIESAFQR.-	2858.15	3	3.72	0.53	458.8	5	24/92	2.32E9
#68	SYL_ECO57 (Q8XBN8					20.17			2 (2 0 0 0 0)	0.25
	100.89 - 101.84	-.AAENNPELAAFIDECR.-	1820.93	2	2.74	0.59	531.9	1	14/30	3.20E9
	128.60	-.NVLQPIGWDAFGLPAEGAAVK.-	2154.45	2	3.45	0.52	430.6	1	15/40	2.68E9
#69	SYK2_ECOLI (P14825					20.17			2 (2 0 0 0 0)	0.21
	88.75 - 89.83	-.EIGNGFSELNDAEDQAER.-	1995.01	2	3.39	0.35	742.0	1	15/34	3.68E9
	84.16	-.EIGNGFSELNDAEDQAER.-	1995.01	2	3.19	0.37	1003.0	1	19/34	1.27E9
#70	SYA_ECO57 (Q8X3W8					20.16			2 (2 0 0 0 0)	0.27
	78.16	-.LSGDTLDGETAFR.-	1382.46	2	3.26	0.41	902.5	1	16/24	1.74E9
	119.58 - 120.16	-.LYDTYGFPVDLTADVCR.-	2006.20	2	2.76	0.35	300.3	1	14/32	4.56E9
#71	Q8XCU8 (Q8XCU8) Ph					20.15			2 (2 0 0 0 0)	0.13
	98.59	-.GIATCVLLGNPAEINR.-	1698.94	2	3.09	0.27	947.9	1	18/30	1.72E9
	65.23	-.NTNITGVIVNK.-	1173.35	2	2.58	0.38	1110.7	1	16/20	1.20E9
#72	PYRG_ECOLI (P08398					10.24			1 (1 0 0 0 0)	0.13
	86.48 - 86.54	-.LGAQQCQLVDDSLVR.-	1702.89	2	4.87	0.67	1733.7	1	19/28	3.03E9
#73	GSH1_ECO57 (Q8X90					10.21			1 (1 0 0 0 0)	0.12
	113.73 - 114.36	-.SLDINPFSPIGVDEQQVR.-	2015.21	2	4.20	0.59	1251.5	1	22/34	2.80E9
#74	YBIT_ECOLI (P75790)					10.21			1 (1 0 0 0 0)	0.11
	109.02 - 109.67	-.ILGGDLEPTLGNVSLDPNER.-	2110.31	2	4.13	0.64	619.2	1	19/38	2.65E9
#75	OXAA_ECO57 (P6562					10.20			1 (1 0 0 0 0)	0.10

	87.72 - 88.65	-.YKFDTIADNENLNISSK.-	1973.13	2	4.05	0.61	424.3	1	13/32	2.36E9
#76	RF3_ECOLI (P33998)					10.20			1 (1 0 0 0 0)	0.11
	146.87 - 147.16	-.GLNNPDLDAAVGEDLAQQLRDELELVK.-	2937.21	3	3.94	0.38	521.9	1	30/104	2.64E9
#77	DEAD_ECO57 (Q8XA8					10.20			1 (1 0 0 0 0)	0.05
	72.12 - 72.55	-.VQQQLESSDLDQYR.-	1709.80	2	3.90	0.66	1084.4	1	17/26	1.27E9
#78	SPEA_ECO57 (Q8XCX					10.19			1 (1 0 0 0 0)	0.13
	93.98 - 94.35	-.VIESLIHSGEPLGLEAGSK.-	1937.18	2	3.71	0.66	747.7	1	18/36	3.04E9
#79	Q8XCW9 (Q8XCW9) N					10.18			1 (1 0 0 0 0)	0.06
	84.41	-.TPSYAHLQQIPAAIR.-	1666.91	2	3.66	0.55	1135.2	1	17/28	1.50E9
#80	DLDH_ECOLI (P00391					10.18			1 (1 0 0 0 0)	0.06
	127.44 - 127.58	-.VIPSIAYTEPEVAWVGLTEK.-	2203.52	2	3.59	0.57	369.4	1	16/38	1.50E9
#81	Q8X765 (Q8X765) Cat					10.18			1 (1 0 0 0 0)	0.12
	103.03	-.FLNDPQAFNEAFAR.-	1640.78	2	3.58	0.53	1316.6	1	21/26	2.66E9
#82	IF2_ECOLI (P02995) T					10.17			1 (1 0 0 0 0)	0.13
	126.41 - 126.95	-.LGAM*ATINQVIDQETAQLVAEEM*GHK.-	2831.17	3	3.32	0.40	529.7	24	24/100	2.95E9
#83	GADC_ECO57 (P5822					10.16			1 (1 0 0 0 0)	0.14
	71.64 - 72.07	-.ANTGVTLEPINSQNAPK.-	1754.92	2	3.24	0.55	400.6	1	17/32	3.13E9
#84	OSTA_ECO57 (Q8XA1					10.16			1 (1 0 0 0 0)	0.17
	132.21 - 133.42	-.VGPVSIFYSPYLQLPVGDK.-	2080.41	2	3.13	0.49	405.6	2	13/36	3.99E9
#85	Q8X9I9 (Q8X9I9) RNA					10.15			1 (1 0 0 0 0)	0.07
	84.58	-.LSQQLAM*TPQLQQAIR.-	1843.14	2	3.04	0.55	347.2	1	11/30	1.59E9
#86	Q8XB65 (Q8XB65) Put					10.15			1 (1 0 0 0 0)	0.07
	76.87	-.TDKETPYVPIPEGGVK.-	1730.94	2	3.03	0.40	500.4	1	15/30	1.60E9
#87	OMPA_ECOLI (P0293					10.15			1 (1 0 0 0 0)	0.05
	79.19	-.FGQGEAAPVVAPAPAPAPEVQTK.-	2233.51	2	3.01	0.33	343.1	1	14/44	1.08E9
#88	Q8XCB3 (Q8XCB3) Va					10.15			1 (1 0 0 0 0)	0.11
	100.96	-.IPAWYDEAGNVYVGR.-	1710.87	2	2.93	0.60	730.9	1	18/28	2.43E9
#89	Q8XAA5 (Q8XAA5) Pu					10.14			1 (1 0 0 0 0)	0.12
	104.43	-.SNTPLNVLALNQPENIENR.-	2137.34	2	2.72	0.45	391.5	1	13/36	2.73E9
#90	RvrsDB|00002655					10.14			1 (1 0 0 0 0)	0.09
	87.49	-.LDNXVSKVGDVAK.-	1358.57	2	2.70	0.21	504.7	6	12/24	2.19E9
#91	LEU2_ECO57 (Q8XA0					10.13			1 (1 0 0 0 0)	0.15
	85.54 - 86.14	-.DINACGEM*ARIQM*QELIK.-	2153.46	2	2.61	0.24	578.5	1	16/34	3.36E9
#92	Q8X9M3 (Q8X9M3) Po					10.13			1 (1 0 0 0 0)	0.10
	101.59	-.VGYINDQYVLNPTQDELK.-	2110.31	2	2.59	0.31	263.4	8	11/34	2.35E9
#93	Q8X666 (Q8X666) D-la					10.13			1 (1 0 0 0 0)	0.19
	106.81 - 107.42	-.GEQVLAYPGTTLYSLEK.-	1870.09	2	2.57	0.43	251.6	3	11/32	4.39E9
#94	Q7BSW8 (Q7BSW8) E					10.13			1 (1 0 0 0 0)	0.07
	118.17	-.ETQNFYYPETLDLTPLR.-	2101.30	2	2.56	0.35	409.5	1	14/32	1.68E9
#95	YNCE_ECO57 (Q8X9X					10.13			1 (1 0 0 0 0)	0.17
	97.61 - 98.22	-.RLYTTNADGELITIDTADNK.-	2225.40	2	2.55	0.13	605.0	1	16/38	3.97E9
#96	ILVD_ECO57 (Q8XAV					10.13			1 (1 0 0 0 0)	0.07
	114.52	-.LNIPVIFVSGGPM*EAGK.-	1746.06	2	2.54	0.53	597.5	1	14/32	1.64E9
#97	UP05_ECOLI (P39170					10.13			1 (1 0 0 0 0)	0.18
	118.96	-.SYGTDVTLGFPINEYNSLR.-	2147.33	2	2.52	0.45	243.1	1	11/36	4.14E9
#98	RvrsDB|00000257					10.11			1 (1 0 0 0 0)	0.09
	61.30 - 62.50	-.KLTKEIK.-	860.08	1	2.13	0.11	616.8	2	10/12	2.05E9
#99	Q8XCJ6 (Q8XCJ6) Glu					10.10			1 (1 0 0 0 0)	0.10
	96.78	-.TPELNLFK.-	962.13	1	1.98	0.09	658.6	1	10/14	2.22E9
#100	DAPA_ECO57 (P6394					10.10			1 (1 0 0 0 0)	0.11
	67.92 - 68.48	-.NIIGIK.-	657.83	1	1.91	0.07	308.9	1	7/10	2.47E9
#101	Q8XBA5 (Q8XBA5) Hy					10.09			1 (1 0 0 0 0)	0.02
	56.73	-.VIPGDEDAPLKR.-	1310.48	1	1.90	0.17	276.5	1	13/22	4.67E8
#102	RvrsDB|00005107					10.09			1 (1 0 0 0 0)	0.01
	24.70 - 25.57	-.SLLIPR.-	698.88	1	1.85	0.02	168.9	9	6/10	2.39E8
#103	RvrsDB|00002816					10.09			1 (1 0 0 0 0)	0.01
	50.41 - 51.07	-.QRNKLNK.-	901.05	1	1.83	0.12	324.2	2	9/12	3.01E8
