# Supplementary material for: Proteins other than the locus of enterocyte effacement-encoded proteins contribute to Escherichia coli O157:H7 adherence to bovine rectoanal junction stratified squamous epithelial cells
Source: BMC Microbiol. 2012 Jun 12;12:103. doi: 10.1186/1471-2180-12-103 (PMC3420319; doi:10.1186/1471-2180-12-103)
Supplement: Additional file 7 — http://www.biomedcentral.com/imedia/1451425738675419/supp7.pdf. DATA SHEETS: O157-DMEM MS/MS data sheet 3. [file 1471-2180-12-103-S7.pdf]

DMEM-03 SequestReport

	Reference					Score	Accession		Peptides (Hits)		Area
	Time(s)	Sequence	MH+	Charge	XC	Delta Cn	Sp	RSp	Ions	Count	Peak Area
#1	EFTU_ECOLI (P02990					820.32			82 (82 0 0 0 0)		37.33
	24.40 - 25.11	-.AFDQIDNAPEEK.-	1377.44	2	3.89	0.52	840.7	1	15/22		3.32E8
	26.78 - 27.69	-.AFDQIDNAPEEK.-	1377.44	2	2.88	0.46	717.2	1	14/22		4.87E8
	30.78 - 31.38	-.AFDQIDNAPEEK.-	1377.44	2	2.83	0.31	747.1	1	14/22		4.50E8
	20.02 - 21.82	-.AFDQIDNAPEEK.-	1377.44	2	3.52	0.42	956.4	1	16/22		5.32E8
	18.08 - 19.48	-.AFDQIDNAPEEK.-	1377.44	2	3.34	0.44	944.8	1	16/22		7.10E8
	15.87 - 17.02	-.AFDQIDNAPEEK.-	1377.44	2	3.98	0.45	966.0	1	16/22		2.60E9
	14.04 - 15.49	-.AFDQIDNAPEEK.-	1377.44	2	3.71	0.44	1166.0	1	17/22		1.05E10
	54.20 - 55.49	-.AFDQIDNAPEEK.-	1377.44	2	3.45	0.41	1041.6	1	16/22		1.72E9
	13.55 - 15.58	-.AFDQIDNAPEEK.-	1377.44	1	2.31	0.29	641.7	1	13/22		4.90E9
	56.29 - 57.58	-.AFDQIDNAPEEK.-	1377.44	2	3.60	0.50	1198.1	1	16/22		1.67E9
	58.19 - 59.29	-.AFDQIDNAPEEK.-	1377.44	2	3.18	0.40	830.6	1	15/22		2.23E9
	12.49 - 13.43	-.AFDQIDNAPEEK.-	1377.44	2	2.98	0.41	860.9	1	15/22		1.37E10
	58.62 - 59.60	-.AFDQIDNAPEEK.-	1377.44	1	2.57	0.34	684.0	1	13/22		1.17E9
	59.89 - 61.08	-.AFDQIDNAPEEK.-	1377.44	2	3.21	0.37	778.4	1	15/22		2.05E9
	22.41 - 23.53	-.AFDQIDNAPEEK.-	1377.44	2	3.74	0.42	1181.2	1	17/22		3.51E8
	70.40 - 71.49	-.AGENVGVLLR.-	1028.19	2	3.65	0.24	852.0	1	16/18		1.82E10
	69.68 - 73.53	-.AGENVGVLLR.-	1028.19	1	2.41	0.18	193.6	9	10/18		4.04E10
	73.67	-.AGENVGVLLR.-	1028.19	2	3.34	0.04	835.6	1	16/18		2.45E9
	137.85 - 138.41	-.AIDKPFLLPIEDVFSISGR.-	2118.46	2	4.04	0.54	717.8	1	21/36		9.89E10
	140.39 - 141.05	-.AIDKPFLLPIEDVFSISGR.-	2118.46	2	3.60	0.63	693.2	1	19/36		7.33E9
	136.58 - 137.87	-.AIDKPFLLPIEDVFSISGR.-	2118.46	3	4.58	0.49	1647.0	1	31/72		8.27E10
	136.14 - 137.28	-.AIDKPFLLPIEDVFSISGR.-	2118.46	2	4.58	0.57	691.9	1	20/36		1.18E11
	53.81 - 54.98	-.ALEGDAEWEAK.-	1219.28	2	3.58	0.60	1157.2	1	17/20		4.85E9
	55.57 - 56.89	-.ALEGDAEWEAK.-	1219.28	2	3.27	0.50	1118.0	1	17/20		6.53E9
	58.26 - 58.83	-.ALEGDAEWEAK.-	1219.28	2	2.82	0.49	987.1	1	16/20		1.27E9
	60.89 - 61.88	-.ALEGDAEWEAK.-	1219.28	2	2.81	0.38	909.5	1	15/20		1.74E9
	63.26 - 63.97	-.ALEGDAEWEAK.-	1219.28	2	3.05	0.55	1003.7	1	16/20		2.51E9
	52.05 - 53.24	-.ALEGDAEWEAK.-	1219.28	2	3.31	0.49	1138.3	1	17/20		4.13E9
	134.55 - 135.81	-.CDM*VDDEELLELVEM*EVR.-	2257.47	2	4.96	0.51	881.8	1	17/34		1.48E10
	145.69	-.CDM*VDDEELLELVEM*EVR.-	2257.47	2	4.21	0.24	754.2	1	15/34		7.28E9
	135.09	-.CDM*VDDEELLELVEM*EVR.-	2257.47	2	4.81	0.48	1177.5	1	18/34		1.49E10
	149.51 - 150.51	-.CDMVDDEELLELVEM*EVR.-	2241.47	2	3.99	0.33	849.1	1	17/34		1.81E10
	140.60 - 142.05	-.CDMVDDEELLELVEM*EVR.-	2241.47	2	3.48	0.44	634.8	1	15/34		1.36E10
	11.71 - 12.72	-.EHILLGR.-	837.99	1	2.17	0.44	369.9	1	9/12		5.82E9
	105.85 - 107.22	-.ELLSQYDFPGDDTPIVR.-	1966.14	2	5.22	0.66	712.1	1	20/32		1.27E11
	107.77	-.ELLSQYDFPGDDTPIVR.-	1966.14	2	4.17	0.59	742.1	1	19/32		1.73E10
	104.37 - 105.49	-.ELLSQYDFPGDDTPIVR.-	1966.14	2	3.88	0.63	602.7	1	18/32		1.54E11
	85.55 - 86.18	-.FESEVYILSK.-	1215.38	1	2.74	0.17	1127.3	1	14/18		1.19E10
	85.50 - 86.10	-.FESEVYILSK.-	1215.38	2	3.36	0.42	915.6	1	16/18		9.44E9
	85.42 - 87.54	-.FESEVYILSK.-	1215.38	1	1.81	0.14	1143.7	1	13/18		2.43E10
	87.05	-.FESEVYILSK.-	1215.38	2	2.75	0.33	885.5	1	16/18		1.22E10
	80.30 - 81.46	-.FESEVYILSKDEGGR.-	1729.87	2	3.89	0.49	928.1	1	18/28		1.06E10
	80.73	-.FESEVYILSKDEGGR.-	1729.87	1	2.95	0.36	261.5	2	13/28		2.84E9
	78.62 - 79.75	-.FESEVYILSKDEGGR.-	1729.87	2	4.23	0.55	1504.2	1	20/28		1.06E10
	74.35 - 75.61	-.GITINTSHVEYDTPTR.-	1804.94	2	4.59	0.61	1221.5	1	21/30		1.34E10
	70.68 - 71.81	-.GITINTSHVEYDTPTR.-	1804.94	2	4.76	0.62	933.2	1	18/30		5.28E10
	69.19 - 70.36	-.GITINTSHVEYDTPTR.-	1804.94	2	4.47	0.68	923.5	1	17/30		6.42E10
	71.51	-.GITINTSHVEYDTPTR.-	1804.94	3	3.37	0.59	922.3	1	25/60		4.28E9
	80.60 - 81.19	-.GQVLAKPGTIKPHTKFESEVYILSK.-	2772.24	2	3.81	0.49	276.2	1	14/48		4.29E9
	79.77 - 81.17	-.GQVLAKPGTIKPHTKFESEVYILSK.-	2772.24	3	5.38	0.55	707.8	1	32/96		5.31E9
	31.13	-.HYAHVDCPGHADYVK.-	1769.89	2	4.18	0.63	1259.6	1	18/28		4.54E8
	127.18 - 127.76	-.ILELAGFLDSYIPEPER.-	1963.22	2	4.38	0.50	1253.3	1	21/32		1.25E10
	141.14 - 142.45	-.ILELAGFLDSYIPEPER.-	1963.22	2	4.96	0.57	807.8	1	20/32		7.81E10
	139.62 - 140.78	-.ILELAGFLDSYIPEPER.-	1963.22	3	5.00	0.46	1444.1	1	34/64		6.18E9
	143.04 - 144.08	-.ILELAGFLDSYIPEPER.-	1963.22	2	3.51	0.52	573.2	1	16/32		1.28E10
	139.45 - 140.58	-.ILELAGFLDSYIPEPER.-	1963.22	2	4.84	0.54	870.1	1	21/32		1.05E11
	86.71 - 87.58	-.M*VVTLIHPIAM*DDGLR.-	1814.16	3	3.75	0.44	686.5	1	25/60		2.09E10
	86.67 - 88.04	-.M*VVTLIHPIAM*DDGLR.-	1814.16	2	4.69	0.44	626.6	1	17/30		2.85E10
	98.12 - 98.73	-.M*VVTLIHPIAMDDGLR.-	1798.16	3	3.14	0.45	1406.2	1	32/60		9.96E9
	98.14 - 99.27	-.M*VVTLIHPIAMDDGLR.-	1798.16	2	3.91	0.46	814.3	1	18/30		8.96E9
	94.12 - 95.07	-.MVVTLIHPIAM*DDGLR.-	1798.16	2	4.47	0.53	1137.6	1	20/30		1.93E10
	94.16 - 94.86	-.MVVTLIHPIAM*DDGLR.-	1798.16	3	4.14	0.49	782.9	1	30/60		7.90E9
	104.98	-.MVVTLIHPIAMDDGLR.-	1782.17	2	3.57	0.50	654.9	1	17/30		6.03E9
	105.05	-.MVVTLIHPIAMDDGLR.-	1782.17	3	3.47	0.53	697.5	1	28/60		3.32E9
	103.16 - 103.68	-.NM*ITGAAQM*DGAILVVAATDGPM*PQ	2779.17	3	5.26	0.57	1178.7	1	31/104		1.32E10
	103.12 - 104.30	-.NM*ITGAAQM*DGAILVVAATDGPM*PQ	2779.17	2	3.07	0.41	591.5	1	16/52		9.82E9
	130.33 - 131.47	-.QVGVPYIIVFLNK.-	1490.81	2	3.13	0.50	522.6	1	17/24		3.72E10
	130.28 - 131.69	-.QVGVPYIIVFLNK.-	1490.81	1	2.42	0.34	102.4	2	12/24		1.89E10
	65.00 - 65.99	-.STCTGVEMFR.-	1188.33	2	2.85	0.52	1566.2	1	16/18		3.46E9
	31.01	-.TKPHVNVGTIGHVDHGK.-	1797.01	2	2.78	0.51	689.4	1	18/32		4.62E8

	99.35 - 101.76	-.TTDVTGTIELPEGVEM*VM*PGDNIK.-	2579.88	2	3.83	0.45	444.4	1	18/46		9.09E10
	99.53 - 100.97	-.TTDVTGTIELPEGVEM*VM*PGDNIK.-	2579.88	3	6.32	0.46	1610.6	1	35/92		1.99E10
	115.26	-.TTLTAAITTVLAK.-	1304.56	2	3.62	0.56	1957.4	1	20/24		5.75E9
	120.07 - 120.91	-.TTLTAAITTVLAK.-	1304.56	2	3.38	0.50	1298.0	1	18/24		7.54E9
	113.59 - 114.68	-.TTLTAAITTVLAK.-	1304.56	2	4.14	0.58	1642.3	1	20/24		2.53E10
	111.83 - 112.97	-.TTLTAAITTVLAK.-	1304.56	2	4.34	0.55	1609.7	1	19/24		4.08E10
	79.96 - 81.07	-.VGEEVEIVGIK.-	1172.35	2	3.92	0.35	1605.8	1	17/20		1.37E10
	71.64 - 73.17	-.VGEEVEIVGIKETQK.-	1658.88	1	4.12	0.63	600.5	1	16/28		1.27E10
	71.61 - 72.95	-.VGEEVEIVGIKETQK.-	1658.88	2	4.59	0.61	1362.8	1	20/28		3.31E10
	73.51 - 74.70	-.VGEEVEIVGIKETQK.-	1658.88	2	4.38	0.63	1485.9	1	19/28		1.79E10
	71.70 - 73.08	-.VGEEVEIVGIKETQK.-	1658.88	3	4.42	0.42	924.0	1	28/56		8.97E9
	71.85	-.VGEEVEIVGIKETQK.-	1658.88	1	4.63	0.54	733.7	1	17/28		4.14E9
#2	DCEA_ECO57 (P5822					530.30			53 (53 0 0 0 0)		10.43
	83.74 - 84.28	-.CVNM*VADLWHAPAPK.-	1725.99	2	3.88	0.57	1503.9	1	19/28	1	3.27E9
	107.93 - 109.02	-.CVNMVADLWHAPAPK.-	1709.99	2	3.51	0.54	1037.2	1	18/28	1	1.57E10
	97.18	-.CVNMVADLWHAPAPK.-	1709.99	2	3.06	0.54	664.0	1	16/28	1	3.04E9
	110.90 - 111.51	-.CVNMVADLWHAPAPK.-	1709.99	2	2.70	0.55	688.2	1	14/28	1	8.09E9
	135.89	-.FGLAPLGCGWVIWR.-	1632.93	2	2.57	0.40	601.1	1	17/26	1	7.86E9
	138.89 - 139.68	-.GFEM*DFAELLLEDYK.-	1837.04	2	4.85	0.62	1813.2	1	20/28	1	1.06E10
	142.30 - 142.87	-.GFEM*DFAELLLEDYKASLK.-	2236.53	3	3.03	0.58	993.1	1	30/72	1	5.15E9
	142.61 - 143.93	-.GFEM*DFAELLLEDYKASLK.-	2236.53	2	3.71	0.47	520.5	1	16/36	1	1.08E10
	125.71 - 126.52	-.GWQVPAFTLGGEATDIVVM*R.-	2164.47	2	5.98	0.74	1357.5	1	25/38	1	8.91E9
	111.78 - 112.63	-.LGPYEFICTGRPDEGIPAVCFK.-	2527.84	3	5.38	0.59	1743.5	1	35/84	1	3.49E10
	111.85 - 112.43	-.LGPYEFICTGRPDEGIPAVCFK.-	2527.84	2	2.97	0.55	402.7	1	16/42	1	2.04E10
	87.98 - 88.50	-.LKEGEDPGYTLYDLSER.-	1986.13	2	3.55	0.52	484.0	1	20/32		1.31E10
	88.06 - 88.73	-.LKEGEDPGYTLYDLSER.-	1986.13	3	3.67	0.50	969.0	1	28/64		6.94E9
	102.18 - 102.78	-.LM*DLSINKNWIDKEEYPQSAAIDLR.-	2980.34	3	4.76	0.47	946.8	1	33/96	1	1.04E10
	53.83 - 55.02	-.LQGIAQQNSFK.-	1234.39	1	1.97	0.23	346.4	3	12/20	1	1.14E9
	53.64 - 55.12	-.LQGIAQQNSFK.-	1234.39	2	2.89	0.36	725.1	1	15/20	1	5.30E9
	19.23 - 20.71	-.LQGIAQQNSFK.-	1234.39	2	3.45	0.51	939.6	1	16/20	1	1.38E9
	28.13 - 29.59	-.LQGIAQQNSFK.-	1234.39	2	3.58	0.51	770.1	1	17/20	1	1.56E9
	14.93 - 24.76	-.LQGIAQQNSFK.-	1234.39	1	2.88	0.44	373.0	1	13/20	1	1.29E9
	14.57 - 15.78	-.LQGIAQQNSFK.-	1234.39	2	3.45	0.50	576.5	1	14/20	1	1.95E9
	30.83 - 30.89	-.LQGIAQQNSFK.-	1234.39	1	2.37	0.39	512.9	1	13/20	1	5.19E8
	27.04 - 27.66	-.LQGIAQQNSFK.-	1234.39	1	2.44	0.35	472.3	1	12/20	1	4.25E8
	17.93 - 19.57	-.LQGIAQQNSFK.-	1234.39	2	3.78	0.62	749.3	1	16/20	1	1.87E9
	26.15 - 27.60	-.LQGIAQQNSFK.-	1234.39	2	3.32	0.58	921.6	1	17/20	1	2.04E9
	29.90 - 31.34	-.LQGIAQQNSFK.-	1234.39	2	3.49	0.62	912.2	1	17/20	1	1.25E9
	23.82 - 25.54	-.LQGIAQQNSFK.-	1234.39	2	3.65	0.48	1002.8	1	18/20	1	1.55E9
	21.89 - 23.66	-.LQGIAQQNSFK.-	1234.39	2	4.25	0.58	866.6	1	17/20	1	1.32E9
	83.02	-.MEAAGKPTDKPNLVCGPVQICWHK.-	2738.15	2	3.47	0.52	349.9	1	16/46	1	1.71E9
	81.93 - 83.00	-.MEAAGKPTDKPNLVCGPVQICWHK.-	2738.15	3	4.08	0.57	509.8	1	25/92	1	6.07E9
	91.17 - 92.29	-.NWIDKEEYPQSAAIDLR.-	2049.23	2	5.46	0.52	2033.9	1	25/32	1	1.91E10
	92.86 - 92.90	-.NWIDKEEYPQSAAIDLR.-	2049.23	2	4.25	0.58	904.8	1	17/32	1	1.83E10
	91.87 - 92.65	-.NWIDKEEYPQSAAIDLR.-	2049.23	3	5.05	0.53	1382.5	1	30/64	1	7.75E9
	114.58	-.PAGQVIAQYYEFLR.-	1655.88	1	2.95	0.40	386.0	1	15/26	1	2.44E9
	114.38	-.PAGQVIAQYYEFLR.-	1655.88	3	5.21	0.48	2422.9	1	34/52	1	2.09E9
	113.08 - 114.23	-.PAGQVIAQYYEFLR.-	1655.88	2	4.52	0.57	1135.6	1	18/26	1	2.63E10
	114.79 - 115.97	-.PAGQVIAQYYEFLR.-	1655.88	2	4.80	0.61	1703.1	1	21/26	1	2.33E10
	85.14 - 85.65	-.QNLATFCQTWDDENVHK.-	2107.22	2	3.58	0.63	492.3	1	14/32	1	5.76E9
	85.21	-.QNLATFCQTWDDENVHK.-	2107.22	3	3.37	0.52	819.1	1	25/64	1	6.89E9
	111.68 - 112.88	-.RFPLHEM*RDDVAFQIINDELYLDGNAF	3265.61	3	5.04	0.50	690.9	1	33/104	1	1.52E10
	115.13	-.RFPLHEM*RDDVAFQIINDELYLDGNAF	3265.61	3	3.25	0.27	594.2	1	29/104	1	8.86E9
	113.42 - 114.57	-.RFPLHEM*RDDVAFQIINDELYLDGNAF	3265.61	3	3.83	0.41	308.7	2	27/104	1	1.24E10
	119.75	-.RFPLHEMRDDVAFQIINDELYLDGNAR	3249.61	3	5.50	0.57	959.6	1	36/104	1	5.05E9
	121.79 - 122.37	-.RFPLHEMRDDVAFQIINDELYLDGNAR	3249.61	3	4.72	0.42	910.6	1	33/104	1	7.89E9
	126.96 - 127.53	-.RGFEM*DFAELLLEDYK.-	1993.23	2	4.75	0.62	1368.8	1	22/30	1	1.72E10
	127.13 - 127.64	-.RGFEM*DFAELLLEDYK.-	1993.23	3	3.92	0.40	1416.5	1	29/60	1	4.35E9
	76.62	-.RM*EAAGKPTDKPNLVCGPVQICWHK	2910.33	3	3.54	0.44	703.8	1	29/96	1	2.43E9
	78.93	-.RMEAAGKPTDKPNLVCGPVQICWHK.-	2894.33	3	4.85	0.53	1178.2	1	33/96	1	2.47E9
	118.27 - 119.51	-.VQNASYQVAAYLADEIAK.-	1955.16	3	4.80	0.59	1826.8	1	32/68	1	9.93E9
	117.93 - 119.15	-.VQNASYQVAAYLADEIAK.-	1955.16	2	5.93	0.59	2335.4	1	25/34	1	2.83E10
	122.44 - 123.69	-.VQNASYQVAAYLADEIAK.-	1955.16	2	4.93	0.54	2460.1	1	27/34	1	1.33E10
	119.67	-.VQNASYQVAAYLADEIAK.-	1955.16	2	5.14	0.49	1503.5	1	23/34	1	3.02E10
	120.07 - 120.91	-.VQNASYQVAAYLADEIAK.-	1955.16	3	3.70	0.52	1035.4	1	26/68	1	7.54E9
	84.24 - 85.53	-.YWDVELR.-	981.09	1	2.20	0.36	456.5	1	10/12	1	1.37E10
#3	ENO_ECOLI (P08324)					510.36			51 (51 0 0 0 0)		13.34
	110.09 - 111.18	-.AAGYELGKDITLAM*DCAASEFYKDGK	2842.12	3	3.05	0.35	515.7	2	28/100		9.79E9
	139.38	-.AAGYELGKDITLAMDCAASEFYK.-	2525.81	2	2.64	0.38	484.9	1	16/44		4.13E9
	137.45 - 138.59	-.AFTSEEFTHFLEELTK.-	1930.10	2	5.50	0.65	1489.4	1	19/30		3.51E10
	121.75 - 122.31	-.AKGM*NTAVGDEGGYAPNLGSNAEAL	3206.53	2	3.46	0.50	473.2	1	20/64		8.73E9
	121.52 - 122.90	-.AKGM*NTAVGDEGGYAPNLGSNAEAL	3206.53	3	6.56	0.66	2024.5	1	46/128		2.51E10
	123.44 - 124.16	-.AKGM*NTAVGDEGGYAPNLGSNAEAL	3206.53	3	5.78	0.58	2178.3	1	44/128		1.18E10
	101.27 - 101.47	-.AVAAVNGPIAQALIGK.-	1493.78	1	2.12	0.49	174.1	6	11/30		4.45E9

	98.81 - 99.40	-.AVAAVNGPIAQALIGK.-	1493.78	2	3.25	0.56	792.1	1	18/30	1.45E10
	97.72 - 99.21	-.AVAAVNGPIAQALIGK.-	1493.78	1	2.79	0.57	273.2	3	12/30	8.52E9
	103.19 - 104.15	-.AVAAVNGPIAQALIGK.-	1493.78	2	2.62	0.51	774.5	1	16/30	1.08E10
	103.62 - 104.32	-.AVAAVNGPIAQALIGK.-	1493.78	1	1.89	0.40	242.8	7	11/30	5.93E9
	97.10 - 98.23	-.AVAAVNGPIAQALIGK.-	1493.78	2	5.03	0.61	1437.0	1	24/30	1.47E10
	89.60	-.AVAAVNGPIAQALIGKDAK.-	1808.12	2	3.36	0.54	640.2	1	17/36	4.33E9
	12.57	-.DAGYTAVISHR.-	1190.29	2	3.34	0.57	1548.5	1	17/20	4.73E9
	83.37	-.DAKDQAGIDKIM*IDLDGTENK.-	2307.52	2	3.84	0.60	853.2	1	17/40	3.80E9
	87.64	-.DITLAM*DCAASEFYKDGK.-	2052.24	2	2.93	0.51	430.4	1	13/34	3.99E9
	109.66	-.DITLAMDCAASEFYKDGK.-	2036.24	2	3.68	0.49	1099.4	1	18/34	3.46E9
	89.46 - 90.03	-.DQAGIDKIM*IDLDGTENK.-	1993.18	2	4.32	0.59	1203.7	1	20/34	6.66E9
	104.75 - 105.62	-.DQAGIDKIMIDLDGTENK.-	1977.18	2	3.90	0.54	993.8	1	17/34	7.29E9
	105.11 - 105.83	-.FGANAILAVSLANAK.-	1460.70	2	4.44	0.48	1594.3	1	24/28	1.13E10
	105.28 - 105.98	-.FGANAILAVSLANAK.-	1460.70	1	2.96	0.49	755.9	1	17/28	6.08E9
	107.46	-.FGANAILAVSLANAK.-	1460.70	1	2.53	0.42	188.5	10	10/28	5.38E9
	116.06	-.FNQIGSLTETLAAIK.-	1606.85	1	2.86	0.47	492.3	1	14/28	5.81E9
	115.63 - 116.75	-.FNQIGSLTETLAAIK.-	1606.85	2	5.06	0.44	1002.3	1	21/28	3.65E10
	119.05 - 119.56	-.FNQIGSLTETLAAIK.-	1606.85	2	4.22	0.38	960.0	1	20/28	8.40E9
	115.70 - 116.28	-.FNQIGSLTETLAAIK.-	1606.85	1	1.93	0.21	326.2	3	11/28	1.74E10
	76.55 - 76.89	-.GIANSILIK.-	929.14	2	2.88	0.36	679.9	1	14/16	3.46E9
	128.68 - 129.67	-.GM*NTAVGDEGGYAPNLGSNAEALAV	3007.28	2	4.60	0.65	412.9	1	21/60	2.41E10
	130.24 - 130.79	-.GM*NTAVGDEGGYAPNLGSNAEALAV	3007.28	2	4.45	0.61	365.8	1	20/60	2.23E10
	130.47 - 131.60	-.GM*NTAVGDEGGYAPNLGSNAEALAV	3007.28	3	3.35	0.29	838.0	1	30/120	1.44E10
	128.79 - 129.91	-.GM*NTAVGDEGGYAPNLGSNAEALAV	3007.28	3	7.12	0.67	2283.6	1	41/120	1.87E10
	86.82 - 88.00	-.GM*PLYEHIAELNGTPGK.-	1844.08	2	4.24	0.56	1441.1	1	22/32	6.65E9
	83.70 - 84.30	-.GM*PLYEHIAELNGTPGK.-	1844.08	2	4.81	0.59	1733.3	1	23/32	1.77E10
	132.44	-.GMNTAVGDEGGYAPNLGSNAEALAVI	2991.28	2	3.51	0.60	159.7	1	16/60	8.62E9
	130.64 - 131.35	-.GMNTAVGDEGGYAPNLGSNAEALAVI	2991.28	3	4.95	0.55	1453.5	1	34/120	8.89E9
	130.81 - 131.37	-.GMNTAVGDEGGYAPNLGSNAEALAVI	2991.28	2	3.97	0.65	359.5	1	20/60	1.29E10
	94.97 - 95.88	-.GMPLYEHIAELNGTPGK.-	1828.08	2	3.00	0.49	377.4	1	14/32	9.04E9
	91.08 - 92.40	-.GMPLYEHIAELNGTPGK.-	1828.08	2	4.65	0.59	1829.1	1	21/32	1.19E10
	203.79 - 205.64	-.GNPTVEAEVHLEGGFVGM*AAAPSGA	2874.09	3	5.23	0.57	1053.1	1	39/116	4.13E8
	103.59 - 104.71	-.IQLVGDDLFVTNTK.-	1563.78	2	4.74	0.45	1536.5	1	21/26	1.57E10
	103.08 - 104.24	-.IQLVGDDLFVTNTK.-	1563.78	1	2.14	0.39	262.0	2	12/26	1.01E10
	106.11	-.IQLVGDDLFVTNTK.-	1563.78	1	2.24	0.51	547.7	1	14/26	4.45E9
	104.51	-.IQLVGDDLFVTNTK.-	1563.78	1	3.96	0.57	492.6	1	15/26	5.39E9
	122.48 - 123.67	-.QYPIVSIEDGLDESDWDGFAYQTK.-	2777.93	2	2.72	0.36	278.5	2	14/46	1.03E10
	127.34 - 127.89	-.QYPIVSIEDGLDESDWDGFAYQTK.-	2777.93	2	2.51	0.52	257.7	2	12/46	6.84E9
	103.46	-.SGETEDATIADLAVGTAAGQIK.-	2119.27	2	3.99	0.52	891.5	1	19/42	8.02E9
	107.45 - 108.36	-.SGETEDATIADLAVGTAAGQIK.-	2119.27	2	3.85	0.23	597.3	1	16/42	3.91E10
	105.72 - 106.86	-.SGETEDATIADLAVGTAAGQIK.-	2119.27	2	5.92	0.64	1873.4	1	26/42	4.88E10
	203.64 - 204.54	-.SGETEDATIADLAVGTAAGQIK.-	2119.27	2	2.85	0.43	488.3	2	14/42	4.80E8
	107.83 - 109.18	-.VLGDKIQLVGDDLFVTNTK.-	2076.38	2	6.22	0.63	1472.1	1	23/36	1.65E10
	107.85 - 108.40	-.VLGDKIQLVGDDLFVTNTK.-	2076.38	3	4.88	0.57	1806.8	1	34/72	9.67E9
#4	ATPB_ECOLI (P00824					340.28			34 (34 0 0 0 0)	4.50
	101.03 - 101.53	-.AAPSYEELSNSQELLETGIK.-	2180.36	2	5.56	0.54	1470.5	1	23/38	9.46E9
	104.02 - 104.58	-.AAPSYEELSNSQELLETGIK.-	2180.36	2	5.63	0.55	1629.8	1	23/38	1.80E10
	75.08	-.DLEHPIEVPVGK.-	1333.52	2	3.37	0.55	1076.7	1	18/22	3.05E9
	122.25	-.DVLLFVDNIYR.-	1367.58	2	3.68	0.52	1373.5	1	17/20	4.35E9
	132.98 - 133.50	-.FLSQPFFVAEVFTGSPGK.-	1959.23	2	4.76	0.66	824.4	1	22/34	2.26E10
	133.24	-.FLSQPFFVAEVFTGSPGK.-	1959.23	3	4.56	0.50	1623.0	1	30/68	3.24E9
	113.93	-.FRDEGRDVLLFVDNIYR.-	2128.38	3	4.77	0.62	1389.1	1	31/64	3.65E9
	113.69 - 114.47	-.FRDEGRDVLLFVDNIYR.-	2128.38	2	2.51	0.29	204.9	16	11/32	5.84E9
	124.57 - 125.44	-.GIM*EGEYDHLPEQAFYM*VGSIEEAVE	3105.40	2	2.68	0.39	235.0	1	14/52	8.99E9
	124.46 - 125.26	-.GIM*EGEYDHLPEQAFYM*VGSIEEAVE	3105.40	3	5.59	0.63	979.6	1	30/104	2.08E10
	145.70 - 145.85	-.GIM*EGEYDHLPEQAFYMVGSIEEAVEI	3089.40	3	3.59	0.43	805.9	1	28/104	7.03E9
	92.17 - 93.43	-.GLDVKDLEHPIEVPVGK.-	1846.12	3	3.75	0.42	1208.2	1	29/64	5.51E9
	92.13 - 93.26	-.GLDVKDLEHPIEVPVGK.-	1846.12	2	4.29	0.52	1328.6	1	21/32	7.78E9
	51.80 - 54.32	-.GVQSILQR.-	901.05	1	1.88	0.27	122.3	1	8/14	1.98E9
	75.92 - 76.49	-.IM*NVLGEPVDM*KGEIGEEER.-	2278.55	2	4.55	0.63	348.6	1	16/38	4.51E9
	84.68	-.IMNVLGEPVDM*KGEIGEEER.-	2262.55	2	3.76	0.44	446.7	1	16/38	2.05E9
	108.42 - 108.70	-.M*PSAVGYQPTLAEEMGVLQER.-	2323.63	2	4.95	0.69	550.3	1	20/40	5.28E9
	96.98	-.MPSAVGYQPTLAEEM*GVLQER.-	2323.63	2	4.59	0.62	586.1	1	19/40	5.68E9
	90.57 - 91.13	-.NIAIEHSGYSVFAGVGER.-	1907.08	2	5.19	0.66	1618.5	1	23/34	1.02E10
	90.70	-.NIAIEHSGYSVFAGVGER.-	1907.08	3	4.14	0.60	1482.8	1	31/68	3.26E9
	108.53 - 109.87	-.QIASLGIYPAVDPLDSTSR.-	2004.23	2	3.20	0.58	480.6	1	16/36	1.63E10
	79.60 - 80.17	-.QLDPLVVGQEHYDTAR.-	1842.00	2	3.51	0.53	369.5	1	13/30	5.54E9
	64.13 - 65.34	-.VALTGLTM*AEK.-	1150.37	2	2.71	0.40	890.8	1	15/20	3.32E9
	72.87	-.VGLFGGAGVGK.-	962.13	2	3.04	0.41	1091.2	1	18/20	1.72E9
	62.48 - 63.01	-.VSLVYGQM*NEPPGNR.-	1677.86	2	3.12	0.47	134.8	73	8/28	1.53E9
	59.66 - 60.24	-.VYDALEVQNGNER.-	1507.59	1	2.81	0.45	280.0	1	12/24	1.77E9
	59.25 - 60.34	-.VYDALEVQNGNER.-	1507.59	2	4.46	0.50	1993.2	1	20/24	3.66E9
	60.94 - 62.09	-.VYDALEVQNGNER.-	1507.59	2	4.30	0.55	2069.5	1	21/24	4.63E9
	135.55 - 136.32	-.YQELKDIIAILGM*DELSEEDKLVVAR.-	3008.43	2	2.84	0.20	155.0	14	13/50	3.88E9

	108.63 - 109.16	-.YTLAGTEVSALLGR.-	1451.65	2	4.82	0.23	1857.2	1	21/26	9.62E9
	37.11 - 37.77	-.YVSLKDTIR.-	1095.27	2	2.99	0.40	624.0	1	13/16	4.34E8
	38.64 - 40.02	-.YVSLKDTIR.-	1095.27	2	2.57	0.30	718.4	1	13/16	4.34E8
	40.63	-.YVSLKDTIR.-	1095.27	2	2.66	0.39	516.4	1	12/16	4.61E8
	35.53 - 36.58	-.YVSLKDTIR.-	1095.27	2	2.90	0.36	638.1	2	12/16	3.40E8
#5	PGK_ECO57 (Q8XD03					300.31			30 (30 0 0 0 0)	5.88
	144.71 - 145.48	-.ADEQILDIGDASAQELAEILK.-	2243.45	2	6.07	0.63	1306.9	1	25/40	1.25E10
	143.30 - 144.17	-.ADEQILDIGDASAQELAEILK.-	2243.45	2	4.77	0.62	490.3	1	16/40	1.42E10
	11.67	-.ADLNVPVKDGK.-	1156.31	2	2.82	0.45	441.1	1	13/20	3.03E9
	12.85 - 13.47	-.ALKEPARPM*VAIVGGSK.-	1741.09	2	3.88	0.55	231.7	1	16/32	2.38E9
	65.09	-.ALKEPARPMVAIVGGSK.-	1725.09	2	4.06	0.51	816.2	1	17/32	1.99E9
	134.10 - 135.23	-.FADVACAGPLLAAELDALGK.-	2003.28	2	5.13	0.68	1050.7	1	23/38	2.37E10
	134.59 - 134.66	-.FADVACAGPLLAAELDALGK.-	2003.28	3	6.29	0.59	3251.3	1	40/76	5.74E9
	112.99 - 114.40	-.IADQLIVGGGIANTFIAAQGHDVGK.-	2466.78	3	3.73	0.53	795.0	1	31/96	9.66E9
	129.29 - 129.95	-.ISYISTGGGAFLEFVEGK.-	1876.10	2	4.40	0.63	1508.3	1	21/34	9.85E9
	103.76	-.KYAALCDVFVM*DAFGTAHR.-	2189.47	2	3.47	0.52	246.6	2	13/36	3.78E9
	88.89 - 89.44	-.LLTTCNIPVPSDVR.-	1585.82	1	2.52	0.33	177.1	2	11/26	4.39E9
	88.77 - 89.31	-.LLTTCNIPVPSDVR.-	1585.82	2	4.09	0.52	1032.2	1	20/26	2.08E10
	134.06	-.LVKDYLDGVDVAEGELVVLENVR.-	2545.87	3	3.23	0.35	960.9	1	28/88	8.03E9
	82.72 - 83.27	-.RLLTTCNIPVPSDVR.-	1742.01	2	3.04	0.43	333.2	1	16/28	3.11E9
	75.38	-.SLYEADLVDEAKR.-	1509.64	1	2.54	0.43	549.7	1	14/24	3.37E9
	75.23 - 75.80	-.SLYEADLVDEAKR.-	1509.64	2	3.78	0.58	1826.4	1	19/24	9.58E9
	147.09 - 148.24	-.SVNDVKADEQILDIGDASAQELAEILK.-	2886.16	2	2.55	0.42	289.8	1	13/52	9.45E9
	150.84 - 151.40	-.SVNDVKADEQILDIGDASAQELAEILK.-	2886.16	3	5.70	0.47	1777.2	1	37/104	1.12E10
	151.53	-.SVNDVKADEQILDIGDASAQELAEILK.-	2886.16	2	2.87	0.40	402.3	1	16/52	7.02E9
	145.42	-.SVNDVKADEQILDIGDASAQELAEILK.-	2886.16	2	4.79	0.57	398.4	1	14/52	8.24E9
	143.82 - 144.38	-.TILWNGPVGVFEFPNFR.-	1994.28	2	5.46	0.68	1736.4	1	21/32	8.37E9
	141.28 - 142.48	-.TILWNGPVGVFEFPNFR.-	1994.28	2	4.99	0.70	1726.0	1	21/32	1.43E10
	130.39	-.TILWNGPVGVFEFPNFRK.-	2122.46	2	2.57	0.41	824.1	1	19/34	4.57E9
	72.40 - 72.98	-.VATEFSETAPATLK.-	1465.63	1	2.11	0.30	244.5	1	11/26	1.04E10
	72.49	-.VATEFSETAPATLK.-	1465.63	1	2.88	0.47	348.1	1	12/26	6.88E9
	72.38 - 72.97	-.VATEFSETAPATLK.-	1465.63	2	3.89	0.64	1092.2	1	20/26	1.54E10
	85.76 - 87.03	-.VLPAVAM*LEER.-	1244.49	2	2.85	0.53	701.3	1	17/20	9.15E9
	106.09	-.VLPAVAMLEER.-	1228.49	2	2.72	0.51	858.0	1	16/20	4.38E9
	121.41 - 122.63	-.VM*VTSHLGRPTEGEYNEEFSLLPVVN	3339.76	3	5.81	0.62	1440.3	1	37/112	1.82E10
	112.35	-.YAALCDVFVM*DAFGTAHR.-	2061.30	2	3.07	0.55	497.8	1	13/34	6.54E9
#6	ATPA_ECOLI (P00822					280.25			28 (28 0 0 0 0)	3.34
	51.88 - 52.97	-.AVDSM*IPIGR.-	1075.27	2	3.11	0.53	1056.4	1	15/18	1.13E9
	50.07 - 51.24	-.AVDSM*IPIGR.-	1075.27	2	2.70	0.50	1077.4	1	15/18	1.36E9
	101.94 - 102.44	-.DRGEDALIIYDDLSK.-	1723.86	2	4.83	0.48	1647.7	1	19/28	1.28E10
	92.09 - 92.74	-.DSVGAVVM*GPYADLAEGM*K.-	1943.19	2	4.98	0.63	1184.9	1	22/36	6.09E9
	103.96 - 104.66	-.DSVGAVVM*GPYADLAEGMK.-	1927.19	2	2.81	0.10	395.5	1	14/36	8.20E9
	122.40	-.ELAAFSQFASDLDDATR.-	1857.96	2	4.68	0.62	1472.6	1	20/32	9.18E9
	113.91 - 114.45	-.ELAAFSQFASDLDDATRK.-	1986.13	2	4.37	0.63	744.0	1	16/34	6.63E9
	114.76	-.GPLDHDGFSAVEAIAPGVIER.-	2151.37	2	4.29	0.45	1265.9	1	21/40	4.51E9
	111.10	-.GPLDHDGFSAVEAIAPGVIER.-	2151.37	2	4.52	0.40	1529.5	1	22/40	5.98E9
	78.77 - 80.64	-.GYLADVELSK.-	1095.23	1	1.89	0.16	585.9	1	11/18	1.10E10
	79.00	-.GYLADVELSK.-	1095.23	1	3.12	0.29	944.0	1	13/18	3.33E9
	78.95	-.GYLADVELSK.-	1095.23	2	3.09	0.45	990.8	1	15/18	4.13E9
	104.35	-.IAQFNVVSEAHNEGTIVSVSDGVIR.-	2642.91	2	3.83	0.59	583.7	1	17/48	5.86E9
	76.66	-.IHGLADCM*QGEM*ISLPGNR.-	2132.40	2	3.74	0.64	714.9	1	17/36	2.34E9
	83.97	-.IHGLADCM*QGEMISLPGNR.-	2116.40	2	4.10	0.20	555.5	1	18/36	3.09E9
	95.90 - 95.95	-.M*QLNSTEISELIK.-	1522.75	1	2.95	0.37	194.3	1	14/24	2.70E9
	95.74 - 96.35	-.M*QLNSTEISELIK.-	1522.75	2	3.92	0.19	1939.0	1	21/24	6.78E9
	112.22 - 112.91	-.QYAPM*SVAQQSLVLFAAER.-	2126.42	2	4.88	0.60	426.0	1	15/36	7.51E9
	99.72 - 99.76	-.TALAIDAIINQR.-	1299.50	1	1.95	0.33	159.5	6	10/22	4.73E9
	99.74	-.TALAIDAIINQR.-	1299.50	2	3.95	0.40	1906.0	1	18/22	3.19E9
	82.01	-.VNAEYVEAFTK.-	1271.40	1	1.94	0.40	577.6	1	13/20	2.57E9
	82.10	-.VNAEYVEAFTK.-	1271.40	2	3.54	0.58	1152.2	1	17/20	2.57E9
	86.84 - 87.41	-.VNAEYVEAFTKGEVK.-	1684.87	2	4.31	0.56	1491.3	1	19/28	4.76E9
	115.19 - 115.76	-.VVNTLGAPIDGKGPLDHDGFSAVEAIAI	3316.71	2	3.39	0.53	182.2	1	14/64	3.09E9
	116.55 - 117.31	-.VVNTLGAPIDGKGPLDHDGFSAVEAIAI	3316.71	3	3.64	0.39	624.8	3	27/128	1.27E10
	117.99 - 118.59	-.VVNTLGAPIDGKGPLDHDGFSAVEAIAI	3316.71	3	4.71	0.56	924.4	1	35/128	6.59E9
	115.02 - 115.59	-.VVNTLGAPIDGKGPLDHDGFSAVEAIAI	3316.71	3	4.56	0.55	995.0	1	34/128	7.86E9
	85.37 - 86.61	-.YAIALNLER.-	1063.23	2	2.78	0.34	1102.3	1	14/16	2.68E9
#7	TIG_ECOLI (P22257) T					130.31			13 (13 0 0 0 0)	1.18
	135.57 - 136.00	-.ANDIDVPAALIDSEIDVLR.-	2040.26	2	3.26	0.49	364.4	1	17/36	5.44E9
	60.17 - 61.04	-.ASDFVLAM*GQGR.-	1268.43	2	2.99	0.52	703.0	1	15/22	2.05E9
	67.76 - 69.00	-.EKINPAGAPTYVPGEYK.-	1835.05	2	3.41	0.55	489.8	1	14/32	5.33E9
	106.79 - 107.01	-.ELPELTAEFIK.-	1290.49	1	2.29	0.37	187.9	9	10/20	4.58E9
	78.33	-.FGVEDGSVEGLR.-	1265.36	2	3.57	0.50	983.1	1	16/22	3.87E9
	73.06 - 73.44	-.INPAGAPTYVPGEYK.-	1577.76	2	3.38	0.53	764.9	1	20/28	8.27E9
	73.23	-.INPAGAPTYVPGEYK.-	1577.76	1	1.98	0.22	239.6	6	11/28	2.52E9
	102.48	-.NFIDAIIK.-	934.11	1	2.00	0.17	599.6	2	11/14	6.66E9

	97.29	-.NFIDAIIKEK.-	1191.40	2	2.56	0.35	564.7	1	12/18	2.58E9
	110.71	-.NVALEEQAVEAVLAK.-	1584.80	2	6.30	0.66	2794.4	1	23/28	6.61E9
	110.77	-.NVALEEQAVEAVLAK.-	1584.80	1	1.82	0.19	333.2	7	10/28	3.59E9
	29.03 - 30.40	-.VKSQAIEGLVK.-	1172.40	2	2.78	0.37	646.6	1	14/20	5.14E8
	68.51	-.VTEKETTFNELM*NQQA.-	1900.06	2	3.79	0.42	727.2	1	14/30	2.42E9
#8	GLYA_ECO57 (Q8XA5					120.27			12 (12 0 0 0 0)	1.67
	14.23	-.GGLILAK.-	671.85	1	2.06	0.13	451.4	1	9/12	1.78E9
	87.89 - 88.67	-.KLNSAVFPGGQGGPLM*HVIAGK.-	2195.57	2	3.26	0.45	224.4	3	14/42	5.73E9
	87.96 - 89.16	-.KLNSAVFPGGQGGPLM*HVIAGK.-	2195.57	3	5.43	0.58	1808.0	1	35/84	4.81E9
	113.21 - 114.06	-.LYNIVPYGIDATGHIDYADLEK.-	2481.74	2	3.03	0.49	537.2	1	22/42	1.11E10
	115.47 - 116.08	-.M*IIGGFSAYSGVVDWAK.-	1818.09	2	5.05	0.57	1926.5	1	24/32	8.66E9
	74.22 - 74.81	-.VRQEEHIELIASENYTSPR.-	2272.46	2	4.71	0.60	1233.7	1	20/36	4.27E9
	205.16	-.VRQEEHIELIASENYTSPR.-	2272.46	2	2.51	0.39	285.0	4	12/36	9.38E7
	109.81	-.VVSGGTDNHLFLVDLVDK.-	1929.16	2	5.02	0.55	1638.6	1	21/34	5.65E9
	122.67 - 123.25	-.VVSGGTDNHLFLVDLVDKNLTGK.-	2442.75	3	4.03	0.50	852.9	1	33/88	5.90E9
	120.01 - 120.58	-.VVSGGTDNHLFLVDLVDKNLTGK.-	2442.75	3	4.22	0.48	625.3	1	30/88	1.07E10
	133.35 - 134.28	-.YYGGCEYVDIVEQLAIDR.-	2164.35	2	3.50	0.56	650.1	1	15/34	9.22E9
	131.29 - 132.36	-.YYGGCEYVDIVEQLAIDR.-	2164.35	2	5.25	0.51	2024.9	1	23/34	8.84E9
#9	DLDH_ECOLI (P00391					120.26			12 (12 0 0 0 0)	1.57
	68.62 - 69.26	-.AGVEVDDRGFIR.-	1334.46	2	2.51	0.18	708.2	4	13/22	3.23E9
	72.17 - 72.89	-.ALAEHGIVFGEPK.-	1368.56	2	3.09	0.57	1077.1	1	17/24	2.68E9
	95.29	-.EKGISYETATFPWAASGR.-	1972.15	2	2.75	0.39	1091.7	1	18/34	5.19E9
	92.42	-.IWDSTDALELK.-	1291.43	2	2.91	0.20	815.5	1	18/20	3.37E9
	84.74	-.KAPAEPQRYDAVLVAIGR.-	1955.25	2	3.22	0.40	468.0	1	15/34	3.84E9
	105.53	-.TNVPHIFAIGDIVGQPM*LAHK.-	2275.66	2	4.33	0.59	1186.1	1	20/40	5.79E9
	98.71 - 99.29	-.TQVVVLGAGPAGYSAAFR.-	1765.01	2	5.11	0.60	1505.9	1	23/34	8.72E9
	98.98	-.TQVVVLGAGPAGYSAAFR.-	1765.01	1	2.23	0.47	191.1	1	17/34	2.75E9
	69.89 - 70.51	-.VINQLTGGLAGM*AK.-	1389.65	2	3.26	0.57	738.8	1	16/26	2.77E9
	124.82 - 125.40	-.VIPSIAYTEPEVAWVGLTEK.-	2203.52	2	4.80	0.59	1252.9	1	23/38	2.15E10
	80.52	-.VTAVEAKEDGIYVTMEGK.-	1941.19	2	4.14	0.52	1061.9	1	17/34	2.69E9
	97.34 - 97.89	-.YNTLGGVCLNVGCIPSK.-	1853.10	2	4.23	0.61	1295.7	1	20/32	9.50E9
#10	ADHE_ECOLI (P17547					110.28			11 (11 0 0 0 0)	1.24
	84.87	-.AAYSSGKPAIGVGAGNTPVVIDETADIK	2859.19	3	5.39	0.59	1163.8	1	37/112	5.04E9
	85.08	-.AAYSSGKPAIGVGAGNTPVVIDETADIK	2859.19	2	4.73	0.60	687.2	1	21/56	2.23E9
	126.30	-.EAGVQEADFLANVDKLSEDAFDDQCT	3384.52	3	5.65	0.61	1233.2	1	32/120	5.74E9
	56.18 - 57.48	-.EYASFTQEQVDK.-	1445.51	2	3.02	0.52	728.8	1	14/22	1.40E9
	59.33 - 60.66	-.FATHGGYLLQGK.-	1292.47	2	2.92	0.37	912.0	1	17/22	1.73E9
	102.93	-.ILIGEVTVVDESEPFAHEK.-	2113.35	3	3.40	0.36	734.1	1	32/72	5.34E9
	102.95	-.ILIGEVTVVDESEPFAHEK.-	2113.35	2	3.02	0.48	673.3	1	16/36	4.20E9
	109.04 - 109.94	-.ILINTPASQGGIGDLYNFK.-	2022.29	2	4.64	0.65	513.5	1	17/36	1.39E10
	110.49	-.ILINTPASQGGIGDLYNFK.-	2022.29	2	4.96	0.66	660.5	1	20/36	1.07E10
	49.06	-.NAIIFSPHPR.-	1152.33	2	3.00	0.56	1114.8	1	15/18	4.77E8
	81.78 - 82.33	-.YAEIADHLGLSAPGDR.-	1685.82	2	4.40	0.50	1076.6	1	20/30	6.09E9
#11	PURA_ECOLI (P12283					100.30			10 (10 0 0 0 0)	1.08
	101.36	-.AVQLNSLSGFCLTK.-	1538.76	1	2.20	0.49	269.3	1	12/26	1.87E9
	110.73	-.ENVTSIIGNGVVLSPAALM*K.-	2030.38	2	5.97	0.72	1761.5	1	24/38	6.38E9
	114.70	-.ENVTSIIGNGVVLSPAALM*K.-	2030.38	2	3.04	0.51	700.3	1	15/38	4.84E9
	103.91	-.GVEPIYETM*PGWSESTFGVK.-	2231.47	2	3.08	0.49	316.1	1	14/38	3.31E9
	86.37	-.LKEVM*EYHNFQLVNYYK.-	2235.55	3	3.23	0.41	1102.9	1	26/64	3.71E9
	86.35	-.LKEVM*EYHNFQLVNYYK.-	2235.55	2	4.00	0.55	969.2	1	18/32	2.71E9
	124.20 - 125.05	-.LLLSEACPLILDYHVALDNAR.-	2397.75	3	3.72	0.35	1047.1	1	30/80	8.85E9
	106.02	-.RIEELTGVPIDIISTGPDR.-	2082.34	2	3.08	0.53	464.9	1	17/36	3.25E9
	135.79 - 136.35	-.VGAGPFPTELFDETGEFLCK.-	2215.44	2	4.47	0.67	843.5	1	21/38	1.01E10
	79.98	-.VGDLFDKETFAEK.-	1499.65	2	2.66	0.24	737.3	1	15/24	4.69E9
#12	Q8XEB4 (Q8XEB4) For					100.26			10 (10 0 0 0 0)	0.79
	118.71 - 119.81	-.DAIPTQSVLTITSNVVYGK.-	2007.27	2	3.27	0.62	584.1	1	17/36	6.08E9
	91.83	-.EM*LLDAM*ENPEKYPQLTIR.-	2324.66	2	2.56	0.43	130.3	3	11/36	3.99E9
	90.24 - 91.15	-.ITEQEAQEM*VDHLVM*K.-	1934.18	2	3.04	0.50	300.0	2	14/30	5.11E9
	131.97	-.NYTPYEGDESFLAGATEATTTLWDK.-	2781.92	2	3.41	0.58	248.0	1	12/48	5.20E9
	83.60	-.SEPIKGDLLNYDEVM*ER.-	2025.23	2	4.01	0.55	955.7	1	18/32	2.83E9
	103.72 - 103.74	-.THNQGVFDVYTPDILR.-	1876.06	2	5.18	0.63	1737.3	1	21/30	5.18E9
	131.09 - 131.26	-.TPEYDELFSGDPIWATESIGGM*GLDG	2931.14	2	3.25	0.41	330.7	1	16/52	5.80E9
	42.27 - 43.54	-.VVGLQTEAPLKR.-	1311.56	2	2.97	0.49	773.1	1	15/22	6.93E8
	40.30 - 41.62	-.VVGLQTEAPLKR.-	1311.56	2	2.75	0.36	717.3	1	14/22	5.82E8
	44.08 - 45.25	-.VVGLQTEAPLKR.-	1311.56	2	2.53	0.41	560.4	1	13/22	6.92E8
#13	Q7DBF8 (Q7DBF8) Glu					100.26			10 (10 0 0 0 0)	0.93
	99.31	-.AASEEYNWDLNYGEIAK.-	1974.07	2	4.86	0.59	1014.5	1	18/32	6.55E9
	86.24	-.AAVLPANLIQAQR.-	1365.61	2	3.36	0.57	648.5	1	16/24	4.19E9
	86.39	-.AAVLPANLIQAQR.-	1365.61	1	1.81	0.30	83.3	283	8/24	2.12E9
	100.48 - 101.14	-.EAYELVAPILTK.-	1347.58	2	3.43	0.46	1165.0	1	17/22	4.85E9
	108.47	-.ELSAEGFNFIGTGVSGGEEGALKGPSII	3239.56	3	4.66	0.59	1014.4	1	33/128	4.48E9
	86.14 - 86.69	-.IAAVAEDGEPCVTYIGADGAGHYVK.-	2564.78	3	4.76	0.58	954.8	1	32/96	5.22E9
	125.14	-.KDEDGNYLVDVILDEAANK.-	2122.28	2	5.18	0.57	913.9	1	18/36	5.59E9
	66.84	-.QIADDYQQALR.-	1321.42	2	2.50	0.45	527.5	1	14/20	1.96E9

	64.77 - 66.03	-.VLSGPQAQPAGDKAEFIEK.-	1986.22	2	4.47	0.58	928.9	1	25/36	4.43E9
	65.11 - 65.65	-.VLSGPQAQPAGDKAEFIEK.-	1986.22	3	3.26	0.50	657.2	1	32/72	3.25E9
#14	ACKA_ECOLI (P15046					100.24			10 (10 0 0 0 0)	1.17
	108.72 - 109.20	-.DAASFAPLHNPAHLIGIEEALK.-	2315.61	2	4.10	0.57	602.5	1	16/42	4.51E9
	108.57 - 109.14	-.DAASFAPLHNPAHLIGIEEALK.-	2315.61	3	4.42	0.59	1368.3	1	35/84	4.72E9
	96.78	-.EGTRPAVVIPTNEELVIAQDASR.-	2466.73	2	3.70	0.52	406.7	1	19/44	7.48E9
	96.76 - 97.32	-.EGTRPAVVIPTNEELVIAQDASR.-	2466.73	3	4.14	0.49	1376.4	1	30/88	5.55E9
	144.41 - 144.94	-.FAIIDAVNGEEYLSGLAECFHLPEAR.-	2923.22	3	4.71	0.40	1053.2	1	37/100	6.59E9
	144.68	-.FAIIDAVNGEEYLSGLAECFHLPEAR.-	2923.22	2	2.74	0.55	580.4	1	17/50	4.37E9
	98.04	-.LDAVVFTGGIGENAAM*VR.-	1837.09	2	3.00	0.51	584.0	1	16/34	5.63E9
	89.37	-.LGVLGFEVDHER.-	1371.52	2	2.79	0.39	914.4	1	16/22	2.80E9
	128.55 - 129.23	-.NVAVFDTAFHQTM*PEESYLYALPYNL	3342.72	3	4.23	0.51	658.2	1	29/108	5.64E9
	100.14	-.YTSSVVIDESVIQGIK.-	1738.96	2	2.55	0.56	483.2	2	13/30	6.33E9
#15	EFG_ECOLI (P02996)					80.28			8 (8 0 0 0 0)	0.80
	108.49	-.AKPVLLEPIM*KVEVETPEENTGDVIGD	3296.74	3	5.59	0.58	1473.5	1	38/116	3.88E9
	100.67 - 101.21	-.DVTTGDTLCDPDAPIILER.-	2102.28	2	3.85	0.61	1320.0	1	21/36	4.38E9
	75.82 - 76.42	-.EFNVEANVGKPQVAYR.-	1822.01	2	3.42	0.48	941.6	1	19/30	3.31E9
	83.47	-.GQYGHVVIDM*YPLEPGSNPK.-	2218.48	2	4.21	0.63	692.1	1	17/38	2.76E9
	92.59 - 93.60	-.HASDDEPFSALAFK.-	1535.64	2	3.80	0.59	1389.5	1	20/26	5.22E9
	124.20 - 125.05	-.IATDPFVGNLTFFR.-	1598.83	2	3.51	0.50	846.1	1	16/26	8.85E9
	105.41	-.M*EFPEPVISIAVEPK.-	1702.99	2	4.11	0.65	720.8	1	19/28	4.31E9
	93.69	-.YDEAPSNVAQAVIEAR.-	1733.86	2	3.56	0.57	624.3	1	18/30	4.16E9
#16	Q8X7N8 (Q8X7N8) Am					70.25			7 (7 0 0 0 0)	0.77
	100.89 - 101.64	-.EAFATIAVAADKVDALK.-	1733.99	2	3.31	0.40	553.1	1	14/32	1.09E10
	76.21	-.FLAGHAEELDLR.-	1371.52	2	2.75	0.46	742.7	1	16/22	2.12E9
	119.00	-.SLIDSGKDYVVSM*LDSLGK.-	2044.31	2	3.78	0.50	527.0	1	17/36	5.81E9
	143.68	-.SLIDSGKDYVVSMLDSLGK.-	2028.31	2	4.98	0.63	1156.6	1	19/36	4.01E9
	105.09	-.SLVNTYQEILKNELAEKEK.-	2250.54	2	3.01	0.45	198.9	5	11/36	5.07E9
	105.07	-.SLVNTYQEILKNELAEKEK.-	2250.54	3	3.23	0.57	525.3	1	26/72	3.39E9
	107.31	-.TPNIQIIHAGLECGLFK.-	1912.22	2	4.73	0.58	1061.2	1	20/32	3.97E9
#17	Q8X722 (Q8X722) Isoc					70.23			7 (7 0 0 0 0)	0.87
	81.59 - 81.63	-.AAIEYAIANDRDSVTLVHK.-	2087.32	2	4.69	0.61	990.2	1	20/36	5.58E9
	102.22	-.FTEGAFKDWGYELAR.-	1790.96	2	2.75	0.43	560.7	1	14/28	4.90E9
	88.56 - 89.12	-.HM*GWTEAADLIVK.-	1487.71	2	3.38	0.50	1061.9	1	18/24	3.89E9
	60.75 - 61.30	-.IRFPEHCGIGIKPCSEEGTKR.-	2472.77	3	3.79	0.53	661.2	1	25/80	1.38E9
	83.23	-.KISWM*EIYTGEK.-	1501.73	2	2.94	0.36	942.1	1	16/22	3.46E9
	135.77 - 136.37	-.STQVYGQDVWLPAETLDLIR.-	2305.57	2	3.79	0.59	669.2	1	18/38	1.76E10
	77.66	-.VAIKGPLTTPVGGGIR.-	1536.84	2	2.63	0.53	458.4	1	17/30	3.10E9
#18	METK_ECOLI (P04384					60.31			6 (6 0 0 0 0)	0.51
	145.08 - 145.67	-.EFFDLRPYGLIQM*LDLLHPIYK.-	2739.23	3	6.19	0.61	2191.2	1	36/84	4.99E9
	77.93	-.FFINPTGR.-	952.09	2	2.66	0.40	763.0	1	12/14	1.12E9
	81.22 - 81.76	-.FVIGGPM*GDCGLTGR.-	1553.76	2	2.79	0.48	535.6	1	15/28	3.78E9
	138.75	-.SLQEAVM*EEIIKPILPAEWLTSATK.-	2815.28	2	3.22	0.46	259.7	1	14/48	5.64E9
	107.89	-.SQVTFQYDDGKIVGIDAVVLSTQHSEE	3451.74	3	4.78	0.55	1154.4	1	35/120	4.42E9
	92.48	-.VPSEQLTLLVR.-	1255.49	2	2.97	0.44	1311.7	1	17/20	3.49E9
#19	SERA_ECOLI (P08328					60.26			6 (6 0 0 0 0)	0.33
	45.15 - 46.55	-.AAGYTNIEFHK.-	1251.37	2	2.59	0.43	1073.1	1	15/20	6.34E8
	64.88 - 66.22	-.GALDDEQLKESIR.-	1474.60	2	3.93	0.37	1294.0	1	16/24	3.13E9
	54.08 - 55.06	-.SRTHLTEDVINAAEK.-	1684.83	2	4.12	0.51	1367.5	1	18/28	8.85E8
	67.49	-.THLTEDVINAAEK.-	1441.57	1	2.60	0.45	666.8	1	14/24	1.76E9
	67.40 - 68.10	-.THLTEDVINAAEK.-	1441.57	2	3.50	0.65	1505.4	1	20/24	2.75E9
	110.30 - 110.82	-.YSDNGSTLSAVNFPEVSLPLHGGR.-	2518.72	3	5.16	0.62	1710.5	1	37/92	5.98E9
#20	Q7DBF5 (Q7DBF5) GD					50.29			5 (5 0 0 0 0)	0.62
	122.86	-.FYQASTSELYGLVQEIPQKESTPFYPR.	3180.51	2	3.20	0.54	290.1	1	16/52	4.35E9
	122.81 - 123.34	-.FYQASTSELYGLVQEIPQKESTPFYPR.	3180.51	3	5.85	0.67	1339.6	1	36/104	8.88E9
	132.38 - 132.47	-.VALITGVTGQDGSYLAEFLLDKGYEVH	3195.61	3	3.84	0.38	439.9	1	26/116	4.92E9
	95.20	-.YFRPAEVDTLLGDPSK.-	1809.01	3	3.53	0.44	891.0	1	27/60	4.19E9
	94.99 - 96.01	-.YFRPAEVDTLLGDPSK.-	1809.01	2	4.75	0.44	651.4	1	19/30	6.34E9
#21	RS1_ECOLI (P02349)					50.27			5 (5 0 0 0 0)	0.40
	93.03	-.AVIESENSAERDQLLENLQEGM*EVK.-	2849.08	2	2.68	0.41	107.0	8	11/48	2.65E9
	119.45	-.GVVVAIDKDVVLVDAGLKSESAIPAEQF	2999.45	3	5.32	0.55	1470.4	1	38/112	3.29E9
	142.28	-.M*TESFAQLFEESLKEIETRPGSIVR.-	2915.27	3	3.36	0.57	1067.0	1	32/96	4.26E9
	95.76	-.QLGEDPWVAIAK.-	1327.51	2	2.64	0.30	464.1	2	14/22	2.69E9
	74.93 - 76.32	-.VKHPSEIVNVGDEITVK.-	1865.12	2	4.03	0.54	951.0	1	20/32	5.28E9
#22	Q8XDF3 (Q8XDF3) As					50.22			5 (5 0 0 0 0)	0.22
	20.28 - 21.80	-.KAEQYLLENETTK.-	1567.72	2	4.31	0.58	1711.3	1	18/24	2.66E8
	17.56 - 18.78	-.KAEQYLLENETTK.-	1567.72	2	4.40	0.62	1734.5	1	18/24	3.66E8
	23.95	-.KAEQYLLENETTK.-	1567.72	2	4.07	0.55	2005.6	1	20/24	1.67E8
	154.61 - 154.95	-.M*FENITAAPADPILGLADLFR.-	2292.64	2	3.52	0.49	467.1	1	19/40	6.24E9
	83.31	-.SVFNSADLEVR.-	1237.34	2	2.88	0.54	755.8	1	15/20	2.94E9
#23	SYY_ECOLI (P00951)					50.21			5 (5 0 0 0 0)	0.48
	68.24	-.AQYVLAEQVTR.-	1278.44	2	3.58	0.57	1745.7	1	17/20	2.00E9
	93.11	-.FQQAGHKPVALVGGATGLIGDPSFK.-	2496.85	2	4.14	0.59	650.1	1	20/48	3.10E9
	118.70	-.FYQFWINTADADVYR.-	1910.08	2	2.88	0.52	710.5	1	15/28	5.34E9

	141.16	-.LAQGPIALYCGFDPTADSLHLGHLVPLI	3391.93	3	3.42	0.57	597.2	1	30/120	6.75E9
	101.55 - 102.10	-.RLHQNQVFGLTVPLITK.-	1965.33	2	2.84	0.04	222.4	4	12/32	4.70E9
#24	DEGP_ECOLI (P0937€					40.25			4 (4 0 0 0 0)	0.24
	50.28 - 51.66	-.NLTSQM*VEYGQVK.-	1513.70	2	2.82	0.49	593.8	1	13/24	9.92E8
	85.84	-.SDIALIQIQNPK.-	1340.55	1	1.86	0.29	532.3	1	12/22	3.18E9
	103.03	-.SGLNAENYENFIQTDAAINR.-	2241.36	2	5.06	0.62	960.4	1	17/38	4.11E9
	84.91 - 85.04	-.VLDSKPSVLALNIQR.-	1653.95	2	2.56	0.49	379.7	1	15/28	2.71E9
#25	DCEB_ECOLI (P28302					40.23			4 (4 0 0 0 0)	1.12
	87.94 - 89.27	-.LKDGEDPGYTLYDLSER.-	1972.10	2	4.59	0.52	681.7	1	22/32	2.34E10
	89.48 - 89.99	-.LKDGEDPGYTLYDLSER.-	1972.10	3	3.71	0.37	1545.9	1	31/64	8.59E9
	89.88	-.LKDGEDPGYTLYDLSER.-	1972.10	2	4.41	0.61	829.1	1	23/32	8.96E9
	87.60 - 88.75	-.LKDGEDPGYTLYDLSER.-	1972.10	3	4.01	0.46	1435.2	1	31/64	1.06E10
#26	CARA_ECOLI (P00907					40.21			4 (4 0 0 0 0)	0.40
	101.35	-.DLPLIASNFR.-	1146.32	2	2.68	0.48	1234.4	1	15/18	2.30E9
	90.19 - 91.54	-.GAQNGCIIAGDNPDAALALEK.-	2099.28	2	4.21	0.48	426.3	1	17/40	6.00E9
	93.09	-.LTIVPAQTSAEDVLK.-	1585.82	2	3.25	0.63	407.6	1	18/28	5.16E9
	112.93 - 113.05	-.M*NPDGIFLSNGPGDPAPCDYAITAIQK	2880.17	2	2.54	0.39	447.1	1	17/52	4.70E9
#27	CLPX_ECOLI (P33138					40.20			4 (4 0 0 0 0)	0.27
	78.55 - 78.68	-.DVSGEGVQQALLK.-	1344.50	1	2.06	0.30	341.0	1	11/24	3.00E9
	78.70	-.DVSGEGVQQALLK.-	1344.50	2	4.08	0.43	1654.9	1	19/24	3.60E9
	62.82 - 63.82	-.NHLDDYVIGQEQAK.-	1630.74	2	3.81	0.59	1392.8	1	18/26	1.79E9
	101.80	-.VVIDESVIDGQSKPLLIYGKPEAQQASG	3072.41	3	4.09	0.42	871.9	1	29/112	4.10E9
#28	SUCC_ECOLI (P0746C					40.20			4 (4 0 0 0 0)	0.42
	73.88	-.KLADSGLNIIAAK.-	1314.56	2	3.51	0.52	1330.7	1	20/24	2.66E9
	78.08 - 79.31	-.LHGGEPANFLDVGGGATK.-	1740.90	2	3.59	0.52	918.2	1	19/34	3.38E9
	129.59 - 130.69	-.LVTYQTDANGQPVNQILVEAATDIAK.-	2774.08	2	3.96	0.59	293.0	1	17/50	9.73E8
	84.79	-.VALDPLTGPM*PYQGR.-	1631.88	2	3.27	0.52	939.2	1	16/28	3.37E9
#29	SYS_ECOLI (P09156)					40.18			4 (4 0 0 0 0)	0.26
	71.76	-.ARGEDIEPLRLEVNK.-	1739.96	1	2.37	0.40	129.8	3	11/28	8.27E8
	82.66	-.DHVTLGEM*HSGLDFAAAVK.-	2015.24	2	3.31	0.56	446.8	1	14/36	2.67E9
	120.54 - 121.31	-.IEVPEVLRPYM*NGLEYIG.-	2109.43	2	3.63	0.29	551.7	1	16/34	6.11E9
	79.46	-.LDVDKLGALEER.-	1358.52	2	3.15	0.39	1355.9	1	19/22	2.39E9
#30	SYN_ECO57 (P58694)					40.16			4 (4 0 0 0 0)	0.82
	96.24	-.KFENPVYWGVDLSSEHER.-	2193.36	2	3.13	0.01	793.9	1	16/34	4.10E9
	93.31	-.VEVAGWVEDPDTYPM*AAK.-	1995.20	2	2.86	0.49	499.9	1	14/34	6.73E9
	90.11	-.VSTLDLENLPR.-	1257.42	2	2.77	0.32	879.2	1	14/20	2.43E9
	67.42 - 68.58	-.VVASPGQGQQFEIQTSK.-	1804.98	2	2.75	0.07	257.2	3	14/32	2.43E10
#31	DNAK_ECOLI (P04475					30.27			3 (3 0 0 0 0)	0.24
	108.87 - 108.97	-.AKLESLVEDLVNR.-	1486.70	2	3.27	0.44	1095.3	1	16/24	4.24E9
	94.38	-.TFEVLATNGDTHLGGEDFDSR.-	2282.37	2	3.76	0.57	815.6	1	21/40	3.75E9
	96.61	-.VLENAEGDRTTPSIIAYTQDGETLVGQI	3331.64	3	5.40	0.60	1071.0	1	32/120	3.21E9
#32	CH60_ECOLI (P06139					30.23			3 (3 0 0 0 0)	0.26
	67.87 - 68.43	-.AIAQVGTISANSDETVGK.-	1761.91	2	4.65	0.69	801.5	1	20/34	4.20E9
	129.81	-.GYLSPYFINKPETGAVELESPFILLADKI	3241.72	2	3.72	0.58	334.2	1	15/56	4.01E9
	87.75	-.QIVLNCGEEPSVVANTVK.-	1958.20	2	4.47	0.65	1532.9	1	23/34	3.90E9
#33	AMPA_ECOLI (P11648					30.19			3 (3 0 0 0 0)	0.17
	89.90 - 90.47	-.AYRPGDVLTTM*SGQTVEVLNTDAEGI	2798.04	3	3.71	0.42	1542.8	1	30/100	4.40E9
	58.42 - 59.21	-.GNASEDARPIVLVGK.-	1526.72	2	3.49	0.49	1004.4	1	17/28	1.14E9
	84.01 - 84.89	-.RGELEGKPGQTLLLHHVPNVLSER.-	2681.05	3	3.61	0.54	880.5	3	28/92	2.19E9
#34	Q8X4L0 (Q8X4L0) Puta					30.18			3 (3 0 0 0 0)	0.25
	73.25 - 73.34	-.ATALILDKDDVVK.-	1401.63	2	3.05	0.34	728.2	1	15/24	3.03E9
	111.40	-.NFIEGDKVNQGDSLYQIDPAPLQAELN	3276.56	3	3.60	0.38	638.0	4	30/116	4.56E9
	103.98	-.TVPYEVAEIRPQVGGIIIK.-	2083.46	2	2.97	0.46	259.2	2	12/36	4.04E9
#35	Q8XBP7 (Q8XBP7) Ou					30.17			3 (3 0 0 0 0)	0.22
	77.13	-.NNLDNAVEQLR.-	1286.38	2	2.95	0.41	957.8	1	15/20	2.30E9
	84.41	-.QAQYNFVGASEQLESAHR.-	2036.15	3	3.31	0.54	1159.5	1	27/68	2.87E9
	120.24 - 120.80	-.QITGNYYPELAALNVENFK.-	2185.42	2	3.34	0.59	208.1	1	13/36	5.12E9
#36	KPY1_ECOLI (P14178)					20.26			2 (2 0 0 0 0)	0.23
	105.43	-.GAVETAEKLDAPLIVVATQGGK.-	2168.48	2	5.13	0.57	1047.2	1	20/42	3.78E9
	128.70	-.GDLGVEIPVEEVIFAQK.-	1844.10	2	3.03	0.56	274.3	1	15/32	6.57E9
#37	ACRA_ECOLI (P31223					20.23			2 (2 0 0 0 0)	0.13
	92.00	-.ARLEEGLNPNAILVPQQGVTR.-	2276.58	2	4.52	0.56	739.4	1	18/40	3.10E9
	88.85	-.QEYDQALADAQQANAAVTAAK.-	2178.30	2	4.64	0.58	722.2	1	17/40	3.00E9
#38	HSLU_ECOLI (P32168					20.23			2 (2 0 0 0 0)	0.11
	73.19	-.HLDALVADEDLSR.-	1454.57	2	3.48	0.62	1460.7	1	20/24	2.45E9
	82.39	-.IAEAAWQVNESTENIGAR.-	1960.09	2	4.59	0.66	1369.5	1	21/34	2.70E9
#39	MANC_ECO57 (O8534					20.22			2 (2 0 0 0 0)	0.27
	127.30	-.IVTFGIIPEYAETGYGYIER.-	2292.57	2	4.35	0.56	437.7	1	19/38	5.76E9
	131.93	-.NPQEDPLLLVLAADHVIAK.-	2057.38	2	3.56	0.60	571.2	1	15/36	6.86E9
#40	Q8XCJ4 (Q8XCJ4) Pyr					20.20			2 (2 0 0 0 0)	0.19
	102.74	-.GDLGVEIGDPELVGIQK.-	1739.95	2	3.98	0.43	944.1	1	20/32	7.37E9
	59.23	-.IVTTLGPATDRDNNLEK.-	1858.04	2	3.08	0.42	402.5	1	15/32	1.20E9
#41	ISCS_ECOLI (P39171)					20.17			2 (2 0 0 0 0)	0.19
	109.42 - 110.32	-.EGFEVTYLAPQRNGIIDLK.-	2164.45	2	3.38	0.57	623.3	1	17/36	5.30E9

	79.02	-.NQIADLVGADPR.-	1269.39	2	3.24	0.54	1415.5	1	17/22		3.45E9
#42	ODO2_ECOLI (P07016					20.15			2 (2 0 0 0 0)		0.09
	63.50	-.DVDTLGM*ADIEK.-	1323.45	2	2.51	0.39	539.2	1	14/22		1.37E9
	63.32 - 64.24	-.LLAEHNLDASAIK.-	1395.59	2	3.04	0.55	1746.3	1	19/24		2.90E9
#43	Q8X8H4 (Q8X8H4) Ou					20.14			2 (2 0 0 0 0)		0.21
	133.72 - 134.34	-.KVDALDLFADGSYELFGR.-	2017.23	2	2.74	0.36	445.4	1	12/34		7.00E9
	69.04	-.YIAEDNEGNAVNPNLPR.-	1887.00	2	2.89	0.34	335.1	1	13/32		2.73E9
#44	Q9LAP1 (Q9LAP1) Iha					20.14			2 (2 0 0 0 0)		0.12
	78.16	-.IPYPTESQNYNLGAR.-	1723.87	1	2.57	0.45	192.4	1	12/28		1.27E9
	77.87	-.IPYPTESQNYNLGAR.-	1723.87	2	2.78	0.37	566.9	1	15/28		4.11E9
#45	Q8X9B0 (Q8X9B0) Citr					10.30			1 (1 0 0 0 0)		0.12
	154.67 - 155.22	-.TAGSSGANPFACIAAGIASLWGPAHGC	3197.50	3	6.10	0.64	1001.6	1	41/132		5.39E9
#46	PUR2_ECO57 (Q8X61					10.26			1 (1 0 0 0 0)		0.02
	61.00 - 61.64	-.VGDKDTGPNTGGM*GAYSPAPVVTDD	2959.16	3	5.21	0.45	1202.0	1	39/112		1.08E9
#47	ALF_ECOLI (P11604) I					10.25			1 (1 0 0 0 0)		0.09
	114.15	-.FTIAASFGNVHGVYKPGNVVLTPTILR.-	2873.34	3	4.94	0.59	1338.6	1	33/104		4.25E9
#48	FABF_ECOLI (P39435)					10.24			1 (1 0 0 0 0)		0.10
	110.45	-.VVVTGLGM*LSPVGNTVESTWK.-	2191.53	2	4.73	0.69	649.2	1	19/40		4.49E9
#49	Q8X8G5 (Q8X8G5) Re					10.23			1 (1 0 0 0 0)		0.12
	100.29 - 100.91	-.GLTNEALNVTLVEAGER.-	1786.97	2	4.56	0.62	956.6	1	18/32		5.37E9
#50	Q8XA80 (Q8XA80) Thr					10.23			1 (1 0 0 0 0)		0.16
	129.40	-.ILSAFIGDEIPQEILEER.-	2073.33	2	4.54	0.49	957.8	1	21/34		7.36E9
#51	KBL_ECOLI (P07912)					10.23			1 (1 0 0 0 0)		0.10
	115.51	-.SRPYLFSNSLAPAIVAASIK.-	2106.45	2	4.51	0.57	694.9	1	19/38		4.37E9
#52	ILVC_ECO57 (P58256)					10.21			1 (1 0 0 0 0)		0.09
	101.23	-.DGAALGYSHGFNIVEVGEQIRK.-	2361.60	3	4.26	0.50	1292.8	1	35/84		4.01E9
#53	EAE_ECO57 (P43261)					10.21			1 (1 0 0 0 0)		0.10
	131.52	-.LPFEYSALPLLGSAPLVAAGGVAGHTN	2752.16	3	4.15	0.47	1756.4	1	38/108		4.53E9
#54	DEOB_ECOLI (P07651					10.21			1 (1 0 0 0 0)		0.05
	68.77 - 69.32	-.TGNRHDLAVEPPAPTVLQK.-	2044.30	2	4.13	0.59	274.4	1	14/36		2.30E9
#55	PEPB_ECO57 (P5847:					10.20			1 (1 0 0 0 0)		0.08
	84.20	-.DTINAPAEELGPSQLAQR.-	1911.06	2	4.01	0.62	443.2	1	14/34		3.57E9
#56	PNTB_ECOLI (P07002					10.20			1 (1 0 0 0 0)		0.10
	99.61	-.NSHSVIITPGYGM*AVAQAQYPVAEITE	2992.35	3	3.98	0.51	1739.0	1	36/108		4.45E9
#57	SURA_ECOLI (P21202					10.20			1 (1 0 0 0 0)		0.11
	121.62	-.IQELPGIFAQALSTAK.-	1687.96	2	3.96	0.49	609.1	1	20/30		4.92E9
#58	IMDH_ECOLI (P06981)					10.19			1 (1 0 0 0 0)		0.07
	88.69	-.FVTDLNQPVSVYM*TPK.-	1856.13	2	3.88	0.63	694.5	1	18/30		3.32E9
#59	Q8X9L2 (Q8X9L2) Sim					10.19			1 (1 0 0 0 0)		0.05
	89.04	-.IVVDCANGATYHIAPNVLR.-	2084.36	2	3.85	0.61	707.9	1	18/36		2.15E9
#60	ENGD_ECOLI (P3121€					10.19			1 (1 0 0 0 0)		0.05
	73.80	-.LDQLAEIVKPQR.-	1410.64	2	3.74	0.43	1171.4	1	15/22		2.24E9
#61	Q8XB65 (Q8XB65) Put					10.18			1 (1 0 0 0 0)		0.05
	73.65	-.TDKETPYVPIPEGGVK.-	1730.94	2	3.59	0.48	631.2	1	17/30		2.32E9
#62	SYH_ECO57 (P60908)					10.17			1 (1 0 0 0 0)		0.03
	59.56 - 60.52	-.AGIEHGLLYNQEQR.-	1628.77	2	3.50	0.46	786.3	1	15/26		1.17E9
#63	RHO_ECOLI (P03002)					10.17			1 (1 0 0 0 0)		0.19
	98.18 - 100.25	-.SADSSYLAGPDDIYVSPSQIR.-	2242.39	2	3.43	0.60	510.7	1	17/40		8.86E9
#64	HFLK_ECOLI (P25662)					10.17			1 (1 0 0 0 0)		0.07
	81.48	-.ELAASGVM*LTSDENVVR.-	1808.01	2	3.40	0.47	542.5	1	15/32		3.04E9
#65	PUR9_ECO57 (Q8X61					10.16			1 (1 0 0 0 0)		0.08
	95.10	-.HANPCGVAIGNSILDAYDR.-	2044.21	2	3.25	0.55	394.2	1	14/36		3.74E9
#66	ENTC_ECOLI (P10377					10.16			1 (1 0 0 0 0)		0.07
	82.56	-.FDEPAVNGDSPDSPFQQK.-	1979.05	2	3.23	0.61	943.4	1	21/34		3.22E9
#67	Q8X633 (Q8X633) Out					10.16			1 (1 0 0 0 0)		0.08
	108.06	-.IQGVETELKIPFNDEWK.-	2047.30	2	3.22	0.33	272.3	2	11/32		3.46E9
#68	GM4D_ECOLI (P32054					10.16			1 (1 0 0 0 0)		0.15
	116.36	-.FYQASTSELYGLVQEIPQK.-	2202.45	2	3.21	0.23	327.9	1	15/36	1	6.87E9
#69	GSA_ECO57 (Q8X4V5					10.16			1 (1 0 0 0 0)		0.10
	90.83	-.AGSGALTLGQPNSPGVPADFAK.-	2056.27	2	3.15	0.52	405.7	1	16/42		4.61E9
#70	PTGB_ECOLI (P05053					10.16			1 (1 0 0 0 0)		0.09
	93.97	-.ATGTSEM*APALVAAFGGK.-	1695.92	2	3.11	0.56	467.4	1	15/34		4.34E9
#71	RvrsDB|00003623					10.15			1 (1 0 0 0 0)		0.21
	72.06	-.EELVRLLR.-	1028.23	2	3.08	0.00	724.8	1	13/14		9.62E9
#72	RECA_ECOLI (P03017					10.15			1 (1 0 0 0 0)		0.04
	60.48 - 61.90	-.IVEIYGPESSGK.-	1279.42	2	2.91	0.35	1199.3	1	18/22		1.90E9
#73	DDLA_ECOLI (P23844					10.15			1 (1 0 0 0 0)		0.02
	47.42 - 48.86	-.HNISFAEVESK.-	1261.37	2	2.90	0.53	1775.0	1	17/20		8.27E8
#74	YFEU_ECO57 (Q8XBJ					10.15			1 (1 0 0 0 0)		0.32
	94.68 - 95.25	-.M*QLEKMITEGSNAASAEIDR.-	2211.46	2	2.90	0.00	512.6	2	17/38		1.46E10
#75	Q8X5J4 (Q8X5J4) Alcc					10.14			1 (1 0 0 0 0)		0.07
	113.23	-.AAVAFAPGKPLEIVEIDVAPPK.-	2233.64	2	2.88	0.46	196.9	1	12/42		3.33E9
#76	Q8XCQ8 (Q8XCQ8) 3-					10.14			1 (1 0 0 0 0)		0.05
	12.52	-.VGLIAGSGGGSPR.-	1128.26	2	2.79	0.43	1130.6	1	18/24		2.21E9

#77	Q8X3N3 (Q8X3N3) Hy|					10.14			1 (1 0 0 0 0)		0.11
	116.41 - 117.14	-.M*IETLLDFSGLEDISR.-	1856.09	2	2.76	0.14	429.6	1	16/30	1	4.84E9
#78	GADC_ECO57 (P5822					10.13			1 (1 0 0 0 0)		0.04
	71.36	-.ANTGVTLEPINSQNAPK.-	1754.92	2	2.67	0.52	281.8	2	12/32		1.75E9
#79	RvrsDB|00003813					10.13			1 (1 0 0 0 0)		0.03
	52.35 - 53.50	-.YGM*NKKKELEEK.-	1513.74	2	2.63	0.17	431.2	1	13/22		1.50E9
#80	Q8X5T3 (Q8X5T3) Put					10.13			1 (1 0 0 0 0)		0.34
	112.12	-.QLAQPLVAAELVVPDFADHVLKR.-	2530.95	2	2.61	0.10	487.4	1	16/44		1.54E10
#81	Q8X5N6 (Q8X5N6) Put					10.13			1 (1 0 0 0 0)		0.03
	63.76	-.VPLDELTPHAEK.-	1349.52	2	2.60	0.48	1382.6	1	17/22		1.57E9
#82	Q8XBZ6 (Q8XBZ6) Pe					10.13			1 (1 0 0 0 0)		0.03
	63.40 - 64.01	-.NADALTLQAPAQR.-	1369.51	2	2.60	0.49	808.1	1	14/24		1.40E9
#83	RvrsDB|00000517					10.13			1 (1 0 0 0 0)		0.12
	139.83 - 140.47	-.TEGNEDVYSYKAPRQAR.-	1985.10	2	2.56	0.32	392.2	1	13/32		5.30E9
#84	SERC_ECO57 (Q8XE/					10.13			1 (1 0 0 0 0)		0.13
	123.86 - 124.87	-.AQIFNFSSGPAM*LPVEVLK.-	2065.42	2	2.52	0.28	270.2	22	10/36		5.78E9
#85	Q8XEF0 (Q8XEF0) Glu					10.11			1 (1 0 0 0 0)		0.04
	13.18	-.ELCDKAEK.-	993.09	1	2.12	0.11	140.3	6	9/14		1.88E9
#86	RvrsDB|00001691					10.10			1 (1 0 0 0 0)		0.07
	94.95	-.SSNLSDSM*LLATTAKK.-	1683.91	1	1.98	0.12	134.1	26	9/30		3.43E9
#87	RvrsDB|00000720					10.10			1 (1 0 0 0 0)		0.09
	80.05 - 80.94	-.EGNNIQVLGK.-	1072.20	1	1.96	0.09	378.4	15	9/18		4.03E9
#88	Q8X9T0 (Q8X9T0) Hel					10.10			1 (1 0 0 0 0)		0.06
	69.42	-.DTADALNK.-	847.89	1	1.94	0.11	909.7	1	11/14		2.55E9
#89	ZNUC_ECOLI (P52648					10.09			1 (1 0 0 0 0)		0.27
	136.39 - 137.32	-.EDILPALK.-	899.07	1	1.90	0.09	227.5	17	8/14		1.22E10
