# Supplementary material for: Proteins other than the locus of enterocyte effacement-encoded proteins contribute to Escherichia coli O157:H7 adherence to bovine rectoanal junction stratified squamous epithelial cells
Source: BMC Microbiol. 2012 Jun 12;12:103. doi: 10.1186/1471-2180-12-103 (PMC3420319; doi:10.1186/1471-2180-12-103)
Supplement: Additional file 8 — http://www.biomedcentral.com/imedia/3116488396754199/supp8.pdf. DATA SHEETS: O157-DMEM MS/MS data sheet 4. [file 1471-2180-12-103-S8.pdf]

DMEM-04 SequestReport

	Reference					Score	Accession		Peptides (Hits)		Area
	Time(s)	Sequence	MH+	Charge	XC	Delta Cn	Sp	RSp	Ions	Count	Peak Area
#1	G3P1_ECOLI (P06977					780.35			78 (78 0 0 0 0)		24.78
	84.41 - 85.56	-.AGIALNDNFVK.-	1162.32	2	3.51	0.42	824.1	1	15/20		1.98E10
	82.49 - 83.84	-.AGIALNDNFVK.-	1162.32	2	2.78	0.27	1176.5	1	16/20		2.13E10
	80.49	-.AGIALNDNFVK.-	1162.32	1	1.84	0.30	336.2	1	12/20		1.29E9
	80.47	-.AGIALNDNFVK.-	1162.32	2	2.94	0.43	942.0	1	16/20		1.59E9
	82.45 - 87.55	-.AGIALNDNFVK.-	1162.32	1	2.49	0.30	482.8	1	13/20		3.22E10
	9.06 - 12.23	-.DGHLIVNGK.-	953.08	1	1.91	0.55	691.0	1	11/16		1.08E10
	30.46	-.DGHLIVNGKK.-	1081.25	2	2.52	0.46	1395.1	1	14/18		2.59E8
	12.19	-.DNTPM*FVK.-	968.11	1	1.84	0.23	108.6	3	8/14		2.38E9
	11.50	-.FDGTVEVK.-	894.99	2	2.51	0.29	808.4	1	14/14		3.44E9
	79.14 - 79.69	-.FDGTVEVKDGHLIVNGKK.-	1957.22	2	4.55	0.41	920.3	1	18/34		1.77E9
	95.00 - 95.95	-.GANFDKYAGQDIVSNASCTTNCLAPLAK.-	2988.24	3	7.09	0.64	2249.0	1	44/108		6.21E9
	95.06 - 96.15	-.GANFDKYAGQDIVSNASCTTNCLAPLAK.-	2988.24	2	4.67	0.63	579.8	1	20/54		2.90E9
	106.15 - 107.24	-.GANFDKYAGQDIVSNASCTTNCLAPLAK.-	2988.24	3	4.44	0.54	674.6	1	30/108		5.15E9
	100.97 - 102.20	-.GANFDKYAGQDIVSNASCTTNCLAPLAK.-	2988.24	3	5.24	0.54	1651.5	1	42/108		9.73E9
	98.54 - 99.80	-.GANFDKYAGQDIVSNASCTTNCLAPLAK.-	2988.24	3	5.51	0.55	1035.1	1	37/108		1.23E10
	16.11 - 17.24	-.GASQNIIPSSTGAAK.-	1402.54	2	2.51	0.22	467.9	3	16/28		2.53E8
	28.05 - 29.92	-.GASQNIIPSSTGAAK.-	1402.54	2	3.01	0.32	504.0	1	17/28		1.19E9
	30.32 - 31.59	-.GASQNIIPSSTGAAK.-	1402.54	2	2.69	0.41	488.8	1	16/28		8.42E8
	24.03 - 25.26	-.GASQNIIPSSTGAAK.-	1402.54	2	3.17	0.30	646.0	1	18/28		3.84E8
	12.08 - 13.26	-.GASQNIIPSSTGAAK.-	1402.54	1	3.25	0.47	418.4	1	15/28		4.16E9
	34.38 - 35.61	-.GASQNIIPSSTGAAK.-	1402.54	2	2.63	0.13	496.3	3	16/28		3.45E8
	12.15 - 13.30	-.GASQNIIPSSTGAAK.-	1402.54	2	3.75	0.34	885.5	1	20/28		1.34E10
	13.90 - 15.38	-.GASQNIIPSSTGAAK.-	1402.54	2	2.99	0.39	712.3	1	19/28		7.36E9
	18.09 - 19.29	-.GASQNIIPSSTGAAK.-	1402.54	2	2.76	0.34	692.1	1	18/28		2.99E8
	22.06 - 23.43	-.GASQNIIPSSTGAAK.-	1402.54	2	2.89	0.35	500.9	1	17/28		2.14E8
	19.89 - 21.34	-.GASQNIIPSSTGAAK.-	1402.54	2	2.87	0.41	450.0	1	17/28		2.21E8
	127.43 - 128.84	-.GVLGYTEDDVVSTDFNGEVCTSVFDAK.-	2926.09	2	5.43	0.71	770.5	1	23/52		4.73E9
	125.78 - 126.84	-.GVLGYTEDDVVSTDFNGEVCTSVFDAK.-	2926.09	2	4.66	0.69	692.6	1	21/52		5.29E9
	129.51 - 130.84	-.GVLGYTEDDVVSTDFNGEVCTSVFDAK.-	2926.09	2	4.70	0.69	547.7	1	21/52		4.59E9
	132.92 - 134.11	-.GVLGYTEDDVVSTDFNGEVCTSVFDAK.-	2926.09	2	3.61	0.53	289.7	1	15/52		3.78E9
	135.04 - 135.88	-.GVLGYTEDDVVSTDFNGEVCTSVFDAK.-	2926.09	2	4.31	0.64	500.3	1	18/52		3.34E9
	123.71 - 125.18	-.GVLGYTEDDVVSTDFNGEVCTSVFDAK.-	2926.09	2	4.93	0.65	625.0	1	19/52		8.06E9
	136.98 - 138.12	-.GVLGYTEDDVVSTDFNGEVCTSVFDAK.-	2926.09	2	3.64	0.64	444.5	1	18/52		3.38E9
	138.88 - 139.98	-.GVLGYTEDDVVSTDFNGEVCTSVFDAK.-	2926.09	2	3.93	0.72	297.6	1	15/52		2.73E9
	143.51	-.GVLGYTEDDVVSTDFNGEVCTSVFDAK.-	2926.09	2	3.09	0.59	240.8	1	13/52		1.85E9
	157.80 - 159.26	-.GVLGYTEDDVVSTDFNGEVCTSVFDAK.-	2926.09	2	3.16	0.57	235.9	1	14/52		1.89E9
	140.73	-.GVLGYTEDDVVSTDFNGEVCTSVFDAK.-	2926.09	2	3.00	0.57	350.4	1	16/52		1.92E9
	121.86 - 123.14	-.GVLGYTEDDVVSTDFNGEVCTSVFDAK.-	2926.09	2	4.85	0.62	649.9	1	20/52		6.39E9
	119.87 - 121.21	-.GVLGYTEDDVVSTDFNGEVCTSVFDAK.-	2926.09	2	3.68	0.63	282.8	1	14/52		4.80E9
	204.03 - 204.71	-.GVLGYTEDDVVSTDFNGEVCTSVFDAK.-	2926.09	2	4.39	0.59	648.0	1	20/52		3.93E8
	83.70 - 84.29	-.LVSWYDNETGYSNK.-	1676.77	2	4.16	0.70	1229.6	1	20/26		7.74E9
	81.87 - 83.12	-.LVSWYDNETGYSNK.-	1676.77	2	4.64	0.67	1496.0	1	21/26		2.89E10
	77.49 - 78.19	-.LVSWYDNETGYSNK.-	1676.77	2	3.72	0.66	881.4	1	19/26		3.16E9
	80.28 - 81.40	-.LVSWYDNETGYSNK.-	1676.77	2	4.31	0.69	755.1	1	15/26		2.76E10
	80.78 - 83.89	-.LVSWYDNETGYSNK.-	1676.77	1	3.28	0.59	572.3	1	17/26		1.23E10
	139.47 - 140.81	-.RSDIEIVAINDLLDADYM*AYM*LK.-	2706.09	3	6.93	0.65	2112.1	1	36/88		6.28E9
	139.86	-.RSDIEIVAINDLLDADYM*AYM*LK.-	2706.09	2	3.15	0.62	406.8	1	18/44		2.18E9
	153.68 - 154.98	-.SDIEIVAINDLLDADYM*AYM*LK.-	2549.90	3	3.54	0.56	845.5	3	23/84		1.39E9
	149.82 - 151.34	-.SDIEIVAINDLLDADYM*AYM*LK.-	2549.90	3	6.46	0.59	2657.5	1	37/84		5.04E9
	125.52 - 126.54	-.VINDNFGIIEGLM*TTVHATTATQK.-	2591.92	3	4.40	0.53	520.9	1	30/92		5.96E9
	120.34 - 121.09	-.VINDNFGIIEGLM*TTVHATTATQK.-	2591.92	2	3.63	0.45	609.6	1	18/46		5.48E9
	127.06 - 128.49	-.VINDNFGIIEGLM*TTVHATTATQK.-	2591.92	3	4.79	0.59	694.0	1	33/92		7.25E9
	121.81 - 123.10	-.VINDNFGIIEGLM*TTVHATTATQK.-	2591.92	3	4.50	0.52	531.5	1	29/92		1.13E10
	120.10 - 121.31	-.VINDNFGIIEGLM*TTVHATTATQK.-	2591.92	3	5.01	0.61	407.4	1	30/92		2.37E10
	132.71 - 133.32	-.VINDNFGIIEGLM*TTVHATTATQK.-	2591.92	3	3.22	0.50	377.8	2	26/92		3.19E9
	123.73 - 124.95	-.VINDNFGIIEGLM*TTVHATTATQK.-	2591.92	3	5.39	0.65	580.4	1	32/92		8.62E9
	150.00 - 151.16	-.VINDNFGIIEGLM*TTVHATTATQK.-	2591.92	3	3.32	0.56	463.8	1	26/92		1.54E9
	162.23 - 162.84	-.VINDNFGIIEGLM*TTVHATTATQK.-	2591.92	3	3.02	0.47	314.2	5	26/92		9.94E8
	117.30 - 118.57	-.VINDNFGIIEGLM*TTVHATTATQK.-	2591.92	2	3.89	0.56	249.8	1	12/46		1.33E10
	118.22 - 119.52	-.VINDNFGIIEGLM*TTVHATTATQK.-	2591.92	3	4.40	0.57	669.1	1	32/92		4.97E10
	203.72 - 205.46	-.VINDNFGIIEGLM*TTVHATTATQK.-	2591.92	3	3.70	0.51	432.0	1	27/92		2.92E8
	118.34 - 119.76	-.VINDNFGIIEGLM*TTVHATTATQK.-	2591.92	2	4.20	0.59	384.2	1	14/46		1.92E10
	138.38 - 138.92	-.VINDNFGIIEGLMTTVHATTATQK.-	2575.92	3	5.22	0.59	525.4	1	34/92		4.57E9
	136.32 - 136.94	-.VINDNFGIIEGLMTTVHATTATQK.-	2575.92	2	3.83	0.56	351.7	1	14/46		2.46E9
	136.07 - 136.66	-.VINDNFGIIEGLMTTVHATTATQK.-	2575.92	3	4.96	0.66	751.1	1	35/92		9.73E9
	85.33 - 87.23	-.VLDLIAHISK.-	1109.34	1	2.86	0.40	486.1	1	13/18		7.43E9
	85.58 - 86.69	-.VLDLIAHISK.-	1109.34	2	3.39	0.44	766.5	1	14/18		7.53E9
	87.31	-.VLDLIAHISK.-	1109.34	2	3.02	0.40	816.7	1	14/18		6.99E9
	93.30 - 93.61	-.VLPELNGKLTGM*AFR.-	1662.98	2	3.19	0.52	827.5	1	20/28		4.84E9
	100.91 - 101.54	-.VPTPNVSVVDLTVR.-	1496.73	2	3.49	0.58	808.7	1	16/26		3.45E9

	93.86 - 94.53	-.VPTPNVSVVDLTVR.-	1496.73	2	3.79	0.63	1474.4	1	17/26		4.42E9
	97.38 - 98.65	-.VPTPNVSVVDLTVR.-	1496.73	2	3.97	0.62	1561.4	1	18/26		8.34E10
	97.61 - 99.24	-.VPTPNVSVVDLTVR.-	1496.73	1	2.90	0.61	474.3	1	16/26		1.49E10
	97.82 - 98.40	-.VPTPNVSVVDLTVR.-	1496.73	1	3.63	0.61	408.5	1	15/26		7.88E9
	99.22 - 100.33	-.VPTPNVSVVDLTVR.-	1496.73	2	3.59	0.67	1407.9	1	18/26		5.54E10
	107.12	-.VPTPNVSVVDLTVRLEK.-	1867.18	3	3.24	0.39	646.2	1	27/64		1.13E9
	10.69	-.VTAERDPANLK.-	1214.35	2	2.90	0.32	478.3	2	14/20		5.79E9
	150.96	-.WDEVGVDVVAEATGLFLTDETARK.-	2622.87	3	5.31	0.56	1124.2	1	38/92		1.56E9
#2	OMPC_ECO57 (Q8XE					760.32			76 (76 0 0 0 0)		16.32
	137.25 - 138.53	-.AQNFEAVAQYQFDFGLRPSLAYLQSK.-	2993.32	3	5.42	0.34	1087.3	1	35/100		4.49E9
	130.52	-.AQNFEAVAQYQFDFGLRPSLAYLQSK.-	2993.32	2	4.01	0.52	296.8	1	16/50		3.54E9
	130.87 - 132.07	-.AQNFEAVAQYQFDFGLRPSLAYLQSK.-	2993.32	3	6.39	0.34	1024.0	1	34/100		1.03E10
	133.36 - 134.58	-.AQNFEAVAQYQFDFGLRPSLAYLQSK.-	2993.32	3	4.69	0.46	930.9	1	32/100		5.16E9
	135.14 - 135.97	-.AQNFEAVAQYQFDFGLRPSLAYLQSK.-	2993.32	3	4.23	0.38	481.9	2	24/100		2.79E9
	128.89 - 130.32	-.AQNFEAVAQYQFDFGLRPSLAYLQSK.-	2993.32	3	4.08	0.45	482.7	1	24/100		1.09E10
	139.04	-.AQNFEAVAQYQFDFGLRPSLAYLQSK.-	2993.32	3	4.18	0.44	1052.7	1	30/100		2.52E9
	12.43 - 13.12	-.DGNKLDLYGK.-	1123.24	1	2.17	0.38	570.1	1	13/18	3	1.55E9
	31.90	-.DGNKLDLYGK.-	1123.24	1	2.93	0.41	400.7	1	12/18	3	3.23E8
	32.40 - 33.94	-.DGNKLDLYGK.-	1123.24	2	3.44	0.40	1283.8	1	16/18	3	4.47E8
	12.41 - 12.95	-.DGNKLDLYGK.-	1123.24	2	2.89	0.38	1153.4	1	14/18	3	2.01E9
	30.50 - 32.07	-.DGNKLDLYGK.-	1123.24	2	3.18	0.34	1333.9	1	15/18	3	5.77E8
	119.09 - 120.36	-.EALRQNGDGVGGSITYDYEGFGIGAAVSSSK	3107.29	3	6.38	0.62	2784.9	1	46/120		5.34E9
	117.15 - 118.51	-.EALRQNGDGVGGSITYDYEGFGIGAAVSSSK	3107.29	3	5.27	0.58	1305.5	1	35/120		4.62E9
	112.85 - 114.15	-.EALRQNGDGVGGSITYDYEGFGIGAAVSSSK	3107.29	3	5.50	0.64	1956.5	1	38/120		4.08E9
	114.73 - 115.96	-.EALRQNGDGVGGSITYDYEGFGIGAAVSSSK	3107.29	3	5.49	0.63	2090.2	1	41/120		4.33E9
	120.98 - 122.19	-.EALRQNGDGVGGSITYDYEGFGIGAAVSSSK	3107.29	3	5.63	0.60	1855.2	1	38/120		4.47E9
	128.16 - 128.71	-.EALRQNGDGVGGSITYDYEGFGIGAAVSSSK	3107.29	3	4.43	0.51	741.9	2	26/120		2.66E9
	122.78 - 123.75	-.EALRQNGDGVGGSITYDYEGFGIGAAVSSSK	3107.29	3	5.81	0.59	2294.0	1	41/120		4.57E9
	76.92 - 79.42	-.FQDVGSFDYGR.-	1291.35	1	1.92	0.36	408.4	1	13/20		1.19E10
	78.61 - 79.77	-.FQDVGSFDYGR.-	1291.35	2	3.46	0.47	1289.3	1	18/20		1.48E10
	76.90 - 78.08	-.FQDVGSFDYGR.-	1291.35	2	3.34	0.50	1158.8	1	16/20		1.94E10
	93.51 - 94.80	-.INLLDDNQFTR.-	1349.48	2	3.71	0.42	1279.6	1	18/20		3.26E10
	90.99 - 91.62	-.INLLDDNQFTR.-	1349.48	2	4.07	0.38	1168.2	1	17/20		7.62E9
	89.34 - 90.87	-.INLLDDNQFTR.-	1349.48	1	2.00	0.34	424.0	1	13/20		4.22E9
	93.59 - 95.63	-.INLLDDNQFTR.-	1349.48	1	1.82	0.33	265.0	2	10/20		1.68E10
	93.73	-.INLLDDNQFTR.-	1349.48	1	2.46	0.38	466.7	1	13/20		8.81E9
	89.28 - 90.39	-.INLLDDNQFTR.-	1349.48	2	4.20	0.49	844.5	1	17/20		8.90E9
	93.18 - 94.35	-.NLGVINGRNYDDEDILK.-	1949.11	2	3.53	0.36	1664.6	1	23/32		4.19E9
	61.34 - 62.54	-.NMSTYVDYK.-	1121.25	1	1.92	0.24	317.0	1	11/16	3	1.86E9
	145.42 - 146.83	-.NTDFFGLVDGLNFAVQYQGK.-	2234.45	2	3.50	0.57	641.2	1	15/38		5.48E9
	147.73 - 148.46	-.NTDFFGLVDGLNFAVQYQGK.-	2234.45	2	3.89	0.59	509.4	1	14/38		3.81E9
	136.43 - 137.63	-.NTDFFGLVDGLNFAVQYQGK.-	2234.45	2	3.94	0.61	580.0	1	18/38		7.34E9
	149.51 - 150.61	-.NTDFFGLVDGLNFAVQYQGK.-	2234.45	2	3.90	0.61	767.9	1	16/38		2.46E9
	135.78 - 137.02	-.NTDFFGLVDGLNFAVQYQGK.-	2234.45	2	3.66	0.48	406.7	1	14/38		5.67E9
	151.79 - 153.01	-.NTDFFGLVDGLNFAVQYQGK.-	2234.45	2	3.34	0.39	313.1	1	13/38		2.02E9
	164.40	-.NTDFFGLVDGLNFAVQYQGK.-	2234.45	2	2.77	0.54	314.7	6	12/38		7.66E8
	65.99 - 68.09	-.NYDDEDILK.-	1125.17	1	2.86	0.36	366.4	1	12/16		7.88E9
	76.09	-.NYDDEDILK.-	1125.17	1	2.57	0.37	671.6	1	12/16		1.38E9
	65.79 - 70.22	-.NYDDEDILK.-	1125.17	1	2.20	0.03	351.3	2	12/16		1.82E10
	72.52	-.NYDDEDILK.-	1125.17	1	2.43	0.38	703.5	1	12/16		9.12E8
	61.71	-.NYDDEDILK.-	1125.17	1	2.04	0.27	265.0	4	10/16		1.45E9
	66.87 - 68.07	-.NYDDEDILK.-	1125.17	2	2.81	0.29	586.1	1	14/16		9.18E9
	65.75	-.PSLAYLQSK.-	1007.17	1	2.34	0.00	548.9	1	12/16		1.16E9
	65.68	-.PSLAYLQSK.-	1007.17	2	2.75	0.00	1231.5	1	14/16		1.22E9
	60.08 - 61.23	-.RTDDQNSPLYIGNGDR.-	1821.89	2	4.77	0.52	1227.8	1	22/30		4.99E9
	60.57 - 61.13	-.RTDDQNSPLYIGNGDR.-	1821.89	3	3.20	0.43	1009.1	1	27/60		1.42E9
	65.13 - 66.26	-.RTDDQNSPLYIGNGDR.-	1821.89	2	3.79	0.53	894.3	1	18/30		1.12E9
	61.82 - 62.81	-.RTDDQNSPLYIGNGDR.-	1821.89	2	3.58	0.50	1143.2	1	20/30		2.23E9
	58.36 - 59.49	-.RTDDQNSPLYIGNGDR.-	1821.89	2	4.65	0.53	1024.1	1	22/30		5.79E9
	86.57 - 87.84	-.RTDDQNSPLYIGNGDRAETYTGGLK.-	2742.90	3	3.67	0.41	1186.0	1	36/96		7.69E9
	83.09 - 83.93	-.RTDDQNSPLYIGNGDRAETYTGGLK.-	2742.90	2	3.13	0.48	303.8	1	16/48		2.88E9
	82.37 - 83.49	-.RTDDQNSPLYIGNGDRAETYTGGLK.-	2742.90	3	4.78	0.48	1075.4	1	37/96		6.91E9
	84.05 - 85.12	-.RTDDQNSPLYIGNGDRAETYTGGLK.-	2742.90	3	5.62	0.59	1579.1	1	40/96		6.09E9
	69.47 - 70.60	-.TDDQNSPLYIGNGDR.-	1665.70	2	3.67	0.52	1380.0	1	19/28		1.19E10
	69.71 - 70.99	-.TDDQNSPLYIGNGDR.-	1665.70	1	2.40	0.42	371.6	1	14/28		2.78E9
	71.10 - 72.23	-.TDDQNSPLYIGNGDR.-	1665.70	2	3.54	0.46	1251.7	1	19/28		8.95E9
	87.88 - 89.25	-.TDDQNSPLYIGNGDRAETYTGGLK.-	2586.71	2	4.14	0.57	513.7	1	19/46		6.34E9
	91.83 - 92.41	-.TDDQNSPLYIGNGDRAETYTGGLK.-	2586.71	3	3.68	0.38	639.8	1	30/92		8.77E9
	89.46	-.TDDQNSPLYIGNGDRAETYTGGLK.-	2586.71	3	3.53	0.49	546.1	1	30/92		5.17E9
	93.40	-.TDDQNSPLYIGNGDRAETYTGGLK.-	2586.71	2	2.68	0.49	218.3	2	13/46		5.22E9
	91.85 - 92.62	-.TDDQNSPLYIGNGDRAETYTGGLK.-	2586.71	2	4.61	0.67	643.1	1	22/46		7.79E9
	68.63 - 69.21	-.VDGLHYFSDDK.-	1296.37	2	3.14	0.49	1359.5	1	16/20		1.70E9
	70.62 - 71.24	-.VDGLHYFSDDK.-	1296.37	1	3.42	0.49	642.4	1	13/20		1.83E9
	70.39 - 71.55	-.VDGLHYFSDDK.-	1296.37	2	3.72	0.55	1240.9	1	17/20		7.27E9

	76.04 - 77.36	-.VDGLHYFSDDKSVDGDQTYM*R.-	2465.60	3	4.40	0.53	1488.0	1	32/80	8.55E9
	84.91 - 85.96	-.VDGLHYFSDDKSVDGDQTYM*R.-	2465.60	3	4.33	0.45	990.2	2	25/80	2.60E9
	84.45 - 85.00	-.VDGLHYFSDDKSVDGDQTYMR.-	2449.60	3	4.50	0.58	1813.8	1	35/80	3.38E9
	84.31 - 84.85	-.VDGLHYFSDDKSVDGDQTYMR.-	2449.60	2	3.51	0.61	1261.4	1	20/40	2.29E9
	50.73 - 51.97	-.VGSLGWANK.-	932.06	2	2.76	0.44	874.3	1	14/16	2.16E9
	50.63 - 55.40	-.VGSLGWANK.-	932.06	1	2.24	0.31	314.8	1	11/16	5.06E9
	54.34	-.VGSLGWANK.-	932.06	2	2.85	0.44	899.1	1	14/16	1.09E9
	52.54 - 53.71	-.VGSLGWANK.-	932.06	2	2.99	0.47	818.9	1	14/16	2.30E9
	89.83 - 90.56	-.YVDVGATYYFNK.-	1440.58	2	3.62	0.49	1243.2	1	17/22	1.13E10
	88.13 - 90.12	-.YVDVGATYYFNK.-	1440.58	1	1.93	0.32	424.5	1	12/22	1.27E10
	88.03 - 89.22	-.YVDVGATYYFNK.-	1440.58	2	5.04	0.57	1735.5	1	20/22	1.46E10
#3	PGK_ECO57 (Q8XD03					370.31			37 (37 0 0 0 0)	5.99
	149.37	-.ADEQILDIGDASAQELAEILK.-	2243.45	2	3.47	0.49	720.8	1	20/40	1.36E9
	147.10 - 147.83	-.ADEQILDIGDASAQELAEILK.-	2243.45	2	4.89	0.57	1215.1	1	25/40	1.60E10
	15.99 - 16.71	-.ADLNVPVK.-	856.00	2	2.69	0.43	917.8	1	12/14	2.19E8
	53.72 - 54.87	-.ALKEPARPM*VAIVGGSK.-	1741.09	2	3.35	0.52	226.6	1	14/32	4.33E9
	68.57 - 69.10	-.ALKEPARPMVAIVGGSK.-	1725.09	2	4.52	0.59	923.4	1	18/32	1.27E9
	144.92 - 146.12	-.DYLDGVDVAEGELVVLENVR.-	2205.41	2	3.28	0.42	591.8	2	15/38	2.54E9
	142.14 - 143.27	-.DYLDGVDVAEGELVVLENVR.-	2205.41	2	5.16	0.53	2239.8	1	25/38	3.06E9
	137.57	-.DYLDGVDVAEGELVVLENVR.-	2205.41	2	3.66	0.55	1380.9	1	22/38	2.09E9
	137.87 - 138.49	-.FADVACAGPLLAAELDALGK.-	2003.28	3	6.03	0.55	3971.3	1	43/76	2.39E9
	137.32 - 138.44	-.FADVACAGPLLAAELDALGK.-	2003.28	2	6.19	0.71	2471.3	1	28/38	1.35E10
	116.22 - 116.86	-.IADQLIVGGGIANTFIAAQGHDVGK.-	2466.78	3	3.86	0.55	913.9	1	34/96	5.02E9
	130.70 - 131.90	-.ISYISTGGGAFLEFVEGK.-	1876.10	2	5.31	0.59	1914.1	1	24/34	4.87E9
	190.02	-.ISYISTGGGAFLEFVEGK.-	1876.10	2	2.86	0.62	698.7	1	17/34	8.31E8
	132.41 - 133.64	-.ISYISTGGGAFLEFVEGK.-	1876.10	2	4.84	0.66	1419.7	1	20/34	4.79E9
	134.36	-.ISYISTGGGAFLEFVEGK.-	1876.10	2	3.88	0.67	1483.5	1	20/34	3.31E9
	107.22	-.KYAALCDVFVM*DAFGTAHR.-	2189.47	3	4.79	0.49	1696.4	1	30/72	3.12E9
	90.33 - 90.42	-.LLTTCNIPVPSDVR.-	1585.82	1	2.72	0.37	253.9	1	12/26	1.85E9
	90.10 - 90.64	-.LLTTCNIPVPSDVR.-	1585.82	2	3.61	0.46	1235.3	1	20/26	1.13E10
	82.22 - 82.86	-.LTVLDSLSK.-	976.15	2	2.68	0.38	657.4	1	14/16	1.83E9
	137.15 - 138.02	-.LVKDYLDGVDVAEGELVVLENVR.-	2545.87	3	5.20	0.51	1648.5	1	33/88	3.06E9
	82.26 - 82.32	-.SLYEADLVDEAK.-	1353.46	1	2.04	0.15	686.1	3	12/22	1.62E9
	82.20 - 82.82	-.SLYEADLVDEAK.-	1353.46	2	3.16	0.49	1461.6	1	17/22	2.79E9
	77.11 - 77.69	-.SLYEADLVDEAKR.-	1509.64	2	3.59	0.48	1809.6	1	19/24	4.73E9
	77.24 - 77.28	-.SLYEADLVDEAKR.-	1509.64	1	2.36	0.41	314.8	2	11/24	1.20E9
	148.34 - 148.63	-.SVNDVKADEQILDIGDASAQELAEILK.-	2886.16	3	5.18	0.47	1295.8	1	34/104	3.14E9
	148.53	-.SVNDVKADEQILDIGDASAQELAEILK.-	2886.16	2	3.59	0.35	564.0	1	17/52	1.30E9
	143.77 - 145.28	-.TILWNGPVGVFEFPNFR.-	1994.28	2	4.77	0.67	1443.4	1	20/32	9.07E9
	147.71 - 148.97	-.TILWNGPVGVFEFPNFR.-	1994.28	2	4.63	0.69	1686.4	1	21/32	4.80E9
	145.95 - 147.19	-.TILWNGPVGVFEFPNFR.-	1994.28	2	4.99	0.67	2049.5	1	23/32	1.28E10
	74.46 - 75.63	-.VATEFSETAPATLK.-	1465.63	2	4.91	0.62	1168.4	1	19/26	9.79E9
	74.37 - 74.92	-.VATEFSETAPATLK.-	1465.63	1	2.47	0.42	321.9	1	12/26	4.83E9
	88.11 - 88.68	-.VLPAVAM*LEER.-	1244.49	2	3.18	0.54	497.1	1	16/20	4.07E9
	109.01 - 109.60	-.VLPAVAMLEER.-	1228.49	2	3.76	0.56	1105.2	1	18/20	1.76E9
	124.62 - 125.99	-.VM*VTSHLGRPTEGEYNEEFSLLPVVNYLK.-	3339.76	3	5.29	0.49	1415.5	1	33/112	8.35E9
	127.14 - 127.96	-.VMVTSHLGRPTEGEYNEEFSLLPVVNYLK.-	3323.76	3	4.68	0.56	1505.1	1	35/112	2.12E9
	115.74	-.YAALCDVFVM*DAFGTAHR.-	2061.30	2	3.43	0.46	415.3	1	14/34	2.28E9
	115.49 - 116.03	-.YAALCDVFVM*DAFGTAHR.-	2061.30	3	4.12	0.59	616.0	1	27/68	2.79E9
#4	ALF_ECOLI (P11604) I					320.33			32 (32 0 0 0 0)	7.71
	122.86 - 123.45	-.AFQELNAIDVL.-	1233.40	2	3.44	0.28	1526.3	1	17/20	3.35E9
	67.29 - 67.93	-.ANEAYLQGQLGNPK.-	1503.64	1	3.24	0.51	603.8	1	16/26	5.92E9
	68.90 - 70.09	-.ANEAYLQGQLGNPK.-	1503.64	2	3.66	0.52	1241.9	1	18/26	7.35E9
	67.15 - 68.28	-.ANEAYLQGQLGNPK.-	1503.64	2	5.35	0.59	1842.2	1	20/26	1.21E10
	110.59 - 111.31	-.APVIVQFSNGGASFIAGK.-	1764.02	2	5.17	0.58	2694.9	1	27/34	4.73E9
	106.31	-.APVIVQFSNGGASFIAGK.-	1764.02	1	3.45	0.63	689.2	1	19/34	1.34E9
	105.71 - 107.00	-.APVIVQFSNGGASFIAGK.-	1764.02	2	4.11	0.62	1455.6	1	23/34	1.40E10
	135.24 - 136.45	-.ENNFALPAVNCVGTDSINAVLETAAK.-	2719.98	2	3.49	0.46	635.9	1	20/50	6.66E9
	131.94 - 132.96	-.ENNFALPAVNCVGTDSINAVLETAAK.-	2719.98	2	3.77	0.58	712.9	1	21/50	1.13E10
	133.58 - 135.00	-.ENNFALPAVNCVGTDSINAVLETAAK.-	2719.98	3	5.06	0.55	1076.8	1	37/100	1.07E10
	133.47 - 134.64	-.ENNFALPAVNCVGTDSINAVLETAAK.-	2719.98	2	4.58	0.63	841.8	1	21/50	1.35E10
	119.01 - 119.56	-.FTIAASFGNVHGVYKPGNVVLTPTILR.-	2873.34	3	3.89	0.42	1658.1	1	34/104	3.29E9
	116.75	-.FTIAASFGNVHGVYKPGNVVLTPTILR.-	2873.34	2	4.29	0.62	737.4	1	19/52	2.36E9
	115.63 - 116.61	-.FTIAASFGNVHGVYKPGNVVLTPTILR.-	2873.34	3	6.57	0.62	2095.7	1	38/104	1.20E10
	117.19 - 118.07	-.FTIAASFGNVHGVYKPGNVVLTPTILR.-	2873.34	3	5.58	0.48	2520.9	1	41/104	1.20E10
	80.97	-.HNLPHNSLNFVFHGGSGSTAQEIK.-	2592.81	2	5.19	0.69	728.6	1	18/46	1.80E9
	100.95 - 102.07	-.IFDFVKPGVITGDDVQK.-	1879.15	2	4.83	0.60	1029.7	1	23/32	1.24E10
	101.00 - 102.18	-.IFDFVKPGVITGDDVQK.-	1879.15	3	3.95	0.13	937.6	1	31/64	1.07E10
	75.86 - 76.96	-.KHNLPHNSLNFVFHGGSGSTAQEIK.-	2720.98	3	4.80	0.44	1855.9	1	37/96	3.02E9
	76.00	-.KHNLPHNSLNFVFHGGSGSTAQEIK.-	2720.98	2	4.16	0.58	568.3	1	17/48	1.66E9
	124.09 - 125.43	-.KLLPWIDGLLDAGEK.-	1668.96	2	2.92	0.39	296.2	5	11/28	1.23E10
	131.04	-.KLLPWIDGLLDAGEK.-	1668.96	3	4.16	0.36	2344.6	1	33/56	1.58E9
	130.89 - 131.15	-.KLLPWIDGLLDAGEK.-	1668.96	2	4.83	0.52	939.0	1	21/28	4.34E9
	129.30	-.LLPWIDGLLDAGEK.-	1540.79	2	3.14	0.39	279.4	1	16/26	2.75E9

	141.33 - 141.89	-.LLPWIDGLLDAGEK.-	1540.79	2	4.25	0.42	875.2	1	22/26	8.67E9
	134.78 - 135.52	-.M*NIDTDTQWATWEGVLNYYK.-	2465.68	3	5.42	0.61	2700.2	1	36/76	5.63E9
	101.29 - 101.62	-.PGVITGDDVQK.-	1129.25	1	2.21	0.37	860.8	1	14/20	2.10E9
	113.20 - 114.13	-.PLFSSHM*IDLSEESLQENIEICSK.-	2824.10	3	5.10	0.59	1089.9	1	30/92	3.86E9
	11.18	-.VFQVAK.-	691.84	1	2.17	0.13	589.4	4	8/10	3.74E9
	102.86 - 103.43	-.VKAPVIVQFSNGGASFIAGK.-	1991.32	3	4.99	0.64	2711.1	1	38/76	5.76E9
	104.38 - 105.08	-.VKAPVIVQFSNGGASFIAGK.-	1991.32	2	4.40	0.63	1515.5	1	23/38	7.18E9
	105.10 - 106.43	-.VKAPVIVQFSNGGASFIAGK.-	1991.32	3	4.85	0.53	2376.8	1	36/76	3.15E9
#5	YNCE_ECO57 (Q8X9X					260.29			26 (26 0 0 0 0)	4.84
	59.20 - 60.89	-.AAEVLVVDTR.-	1073.23	1	2.18	0.34	717.4	1	12/18	2.80E9
	59.40 - 60.55	-.AAEVLVVDTR.-	1073.23	2	3.76	0.57	1557.8	1	16/18	1.36E9
	60.38	-.AAEVLVVDTR.-	1073.23	1	2.52	0.32	434.4	1	11/18	1.46E9
	61.11	-.AAEVLVVDTR.-	1073.23	2	3.34	0.47	1271.6	1	15/18	1.16E9
	85.69 - 86.26	-.AAEVLVVDTRNGNILAK.-	1784.05	2	4.95	0.54	1548.0	1	21/32	3.92E9
	104.31	-.DSVIWVVDGENIK.-	1474.64	1	4.34	0.56	837.3	1	15/24	1.87E9
	104.20	-.DSVIWVVDGENIK.-	1474.64	1	2.32	0.38	533.0	1	13/24	2.40E9
	103.03 - 104.18	-.DSVIWVVDGENIK.-	1474.64	2	4.87	0.49	1452.0	1	18/24	7.48E9
	91.42 - 92.56	-.ELVADDATNTVYISGIGK.-	1867.05	2	5.14	0.70	1711.8	1	23/34	4.49E9
	94.64	-.ELVADDATNTVYISGIGK.-	1867.05	2	5.20	0.74	1483.7	1	21/34	5.51E9
	93.14 - 94.13	-.ELVADDATNTVYISGIGK.-	1867.05	2	5.52	0.66	1719.8	1	22/34	1.13E10
	204.16 - 205.55	-.ELVADDATNTVYISGIGK.-	1867.05	2	4.05	0.60	685.0	1	15/34	2.19E8
	112.06 - 113.11	-.LLDDGKEHFFINISLDTAR.-	2205.46	2	3.40	0.53	988.0	1	17/36	1.94E9
	92.78	-.LYTTNADGELITIDTADNK.-	2069.21	2	4.73	0.69	1453.2	1	22/36	3.17E9
	88.82 - 90.15	-.LYTTNADGELITIDTADNK.-	2069.21	2	5.44	0.64	1613.0	1	22/36	1.32E10
	94.21 - 95.13	-.LYTTNADGELITIDTADNK.-	2069.21	2	5.00	0.55	1366.1	1	22/36	5.02E9
	90.04	-.LYTTNADGELITIDTADNK.-	2069.21	3	3.85	0.46	1693.1	1	30/72	1.98E9
	90.81 - 91.98	-.LYTTNADGELITIDTADNK.-	2069.21	2	5.74	0.60	1955.6	1	24/36	1.62E10
	50.55	-.MSTGLALDSK.-	1023.19	1	2.04	0.37	544.5	1	12/18	4.84E8
	84.20	-.RLYTTNADGELITIDTADNK.-	2225.40	2	3.05	0.39	473.6	2	16/38	1.73E9
	74.58 - 75.73	-.TFDTPTHPNSLALSADGK.-	1873.01	2	4.83	0.59	955.4	1	21/34	8.81E9
	76.30 - 77.17	-.TFDTPTHPNSLALSADGK.-	1873.01	2	4.74	0.57	949.5	1	23/34	8.74E9
	77.75	-.TFDTPTHPNSLALSADGK.-	1873.01	2	3.95	0.54	1008.4	1	22/34	2.34E9
	118.24	-.VAAPESLAVLFNPAR.-	1555.80	1	3.41	0.53	529.9	1	15/28	2.52E9
	118.13 - 118.76	-.VAAPESLAVLFNPAR.-	1555.80	1	3.19	0.45	638.0	1	16/28	3.63E9
	117.80 - 118.70	-.VAAPESLAVLFNPAR.-	1555.80	2	4.57	0.59	1448.5	1	18/28	1.89E10
#6	RPOA_ECOLI (P00574					250.27			25 (25 0 0 0 0)	2.40
	86.51 - 87.86	-.AEAIHYIGDLVQR.-	1485.67	2	3.83	0.38	1448.0	1	19/24	4.89E9
	88.09	-.AEAIHYIGDLVQR.-	1485.67	1	2.19	0.05	608.5	1	14/24	1.22E9
	88.07	-.AEAIHYIGDLVQR.-	1485.67	3	4.56	0.30	1830.2	1	32/48	9.68E8
	125.29 - 125.87	-.EEKPEFDPILLRPVDDLELTVR.-	2624.97	3	3.52	0.18	452.2	1	26/84	7.95E9
	165.03	-.EGVQEDILEILLNLK.-	1726.99	3	4.77	0.39	1713.6	1	28/56	8.06E8
	164.05 - 165.37	-.EGVQEDILEILLNLK.-	1726.99	2	4.68	0.56	1489.3	1	18/28	2.88E9
	62.70 - 63.66	-.GFGHTLGNALR.-	1143.28	1	2.26	0.32	259.5	1	13/20	8.50E8
	61.96 - 63.34	-.GFGHTLGNALR.-	1143.28	2	2.79	0.40	800.5	1	17/20	1.50E9
	64.20 - 64.71	-.GFGHTLGNALR.-	1143.28	2	2.81	0.48	798.1	1	15/20	1.44E9
	62.56 - 63.43	-.GFGHTLGNALR.-	1143.28	2	2.65	0.43	946.3	1	16/20	1.50E9
	11.95 - 12.53	-.IAYNVEAAR.-	1007.13	2	3.13	0.37	1037.6	1	14/16	2.36E9
	107.60	-.ILLSSM*PGCAVTEVEIDGVLHEYSTK.-	2866.23	3	4.18	0.52	955.2	1	40/100	2.54E9
	118.49 - 119.89	-.ILLSSM*PGCAVTEVEIDGVLHEYSTK.-	2866.23	3	3.78	0.41	657.7	1	37/100	4.78E9
	82.62 - 83.28	-.LLVDACYSPVER.-	1422.60	2	3.47	0.47	1356.9	1	17/22	4.64E9
	82.78	-.LLVDACYSPVER.-	1422.60	1	1.83	0.18	152.7	3	10/22	1.63E9
	58.09 - 58.69	-.LVDIEQVSSTHAK.-	1427.59	1	1.99	0.15	459.0	1	14/24	8.28E8
	57.82 - 58.93	-.LVDIEQVSSTHAK.-	1427.59	2	4.19	0.58	1709.5	1	20/24	1.23E9
	94.41 - 95.02	-.LVIEM*ETNGTIDPEEAIR.-	2047.27	2	5.30	0.45	1741.5	1	23/34	3.56E9
	73.22 - 74.61	-.M*QGSVTEFLKPR.-	1409.64	2	3.10	0.51	1021.1	1	17/22	3.52E9
	88.36	-.SLTEIKDVLASR.-	1332.53	1	1.91	0.32	410.7	1	13/22	1.26E9
	88.15	-.SLTEIKDVLASR.-	1332.53	2	3.61	0.49	1657.3	1	19/22	3.65E9
	106.02 - 107.37	-.TDLDKLVIEM*ETNGTIDPEEAIR.-	2619.89	3	3.78	0.46	676.7	1	26/88	5.42E9
	106.17	-.TDLDKLVIEM*ETNGTIDPEEAIR.-	2619.89	2	3.83	0.54	890.7	1	22/44	1.98E9
	71.22 - 72.60	-.VQGKDEVILTLNK.-	1457.70	2	4.76	0.57	1696.5	1	21/24	2.14E9
	72.87	-.VQGKDEVILTLNK.-	1457.70	2	4.18	0.43	1404.5	1	19/24	2.24E9
#7	Q8XDF1 (Q8XDF1) Ou					240.28			24 (24 0 0 0 0)	3.35
	61.38 - 62.30	-.AEQWATGLK.-	1004.12	1	1.93	0.39	540.0	1	12/16	1.10E9
	62.77 - 63.89	-.AVGLHYFSK.-	1022.18	2	2.99	0.47	1152.7	1	15/16	1.55E9
	69.94 - 70.49	-.FTNISGFANK.-	1099.22	1	2.40	0.46	591.6	1	13/18	6.01E9
	69.96 - 70.51	-.FTNISGFANK.-	1099.22	2	3.05	0.47	1254.7	1	16/18	3.25E9
	70.14	-.FTNISGFANK.-	1099.22	1	2.38	0.43	603.7	1	13/18	2.80E9
	15.22 - 16.50	-.KAEQWATGLK.-	1132.29	2	2.92	0.38	837.1	1	14/18	9.60E8
	17.26 - 18.21	-.KAEQWATGLK.-	1132.29	2	3.29	0.40	1050.2	1	15/18	2.83E8
	13.52 - 14.71	-.KAEQWATGLK.-	1132.29	2	2.60	0.46	743.2	1	14/18	9.82E8
	123.36	-.NM*STYVDYIINQIDSDNK.-	2150.31	2	4.91	0.61	1172.2	1	18/34	1.50E9
	127.10	-.NM*STYVDYIINQIDSDNK.-	2150.31	3	3.58	0.45	1434.4	1	28/68	1.62E9
	126.86 - 127.41	-.NM*STYVDYIINQIDSDNK.-	2150.31	2	4.92	0.62	1374.2	1	21/34	6.38E9
	144.42 - 145.32	-.NMSTYVDYIINQIDSDNK.-	2134.31	2	5.16	0.68	1314.2	1	19/34	3.62E9

	159.62 - 160.19	-.NSNFFGLVDGLNFAVQYLGK.-	2204.47	2	5.23	0.43	1469.0	1	23/38	1.95E9
	149.07 - 149.13	-.NSNFFGLVDGLNFAVQYLGK.-	2204.47	2	4.82	0.48	870.9	1	21/38	1.89E9
	72.83 - 74.01	-.TNLQEAQLLGNGK.-	1386.54	2	4.17	0.49	2547.8	1	21/24	7.62E9
	62.42 - 63.02	-.TNLQEAQLLGNGKK.-	1514.71	3	3.64	0.41	1104.0	1	28/52	1.33E9
	62.68	-.TNLQEAQLLGNGKK.-	1514.71	1	2.25	0.44	205.4	1	14/26	6.14E8
	62.05 - 63.20	-.TNLQEAQLLGNGKK.-	1514.71	2	3.68	0.52	1740.4	1	20/26	2.21E9
	155.93	-.VGGVATYRNSNFFGLVDGLNFAVQYLGK.-	3008.38	3	4.06	0.52	870.3	1	30/108	1.03E9
	74.85 - 75.40	-.YADVGSFDYGR.-	1250.30	1	2.26	0.47	313.6	1	13/20	3.99E9
	74.75 - 76.02	-.YADVGSFDYGR.-	1250.30	2	3.14	0.55	1181.5	1	17/20	8.94E9
	86.07 - 87.27	-.YDANNIYLAANYGETR.-	1848.95	2	5.66	0.60	1991.2	1	25/30	9.90E9
	89.65 - 91.03	-.YDANNIYLAANYGETR.-	1848.95	2	5.05	0.59	1021.6	1	18/30	9.00E9
	87.90 - 88.99	-.YDANNIYLAANYGETR.-	1848.95	2	5.20	0.56	1413.8	1	23/30	1.32E10
#8	EFTU_ECOLI (P02990					180.23			18 (18 0 0 0 0)	2.43
	48.84 - 49.54	-.AFDQIDNAPEEK.-	1377.44	2	2.91	0.45	868.3	1	15/22	8.26E8
	47.16 - 47.73	-.AFDQIDNAPEEK.-	1377.44	2	2.93	0.44	1097.1	1	16/22	3.59E8
	74.48 - 75.06	-.AGENVGVLLR.-	1028.19	1	1.94	0.14	267.7	4	11/18	2.67E9
	74.52 - 75.12	-.AGENVGVLLR.-	1028.19	2	3.12	0.03	719.1	2	14/18	1.83E9
	141.47 - 142.01	-.AIDKPFLLPIEDVFSISGR.-	2118.46	2	4.32	0.62	753.8	1	20/36	3.94E9
	141.51 - 142.10	-.AIDKPFLLPIEDVFSISGR.-	2118.46	3	4.48	0.49	1562.0	1	34/72	6.50E9
	151.60 - 152.23	-.CDMVDDEELLELVEM*EVR.-	2241.47	2	3.67	0.27	1411.2	1	20/34	2.00E9
	107.68 - 108.72	-.ELLSQYDFPGDDTPIVR.-	1966.14	2	4.32	0.63	510.4	1	17/32	1.33E10
	87.67	-.FESEVYILSK.-	1215.38	2	3.27	0.47	851.9	1	16/18	1.59E9
	72.25 - 72.97	-.GITINTSHVEYDTPTR.-	1804.94	2	3.96	0.65	874.7	1	17/30	2.53E9
	72.39 - 72.46	-.GITINTSHVEYDTPTR.-	1804.94	3	3.41	0.53	577.2	2	21/60	1.09E9
	79.27 - 79.89	-.GYRPQFYFR.-	1234.39	2	2.51	0.28	549.2	2	12/16	2.09E9
	143.21 - 144.44	-.ILELAGFLDSYIPEPER.-	1963.22	2	4.63	0.47	883.5	1	21/32	1.31E10
	143.64	-.ILELAGFLDSYIPEPER.-	1963.22	3	3.44	0.35	1100.1	1	32/64	1.46E9
	89.07 - 89.60	-.M*VVTLIHPIAM*DDGLR.-	1814.16	2	3.24	0.42	581.1	1	17/30	2.24E9
	102.05 - 102.68	-.TTDVTGTIELPEGVEM*VM*PGDNIK.-	2579.88	2	2.93	0.55	186.3	1	13/46	5.56E9
	114.94 - 116.31	-.TTLTAAITTVLAK.-	1304.56	2	3.63	0.25	2104.2	1	20/24	3.16E9
	81.76 - 82.39	-.VGEEVEIVGIK.-	1172.35	2	3.75	0.44	1711.9	1	17/20	2.55E9
#9	FTSZ_ECOLI (P06138)					160.25			16 (16 0 0 0 0)	1.13
	131.82	-.DLGILTVAVVTKPFNFEGK.-	2049.40	2	4.75	0.61	766.2	1	17/36	1.96E9
	135.74 - 136.33	-.GISLLDAFGAANDVLK.-	1604.83	2	4.97	0.64	1302.9	1	21/30	3.80E9
	75.46	-.HVDSLITIPNDK.-	1352.52	2	3.85	0.64	1243.9	1	18/22	1.73E9
	75.56 - 75.61	-.HVDSLITIPNDK.-	1352.52	1	2.66	0.46	470.9	1	14/22	9.56E8
	114.82 - 116.07	-.IEGVEFFAVNTDAQALR.-	1881.08	2	3.12	0.34	810.6	1	15/32	4.63E9
	71.01 - 72.35	-.KTAVGQTIQIGSGITK.-	1602.86	2	3.69	0.53	1212.8	1	20/30	2.00E9
	85.62	-.LDEFETVGNTIR.-	1394.51	2	4.30	0.53	1533.8	1	19/22	2.27E9
	79.85 - 81.11	-.M*AFAEQGITELSK.-	1441.63	2	4.30	0.60	1684.6	1	20/24	3.50E9
	91.19	-.M*FEPM*ELTNDAVIK.-	1670.93	2	3.22	0.54	543.9	1	17/26	3.60E9
	29.98 - 31.20	-.RPEITLVTNK.-	1171.37	2	2.59	0.47	1167.6	1	15/18	7.46E8
	30.92 - 32.36	-.RPEITLVTNK.-	1171.37	2	2.91	0.42	956.2	1	15/18	9.32E8
	32.88 - 34.48	-.RPEITLVTNK.-	1171.37	2	2.96	0.51	810.2	1	14/18	5.70E8
	79.44 - 80.65	-.TAVGQTIQIGSGITK.-	1474.69	2	3.46	0.54	952.9	1	16/28	2.50E9
	79.60	-.TAVGQTIQIGSGITK.-	1474.69	1	1.82	0.15	145.8	23	9/28	8.44E8
	45.15 - 46.65	-.VTVVATGIGM*DK.-	1207.42	2	3.09	0.45	1347.1	1	17/22	4.02E8
	46.81 - 48.22	-.VTVVATGIGM*DK.-	1207.42	2	2.88	0.45	1537.4	1	17/22	4.27E8
#10	K6P1_ECOLI (P06998)					160.24			16 (16 0 0 0 0)	1.27
	34.26 - 42.43	-.AVAIENLK.-	858.02	1	2.05	0.08	509.2	2	9/14	7.92E8
	33.35 - 40.29	-.AVAIENLK.-	858.02	1	2.28	0.17	416.7	2	10/14	6.23E8
	66.45	-.EDLVNEIK.-	960.06	1	1.98	0.24	280.4	3	9/14	1.11E9
	75.29 - 75.92	-.FPEFRDENIR.-	1323.44	2	2.82	0.19	840.2	1	14/18	2.48E9
	75.96	-.IGVLTSGGDAPGM*NAAIR.-	1716.94	2	4.33	0.62	809.9	1	20/34	4.52E9
	74.27 - 75.42	-.IGVLTSGGDAPGM*NAAIR.-	1716.94	2	4.27	0.60	498.7	1	16/34	4.52E9
	86.42 - 87.05	-.IGVLTSGGDAPGMNAAIR.-	1700.94	2	3.06	0.58	446.7	1	15/34	4.58E9
	43.85 - 45.52	-.ISVVEVM*GR.-	1006.20	2	2.64	0.50	710.6	2	13/16	3.93E8
	37.88 - 39.07	-.ISVVEVM*GR.-	1006.20	2	2.67	0.54	714.5	1	13/16	2.70E8
	41.94 - 43.31	-.ISVVEVM*GR.-	1006.20	2	2.65	0.34	749.4	2	13/16	4.35E8
	39.62 - 41.23	-.ISVVEVM*GR.-	1006.20	2	2.64	0.46	832.0	1	14/16	3.26E8
	35.88 - 37.17	-.ISVVEVM*GR.-	1006.20	2	2.67	0.43	713.3	1	13/16	2.22E8
	46.13 - 47.18	-.ISVVEVM*GR.-	1006.20	2	2.76	0.33	885.5	1	14/16	3.98E8
	116.84 - 117.96	-.LTEM*GFPCIGLPGTIDNDIK.-	2208.51	2	4.73	0.50	741.2	1	21/38	7.85E9
	125.81 - 125.93	-.LTEMGFPCIGLPGTIDNDIK.-	2192.51	2	4.06	0.54	505.9	1	17/38	2.71E9
	125.10 - 126.34	-.M*GAYAIDLLLAGYGGR.-	1657.92	2	4.44	0.65	2009.1	1	22/30	3.67E9
#11	Q8X5N8 (Q8X5N8) Pu					140.34			14 (14 0 0 0 0)	1.70
	73.42 - 73.93	-.ADATVVEQEWR.-	1304.39	2	2.99	0.39	1106.9	1	17/20	3.03E9
	76.08 - 77.30	-.DIAGLM*NIR.-	1019.20	2	2.68	0.43	660.8	1	12/16	1.52E9
	102.28 - 102.99	-.EILAALESVGETK.-	1360.54	2	4.02	0.57	2263.0	1	21/24	5.83E9
	90.68 - 91.24	-.FITDENTPLELK.-	1420.59	2	3.70	0.42	1032.5	1	18/22	5.95E9
	90.93	-.FITDENTPLELK.-	1420.59	1	2.36	0.28	381.6	1	11/22	1.88E9
	88.57	-.GCVQIFTGVVEK.-	1337.54	1	1.88	0.29	463.3	1	12/22	1.60E9
	88.26 - 88.89	-.GCVQIFTGVVEK.-	1337.54	2	2.82	0.39	1078.5	1	16/22	3.82E9
	129.93	-.KPTSDGYVTSLELFAHDGTQIAQLYGQR.-	3097.38	3	5.46	0.55	1452.9	1	33/108	2.74E9

	128.24 - 128.47	-.KPTSDGYVTSLELFAHDGTQIAQLYGQR.-	3097.38	3	6.82	0.59	2567.9	1	38/108		3.36E9
	91.64	-.NEYAVHEQVGTFTNQHLNGHAGLILNPR.-	3131.41	3	5.17	0.64	1519.4	1	33/108		2.21E9
	148.55 - 150.07	-.VSNSALAQILESAQQDGNEIM*VFVGNR.-	2908.19	3	5.84	0.65	1712.5	1	35/104		4.37E9
	149.71 - 149.78	-.VSNSALAQILESAQQDGNEIM*VFVGNR.-	2908.19	2	4.75	0.61	606.4	1	16/52		1.39E9
	111.02 - 112.21	-.VYATDNTDM*AAWSELLAR.-	2044.23	2	5.60	0.63	1627.5	1	21/34		6.37E9
	125.08 - 125.22	-.VYATDNTDMAAWSELLAR.-	2028.23	2	4.54	0.59	1147.3	1	19/34		2.55E9
#12	OMPT_ECO57 (P5860					140.20			14 (14 0 0 0 0)		1.03
	103.96 - 104.50	-.GGSYIYSSEEGFRDDIGSFPNGER.-	2640.72	2	2.53	0.27	223.0	1	13/46		2.38E9
	91.90 - 93.13	-.GWLLNEPNYR.-	1262.40	2	2.66	0.32	997.9	1	14/18		3.50E9
	99.51 - 100.41	-.HPDTQLNYANEFDLNIK.-	2033.19	2	2.63	0.33	309.9	3	12/32		3.48E9
	13.43 - 14.02	-.KVSQLDWK.-	1004.17	2	2.56	0.30	701.8	1	13/14		1.07E9
	48.01 - 49.45	-.LGLM*AGYQESR.-	1241.40	2	3.40	0.45	1289.2	1	18/20		1.21E9
	51.88	-.LGLM*AGYQESR.-	1241.40	2	2.78	0.50	919.4	1	15/20		9.03E8
	50.02 - 51.19	-.LGLM*AGYQESR.-	1241.40	2	3.58	0.51	1124.7	1	17/20		1.63E9
	78.33	-.M*PYIGLTGSYR.-	1274.47	2	2.74	0.44	812.5	1	14/20		1.78E9
	111.77 - 112.56	-.NGAGIENYNFITTAGLK.-	1783.96	2	3.92	0.61	710.1	2	18/32		3.71E9
	11.29 - 12.11	-.VYLAEEGGR.-	994.08	1	2.10	0.27	511.1	1	12/16		1.35E9
	11.27	-.VYLAEEGGR.-	994.08	2	2.70	0.45	1135.1	1	15/16		2.02E9
	93.47 - 94.07	-.YEDFELGGTFK.-	1306.40	2	3.62	0.63	1369.5	1	18/20		3.69E9
	65.89	-.YSGWVEASDNDEHYDPGKR.-	2226.26	2	3.64	0.48	431.5	1	18/36		7.63E8
	68.36	-.YSGWVEASDNDEHYDPGKR.-	2226.26	2	3.75	0.58	394.6	1	17/36		8.80E8
#13	DCEA_ECO57 (P5822					120.27			12 (12 0 0 0 0)		1.01
	140.51	-.FGLAPLGCGWVIWR.-	1632.93	2	2.95	0.49	651.4	1	17/26	1	1.53E9
	142.61 - 143.44	-.GFEM*DFAELLLEDYK.-	1837.04	2	3.41	0.51	962.2	1	15/28	1	2.30E9
	128.53 - 129.12	-.GWQVPAFTLGGEATDIVVM*R.-	2164.47	2	5.45	0.64	1715.7	1	27/38	1	2.21E9
	115.03 - 115.86	-.LGPYEFICTGRPDEGIPAVCFK.-	2527.84	3	4.30	0.56	1598.3	1	33/84	1	4.91E9
	115.33	-.LGPYEFICTGRPDEGIPAVCFK.-	2527.84	2	3.55	0.55	329.1	1	15/42	1	2.01E9
	90.31	-.LKEGEDPGYTLYDLSER.-	1986.13	2	4.02	0.56	299.8	1	16/32		1.38E9
	49.21 - 50.42	-.LQGIAQQNSFK.-	1234.39	2	3.49	0.58	708.2	1	15/20	1	7.32E8
	47.26 - 48.61	-.LQGIAQQNSFK.-	1234.39	2	3.35	0.55	910.7	1	16/20	1	4.82E8
	49.49 - 50.22	-.LQGIAQQNSFK.-	1234.39	1	2.17	0.49	402.0	1	11/20	1	3.75E8
	117.53	-.PAGQVIAQYYEFLR.-	1655.88	2	4.72	0.57	1588.0	1	20/26	1	4.48E9
	121.27 - 122.26	-.VQNASYQVAAYLADEIAK.-	1955.16	2	5.34	0.59	2366.9	1	26/34	1	6.19E9
	121.49	-.VQNASYQVAAYLADEIAK.-	1955.16	3	5.17	0.54	2363.4	1	33/68	1	1.21E9
#14	Q8XEB4 (Q8XEB4) For					120.23			12 (12 0 0 0 0)		0.89
	121.11	-.DAIPTQSVLTITSNVVYGK.-	2007.27	2	2.99	0.60	397.6	1	15/36		3.65E9
	68.40	-.GDWQNEVNVR.-	1217.27	2	3.45	0.14	1494.3	1	16/18		1.11E9
	92.12 - 93.03	-.ITEQEAQEM*VDHLVM*K.-	1934.18	2	2.87	0.14	313.0	2	14/30		2.85E9
	85.16	-.SEPIKGDLLNYDEVM*ER.-	2025.23	2	3.01	0.57	902.4	1	18/32		1.38E9
	87.50	-.SGVLTGLPDAYGR.-	1306.45	2	2.83	0.19	833.3	1	16/24		1.89E9
	101.23 - 102.30	-.THAPVDFDTAVASTITSHDAGYINK.-	2632.82	3	3.96	0.62	412.5	1	30/96		2.30E9
	115.37	-.TM*ACGIAGLSVAADSLSAIK.-	1953.24	2	2.67	0.48	368.8	4	13/38		1.95E9
	133.51	-.TPEYDELFSGDPIWATESIGGM*GLDGR.-	2931.14	2	2.57	0.52	129.6	25	11/52		2.58E9
	105.90	-.TSTFLDVYIER.-	1344.50	2	2.65	0.37	763.9	1	13/20		1.61E9
	100.89	-.VALYGIDYLM*K.-	1302.56	2	3.14	0.43	973.3	1	16/20		1.60E9
	114.67	-.VALYGIDYLMK.-	1286.57	2	2.94	0.40	679.0	1	16/20		8.68E8
	93.63	-.VDDLAVDLVER.-	1244.38	2	4.54	0.54	1683.7	1	18/20		2.48E9
#15	DHAS_ECOLI (P00353					110.26			11 (11 0 0 0 0)		1.27
	105.27	-.ALDIIVTCQGGDYTNEIYPK.-	2271.50	2	5.29	0.68	1377.1	1	22/38		2.43E9
	140.42	-.DVSIPTVEELLAAHNPWAK.-	2091.35	2	2.93	0.54	338.2	1	14/36		1.99E9
	116.56	-.ELLTQM*GHLYGHVADELATPSSAILDIER.-	3197.57	3	4.26	0.49	1001.3	1	35/112		1.71E9
	134.31	-.ELLTQMGHLYGHVADELATPSSAILDIER.-	3181.57	3	4.09	0.41	324.0	16	23/112		1.94E9
	96.19 - 97.03	-.ELTPAAVTGTLTTPVGR.-	1684.92	2	3.56	0.52	815.0	1	17/32		5.16E9
	126.03	-.ESGWQGYWIDAASSLR.-	1826.95	2	4.23	0.57	1043.8	1	18/30		3.04E9
	103.01 - 103.05	-.ILNTSSVIPVDGLCVR.-	1744.02	2	2.88	0.40	303.7	1	14/30		2.99E9
	104.90 - 106.13	-.ILNTSSVIPVDGLCVR.-	1744.02	2	3.45	0.58	249.7	1	12/30		5.88E9
	153.55	-.LNM*GPEFLSAFTVGDQLLWGAAEPLRR.-	3006.43	3	3.33	0.48	389.9	4	26/104		1.59E9
	120.54 - 121.55	-.M*KDDAIIILDPVNQDVITDGLNNGIR.-	2870.23	3	4.78	0.43	2121.4	1	38/100		6.55E9
	121.62	-.M*KDDAIIILDPVNQDVITDGLNNGIR.-	2870.23	2	3.26	0.51	360.6	1	18/50		1.55E9
#16	TALA_ECOLI (P78258)					100.27			10 (10 0 0 0 0)		0.55
	134.21	-.AAGLSQYEHLIDDAIAWGK.-	2059.27	2	5.38	0.56	1770.0	1	20/36		2.06E9
	140.20 - 140.93	-.EGINCNLTLLFSFAQAR.-	1955.20	2	3.77	0.55	600.4	1	15/32	1	3.85E9
	65.44	-.HLVDLYQQQGVEK.-	1557.73	1	2.21	0.42	421.3	2	12/24		3.36E8
	65.09 - 65.40	-.HLVDLYQQQGVEK.-	1557.73	2	4.29	0.59	1505.9	1	21/24		1.24E9
	71.67 - 72.37	-.HYHPQDATTNPSLLLK.-	1836.04	2	3.32	0.53	701.2	1	18/30		1.57E9
	71.78	-.KLEDLLAAK.-	1001.20	2	2.61	0.18	742.0	1	14/16		6.01E8
	71.61	-.KLEDLLAAK.-	1001.20	1	1.88	0.12	583.6	1	12/16		9.96E8
	56.92 - 57.50	-.KPM*DPYVVEEDPGVK.-	1719.94	2	3.48	0.50	624.2	1	17/28		1.23E9
	70.83	-.KPMDPYVVEEDPGVK.-	1703.94	2	2.97	0.27	427.4	1	15/28		1.21E9
	73.28	-.RTEQILALTGCDR.-	1533.71	2	4.26	0.48	1104.3	1	18/24		1.97E9
#17	SERC_ECO57 (Q8XE/					100.26			10 (10 0 0 0 0)		0.96
	117.09 - 118.17	-.AELLYGVIDNSDFYR.-	1775.94	2	3.20	0.41	616.6	2	14/28		4.10E9
	101.97 - 102.01	-.ALTDFM*VEFER.-	1374.54	2	3.18	0.52	883.9	1	13/20		2.75E9
	125.76 - 126.47	-.AQIFNFSSGPAM*LPVEVLK.-	2065.42	2	4.64	0.63	798.5	1	16/36		5.57E9

	135.66	-.AQIFNFSSGPAMLPVEVLK.-	2049.42	2	4.75	0.52	1090.8	1	18/36	1.63E9
	112.31 - 112.83	-.GQFAAVPLNILGDK.-	1443.67	2	2.67	0.48	462.3	1	15/26	4.10E9
	151.72	-.M*NVPFQLADSALDKLFLEESFAAGLHALK.-	3193.66	3	4.55	0.60	371.8	1	30/112	1.68E9
	108.15 - 108.74	-.NIGPAGLTIVIVR.-	1323.61	2	3.56	0.50	818.5	1	16/24	2.94E9
	109.68 - 110.07	-.TTADYVDAGYWAASAIK.-	1803.95	2	5.29	0.63	1139.0	1	20/32	2.79E9
	39.34 - 43.02	-.YGVIYAGAQK.-	1070.22	1	2.21	0.34	582.2	1	11/18	3.28E8
	40.66 - 42.05	-.YGVIYAGAQK.-	1070.22	2	3.38	0.64	1145.3	1	16/18	3.95E8
#18	PUR5_ECO57 (Q8XAC					100.25			10 (10 0 0 0 0)	1.07
	84.52	-.DAGVDIDAGNALVGR.-	1443.55	2	3.84	0.27	1831.3	1	21/28	2.46E9
	111.33	-.TFNCGVGM*IIALPAPEVDK.-	2049.37	2	3.52	0.60	605.8	1	17/36	3.52E9
	109.43 - 110.73	-.TFNCGVGM*IIALPAPEVDK.-	2049.37	2	3.31	0.48	454.6	1	15/36	5.84E9
	119.18	-.TFNCGVGMIIALPAPEVDK.-	2033.37	2	2.78	0.40	536.3	1	14/36	2.36E9
	126.28	-.VDVNAIAHLTGGGFWENIPR.-	2167.41	2	3.77	0.54	730.3	1	18/38	2.92E9
	109.66	-.VSDGDVLIALGSSGPHSNGYSLVR.-	2401.62	3	3.53	0.46	2362.5	1	39/92	4.54E9
	108.42 - 109.22	-.VSDGDVLIALGSSGPHSNGYSLVR.-	2401.62	2	2.53	0.51	530.0	1	15/46	2.88E9
	67.99 - 68.78	-.YREPVLVSGTDGVGTK.-	1678.87	2	4.77	0.62	1604.3	1	23/30	2.88E9
	66.06 - 66.57	-.YREPVLVSGTDGVGTK.-	1678.87	2	4.98	0.59	1332.9	1	22/30	1.05E9
	68.32 - 68.94	-.YREPVLVSGTDGVGTK.-	1678.87	3	3.31	0.45	1819.0	1	29/60	9.41E8
#19	Q8XAA9 (Q8XAA9) Pu					90.28			9 (9 0 0 0 0)	0.33
	96.55	-.ALNADDGKEIWSVSLAEK.-	1947.14	2	4.82	0.66	1447.7	1	21/34	1.38E9
	100.43	-.EPALLSGGVTVSGGHVYIGSEK.-	2158.40	2	5.66	0.65	1590.2	1	24/42	1.57E9
	136.82 - 137.69	-.LLTSPVLYNGNLVVGDSEGYLHWINVEDGR.-	3331.68	3	3.78	0.50	567.7	1	29/116	3.61E9
	46.67 - 47.39	-.VDSSGFQTEPVAADGK.-	1608.69	2	4.55	0.63	1358.0	1	20/30	3.64E8
	48.35	-.VDSSGFQTEPVAADGK.-	1608.69	2	2.57	0.48	702.9	1	16/30	4.11E8
	44.86 - 45.40	-.VDSSGFQTEPVAADGK.-	1608.69	2	4.29	0.60	1174.8	1	19/30	6.56E8
	42.62 - 44.29	-.VDSSGFQTEPVAADGK.-	1608.69	2	4.80	0.63	1335.5	1	20/30	5.85E8
	40.83 - 42.03	-.VDSSGFQTEPVAADGK.-	1608.69	2	4.08	0.56	1082.7	1	19/30	3.35E8
	39.01	-.VDSSGFQTEPVAADGK.-	1608.69	2	3.18	0.47	499.2	1	14/30	1.89E8
#20	Q7DBF3 (Q7DBF3) Pe					90.23			9 (9 0 0 0 0)	0.52
	38.02 - 41.38	-.EIADIYK.-	851.97	1	1.98	0.27	198.0	1	9/12	2.18E8
	88.22	-.EKEYVNECLDSTWISSK.-	2089.24	2	4.65	0.56	1159.1	1	19/32	1.21E9
	79.91 - 81.24	-.LIETRPVFYPVHTM*PM*YSEK.-	2471.88	3	3.34	0.27	577.8	4	25/76	2.40E9
	51.02	-.NINSLVQVHK.-	1152.33	1	1.97	0.42	278.8	1	12/18	4.58E8
	50.44 - 51.59	-.NINSLVQVHK.-	1152.33	2	3.11	0.35	580.2	1	13/18	7.58E8
	112.50 - 113.09	-.NLFVIEDCAEAFGSK.-	1700.86	2	4.30	0.53	1652.9	1	20/28	3.41E9
	72.43	-.TITTGEGGM*VVTNDKTLYDR.-	2188.40	2	4.22	0.60	624.3	1	19/38	1.11E9
	114.90 - 115.70	-.YTGATPIFVDSDNETWQM*SVSDIEQK.-	2979.18	2	3.71	0.55	471.1	1	15/50	1.57E9
	132.57 - 133.11	-.YVGTFGDISTFSFFGNK.-	1888.07	2	4.08	0.64	879.8	1	18/32	3.18E9
#21	Q8XAS6 (Q8XAS6) Hy					70.28			7 (7 0 0 0 0)	0.68
	120.96	-.GEATADAAQSDALLSLGGAITAYK.-	2295.49	2	4.40	0.62	1074.4	1	21/46	2.57E9
	118.74 - 119.28	-.GM*DQYADQLYTDVVDLQK.-	2119.30	2	5.58	0.68	2061.1	1	23/34	4.09E9
	133.01	-.GMDQYADQLYTDVVDLQK.-	2103.30	2	4.91	0.60	1167.9	1	20/34	1.99E9
	150.09 - 151.22	-.IEPIAELFSDLDGSIDAR.-	1962.15	2	4.66	0.61	1547.9	1	21/34	1.99E9
	139.09	-.IEPIAELFSDLDGSIDAR.-	1962.15	2	4.78	0.64	1874.5	1	22/34	2.87E9
	82.51	-.IVDLLRPQLQK.-	1323.61	2	3.27	0.57	1583.7	1	16/20	1.46E9
	108.38 - 108.97	-.VVGGAAGLIEEVAASK.-	1471.68	2	4.28	0.56	1286.6	1	20/30	3.74E9
#22	ENO_ECOLI (P08324)					70.26			7 (7 0 0 0 0)	0.90
	142.35	-.AFTSEEFTHFLEELTK.-	1930.10	2	5.12	0.63	1460.1	1	19/30	2.07E9
	124.99	-.AKGM*NTAVGDEGGYAPNLGSNAEALAVIAE	3206.53	3	4.90	0.53	1043.0	1	36/128	2.45E9
	118.15 - 119.22	-.FNQIGSLTETLAAIK.-	1606.85	2	4.33	0.45	1171.5	1	22/28	5.18E9
	131.62	-.GM*NTAVGDEGGYAPNLGSNAEALAVIAEAV	3007.28	2	3.76	0.64	147.3	1	14/60	1.93E9
	84.12 - 85.14	-.GM*PLYEHIAELNGTPGK.-	1844.08	2	2.82	0.46	418.6	1	14/32	4.53E9
	106.88 - 108.19	-.IQLVGDDLFVTNTK.-	1563.78	2	3.66	0.56	968.1	1	18/26	3.89E9
	108.47 - 109.85	-.SGETEDATIADLAVGTAAGQIK.-	2119.27	2	5.08	0.48	1546.2	1	24/42	4.49E9
#23	EFG_ECOLI (P02996)					70.26			7 (7 0 0 0 0)	0.56
	77.47 - 78.06	-.EFNVEANVGKPQVAYR.-	1822.01	2	4.11	0.56	718.2	1	17/30	1.49E9
	151.45 - 152.12	-.LGANPVPLQLAIGAEEHFTGVVDLVK.-	2689.10	3	5.01	0.51	491.7	1	31/100	1.27E9
	90.08	-.LHFGSYHDVDSSELAFK.-	1953.10	2	3.62	0.62	1034.1	1	19/32	1.50E9
	107.10 - 108.36	-.M*EFPEPVISIAVEPK.-	1702.99	2	3.49	0.36	274.4	3	14/28	4.24E9
	95.88 - 96.42	-.VEVETPEENTGDVIGDLSR.-	2060.16	2	5.10	0.63	684.3	1	21/36	3.18E9
	95.49	-.YDEAPSNVAQAVIEAR.-	1733.86	2	4.63	0.61	1266.6	1	23/30	2.26E9
	84.81	-.YLGGEELTEAEIK.-	1452.59	2	3.63	0.61	986.2	1	20/24	1.52E9
#24	ADHE_ECOLI (P17547					70.24			7 (7 0 0 0 0)	0.46
	86.47 - 87.21	-.AAYSSGKPAIGVGAGNTPVVIDETADIKR.-	2859.19	3	4.87	0.55	1439.1	1	39/112	2.79E9
	86.88	-.AAYSSGKPAIGVGAGNTPVVIDETADIKR.-	2859.19	2	3.35	0.51	526.6	1	17/56	6.33E8
	129.58	-.EAGVQEADFLANVDKLSEDAFDDQCTGANP	3384.52	3	4.33	0.47	664.2	1	28/120	1.23E9
	63.37 - 64.51	-.FATHGGYLLQGK.-	1292.47	2	3.70	0.34	1124.3	1	19/22	1.39E9
	111.29 - 112.71	-.ILINTPASQGGIGDLYNFK.-	2022.29	2	4.80	0.70	799.2	1	21/36	4.21E9
	56.74	-.NAIIFSPHPR.-	1152.33	2	2.68	0.40	928.4	1	14/18	7.15E8
	78.63 - 79.34	-.NHFASEYIYNAYKDEK.-	1993.12	3	4.11	0.56	1215.2	1	26/60	1.77E9
#25	MALE_ECOLI (P02928					60.30			6 (6 0 0 0 0)	0.31
	122.52	-.EFLENYLLTDEGLEAVNK.-	2098.30	2	6.09	0.57	1605.1	1	23/34	2.05E9
	114.26	-.GEIM*PNIPQM*SAFWYAVR.-	2143.47	2	3.19	0.49	334.9	1	16/34	1.24E9
	159.79 - 161.04	-.LIAYPIAVEALSLIYNK.-	1892.27	2	5.04	0.70	1514.9	1	24/32	1.49E9

	160.73	-.LIAYPIAVEALSLIYNK.-	1892.27	3	5.41	0.63	3586.9	1	39/64	5.80E8
	101.83	-.VNYGVTVLPTFK.-	1338.58	1	2.26	0.24	658.2	1	12/22	1.78E9
	101.73	-.VNYGVTVLPTFK.-	1338.58	2	2.73	0.31	919.2	1	15/22	1.40E9
#26	TALB_ECOLI (P30148)					60.22			6 (6 0 0 0 0)	0.44
	11.32	-.ELAESEGAIER.-	1204.27	2	2.53	0.22	1276.9	1	17/20	1.96E9
	86.24	-.ITESEFLWQHNQDPM*AVDK.-	2305.51	2	3.13	0.41	345.9	1	16/36	1.64E9
	70.07	-.LASTWQGIR.-	1032.18	2	2.65	0.31	897.8	1	14/16	8.44E8
	115.51 - 116.05	-.LYQPQDATTNPSLILNAAQIPEYR.-	2718.02	2	3.26	0.50	222.8	2	12/46	3.58E9
	112.02	-.NIGEILELAGCDR.-	1460.61	2	3.64	0.57	1703.1	1	19/24	2.04E9
	75.69 - 76.44	-.QYTTVVADTGDIAAM*K.-	1700.89	2	4.44	0.60	937.9	1	19/30	2.10E9
#27	HLDD_ECO57 (P6791					60.22			6 (6 0 0 0 0)	0.63
	68.20	-.EYEKPLNVYGYSK.-	1590.76	2	3.52	0.60	591.0	1	19/24	1.01E9
	105.20	-.FLFDEYVR.-	1089.23	2	2.76	0.43	413.1	2	12/14	1.03E9
	111.35	-.GITDILVVDNLK.-	1300.53	2	3.67	0.53	1447.0	1	17/22	1.57E9
	34.14 - 35.06	-.LFEGSENFKR.-	1227.35	2	2.86	0.27	636.8	1	13/18	3.16E8
	96.30 - 97.55	-.QILPEANSQIVGFR.-	1572.79	2	2.74	0.39	504.6	1	16/26	1.06E10
	93.09 - 93.24	-.YQAFTQADLTNLR.-	1541.69	2	4.32	0.62	1789.0	1	20/24	2.66E9
#28	ASNA_ECO57 (P6362					60.22			6 (6 0 0 0 0)	0.61
	92.97	-.ALPDAQFEVVHSLAK.-	1625.85	2	3.72	0.63	512.3	1	19/28	2.94E9
	130.41	-.ATEAAVSEEFGLAPFLPDQIHFVHSQELLSR.-	3440.81	3	3.89	0.43	582.8	1	29/120	3.04E9
	111.22 - 111.83	-.LGLIEVQAPILSR.-	1409.70	2	4.31	0.54	1190.4	1	17/24	4.02E9
	108.63	-.LSPLHSVYVDQWDWER.-	2031.22	2	4.29	0.66	744.2	1	17/30	2.46E9
	108.55	-.LSPLHSVYVDQWDWER.-	2031.22	3	4.13	0.48	1243.6	1	28/60	1.54E9
	107.92 - 109.30	-.LSPLHSVYVDQWDWER.-	2031.22	2	3.25	0.15	550.5	3	14/30	2.75E9
#29	NADE_ECO57 (Q8XD					60.19			6 (6 0 0 0 0)	0.32
	104.40	-.EACIELSDFVR.-	1339.47	2	2.63	0.39	1019.4	1	16/20	1.93E9
	55.84	-.GAVLASEQALR.-	1115.27	2	2.90	0.35	1393.3	1	16/20	7.89E8
	55.61	-.GAVLASEQALR.-	1115.27	1	1.94	0.27	195.0	5	11/20	5.07E8
	110.30 - 110.32	-.LPYGVQADEQDCQDAIAFIQPDR.-	2650.83	2	2.73	0.47	359.9	1	14/44	9.52E8
	117.50	-.RPPITVFDDFWK.-	1521.74	2	2.87	0.22	1324.7	1	17/22	2.19E9
	78.15 - 79.11	-.YGDGGTDINPLYR.-	1441.53	2	3.88	0.67	2172.1	1	19/24	2.39E9
#30	Q8X7H3 (Q8X7H3) Hy					50.28			5 (5 0 0 0 0)	0.51
	86.80 - 87.44	-.AGLINSGGAAGGETDLSDAVR.-	1932.04	2	5.22	0.69	1496.8	1	25/40	3.69E9
	84.50	-.AGLINSGGAAGGETDLSDAVR.-	1932.04	2	5.62	0.66	1300.2	1	24/40	2.54E9
	102.55	-.CMTIPSDQLYLPGHDYVDR.-	2281.52	2	2.94	0.53	252.7	1	13/36	2.71E9
	91.44 - 92.67	-.LINAVQDVYLDSK.-	1478.67	2	4.24	0.56	1681.7	1	19/24	3.09E9
	89.01	-.LTSENPIDLVR.-	1257.42	2	2.95	0.40	814.7	1	15/20	2.03E9
#31	HFLC_ECOLI (P25661					50.26			5 (5 0 0 0 0)	0.32
	91.73	-.DALNSGSAGTEDEVTTPAADNAIAEAAER.-	2847.90	2	4.11	0.64	415.2	1	20/56	1.73E9
	127.80	-.LFADAFSKDPDFYAFIR.-	2024.26	3	5.14	0.53	1898.8	1	36/64	7.92E8
	127.90	-.LFADAFSKDPDFYAFIR.-	2024.26	2	3.95	0.57	745.8	1	19/32	1.99E9
	110.67 - 111.24	-.QINLPTEVSEAIYNR.-	1747.93	2	3.64	0.46	988.1	1	21/28	2.51E9
	109.26 - 109.77	-.YYLATGGGDISQAEVLLK.-	1899.13	2	2.77	0.52	599.0	1	15/34	1.89E9
#32	GCST_ECO57 (Q8XD					50.25			5 (5 0 0 0 0)	0.24
	58.79 - 59.42	-.ALVEGGVKPCGLGAR.-	1484.72	2	2.87	0.44	718.4	1	17/28	1.06E9
	116.11	-.FTDAQGNQHEGIITSGTFSPTLGYSIALAR.-	3154.44	3	4.90	0.45	498.6	1	27/116	2.24E9
	81.32	-.GIGETAIVQIR.-	1157.35	1	1.92	0.26	402.3	1	13/20	1.02E9
	81.18	-.GIGETAIVQIR.-	1157.35	2	3.42	0.44	2062.6	1	18/20	1.43E9
	64.26	-.YLLANDVAK.-	1007.17	1	2.03	0.19	502.6	2	11/16	8.60E8
#33	GALU_ECOLI (P25520					50.24			5 (5 0 0 0 0)	0.62
	77.84	-.ADVAPSNLAIVGR.-	1283.46	2	3.35	0.50	914.4	1	19/24	3.07E9
	83.72 - 84.27	-.GVELAPGESVPM*VGVVEKPK.-	2039.38	2	3.97	0.58	1029.7	1	23/38	3.02E9
	101.41	-.LGYM*QAFVEYGIR.-	1563.80	2	3.70	0.46	1220.4	1	17/24	2.32E9
	137.36 - 138.59	-.TPPGAGDEIQLTDAIDM*LIEK.-	2244.51	2	4.85	0.60	1042.4	1	19/40	4.73E9
	133.94 - 134.52	-.YVLSADIWPLLAK.-	1489.78	2	2.88	0.43	804.0	1	16/24	3.96E9
#34	ATPB_ECOLI (P00824					50.23			5 (5 0 0 0 0)	0.35
	92.90	-.NIAIEHSGYSVFAGVGER.-	1907.08	2	4.66	0.64	1190.0	1	20/34	1.57E9
	112.24	-.QIASLGIYPAVDPLDSTSR.-	2004.23	2	2.55	0.53	379.6	1	15/36	1.84E9
	110.75 - 112.04	-.QIASLGIYPAVDPLDSTSR.-	2004.23	2	3.85	0.65	343.9	1	14/36	2.37E9
	140.40	-.YQELKDIIAILGM*DELSEEDKLVVAR.-	3008.43	3	4.01	0.50	542.3	4	26/100	1.80E9
	111.79	-.YTLAGTEVSALLGR.-	1451.65	2	2.84	0.01	837.6	2	16/26	1.88E9
#35	SYW_ECO57 (P67589					50.23			5 (5 0 0 0 0)	0.47
	108.71	-.FNALYGDIFK.-	1188.36	2	2.97	0.23	874.9	1	15/18	1.10E9
	94.76 - 95.86	-.GEVADAVSGM*LTELQER.-	1821.99	2	4.66	0.64	1104.9	1	21/32	3.65E9
	128.26	-.GEVADAVSGMLTELQER.-	1805.99	2	4.28	0.50	1392.3	1	20/32	1.42E9
	103.22 - 104.07	-.M*TKPIVFSGAQPSGELTIGNYM*GALR.-	2772.19	3	3.10	0.43	280.9	3	25/100	3.55E9
	93.67 - 93.90	-.SDDNRNNVIGLLEDPK.-	1799.92	2	3.22	0.50	281.6	1	15/30	3.08E9
#36	GADC_ECO57 (P5822					50.19			5 (5 0 0 0 0)	0.64
	71.59	-.ANTGVTLEPINSQNAPK.-	1754.92	1	2.53	0.51	149.4	5	10/32	9.62E8
	73.01	-.ANTGVTLEPINSQNAPK.-	1754.92	1	3.27	0.60	244.5	1	13/32	1.03E9
	73.40 - 74.65	-.ANTGVTLEPINSQNAPK.-	1754.92	2	3.61	0.53	507.6	1	17/32	4.66E9
	71.53 - 72.81	-.ANTGVTLEPINSQNAPK.-	1754.92	2	3.77	0.54	434.1	1	16/32	7.91E9
	87.40 - 87.95	-.NLLPAAFAK.-	945.14	1	1.90	0.43	384.4	1	11/16	2.92E9
#37	Q8X9C9 (Q8X9C9) Re					50.18			5 (5 0 0 0 0)	0.46

	92.47	-.ALEM*IDM*HGGDLFSEE.-	1826.98	2	3.55	0.41	450.1	1	14/30	2.57E9
	77.06	-.GM*VLTGGGALLR.-	1161.40	2	2.90	0.32	1095.6	1	17/22	9.76E8
	95.27	-.GQGIVLNEPSVVAIR.-	1552.80	2	3.69	0.54	1020.5	1	17/28	3.62E9
	104.42 - 105.02	-.IGGDRFDEAIINYVR.-	1738.93	2	3.33	0.31	817.4	2	15/28	2.43E9
	84.56 - 85.08	-.VLVCVPVGATQVER.-	1527.79	2	2.82	0.48	632.2	1	19/26	3.05E9
#38	ENTC_ECOLI (P10377					40.27			4 (4 0 0 0 0)	0.39
	83.42	-.FDEPAVNGDSPDSPFQQK.-	1979.05	2	4.01	0.50	1235.9	1	22/34	2.08E9
	118.53 - 119.78	-.LFAGAGIVPASSPLGEWR.-	1829.09	2	3.54	0.37	377.5	1	16/34	2.77E9
	142.72 - 143.33	-.LIAQNPVSYNFHVPLADGGVLLGASPELLLR.-	3275.79	3	5.43	0.61	1070.2	1	32/120	3.30E9
	126.36	-.SSELHVPSSPQLITTPTLWHLATPFEGK.-	3075.46	3	3.26	0.46	527.9	1	30/108	2.48E9
#39	PHOL_ECOLI (P77349					40.24			4 (4 0 0 0 0)	0.39
	94.25 - 95.08	-.GQIQDIEPEQIHLAIK.-	1833.08	2	3.15	0.14	404.4	2	14/30	3.63E9
	95.90	-.LTGRPICVTAAADILR.-	1728.02	2	3.15	0.40	501.6	1	17/30	1.94E9
	132.66 - 133.70	-.TPNQAQYIANILDHDITFGVGPAGTGK.-	2800.08	3	4.90	0.54	2189.1	1	37/104	4.31E9
	61.29 - 61.80	-.VLEQSAESVPEYGK.-	1536.67	2	3.21	0.66	685.6	1	15/26	8.93E8
#40	AROG_ECOLI (P0088					40.19			4 (4 0 0 0 0)	0.28
	85.46	-.ELASGLSCPVGFKNGTDGTIK.-	2152.39	2	3.14	0.37	799.5	1	19/40	1.57E9
	115.53	-.SITDACIGWEDTDALLR.-	1937.09	2	2.72	0.48	454.9	1	14/32	2.24E9
	99.98	-.VAIDAINAAGAPHCFLSVTK.-	2056.35	3	3.66	0.40	811.7	1	27/76	1.43E9
	99.88	-.VAIDAINAAGAPHCFLSVTK.-	2056.35	2	3.85	0.44	613.3	1	17/38	2.32E9
#41	Q8X8D9 (Q8X8D9) Sp					40.18			4 (4 0 0 0 0)	0.33
	105.25 - 105.43	-.DGAYDLVVPSTYYVDK.-	1805.96	2	3.20	0.44	755.2	1	19/30	2.97E9
	101.50	-.LINFLLRPDVAK.-	1399.71	2	3.36	0.38	917.3	1	14/22	1.73E9
	110.61 - 111.58	-.SVTSWADLWKPEYK.-	1710.91	2	3.58	0.67	1058.8	1	17/26	2.86E9
	69.23 - 69.82	-.VIYSTYESNETM*YAK.-	1815.98	2	3.62	0.63	821.6	1	16/28	1.46E9
#42	RECA_ECOLI (P03017					40.17			4 (4 0 0 0 0)	0.17
	62.23	-.AEIEGEIGDSHM*GLAAR.-	1772.92	2	3.31	0.45	501.3	1	14/32	9.47E8
	79.97	-.ALAAALGQIEK.-	1085.28	1	1.99	0.36	670.0	1	13/20	7.41E8
	84.54	-.IGVM*FGNPETTTGGNALK.-	1824.05	2	2.68	0.40	385.8	1	15/34	1.66E9
	64.30 - 64.84	-.IVEIYGPESSGK.-	1279.42	2	2.50	0.21	778.1	1	15/22	1.17E9
#43	SYGA_ECO57 (P6702					30.29			3 (3 0 0 0 0)	0.31
	71.80 - 73.19	-.ALGPEPM*AAAYVQPSR.-	1674.90	2	3.48	0.59	910.3	1	18/30	4.35E9
	121.08	-.EAQQLLALENPLPLPAYER.-	2166.46	2	5.85	0.66	1375.5	1	20/36	3.24E9
	153.05	-.TFQGLILTLQDYWAR.-	1826.09	2	2.75	0.47	839.8	1	17/28	8.95E8
#44	Q8XEF9 (Q8XEF9) Alc					30.26			3 (3 0 0 0 0)	0.18
	102.88	-.TGVILGHEGIGVVAEVGPGVTSLKPGDR.-	2716.09	3	3.85	0.42	1455.3	1	36/108	1.68E9
	86.40 - 86.74	-.VIAIDVNDEQLK.-	1357.54	2	2.61	0.46	436.2	6	11/22	2.04E9
	92.37	-.VPDGLDSAAASSITCAGVTTYK.-	2185.37	2	5.15	0.74	1158.2	1	21/42	1.23E9
#45	SYFA_ECO57 (P67037					30.25			3 (3 0 0 0 0)	0.20
	82.47	-.AAISQASDVAALDNVR.-	1601.74	2	5.08	0.63	2057.0	1	21/30	2.33E9
	69.12	-.ELPPEERPAAGAVINEAK.-	1892.10	2	3.26	0.46	208.0	5	14/34	1.04E9
	89.11	-.LAAETIDVSLPGR.-	1342.52	2	3.94	0.60	1559.1	1	18/24	2.05E9
#46	AROB_ECO57 (Q8X82					30.25			3 (3 0 0 0 0)	0.22
	122.11	-.FIQVPTTLLSQVDSSVGGK.-	1977.25	2	5.05	0.60	689.0	1	22/36	2.13E9
	127.94	-.NM*IGAFYQPASVVVDLDCLK.-	2257.59	2	4.07	0.56	464.8	1	17/38	1.63E9
	146.55 - 147.08	-.SYPITIASGLFNEPASFLPLK.-	2266.62	2	4.26	0.53	620.4	1	16/40	2.15E9
#47	TKRA_ECO57 (P5822					30.23			3 (3 0 0 0 0)	0.30
	110.86	-.ATSTISVGYDNFDVDALTAR.-	2117.26	2	3.72	0.52	395.8	1	15/38	2.29E9
	111.64 - 112.01	-.GPVVDENALIAALQK.-	1538.77	2	4.68	0.70	1836.8	1	21/28	3.43E9
	132.88	-.YGM*AACAVDNLIDALQGK.-	1927.16	2	4.11	0.62	1266.2	1	20/34	2.57E9
#48	GNE_ECO57 (Q8X7P7					30.23			3 (3 0 0 0 0)	0.14
	155.37	-.DQQALDQALAGFDTVVLLAAEHR.-	2482.73	3	4.51	0.45	1328.4	1	33/88	7.28E8
	109.32	-.LLETAIADFNIK.-	1348.57	2	3.51	0.55	1635.2	1	18/22	1.32E9
	106.75	-.SLTIIRPTVIFGER.-	1602.90	2	2.94	0.43	897.8	1	17/26	1.79E9
#49	GLYA_ECO57 (Q8XA5					30.22			3 (3 0 0 0 0)	0.27
	115.92	-.LYNIVPYGIDATGHIDYADLEK.-	2481.74	3	3.21	0.34	585.1	1	27/84	3.06E9
	76.42	-.VRQEEHIELIASENYTSPR.-	2272.46	2	4.44	0.53	979.1	1	18/36	7.95E8
	132.82 - 134.23	-.YYGGCEYVDIVEQLAIDR.-	2164.35	2	3.74	0.47	709.9	1	16/34	3.61E9
#50	CH60_ECOLI (P06139					30.22			3 (3 0 0 0 0)	0.18
	44.49 - 46.52	-.ATLEDLGQAK.-	1046.16	1	1.88	0.34	283.5	1	11/18	2.70E8
	95.67	-.DTTTIIDGVGEEAAIQGR.-	1846.97	2	4.43	0.47	1071.2	1	19/34	2.91E9
	89.13	-.QIVLNCGEEPSVVANTVK.-	1958.20	2	3.76	0.58	1395.9	1	22/34	1.88E9
#51	Q8XE84 (Q8XE84) Put					30.21			3 (3 0 0 0 0)	0.16
	64.37 - 65.17	-.LTGVSEELGATR.-	1233.35	2	3.30	0.50	911.6	1	18/22	1.40E9
	161.73	-.WDYNTPIEETLEALNDVVK.-	2250.45	2	4.12	0.58	435.9	1	16/36	1.11E9
	91.28	-.YIGASSM*HASQFAQALELQK.-	2197.46	2	4.01	0.57	510.8	1	16/38	1.85E9
#52	PROB_ECOLI (P07005					30.20			3 (3 0 0 0 0)	0.18
	44.32 - 45.27	-.AIAGDSVSGLGTGGM*STK.-	1625.78	2	2.77	0.46	984.0	1	17/34	2.54E8
	133.53 - 134.13	-.VGDNDNLSALAAILAGADK.-	1829.00	2	3.62	0.53	734.0	1	15/36	2.50E9
	131.65 - 132.17	-.VGDNDNLSALAAILAGADK.-	1829.00	2	4.09	0.64	863.0	1	18/36	2.13E9
#53	DCEB_ECOLI (P28302					30.20			3 (3 0 0 0 0)	0.19
	90.17	-.LKDGEDPGYTLYDLSER.-	1972.10	2	3.96	0.54	449.1	1	19/32	1.44E9
	91.77	-.LKDGEDPGYTLYDLSER.-	1972.10	2	3.32	0.46	528.6	1	20/32	1.55E9
	90.06	-.LKDGEDPGYTLYDLSER.-	1972.10	3	3.04	0.18	1165.9	1	26/64	2.35E9

#54	Q8X9Q7 (Q8X9Q7) Fe					30.19			3 (3 0 0 0 0)		0.22
	122.66 - 123.26	-.DANFSLEGLTGFTM*YGK.-	1868.06	2	2.94	0.54	763.1	1	16/32		2.30E9
	83.97	-.SNDVIQDDVFR.-	1308.38	2	2.97	0.42	1013.6	1	16/20		1.50E9
	99.16	-.VPAYDPEAVAEHAIGM*M*M*TLNR.-	2465.81	3	3.78	0.53	998.9	1	30/84		2.10E9
#55	SELD_ECO57 (P6679					30.17			3 (3 0 0 0 0)		0.17
	90.60 - 91.26	-.FVDPNLLVGNETR.-	1474.64	2	2.96	0.41	1104.6	1	15/24		2.30E9
	111.97	-.LFLTKPLGIGVLTTAEK.-	1802.19	2	2.79	0.34	260.7	1	15/32		1.48E9
	65.19	-.VLETILHSEQAK.-	1368.56	2	3.43	0.51	767.2	1	15/22		8.51E8
#56	Q7DBF6 (Q7DBF6) Fu					30.14			3 (3 0 0 0 0)		0.12
	65.33 - 66.63	-.IDQVYLAAAK.-	1092.27	2	2.76	0.41	1544.0	1	16/18	1	9.68E8
	65.25	-.IDQVYLAAAK.-	1092.27	1	1.80	0.08	435.4	29	10/18	1	9.21E8
	110.13	-.LLFLGSSCIYPK.-	1398.67	2	2.84	0.46	1030.0	1	17/22	1	1.50E9
#57	Q8X8S6 (Q8X8S6) Re					20.30			2 (2 0 0 0 0)		0.14
	126.05	-.FSSAFSALAETLDNQEEPEKLTIEPSVK.-	3082.36	3	5.97	0.57	1389.1	1	36/108		1.96E9
	104.02	-.LAQYIQQVDDKVNQELEK.-	2162.39	2	3.96	0.33	828.6	1	16/34		1.87E9
#58	MDH_ECO57 (P61891					20.25			2 (2 0 0 0 0)		0.14
	153.70	-.SIGTLSAFEQNALEGM*LDTLK.-	2255.53	2	2.51	0.08	475.0	2	13/40		1.41E9
	132.90	-.TQLPSGSELSLYDIAPVTPGVAVDLSHIPTAVK	3377.83	3	4.99	0.48	882.3	1	34/128		2.36E9
#59	YBHE_ECO57 (Q8X92					20.24			2 (2 0 0 0 0)		0.15
	110.47 - 111.71	-.ICLFTVSDDGHLVAQDPAEVTTVEGAGPR.-	3056.32	3	4.80	0.56	641.5	5	27/112		2.62E9
	85.10	-.YLYVGVRPEFR.-	1399.62	2	2.64	0.45	395.2	1	12/20		1.58E9
#60	YCCZ_ECOLI (P75881					20.23			2 (2 0 0 0 0)		0.11
	90.72	-.AKPIM*SQANPELEQQIANYEYR.-	2610.88	2	4.12	0.46	756.2	1	20/42		1.26E9
	90.66	-.AKPIM*SQANPELEQQIANYEYR.-	2610.88	3	4.70	0.60	2000.3	1	32/84		1.77E9
#61	Q8XD41 (Q8XD41) Hy					20.21			2 (2 0 0 0 0)		0.14
	111.54 - 112.99	-.AALANIFSELPSK.-	1361.57	2	4.23	0.54	1564.2	1	19/24		1.98E9
	114.28 - 114.92	-.VRDDANTLCIEPLPYSLEE.-	2235.43	2	4.22	0.58	563.6	1	16/36		1.91E9
#62	TDH_ECO57 (Q8XEJ1					20.21			2 (2 0 0 0 0)		0.15
	109.62	-.M*AALIQSGLDLSPIITHR.-	1953.30	2	4.18	0.62	745.6	1	17/34		1.99E9
	77.71 - 78.65	-.NVVITDVNEYR.-	1322.45	2	2.77	0.47	1049.2	1	15/20		2.25E9
#63	HIS8_ECO57 (Q9S5G					20.20			2 (2 0 0 0 0)		0.14
	136.11 - 136.56	-.EIPCVEQVFDSETNYILAR.-	2284.50	2	4.10	0.52	762.0	1	19/36		2.83E9
	109.47	-.SLWDQGIILR.-	1201.40	2	3.31	0.04	1470.7	1	16/18		1.09E9
#64	Q8XCF0 (Q8XCF0) Fr					20.20			2 (2 0 0 0 0)		0.23
	106.61	-.AGLVDILGASGAENVQGEVQQK.-	2184.39	2	4.06	0.59	603.5	1	17/42		2.02E9
	94.57 - 95.76	-.VTPVGTPVTEEDFLQPGNK.-	2029.24	2	3.57	0.56	770.1	1	20/36		4.30E9
#65	Q7DBF5 (Q7DBF5) GD					20.20			2 (2 0 0 0 0)		0.19
	102.09 - 102.43	-.GIVDSVEGQDAPGVKPGDVIVAVDPR.-	2590.87	3	4.00	0.57	1441.1	1	31/100		3.48E9
	97.40	-.YFRPAEVDTLLGDPSK.-	1809.01	2	4.02	0.38	710.6	1	20/30		1.79E9
#66	ATPA_ECOLI (P00822					20.20			2 (2 0 0 0 0)		0.12
	104.63	-.DRGEDALIIYDDLSK.-	1723.86	2	3.92	0.48	2530.6	1	21/28		1.76E9
	83.63	-.VNAEYVEAFTK.-	1271.40	2	3.66	0.46	1698.1	1	18/20		1.46E9
#67	RS1_ECOLI (P02349)					20.19			2 (2 0 0 0 0)		0.12
	94.66	-.AVIESENSAERDQLLENLQEGM*EVK.-	2849.08	3	3.37	0.60	718.8	1	27/96		2.21E9
	76.85	-.VKHPSEIVNVGDEITVK.-	1865.12	2	3.83	0.56	949.8	1	21/32		1.17E9
#68	LIVJ_ECOLI (P02917)					20.19			2 (2 0 0 0 0)		0.10
	76.69	-.NYDQVPANKPIVDAIK.-	1786.02	2	2.84	0.48	462.9	1	17/30		9.19E8
	120.60	-.TQFM*GPEGVANVSLSNIAGESAEGLLVTKPK	3162.56	3	3.76	0.54	499.8	1	27/120		1.77E9
#69	DCUP_ECO57 (Q8X6					20.18			2 (2 0 0 0 0)		0.13
	129.99 - 130.54	-.ELKGEVPLIGFSGSPWTLATYM*VEGGSSK.-	3058.45	3	3.60	0.47	607.1	7	26/112		2.41E9
	78.00	-.VALQGNM*DPSM*LYAPPAR.-	1964.26	2	3.15	0.43	765.1	1	18/34		1.21E9
#70	Q8X677 (Q8X677) Mar					20.18			2 (2 0 0 0 0)		0.12
	87.17	-.DVIESDKSTLLGEAVAK.-	1775.98	2	3.59	0.49	726.4	1	16/32		1.49E9
	86.92	-.SALDSQQGEPWQTIR.-	1716.83	2	2.60	0.36	421.4	1	14/28		1.69E9
#71	Q8XBD3 (Q8XBD3) Li					20.18			2 (2 0 0 0 0)		0.09
	101.02	-.ASTTM*DVQSAADDTGLPM*LVVR.-	2311.58	2	2.55	0.58	523.1	1	17/42		1.18E9
	77.57	-.YQISVKPQGYQQAVTVK.-	1938.22	2	3.57	0.54	257.5	1	16/32		1.33E9
#72	TRXB_ECOLI (P09625					20.18			2 (2 0 0 0 0)		0.14
	112.45 - 113.60	-.LLILGSGPAGYTAAVYAAR.-	1865.17	2	3.38	0.59	909.6	1	18/36		2.53E9
	68.42 - 68.44	-.TLEEVTGDQM*GVTGVR.-	1708.87	2	3.57	0.44	826.8	1	17/30		1.38E9
#73	Q8XBT7 (Q8XBT7) Pu					20.16			2 (2 0 0 0 0)		0.15
	123.51	-.FIAAAANYPENIDPWHILQTGGK.-	2527.82	3	3.02	0.41	734.4	1	24/88		1.52E9
	140.83 - 141.45	-.NFFEQLGVPTHLSDYGLDGSSIPALLK.-	2920.27	3	3.19	0.39	907.5	1	31/104		2.57E9
#74	Q8XAS0 (Q8XAS0) Pu					20.14			2 (2 0 0 0 0)		0.09
	65.75	-.PSLAYLKSK.-	1007.21	1	2.34	0.34	548.9	1	12/16		1.16E9
	65.68	-.PSLAYLKSK.-	1007.21	2	2.75	0.57	1231.5	1	14/16		1.22E9
#75	Q8XCY2 (Q8XCY2) Tr					10.23			1 (1 0 0 0 0)		0.07
	125.12	-.TPGHPEVGYTAGVETTTGPLGQGIANAVGM*	3412.77	3	4.61	0.52	767.6	1	31/136		1.99E9
#76	Q8X9W9 (Q8X9W9) P					10.23			1 (1 0 0 0 0)		0.08
	110.09 - 110.63	-.EGETLVVAAATGPVGATVGQIGK.-	2126.40	2	4.50	0.58	634.8	1	19/44		2.29E9
#77	DEGP_ECOLI (P0937					10.22			1 (1 0 0 0 0)		0.04
	161.75	-.VGDYTVAIGNPFGLGETVTSGIVSALGR.-	2752.07	3	4.32	0.49	795.2	1	27/108		1.05E9
#78	DNAK_ECOLI (P04475					10.21			1 (1 0 0 0 0)		0.07
	95.97	-.TFEVLATNGDTHLGGEDFDSR.-	2282.37	2	4.12	0.63	1537.4	1	25/40		1.89E9

#79	PURR_ECOLI (P1503					10.20			1 (1 0 0 0 0)		0.10
	106.77 - 107.31	-.VPESWIVQGDFEPESGYR.-	2096.24	2	4.08	0.44	1543.9	1	20/34		2.72E9
#80	Q8X623 (Q8X623) Cyc					10.20			1 (1 0 0 0 0)		0.09
	109.58	-.M*LDPFM*QYSCAYWK.-	1873.14	2	4.07	0.44	1107.6	1	20/26		2.45E9
#81	Q8XBW7 (Q8XBW7) C					10.20			1 (1 0 0 0 0)		0.06
	96.20	-.APSLYQTNPNYILYSK.-	1873.10	2	4.04	0.66	1149.0	1	19/30		1.62E9
#82	PYRD_ECOLI (P05021					10.20			1 (1 0 0 0 0)		0.06
	146.57	-.IAPDLSEEELIQVADSLVR.-	2098.34	2	4.03	0.62	1661.3	1	22/36		1.74E9
#83	Q8XDF3 (Q8XDF3) As					10.20			1 (1 0 0 0 0)		0.05
	157.71 - 158.34	-.M*FENITAAPADPILGLADLFR.-	2292.64	2	3.98	0.55	529.7	1	18/40		1.37E9
#84	G6PI_ECOLI (P11537)					10.20			1 (1 0 0 0 0)		0.10
	140.43	-.LLSNFFAQTEALAFGK.-	1758.01	2	3.93	0.62	637.9	1	16/30		2.77E9
#85	Q8X9B0 (Q8X9B0) Citr					10.20			1 (1 0 0 0 0)		0.04
	159.15	-.TAGSSGANPFACIAAGIASLWGPAHGGANEA	3197.50	3	3.91	0.49	499.8	1	32/132		1.10E9
#86	Q8XBL3 (Q8XBL3) PE					10.19			1 (1 0 0 0 0)		0.08
	111.88	-.VLAEQALAQPTTDELM*TLVNK.-	2302.63	2	3.89	0.52	271.5	1	14/40		2.09E9
#87	Q8X633 (Q8X633) Out					10.19			1 (1 0 0 0 0)		0.06
	156.68 - 157.54	-.YTLPLTAINQFLTVGGEWR.-	2180.49	2	3.80	0.65	669.9	1	16/36		1.54E9
#88	Q8XE22 (Q8XE22) 6-p					10.19			1 (1 0 0 0 0)		0.08
	79.02 - 79.67	-.LTQLISAAQNQGIR.-	1513.73	2	3.79	0.59	1678.3	1	20/26		2.18E9
#89	Q8X942 (Q8X942) UD					10.19			1 (1 0 0 0 0)		0.10
	94.72 - 95.74	-.IPYVESFPTGTPQSPYGK.-	1969.18	2	3.78	0.45	770.9	1	22/34		2.61E9
#90	AROC_ECO57 (P6361					10.19			1 (1 0 0 0 0)		0.08
	130.01	-.DWSQVEQNPFFCPDPDKIDALDELM*R.-	3183.44	3	3.78	0.53	797.4	1	28/100		2.13E9
#91	OMPA_ECOLI (P0293					10.19			1 (1 0 0 0 0)		0.04
	78.11	-.FGQGEAAPVVAPAPAPAPEVQTK.-	2233.51	2	3.78	0.60	332.2	1	15/44		1.09E9
#92	DLDH_ECOLI (P00391					10.19			1 (1 0 0 0 0)		0.08
	127.48 - 127.65	-.VIPSIAYTEPEVAWVGLTEK.-	2203.52	2	3.77	0.60	1068.9	1	23/38		2.12E9
#93	GM4D_ECOLI (P3205					10.19			1 (1 0 0 0 0)		0.13
	118.72	-.FYQASTSELYGLVQEIPQK.-	2202.45	2	3.72	0.49	454.4	1	16/36	1	3.46E9
#94	GCSP_ECO57 (Q8XD					10.18			1 (1 0 0 0 0)		0.08
	111.60	-.LIDYGFHAPTM*SFPVAGTLM*VEPTESESK.-	3188.57	3	3.68	0.28	1378.8	1	32/112		2.27E9
#95	Q8XAR1 (Q8XAR1) Pu					10.18			1 (1 0 0 0 0)		0.10
	135.37 - 135.99	-.LALWQNYYDALAPLAK.-	1851.14	2	3.61	0.58	897.5	1	16/30		2.62E9
#96	GSHB_ECO57 (P5857					10.18			1 (1 0 0 0 0)		0.06
	107.20	-.LGIVM*DPIANINIK.-	1527.86	2	3.59	0.61	692.5	1	16/26		1.62E9
#97	G3P3_ECO57 (P58072					10.18			1 (1 0 0 0 0)		0.08
	136.86 - 138.08	-.TVAWYDNEYGFVTQLIR.-	2076.30	2	3.55	0.56	747.3	1	17/32		2.29E9
#98	YBIC_ECO57 (P58409					10.17			1 (1 0 0 0 0)		0.11
	95.19 - 96.46	-.AFGQVAAHEAM*ALGIEK.-	1760.01	2	3.45	0.43	600.1	1	16/32		3.00E9
#99	SERB_ECOLI (P06862					10.17			1 (1 0 0 0 0)		0.07
	112.75	-.FTGNVIGDIVDAQYK.-	1640.82	2	3.40	0.67	609.9	1	17/28		1.94E9
#100	YFIF_ECOLI (P33635)					10.17			1 (1 0 0 0 0)		0.08
	94.96	-.VYGENACQALFQSRPEAIVR.-	2309.56	3	3.35	0.54	920.4	1	28/76		2.08E9
#101	ADD_ECO57 (Q8X661					10.17			1 (1 0 0 0 0)		0.14
	113.07 - 114.10	-.ASINTDDPGVQGVDIIHEYTVAAPAAGLSR.-	3039.30	3	3.34	0.43	597.1	2	28/116		3.77E9
#102	METN_ECO57 (P6335					10.17			1 (1 0 0 0 0)		0.13
	103.47 - 104.78	-.LEFTGQSVDAPLLSETAR.-	1935.13	2	3.34	0.48	825.9	1	16/34		3.61E9
#103	DACA_ECOLI (P04287					10.17			1 (1 0 0 0 0)		0.07
	94.55	-.ASLGVDKDVYLTIPR.-	1647.90	2	3.32	0.43	1640.6	1	21/28		1.90E9
#104	Q8X5Q2 (Q8X5Q2) Oli					10.16			1 (1 0 0 0 0)		0.07
	127.71 - 128.20	-.AEFGVDELQPWDIAYYSEK.-	2261.43	2	3.22	0.59	283.3	1	12/36		1.83E9
#105	NDPA_ECO57 (Q8XE6					10.16			1 (1 0 0 0 0)		0.10
	120.62	-.AYGLFSEESELAQTLR.-	1814.97	2	3.22	0.51	1008.1	1	17/30		2.83E9
#106	Q8XCG2 (Q8XCG2) H					10.16			1 (1 0 0 0 0)		0.18
	93.30 - 93.61	-.VEQISQPDVNINLVTLNAKGSEK.-	2497.79	3	3.20	0.38	342.0	12	24/88		4.84E9
#107	Q8XDE6 (Q8XDE6) An					10.16			1 (1 0 0 0 0)		0.08
	96.11 - 96.84	-.HQQGQPLSLPVHVADAFR.-	2001.24	2	3.14	0.49	943.0	1	19/34		2.18E9
#108	Q8X6U3 (Q8X6U3) D-					10.16			1 (1 0 0 0 0)		0.04
	91.56	-.GSSVM*FLKPGDQVSVADLNK.-	2109.39	2	3.10	0.40	377.5	1	15/38		1.19E9
#109	YFEU_ECO57 (Q8XBJ					10.16			1 (1 0 0 0 0)		0.31
	96.94 - 97.47	-.M*QLEKMITEGSNAASAEIDR.-	2211.46	3	3.10	0.01	1662.9	1	30/76		8.46E9
#110	MLTA_ECOLI (P46885					10.15			1 (1 0 0 0 0)		0.06
	129.38	-.LYGNQSNVYNAVQEWLR.-	2055.24	2	3.04	0.28	220.9	5	10/32		1.52E9
#111	RvrsDB|00000099					10.15			1 (1 0 0 0 0)		0.06
	150.15 - 150.75	-.AWNDLDM*ANGRIYC#MGNILSSGGLGK.-	2888.19	3	3.02	0.09	938.6	1	31/100		1.52E9
#112	ISPG_ECO57 (P62622					10.15			1 (1 0 0 0 0)		0.05
	69.29 - 69.86	-.TTDVEATVNQIK.-	1319.44	2	3.01	0.47	1166.3	1	16/22		1.43E9
#113	Q9LAP1 (Q9LAP1) Iha					10.15			1 (1 0 0 0 0)		0.10
	79.38 - 80.32	-.IPYPTESQNYNLGAR.-	1723.87	2	3.00	0.38	777.5	1	17/28		2.68E9
#114	Q8X5M4 (Q8X5M4) Ke					10.15			1 (1 0 0 0 0)		0.07
	100.13	-.LPGLYYIETDSTGER.-	1714.85	2	2.93	0.47	790.5	1	16/28		1.94E9
#115	ILVE_ECOLI (P00510)					10.14			1 (1 0 0 0 0)		0.01
	18.70	-.SVDGIQVGEGR.-	1117.20	2	2.84	0.38	1435.9	1	16/20		1.41E8

#116	HSLO_ECOLI (P45803					10.14			1 (1 0 0 0 0)	0.05
	53.86 - 55.23	-.VQGEIPENADLK.-	1313.44	2	2.82	0.12	659.0	1	17/22	1.50E9
#117	RvrsDB|00004199					10.14			1 (1 0 0 0 0)	0.26
	97.41 - 98.63	-.TSQEDTAEQRSIVESFLQR.-	2225.36	2	2.76	0.10	619.0	2	15/36	7.26E9
#118	YQJD_ECO57 (P6458:					10.14			1 (1 0 0 0 0)	0.21
	111.41 - 112.48	-.EELSKIRSKAEQALK.-	1730.99	2	2.72	0.17	929.4	1	16/28	5.81E9
#119	CLPB_ECO57 (P6328					10.14			1 (1 0 0 0 0)	0.06
	74.15	-.GELHCVGATTLDEYR.-	1721.84	2	2.72	0.47	1244.1	1	17/28	1.58E9
#120	Q8XAJ4 (Q8XAJ4) Hyp					10.13			1 (1 0 0 0 0)	0.11
	60.21 - 61.42	-.C#VYDPIKGNFIIKDS.-	1827.03	2	2.63	0.34	533.8	1	15/28	2.95E9
#121	Q8XCY5 (Q8XCY5) Hy					10.13			1 (1 0 0 0 0)	0.45
	97.00 - 97.59	-.CKAM*GVDVNRVYSLDVLVR.-	2211.57	2	2.62	0.05	191.1	36	9/36	1.23E10
#122	Q8X497 (Q8X497) Hyp					10.13			1 (1 0 0 0 0)	0.05
	81.22	-.LSNDYSDFIAK.-	1273.37	2	2.56	0.45	1356.8	1	17/20	1.25E9
#123	RPOB_ECOLI (P00575					10.13			1 (1 0 0 0 0)	0.05
	68.71	-.LGDLPTSGQIR.-	1157.30	2	2.55	0.43	601.3	1	15/20	1.38E9
#124	Q8X6G7 (Q8X6G7) Z4					10.13			1 (1 0 0 0 0)	0.06
	124.21	-.ISDGLKEIVSLK.-	1302.54	2	2.54	0.16	692.6	3	14/22	1.60E9
#125	RvrsDB|00003534					10.11			1 (1 0 0 0 0)	0.03
	65.46	-.M*LLASSAINR.-	1092.30	1	2.26	0.11	679.6	6	12/18	7.93E8
#126	Q8X4B0 (Q8X4B0) Z07					10.11			1 (1 0 0 0 0)	0.05
	78.53	-.DRHPDGSLLK.-	1138.26	1	2.20	0.29	184.5	15	10/18	1.28E9
#127	RvrsDB|00005266					10.11			1 (1 0 0 0 0)	0.03
	45.95 - 49.37	-.RALDGK.-	659.76	1	1.84	0.09	270.1	2	7/10	8.53E8
#128	RvrsDB|00004509					10.11			1 (1 0 0 0 0)	0.10
	82.35	-.RRAPKAMM*.-	977.23	1	1.81	0.00	287.0	3	9/14	2.70E9
#129	PYRG_ECOLI (P08398					10.10			1 (1 0 0 0 0)	0.07
	67.30 - 67.84	-.YEVNNM*LLK.-	1140.34	1	1.99	0.12	464.3	1	12/16	2.03E9
#130	RvrsDB|00003017					10.09			1 (1 0 0 0 0)	0.06
	88.49	-.QAAREFFEVNDK.-	1454.57	1	1.85	0.09	385.9	1	12/22	1.70E9
