# Supplementary material for: Proteins other than the locus of enterocyte effacement-encoded proteins contribute to Escherichia coli O157:H7 adherence to bovine rectoanal junction stratified squamous epithelial cells
Source: BMC Microbiol. 2012 Jun 12;12:103. doi: 10.1186/1471-2180-12-103 (PMC3420319; doi:10.1186/1471-2180-12-103)
Supplement: Additional file 9 — http://www.biomedcentral.com/imedia/1233524502675419/supp9.pdf. DATA SHEETS: O157-DMEM MS/MS data sheet 5. [file 1471-2180-12-103-S9.pdf]

DMEM-05 SequestReport

	Reference					Score	Accession		Peptides (Hits)		Area
	Time(s)	Sequence	MH+	Charge	XC	Delta Cn	Sp	RSp	Ions	Count	Peak Area
#1	OMPA_ECOLI (P0293					460.34			46 (46 0 0 0 0)		18.52
	82.77 - 83.89	-.AALIDCLAPDR.-	1215.38	2	3.47	0.31	1206.1	1	17/20		1.29E10
	82.99 - 83.53	-.AALIDCLAPDR.-	1215.38	1	2.33	0.41	498.7	1	15/20		6.07E9
	82.84 - 84.02	-.AALIDCLAPDR.-	1215.38	1	1.83	0.11	401.7	1	13/20		1.02E10
	74.06 - 75.02	-.AALIDCLAPDRR.-	1371.56	2	2.70	0.16	757.9	1	14/22		4.68E9
	97.92 - 98.58	-.AQSVVDYLISK.-	1223.40	2	3.42	0.48	971.0	1	17/20		8.15E9
	126.88 - 127.98	-.ATLKPEGQAALDQLYSQLSNLDPK.-	2601.89	2	4.46	0.62	412.7	1	18/46		2.01E10
	126.60 - 127.74	-.ATLKPEGQAALDQLYSQLSNLDPK.-	2601.89	3	4.22	0.43	541.1	1	29/92		7.83E10
	84.38 - 84.92	-.DGSVVVLGYTDR.-	1281.40	1	2.25	0.45	272.7	1	14/22		3.05E9
	84.51 - 85.24	-.DGSVVVLGYTDR.-	1281.40	2	2.91	0.37	1624.3	1	18/22		3.31E9
	80.67 - 82.05	-.DGSVVVLGYTDR.-	1281.40	2	3.64	0.55	1239.8	1	17/22		8.79E9
	78.93 - 81.54	-.DGSVVVLGYTDR.-	1281.40	1	2.37	0.51	307.7	1	15/22		1.15E10
	78.66 - 80.10	-.DGSVVVLGYTDR.-	1281.40	2	3.36	0.42	1273.2	1	18/22		9.06E9
	79.07 - 80.60	-.DGSVVVLGYTDR.-	1281.40	1	3.00	0.42	415.4	1	16/22		7.09E9
	29.89	-.DNTWYTGAK.-	1056.11	1	2.20	0.38	343.5	1	10/16		9.90E8
	76.56 - 77.67	-.FGQGEAAPVVAPAPAPAPEVQTK.-	2233.51	2	5.08	0.63	918.3	1	23/44		8.23E10
	76.64 - 77.21	-.FGQGEAAPVVAPAPAPAPEVQTK.-	2233.51	2	5.34	0.72	756.6	1	21/44		9.88E10
	78.99 - 79.54	-.FGQGEAAPVVAPAPAPAPEVQTK.-	2233.51	2	5.54	0.70	759.3	1	21/44		5.82E9
	12.69 - 15.90	-.GIKDVVTQPQA.-	1156.31	1	1.92	0.25	218.5	3	11/20		3.12E9
	12.59 - 13.83	-.GIKDVVTQPQA.-	1156.31	2	3.17	0.49	892.6	2	15/20		4.35E9
	18.55 - 19.22	-.GIKDVVTQPQA.-	1156.31	2	2.52	0.32	677.3	2	14/20		2.95E8
	44.68 - 45.66	-.GM*GESNPVTGNTCDNVK.-	1796.89	2	3.35	0.62	654.5	1	17/32		4.66E8
	42.64 - 43.86	-.GM*GESNPVTGNTCDNVK.-	1796.89	2	2.99	0.40	486.9	1	16/32		4.96E8
	44.54 - 45.30	-.IGSDAYNQGLSER.-	1410.47	2	3.52	0.49	1348.7	1	17/24		4.87E8
	42.54 - 43.94	-.IGSDAYNQGLSER.-	1410.47	2	3.08	0.48	806.8	1	15/24		4.53E8
	39.24 - 40.05	-.IGSDAYNQGLSER.-	1410.47	2	3.61	0.60	1365.2	1	19/24		3.47E8
	11.71	-.IGSDAYNQGLSER.-	1410.47	1	2.19	0.42	301.5	1	14/24		2.89E9
	11.56	-.IGSDAYNQGLSER.-	1410.47	1	1.92	0.38	127.1	14	9/24		3.22E9
	35.80	-.IGSDAYNQGLSER.-	1410.47	2	2.73	0.44	781.3	1	15/24		2.34E8
	40.88 - 42.08	-.IGSDAYNQGLSER.-	1410.47	2	3.97	0.56	1147.8	1	18/24		3.39E8
	109.91	-.LGYPITDDLDIYTR.-	1655.83	1	3.20	0.57	503.5	1	14/26		8.19E9
	104.29 - 104.86	-.LGYPITDDLDIYTR.-	1655.83	2	3.58	0.58	1157.5	1	18/26		6.15E9
	109.68 - 110.93	-.LGYPITDDLDIYTR.-	1655.83	2	3.40	0.63	1058.3	1	18/26		7.29E10
	109.86 - 110.38	-.LGYPITDDLDIYTR.-	1655.83	1	2.64	0.48	599.4	1	15/26		9.26E9
	113.73 - 115.06	-.NHDTGVSPVFAGGVEYAITPEIATR.-	2602.84	2	2.59	0.56	592.8	1	18/48		4.85E9
	108.09 - 109.47	-.NHDTGVSPVFAGGVEYAITPEIATR.-	2602.84	2	6.89	0.73	2054.3	1	27/48		1.44E10
	112.19	-.NHDTGVSPVFAGGVEYAITPEIATR.-	2602.84	2	5.03	0.59	1755.1	1	24/48		5.42E9
	88.97 - 89.59	-.RAQSVVDYLISK.-	1379.59	3	4.07	0.51	1261.5	1	27/44		2.34E9
	88.82 - 89.96	-.RAQSVVDYLISK.-	1379.59	2	3.74	0.36	1152.0	1	18/22		1.84E10
	89.01 - 89.66	-.RAQSVVDYLISK.-	1379.59	1	2.53	0.54	257.8	2	12/22		3.52E9
	89.45 - 90.67	-.SDVLFNFNK.-	1084.21	2	3.41	0.51	936.1	1	14/16		7.47E9
	94.70	-.SDVLFNFNK.-	1084.21	1	2.15	0.44	394.1	2	9/16		2.27E9
	91.25	-.SDVLFNFNK.-	1084.21	2	3.01	0.49	995.8	1	13/16		7.92E9
	89.16 - 92.10	-.SDVLFNFNK.-	1084.21	1	2.37	0.42	491.7	1	12/16		2.10E10
	106.62 - 108.03	-.SNVYGKNHDTGVSPVFAGGVEYAITPEIATR.	3251.55	3	6.23	0.63	2337.9	1	40/120		8.58E9
	106.78	-.SNVYGKNHDTGVSPVFAGGVEYAITPEIATR.	3251.55	2	4.21	0.72	816.4	1	22/60		2.95E9
	108.60 - 109.28	-.SNVYGKNHDTGVSPVFAGGVEYAITPEIATR.	3251.55	3	3.49	0.52	1145.9	1	33/120		4.82E9
#2	EFTS_ECOLI (P02997					340.27			34 (34 0 0 0 0)		3.74
	92.97 - 93.53	-.ALTEANGDIELAIENM*R.-	1877.07	2	5.06	0.55	1724.5	1	20/32		5.66E9
	85.09 - 86.25	-.ALTEANGDIELAIENM*RK.-	2005.24	2	3.51	0.53	558.7	1	16/34		4.13E9
	70.07 - 71.01	-.DAGFQAFADK.-	1070.14	2	3.19	0.50	1076.4	1	15/18		2.63E9
	70.53 - 71.10	-.DAGFQAFADK.-	1070.14	1	2.45	0.27	765.4	1	13/18		8.90E9
	70.68	-.DAGFQAFADK.-	1070.14	1	3.31	0.24	720.9	1	13/18		4.41E9
	57.70	-.EHNAEVTGFIR.-	1273.38	2	2.64	0.49	843.4	1	15/20		2.86E9
	56.21 - 57.36	-.EHNAEVTGFIR.-	1273.38	1	2.12	0.42	329.5	1	16/20		2.66E9
	55.94 - 57.11	-.EHNAEVTGFIR.-	1273.38	2	2.70	0.45	1126.1	1	16/20		2.82E9
	56.89	-.EHNAEVTGFIR.-	1273.38	3	3.05	0.43	1445.9	1	24/40		5.34E8
	75.50	-.EYQVQLDIAM*QSGKPK.-	1852.10	2	3.05	0.47	307.9	2	13/30		5.21E9
	73.93 - 75.14	-.EYQVQLDIAM*QSGKPK.-	1852.10	2	4.13	0.54	864.5	1	18/30		5.47E9
	61.93	-.FEVGEGIEK.-	1008.11	1	1.83	0.26	366.1	1	9/16		1.25E9
	130.00 - 130.72	-.FEVGEGIEKVETDFAAEVAAM*SK.-	2474.73	2	4.64	0.66	801.6	1	22/44		3.72E9
	129.96 - 131.13	-.FEVGEGIEKVETDFAAEVAAM*SK.-	2474.73	3	5.03	0.56	1657.7	1	34/88		6.85E9
	148.53	-.FEVGEGIEKVETDFAAEVAAMSK.-	2458.73	3	3.97	0.50	2703.5	1	37/88		1.50E9
	98.55	-.FTGEVSLTGQPFVM*EPSK.-	1971.22	2	2.60	0.45	307.6	5	12/34		2.14E9
	94.07 - 95.25	-.FTGEVSLTGQPFVM*EPSK.-	1971.22	2	5.33	0.58	1036.1	1	22/34		1.44E10
	103.92 - 104.50	-.FTGEVSLTGQPFVMEPSK.-	1955.22	2	5.21	0.46	1056.3	1	20/34		5.09E9
	76.25	-.HIAM*HVAASKPEFIKPEDVSAEVVEK.-	2879.28	2	3.03	0.50	219.7	1	14/50		1.94E9
	44.06 - 45.29	-.IGENINIR.-	929.06	2	3.02	0.32	1110.1	1	13/14		1.43E9
	38.86 - 40.09	-.IGENINIR.-	929.06	2	2.84	0.23	1006.2	2	13/14		4.31E8
	45.78	-.IGENINIR.-	929.06	1	1.86	0.03	428.6	1	11/14		5.67E8
	45.81 - 47.17	-.IGENINIR.-	929.06	2	2.80	0.32	1196.2	1	13/14		8.57E8

	36.86 - 38.24	-.IGENINIR.-	929.06	2	2.68	0.16	1289.8	2	13/14	2.56E8
	42.24 - 43.48	-.IGENINIR.-	929.06	2	2.91	0.27	1174.1	1	13/14	1.29E9
	37.89 - 47.15	-.IGENINIR.-	929.06	1	2.29	0.23	369.3	1	11/14	2.22E9
	41.64 - 44.25	-.IGENINIR.-	929.06	1	1.95	0.22	384.1	1	11/14	7.99E8
	57.13 - 58.02	-.ITDVEVLK.-	917.08	1	2.07	0.22	431.0	1	10/14	2.15E9
	88.70 - 89.36	-.KFTGEVSLTGQPFVM*EPSK.-	2099.39	2	4.14	0.55	798.9	1	18/36	2.79E9
	103.92 - 104.50	-.M*VEGRMKKFTGEVSLTGQPFVMEPSK.-	2931.44	3	3.11	0.02	467.1	1	24/100	5.09E9
	75.97 - 76.58	-.RVAALEGDVLGSYQHGAR.-	1900.09	2	4.49	0.51	544.0	1	18/34	3.32E9
	80.31 - 81.46	-.VAALEGDVLGSYQHGAR.-	1743.90	2	4.18	0.59	858.7	1	18/32	8.69E9
	80.73 - 81.36	-.VAALEGDVLGSYQHGAR.-	1743.90	3	4.08	0.51	1267.8	1	31/64	5.36E9
	80.94	-.VAALEGDVLGSYQHGAR.-	1743.90	1	2.31	0.32	190.5	2	11/32	1.30E9
#3	RL1_ECOLI (P02384) 5					290.25			29 (29 0 0 0 0)	3.22
	86.58 - 87.90	-.AAGAELVGM*EDLADQIK.-	1747.95	2	4.10	0.58	882.4	1	21/32	1.42E10
	88.47 - 89.06	-.AAGAELVGM*EDLADQIK.-	1747.95	2	5.00	0.62	987.0	1	21/32	7.26E9
	110.55	-.ENLEALLVALKK.-	1341.62	2	3.30	0.35	1038.5	1	16/22	2.40E9
	77.15 - 77.90	-.KGEM*NFDVVIASPDAM*R.-	1913.17	2	3.89	0.45	445.7	1	17/32	8.45E9
	87.71	-.KGEM*NFDVVIASPDAMR.-	1897.17	2	3.12	0.47	504.0	1	18/32	2.56E9
	91.97 - 92.30	-.KVSISTTM*GAGVAVDQAGLSASVN.-	2280.54	2	3.10	0.49	267.6	4	13/46	2.42E9
	140.62 - 141.25	-.LKENLEALLVALKK.-	1582.95	2	2.79	0.36	504.6	1	14/26	2.69E9
	117.76 - 118.31	-.QYDINEAIALLK.-	1391.60	2	3.10	0.32	347.1	1	15/22	9.36E9
	20.50 - 22.29	-.VAVFTQGANAEAAK.-	1377.53	2	4.41	0.45	1792.8	1	21/26	7.27E8
	52.72 - 53.97	-.VAVFTQGANAEAAK.-	1377.53	2	3.92	0.45	1198.0	1	18/26	1.13E9
	36.90 - 38.01	-.VAVFTQGANAEAAK.-	1377.53	2	4.21	0.36	1410.2	1	19/26	4.86E8
	18.24 - 19.92	-.VAVFTQGANAEAAK.-	1377.53	2	4.27	0.44	1757.0	1	22/26	9.25E8
	34.86 - 36.27	-.VAVFTQGANAEAAK.-	1377.53	2	4.44	0.36	1660.6	1	21/26	6.39E8
	16.07 - 17.68	-.VAVFTQGANAEAAK.-	1377.53	2	4.44	0.48	1906.1	1	22/26	9.27E8
	22.89 - 24.71	-.VAVFTQGANAEAAK.-	1377.53	2	4.11	0.50	1669.5	1	21/26	7.29E8
	32.98 - 34.31	-.VAVFTQGANAEAAK.-	1377.53	2	4.01	0.37	1874.7	1	21/26	1.16E9
	31.12 - 32.37	-.VAVFTQGANAEAAK.-	1377.53	2	4.53	0.50	1569.3	1	21/26	3.54E9
	30.31	-.VAVFTQGANAEAAK.-	1377.53	1	3.00	0.39	472.6	1	14/26	9.38E8
	27.42 - 28.81	-.VAVFTQGANAEAAK.-	1377.53	2	3.93	0.42	1518.5	1	20/26	2.23E9
	26.59 - 33.34	-.VAVFTQGANAEAAK.-	1377.53	1	2.75	0.49	582.2	1	15/26	2.36E9
	54.22	-.VAVFTQGANAEAAK.-	1377.53	1	2.73	0.44	568.4	1	13/26	4.45E8
	14.20 - 15.45	-.VAVFTQGANAEAAK.-	1377.53	2	4.06	0.47	1997.7	1	22/26	7.98E8
	25.68 - 26.80	-.VAVFTQGANAEAAK.-	1377.53	2	3.86	0.48	1876.3	1	21/26	1.31E9
	42.37 - 43.27	-.VDFDADKLK.-	1051.18	2	2.54	0.31	974.4	1	13/16	3.80E8
	151.06 - 151.75	-.VDFDADKLKENLEALLVALK.-	2245.60	3	4.11	0.51	1713.5	1	34/76	2.63E9
	140.62 - 141.25	-.VDFDADKLKENLEALLVALKK.-	2373.77	3	4.15	0.40	615.9	1	24/80	2.79E9
	63.60 - 65.03	-.VGTVTPNVAEAVK.-	1285.47	2	2.95	0.40	661.4	1	17/24	1.30E10
	79.09 - 79.74	-.VVGQLGQVLGPR.-	1223.45	1	2.28	0.52	264.5	1	13/22	5.92E9
	79.05 - 80.28	-.VVGQLGQVLGPR.-	1223.45	2	3.51	0.57	1960.1	1	20/22	9.86E9
#4	GPMA_ECO57 (P6270					220.25			22 (22 0 0 0 0)	6.93
	160.24 - 161.15	-.AIHTLWNVLDELDQAWLPVEK.-	2491.83	3	3.20	0.24	371.5	8	24/80	9.27E9
	117.11 - 118.40	-.ELPLTESLALTIDR.-	1571.80	2	4.98	0.59	928.2	1	18/26	3.33E10
	118.14 - 119.50	-.ELPLTESLALTIDR.-	1571.80	1	2.75	0.41	130.1	1	14/26	5.02E9
	102.68	-.FTGWYDVDLSEK.-	1460.57	2	2.87	0.63	832.2	1	17/22	8.92E9
	101.47	-.FTGWYDVDLSEK.-	1460.57	1	3.30	0.53	554.5	1	14/22	3.57E9
	100.83 - 102.04	-.FTGWYDVDLSEK.-	1460.57	1	2.34	0.37	481.9	1	13/22	5.25E9
	100.71 - 102.13	-.FTGWYDVDLSEK.-	1460.57	2	3.99	0.51	975.5	1	18/22	1.09E10
	83.49 - 84.44	-.GFAVTPPELTK.-	1160.35	1	2.05	0.42	280.6	1	10/20	1.07E10
	11.19 - 12.49	-.HYGALQGLNK.-	1101.24	1	2.75	0.31	488.4	1	12/18	8.35E9
	114.03 - 115.22	-.LLKEEGYSFDFAYTSVLK.-	2111.38	2	4.67	0.56	1269.8	1	21/34	5.56E9
	106.74	-.LLKEEGYSFDFAYTSVLKR.-	2267.57	3	3.02	0.51	798.6	2	24/72	3.07E9
	113.00 - 114.17	-.LSEKELPLTESLALTIDR.-	2029.32	2	4.50	0.55	444.3	1	19/34	8.83E9
	113.06 - 114.34	-.LSEKELPLTESLALTIDR.-	2029.32	3	3.58	0.34	1286.5	1	28/68	6.63E9
	75.83 - 76.41	-.RGFAVTPPELTK.-	1316.53	2	3.22	0.38	973.1	1	16/22	6.83E9
	109.80 - 111.04	-.VIPYWNETILPR.-	1501.75	2	3.16	0.55	410.2	1	16/22	3.02E10
	110.59 - 111.29	-.VIPYWNETILPR.-	1501.75	1	2.54	0.28	571.2	1	14/22	6.66E9
	77.25 - 78.12	-.YYLGNADEIAAK.-	1328.45	2	3.60	0.57	1023.0	1	19/22	4.93E9
	78.07	-.YYLGNADEIAAK.-	1328.45	1	2.33	0.56	818.6	1	15/22	3.63E9
	73.52 - 75.25	-.YYLGNADEIAAK.-	1328.45	1	1.80	0.15	619.2	1	13/22	1.32E10
	73.14 - 73.98	-.YYLGNADEIAAK.-	1328.45	2	3.23	0.47	1166.5	1	19/22	1.57E10
	67.00	-.YYLGNADEIAAK.-	1328.45	2	3.30	0.46	982.4	1	17/22	1.42E9
	74.70 - 75.75	-.YYLGNADEIAAK.-	1328.45	2	3.51	0.49	1061.4	1	19/22	1.81E10
#5	RS4_ECOLI (P02354)					210.28			21 (21 0 0 0 0)	2.17
	24.43 - 24.95	-.AALELAEQR.-	1001.12	2	2.52	0.30	758.6	1	11/16	2.37E8
	19.77 - 21.57	-.AALELAEQR.-	1001.12	2	2.56	0.30	851.5	1	13/16	6.02E8
	17.55 - 19.20	-.AALELAEQR.-	1001.12	2	3.12	0.30	828.8	1	13/16	7.81E8
	15.67 - 17.01	-.AALELAEQR.-	1001.12	2	2.62	0.27	736.0	1	12/16	1.03E9
	14.08 - 23.83	-.AALELAEQR.-	1001.12	1	2.32	0.26	311.2	1	10/16	8.63E8
	13.91 - 15.05	-.AALELAEQR.-	1001.12	2	2.73	0.28	855.7	1	13/16	9.10E8
	22.09 - 23.50	-.AALELAEQR.-	1001.12	2	2.73	0.34	718.9	1	12/16	3.23E8
	81.11	-.EKPTWLEVDAGKM*EGTFK.-	2083.35	2	3.39	0.54	384.8	2	13/34	2.81E9
	81.05 - 81.61	-.EKPTWLEVDAGKM*EGTFK.-	2083.35	3	3.58	0.35	647.8	1	28/68	3.51E9

	127.42 - 128.02	-.GNTGENLLALLEGR.-	1457.62	2	3.95	0.56	1226.0	1	17/26	4.03E9
	57.93 - 59.70	-.IYGVLER.-	850.00	1	1.81	0.20	244.8	3	9/12	1.68E9
	117.78 - 118.63	-.LKGNTGENLLALLEGR.-	1698.95	2	5.32	0.55	1562.5	1	21/30	6.53E9
	118.17 - 118.77	-.LKGNTGENLLALLEGR.-	1698.95	3	3.84	0.28	1019.4	1	26/60	3.81E9
	124.45	-.LKGNTGENLLALLEGR.-	1698.95	2	2.71	0.48	747.5	1	18/30	2.78E9
	68.60 - 69.17	-.LSDYGVQLR.-	1051.18	2	3.27	0.28	1280.9	1	15/16	2.70E9
	68.58 - 69.15	-.LSDYGVQLR.-	1051.18	1	1.83	0.09	194.2	3	10/16	4.27E9
	60.03 - 61.27	-.REGTDLFLK.-	1079.23	2	2.73	0.33	678.3	1	12/16	1.84E9
	12.95 - 13.87	-.RIYGVLER.-	1006.18	2	2.96	0.27	637.6	3	11/14	1.46E9
	120.89	-.SDLSADINEHLIVELYSK.-	2047.25	2	3.01	0.37	362.0	1	13/34	2.84E9
	102.11 - 103.23	-.VVNIASYQVSPNDVVSIR.-	1961.21	2	5.69	0.60	1059.5	1	22/34	2.33E10
	102.91	-.VVNIASYQVSPNDVVSIR.-	1961.21	3	4.45	0.60	2644.1	1	34/68	2.65E9
#6	RS3_ECOLI (P02352)					200.27			20 (20 0 0 0 0)	3.69
	83.34 - 84.23	-.ADIDYNTSEAHTTYGVIGVK.-	2155.31	2	4.30	0.59	1282.6	1	21/38	4.48E9
	80.39 - 81.23	-.ADIDYNTSEAHTTYGVIGVK.-	2155.31	2	5.28	0.60	1329.4	1	22/38	7.09E9
	84.59 - 85.12	-.EFADNLDSDFK.-	1301.34	1	1.93	0.30	293.6	1	15/20	5.32E9
	84.55 - 85.40	-.EFADNLDSDFK.-	1301.34	2	2.72	0.51	799.5	1	14/20	6.21E9
	60.91	-.GEILGGM*AAVEQPEKPAAQPK.-	2138.43	2	3.07	0.44	484.1	1	17/40	3.05E9
	63.62 - 64.89	-.GEILGGM*AAVEQPEKPAAQPK.-	2138.43	2	4.04	0.63	324.8	1	16/40	1.72E9
	57.48 - 58.57	-.GEILGGM*AAVEQPEKPAAQPK.-	2138.43	2	3.81	0.58	425.0	1	18/40	3.87E9
	58.14 - 59.27	-.GEILGGM*AAVEQPEKPAAQPK.-	2138.43	3	3.86	0.48	730.7	1	30/80	4.87E9
	59.15 - 60.32	-.GEILGGM*AAVEQPEKPAAQPK.-	2138.43	2	4.10	0.65	651.6	1	20/40	8.16E9
	78.24 - 78.36	-.GEILGGMAAVEQPEKPAAQPK.-	2122.43	2	4.59	0.54	961.5	1	20/40	2.91E9
	99.52 - 100.69	-.KVVADIAGVPAQINIAEVR.-	1964.30	2	5.49	0.65	707.5	1	22/36	1.41E10
	99.54 - 100.12	-.KVVADIAGVPAQINIAEVR.-	1964.30	3	4.09	0.50	1327.9	1	35/72	4.67E9
	107.21 - 107.76	-.LGIVKPWNSTWFANTK.-	1863.15	2	3.76	0.62	327.7	1	16/30	5.02E9
	107.30	-.LGIVKPWNSTWFANTK.-	1863.15	3	4.04	0.54	1532.4	1	28/60	2.95E9
	84.32 - 85.01	-.LVADSITSQLER.-	1332.49	2	4.61	0.60	1507.9	1	19/22	1.24E10
	12.04	-.PGIVIGK.-	683.86	1	1.87	0.25	396.7	1	8/12	1.51E9
	60.45 - 61.75	-.VTIHTARPGIVIGK.-	1462.77	2	2.60	0.38	627.8	1	13/26	3.24E9
	56.90 - 58.12	-.VTIHTARPGIVIGK.-	1462.77	2	2.58	0.45	633.2	1	13/26	2.63E9
	107.09	-.VVADIAGVPAQINIAEVR.-	1836.13	3	4.76	0.41	2602.1	1	38/68	3.10E9
	106.95 - 107.39	-.VVADIAGVPAQINIAEVR.-	1836.13	2	4.14	0.57	440.0	1	19/34	1.98E10
#7	EFTU_ECOLI (P02990					190.24			19 (19 0 0 0 0)	3.30
	72.74 - 73.29	-.AGENVGVLLR.-	1028.19	2	3.00	0.13	782.8	1	15/18	2.62E9
	140.16 - 141.32	-.AIDKPFLLPIEDVFSISGR.-	2118.46	2	4.89	0.56	668.2	1	20/36	7.47E9
	56.65	-.ALEGDAEWEAK.-	1219.28	1	1.94	0.35	335.9	3	10/20	9.59E8
	55.98 - 57.23	-.ALEGDAEWEAK.-	1219.28	2	3.60	0.56	1134.4	1	17/20	2.61E9
	137.62 - 137.81	-.CDM*VDDEELLELVEM*EVR.-	2257.47	2	4.13	0.48	720.9	1	15/34	2.10E9
	11.43 - 12.35	-.EHILLGR.-	837.99	1	2.20	0.36	371.7	2	9/12	2.97E9
	106.37 - 107.51	-.ELLSQYDFPGDDTPIVR.-	1966.14	2	4.68	0.63	863.7	1	21/32	2.11E10
	86.77	-.FESEVYILSK.-	1215.38	2	3.37	0.43	861.8	1	16/18	2.85E9
	81.69	-.FESEVYILSKDEGGR.-	1729.87	2	2.82	0.49	419.4	1	11/28	2.63E9
	71.12	-.GITINTSHVEYDTPTR.-	1804.94	2	4.02	0.61	868.8	1	17/30	3.68E9
	143.55 - 144.31	-.ILELAGFLDSYIPEPER.-	1963.22	2	4.66	0.58	675.9	1	19/32	1.29E10
	141.97 - 142.96	-.ILELAGFLDSYIPEPER.-	1963.22	2	4.80	0.53	868.0	1	21/32	1.61E10
	88.09	-.M*VVTLIHPIAM*DDGLR.-	1814.16	3	3.20	0.44	659.9	1	25/60	4.23E9
	88.03 - 89.18	-.M*VVTLIHPIAM*DDGLR.-	1814.16	2	3.31	0.26	519.8	1	16/30	5.47E9
	95.65 - 96.46	-.MVVTLIHPIAM*DDGLR.-	1798.16	2	3.13	0.36	843.1	1	16/30	3.92E9
	11.87	-.STCTGVEM*FR.-	1204.33	2	2.68	0.42	719.5	1	13/18	2.63E9
	114.76	-.TTLTAAITTVLAK.-	1304.56	2	3.96	0.54	1637.5	1	19/24	3.15E9
	80.77 - 81.71	-.VGEEVEIVGIK.-	1172.35	2	3.37	0.33	1883.9	1	17/20	4.57E9
	72.96	-.VGEEVEIVGIKETQK.-	1658.88	2	4.00	0.59	1139.3	1	18/28	2.83E9
#8	OMPC_ECO57 (Q8XE					190.23			19 (19 0 0 0 0)	1.82
	129.94	-.AQNFEAVAQYQFDFGLRPSLAYLQSK.-	2993.32	2	3.54	0.22	297.0	1	15/50	1.42E9
	128.51 - 129.74	-.AQNFEAVAQYQFDFGLRPSLAYLQSK.-	2993.32	3	3.84	0.47	726.1	1	27/100	4.67E9
	130.30 - 130.84	-.AQNFEAVAQYQFDFGLRPSLAYLQSK.-	2993.32	3	4.45	0.41	958.1	1	30/100	4.98E9
	116.95	-.EALRQNGDGVGGSITYDYEGFGIGAAVSSSK	3107.29	3	3.44	0.30	582.2	2	32/120	3.46E9
	119.96 - 121.27	-.EALRQNGDGVGGSITYDYEGFGIGAAVSSSK	3107.29	3	3.40	0.18	1017.0	1	31/120	6.10E9
	77.40 - 78.01	-.FQDVGSFDYGR.-	1291.35	2	3.13	0.46	1293.6	1	18/20	4.80E9
	93.18	-.INLLDDNQFTR.-	1349.48	1	1.88	0.26	418.4	1	12/20	3.79E9
	93.06 - 93.72	-.INLLDDNQFTR.-	1349.48	2	3.33	0.46	1088.7	1	16/20	7.30E9
	65.95	-.NYDDEDILK.-	1125.17	1	2.84	0.33	383.3	1	12/16	2.41E9
	65.69 - 66.28	-.NYDDEDILK.-	1125.17	1	2.31	0.09	294.1	1	11/16	3.78E9
	56.92 - 58.17	-.RTDDQNSPLYIGNGDR.-	1821.89	2	4.02	0.51	1244.5	1	22/30	1.53E9
	80.96 - 82.17	-.RTDDQNSPLYIGNGDRAETYTGGLK.-	2742.90	3	4.07	0.50	1269.2	1	33/96	2.85E9
	68.06 - 68.64	-.TDDQNSPLYIGNGDR.-	1665.70	2	3.92	0.47	1448.4	1	20/28	2.58E9
	87.25 - 88.13	-.TDDQNSPLYIGNGDRAETYTGGLK.-	2586.71	2	3.77	0.62	504.7	1	19/46	2.56E9
	76.37	-.VDGLHYFSDDKSVDGDQTYM*R.-	2465.60	3	4.66	0.67	1872.4	1	33/80	2.09E9
	49.99 - 50.88	-.VGSLGWANK.-	932.06	1	2.01	0.28	331.1	1	10/16	8.48E8
	48.88 - 50.13	-.VGSLGWANK.-	932.06	2	2.82	0.46	889.4	1	14/16	7.12E8
	48.77 - 51.63	-.VGSLGWANK.-	932.06	1	1.99	0.28	292.0	1	10/16	1.39E9
	50.70 - 51.91	-.VGSLGWANK.-	932.06	2	2.90	0.48	993.2	1	15/16	5.86E8
#9	RS2_ECOLI (P02351)					170.27			17 (17 0 0 0 0)	2.83

	84.49 - 85.30	-.DAALSCDQFFVNHR.-	1680.80	2	4.27	0.59	1809.8	1	19/26		3.43E9
	82.09 - 83.45	-.DAALSCDQFFVNHR.-	1680.80	2	3.19	0.47	1151.1	1	17/26		6.37E9
	74.16	-.DLETQSQDGTFDKLTK.-	1826.94	2	3.69	0.44	695.1	1	15/30		4.08E9
	80.05	-.ELEKLENSLGGIK.-	1430.63	2	3.29	0.46	1115.4	1	19/24		2.47E9
	68.39 - 69.51	-.ILFVGTK.-	777.97	1	2.03	0.32	534.8	1	10/12		4.59E9
	39.20 - 40.36	-.ILFVGTKR.-	934.16	2	2.75	0.32	545.1	2	11/14		3.59E8
	116.29 - 117.37	-.LENSLGGIKDM*GGLPDALFVIDADHEHIAIK.-	3306.74	3	5.47	0.56	1506.4	1	36/120		6.28E9
	11.64	-.LKDLETQSQDGTFDK.-	1725.84	2	4.76	0.53	1390.6	1	19/28		2.24E9
	70.66 - 71.21	-.LKDLETQSQDGTFDKLTK.-	2068.27	2	4.71	0.57	1421.7	1	23/34		3.04E9
	70.47 - 71.83	-.LKDLETQSQDGTFDKLTK.-	2068.27	3	3.95	0.36	1566.9	1	34/68		3.93E9
	105.58	-.SQDLASQAEESFVEAE.-	1740.76	1	3.17	0.46	361.6	1	12/30		3.67E9
	102.66 - 103.27	-.TVPM*FNEALAELNK.-	1593.83	1	2.10	0.41	76.6	1	11/26		3.28E9
	102.74	-.TVPM*FNEALAELNK.-	1593.83	1	2.05	0.19	55.1	9	10/26		3.02E9
	102.89 - 103.46	-.TVPM*FNEALAELNK.-	1593.83	2	2.91	0.52	235.4	1	16/26		1.79E10
	100.94 - 102.34	-.TVPM*FNEALAELNK.-	1593.83	2	2.60	0.50	88.0	9	12/26		2.27E10
	45.62 - 46.81	-.VHIINLEK.-	966.16	2	2.50	0.38	591.5	1	13/14		8.83E8
	41.41 - 48.65	-.VHIINLEK.-	966.16	1	2.38	0.35	531.5	2	11/14		1.61E9
#10	DCEA_ECO57 (P5822					170.24			17 (17 0 0 0 0)		1.66
	109.72	-.CVNMVADLWHAPAPK.-	1709.99	2	2.96	0.56	921.3	1	17/28	1	3.17E9
	142.22	-.GFEM*DFAELLLEDYK.-	1837.04	2	3.70	0.49	1985.0	1	21/28	1	2.18E9
	112.77 - 113.94	-.LGPYEFICTGRPDEGIPAVCFK.-	2527.84	3	3.29	0.34	719.3	1	27/84	1	7.38E9
	114.49	-.LGPYEFICTGRPDEGIPAVCFK.-	2527.84	3	4.31	0.57	1634.9	1	34/84	1	4.06E9
	89.24	-.LKEGEDPGYTLYDLSER.-	1986.13	2	3.73	0.55	460.1	1	18/32		1.80E9
	34.08 - 35.22	-.LQGIAQQNSFK.-	1234.39	2	3.17	0.46	713.8	1	16/20	1	5.64E8
	39.47 - 40.12	-.LQGIAQQNSFK.-	1234.39	2	3.20	0.52	792.6	1	16/20	1	3.29E8
	32.17 - 33.46	-.LQGIAQQNSFK.-	1234.39	2	3.11	0.55	720.3	1	15/20	1	6.66E8
	37.74 - 38.69	-.LQGIAQQNSFK.-	1234.39	2	2.74	0.47	731.5	1	15/20	1	4.78E8
	35.73 - 37.13	-.LQGIAQQNSFK.-	1234.39	2	3.41	0.48	895.9	1	17/20	1	4.99E8
	93.08	-.NWIDKEEYPQSAAIDLR.-	2049.23	3	3.54	0.38	1375.8	1	28/64	1	2.07E9
	92.99	-.NWIDKEEYPQSAAIDLR.-	2049.23	2	4.63	0.64	1307.0	1	21/32	1	2.98E9
	115.68 - 116.80	-.PAGQVIAQYYEFLR.-	1655.88	2	3.80	0.47	1005.1	1	16/26	1	9.37E9
	86.60 - 86.79	-.QNLATFCQTWDDENVHK.-	2107.22	2	3.82	0.61	623.8	1	16/32	1	1.98E9
	129.17 - 130.06	-.RGFEM*DFAELLLEDYK.-	1993.23	2	3.49	0.43	937.5	1	20/30	1	5.51E9
	122.94 - 124.31	-.VQNASYQVAAYLADEIAK.-	1955.16	2	2.98	0.53	493.7	1	14/34	1	4.97E9
	120.63 - 121.92	-.VQNASYQVAAYLADEIAK.-	1955.16	2	4.86	0.53	1406.7	1	20/34	1	4.74E9
#11	RL2_ECO57 (P60424)					160.25			16 (16 0 0 0 0)		2.11
	79.30 - 79.87	-.AGDQIQSGVDAAIKPGNTLPM*R.-	2256.53	2	3.86	0.58	280.6	1	16/42		8.58E9
	80.03 - 81.25	-.AGDQIQSGVDAAIKPGNTLPM*R.-	2256.53	2	4.58	0.68	315.7	1	16/42		9.52E9
	79.28 - 79.99	-.AGDQIQSGVDAAIKPGNTLPM*R.-	2256.53	3	5.01	0.48	950.5	1	32/84		1.20E10
	64.78	-.ATLGEVGNAEHM*LR.-	1514.69	2	2.89	0.33	1216.5	1	18/26		5.55E9
	63.25 - 64.22	-.ATLGEVGNAEHM*LR.-	1514.69	2	3.34	0.41	729.0	1	15/26		6.43E9
	53.20 - 54.43	-.ATLGEVGNAEHM*LR.-	1514.69	2	3.09	0.47	850.4	1	17/26		1.17E9
	31.78 - 38.92	-.DGAYVTLR.-	895.00	1	1.91	0.30	195.2	1	10/14		1.56E9
	32.52 - 33.85	-.DGAYVTLR.-	895.00	2	2.57	0.50	989.1	1	13/14		5.34E8
	34.49 - 35.82	-.DGAYVTLR.-	895.00	2	2.63	0.42	1108.7	1	13/14		6.50E8
	33.08 - 34.21	-.HPVTPWGVQTK.-	1250.43	2	2.72	0.54	572.0	1	16/20		9.70E8
	63.42	-.NFGKHPVTPWGVQTK.-	1696.93	2	4.07	0.56	523.6	1	16/28		1.63E9
	10.87	-.NIPVGSTVHNVEM*KPGK.-	1824.10	2	3.90	0.57	653.8	1	18/32		4.23E9
	68.69 - 69.27	-.SAGTYVQIVAR.-	1165.33	2	3.09	0.46	1127.2	1	16/20		3.28E9
	71.52	-.SAGTYVQIVAR.-	1165.33	2	2.68	0.51	928.3	1	15/20		2.52E9
	90.76 - 91.51	-.SANIALVLYK.-	1092.31	1	1.88	0.28	572.6	1	13/18		4.78E9
	90.71 - 91.36	-.SANIALVLYK.-	1092.31	2	3.54	0.66	1088.3	1	16/18		3.56E9
#12	YNCE_ECO57 (Q8X9X					150.27			15 (15 0 0 0 0)		2.21
	57.79 - 59.12	-.AAEVLVVDTR.-	1073.23	2	4.02	0.48	1552.9	1	15/18		1.60E9
	57.21 - 59.10	-.AAEVLVVDTR.-	1073.23	1	1.87	0.43	451.0	1	10/18		1.48E9
	103.39 - 103.50	-.DSVIWVVDGENIK.-	1474.64	1	2.92	0.43	583.7	1	14/24		2.31E9
	93.20 - 94.30	-.ELVADDATNTVYISGIGK.-	1867.05	2	5.05	0.67	1344.0	1	21/34		6.69E9
	91.15 - 92.49	-.ELVADDATNTVYISGIGK.-	1867.05	2	4.36	0.65	1588.9	1	22/34		7.72E9
	88.95 - 90.34	-.LYTTNADGELITIDTADNK.-	2069.21	2	5.08	0.53	1020.7	1	19/36		1.05E10
	90.94 - 92.16	-.LYTTNADGELITIDTADNK.-	2069.21	2	4.45	0.64	1094.4	1	19/36		5.11E9
	204.15 - 205.68	-.LYTTNADGELITIDTADNK.-	2069.21	2	2.59	0.52	396.1	2	12/36		1.59E8
	93.30 - 94.18	-.LYTTNADGELITIDTADNK.-	2069.21	2	5.03	0.63	1464.9	1	22/36		4.39E9
	11.45	-.M*STGLALDSK.-	1039.19	1	1.80	0.06	247.7	1	13/18		2.11E9
	83.72	-.RLYTTNADGELITIDTADNK.-	2225.40	2	5.32	0.50	1503.2	1	23/38		1.88E9
	73.10 - 73.64	-.TFDTPTHPNSLALSADGK.-	1873.01	2	3.85	0.56	649.3	1	19/34		5.04E9
	75.68	-.TFDTPTHPNSLALSADGK.-	1873.01	2	4.36	0.56	720.7	1	19/34		4.14E9
	117.30	-.VAAPESLAVLFNPAR.-	1555.80	1	2.32	0.38	324.1	1	12/28		1.95E9
	117.07 - 118.42	-.VAAPESLAVLFNPAR.-	1555.80	2	4.62	0.57	2313.4	1	22/28		1.50E10
#13	G3P1_ECOLI (P06977					140.26			14 (14 0 0 0 0)		2.11
	98.36 - 98.94	-.GANFDKYAGQDIVSNASCTTNCLAPLAK.-	2988.24	3	5.01	0.60	869.7	1	35/108		3.74E9
	12.00	-.GASQNIIPSSTGAAK.-	1402.54	1	1.94	0.27	671.6	1	17/28		2.49E9
	11.49 - 12.10	-.GASQNIIPSSTGAAK.-	1402.54	2	2.82	0.36	696.7	1	18/28		4.31E9
	79.10 - 80.54	-.LVSWYDNETGYSNK.-	1676.77	2	2.98	0.44	761.8	1	15/26		5.94E9
	80.58	-.LVSWYDNETGYSNK.-	1676.77	1	3.05	0.54	303.4	1	13/26		1.49E9

	139.43	-.RSDIEIVAINDLLDADYM*AYM*LK.-	2706.09	3	3.54	0.48	1343.2	1	30/88	2.36E9
	118.58 - 119.79	-.VINDNFGIIEGLM*TTVHATTATQK.-	2591.92	3	4.76	0.48	483.7	1	32/92	7.40E9
	116.70 - 117.28	-.VINDNFGIIEGLM*TTVHATTATQK.-	2591.92	2	3.45	0.51	226.4	6	11/46	3.75E9
	116.68 - 117.85	-.VINDNFGIIEGLM*TTVHATTATQK.-	2591.92	3	5.18	0.54	397.8	2	26/92	9.21E9
	135.55	-.VINDNFGIIEGLMTTVHATTATQK.-	2575.92	3	4.34	0.62	894.6	1	32/92	2.33E9
	85.67	-.VLDLIAHISK.-	1109.34	2	2.87	0.40	803.5	1	14/18	1.63E9
	84.72 - 85.42	-.VLDLIAHISK.-	1109.34	1	1.89	0.34	358.8	1	11/18	3.26E9
	97.20 - 98.51	-.VPTPNVSVVDLTVR.-	1496.73	1	1.80	0.33	165.7	9	10/26	3.19E9
	96.57 - 97.78	-.VPTPNVSVVDLTVR.-	1496.73	2	3.68	0.55	1598.3	1	19/26	1.59E10
#14	Q8X8Y7 (Q8X8Y7) 2,3,					130.38			13 (13 0 0 0 0)	1.04
	130.24 - 131.11	-.EAVNQVIALLDSGALR.-	1669.90	2	3.97	0.44	1046.8	1	17/30	4.96E9
	131.36	-.EAVNQVIALLDSGALR.-	1669.90	3	4.52	0.50	2301.7	1	34/60	1.41E9
	94.91	-.IDGQWVTHQWLK.-	1511.71	2	3.36	0.36	734.5	1	15/22	2.90E9
	59.38 - 59.99	-.INDNQVIEGAESR.-	1445.52	2	3.33	0.42	1204.8	1	18/24	2.85E9
	57.62 - 58.81	-.INDNQVIEGAESR.-	1445.52	2	3.93	0.49	1261.7	1	19/24	3.61E9
	120.28 - 120.83	-.M*QQLQNIIETAFER.-	1737.96	2	4.80	0.64	2256.0	1	21/26	7.41E9
	126.14	-.NVHLSGGVGIGGVLEPLQANPTIIEDNCFIGAI	3419.83	3	6.76	0.59	676.7	1	31/128	2.20E9
	128.00 - 128.55	-.NVHLSGGVGIGGVLEPLQANPTIIEDNCFIGAI	3419.83	3	7.58	0.60	1833.7	1	44/128	2.47E9
	66.97 - 67.60	-.VPAGSVVVSGNLPSK.-	1411.63	2	3.66	0.65	1425.4	1	19/28	2.23E9
	67.02	-.VPAGSVVVSGNLPSK.-	1411.63	1	1.97	0.54	176.4	10	10/28	7.68E8
	47.26 - 48.45	-.VPAGSVVVSGNLPSKDGK.-	1711.94	2	3.80	0.62	407.9	1	15/34	6.14E8
	45.34 - 46.73	-.VPAGSVVVSGNLPSKDGK.-	1711.94	2	4.30	0.53	600.5	1	18/34	4.20E8
	49.03 - 50.34	-.VPAGSVVVSGNLPSKDGK.-	1711.94	2	4.30	0.62	363.8	1	15/34	1.11E9
#15	MDH_ECO57 (P61891					120.36			12 (12 0 0 0 0)	1.54
	157.77 - 159.03	-.ACIGIITNPVNTTVAIAAEVLK.-	2269.66	2	6.07	0.67	548.7	1	18/42	1.69E9
	147.16 - 147.77	-.ACIGIITNPVNTTVAIAAEVLKK.-	2397.83	3	4.00	0.48	1084.9	1	35/88	3.85E9
	88.53	-.DIALGEEFVNK.-	1235.37	2	3.99	0.40	1270.7	1	17/20	2.41E9
	100.65	-.FFSQPLLLGK.-	1150.40	2	2.63	0.54	684.3	1	15/18	1.74E9
	95.63 - 96.25	-.FFSQPLLLGKNGVEER.-	1835.10	2	3.74	0.47	1178.5	1	19/30	3.18E9
	119.09 - 119.71	-.LFGVTTLDIIR.-	1248.50	2	3.63	0.53	1556.3	1	18/20	3.09E9
	94.33	-.SDLFNVNAGIVK.-	1277.45	2	4.32	0.50	1545.4	1	19/22	3.16E9
	146.19	-.SIGTLSAFEQNALEGMLDTLKK.-	2367.71	3	3.34	0.44	1281.9	1	36/84	1.17E9
	131.71 - 133.14	-.TQLPSGSELSLYDIAPVTPGVAVDLSHIPTAVI	3377.83	3	5.33	0.54	1214.0	1	40/128	2.48E10
	132.07	-.TQLPSGSELSLYDIAPVTPGVAVDLSHIPTAVI	3377.83	2	3.14	0.53	197.3	1	16/64	1.34E9
	160.73	-.VAVLGAAGGIGQALALLLK.-	1736.14	3	7.26	0.51	3527.4	1	37/72	4.61E8
	160.49 - 161.99	-.VAVLGAAGGIGQALALLLK.-	1736.14	2	6.41	0.63	1968.3	1	25/36	2.20E9
#16	PTNA_ECOLI (P08186					120.29			12 (12 0 0 0 0)	1.36
	138.94 - 140.18	-.DDDPSFDELVALAVETGR.-	1950.05	2	4.83	0.60	1421.6	1	21/34	5.36E9
	135.95 - 136.48	-.GVLFLVDTWGGSPFNAASR.-	1995.23	2	5.76	0.61	1608.4	1	24/36	5.25E9
	74.91	-.IIVVSDEVAADTVRK.-	1615.85	2	3.81	0.62	1019.5	1	18/28	1.90E9
	70.32 - 70.89	-.ITSVNVGGM*AFR.-	1268.47	2	2.73	0.54	786.4	1	16/22	3.20E9
	133.43 - 134.08	-.IVVDKEHYEVIAGVNIPM*LVETLM*AR.-	2973.50	3	3.46	0.23	481.2	2	28/100	2.10E9
	90.27	-.KTLLTQVAPPGVTAHVVDVAK.-	2145.53	2	3.61	0.53	403.2	1	16/40	1.81E9
	10.51	-.LIHGQVATR.-	995.16	1	2.05	0.30	261.9	10	10/16	2.23E9
	135.93	-.TAEM*LLGEQENVGWIDFVPGENAETLIEK.-	3250.58	2	3.19	0.38	243.1	1	15/56	2.12E9
	99.31	-.TLLTQVAPPGVTAHVVDVAK.-	2017.36	3	3.86	0.45	867.2	1	31/76	4.64E9
	99.29	-.TLLTQVAPPGVTAHVVDVAK.-	2017.36	2	3.45	0.60	302.7	1	17/38	5.62E9
	89.22	-.TQVNNAVSVDEKDIEAFK.-	2008.18	2	2.81	0.33	422.7	1	14/34	2.99E9
	98.62 - 99.17	-.VM*LLFTNPTDVER.-	1551.79	2	4.13	0.53	2292.0	1	20/24	6.02E9
#17	ENO_ECOLI (P08324)					120.28			12 (12 0 0 0 0)	1.70
	141.34 - 141.91	-.AFTSEEFTHFLEELTK.-	1930.10	2	5.02	0.54	1344.4	1	19/30	4.34E9
	124.19 - 124.75	-.AKGM*NTAVGDEGGYAPNLGSNAEALAVIAE	3206.53	3	5.57	0.59	1916.4	1	42/128	5.14E9
	12.23 - 13.33	-.DAGYTAVISHR.-	1190.29	2	2.96	0.59	1166.9	1	16/20	2.18E9
	121.09	-.FNQIGSLTETLAAIK.-	1606.85	2	2.90	0.32	886.1	1	18/28	2.68E9
	118.52	-.FNQIGSLTETLAAIK.-	1606.85	2	4.03	0.41	1277.7	1	23/28	5.59E9
	116.84 - 117.94	-.FNQIGSLTETLAAIK.-	1606.85	2	4.86	0.58	1182.9	1	22/28	5.65E9
	130.78 - 132.10	-.GM*NTAVGDEGGYAPNLGSNAEALAVIAEAV	3007.28	2	4.31	0.66	365.2	1	21/60	5.03E9
	84.25	-.GM*PLYEHIAELNGTPGK.-	1844.08	2	4.27	0.57	2204.0	1	23/32	4.42E9
	133.24	-.GMNTAVGDEGGYAPNLGSNAEALAVIAEAVK	2991.28	2	2.97	0.48	112.1	2	12/60	1.91E9
	105.91 - 106.27	-.IQLVGDDLFVTNTK.-	1563.78	2	3.53	0.42	1011.5	1	17/26	5.53E9
	106.08	-.IQLVGDDLFVTNTK.-	1563.78	1	3.11	0.51	392.7	1	14/26	2.11E9
	107.41 - 107.97	-.SGETEDATIADLAVGTAAGQIK.-	2119.27	2	5.23	0.50	981.6	1	19/42	9.36E9
#18	Q8XEB4 (Q8XEB4) For					120.25			12 (12 0 0 0 0)	1.07
	120.56 - 121.07	-.DAIPTQSVLTITSNVVYGK.-	2007.27	2	3.87	0.59	625.4	1	16/36	5.37E9
	92.83	-.EM*LLDAM*ENPEKYPQLTIR.-	2324.66	2	2.90	0.45	132.5	4	13/36	2.44E9
	105.62	-.IFTEYRKTHNQGVFDVYTPDILR.-	2814.15	3	3.54	0.50	550.8	2	22/88	3.78E9
	134.27 - 135.50	-.NYTPYEGDESFLAGATEATTTLWDK.-	2781.92	2	4.20	0.66	660.6	1	19/48	2.86E9
	84.63	-.SEPIKGDLLNYDEVM*ER.-	2025.23	2	4.07	0.53	1052.9	1	20/32	2.38E9
	86.64	-.SGVLTGLPDAYGR.-	1306.45	2	3.14	0.35	799.9	1	16/24	1.88E9
	99.93 - 100.22	-.THAPVDFDTAVASTITSHDAGYINK.-	2632.82	3	4.92	0.60	579.7	1	30/96	2.84E9
	105.62	-.THNQGVFDVYTPDILR.-	1876.06	2	5.00	0.69	1831.0	1	21/30	3.78E9
	114.28 - 114.87	-.TM*ACGIAGLSVAADSLSAIK.-	1953.24	2	4.11	0.58	715.9	1	19/38	3.88E9
	99.80	-.VALYGIDYLM*K.-	1302.56	2	3.01	0.43	1022.3	1	17/20	1.69E9
	92.61	-.VDDLAVDLVER.-	1244.38	2	3.58	0.40	1805.9	1	18/20	2.52E9

	53.22 - 53.46	-.VVGLQTEAPLKR.-	1311.56	2	2.79	0.38	863.4	1	15/22	6.53E8
#19	Q8X8L3 (Q8X8L3) Uric					110.36			11 (11 0 0 0 0)	1.54
	135.10 - 136.00	-.AELDGKPVIVCSTGIGGPSTSIAVEELAQLGIF	3339.78	3	5.11	0.46	763.7	3	28/128	9.80E9
	133.37 - 134.50	-.AELDGKPVIVCSTGIGGPSTSIAVEELAQLGIF	3339.78	3	6.38	0.50	1407.6	1	36/128	7.85E9
	151.30 - 152.57	-.LDGASLHFAPLEFPAVADFECTTALVEAAK.-	3192.56	3	7.10	0.63	2100.8	1	39/116	7.63E9
	152.01	-.LDGASLHFAPLEFPAVADFECTTALVEAAK.-	3192.56	2	3.11	0.48	148.1	13	10/58	8.77E8
	153.08 - 154.06	-.LDGASLHFAPLEFPAVADFECTTALVEAAK.-	3192.56	3	6.33	0.55	1827.1	1	38/116	8.47E9
	154.72	-.LDGASLHFAPLEFPAVADFECTTALVEAAK.-	3192.56	3	4.21	0.56	1219.4	1	31/116	1.41E9
	88.38	-.NDLQGATLAIVPGDPDRVEK.-	2109.33	3	3.69	0.54	986.4	1	31/76	3.26E9
	88.24 - 88.84	-.NDLQGATLAIVPGDPDRVEK.-	2109.33	2	3.79	0.56	808.4	1	20/38	4.18E9
	93.87	-.NDLQGATLAIVPGDPDRVEK.-	2109.33	2	3.83	0.51	550.7	1	17/38	2.31E9
	82.61	-.SDVFHLGLTK.-	1117.28	1	1.85	0.33	451.2	1	10/18	1.58E9
	82.80	-.SDVFHLGLTK.-	1117.28	2	2.72	0.51	747.7	1	13/18	1.41E9
#20	FABI_ECOLI (P29132)					110.28			11 (11 0 0 0 0)	1.31
	80.22	-.EGAELAFTYQNDKLK.-	1727.90	2	2.60	0.37	688.2	1	14/28	2.82E9
	116.22	-.FDGFVHSIGFAPGDQLDGDYVNAVTR.-	2799.00	2	4.01	0.62	622.3	1	21/50	1.98E9
	115.36 - 116.57	-.FDGFVHSIGFAPGDQLDGDYVNAVTR.-	2799.00	3	4.13	0.31	567.0	4	25/100	5.86E9
	113.64 - 114.74	-.FDGFVHSIGFAPGDQLDGDYVNAVTR.-	2799.00	3	3.16	0.31	684.7	1	29/100	9.06E9
	78.32	-.IAHDISSYSFVAM*AK.-	1656.88	2	4.29	0.58	1232.0	1	19/28	3.21E9
	78.76 - 79.89	-.LSIAYGIAQAM*HR.-	1447.69	2	2.50	0.37	542.3	2	14/24	3.27E9
	129.40	-.SM*LNPGSALLTLSYLGAER.-	2010.30	3	3.92	0.46	1166.0	1	28/72	1.47E9
	129.13 - 130.37	-.SM*LNPGSALLTLSYLGAER.-	2010.30	2	5.61	0.65	1140.0	1	22/36	8.31E9
	137.48 - 138.02	-.SMLNPGSALLTLSYLGAER.-	1994.30	2	3.74	0.41	983.0	1	18/36	4.58E9
	29.98 - 31.27	-.VNAISAGPIR.-	998.16	2	3.12	0.56	911.1	1	15/18	6.26E8
	32.05	-.VNAISAGPIR.-	998.16	2	3.04	0.47	956.2	1	15/18	4.43E8
#21	CYSK_ECOLI (P11096					100.25			10 (10 0 0 0 0)	1.11
	82.46 - 83.07	-.ALGANLVLTEGAK.-	1257.46	2	3.41	0.37	1450.7	1	19/24	1.93E9
	111.90	-.GKTDLISVAVEPTDSPVIAQALAGEEIKPGPHI	3369.81	3	4.60	0.57	828.0	1	32/128	2.25E9
	113.88 - 114.57	-.GVLKPGVELVEPTSGNTGIALAYVAAAR.-	2755.16	3	5.01	0.54	1340.7	1	33/108	5.12E9
	114.23	-.GVLKPGVELVEPTSGNTGIALAYVAAAR.-	2755.16	2	3.99	0.60	355.1	1	19/54	1.74E9
	93.59 - 94.87	-.IFEDNSLTIGHTPLVR.-	1813.05	2	4.16	0.63	866.6	1	18/30	2.77E9
	117.05 - 117.64	-.IQGIGAGFIPANLDLK.-	1627.91	2	4.51	0.60	1576.4	1	23/30	7.00E9
	75.48 - 76.29	-.VIGITNEEAISTAR.-	1474.64	2	3.01	0.51	911.7	1	17/26	3.53E9
	101.90 - 102.47	-.YLLLQQFSNPANPEIHEK.-	2142.40	2	4.95	0.62	962.5	1	19/34	4.49E9
	101.98	-.YLLLQQFSNPANPEIHEK.-	2142.40	3	3.44	0.44	1219.5	1	29/68	3.50E9
	139.84 - 140.40	-.YLSTALFADLFTEK.-	1619.84	2	3.04	0.48	1052.4	1	17/26	2.75E9
#22	KPRS_ECOLI (P08330					100.24			10 (10 0 0 0 0)	1.07
	90.59 - 91.59	-.ANVSQVM*HIIGDVAGR.-	1683.92	2	4.14	0.63	939.4	1	18/30	3.71E9
	108.67	-.ITAVIPYFGYAR.-	1371.61	2	2.63	0.42	499.3	1	15/22	3.26E9
	74.14 - 75.31	-.LFAGNATPELAQR.-	1388.55	2	3.09	0.44	984.6	1	16/24	5.25E9
	65.26 - 65.85	-.LLNDTDM*AIIDKR.-	1534.76	2	2.97	0.54	404.9	2	12/24	1.99E9
	72.16	-.LYTSLGDAAVGR.-	1223.36	1	2.20	0.45	249.3	1	12/22	2.40E9
	72.06 - 72.62	-.LYTSLGDAAVGR.-	1223.36	2	3.56	0.57	1306.5	1	19/22	3.67E9
	121.19	-.NSVIDEVVVCDTIPLSDEIK.-	2246.49	2	4.37	0.57	674.1	1	18/38	4.95E9
	91.28	-.VFAYATHPIFSGNAANNLR.-	2064.29	2	3.90	0.60	308.1	1	15/36	2.43E9
	91.19	-.VFAYATHPIFSGNAANNLR.-	2064.29	3	4.72	0.57	1202.4	1	33/72	2.48E9
	103.14	-.VVADFLSSVGVDR.-	1364.53	2	4.57	0.64	2411.1	1	21/24	3.80E9
#23	TPIS_ECOLI (P04790)					90.32			9 (9 0 0 0 0)	1.13
	80.83	-.ADAFAVIVK.-	934.11	2	3.27	0.44	1258.6	1	15/16	1.50E9
	80.45 - 81.80	-.ADAFAVIVK.-	934.11	1	1.83	0.10	625.7	4	10/16	5.22E9
	85.44	-.DIGAQYIIIGHSER.-	1572.75	1	2.35	0.19	214.3	2	12/26	1.71E9
	83.38 - 85.36	-.DIGAQYIIIGHSER.-	1572.75	2	4.68	0.46	1749.5	1	20/26	5.00E9
	113.46	-.EAEGSHIM*LGAQNVDLNLSGAFTGETSAAM	3296.63	3	3.75	0.47	753.4	1	31/124	5.87E9
	111.92	-.EAEGSHIM*LGAQNVDLNLSGAFTGETSAAM	3296.63	2	3.38	0.55	214.2	1	15/62	1.65E9
	111.43 - 112.83	-.EAEGSHIM*LGAQNVDLNLSGAFTGETSAAM	3296.63	3	6.50	0.60	1019.2	1	37/124	8.54E9
	103.08 - 103.20	-.EQGLTPVLCIGETEAENEAGK.-	2246.41	2	3.63	0.60	539.4	1	17/40	4.12E9
	105.60	-.EQGLTPVLCIGETEAENEAGKTEEVCAR.-	3092.30	2	3.57	0.60	251.1	1	14/54	2.24E9
#24	ATPB_ECOLI (P00824					90.28			9 (9 0 0 0 0)	0.70
	105.68	-.AAPSYEELSNSQELLETGIK.-	2180.36	2	2.75	0.44	492.0	1	14/38	2.71E9
	123.98	-.DVLLFVDNIYR.-	1367.58	2	3.26	0.47	1152.3	1	16/20	1.96E9
	135.48	-.FLSQPFFVAEVFTGSPGK.-	1959.23	2	3.95	0.52	507.9	1	18/34	2.49E9
	126.26 - 127.53	-.GIM*EGEYDHLPEQAFYM*VGSIEEAVEK.-	3105.40	3	5.66	0.59	1343.1	1	31/104	3.86E9
	92.03	-.NIAIEHSGYSVFAGVGER.-	1907.08	2	4.88	0.63	1048.1	1	19/34	1.97E9
	111.23	-.QIASLGIYPAVDPLDSTSR.-	2004.23	2	3.42	0.63	400.2	1	16/36	3.28E9
	66.33 - 67.04	-.VALTGLTM*AEK.-	1150.37	2	2.83	0.47	1015.4	1	15/20	1.73E9
	139.73	-.YQELKDIIAILGM*DELSEEDKLVVAR.-	3008.43	3	3.53	0.43	731.8	2	28/100	1.93E9
	110.29	-.YTLAGTEVSALLGR.-	1451.65	2	3.92	0.44	1494.0	1	19/26	2.45E9
#25	METQ_ECO57 (Q8X8\					90.27			9 (9 0 0 0 0)	1.45
	135.36 - 136.50	-.DGVGLLPTVLDVVENPK.-	1766.03	2	3.51	0.54	324.1	1	16/32	2.79E9
	74.23 - 74.79	-.FVQAYQSDEVYEAANK.-	1862.97	2	4.73	0.60	2001.0	1	24/30	4.55E9
	90.48 - 91.00	-.IVELEAPQLPR.-	1265.48	2	3.51	0.50	1121.1	1	16/20	3.22E9
	126.32 - 127.57	-.LKDGVGLLPTVLDVVENPK.-	2007.36	2	3.15	0.41	507.5	1	17/36	5.16E9
	124.79 - 125.74	-.LKDGVGLLPTVLDVVENPK.-	2007.36	2	4.19	0.56	903.5	1	22/36	3.78E9
	125.68	-.LKDGVGLLPTVLDVVENPK.-	2007.36	3	4.29	0.52	2495.3	1	37/72	3.95E9

	106.97 - 107.53	-.LVAVGNTFVYPIAGYSK.-	1800.09	2	5.09	0.64	1322.5	1	20/32	9.08E9
	89.53 - 90.08	-.SLDELQDGSQVAVPNDPTNLGR.-	2326.46	2	4.06	0.48	505.9	1	21/42	8.42E9
	95.88 - 97.09	-.VGVIVGAEQQVAEVAQK.-	1725.97	2	5.37	0.68	1935.6	1	24/32	5.05E9
#26	PUR7_ECOLI (P21155					90.26			9 (9 0 0 0 0)	1.20
	83.78 - 84.36	-.GEVVLGDEFSPDGSR.-	1564.64	2	3.92	0.58	1489.3	1	19/28	4.32E9
	132.53	-.LFDDAGLILVDFK.-	1466.70	2	3.30	0.53	1433.1	1	17/24	1.68E9
	160.37 - 161.53	-.LGIEEGIELNPPLFDLFLK.-	2158.52	2	4.74	0.58	647.4	1	20/36	1.43E9
	162.26 - 163.72	-.LGIEEGIELNPPLFDLFLK.-	2158.52	2	3.14	0.48	229.0	1	12/36	1.59E9
	71.37 - 72.02	-.LLSDTECLVK.-	1178.35	2	2.67	0.35	547.7	1	15/18	3.18E9
	126.24 - 126.95	-.QSLGGLIEAYEAVAR.-	1577.76	2	3.93	0.61	1001.6	1	21/28	8.10E9
	148.87 - 149.71	-.RLGIEEGIELNPPLFDLFLK.-	2314.71	2	2.77	0.59	261.9	1	13/38	2.47E9
	119.87 - 121.31	-.TVYSTENPDLLVLEFR.-	1897.12	2	5.26	0.57	1464.8	1	23/30	1.11E10
	109.74 - 110.95	-.TVYSTENPDLLVLEFRNDTSAGDGAR.-	2842.03	3	3.05	0.37	437.1	1	27/100	4.25E9
#27	PGK_ECO57 (Q8XD03					90.26			9 (9 0 0 0 0)	0.95
	146.78	-.ADEQILDIGDASAQELAEILK.-	2243.45	2	5.08	0.64	1368.5	1	23/40	3.10E9
	136.64 - 137.45	-.FADVACAGPLLAAELDALGK.-	2003.28	2	5.20	0.67	1364.2	1	25/38	4.70E9
	114.42 - 115.42	-.IADQLIVGGGIANTFIAAQGHDVGK.-	2466.78	3	3.96	0.60	1006.2	1	35/96	3.58E9
	89.41 - 90.00	-.LLTTCNIPVPSDVR.-	1585.82	2	3.89	0.46	797.7	1	16/26	3.91E9
	76.10 - 77.17	-.SLYEADLVDEAKR.-	1509.64	2	3.75	0.63	1809.8	1	19/24	2.78E9
	145.40	-.TILWNGPVGVFEFPNFR.-	1994.28	2	3.48	0.58	1028.8	1	17/32	1.57E9
	73.25	-.VATEFSETAPATLK.-	1465.63	1	1.89	0.41	162.9	27	8/26	1.80E9
	73.22	-.VATEFSETAPATLK.-	1465.63	2	3.67	0.51	648.4	1	15/26	3.16E9
	125.11 - 126.09	-.VM*VTSHLGRPTEGEYNEEFSLLPVVNYLK.-	3339.76	3	4.30	0.55	839.2	1	29/112	5.67E9
#28	Q8XDF1 (Q8XDF1) Ou					80.25			8 (8 0 0 0 0)	0.69
	126.49	-.NM*STYVDYIINQIDSDNK.-	2150.31	2	4.96	0.60	1349.4	1	20/34	2.20E9
	147.93 - 148.95	-.NSNFFGLVDGLNFAVQYLGK.-	2204.47	2	3.01	0.48	186.8	18	11/38	2.83E9
	159.40 - 160.67	-.NSNFFGLVDGLNFAVQYLGK.-	2204.47	2	4.03	0.35	567.0	1	15/38	1.25E9
	72.14 - 73.48	-.TNLQEAQLLGNGK.-	1386.54	2	3.74	0.61	1265.2	1	18/24	4.58E9
	61.40 - 62.45	-.TNLQEAQLLGNGKK.-	1514.71	2	3.43	0.54	1367.5	1	17/26	4.62E9
	59.76 - 60.87	-.TNLQEAQLLGNGKK.-	1514.71	2	3.16	0.48	1727.0	1	20/26	1.50E9
	73.72 - 73.81	-.YADVGSFDYGR.-	1250.30	1	1.80	0.58	344.7	1	13/20	1.52E9
	87.55	-.YDANNIYLAANYGETR.-	1848.95	2	2.83	0.40	756.7	1	17/30	3.48E9
#29	OMPT_ECO57 (P5860					80.16			8 (8 0 0 0 0)	0.20
	92.05	-.GWLLNEPNYR.-	1262.40	2	2.90	0.14	988.3	1	14/18	2.55E9
	36.29 - 37.72	-.LGLM*AGYQESR.-	1241.40	2	2.94	0.46	1090.5	1	17/20	3.79E8
	38.60 - 39.83	-.LGLM*AGYQESR.-	1241.40	2	3.13	0.44	1259.6	1	18/20	4.15E8
	40.67 - 41.60	-.LGLM*AGYQESR.-	1241.40	2	3.02	0.49	1173.5	1	17/20	3.76E8
	42.88 - 43.62	-.LGLM*AGYQESR.-	1241.40	2	2.92	0.47	856.1	1	15/20	3.63E8
	34.27 - 35.69	-.LGLM*AGYQESR.-	1241.40	2	2.92	0.40	689.5	1	14/20	4.11E8
	32.63 - 33.48	-.LGLM*AGYQESR.-	1241.40	2	2.94	0.40	608.4	1	12/20	4.03E8
	92.95	-.YEDFELGGTFK.-	1306.40	2	3.22	0.50	1198.1	1	17/20	1.59E9
#30	DAPA_ECO57 (P6394					70.29			7 (7 0 0 0 0)	0.49
	99.78	-.AIAEHTDLPQILYNVPSR.-	2038.29	2	3.73	0.51	859.0	1	18/34	3.43E9
	99.83	-.AIAEHTDLPQILYNVPSR.-	2038.29	3	3.38	0.35	969.3	1	23/68	1.97E9
	105.09	-.IPVIAGTGANATAEAISLTQR.-	2055.32	2	5.85	0.63	1342.6	1	22/40	2.55E9
	103.37	-.IPVIAGTGANATAEAISLTQR.-	2055.32	2	5.29	0.59	2339.0	1	27/40	2.97E9
	105.15	-.IPVIAGTGANATAEAISLTQR.-	2055.32	3	3.55	0.50	1312.7	1	32/80	1.80E9
	88.36	-.LFVEPNPIPVK.-	1253.52	2	2.94	0.32	731.8	1	14/20	2.07E9
	51.12 - 52.51	-.LPM*TPITDSGR.-	1204.38	2	2.54	0.33	515.8	4	13/20	7.29E8
#31	PTND_ECOLI (P08188					70.28			7 (7 0 0 0 0)	0.66
	68.29	-.ANGAEIDDGAINGIK.-	1458.56	1	2.29	0.40	657.3	1	17/28	1.12E9
	68.00 - 68.62	-.ANGAEIDDGAINGIK.-	1458.56	2	3.65	0.48	1482.2	1	19/28	2.28E9
	155.23 - 156.69	-.HLEFFNTQPFVAAPILGVTLALEEQR.-	2942.36	3	5.56	0.63	2377.3	1	40/100	2.42E9
	147.57 - 148.77	-.LTEGASILGLFVM*GALVNK.-	1950.33	2	4.95	0.39	604.0	3	17/36	3.45E9
	110.02 - 110.86	-.M*QALGFCFSM*VPAIR.-	1761.10	2	3.73	0.55	1190.2	1	18/28	5.36E9
	106.46	-.SNLFQGSWNFER.-	1485.59	2	3.09	0.52	960.9	1	16/22	4.34E9
	94.10	-.WTHVNIPLVVSR.-	1421.67	2	2.81	0.57	862.5	1	16/22	1.99E9
#32	Q8X773 (Q8X773) Hyp					60.29			6 (6 0 0 0 0)	0.55
	128.92 - 129.76	-.DQAVLIEPFDTVTVQGFYR.-	2199.45	2	3.64	0.45	603.3	1	17/36	3.93E9
	71.71	-.FSGNYGNM*TEVSYQVAK.-	1912.07	2	3.23	0.43	333.1	2	13/32	1.85E9
	67.70 - 68.83	-.IVQSPDVIPADSEAGR.-	1654.80	2	3.19	0.46	1076.2	1	19/30	2.63E9
	74.27 - 75.27	-.KVEIPGVATTASPSSEVGR.-	1886.10	2	4.79	0.57	538.7	1	20/36	3.08E9
	133.00 - 133.74	-.VINGVVELPKDQAVLIEPFDTVTVQGFYR.-	3248.72	3	5.84	0.43	1788.3	1	39/112	4.01E9
	87.86	-.YTVTLPDGTKVEELNK.-	1808.02	2	2.74	0.55	307.3	1	15/30	2.07E9
#33	FKBA_ECO57 (P65765					60.28			6 (6 0 0 0 0)	0.66
	12.33	-.DSDTVVVNYK.-	1140.23	1	2.30	0.27	419.4	1	13/18	1.50E9
	76.66	-.GTLIDGKEFDNSYTR.-	1716.83	2	2.92	0.27	1078.7	1	18/28	3.62E9
	107.99	-.LDKDQLIAGVQDAFADK.-	1848.05	2	5.66	0.67	1685.2	1	23/32	4.97E9
	126.00 - 126.62	-.LSDQEIEQTLQAFEAR.-	1879.02	2	5.30	0.39	1874.0	1	20/30	5.72E9
	126.13	-.LSDQEIEQTLQAFEAR.-	1879.02	3	4.40	0.49	2446.7	1	33/60	1.19E9
	93.80	-.TSSTGLVYQVVEAGKGEAPK.-	2022.25	2	4.26	0.67	677.9	1	18/38	3.82E9
#34	EFG_ECOLI (P02996)					60.28			6 (6 0 0 0 0)	0.60
	101.55	-.DVTTGDTLCDPDAPIILER.-	2102.28	2	4.50	0.49	939.6	1	19/36	2.12E9
	76.52	-.EFNVEANVGKPQVAYR.-	1822.01	2	4.38	0.57	1765.2	1	22/30	2.20E9

	97.75 - 98.78	-.IHAEVPLSEM*FGYATQLR.-	2079.37	2	4.10	0.66	782.2	1	22/34	2.25E9
	150.93 - 152.31	-.LGANPVPLQLAIGAEEHFTGVVDLVK.-	2689.10	3	5.66	0.58	558.6	1	32/100	4.02E9
	95.02 - 95.59	-.VEVETPEENTGDVIGDLSR.-	2060.16	2	3.94	0.60	560.5	1	19/36	5.18E9
	94.37	-.YDEAPSNVAQAVIEAR.-	1733.86	2	4.80	0.61	1169.0	1	21/30	3.41E9
#35	Q7BSW5 (Q7BSW5) P					60.24			6 (6 0 0 0 0)	0.49
	123.78 - 124.29	-.AGLGYQFDLLANGETVLR.-	1938.17	2	4.81	0.52	1019.1	1	20/34	4.10E9
	52.80 - 53.38	-.DKDYNPLIGR.-	1191.32	2	2.72	0.40	890.2	1	15/18	7.28E8
	50.86 - 52.13	-.DKDYNPLIGR.-	1191.32	2	2.69	0.48	1216.0	1	16/18	5.91E8
	111.56	-.YHVTEDAWIEPQAELVYGSVSGK.-	2579.80	3	3.16	0.53	1126.8	1	28/88	2.85E9
	77.72	-.YVHHDNEYTATFAGLGTR.-	2053.18	2	2.74	0.52	551.4	1	17/34	3.17E9
	75.73 - 77.04	-.YVHHDNEYTATFAGLGTR.-	2053.18	2	4.55	0.56	1173.8	1	19/34	4.20E9
#36	ENTB_ECOLI (P15048					60.21			6 (6 0 0 0 0)	0.63
	71.87 - 73.02	-.LQAYALPESHDIPQNK.-	1825.02	2	4.29	0.52	975.6	1	21/30	4.12E9
	97.51	-.NPTIDAWWK.-	1131.27	2	2.65	0.43	619.8	1	12/16	1.35E9
	97.45 - 97.49	-.NPTIDAWWK.-	1131.27	1	1.98	0.35	397.6	2	12/16	2.89E9
	83.70	-.VHGDIDFVM*LAK.-	1361.59	2	2.94	0.44	558.6	1	15/22	2.76E9
	92.09 - 92.28	-.VVM*TEELLPAPIPASK.-	1712.05	1	1.89	0.35	45.4	15	10/30	1.83E9
	91.80 - 92.78	-.VVM*TEELLPAPIPASK.-	1712.05	2	4.16	0.58	818.5	1	20/30	7.11E9
#37	KAD_ECOLI (P05082)					60.21			6 (6 0 0 0 0)	0.73
	71.14 - 71.67	-.FNPPKVEGKDDVTGEELTTR.-	2233.42	2	4.12	0.57	528.7	1	20/38	2.42E9
	77.71	-.IILLGAPGAGK.-	1010.26	2	2.90	0.19	1005.1	1	17/20	1.55E9
	85.71	-.LVEYHQM*TAPLIGYYSK.-	2030.33	3	3.36	0.40	909.5	1	29/64	3.87E9
	97.71 - 98.74	-.LVEYHQMTAPLIGYYSK.-	2014.33	2	2.75	0.01	192.1	3	14/32	6.59E9
	123.15 - 123.72	-.LVTDELVIALVK.-	1313.61	2	4.26	0.53	1570.8	1	20/22	3.69E9
	87.44 - 88.01	-.YGIPQISTGDM*LR.-	1467.67	2	2.81	0.54	834.5	1	19/24	4.91E9
#38	SUCD_ECOLI (P0745					60.18			6 (6 0 0 0 0)	0.51
	75.89 - 76.46	-.EHVTKPVVGYIAGVTAPK.-	1867.18	2	3.66	0.57	473.5	1	20/34	3.45E9
	69.67 - 70.22	-.FAALEAAGVK.-	977.14	1	2.47	0.19	950.7	1	13/18	1.72E9
	93.89 - 94.26	-.GGTTHLGLPVFNTVR.-	1569.79	2	3.44	0.47	1123.4	1	19/28	4.98E9
	133.77 - 134.16	-.LIITITEGIPTLDM*LTVK.-	1988.42	2	2.67	0.44	193.2	1	11/34	3.29E9
	68.27 - 68.87	-.M*IGPNCPGVITPGECK.-	1746.99	2	2.93	0.45	849.3	1	16/30	1.69E9
	82.65	-.VICQGFTGSQGTFHSEQAIAYGTK.-	2588.81	2	3.46	0.63	286.7	4	14/46	1.20E9
#39	Q8X5N7 (Q8X5N7) Pu					50.37			5 (5 0 0 0 0)	0.44
	144.38 - 144.95	-.QLSSEGILSLRPDSVITWQDAGPQIVLDQLR.-	3436.86	3	7.49	0.59	1629.8	1	38/120	3.21E9
	68.23 - 68.37	-.SIAGITHTAAWK.-	1256.44	2	3.04	0.51	965.7	1	16/22	1.23E9
	105.44	-.TLQVPEQGEALVTQISQR.-	1998.23	2	4.48	0.58	603.0	1	18/34	5.30E9
	76.08 - 76.67	-.VPATLQQM*YANIR.-	1521.77	2	3.02	0.58	1146.5	1	16/24	2.37E9
	61.17 - 62.55	-.VVGVDETTSYPPETAK.-	1693.83	2	2.80	0.63	650.4	1	16/30	1.79E9
#40	CH60_ECOLI (P06139					50.29			5 (5 0 0 0 0)	0.41
	68.43 - 69.65	-.AIAQVGTISANSDETVGK.-	1761.91	2	4.65	0.67	1010.2	1	21/34	2.63E9
	94.72	-.DTTTIIDGVGEEAAIQGR.-	1846.97	2	2.57	0.28	464.9	1	14/34	2.53E9
	140.26	-.GYLSPYFINKPETGAVELESPFILLADK.-	3113.55	3	5.87	0.61	1420.5	1	36/108	2.00E9
	131.91 - 132.42	-.GYLSPYFINKPETGAVELESPFILLADKK.-	3241.72	3	5.41	0.53	1564.7	1	37/112	3.91E9
	88.28	-.QIVLNCGEEPSVVANTVK.-	1958.20	2	4.73	0.66	1307.2	1	22/34	1.96E9
#41	Q8X8I7 (Q8X8I7) Malo					50.28			5 (5 0 0 0 0)	0.52
	93.49	-.ACEEAAEGQVVSPVNFNSPGQVVIAGHK.-	2896.16	2	3.16	0.54	256.9	1	15/54	1.66E9
	93.43 - 94.53	-.ACEEAAEGQVVSPVNFNSPGQVVIAGHK.-	2896.16	3	5.67	0.56	1047.0	1	31/108	3.98E9
	101.53 - 102.15	-.ITFNAPTVPVVNNVDVK.-	1828.10	2	3.45	0.54	244.7	1	13/32	4.20E9
	121.78 - 122.34	-.IVDTLTASALNEPSAM*AAALEL.-	2218.51	2	3.65	0.59	607.4	1	14/42	3.94E9
	94.22	-.SVEYM*AAQGVEHLYEVGPGK.-	2181.41	2	3.75	0.55	685.3	1	16/38	2.74E9
#42	Q8XC86 (Q8XC86) Se					50.25			5 (5 0 0 0 0)	0.41
	94.12 - 94.95	-.ASDFAEDLADAAEK.-	1453.49	2	3.08	0.48	1165.9	1	17/26	3.84E9
	75.77	-.ATESVADVAEEASSAM*QK.-	1840.95	2	2.80	0.49	956.2	1	17/34	2.65E9
	53.82 - 55.02	-.DLLEVQNR.-	987.09	1	1.86	0.24	228.0	2	9/14	6.24E8
	91.38 - 91.69	-.QLAQSYQIQQAVFESQNK.-	2111.30	2	4.94	0.62	904.8	1	18/34	1.96E9
	93.10 - 93.66	-.SLENFQQGNLELYK.-	1683.84	2	4.91	0.47	1787.4	1	21/26	4.08E9
#43	ADHE_ECOLI (P17547					50.25			5 (5 0 0 0 0)	0.46
	128.71	-.EAGVQEADFLANVDKLSEDAFDDQCTGANP	3384.52	3	4.93	0.57	581.6	1	27/120	3.34E9
	62.12 - 62.32	-.FATHGGYLLQGK.-	1292.47	2	3.22	0.32	1231.9	1	19/22	1.51E9
	111.33 - 111.84	-.ILINTPASQGGIGDLYNFK.-	2022.29	2	4.70	0.70	758.1	1	21/36	5.02E9
	61.11	-.LSEDAFDDQCTGANPR.-	1796.82	2	3.32	0.51	851.9	1	15/30	6.96E8
	82.86 - 83.43	-.YAEIADHLGLSAPGDR.-	1685.82	2	3.95	0.55	1414.1	1	18/30	4.05E9
#44	ATPG_ECOLI (P00837					50.23			5 (5 0 0 0 0)	0.43
	64.87 - 65.46	-.ELQLVYNK.-	1007.17	1	2.27	0.18	369.9	1	10/14	2.77E9
	116.74 - 117.33	-.FINTM*SQVPTISQLLPLPASDDDDLKHK.-	3141.54	3	4.67	0.57	751.0	1	30/108	5.36E9
	110.44	-.GLCGGLNINLFK.-	1306.53	2	3.15	0.42	960.0	1	16/22	1.79E9
	98.43	-.RYVESQVYQGVVENLASEQAAR.-	2497.71	2	4.20	0.59	498.9	1	17/42	1.81E9
	62.01 - 62.63	-.VM*LQAYDEGRLDK.-	1554.75	2	2.94	0.37	399.6	1	14/24	1.83E9
#45	YBIS_ECOLI (P75789)					50.21			5 (5 0 0 0 0)	0.40
	125.50	-.AAGEPLPAVVPAGPDNPM*GLYALYIGR.-	2727.13	2	3.32	0.49	418.0	1	19/52	2.49E9
	123.74	-.GGTVLNIPQQLILPDTVHEGIVINSAEM*R.-	3132.58	3	3.60	0.47	620.0	1	34/112	1.80E9
	103.71	-.SVQTVTGQPDVDQVVLDEAIK.-	2242.47	2	3.62	0.47	357.4	1	17/40	3.03E9
	72.49	-.VQFIDEPVK.-	1075.24	1	2.17	0.27	784.3	2	11/16	1.67E9
	121.54	-.YIEVHNPLSTTEAQFEGQEIVPITLTK.-	3059.42	3	4.15	0.51	923.0	1	32/104	3.80E9

#46	ALF_ECOLI (P11604) I					50.21			5 (5 0 0 0 0)	0.46
	66.16	-.ANEAYLQGQLGNPK.-	1503.64	1	1.92	0.26	396.8	3	13/26	1.06E9
	115.99 - 116.62	-.FTIAASFGNVHGVYKPGNVVLTPTILR.-	2873.34	3	4.11	0.37	1355.3	1	31/104	3.89E9
	100.40 - 101.17	-.IFDFVKPGVITGDDVQK.-	1879.15	2	3.89	0.53	592.7	1	19/32	3.16E9
	105.46	-.VKAPVIVQFSNGGASFIAGK.-	1991.32	2	3.13	0.55	536.0	1	16/38	2.43E9
	102.05 - 103.18	-.VKAPVIVQFSNGGASFIAGK.-	1991.32	2	3.85	0.65	897.7	1	19/38	4.13E9
#47	Q8XDU6 (Q8XDU6) Hy					40.30			4 (4 0 0 0 0)	0.40
	125.83	-.KIFALPVIEQISPVLSR.-	1911.32	2	2.81	0.55	324.6	1	13/32	2.03E9
	95.33	-.KLDELDLIVVDHPQVK.-	1862.16	3	4.92	0.53	3493.8	1	37/60	2.25E9
	88.42	-.RKLDELDLIVVDHPQVK.-	2018.35	3	3.08	0.14	1128.6	5	24/64	3.05E9
	90.54 - 91.11	-.VYLNPQDCSVINDEALNR.-	2121.29	2	5.94	0.57	1303.1	1	22/34	5.27E9
#48	Q8X5V4 (Q8X5V4) Qui					40.25			4 (4 0 0 0 0)	0.15
	158.65 - 159.65	-.AAILPAAISFEQAAASFLK.-	1920.24	2	4.92	0.64	1726.4	1	23/36	1.14E9
	158.99	-.AAILPAAISFEQAAASFLK.-	1920.24	3	5.07	0.62	1871.2	1	35/72	3.05E8
	151.39	-.EELTEASNELFSLIASGVIK.-	2151.40	2	3.93	0.48	628.9	1	14/38	1.45E9
	90.69	-.GSLYVTRPSLQGYITTR.-	1913.17	2	2.66	0.40	267.4	1	13/32	1.89E9
#49	Q8X8I5 (Q8X8I5) 3-oxc					40.24			4 (4 0 0 0 0)	0.36
	122.12	-.GLM*LNVTDPASIESVLEK.-	1933.21	2	4.46	0.59	1088.7	1	20/34	4.34E9
	92.51	-.IITIGSVVGTM*GNGGQANYAAAK.-	2210.50	2	4.79	0.57	756.7	1	22/44	2.59E9
	101.08	-.VIGTATSENGAQAISDYLGANGK.-	2238.40	2	4.10	0.48	1312.2	1	22/44	2.64E9
	99.06	-.VIGTATSENGAQAISDYLGANGK.-	2238.40	2	4.86	0.50	1693.1	1	25/44	1.81E9
#50	GRPE_ECO57 (Q7ABI					40.24			4 (4 0 0 0 0)	0.37
	94.60 - 95.16	-.ANPDM*SAM*VEGIELTLK.-	1852.12	2	4.74	0.64	398.6	1	18/32	3.55E9
	136.27 - 137.26	-.FINELLPVIDSLDR.-	1644.89	2	4.04	0.42	717.4	1	18/26	4.35E9
	85.75	-.IANLEAQLAEAQTR.-	1528.69	1	2.07	0.45	137.7	31	9/26	1.23E9
	85.48	-.IANLEAQLAEAQTR.-	1528.69	2	3.68	0.58	1994.7	1	20/26	2.58E9
#51	YGGB_ECOLI (P11666					40.23			4 (4 0 0 0 0)	0.32
	116.03	-.EFDAAGISFPYPQM*DVNFK.-	2193.42	2	3.18	0.50	307.8	2	14/36	3.01E9
	95.84	-.IIAGNIINFSR.-	1218.43	2	3.76	0.53	1164.2	1	17/20	1.65E9
	130.47 - 131.15	-.VWSNSGDLQNVYWDVLER.-	2181.35	2	2.93	0.42	713.8	1	17/34	3.18E9
	125.56	-.VWSNSGDLQNVYWDVLER.-	2181.35	2	4.54	0.60	992.4	1	19/34	2.44E9
#52	O82921 (O82921) Hyp					40.21			4 (4 0 0 0 0)	0.21
	50.15 - 50.96	-.ALYDAVASGTK.-	1096.22	2	2.89	0.36	1284.3	1	17/20	4.70E8
	49.41 - 50.06	-.ALYDAVASGTK.-	1096.22	1	2.31	0.38	592.4	1	15/20	7.05E8
	105.00	-.IGLWGTSLGGGHVFSAAAQDQR.-	2229.44	3	3.49	0.58	1173.0	1	33/84	2.39E9
	104.96	-.IGLWGTSLGGGHVFSAAAQDQR.-	2229.44	2	4.23	0.64	1445.8	1	22/42	3.10E9
#53	RL3_ECO57 (P60440)					40.20			4 (4 0 0 0 0)	0.59
	73.20 - 73.79	-.GAVPGATGSDLIVKPAVK.-	1680.97	2	4.03	0.65	730.0	1	21/34	4.29E9
	76.83	-.GAVPGATGSDLIVKPAVKA.-	1752.05	2	3.55	0.56	550.7	1	17/36	3.01E9
	123.03 - 124.43	-.IFTEDGVSIPVTVIEVEANR.-	2189.45	2	3.72	0.60	386.7	1	18/38	8.78E9
	76.60	-.VTVQSLDVVR.-	1116.29	2	3.47	0.46	1179.4	1	17/18	2.81E9
#54	MIND_ECOLI (P18197)					40.19			4 (4 0 0 0 0)	0.32
	89.77	-.ASNQGEPVILDINADAGK.-	1812.96	2	3.48	0.49	493.7	1	16/34	4.28E9
	84.21	-.TENLYILPASQTR.-	1506.69	2	3.19	0.52	701.3	1	16/24	3.83E9
	51.75 - 53.04	-.TTSSAAIATGLAQK.-	1320.48	2	3.83	0.39	2332.0	1	22/26	1.10E9
	49.92 - 51.18	-.TTSSAAIATGLAQK.-	1320.48	2	3.52	0.40	1816.6	1	20/26	1.02E9
#55	HISJ_ECOLI (P39182)					40.19			4 (4 0 0 0 0)	0.44
	114.26 - 115.45	-.IDAAFQDEVAASEGFLK.-	1811.97	2	3.78	0.55	953.2	1	17/32	5.82E9
	140.36 - 141.55	-.INTQCTFVENPLDALIPSLK.-	2274.59	2	3.78	0.55	316.9	1	15/38	4.01E9
	82.75 - 82.78	-.LFGVGTGM*GLR.-	1124.34	2	2.66	0.44	489.1	1	14/20	2.45E9
	64.64 - 65.22	-.NSDIQPTVESLK.-	1331.45	2	2.84	0.35	816.9	1	16/22	1.60E9
#56	RS1_ECOLI (P02349)					40.18			4 (4 0 0 0 0)	0.26
	99.08	-.AFLPGSLVDVRPVR.-	1526.81	2	2.61	0.36	336.8	1	13/26	2.39E9
	121.48	-.GVVVAIDKDVVLVDAGLKSESAIPAEQFK.-	2999.45	3	3.34	0.30	714.9	5	29/112	1.85E9
	93.03	-.KGDEIAAVVLQVDAERER.-	1999.22	3	3.55	0.42	654.3	1	27/68	1.98E9
	145.74	-.M*TESFAQLFEESLKEIETRPGSIVR.-	2915.27	3	3.13	0.51	893.6	1	28/96	2.00E9
#57	Q8X5N8 (Q8X5N8) Put					30.31			3 (3 0 0 0 0)	0.28
	101.63	-.EILAALESVGETK.-	1360.54	2	3.33	0.50	2305.7	1	20/24	2.50E9
	126.90 - 128.12	-.KPTSDGYVTSLELFAHDGTQIAQLYGQR.-	3097.38	3	3.61	0.43	1050.8	1	31/108	2.70E9
	109.70 - 109.87	-.VYATDNTDM*AAWSELLAR.-	2044.23	2	6.24	0.65	1443.5	1	21/34	3.67E9
#58	ACCA_ECOLI (P30867					30.31			3 (3 0 0 0 0)	0.27
	142.08 - 143.22	-.AQLLADLADLDVLSTEDLK.-	2044.29	2	4.59	0.54	1160.9	1	19/36	3.51E9
	66.06	-.AYADDKAIVGGIAR.-	1420.60	2	3.02	0.56	957.9	1	15/26	1.63E9
	139.57 - 139.65	-.SLNFLDFEQPIAELEAK.-	1965.19	2	6.17	0.48	1671.7	1	21/32	3.46E9
#59	PYRH_ECOLI (P29464					30.26			3 (3 0 0 0 0)	0.33
	114.44 - 115.76	-.LSGEALQGTEGFGIDASILDR.-	2150.33	2	4.79	0.63	1077.2	1	19/40	4.84E9
	110.17 - 110.76	-.VDGVFTADPAKDPTATM*YEQLTYSEVLEK.-	3236.55	3	5.15	0.63	1898.4	1	37/112	3.39E9
	95.44	-.VM*DLAAFTLAR.-	1224.46	2	3.16	0.40	1704.2	1	17/20	2.26E9
#60	Q8XBL3 (Q8XBL3) PEI					30.24			3 (3 0 0 0 0)	0.12
	62.86	-.EIEIYKQELRDEGK.-	1750.93	2	3.05	0.46	366.0	1	11/26	1.16E9
	162.88 - 163.99	-.IIDLSAIQDEVILVAADLTPSETAQLNLKK.-	3223.70	3	4.72	0.45	850.6	1	34/116	1.29E9
	62.34 - 62.90	-.ISADQVDQEVER.-	1389.45	2	3.33	0.33	1492.2	1	19/22	1.22E9
#61	KDSA_ECO57 (Q8XDE					30.23			3 (3 0 0 0 0)	0.29
	143.57 - 144.17	-.IITDVHEPSQAQPVADVVDVIQLPAFLAR.-	3143.58	3	4.65	0.59	1131.2	1	38/112	6.33E9

	158.47	-.VVSIGDINVANDLPFVLFGGM*NVLESR.-	2893.31	2	3.37	0.49	307.2	1	16/52	7.16E8
	157.14 - 158.34	-.VVSIGDINVANDLPFVLFGGM*NVLESR.-	2893.31	3	4.53	0.54	996.8	1	30/104	2.18E9
#62	HIS1_ECO57 (Q8X8T4					30.23			3 (3 0 0 0 0)	0.33
	149.65	-.AGLADAICDLVSTGATLEANGLR.-	2289.52	2	4.56	0.55	1361.1	1	23/44	1.41E9
	123.62 - 124.17	-.LDEVIALLPGAERPTILPLAGDQQR.-	2687.09	3	3.87	0.36	925.7	1	36/96	4.84E9
	94.74 - 95.37	-.LIAM*AENM*PIDILR.-	1632.97	2	4.46	0.49	1023.3	1	17/26	4.13E9
#63	Q8XDC4 (Q8XDC4) Fc					30.23			3 (3 0 0 0 0)	0.16
	115.82	-.FDIPFELVSHEGLSR.-	1746.94	3	3.22	0.55	920.3	1	30/56	1.16E9
	97.82	-.M*ADAIDAYQPDYVVLAK.-	1900.14	2	4.16	0.58	950.5	1	19/32	2.85E9
	151.99 - 152.13	-.TELEGIFNDSTLLADXDSALPEGSVR.-	2763.99	2	4.56	0.64	893.7	1	20/50	1.14E9
#64	TRXB_ECOLI (P09625					30.22			3 (3 0 0 0 0)	0.40
	111.58 - 112.65	-.LLILGSGPAGYTAAVYAAR.-	1865.17	2	2.51	0.26	315.9	2	13/36	8.36E9
	66.87	-.TLEEVTGDQM*GVTGVR.-	1708.87	2	4.47	0.51	1319.7	1	21/30	1.68E9
	94.62 - 95.39	-.VQSGIHGNATQTSIPGVFAAGDVM*DHIYR.-	3059.36	3	3.49	0.33	691.8	2	29/112	2.73E9
#65	Q8X881 (Q8X881) Hyp					30.20			3 (3 0 0 0 0)	0.24
	69.42 - 69.96	-.LGTDIGVVGPDAGGQK.-	1484.64	2	3.98	0.54	959.6	1	19/30	3.73E9
	69.57	-.LGTDIGVVGPDAGGQK.-	1484.64	1	2.24	0.46	660.1	1	16/30	1.07E9
	56.00 - 57.27	-.YHAWDDQIENR.-	1447.49	2	2.90	0.45	738.4	1	14/20	2.77E9
#66	Q8XBC5 (Q8XBC5) Pu					30.20			3 (3 0 0 0 0)	0.26
	116.47	-.KYDFSTPYTISGIQALVK.-	2032.33	2	3.94	0.61	1019.7	1	22/34	2.78E9
	86.08 - 87.27	-.RIDVVINQVTISDER.-	1757.97	2	3.95	0.54	833.7	1	19/28	2.87E9
	111.88	-.VGVGLGTNYEEWLR.-	1593.77	2	3.79	0.66	607.3	1	15/26	2.63E9
#67	Q8X528 (Q8X528) 2,3-					30.19			3 (3 0 0 0 0)	0.17
	66.91	-.GGAIVTVASDAAHTPR.-	1523.68	3	3.08	0.48	799.3	1	27/60	9.47E8
	66.62 - 67.22	-.GGAIVTVASDAAHTPR.-	1523.68	2	3.86	0.59	1395.0	1	21/30	2.69E9
	121.69	-.GIGYATALAFVEAGAK.-	1539.76	2	3.88	0.57	1286.7	1	20/30	1.85E9
#68	PROQ_ECO57 (Q8XC					30.19			3 (3 0 0 0 0)	0.17
	102.21	-.EEQHTPVSDISALTVGQALK.-	2124.34	2	3.49	0.54	447.6	1	18/38	2.69E9
	109.49	-.IGIFQDLVDR.-	1176.35	2	3.87	0.35	1321.1	1	17/18	1.80E9
	68.46	-.VDLDGNPCGELDEQHVEHAR.-	2291.37	2	3.32	0.48	692.6	1	16/38	9.18E8
#69	GPDA_ECOLI (P37606					30.19			3 (3 0 0 0 0)	0.22
	120.16	-.EALGDQIPLAVISGPTFAK.-	1928.22	2	3.74	0.67	504.7	1	18/36	3.46E9
	157.28	-.ELAAGLPTAISLASTDQTFADDLQQLLHCGK.	3286.63	3	3.64	0.43	312.5	1	29/120	1.45E9
	146.13	-.FGVEM*PITEEIYQVLYCGK.-	2293.62	2	2.99	0.59	180.5	3	11/36	2.15E9
#70	DEOD_ECOLI (P09743					30.19			3 (3 0 0 0 0)	0.19
	96.17	-.FKDHDFAAIADFDM*VR.-	1915.12	2	3.72	0.70	1162.9	1	17/30	1.84E9
	106.29	-.IALESVLLGDKE.-	1287.49	2	3.56	0.49	1673.0	1	17/22	2.51E9
	97.63	-.YIAETFLEDAR.-	1328.45	2	3.10	0.47	1098.4	1	17/20	1.79E9
#71	Q8XB03 (Q8XB03) Hig					30.18			3 (3 0 0 0 0)	0.15
	88.88	-.LISADGKPVLPTEENFANAAK.-	2186.45	2	3.01	0.52	362.2	1	16/40	1.56E9
	61.52	-.LPSQNIAVVR.-	1097.29	2	2.75	0.44	1379.6	1	17/18	9.94E8
	95.41	-.QIIANTVDFGASDAPLSDEK.-	2092.25	2	3.59	0.57	624.0	1	17/38	2.32E9
#72	Q8X9I0 (Q8X9I0) Sigm					30.16			3 (3 0 0 0 0)	0.17
	125.20	-.GEIRPLAQADAAELDALIVPGGFGAAK.-	2652.00	2	2.87	0.35	438.7	1	18/52	3.11E9
	138.44 - 139.76	-.IVTTPAYM*LAQNIAEAASGIDK.-	2294.61	2	3.25	0.59	157.5	43	10/42	2.26E9
	21.45 - 24.47	-.NVLIEAAR.-	886.03	1	1.97	0.35	138.7	1	9/14	1.89E8
#73	PYRB_ECOLI (P00479					30.16			3 (3 0 0 0 0)	0.14
	85.96 - 87.06	-.LGASVVGFSDSANTSLGK.-	1710.87	2	3.11	0.51	561.2	1	18/34	3.84E9
	18.75 - 20.39	-.TVHSLTQALAK.-	1169.36	2	2.75	0.48	574.6	1	15/20	2.65E8
	16.81 - 18.07	-.TVHSLTQALAK.-	1169.36	2	2.65	0.47	774.0	1	14/20	3.06E8
#74	EAE_ECO57 (P43261)					20.29			2 (2 0 0 0 0)	0.20
	122.25	-.GELPNIWLQYGQFK.-	1693.93	2	2.69	0.64	738.7	1	15/26	2.72E9
	133.87	-.LPFEYSALPLLGSAPLVAAGGVAGHTNK.-	2752.16	3	5.81	0.61	2012.6	1	37/108	3.58E9
#75	YEEZ_ECOLI (P76370					20.28			2 (2 0 0 0 0)	0.09
	158.16 - 158.73	-.SGPGDEFYLQAVQELVDSALAHR.-	2503.71	3	5.63	0.63	1630.9	1	37/88	1.23E9
	137.75 - 138.08	-.VAIVGLGWLGM*PLAM*SLSAR.-	2075.53	2	4.23	0.68	383.5	1	14/38	1.51E9
#76	ARCA_ECOLI (P03026					20.26			2 (2 0 0 0 0)	0.19
	93.24	-.HFESTPDTPEIIATIHGEGYR.-	2371.55	3	5.17	0.60	1615.5	1	32/80	2.29E9
	116.25	-.ILGLEIGADDYITKPFNPR.-	2133.43	2	4.42	0.58	1032.6	1	19/36	3.81E9
#77	ACCD_ECOLI (P08193					20.23			2 (2 0 0 0 0)	0.16
	126.72 - 127.44	-.LHSLLDEGSLVELGSELEPK.-	2166.41	2	4.62	0.06	1597.3	1	24/38	3.61E9
	65.34 - 65.93	-.LM*NLPAPNPEAPR.-	1436.66	2	3.06	0.48	848.6	1	19/24	1.63E9
#78	PSPA_ECOLI (P23853					20.23			2 (2 0 0 0 0)	0.18
	121.71	-.SLDDQFAELKADDAISEQLAQLK.-	2549.77	2	2.95	0.51	342.1	1	13/44	2.36E9
	96.86	-.SLEHEVTLVDDTLAR.-	1698.86	2	4.55	0.61	1265.2	1	18/28	3.29E9
#79	Q8X722 (Q8X722) Isoc					20.23			2 (2 0 0 0 0)	0.12
	82.63	-.AAIEYAIANDRDSVTLVHK.-	2087.32	2	4.54	0.64	684.1	1	18/36	1.32E9
	139.01 - 139.55	-.STQVYGQDVWLPAETLDLIR.-	2305.57	2	2.65	0.49	481.7	1	14/38	2.44E9
#80	DLHH_ECO57 (Q8X8L					20.23			2 (2 0 0 0 0)	0.14
	146.30 - 146.94	-.EGDPNDFADIPTLLSGLVAK.-	2073.29	2	3.89	0.56	293.6	1	13/38	2.49E9
	124.69	-.VPDSQVLADLDHVASWASR.-	2067.25	3	4.52	0.54	1591.7	1	32/72	2.00E9
#81	DNAK_ECOLI (P04475					20.22			2 (2 0 0 0 0)	0.13
	100.47 - 100.51	-.IINEPTAAALAYGLDK.-	1660.89	2	3.67	0.51	704.6	1	19/30	2.59E9
	95.14	-.TFEVLATNGDTHLGGEDFDSR.-	2282.37	2	4.45	0.65	1550.3	1	23/40	1.63E9

#82	Q8XBV4 (Q8XBV4) Fe					20.22			2 (2 0 0 0 0)	0.13
	112.69	-.DADAIYANPLLAHLPAVQNK.-	2135.41	2	4.36	0.59	1035.3	1	19/38	3.59E9
	52.86	-.VADDQGFLR.-	1021.11	2	2.78	0.31	1428.2	1	15/16	5.74E8
#83	ILVE_ECOLI (P00510)					20.22			2 (2 0 0 0 0)	0.18
	108.79	-.AGGNYLSSLLVGSEAR.-	1594.75	2	3.74	0.61	1096.1	1	17/30	2.35E9
	127.07	-.DGVLFTPPFTSSALPGITR.-	1977.25	2	4.36	0.67	784.2	1	22/36	3.29E9
#84	Q8X570 (Q8X570) Puta					20.22			2 (2 0 0 0 0)	0.21
	121.80	-.AIPTVYLFQNGQPVDGFQGPQPEEAIR.-	2973.29	2	3.77	0.51	623.2	1	20/52	1.93E9
	126.28 - 126.37	-.KTFQEILAALGTGDALASK.-	1935.21	2	4.34	0.62	1056.0	1	19/36	4.79E9
#85	Q9LAP1 (Q9LAP1) Iha					20.21			2 (2 0 0 0 0)	0.14
	99.14	-.GPM*STLYGSDAM*GGVVNIITR.-	2172.47	2	4.27	0.54	980.7	1	21/40	1.84E9
	77.97	-.IPYPTESQNYNLGAR.-	1723.87	2	3.30	0.44	464.0	1	14/28	2.57E9
#86	CPXR_ECOLI (P16244					20.21			2 (2 0 0 0 0)	0.21
	117.09	-.ILLVDDDRELTSLLK.-	1744.02	2	3.74	0.50	1178.6	1	18/28	2.87E9
	119.17	-.VLGLELGADDYLPKPFNDR.-	2133.39	2	4.15	0.63	954.6	1	19/36	3.68E9
#87	ATPA_ECOLI (P00822					20.20			2 (2 0 0 0 0)	0.20
	124.47 - 124.54	-.ELAAFSQFASDLDDATR.-	1857.96	2	2.53	0.33	340.5	1	11/32	3.24E9
	112.81	-.GPLDHDGFSAVEAIAPGVIER.-	2151.37	2	4.04	0.44	824.2	1	18/40	2.95E9
#88	DCEB_ECOLI (P28302					20.20			2 (2 0 0 0 0)	0.14
	90.80	-.LKDGEDPGYTLYDLSER.-	1972.10	2	3.70	0.59	499.9	1	18/32	1.89E9
	89.28	-.LKDGEDPGYTLYDLSER.-	1972.10	2	4.01	0.52	379.1	1	17/32	2.64E9
#89	Q8XBI9 (Q8XBI9) Hypo					20.19			2 (2 0 0 0 0)	0.14
	90.78	-.ALSGGVGAEELKDFPGYGK.-	1896.09	2	3.76	0.64	907.7	1	18/36	2.47E9
	89.75	-.FTKPVTGGYYFAPSLDK.-	1892.14	2	2.86	0.51	423.0	1	16/32	2.14E9
#90	Q8X641 (Q8X641) Gal					20.18			2 (2 0 0 0 0)	0.21
	146.89 - 148.14	-.SSIPVFGVDALPEALALVK.-	1927.27	2	3.65	0.53	417.3	1	16/36	3.30E9
	87.11 - 87.82	-.VPYVGVDKDNLAEFSK.-	1781.99	2	2.95	0.45	272.3	18	12/30	3.53E9
#91	NAGB_ECOLI (P09375					20.18			2 (2 0 0 0 0)	0.16
	126.82	-.NFFDHVDIPAENINLLNGNAPDIDAECR.-	3185.40	3	3.28	0.38	583.7	4	27/108	2.05E9
	81.50	-.YFNELEAENIK.-	1370.49	2	3.55	0.46	1650.2	1	17/20	2.93E9
#92	EXBB_ECOLI (P18783					20.18			2 (2 0 0 0 0)	0.18
	69.98 - 70.91	-.DLDLEASAAAHPVR.-	1465.59	2	3.55	0.61	1660.9	1	18/26	2.22E9
	82.29 - 83.56	-.SLNQANDIAADFGSK.-	1551.64	2	2.69	0.38	925.9	1	16/28	3.44E9
#93	LPXA_ECO57 (Q8X8X					20.18			2 (2 0 0 0 0)	0.18
	113.43	-.DNEIYQFASIGEVNQDLK.-	2084.23	2	2.54	0.34	210.5	5	10/34	2.24E9
	107.63 - 108.77	-.TLDEVKPEIAELAETYPEVK.-	2275.54	2	3.50	0.54	258.4	1	15/38	3.57E9
#94	Q8X966 (Q8X966) Pyru					20.17			2 (2 0 0 0 0)	0.15
	128.14	-.DVNVPDIGSDEVEVTEILVK.-	2171.39	2	3.08	0.48	333.6	1	16/38	2.61E9
	87.67	-.FGEIEEVELGR.-	1278.39	2	3.36	0.48	1446.4	1	18/20	2.18E9
#95	CARA_ECOLI (P00907					20.16			2 (2 0 0 0 0)	0.15
	167.30	-.FLETDIPVFGICLGHQLLALASGAK.-	2672.11	3	3.19	0.38	474.3	1	28/96	1.46E9
	93.22 - 93.78	-.LTIVPAQTSAEDVLK.-	1585.82	2	3.05	0.61	278.3	1	15/28	3.45E9
#96	TIG_ECOLI (P22257) T					20.16			2 (2 0 0 0 0)	0.13
	78.59	-.FGVEDGSVEGLR.-	1265.36	2	3.12	0.46	1443.8	1	18/22	1.89E9
	74.00 - 74.56	-.INPAGAPTYVPGEYK.-	1577.76	2	2.81	0.34	407.4	1	14/28	2.30E9
#97	Q8X600 (Q8X600) Puta					20.15			2 (2 0 0 0 0)	0.20
	82.40 - 82.44	-.GLSLDVRPGEVHAIM*GPNGSGK.-	2208.49	2	2.73	0.47	322.9	1	16/42	1.24E9
	102.61	-.ILDYIKPDYVHVLYQGR.-	2093.41	3	3.06	0.28	777.6	1	25/64	5.10E9
#98	SSPA_ECOLI (P05838					20.15			2 (2 0 0 0 0)	0.11
	99.43	-.LPQLGIEFSGPGAK.-	1414.63	2	2.69	0.60	819.9	1	17/26	1.81E9
	95.61	-.SVM*TLFSGPTDIYSHQVR.-	2055.30	2	2.96	0.44	666.5	1	16/34	1.60E9
#99	Q7AK38 (Q7AK38) Shi					20.14			2 (2 0 0 0 0)	0.10
	86.89	-.HSLTTSYLDLM*SHSGTSLTQSVAR.-	2609.86	2	2.85	0.46	305.0	1	16/46	1.25E9
	83.98	-.TYVDSLNVIR.-	1180.34	2	2.82	0.49	763.9	1	15/18	2.08E9
#100	Q8X6G0 (Q8X6G0) Pu					20.14			2 (2 0 0 0 0)	0.21
	100.34	-.EM*AQISSLDLGYVGESVK.-	1943.17	2	2.50	0.30	603.7	1	14/34	3.27E9
	89.57 - 90.21	-.LIDTAAVYGNEDAVGEAVR.-	1964.12	2	2.82	0.43	124.8	8	11/36	3.27E9
#101	TALB_ECOLI (P30148)					20.14			2 (2 0 0 0 0)	0.18
	85.51	-.ITESEFLWQHNQDPM*AVDK.-	2305.51	2	2.78	0.49	430.6	1	16/36	1.92E9
	114.86	-.LYQPQDATTNPSLILNAAQIPEYR.-	2718.02	2	2.80	0.39	327.6	2	14/46	3.77E9
#102	Q8XCV2 (Q8XCV2) Pu					10.29			1 (1 0 0 0 0)	0.12
	136.99 - 137.56	-.LEQIEATETEGITALPGAIALLNHLNK.-	2861.24	3	5.87	0.66	2021.9	1	39/104	3.77E9
#103	Q8XCT6 (Q8XCT6) 5,1					10.29			1 (1 0 0 0 0)	0.12
	115.43 - 116.16	-.APGLAVVLVGSNPASQIYVASK.-	2142.48	2	5.70	0.74	1359.0	1	25/42	3.78E9
#104	Q8X622 (Q8X622) Ribo					10.27			1 (1 0 0 0 0)	0.10
	102.85	-.VNIEIDPQTQAVVDTVER.-	2027.22	2	5.36	0.54	1339.8	1	23/34	3.10E9
#105	GALF_ECOLI (P78083					10.27			1 (1 0 0 0 0)	0.04
	149.40 - 149.44	-.PAIGDNPFVVVLPDVVIDDASADPLR.-	2706.04	2	5.33	0.46	623.0	1	22/50	1.38E9
#106	Q8X9B0 (Q8X9B0) Citr					10.26			1 (1 0 0 0 0)	0.03
	158.54 - 159.09	-.TAGSSGANPFACIAAGIASLWGPAHGGANE/	3197.50	3	5.20	0.53	741.0	1	40/132	1.11E9
#107	RPOA_ECOLI (P00574					10.25			1 (1 0 0 0 0)	0.02
	164.63	-.EGVQEDILEILLNLK.-	1726.99	2	5.07	0.54	2124.6	1	21/28	6.23E8
#108	Q8X633 (Q8X633) Out					10.24			1 (1 0 0 0 0)	0.08
	123.55	-.GPM*SSLYGSDALGGVVNIITK.-	2096.39	2	4.88	0.63	1389.0	1	21/40	2.39E9

#109	Q8X882 (Q8X882) Hyp					10.24			1 (1 0 0 0 0)		0.06
	138.50	-.SLLTNETSELDLLDQRPFEQTDFDILK.-	3182.48	3	4.86	0.61	1093.2	1	31/104		2.01E9
#110	YHBG_ECOLI (P3122					10.24			1 (1 0 0 0 0)		0.05
	153.22	-.FILLDEPFAGVDPISVIDIK.-	2202.58	2	4.84	0.52	1220.7	1	24/38		1.70E9
#111	UVRY_ECO57 (P6679					10.24			1 (1 0 0 0 0)		0.06
	109.34	-.YIASDIAQQM*ALSQIEPEKTESPFASLSER.-	3357.69	3	4.81	0.48	1097.1	1	31/116		1.79E9
#112	RNC_ECOLI (P05797)					10.24			1 (1 0 0 0 0)		0.05
	85.55	-.GEAHDQEFTIHCQVSGLSEPVVGTGSSR.-	2986.15	3	4.77	0.60	1080.9	1	31/108		1.62E9
#113	GM4D_ECOLI (P3205					10.23			1 (1 0 0 0 0)		0.13
	118.23	-.FYQASTSELYGLVQEIPQK.-	2202.45	2	4.66	0.51	655.1	1	17/36	1	4.22E9
#114	G6PI_ECOLI (P11537)					10.23			1 (1 0 0 0 0)		0.07
	139.78	-.LLSNFFAQTEALAFGK.-	1758.01	2	4.56	0.65	887.9	1	18/30		2.13E9
#115	DLDH_ECOLI (P00391					10.23			1 (1 0 0 0 0)		0.08
	127.13	-.VIPSIAYTEPEVAWVGLTEK.-	2203.52	2	4.52	0.55	577.8	1	19/38		2.70E9
#116	ODP1_ECOLI (P06958					10.22			1 (1 0 0 0 0)		0.06
	146.28	-.LELPSLQDFGALLEEQSK.-	2018.25	2	4.46	0.65	905.0	1	20/34		2.01E9
#117	Q8X592 (Q8X592) Hyp					10.22			1 (1 0 0 0 0)		0.09
	67.54 - 68.14	-.LSDEVTDSPIVDK.-	1418.53	2	4.45	0.56	969.8	1	19/24		2.94E9
#118	IMDH_ECOLI (P06981)					10.22			1 (1 0 0 0 0)		0.08
	131.47	-.EALTFDDVLLVPAHSTVLPNTADLSTQLTK.-	3211.61	3	4.42	0.40	785.5	1	32/116		2.63E9
#119	SERC_ECO57 (Q8XE					10.22			1 (1 0 0 0 0)		0.10
	124.71 - 125.49	-.AQIFNFSSGPAM*LPVEVLK.-	2065.42	2	4.38	0.60	868.9	1	17/36		3.28E9
#120	Q8X669 (Q8X669) Put					10.21			1 (1 0 0 0 0)		0.03
	162.78 - 163.15	-.IDTEGALLGNIILQVLESHGVPTVNK.-	2732.13	3	4.24	0.48	553.6	1	30/100		9.08E8
#121	Q8XE63 (Q8XE63) Cyt					10.21			1 (1 0 0 0 0)		0.23
	124.41 - 124.98	-.LAAPSEYNQVEYFSNVKPNLFADVINK.-	3072.42	3	4.18	0.57	464.7	1	32/104		7.17E9
#122	RPOB_ECOLI (P0057					10.21			1 (1 0 0 0 0)		0.08
	108.90 - 109.46	-.SVFPIQSYSGNSELQYVSYR.-	2325.52	2	4.15	0.52	662.5	1	17/38		2.67E9
#123	GLS1_ECO57 (Q8XD2					10.20			1 (1 0 0 0 0)		0.06
	152.61 - 153.67	-.QCSTLINTVELATLGATLAAGGVNPLTHK.-	2952.35	3	4.07	0.56	1085.1	1	35/112		1.95E9
#124	UBIE_ECOLI (P27851)					10.20			1 (1 0 0 0 0)		0.09
	120.48	-.LLVLEFSKPIIEPLSK.-	1827.24	2	4.05	0.65	980.1	1	17/30		2.87E9
#125	Q8X8H4 (Q8X8H4) Ou					10.20			1 (1 0 0 0 0)		0.05
	70.20	-.YIAEDNEGNAVNPNLPR.-	1887.00	2	4.02	0.54	752.2	1	17/32		1.69E9
#126	CBPA_ECO57 (Q7AF					10.20			1 (1 0 0 0 0)		0.18
	104.11 - 105.25	-.FKEVAEAWEVLSDEQR.-	1937.10	2	4.00	0.57	871.6	1	17/30		5.77E9
#127	Q8X8G5 (Q8X8G5) Re					10.19			1 (1 0 0 0 0)		0.07
	102.17 - 102.38	-.GLTNEALNVTLVEAGER.-	1786.97	2	3.88	0.57	636.3	1	15/32		2.11E9
#128	Q8XCY2 (Q8XCY2) Tr					10.19			1 (1 0 0 0 0)		0.08
	124.92	-.TPGHPEVGYTAGVETTTGPLGQGIANAVGM	3412.77	3	3.85	0.47	680.3	4	29/136		2.50E9
#129	YEBC_ECO57 (P6717					10.19			1 (1 0 0 0 0)		0.07
	88.72	-.DALEAAGLKADSAEVSM*IPSTK.-	2221.47	2	3.85	0.65	712.6	1	19/42		2.28E9
#130	GLO2_ECOLI (Q47677					10.18			1 (1 0 0 0 0)		0.17
	92.32 - 93.01	-.FPQIVVYGPQETQDK.-	1749.95	2	3.69	0.56	919.2	1	17/28		5.56E9
#131	DEOC_ECO57 (Q8XB					10.18			1 (1 0 0 0 0)		0.04
	132.79	-.FGASSLLASLLK.-	1207.44	2	3.69	0.54	1921.1	1	19/22		1.13E9
#132	HIS6_ECO57 (P60665)					10.18			1 (1 0 0 0 0)		0.13
	101.43 - 102.57	-.ISINSPALADPTLITR.-	1682.94	2	3.67	0.59	704.3	1	18/30		4.13E9
#133	RECA_ECOLI (P03017					10.18			1 (1 0 0 0 0)		0.07
	126.64	-.KLGVDIDNLLCSQPDTGEQALEICDALAR.-	3216.53	3	3.65	0.44	856.8	1	28/112		2.08E9
#134	PANB_ECO57 (Q8X92					10.18			1 (1 0 0 0 0)		0.07
	109.23	-.AVPVCGHLGLTPQSVNIFGGYK.-	2315.65	2	3.61	0.61	865.4	1	19/42		2.10E9
#135	SPEB_ECO57 (P6065					10.18			1 (1 0 0 0 0)		0.08
	122.29 - 122.83	-.M*LSFGGDHFVTLPLLR.-	1820.15	2	3.55	0.46	788.9	1	18/30		2.63E9
#136	RNPH_ECO57 (P6668					10.18			1 (1 0 0 0 0)		0.06
	139.34	-.IIEVQGTAEGEPFTHEELLTLLALAR.-	2852.23	3	3.54	0.43	388.0	24	23/100		1.96E9
#137	FMT_ECO57 (Q8X8F1					10.17			1 (1 0 0 0 0)		0.07
	92.74	-.ASVIDTATNAAPGTILEANK.-	1958.16	2	3.49	0.64	292.6	1	13/38		2.34E9
#138	EFPL_ECOLI (P33028)					10.17			1 (1 0 0 0 0)		0.08
	89.12	-.FKGDDIVDTVTLTR.-	1580.77	2	3.45	0.56	1609.0	1	21/26		2.64E9
#139	KSGA_ECO57 (Q8XA1					10.17			1 (1 0 0 0 0)		0.08
	108.85	-.GQAM*VEIGPGLAALTEPVGER.-	2112.39	2	3.44	0.60	371.4	1	14/40		2.48E9
#140	SLYD_ECOLI (P30856)					10.17			1 (1 0 0 0 0)		0.05
	90.19	-.FNVEVVAIR.-	1047.23	2	3.38	0.41	1647.4	1	15/16		1.54E9
#141	Q8X5N6 (Q8X5N6) Pu					10.17			1 (1 0 0 0 0)		0.04
	155.19	-.DIGLDGVDLYALNVLSNTPLGK.-	2288.58	2	3.37	0.45	844.3	1	18/42		1.37E9
#142	TYSY_ECOLI (P00470)					10.17			1 (1 0 0 0 0)		0.09
	101.00	-.HIDQITTVLNQLK.-	1523.76	2	3.35	0.68	943.5	1	17/24		2.70E9
#143	CARB_ECO57 (P6373					10.17			1 (1 0 0 0 0)		0.02
	161.89	-.LYFEPVTLEDVLEIVR.-	1936.24	2	3.35	0.55	391.1	1	15/30		6.42E8
#144	PUR9_ECO57 (Q8X61					10.17			1 (1 0 0 0 0)		0.06
	101.59	-.GLPVTEVSDYTGFPEM*M*DGR.-	2234.45	2	3.33	0.56	371.1	1	14/38		1.98E9
#145	PURA_ECOLI (P12283					10.16			1 (1 0 0 0 0)		0.07
	138.19 - 138.63	-.VGAGPFPTELFDETGEFLCK.-	2215.44	2	3.29	0.51	293.7	2	12/38		2.26E9

#146	Q8XDY1 (Q8XDY1) Dih					10.16			1 (1 0 0 0 0)	0.18
	120.04 - 120.99	-.QALDM*GLVNTVVPLADLEK.-	2043.37	2	3.27	0.52	362.7	1	13/36	5.59E9
#147	YGGE_ECOLI (P11668					10.16			1 (1 0 0 0 0)	0.04
	61.19 - 61.97	-.SVSLGVAQPDAYKDK.-	1578.75	2	3.26	0.46	953.2	1	18/28	1.27E9
#148	KPY1_ECOLI (P14178)					10.16			1 (1 0 0 0 0)	0.09
	130.33 - 131.26	-.GDLGVEIPVEEVIFAQK.-	1844.10	2	3.24	0.37	246.8	2	14/32	2.84E9
#149	RSUA_ECOLI (P33918					10.16			1 (1 0 0 0 0)	0.09
	109.07	-.TYLVTLESPVADDTAEQFAK.-	2199.40	2	3.21	0.54	302.7	1	14/38	2.98E9
#150	YFIO_ECOLI (P77146)					10.16			1 (1 0 0 0 0)	0.11
	124.49 - 125.79	-.GLTNM*ALDDSALQGFFGVDR.-	2144.35	2	3.19	0.48	902.6	1	18/38	3.44E9
#151	METK_ECOLI (P04384					10.16			1 (1 0 0 0 0)	0.09
	141.93 - 142.78	-.SLQEAVM*EEIIKPILPAEWLTSATK.-	2815.28	3	3.18	0.43	707.3	4	24/96	2.96E9
#152	CYSQ_ECO57 (Q8XC(					10.16			1 (1 0 0 0 0)	0.09
	120.37	-.TLTPDIPVLSEEDPPGWEVR.-	2251.48	2	3.16	0.39	714.8	1	18/38	2.80E9
#153	Q8X6N9 (Q8X6N9) Arg					10.16			1 (1 0 0 0 0)	0.08
	136.02	-.IDSVFGDTAVVTEWLK.-	1781.00	2	3.15	0.56	836.5	1	16/30	2.43E9
#154	Q8XDF3 (Q8XDF3) Asp					10.16			1 (1 0 0 0 0)	0.05
	157.38 - 158.05	-.M*FENITAAPADPILGLADLFR.-	2292.64	2	3.13	0.53	211.8	5	12/40	1.60E9
#155	Q8X9Q1 (Q8X9Q1) Pu					10.15			1 (1 0 0 0 0)	0.04
	70.10	-.EVDAELAKPENSDVTIGR.-	1944.09	2	3.08	0.33	687.0	1	19/34	1.28E9
#156	Q8XAW6 (Q8XAW6) D					10.15			1 (1 0 0 0 0)	0.07
	97.36	-.ILLINPTDSDAVGNAVK.-	1740.98	2	3.08	0.44	447.2	1	16/32	2.09E9
#157	MODE_ECOLI (P46930					10.15			1 (1 0 0 0 0)	0.09
	125.70	-.APWVGITQDEAVAQNADNQLPGIISHIER.-	3144.44	3	3.04	0.01	212.6	274	19/112	2.98E9
#158	RvrsDB|00000576					10.15			1 (1 0 0 0 0)	0.04
	155.63 - 156.50	-.GRVWLRIWILPQILYLLATYLLELM.-	3088.83	3	3.04	0.35	549.9	3	26/96	1.40E9
#159	Q8XBD3 (Q8XBD3) Lip					10.15			1 (1 0 0 0 0)	0.07
	76.31	-.YQISVKPQGYQQAVTVK.-	1938.22	2	3.02	0.56	255.4	1	15/32	2.36E9
#160	APAH_ECO57 (Q8XA1					10.15			1 (1 0 0 0 0)	0.04
	155.79	-.LTPLLEAPDADELLNWLR.-	2080.37	2	2.99	0.02	302.0	2	14/34	1.26E9
#161	Q8X5Q1 (Q8X5Q1) Hy					10.15			1 (1 0 0 0 0)	0.08
	130.35	-.IWQQDDLPAELEPYINVVK.-	2271.55	2	2.99	0.39	219.3	1	13/36	2.62E9
#162	FTSH_ECO57 (Q8X9L					10.15			1 (1 0 0 0 0)	0.05
	151.37 - 152.11	-.GTPGFSGADLANLVNEAALFAAR.-	2263.50	2	2.93	0.29	267.5	3	13/44	1.52E9
#163	DDLB_ECO57 (Q8X9Y					10.14			1 (1 0 0 0 0)	0.06
	86.48	-.VVAENALQDALR.-	1299.46	2	2.53	0.47	1103.7	1	16/22	1.87E9
#164	RvrsDB|00002753					10.14			1 (1 0 0 0 0)	0.18
	102.22 - 103.35	-.EDEHYKSIEDK.-	1393.44	2	2.87	0.03	528.7	5	12/20	5.56E9
#165	Q7DBF8 (Q7DBF8) Glu					10.14			1 (1 0 0 0 0)	0.03
	66.55	-.VLSGPQAQPAGDKAEFIEK.-	1986.22	2	2.86	0.44	349.9	1	16/36	1.09E9
#166	Q8X9C1 (Q8X9C1) Put					10.14			1 (1 0 0 0 0)	0.10
	100.49 - 101.04	-.LVADLPESFYTQAAK.-	1653.86	2	2.83	0.50	131.6	13	10/28	3.05E9
#167	GADC_ECO57 (P5822					10.14			1 (1 0 0 0 0)	0.05
	70.24 - 70.55	-.ANTGVTLEPINSQNAPK.-	1754.92	2	2.80	0.47	343.8	1	15/32	1.61E9
#168	Q8XCU8 (Q8XCU8) Ph					10.14			1 (1 0 0 0 0)	0.04
	64.68	-.KHQFAQSLNYEIAK.-	1677.89	2	2.79	0.32	388.1	1	13/26	1.39E9
#169	FABH_ECOLI (P24249					10.14			1 (1 0 0 0 0)	0.05
	93.70	-.YALVVGSDVLAR.-	1263.47	2	2.78	0.40	904.7	1	16/22	1.67E9
#170	SYN_ECO57 (P58694)					10.14			1 (1 0 0 0 0)	0.05
	68.48	-.VVASPGQGQQFEIQTSK.-	1804.98	2	2.73	0.54	240.3	3	12/32	1.49E9
#171	Q8X9A8 (Q8X9A8) Suc					10.13			1 (1 0 0 0 0)	0.04
	65.63 - 66.43	-.IKPYLLNNGQNPPAR.-	1695.95	2	2.70	0.26	326.2	11	12/28	1.42E9
#172	RvrsDB|00004288					10.13			1 (1 0 0 0 0)	0.05
	94.16	-.GTDEADAAFLASQRDELGFAK.-	2213.35	2	2.69	0.04	468.7	1	15/40	1.71E9
#173	PHOB_ECOLI (P08402					10.13			1 (1 0 0 0 0)	0.05
	86.94	-.GLETGADDYITKPFSPK.-	1840.02	2	2.69	0.31	332.1	10	11/32	1.67E9
#174	HCHA_ECO57 (Q8XB7					10.13			1 (1 0 0 0 0)	0.15
	91.74 - 92.89	-.LLTGDSPFAANALGK.-	1475.67	2	2.67	0.20	669.9	3	14/28	4.87E9
#175	SPEE_ECO57 (P66834					10.13			1 (1 0 0 0 0)	0.04
	13.18 - 14.22	-.HLSTEIIQAR.-	1168.33	2	2.64	0.37	900.5	1	15/18	1.13E9
#176	RvrsDB|00002252					10.13			1 (1 0 0 0 0)	0.12
	134.40 - 134.98	-.EGDAMQILAALLK.-	1373.64	2	2.61	0.10	864.7	2	15/24	3.76E9
#177	RvrsDB|00004199					10.13			1 (1 0 0 0 0)	0.18
	96.90 - 97.43	-.TSQEDTAEQRSIVESFLQR.-	2225.36	2	2.59	0.10	437.7	2	14/36	5.82E9
#178	HDHA_ECOLI (P25529					10.13			1 (1 0 0 0 0)	0.10
	113.39 - 113.98	-.VNGIAPGAILTDALK.-	1453.71	2	2.59	0.57	383.2	1	17/28	3.27E9
#179	TRPA_ECO57 (Q8X7B					10.13			1 (1 0 0 0 0)	0.07
	115.80	-.VGVDSVLVADVPVEESAPFR.-	2086.33	2	2.55	0.55	392.2	1	14/38	2.16E9
#180	YGGS_ECO57 (P6708					10.13			1 (1 0 0 0 0)	0.04
	156.57	-.SGIQLAELDELAAAVAELPR.-	2067.33	2	2.55	0.18	265.8	17	11/38	1.15E9
#181	SECY_ECOLI (P03844					10.13			1 (1 0 0 0 0)	0.16
	117.89 - 118.65	-.GTIIEM*FNM*FSGGALSR.-	1864.14	2	2.53	0.33	425.4	1	13/32	5.05E9
#182	RLMB_ECO57 (P63179					10.13			1 (1 0 0 0 0)	0.07
	66.57 - 67.49	-.KVACGAAESVPLIR.-	1471.72	2	2.51	0.45	755.1	1	16/26	2.33E9

#183	YGEW_ECO57 (Q8X6					10.13			1 (1 0 0 0 0)	0.09
	134.73	-.PYIIAAMILSRK.-	1376.74	1	2.50	0.19	332.5	7	11/22	2.82E9
#184	RvrsDB|00001234					10.12			1 (1 0 0 0 0)	0.04
	66.85	-.YDLLNPNRVVGSK.-	1475.68	1	2.17	0.09	223.6	6	11/24	1.38E9
#185	Q8XCN4 (Q8XCN4) Pu					10.11			1 (1 0 0 0 0)	0.02
	63.10	-.NPLYSTAIGK.-	1064.22	1	1.94	0.37	678.8	1	14/18	5.57E8
#186	FLIQ_ECOLI (P33134)					10.10			1 (1 0 0 0 0)	0.32
	105.21 - 105.51	-.M*TPESVM*M*MGTEAM*K.-	1739.07	1	2.06	0.03	196.9	30	10/28	1.03E10
#187	PYRG_ECOLI (P0839					10.09			1 (1 0 0 0 0)	0.03
	66.08	-.YEVNNM*LLK.-	1140.34	1	1.81	0.22	184.9	4	9/16	9.37E8
