# Supplementary material for: Proteins other than the locus of enterocyte effacement-encoded proteins contribute to Escherichia coli O157:H7 adherence to bovine rectoanal junction stratified squamous epithelial cells
Source: BMC Microbiol. 2012 Jun 12;12:103. doi: 10.1186/1471-2180-12-103 (PMC3420319; doi:10.1186/1471-2180-12-103)
Supplement: Additional file 10 — http://www.biomedcentral.com/imedia/1610501146675419/supp10.pdf. DATA SHEETS: O157-DMEM MS/MS data sheet 6. [file 1471-2180-12-103-S10.pdf]

DMEM-06 SequestReport

	Reference					Score	Accession		Peptides (Hits)		Area
	Time(s)	Sequence	MH+	Charge	XC	Delta Cn	Sp	RSp	Ions	Count	Peak Area
#1	Q8XBL1 (Q8XBL1) PTS					330.33			33 (33 0 0 0 0)		5.17
	160.36 - 161.04	-.DTGTIEIIAPLSGEIVNIEDVPDVVFAEK.-	3085.45	2	3.22	0.64	290.7	1	15/56		2.00E9
	158.39 - 159.55	-.DTGTIEIIAPLSGEIVNIEDVPDVVFAEK.-	3085.45	2	5.02	0.63	927.5	1	21/56		5.17E9
	158.33 - 159.51	-.DTGTIEIIAPLSGEIVNIEDVPDVVFAEK.-	3085.45	3	6.51	0.63	1427.3	1	37/112		1.17E10
	32.23 - 32.91	-.IVGDGIAIKPTGNK.-	1383.62	2	3.65	0.57	1280.0	1	19/26		5.77E8
	33.83 - 34.53	-.IVGDGIAIKPTGNK.-	1383.62	2	3.18	0.53	884.3	1	16/26		3.67E8
	37.00 - 38.39	-.IVGDGIAIKPTGNK.-	1383.62	2	3.11	0.47	1051.8	1	19/26		1.72E8
	40.10 - 41.63	-.IVGDGIAIKPTGNK.-	1383.62	2	3.52	0.59	1188.1	1	19/26		2.63E8
	42.16 - 43.45	-.IVGDGIAIKPTGNK.-	1383.62	2	3.25	0.54	661.7	1	16/26		6.24E8
	43.99 - 45.15	-.IVGDGIAIKPTGNK.-	1383.62	2	3.52	0.53	981.3	1	18/26		1.83E9
	30.13	-.IVGDGIAIKPTGNK.-	1383.62	1	1.96	0.33	286.1	1	13/26		3.12E8
	45.66 - 46.80	-.IVGDGIAIKPTGNK.-	1383.62	2	4.30	0.53	707.4	1	16/26		2.18E9
	47.30 - 48.70	-.IVGDGIAIKPTGNK.-	1383.62	2	3.72	0.55	899.9	1	18/26		2.11E9
	48.40	-.IVGDGIAIKPTGNK.-	1383.62	1	3.40	0.37	471.1	1	15/26		2.57E8
	49.32 - 50.69	-.IVGDGIAIKPTGNK.-	1383.62	2	2.97	0.54	537.7	1	14/26		2.13E9
	54.06	-.IVGDGIAIKPTGNK.-	1383.62	2	2.95	0.47	848.5	2	17/26		5.71E8
	56.73 - 57.79	-.IVGDGIAIKPTGNK.-	1383.62	2	3.89	0.57	1350.0	1	21/26		6.06E8
	25.46 - 27.08	-.IVGDGIAIKPTGNK.-	1383.62	2	2.75	0.47	773.1	1	15/26		2.34E8
	29.56 - 30.68	-.IVGDGIAIKPTGNK.-	1383.62	2	3.20	0.52	922.5	1	16/26		7.54E8
	45.57 - 49.40	-.IVGDGIAIKPTGNK.-	1383.62	1	1.92	0.17	268.7	3	12/26		6.03E8
	148.94	-.KDTGTIEIIAPLSGEIVNIEDVPDVVFAEK.-	3213.62	3	4.03	0.52	1056.1	1	33/116		1.10E9
	76.62 - 77.75	-.LSGSVTVGETPVIR.-	1415.62	1	1.99	0.43	104.0	2	10/26		4.97E9
	76.35 - 77.73	-.LSGSVTVGETPVIR.-	1415.62	2	3.68	0.53	582.1	1	16/26		1.53E10
	76.73 - 77.39	-.LSGSVTVGETPVIR.-	1415.62	1	2.51	0.52	176.2	1	13/26		3.33E9
	11.42	-.M*VAPVDGTIGK.-	1104.30	1	2.26	0.35	201.0	1	13/20		1.74E9
	11.27	-.M*VAPVDGTIGK.-	1104.30	1	1.96	0.22	257.0	1	14/20		2.42E9
	57.73 - 58.28	-.MVAPVDGTIGK.-	1088.30	1	1.97	0.33	500.8	1	13/20		3.34E8
	84.87 - 85.47	-.STLTPVVISNM*DEIK.-	1663.92	2	3.45	0.57	728.6	1	17/28		6.62E9
	85.16	-.STLTPVVISNM*DEIK.-	1663.92	1	2.11	0.36	65.6	5	10/28		1.66E9
	109.63 - 110.19	-.STLTPVVISNM*DEIKELIK.-	2147.52	2	3.51	0.51	615.7	1	17/36		7.11E9
	113.58 - 114.99	-.STLTPVVISNM*DEIKELIK.-	2147.52	2	2.95	0.53	325.6	1	12/36		3.01E9
	128.89 - 129.45	-.STLTPVVISNMDEIKELIK.-	2131.52	2	2.89	0.46	465.1	1	15/36		2.56E9
	117.11 - 118.37	-.VGDTVIEFDLPQLEEK.-	1833.03	2	4.47	0.58	1257.6	2	19/30		2.88E10
	117.00	-.VKVGDTVIEFDLPQLEEK.-	2060.33	2	5.25	0.47	1765.3	1	23/34		3.10E9
#2	Q9LAN9 (Q9LAN9) Pu					320.37			32 (32 0 0 0 0)		6.30
	112.42 - 113.77	-.AVGQGFAGGLSALASQHGVNV.-	1941.14	2	5.34	0.61	1633.0	1	24/40		2.51E10
	89.81 - 91.25	-.EAPTM*NVAM*VGLGWDAR.-	1851.10	2	3.69	0.43	464.5	1	16/32		6.06E9
	101.21	-.EAPTM*NVAMVGLGWDAR.-	1835.10	2	2.86	0.32	988.6	1	18/32		3.13E9
	129.90 - 131.19	-.FDLSEDASTETAM*VFGELYR.-	2298.47	2	3.74	0.63	595.3	1	15/38		8.70E9
	126.64 - 127.72	-.FDLSEDASTETAM*VFGELYR.-	2298.47	2	3.95	0.67	712.2	1	16/38		2.36E9
	131.50 - 132.71	-.FDLSEDASTETAM*VFGELYR.-	2298.47	2	7.46	0.72	2629.2	1	26/38		1.24E10
	133.28 - 133.89	-.FDLSEDASTETAM*VFGELYR.-	2298.47	2	5.05	0.68	1148.4	1	19/38		7.36E9
	137.79 - 138.48	-.FDLSEDASTETAMVFGELYR.-	2282.47	2	4.52	0.65	536.1	1	14/38		2.58E9
	55.40 - 56.61	-.KQNFGM*VSNSFM*R.-	1578.80	2	2.83	0.29	450.1	1	14/24		1.25E9
	53.60 - 54.91	-.KQNFGM*VSNSFM*R.-	1578.80	2	3.43	0.55	448.2	1	14/24		1.32E9
	70.75 - 72.11	-.KQNFGM*VSNSFMR.-	1562.80	2	2.71	0.03	674.0	1	18/24		1.95E9
	68.79	-.TGEGDGDDEQVKIDLTK.-	1820.89	2	3.83	0.50	1170.9	1	19/32		1.21E9
	65.32 - 66.42	-.TGEGDGDDEQVKIDLTK.-	1820.89	2	4.59	0.61	1836.3	1	22/32		3.62E9
	65.96 - 66.93	-.TGEGDGDDEQVKIDLTK.-	1820.89	3	3.61	0.38	509.6	1	24/64		2.44E9
	67.02 - 67.58	-.TGEGDGDDEQVKIDLTK.-	1820.89	2	4.87	0.60	1029.0	1	19/32		3.16E9
	64.02 - 65.44	-.TGEGDGDDEQVKIDLTK.-	1820.89	3	3.48	0.51	617.0	1	25/64		2.64E9
	66.56 - 67.74	-.TSPDGAVEHQGDNRTGEGDGDDEQVKIDLT	3285.35	3	4.02	0.35	224.6	7	27/120		1.31E9
	64.84 - 65.99	-.TSPDGAVEHQGDNRTGEGDGDDEQVKIDLT	3285.35	3	4.54	0.40	937.5	1	34/120		1.61E9
	102.08	-.VLSDAHFIFFNNK.-	1552.76	2	4.91	0.44	2061.1	1	19/24		2.57E9
	104.42 - 105.69	-.VLSDAHFIFFNNK.-	1552.76	2	3.84	0.44	1131.1	1	18/24		3.53E9
	114.47	-.VTDGQGFDLDASVFAVGEDGK.-	2128.24	2	5.35	0.74	1613.6	1	21/40		7.42E9
	122.13 - 122.71	-.VTDGQGFDLDASVFAVGEDGK.-	2128.24	2	3.76	0.64	695.7	1	17/40		1.77E9
	114.07 - 115.16	-.VTDGQGFDLDASVFAVGEDGK.-	2128.24	2	4.72	0.67	1499.8	1	22/40		6.95E9
	127.09	-.VTDGQGFDLDASVFAVGEDGK.-	2128.24	2	3.63	0.63	917.4	1	17/40		1.46E9
	120.18 - 121.21	-.VTDGQGFDLDASVFAVGEDGK.-	2128.24	2	5.29	0.66	1119.8	1	18/40		2.70E9
	117.85 - 119.08	-.VTDGQGFDLDASVFAVGEDGK.-	2128.24	2	5.15	0.70	1781.5	1	23/40		4.05E9
	112.52 - 113.89	-.VTDGQGFDLDASVFAVGEDGK.-	2128.24	2	5.19	0.73	1648.8	1	21/40		8.20E9
	115.86 - 116.96	-.VTDGQGFDLDASVFAVGEDGK.-	2128.24	2	3.99	0.58	924.6	1	18/40		5.47E9
	110.85	-.VTDGQGFDLDASVFAVGEDGK.-	2128.24	2	3.06	0.50	303.4	1	12/40		3.96E9
	147.18 - 148.39	-.VTDGQGFDLDASVFAVGEDGK.-	2128.24	2	3.49	0.51	518.2	1	15/40		1.96E9
	149.64 - 150.68	-.VTDGQGFDLDASVFAVGEDGK.-	2128.24	2	2.71	0.49	332.9	2	13/40		1.44E9
	204.46	-.VTDGQGFDLDASVFAVGEDGK.-	2128.24	2	2.78	0.53	299.9	3	12/40		6.97E7
#3	AHPC_ECOLI (P26427					290.33			29 (29 0 0 0 0)		6.13
	40.64	-.AAQYVASHPGEVCPAK.-	1685.86	2	2.53	0.53	493.2	1	14/30		1.61E8
	43.69 - 45.19	-.AAQYVASHPGEVCPAK.-	1685.86	2	3.57	0.61	976.4	1	19/30		3.51E8
	10.63	-.AAQYVASHPGEVCPAK.-	1685.86	2	4.62	0.70	1164.2	1	20/30		3.61E9

	169.76	-.ATFVVDPQGIIQAIEVTAEGIGRDASDLLR.-	3156.54	3	4.55	0.58	646.9	1	36/116	1.35E9
	31.77 - 43.40	-.DASDLLR.-	789.86	1	2.19	0.20	286.0	3	9/12	3.98E9
	95.82	-.EGEATLAPSLDLVGK.-	1500.68	1	2.99	0.55	201.7	1	13/28	1.26E9
	95.43 - 96.76	-.EGEATLAPSLDLVGK.-	1500.68	2	2.59	0.47	448.7	1	15/28	7.35E9
	116.10 - 116.62	-.EGEATLAPSLDLVGKI.-	1613.84	2	3.91	0.57	1074.4	1	22/30	1.87E10
	116.25	-.EGEATLAPSLDLVGKI.-	1613.84	1	2.01	0.26	242.7	1	14/30	2.43E9
	83.39 - 83.96	-.LGVDVYAVSTDTHFTHK.-	1891.07	3	3.89	0.54	1469.1	1	30/64	6.78E9
	83.32 - 84.41	-.LGVDVYAVSTDTHFTHK.-	1891.07	2	3.75	0.53	926.3	1	19/32	8.66E9
	81.38	-.LGVDVYAVSTDTHFTHK.-	1891.07	3	3.52	0.48	1618.6	1	30/64	1.54E9
	67.84 - 69.16	-.NFDNMREDEGLADR.-	1682.75	2	3.28	0.40	524.4	1	13/26	2.54E9
	85.37	-.NGEFIEITEK.-	1180.29	1	1.81	0.29	719.2	1	13/18	1.16E9
	82.47 - 83.07	-.NGEFIEITEKDTEGR.-	1738.83	2	4.01	0.57	1255.2	1	19/28	3.94E9
	85.30 - 86.49	-.NGEFIEITEKDTEGR.-	1738.83	2	3.73	0.54	978.9	1	17/28	3.33E9
	77.68	-.NGEFIEITEKDTEGR.-	1738.83	2	3.05	0.61	525.7	1	14/28	1.87E9
	84.83 - 86.15	-.NQAFKNGEFIEITEK.-	1768.95	2	3.94	0.43	1114.2	1	20/28	3.69E9
	86.75	-.NQAFKNGEFIEITEK.-	1768.95	2	4.16	0.53	1432.1	1	22/28	2.75E9
	88.73 - 89.29	-.NQAFKNGEFIEITEKDTEGR.-	2327.49	3	6.60	0.58	3060.9	1	42/76	7.00E9
	88.83 - 89.35	-.NQAFKNGEFIEITEKDTEGR.-	2327.49	2	4.70	0.55	1191.0	1	23/38	3.52E9
	86.77	-.NQAFKNGEFIEITEKDTEGR.-	2327.49	3	5.16	0.44	1796.5	1	32/76	2.70E9
	10.82 - 11.74	-.SLINTK.-	675.80	1	1.80	0.14	356.9	1	8/10	4.77E9
	95.62 - 96.13	-.WKEGEATLAPSLDLVGK.-	1815.06	2	3.88	0.55	933.7	1	17/32	2.98E9
	113.14 - 113.66	-.WKEGEATLAPSLDLVGKI.-	1928.22	3	3.84	0.45	873.9	1	28/68	5.17E9
	112.46 - 113.68	-.WKEGEATLAPSLDLVGKI.-	1928.22	2	4.94	0.49	1340.2	1	22/34	8.71E9
	75.58 - 76.83	-.YAM*IGDPTGALTR.-	1382.57	2	3.76	0.56	957.8	1	18/24	1.90E10
	92.26 - 93.19	-.YAM*IGDPTGALTRNFDNM*REDEGLADR.-	3062.30	3	3.67	0.51	661.9	3	29/104	2.23E9
	87.08 - 87.94	-.YAMIGDPTGALTR.-	1366.57	2	3.44	0.51	654.0	1	17/24	4.41E9
#4	RL6_ECOLI (P02390) 5					290.27			29 (29 0 0 0 0)	4.18
	107.33	-.ALLNSM*VIGVTEGFTK.-	1696.99	3	3.29	0.52	1441.2	1	28/60	1.44E9
	107.14	-.ALLNSM*VIGVTEGFTK.-	1696.99	1	1.83	0.48	53.7	7	11/30	1.99E9
	107.08	-.ALLNSM*VIGVTEGFTK.-	1696.99	1	3.11	0.58	108.8	2	14/30	1.81E9
	106.66 - 107.75	-.ALLNSM*VIGVTEGFTK.-	1696.99	2	5.49	0.68	1015.1	1	20/30	1.99E10
	99.57 - 100.15	-.ALLNSM*VIGVTEGFTKK.-	1825.16	2	2.53	0.37	293.3	2	12/32	1.97E9
	129.64 - 130.20	-.ALLNSMVIGVTEGFTK.-	1680.99	2	3.66	0.57	850.9	1	16/30	2.24E9
	72.82 - 73.49	-.APVVVPAGVDVK.-	1151.38	1	2.10	0.10	721.7	1	14/22	4.12E9
	72.69 - 73.70	-.APVVVPAGVDVK.-	1151.38	2	3.65	0.56	931.0	1	18/22	9.45E9
	72.67 - 73.85	-.APVVVPAGVDVK.-	1151.38	1	1.95	0.47	656.0	1	13/22	8.29E9
	70.05	-.DGYADGWAQAGTAR.-	1439.47	1	1.95	0.38	277.0	1	14/26	1.16E9
	61.95 - 63.28	-.DGYADGWAQAGTAR.-	1439.47	2	2.90	0.50	964.6	1	16/26	1.22E9
	73.28	-.DGYADGWAQAGTAR.-	1439.47	2	3.16	0.49	1206.8	1	17/26	1.46E9
	69.47 - 70.18	-.DGYADGWAQAGTAR.-	1439.47	2	3.90	0.58	2263.1	1	21/26	2.86E9
	69.80 - 70.42	-.DGYADGWAQAGTAR.-	1439.47	1	2.05	0.24	148.8	4	11/26	1.54E9
	82.82 - 83.41	-.GADKQVIGQVAADLR.-	1541.74	3	4.60	0.49	1891.7	1	33/56	2.02E9
	82.94	-.GADKQVIGQVAADLR.-	1541.74	1	2.16	0.35	238.9	1	13/28	1.56E9
	44.18 - 49.75	-.HADNTLTFGPR.-	1229.33	1	2.02	0.36	430.6	1	13/20	1.06E9
	44.51 - 45.79	-.HADNTLTFGPR.-	1229.33	2	3.71	0.63	918.2	1	16/20	1.88E9
	46.42 - 47.56	-.HADNTLTFGPR.-	1229.33	2	3.83	0.62	1068.2	1	18/20	2.80E9
	48.18 - 49.51	-.HADNTLTFGPR.-	1229.33	2	3.21	0.65	727.9	1	15/20	2.18E9
	58.39 - 59.33	-.HADNTLTFGPR.-	1229.33	2	3.00	0.47	1097.0	1	17/20	8.70E8
	42.09 - 43.87	-.HADNTLTFGPR.-	1229.33	2	3.59	0.49	880.4	1	16/20	1.20E9
	64.70 - 65.90	-.INGQVITIK.-	986.19	2	3.00	0.29	838.1	1	15/16	3.25E9
	70.29 - 71.48	-.KLQLVGVGYR.-	1133.37	2	3.28	0.49	1500.4	1	17/18	3.36E9
	70.39 - 70.97	-.KLQLVGVGYR.-	1133.37	1	2.22	0.55	356.2	1	14/18	2.35E9
	79.94	-.LQLVGVGYR.-	1005.20	2	3.38	0.49	870.6	1	14/16	1.53E9
	79.23 - 80.58	-.QVIGQVAADLR.-	1170.34	2	3.40	0.58	1196.2	1	18/20	5.61E9
	11.57	-.TLNDAVEVK.-	989.11	1	1.91	0.24	297.0	6	9/16	2.05E9
	11.61	-.TLNDAVEVK.-	989.11	2	3.07	0.43	568.4	1	15/16	1.53E9
#5	Q8X9P9 (Q8X9P9) Put					250.32			25 (25 0 0 0 0)	3.60
	103.80 - 104.44	-.GDSDFIFYNNLTSSDGSVTHTGDNR.-	2720.76	3	5.32	0.54	640.0	1	33/96	3.39E9
	100.00 - 101.31	-.GDSDFIFYNNLTSSDGSVTHTGDNR.-	2720.76	3	5.36	0.55	763.5	1	33/96	5.25E9
	98.35	-.GDSDFIFYNNLTSSDGSVTHTGDNR.-	2720.76	2	3.07	0.51	392.0	1	16/48	1.51E9
	101.87 - 103.27	-.GDSDFIFYNNLTSSDGSVTHTGDNR.-	2720.76	3	4.61	0.48	990.4	1	37/96	4.96E9
	101.52	-.GDSDFIFYNNLTSSDGSVTHTGDNR.-	2720.76	2	3.30	0.39	366.9	1	16/48	2.34E9
	91.17	-.IIFVVTIHDAQAR.-	1483.74	2	2.83	0.44	1618.6	1	19/24	1.73E9
	69.10 - 69.87	-.IKLDAVPSEVDK.-	1314.51	2	3.40	0.36	652.6	1	14/22	2.57E9
	132.62 - 133.76	-.IKLDAVPSEVDKIIFVVTIHDAQAR.-	2779.23	3	3.57	0.55	668.9	1	31/96	3.11E9
	130.58 - 131.32	-.LDAVPSEVDKIIFVVTIHDAQAR.-	2537.90	3	4.67	0.56	842.2	1	35/88	1.73E9
	29.93 - 30.26	-.LVNDDNQTEVAR.-	1374.44	2	3.16	0.59	1012.2	1	16/22	4.49E8
	100.58 - 101.14	-.NVLVGLGWDAR.-	1200.37	2	3.86	0.59	1321.5	1	17/20	3.04E9
	78.23	-.RQSFGQVSGAFIR.-	1453.63	3	3.07	0.36	2166.2	1	30/48	1.14E9
	78.04 - 78.66	-.RQSFGQVSGAFIR.-	1453.63	2	4.18	0.43	1240.5	1	17/24	3.51E9
	123.48 - 124.65	-.STDGQDFDLDASAFLLASNGK.-	2173.28	2	5.28	0.63	1547.3	1	22/40	6.67E9
	132.77 - 134.16	-.STDGQDFDLDASAFLLASNGK.-	2173.28	2	4.31	0.58	1647.6	1	21/40	1.93E9
	126.97 - 128.20	-.STDGQDFDLDASAFLLASNGK.-	2173.28	2	4.69	0.56	814.4	1	20/40	4.68E9
	129.01 - 130.29	-.STDGQDFDLDASAFLLASNGK.-	2173.28	2	4.50	0.58	1006.1	1	19/40	2.93E9

	188.67 - 190.34	-.STDGQDFDLDASAFLLASNGK.-	2173.28	2	3.45	0.53	260.2	1	18/40	7.95E8
	130.81 - 132.11	-.STDGQDFDLDASAFLLASNGK.-	2173.28	2	5.08	0.60	1264.3	1	22/40	3.30E9
	125.24 - 126.45	-.STDGQDFDLDASAFLLASNGK.-	2173.28	2	5.56	0.67	1859.8	1	25/40	6.50E9
	121.53 - 122.90	-.STDGQDFDLDASAFLLASNGK.-	2173.28	2	4.40	0.43	1257.7	1	21/40	9.22E9
	190.92 - 191.99	-.STDGQDFDLDASAFLLASNGK.-	2173.28	2	4.46	0.57	498.5	1	21/40	9.35E8
	95.16 - 95.95	-.VRGDSDFIFYNNLTSSDGSVTHTGDNR.-	2976.08	3	6.46	0.61	2523.0	1	42/104	3.36E9
	92.52 - 93.17	-.VRGDSDFIFYNNLTSSDGSVTHTGDNR.-	2976.08	3	6.05	0.63	2172.2	1	39/104	2.07E9
	141.53	-.YDLTEDASTETAMLFGELYR.-	2326.52	2	3.68	0.59	566.4	1	15/38	2.62E9
#6	RL5_ECO57 (P62401)					240.27			24 (24 0 0 0 0)	6.30
	133.06 - 133.30	-.ALLAAFDFPFR.-	1268.49	2	4.35	0.58	1935.3	1	18/20	4.51E9
	117.34 - 117.91	-.ALLAAFDFPFRK.-	1396.66	2	3.38	0.39	1022.2	1	15/22	3.97E9
	101.61 - 102.40	-.EQIIFPEIDYDKVDR.-	1881.08	2	4.23	0.51	847.6	1	20/28	1.11E10
	100.90 - 102.06	-.EQIIFPEIDYDKVDR.-	1881.08	3	3.06	0.45	575.7	2	23/56	7.67E9
	100.69 - 101.83	-.EQIIFPEIDYDKVDR.-	1881.08	2	3.61	0.52	620.0	1	17/28	1.03E10
	80.75	-.GLDITITTTAK.-	1134.31	2	3.36	0.63	1278.0	1	17/20	2.91E9
	78.96 - 79.89	-.GLDITITTTAK.-	1134.31	2	2.89	0.51	972.9	1	16/20	2.39E9
	81.59 - 82.18	-.ITLNM*GVGEAIADK.-	1448.67	2	3.08	0.41	1264.1	1	19/26	3.28E9
	73.39 - 73.94	-.ITLNM*GVGEAIADKK.-	1576.84	3	3.37	0.41	837.2	1	27/56	2.98E9
	73.92 - 75.21	-.ITLNM*GVGEAIADKK.-	1576.84	2	3.28	0.56	669.8	1	17/28	6.08E9
	72.29 - 73.35	-.ITLNM*GVGEAIADKK.-	1576.84	2	3.28	0.37	729.7	1	17/28	7.48E9
	86.79 - 86.92	-.ITLNMGVGEAIADKK.-	1560.84	2	2.69	0.45	495.3	1	14/28	2.24E9
	79.57 - 80.73	-.KLM*TEFNYNSVM*QVPR.-	1990.29	2	3.70	0.38	585.4	1	18/30	5.25E9
	75.77 - 76.71	-.KLM*TEFNYNSVM*QVPR.-	1990.29	2	3.08	0.52	580.0	1	16/30	2.49E9
	10.97 - 11.01	-.LHDYYKDEVVK.-	1409.57	1	2.52	0.41	514.5	1	12/20	1.54E9
	105.56 - 106.29	-.LLDNAAADLAAISGQK.-	1571.76	2	4.14	0.62	1520.0	1	20/30	4.07E9
	107.77	-.LLDNAAADLAAISGQKPLITK.-	2124.47	3	3.12	0.43	709.0	1	37/80	2.26E9
	110.77 - 112.16	-.LLDNAAADLAAISGQKPLITK.-	2124.47	3	4.21	0.52	636.9	1	34/80	2.95E10
	110.83 - 112.18	-.LLDNAAADLAAISGQKPLITK.-	2124.47	2	5.34	0.64	1560.6	1	22/40	1.64E10
	87.92	-.LM*TEFNYNSVM*QVPR.-	1862.12	2	4.34	0.70	1348.8	1	18/28	1.75E9
	81.36 - 81.90	-.LM*TEFNYNSVM*QVPR.-	1862.12	2	4.16	0.52	812.2	1	17/28	2.75E9
	85.70 - 86.26	-.LM*TEFNYNSVM*QVPR.-	1862.12	2	4.68	0.63	1309.4	1	19/28	4.65E9
	101.44	-.LM*TEFNYNSVMQVPR.-	1846.12	2	2.58	0.46	344.6	1	12/28	2.41E9
	92.37 - 92.64	-.LMTEFNYNSVM*QVPR.-	1846.12	2	3.41	0.51	575.6	1	14/28	1.68E9
#7	DPS_ECOLI (P27430)					220.30			22 (22 0 0 0 0)	2.56
	79.92	-.AIGEAKDDDTADILTAASR.-	1934.05	3	4.46	0.37	1371.5	1	28/72	3.69E9
	79.27 - 80.66	-.AIGEAKDDDTADILTAASR.-	1934.05	2	5.23	0.59	1890.3	1	24/36	7.59E9
	53.58 - 54.70	-.ATNLLYTR.-	952.09	2	2.94	0.37	1092.6	1	13/14	5.52E8
	53.09 - 56.01	-.ATNLLYTR.-	952.09	1	2.06	0.34	195.9	1	10/14	2.00E9
	60.62 - 61.21	-.ATNLLYTRNDVSDSEK.-	1826.94	2	4.31	0.55	1746.0	1	20/30	1.24E9
	62.07 - 63.46	-.ATNLLYTRNDVSDSEK.-	1826.94	2	4.01	0.61	1356.5	1	20/30	1.71E9
	94.61 - 95.19	-.AVQLGGVALGTTQVINSK.-	1757.03	1	3.78	0.57	594.9	1	18/34	1.79E9
	94.38 - 95.67	-.AVQLGGVALGTTQVINSK.-	1757.03	2	4.94	0.60	2523.3	1	26/34	8.16E9
	80.17	-.DDDTADILTAASR.-	1364.40	2	3.23	0.43	1197.8	1	18/24	1.66E9
	162.70	-.DLDKFLWFIESNIE.-	1769.98	2	4.02	0.38	878.7	1	15/26	3.86E8
	93.34 - 93.97	-.GANFIAVHEM*LDGFR.-	1693.91	2	3.62	0.38	734.6	1	16/28	2.68E9
	70.50 - 71.05	-.KAIGEAKDDDTADILTAASR.-	2062.23	3	4.55	0.31	1482.9	1	34/76	2.76E9
	71.30	-.KAIGEAKDDDTADILTAASR.-	2062.23	2	5.96	0.54	1898.7	1	24/38	2.89E9
	13.24 - 13.81	-.KATVELLNR.-	1044.23	1	1.88	0.17	306.5	1	11/16	4.48E8
	13.10 - 14.01	-.KATVELLNR.-	1044.23	2	3.12	0.32	1393.3	1	15/16	8.71E8
	130.44 - 131.03	-.QVIQFIDLSLITK.-	1518.82	2	4.29	0.49	1373.5	1	20/24	3.02E9
	80.89 - 82.13	-.SYPLDIHNVQDHLK.-	1679.86	2	4.41	0.44	1009.1	1	19/26	6.59E9
	81.94	-.SYPLDIHNVQDHLK.-	1679.86	1	2.39	0.20	319.3	1	12/26	1.03E9
	69.28 - 70.37	-.TALIDHLDTM*AER.-	1502.68	2	3.76	0.50	1139.3	1	18/24	4.56E9
	85.96	-.TALIDHLDTMAER.-	1486.68	2	3.22	0.56	902.3	1	16/24	1.32E9
	51.56 - 52.86	-.YAIVANDVR.-	1021.15	1	2.16	0.23	238.9	1	12/16	1.01E9
	51.83 - 53.02	-.YAIVANDVR.-	1021.15	2	3.27	0.29	1393.8	1	16/16	7.13E8
#8	Q8X9Q6 (Q8X9Q6) Pu					180.24			18 (18 0 0 0 0)	2.00
	117.74 - 118.26	-.ESSALSQLHFGLGWDPVK.-	1972.19	2	3.64	0.50	456.2	1	14/34	2.72E9
	137.53 - 137.89	-.GLLGGLFGGNDSIDLDAGCVLM*DSTGK.-	2699.97	2	4.07	0.54	891.1	1	20/52	1.96E9
	139.60 - 140.20	-.GLLGGLFGGNDSIDLDAGCVLM*DSTGK.-	2699.97	2	2.69	0.52	587.7	1	17/52	1.71E9
	90.00 - 91.27	-.GQSFNDVENAFCR.-	1544.60	2	2.57	0.52	622.1	1	12/24	2.54E9
	88.10	-.GQSFNDVENAFCR.-	1544.60	1	2.24	0.42	226.5	1	12/24	7.34E8
	87.78 - 88.45	-.GQSFNDVENAFCR.-	1544.60	2	3.53	0.67	798.9	1	14/24	3.01E9
	129.78 - 131.15	-.LPANVEYLAFTVNSFR.-	1842.09	2	4.79	0.55	1430.1	1	20/30	5.17E9
	130.16	-.LPANVEYLAFTVNSFR.-	1842.09	3	3.88	0.58	1405.9	1	29/60	1.28E9
	133.08	-.LPANVEYLAFTVNSFR.-	1842.09	2	3.88	0.56	1205.6	1	18/30	1.68E9
	74.19 - 75.02	-.LTEQGSHTGIVISSLR.-	1698.90	2	3.99	0.59	1312.1	1	19/30	3.80E9
	89.67 - 91.04	-.NNGNWDFTALGHACR.-	1733.82	2	3.60	0.56	1162.7	1	19/28	2.69E9
	70.73 - 71.29	-.STCGAVVHSGDNLTGEGDGDDEVIK.-	2533.60	2	2.88	0.49	201.9	17	12/48	3.57E9
	70.61 - 71.75	-.STCGAVVHSGDNLTGEGDGDDEVIK.-	2533.60	3	4.75	0.63	1043.2	1	29/96	3.88E9
	85.28 - 85.86	-.STCGAVVHSGDNLTGEGDGDDEVIKVNLSR.	3103.25	3	4.49	0.51	176.7	85	23/116	2.18E9
	83.50 - 84.70	-.TIDDM*HSDIVSAVIR.-	1688.89	2	3.29	0.46	840.4	1	19/28	3.00E9
	84.09	-.TIDDM*HSDIVSAVIR.-	1688.89	3	3.27	0.31	714.7	1	28/56	1.53E9
	95.78 - 96.78	-.TIDDMHSDIVSAVIR.-	1672.89	2	4.00	0.56	1118.0	1	19/28	1.73E9

	102.33	-.TIDTIWFR.-	1052.21	2	2.87	0.39	705.4	1	13/14		1.08E9
#9	TPX_ECOLI (P37901)					160.30			16 (16 0 0 0 0)		4.59
	97.44	-.DLSDVTLGQFAGK.-	1351.49	1	3.01	0.57	541.8	1	14/24		3.36E9
	97.13 - 97.75	-.DLSDVTLGQFAGK.-	1351.49	2	4.56	0.63	1675.5	1	21/24		7.95E9
	119.79 - 120.39	-.FCGAEGLNNVITLSTFR.-	1900.12	2	5.47	0.71	2278.9	1	22/32		8.26E9
	118.20 - 119.38	-.FCGAEGLNNVITLSTFR.-	1900.12	2	4.85	0.65	1656.6	1	22/32		1.05E10
	122.96 - 124.22	-.FCGAEGLNNVITLSTFR.-	1900.12	2	4.91	0.56	1223.1	1	18/32		2.45E9
	106.78 - 107.31	-.KVLNIFPSIDTGVCAASVR.-	2048.37	3	3.99	0.49	1736.1	1	32/72		2.78E9
	106.84	-.KVLNIFPSIDTGVCAASVR.-	2048.37	2	4.20	0.53	559.5	1	20/36		3.08E9
	129.07	-.NAEFLQAYGVAIADGPLK.-	1878.12	2	4.76	0.54	1546.8	1	21/34		2.17E9
	118.65 - 119.94	-.NAEFLQAYGVAIADGPLK.-	1878.12	2	3.56	0.63	816.3	1	16/34		1.47E10
	120.46 - 121.65	-.NAEFLQAYGVAIADGPLK.-	1878.12	2	5.62	0.59	2088.4	1	23/34		1.15E10
	83.09 - 83.62	-.SQTVHFQGNPVTVANSIPQAGSK.-	2368.59	3	4.41	0.39	914.2	1	31/88		5.93E9
	79.11 - 79.53	-.SQTVHFQGNPVTVANSIPQAGSK.-	2368.59	3	3.57	0.27	1217.6	1	32/88		3.18E9
	83.16 - 83.73	-.SQTVHFQGNPVTVANSIPQAGSK.-	2368.59	2	6.02	0.62	1621.5	1	26/44		7.05E9
	78.64 - 79.25	-.SQTVHFQGNPVTVANSIPQAGSK.-	2368.59	2	3.04	0.43	722.6	1	19/44		2.87E9
	116.14 - 117.25	-.VLNIFPSIDTGVCAASVR.-	1920.19	2	4.75	0.49	383.8	1	18/34		1.41E10
	116.44	-.VLNIFPSIDTGVCAASVR.-	1920.19	3	4.77	0.62	1279.3	1	30/68		1.80E9
#10	OMPC_ECO57 (Q8XE					160.22			16 (16 0 0 0 0)		1.21
	13.12	-.DGNKLDLYGK.-	1123.24	2	2.65	0.40	1112.5	1	14/18	3	8.61E8
	93.69 - 93.76	-.INLLDDNQFTR.-	1349.48	1	2.10	0.26	390.0	1	12/20		1.58E9
	93.59 - 94.17	-.INLLDDNQFTR.-	1349.48	2	3.28	0.43	1087.6	1	17/20		4.12E9
	62.17	-.NMSTYVDYK.-	1121.25	1	2.12	0.38	301.8	1	10/16	3	3.61E8
	67.30	-.NYDDEDILK.-	1125.17	1	3.06	0.32	418.3	1	12/16		1.32E9
	66.89 - 67.47	-.NYDDEDILK.-	1125.17	1	2.69	0.09	387.3	1	12/16		2.77E9
	60.33 - 61.60	-.RTDDQNSPLYIGNGDR.-	1821.89	2	3.54	0.46	576.3	1	18/30		8.38E8
	82.86	-.RTDDQNSPLYIGNGDRAETYTGGLK.-	2742.90	3	3.95	0.55	1713.7	1	36/96		1.91E9
	87.48	-.RTDDQNSPLYIGNGDRAETYTGGLK.-	2742.90	2	3.25	0.36	360.3	1	17/48		7.79E8
	91.64	-.TDDQNSPLYIGNGDRAETYTGGLK.-	2586.71	2	3.85	0.56	386.7	1	18/46		1.51E9
	91.62	-.TDDQNSPLYIGNGDRAETYTGGLK.-	2586.71	3	3.15	0.45	629.1	1	27/92		2.37E9
	87.84 - 88.50	-.TDDQNSPLYIGNGDRAETYTGGLK.-	2586.71	2	2.85	0.51	231.8	1	15/46		1.81E9
	77.20	-.VDGLHYFSDDKSVDGDQTYM*R.-	2465.60	2	4.06	0.56	1066.0	1	21/40		1.45E9
	76.52 - 77.10	-.VDGLHYFSDDKSVDGDQTYM*R.-	2465.60	3	4.36	0.47	1462.6	1	30/80		1.62E9
	54.53 - 55.17	-.VGSLGWANK.-	932.06	2	2.74	0.32	910.0	1	13/16		8.27E8
	88.08 - 89.21	-.YVDVGATYYFNK.-	1440.58	2	3.22	0.38	1611.5	1	19/22		2.71E9
#11	RS4_ECOLI (P02354)					150.29			15 (15 0 0 0 0)		1.29
	38.21 - 39.89	-.AALELAEQR.-	1001.12	2	2.77	0.28	851.6	1	13/16		5.54E8
	34.47 - 35.96	-.AALELAEQR.-	1001.12	2	2.60	0.30	697.6	1	11/16		4.15E8
	35.16 - 40.14	-.AALELAEQR.-	1001.12	1	2.61	0.39	321.0	3	10/16		6.20E8
	40.42 - 41.03	-.AALELAEQR.-	1001.12	2	2.67	0.31	713.6	1	12/16		3.72E8
	74.30	-.EKPTWLEVDAGK.-	1373.54	1	2.46	0.18	173.4	1	11/22		1.04E9
	81.80	-.EKPTWLEVDAGKM*EGTFK.-	2083.35	2	3.00	0.55	649.7	1	15/34		1.50E9
	127.85	-.GNTGENLLALLEGR.-	1457.62	2	3.84	0.45	853.1	1	16/26		2.34E9
	119.19 - 119.77	-.LKGNTGENLLALLEGR.-	1698.95	2	4.40	0.59	950.4	1	19/30		3.09E9
	119.14	-.LKGNTGENLLALLEGR.-	1698.95	3	3.67	0.25	829.4	6	23/60		1.39E9
	69.81	-.LSDYGVQLR.-	1051.18	2	3.16	0.24	1381.4	1	15/16		1.46E9
	62.40 - 63.52	-.REGTDLFLK.-	1079.23	2	2.72	0.18	600.9	1	12/16		1.17E9
	32.88 - 34.29	-.RIYGVLER.-	1006.18	2	2.78	0.31	1145.1	1	14/14		5.81E8
	30.77 - 32.37	-.RIYGVLER.-	1006.18	2	3.04	0.33	1120.6	1	14/14		8.74E8
	103.23	-.VVNIASYQVSPNDVVSIR.-	1961.21	3	5.04	0.64	1977.3	1	32/68		1.86E9
	102.98 - 103.56	-.VVNIASYQVSPNDVVSIR.-	1961.21	2	5.90	0.56	912.5	1	20/34		1.14E10
#12	EFTU_ECOLI (P02990					150.23			15 (15 0 0 0 0)		1.67
	50.71 - 52.17	-.AFDQIDNAPEEK.-	1377.44	2	2.86	0.13	805.4	2	15/22		1.28E9
	52.75 - 53.37	-.AFDQIDNAPEEK.-	1377.44	2	3.46	0.39	615.4	1	13/22		1.21E9
	141.17 - 141.41	-.AIDKPFLLPIEDVFSISGR.-	2118.46	2	3.66	0.50	601.1	1	18/36		2.38E9
	59.81 - 60.98	-.ALEGDAEWEAK.-	1219.28	2	2.79	0.51	940.2	1	16/20		6.79E8
	106.86 - 108.10	-.ELLSQYDFPGDDTPIVR.-	1966.14	2	4.68	0.61	979.7	1	21/32		9.57E9
	87.23	-.FESEVYILSK.-	1215.38	2	3.39	0.52	879.8	1	16/18		1.07E9
	71.63 - 72.21	-.GITINTSHVEYDTPTR.-	1804.94	2	4.55	0.68	792.1	1	18/30		3.41E9
	29.69	-.GQVLAKPGTIKPHTK.-	1575.88	2	2.82	0.58	511.8	1	16/28		2.66E8
	142.49 - 143.28	-.ILELAGFLDSYIPEPER.-	1963.22	2	4.29	0.48	677.1	1	18/32		4.59E9
	88.94 - 89.37	-.M*VVTLIHPIAM*DDGLR.-	1814.16	3	3.45	0.50	668.1	1	25/60		1.62E9
	88.90 - 89.54	-.M*VVTLIHPIAM*DDGLR.-	1814.16	2	3.73	0.47	650.7	1	17/30		2.32E9
	149.24	-.PFLLPIEDVFSISGR.-	1690.96	2	2.83	0.53	548.2	1	17/28		5.89E8
	115.27 - 116.12	-.TTLTAAITTVLAK.-	1304.56	2	3.73	0.53	1300.3	1	19/24		2.99E9
	81.63 - 82.61	-.VGEEVEIVGIK.-	1172.35	2	3.41	0.37	1662.4	1	17/20		3.60E9
	73.77	-.VGEEVEIVGIKETQK.-	1658.88	2	3.88	0.60	1141.6	1	18/28		1.33E9
#13	DCEA_ECO57 (P5822					130.30			13 (13 0 0 0 0)		1.13
	142.11 - 143.04	-.GFEM*DFAELLLEDYK.-	1837.04	2	4.04	0.56	1289.6	1	17/28	1	1.92E9
	127.70 - 128.29	-.GWQVPAFTLGGEATDIVVM*R.-	2164.47	2	5.94	0.72	2249.3	1	30/38	1	2.35E9
	114.51 - 115.04	-.LGPYEFICTGRPDEGIPAVCFK.-	2527.84	3	5.15	0.57	1375.6	1	31/84	1	3.05E9
	114.81	-.LGPYEFICTGRPDEGIPAVCFK.-	2527.84	2	2.65	0.42	298.6	1	13/42	1	1.96E9
	89.69	-.LKEGEDPGYTLYDLSER.-	1986.13	2	3.35	0.52	524.2	1	19/32		1.39E9
	52.01 - 52.64	-.LQGIAQQNSFK.-	1234.39	2	3.35	0.57	693.8	1	15/20	1	1.05E9

	51.95 - 52.53	-.LQGIAQQNSFK.-	1234.39	1	1.88	0.28	378.8	1	12/20	1	5.05E8
	93.84	-.NWIDKEEYPQSAAIDLR.-	2049.23	2	4.50	0.57	859.7	1	19/32	1	1.63E9
	117.02	-.PAGQVIAQYYEFLR.-	1655.88	2	4.90	0.58	1836.5	1	21/26	1	2.89E9
	130.63	-.RGFEM*DFAELLLEDYK.-	1993.23	3	3.46	0.26	899.0	1	23/60	1	9.42E8
	123.29 - 124.71	-.VQNASYQVAAYLADEIAK.-	1955.16	2	3.78	0.57	1087.0	1	18/34	1	3.23E9
	121.33	-.VQNASYQVAAYLADEIAK.-	1955.16	3	3.07	0.50	1758.9	1	30/68	1	9.53E8
	119.73 - 121.06	-.VQNASYQVAAYLADEIAK.-	1955.16	2	5.10	0.62	2154.0	1	24/34	1	3.23E9
#14	G3P1_ECOLI (P06977					130.25			13 (13 0 0 0 0)		1.93
	83.98 - 85.11	-.AGIALNDNFVK.-	1162.32	2	3.27	0.47	999.7	1	17/20		3.93E9
	84.02 - 85.26	-.AGIALNDNFVK.-	1162.32	1	2.24	0.24	498.3	1	13/20		2.18E9
	98.81	-.GANFDKYAGQDIVSNASCTTNCLAPLAK.-	2988.24	3	5.08	0.53	1145.0	1	34/108		1.89E9
	18.63 - 20.06	-.GASQNIIPSSTGAAK.-	1402.54	2	2.82	0.28	466.9	1	16/28		2.62E8
	81.47	-.LVSWYDNETGYSNK.-	1676.77	1	2.13	0.52	151.6	3	9/26		1.39E9
	81.22 - 81.44	-.LVSWYDNETGYSNK.-	1676.77	2	3.82	0.68	930.8	1	17/26		6.13E9
	116.88 - 118.05	-.VINDNFGIIEGLM*TTVHATTATQK.-	2591.92	3	3.47	0.53	350.8	1	25/92		7.48E9
	119.88 - 120.52	-.VINDNFGIIEGLM*TTVHATTATQK.-	2591.92	3	3.49	0.55	480.3	1	26/92		3.41E9
	135.38 - 135.98	-.VINDNFGIIEGLMTTVHATTATQK.-	2575.92	3	4.35	0.59	677.4	1	32/92		2.09E9
	86.03	-.VLDLIAHISK.-	1109.34	2	3.23	0.52	1062.0	1	16/18		1.44E9
	86.17	-.VLDLIAHISK.-	1109.34	1	1.92	0.38	504.5	1	13/18		1.31E9
	97.57 - 98.17	-.VPTPNVSVVDLTVR.-	1496.73	2	3.68	0.59	1220.8	1	17/26		9.86E9
	97.98	-.VPTPNVSVVDLTVR.-	1496.73	1	2.32	0.29	411.5	1	15/26		1.33E9
#15	IPYR_ECOLI (P17288)					120.19			12 (12 0 0 0 0)		1.03
	74.68 - 76.04	-.AQIAHFFEHYK.-	1391.56	2	2.74	0.41	1449.8	1	15/20		2.27E9
	84.99 - 86.32	-.AQIAHFFEHYKDLEK.-	1877.09	2	3.54	0.56	619.6	1	16/28		4.71E9
	85.05 - 85.63	-.AQIAHFFEHYKDLEK.-	1877.09	3	3.79	0.45	602.6	1	22/56		2.43E9
	97.28 - 97.88	-.EYDHIKDVNDLPELLK.-	1942.16	3	3.19	0.41	869.5	1	25/60		2.86E9
	94.65	-.EYDHIKDVNDLPELLK.-	1942.16	2	3.56	0.58	1144.8	1	17/30		1.68E9
	97.21 - 97.77	-.EYDHIKDVNDLPELLK.-	1942.16	2	3.65	0.45	925.3	1	17/30		3.69E9
	63.32 - 64.45	-.SLLNVPAGK.-	899.07	1	1.91	0.40	372.7	1	10/16		2.51E9
	63.63	-.SLLNVPAGK.-	899.07	1	2.20	0.26	508.3	1	12/16		1.22E9
	15.94 - 17.75	-.VEGWENAEAAK.-	1204.27	2	2.87	0.46	667.4	1	16/20		4.31E8
	18.34 - 20.18	-.VEGWENAEAAK.-	1204.27	2	2.76	0.48	555.0	1	15/20		2.36E8
	21.47 - 22.85	-.VEGWENAEAAK.-	1204.27	2	2.57	0.43	636.1	1	16/20		1.65E8
	14.18 - 15.38	-.VEGWENAEAAK.-	1204.27	2	2.69	0.48	695.1	1	16/20		5.94E8
#16	IF3_ECOLI (P02999) T					110.32			11 (11 0 0 0 0)		1.71
	96.36 - 97.69	-.AEEAGVDLVEISPNAEPPVCR.-	2253.45	2	4.32	0.33	664.7	1	17/40		5.07E9
	97.94	-.AEEAGVDLVEISPNAEPPVCR.-	2253.45	2	3.43	0.47	618.6	1	16/40		4.02E9
	106.14	-.EALEKAEEAGVDLVEISPNAEPPVCR.-	2824.08	3	4.99	0.45	1716.5	1	41/100		3.86E9
	12.21 - 13.14	-.EM*AHQQIGM*EVLNR.-	1688.91	2	3.12	0.39	371.5	1	13/26		1.26E9
	69.76	-.EM*AHQQIGMEVLNR.-	1672.91	2	3.15	0.42	440.0	1	13/26		1.39E9
	59.67 - 60.29	-.EMAHQQIGM*EVLNR.-	1672.91	2	3.03	0.31	344.1	3	12/26		4.57E8
	106.72 - 108.01	-.LTGLEGEQLGIVSLR.-	1585.83	2	4.64	0.43	1447.6	1	19/28		9.66E9
	107.07	-.LTGLEGEQLGIVSLR.-	1585.83	1	2.70	0.50	140.8	1	11/28		1.32E9
	103.70 - 104.30	-.VKDDLQELAVVESFPTK.-	1919.17	3	4.50	0.52	2076.4	1	31/64		3.32E9
	103.68 - 104.27	-.VKDDLQELAVVESFPTK.-	1919.17	2	5.83	0.61	3561.1	1	26/32		4.86E9
	110.71 - 111.41	-.VKDDLQELAVVESFPTKIEGR.-	2374.68	3	6.31	0.68	1675.7	1	38/80		2.77E9
#17	RL4_ECO57 (P60725)					100.32			10 (10 0 0 0 0)		2.83
	86.67 - 87.19	-.DAQSALTVSETTFGR.-	1583.68	1	3.03	0.55	133.3	1	12/28		5.52E9
	86.81	-.DAQSALTVSETTFGR.-	1583.68	1	2.34	0.41	112.7	3	11/28		3.01E9
	85.82 - 86.96	-.DAQSALTVSETTFGR.-	1583.68	2	5.15	0.68	1520.3	1	20/28		9.54E9
	120.35 - 121.38	-.DATGIDPVSLIAFDK.-	1562.75	2	5.05	0.66	964.5	1	21/28		1.96E10
	120.41 - 120.94	-.DATGIDPVSLIAFDK.-	1562.75	1	3.50	0.57	546.2	1	17/28		4.27E9
	119.44 - 120.14	-.DFNEALVHQVVVAYAAGAR.-	2031.26	3	4.27	0.60	1372.3	1	32/72		6.24E9
	119.42 - 120.00	-.DFNEALVHQVVVAYAAGAR.-	2031.26	2	6.38	0.48	1305.4	1	23/36		9.19E9
	127.46	-.DFNEALVHQVVVAYAAGAR.-	2031.26	3	4.13	0.43	1676.4	1	32/72		9.39E8
	127.48 - 128.22	-.DFNEALVHQVVVAYAAGAR.-	2031.26	2	2.72	0.41	196.3	13	11/36		1.96E9
	102.93 - 103.46	-.SILSELVR.-	917.09	2	2.76	0.31	686.8	1	13/14		2.42E9
#18	ENO_ECOLI (P08324)					100.25			10 (10 0 0 0 0)		0.89
	141.65 - 142.29	-.AFTSEEFTHFLEELTK.-	1930.10	2	4.60	0.56	1484.9	1	19/30		3.02E9
	141.72	-.AFTSEEFTHFLEELTK.-	1930.10	3	3.06	0.52	681.3	1	26/60		1.57E9
	31.87 - 32.49	-.DAGYTAVISHR.-	1190.29	2	2.92	0.53	1473.3	1	16/20		3.09E8
	29.81 - 31.00	-.DAGYTAVISHR.-	1190.29	2	3.22	0.64	979.6	1	15/20		8.66E8
	27.68 - 29.27	-.DAGYTAVISHR.-	1190.29	2	3.37	0.46	1564.7	1	17/20		8.51E8
	118.63 - 119.62	-.FNQIGSLTETLAAIK.-	1606.85	2	5.04	0.38	1416.9	1	24/28		4.96E9
	131.26	-.GM*NTAVGDEGGYAPNLGSNAEALAVIAEAVI	3007.28	3	3.67	0.46	640.6	3	29/120		1.46E9
	84.55 - 84.57	-.GM*PLYEHIAELNGTPGK.-	1844.08	2	3.89	0.55	1151.8	1	19/32		2.00E9
	106.27	-.IQLVGDDLFVTNTK.-	1563.78	2	3.98	0.47	1223.2	1	21/26		3.18E9
	105.00	-.SGETEDATIADLAVGTAAGQIK.-	2119.27	2	3.53	0.41	633.6	1	17/42		1.61E9
#19	GLNH_ECOLI (P10344					100.24			10 (10 0 0 0 0)		1.15
	120.20	-.ADAVLHDTPNILYFIK.-	1831.10	3	4.53	0.46	1890.8	1	31/60		1.56E9
	99.26 - 99.84	-.AVGDSLEAQQYGIAFPK.-	1794.99	2	4.70	0.60	1551.5	1	20/32		6.35E9
	99.65	-.AVGDSLEAQQYGIAFPK.-	1794.99	1	1.84	0.34	74.4	262	7/32		8.47E8
	97.51 - 98.13	-.AVGDSLEAQQYGIAFPK.-	1794.99	2	3.04	0.35	266.4	7	13/32		3.50E9
	123.06 - 123.64	-.ELKLDYELKPM*DFSGIIPALQTK.-	2667.11	3	3.74	0.56	805.1	1	24/88		1.61E9

	122.59 - 123.95	-.LVVATDTAFVPFEFK.-	1684.96	2	4.36	0.59	999.5	1	18/28	5.06E9
	109.86	-.NVDLALAGITITDER.-	1601.78	1	2.78	0.42	203.4	1	14/28	8.77E8
	102.36	-.QFPNIDNAYM*ELGTNR.-	1900.06	2	3.27	0.52	502.1	2	14/30	3.60E9
	107.81	-.QFPNIDNAYMELGTNR.-	1884.06	2	2.61	0.37	338.8	1	12/30	1.80E9
	64.18	-.SGLLVM*VK.-	863.10	2	2.60	0.27	672.3	2	12/14	3.48E8
#20	Q8X8I5 (Q8X8I5) 3-oxo					90.27			9 (9 0 0 0 0)	1.16
	112.35	-.AEFGEVDILVNNAGITR.-	1819.01	2	4.01	0.48	893.6	1	17/32	3.10E9
	75.69	-.AGILVQVPAGR.-	1081.29	1	2.03	0.34	396.5	1	13/20	1.21E9
	11.46 - 12.71	-.AIAETLAAR.-	916.06	2	2.55	0.40	772.3	1	14/16	1.46E9
	104.81 - 106.02	-.GITVNVVAPGFIETDM*TR.-	1937.21	2	3.41	0.58	421.9	1	16/34	3.87E9
	122.34 - 122.92	-.GLM*LNVTDPASIESVLEK.-	1933.21	2	5.44	0.61	1560.6	1	25/34	4.04E9
	128.14 - 128.82	-.GLMLNVTDPASIESVLEK.-	1917.21	2	2.54	0.44	247.6	2	12/34	1.51E9
	92.90	-.IITIGSVVGTM*GNGGQANYAAAK.-	2210.50	2	5.39	0.72	713.1	1	22/44	2.29E9
	99.88 - 101.17	-.VIGTATSENGAQAISDYLGANGK.-	2238.40	2	5.14	0.67	1294.8	1	24/44	4.56E9
	98.15 - 99.34	-.VIGTATSENGAQAISDYLGANGK.-	2238.40	2	5.39	0.65	1826.3	1	27/44	3.60E9
#21	LUXS_ECO57 (Q8X90					90.20			9 (9 0 0 0 0)	1.04
	105.61	-.GIHTLEHLFAGFM*R.-	1645.91	2	4.07	0.68	1059.7	1	18/26	1.90E9
	74.46	-.INSNEELALPK.-	1228.38	2	3.60	0.45	1841.0	1	18/20	1.58E9
	70.33 - 71.54	-.INSNEELALPK.-	1228.38	2	3.20	0.33	994.2	1	17/20	3.49E9
	83.93	-.PLLDSFTVDHTR.-	1401.55	3	3.62	0.47	1183.3	1	27/44	6.59E8
	84.00 - 85.07	-.PLLDSFTVDHTR.-	1401.55	2	3.15	0.44	1601.4	1	17/22	2.30E9
	93.36 - 94.77	-.TGFYM*SLIGTPDEQR.-	1731.91	2	3.32	0.37	752.4	1	18/28	7.26E9
	92.20 - 93.26	-.TM*NTPHGDAITVFDLR.-	1805.01	2	4.09	0.55	1637.1	1	23/30	2.83E9
	92.29 - 92.94	-.TM*NTPHGDAITVFDLR.-	1805.01	3	4.10	0.52	1503.6	1	28/60	1.87E9
	97.11	-.TMNTPHGDAITVFDLR.-	1789.01	2	2.84	0.62	556.3	1	14/30	1.27E9
#22	CRP_ECOLI (P03020)					90.19			9 (9 0 0 0 0)	0.46
	88.60	-.IAQTLLNLAK.-	1085.32	1	1.96	0.20	482.5	1	11/18	1.84E9
	88.68 - 88.71	-.IAQTLLNLAK.-	1085.32	2	3.83	0.30	1290.2	1	16/18	1.28E9
	45.55 - 46.16	-.M*LEDQNLISAHGK.-	1472.65	2	3.02	0.35	1295.6	1	17/24	5.10E8
	43.73 - 44.92	-.M*LEDQNLISAHGK.-	1472.65	2	3.25	0.34	1195.1	1	17/24	5.11E8
	41.36 - 43.14	-.M*LEDQNLISAHGK.-	1472.65	2	3.86	0.50	1819.5	1	20/24	4.39E8
	57.60 - 58.43	-.QEIGQIVGCSR.-	1247.38	2	2.83	0.48	539.9	1	15/20	5.83E8
	62.32	-.TACEVAEISYK.-	1271.39	2	2.57	0.51	932.3	1	15/20	6.55E8
	19.90	-.TIVVYGTR.-	909.07	2	2.62	0.54	436.9	2	10/14	1.80E8
	101.90 - 103.21	-.VGNLAFLDVTGR.-	1262.44	2	3.33	0.58	914.2	1	16/22	4.30E9
#23	DSBA_ECOLI (P24991					80.32			8 (8 0 0 0 0)	0.82
	129.41 - 130.01	-.DLTQAWAVAM*ALGVEDK.-	1835.07	2	2.61	0.36	430.6	1	13/32	2.82E9
	149.62 - 150.85	-.DLTQAWAVAM*ALGVEDKVTVPLFEGVQK.-	3033.49	3	4.49	0.57	917.2	1	34/108	2.43E9
	99.73	-.SASDIRDVFINAGIK.-	1606.81	2	3.95	0.32	1062.5	1	17/28	2.01E9
	132.36	-.SASDIRDVFINAGIKGEEYDAAWNSFVVK.-	3203.51	3	5.34	0.60	1457.5	1	35/112	1.20E9
	68.07 - 68.64	-.YHVNFM*GGDLGK.-	1354.52	2	3.26	0.43	538.0	1	13/22	1.57E9
	98.61 - 99.17	-.YQLNPQGM*DTSNM*DVFVQQYADTVK.-	2926.19	2	4.32	0.51	592.0	1	17/48	3.28E9
	98.59 - 99.15	-.YQLNPQGM*DTSNM*DVFVQQYADTVK.-	2926.19	3	6.47	0.71	1335.5	1	33/96	3.48E9
	114.58	-.YQLNPQGM*DTSNMDVFVQQYADTVK.-	2910.19	2	4.18	0.39	630.8	1	19/48	1.43E9
#24	RRF_ECOLI (P16174)					80.31			8 (8 0 0 0 0)	1.52
	86.38 - 87.00	-.AIM*ASDLGLNPNSAGSDIR.-	1919.11	2	5.66	0.47	1917.2	1	26/36	4.85E9
	84.47 - 85.34	-.AIM*ASDLGLNPNSAGSDIR.-	1919.11	2	4.55	0.57	1309.0	1	26/36	3.17E9
	115.22 - 115.73	-.AIM*ASDLGLNPNSAGSDIRVPLPPLTEER.-	3051.42	3	6.15	0.63	1730.3	1	42/112	4.88E9
	116.80	-.AIM*ASDLGLNPNSAGSDIRVPLPPLTEER.-	3051.42	3	3.04	0.42	772.4	1	28/112	4.08E9
	118.69	-.ASPSLLDGIVVEYYGTPTPLR.-	2249.55	2	4.70	0.55	1736.1	1	24/40	3.35E9
	129.48 - 130.48	-.ASPSLLDGIVVEYYGTPTPLR.-	2249.55	2	5.78	0.53	2637.8	1	25/40	7.49E9
	96.47	-.IEAALADKEAELM*QF.-	1695.92	2	2.63	0.47	943.1	1	16/28	2.30E9
	92.24 - 92.78	-.KIEAALADKEAELM*QF.-	1824.09	2	4.77	0.51	1608.3	1	20/30	3.51E9
#25	YAJQ_ECOLI (P77482					80.31			8 (8 0 0 0 0)	1.02
	85.92 - 86.51	-.GGDLGQPFQFK.-	1194.32	2	2.81	0.35	572.5	1	14/20	2.18E9
	85.94 - 86.07	-.GGDLGQPFQFK.-	1194.32	1	1.82	0.29	407.0	2	11/20	1.47E9
	85.80	-.GIEGSSLDVPENIVHSGK.-	1839.00	2	3.95	0.58	313.9	1	14/34	3.91E9
	84.11 - 85.49	-.GIEGSSLDVPENIVHSGK.-	1839.00	2	3.90	0.69	435.7	1	17/34	5.47E9
	64.88 - 65.06	-.LKVQAQIQGDEIR.-	1498.71	2	4.09	0.57	1477.9	1	19/24	6.74E8
	143.43 - 144.06	-.VLSESDFQVNQLLDILR.-	1990.25	2	5.32	0.49	2234.7	1	24/32	6.46E9
	143.55	-.VLSESDFQVNQLLDILR.-	1990.25	3	6.10	0.38	3189.5	1	37/64	1.08E9
	11.84	-.VQAQIQGDEIR.-	1257.38	2	3.19	0.48	942.2	1	15/20	1.43E9
#26	PGK_ECO57 (Q8XD03					80.27			8 (8 0 0 0 0)	0.96
	146.20 - 147.39	-.ADEQILDIGDASAQELAEILK.-	2243.45	2	5.30	0.62	1430.6	1	26/40	3.28E9
	130.60 - 131.74	-.ISYISTGGGAFLEFVEGK.-	1876.10	2	3.44	0.01	959.9	1	17/34	2.74E9
	89.08 - 90.47	-.LLTTCNIPVPSDVR.-	1585.82	2	2.84	0.36	508.3	1	14/26	3.83E9
	76.69	-.SLYEADLVDEAKR.-	1509.64	2	2.51	0.47	1003.5	1	15/24	1.69E9
	144.96	-.TILWNGPVGVFEFPNFR.-	1994.28	2	3.78	0.62	1458.1	1	20/32	2.17E9
	147.41	-.TILWNGPVGVFEFPNFR.-	1994.28	2	2.84	0.39	598.7	1	16/32	1.21E9
	73.58 - 74.15	-.VATEFSETAPATLK.-	1465.63	2	2.95	0.60	709.0	1	15/26	2.52E9
	124.18 - 125.47	-.VM*VTSHLGRPTEGEYNEEFSLLPVVNYLK.-	3339.76	3	3.50	0.42	835.3	1	28/112	3.73E9
#27	DEOD_ECOLI (P09743					80.25			8 (8 0 0 0 0)	0.56
	78.92	-.ALTICTVSDHIR.-	1386.57	2	2.86	0.50	827.7	1	15/22	1.52E9
	97.05	-.FKDHDFAAIADFDM*VR.-	1915.12	2	5.03	0.70	1767.1	1	19/30	1.93E9

	96.96	-.FKDHDFAAIADFDM*VR.-	1915.12	3	4.08	0.52	982.8	1	26/60	1.15E9
	106.48	-.IALESVLLGDKE.-	1287.49	2	3.89	0.60	2237.8	1	19/22	2.92E9
	71.34	-.ISVM*GHGM*GIPSCSIYTK.-	1971.28	2	2.85	0.46	188.2	1	14/34	9.66E8
	12.12	-.LRDVVIGM*GACTDSK.-	1638.86	2	2.83	0.45	356.1	2	14/28	1.11E9
	28.18 - 29.95	-.VGSCGAVLPHVK.-	1224.43	2	2.71	0.34	1050.0	1	15/22	4.08E8
	98.23	-.YIAETFLEDAR.-	1328.45	2	3.20	0.43	972.0	1	16/20	2.33E9
#28	FLAV_ECO57 (P61951					80.25			8 (8 0 0 0 0)	0.90
	104.34 - 105.67	-.AITGIFFGSDTGNTENIAK.-	1957.13	2	4.93	0.67	1402.2	1	22/36	5.01E9
	108.80 - 109.75	-.AITGIFFGSDTGNTENIAK.-	1957.13	2	4.57	0.60	1090.6	1	19/36	3.14E9
	107.03	-.AITGIFFGSDTGNTENIAK.-	1957.13	2	2.61	0.52	696.6	1	16/36	2.36E9
	88.27	-.GATIVGHWPTAGYHFEASK.-	2030.23	2	4.12	0.61	744.5	1	19/36	1.69E9
	88.19 - 89.19	-.GATIVGHWPTAGYHFEASK.-	2030.23	3	3.14	0.47	1415.7	1	31/72	2.15E9
	101.15	-.GLADDDHFVGLAIDEDRQPELTAER.-	2783.95	2	3.54	0.54	555.9	1	17/48	1.54E9
	147.49 - 148.16	-.LVALFGCGDQEDYAEYFCDALGTIR.-	2885.12	2	3.24	0.54	864.0	1	19/48	1.44E9
	147.47 - 148.09	-.LVALFGCGDQEDYAEYFCDALGTIR.-	2885.12	3	4.45	0.55	981.0	2	27/96	2.60E9
#29	SODM_ECO57 (P6682					80.24			8 (8 0 0 0 0)	0.88
	95.54	-.DFGSVDNFKAEFEK.-	1633.74	2	4.01	0.61	1670.2	1	20/26	2.85E9
	118.73 - 119.46	-.EFWNVVNWDEAAAR.-	1707.83	2	4.83	0.63	1260.4	1	21/26	7.08E9
	131.63 - 131.95	-.FGSGWAWLVLK.-	1264.50	2	4.19	0.45	1814.9	1	18/20	1.32E9
	139.06 - 140.47	-.HHQTYVNNANAALESLPEFANLPVEELITK.-	3364.71	3	4.29	0.50	562.2	2	31/116	3.28E9
	132.33	-.HHQTYVNNANAALESLPEFANLPVEELITK.-	3364.71	3	3.07	0.38	632.9	1	31/116	1.34E9
	60.27 - 60.90	-.NNAGGHANHSLFWK.-	1553.67	2	4.12	0.56	1467.6	1	19/26	7.10E8
	51.20 - 52.56	-.NNAGGHANHSLFWK.-	1553.67	2	3.55	0.53	1294.2	1	18/26	5.36E8
	123.25 - 124.03	-.SYTLPSLPYAYDALEPHFDK.-	2328.56	2	3.09	0.59	187.6	1	13/38	2.31E9
#30	Q8XB46 (Q8XB46) Hyp					80.24			8 (8 0 0 0 0)	0.59
	78.26	-.LLADDIVPSR.-	1099.26	2	2.76	0.32	965.1	1	15/18	1.38E9
	98.50 - 99.40	-.VETTDGVVQLSGIVDSQAQSDR.-	2305.44	2	4.71	0.63	983.5	1	20/42	2.32E9
	99.98	-.VETTDGVVQLSGIVDSQAQSDRAESIAK.-	2905.12	3	4.03	0.45	1124.2	1	32/108	2.00E9
	64.10	-.VGNFM*DDSAITAK.-	1385.53	2	3.91	0.62	937.0	1	17/24	6.69E8
	60.38 - 61.23	-.VGNFM*DDSAITAK.-	1385.53	2	2.79	0.56	908.9	1	16/24	2.19E9
	58.67 - 60.01	-.VGNFM*DDSAITAK.-	1385.53	2	3.58	0.60	921.3	1	17/24	1.54E9
	204.62	-.VVTLSGFVESQAQAEEAVK.-	1993.20	2	2.80	0.46	345.8	4	11/36	5.09E7
	107.64 - 108.83	-.VVTLSGFVESQAQAEEAVK.-	1993.20	2	4.06	0.50	1107.2	1	19/36	2.86E9
#31	NUSG_ECOLI (P16921					80.23			8 (8 0 0 0 0)	0.81
	97.79 - 97.90	-.ATPVELDFSQVEKA.-	1534.69	1	2.43	0.49	363.8	1	13/26	1.37E9
	115.08 - 115.79	-.LHNM*EDLFGEVM*VPTEEVVEIR.-	2619.95	3	4.47	0.39	959.8	1	30/84	3.76E9
	115.20	-.LHNM*EDLFGEVM*VPTEEVVEIR.-	2619.95	2	2.80	0.42	477.6	1	15/42	1.39E9
	70.10 - 70.44	-.TLFEPGEM*VR.-	1195.37	2	2.81	0.48	372.7	1	12/18	1.84E9
	91.95	-.VMGFIGGTSDRPAPISDKEVDAIM*NR.-	2794.16	3	3.05	0.39	812.5	1	25/100	1.66E9
	122.39 - 123.33	-.VNDGPFADFNGVVEEVDYEK.-	2244.36	2	4.37	0.63	570.7	1	16/38	2.46E9
	125.95 - 127.01	-.VNDGPFADFNGVVEEVDYEK.-	2244.36	2	4.61	0.64	1083.7	1	20/38	1.96E9
	110.69 - 111.81	-.VNDGPFADFNGVVEEVDYEK.-	2244.36	2	4.42	0.68	1339.4	1	22/38	3.55E9
#32	PAL_ECOLI (P07176) I					70.35			7 (7 0 0 0 0)	1.25
	82.79	-.GVSADQISIVSYGK.-	1424.58	1	2.89	0.54	690.5	1	15/26	2.53E9
	82.59 - 82.96	-.GVSADQISIVSYGK.-	1424.58	2	3.70	0.57	1121.3	1	17/26	3.92E9
	112.06 - 112.62	-.LQM*QQLQQNNIVYFDLDKYDIR.-	2803.14	3	7.06	0.64	1392.8	1	33/84	9.23E9
	112.33 - 112.85	-.LQM*QQLQQNNIVYFDLDKYDIR.-	2803.14	2	3.22	0.52	135.3	3	12/42	1.57E9
	119.50	-.LQMQQLQQNNIVYFDLDKYDIR.-	2787.14	3	4.81	0.55	675.9	1	27/84	2.20E9
	100.56 - 101.71	-.SDFAQM*LDAHANFLR.-	1752.93	2	3.35	0.45	708.7	1	17/28	6.44E9
	114.60 - 114.74	-.SDFAQMLDAHANFLR.-	1736.93	2	3.08	0.52	954.0	1	17/28	1.76E9
#33	Q8X5P5 (Q8X5P5) Out					70.31			7 (7 0 0 0 0)	2.39
	110.81 - 111.66	-.NHFVTILGTIQGEQPGFINK.-	2214.51	2	5.41	0.54	1227.5	1	22/38	5.99E9
	119.25	-.NHFVTILGTIQGEQPGFINK.-	2214.51	2	6.10	0.66	1783.1	1	24/38	1.61E9
	87.90 - 88.48	-.QSGFLDPVNYR.-	1296.41	2	3.07	0.20	428.6	1	14/20	8.75E9
	76.02 - 76.60	-.SFVAVHNQPGLYVGQQAR.-	1972.20	3	4.56	0.54	1464.3	1	31/68	6.17E9
	76.00 - 76.58	-.SFVAVHNQPGLYVGQQAR.-	1972.20	2	5.16	0.62	1272.3	1	23/34	1.39E10
	137.00 - 138.32	-.TDTLLEIAVLPLDSYAKPDIEANYQGR.-	3007.34	3	4.11	0.49	756.8	1	29/104	1.42E10
	136.61 - 137.23	-.TDTLLEIAVLPLDSYAKPDIEANYQGR.-	3007.34	2	4.00	0.69	545.1	1	20/52	2.29E9
#34	ATPB_ECOLI (P00824					70.28			7 (7 0 0 0 0)	0.55
	106.04	-.AAPSYEELSNSQELLETGIK.-	2180.36	2	4.50	0.47	904.9	1	19/38	2.86E9
	102.34	-.AAPSYEELSNSQELLETGIK.-	2180.36	2	5.60	0.52	1450.7	1	24/38	1.33E9
	136.06	-.FLSQPFFVAEVFTGSPGK.-	1959.23	2	3.76	0.54	462.9	1	17/34	1.03E9
	126.78 - 127.62	-.GIM*EGEYDHLPEQAFYM*VGSIEEAVEK.-	3105.40	3	5.19	0.58	920.0	1	31/104	2.90E9
	92.60	-.NIAIEHSGYSVFAGVGER.-	1907.08	2	2.74	0.36	455.5	1	14/34	1.90E9
	111.39	-.QIASLGIYPAVDPLDSTSR.-	2004.23	2	2.55	0.48	296.4	1	14/36	1.52E9
	62.82	-.VYDALEVQNGNER.-	1507.59	2	2.66	0.52	1177.6	1	16/24	7.16E8
#35	CLPP_ECOLI (P19245					70.25			7 (7 0 0 0 0)	0.98
	138.46 - 139.82	-.FLSAPEAVEYGLVDSILTHR.-	2218.49	2	3.41	0.59	658.2	1	16/38	3.52E9
	138.41 - 139.08	-.FLSAPEAVEYGLVDSILTHR.-	2218.49	3	3.13	0.59	575.5	1	31/76	5.33E9
	84.66 - 84.68	-.M*NELM*ALHTGQSLEQIER.-	2133.39	3	4.90	0.60	1271.6	1	32/68	3.26E9
	84.64	-.M*NELM*ALHTGQSLEQIER.-	2133.39	2	3.49	0.52	349.7	1	17/34	4.41E9
	91.78	-.M*NELMALHTGQSLEQIER.-	2117.39	2	3.13	0.25	333.1	1	13/34	1.74E9
	64.47 - 65.04	-.SFDIYSR.-	887.96	1	1.91	0.38	248.1	2	9/12	1.63E9
	81.46	-.VM*IHQPLGGYQGQATDIEIHAR.-	2451.75	2	4.01	0.56	874.4	1	25/42	1.72E9

#36	ATPF_ECOLI (P00859					70.24			7 (7 0 0 0 0)	0.51
	61.40 - 62.59	-.AEAQVIIEQANK.-	1314.47	2	4.28	0.48	1680.3	1	19/22	1.21E9
	62.28 - 62.44	-.AEAQVIIEQANK.-	1314.47	1	2.26	0.36	654.3	1	13/22	5.31E8
	56.53 - 57.67	-.AKAEAQVIIEQANK.-	1513.72	2	4.36	0.65	1689.2	1	18/26	7.25E8
	68.54	-.KQVAILAVAGAEK.-	1298.56	2	3.46	0.52	1661.5	1	19/24	1.02E9
	76.54	-.SQILDEAKAEAEQER.-	1717.82	2	4.72	0.68	1587.9	1	19/28	1.53E9
	139.18 - 140.65	-.SVDEAANSDIVDKLVAEL.-	1889.05	2	3.77	0.53	501.1	1	15/34	2.72E9
	137.02 - 137.59	-.SVDEAANSDIVDKLVAEL.-	1889.05	2	2.90	0.45	332.3	1	14/34	3.46E9
#37	Q8XEB8 (Q8XEB8) Hy					70.22			7 (7 0 0 0 0)	0.79
	119.37 - 120.75	-.LDKNDAAVLLVDHQAGLLSLVR.-	2361.73	3	4.36	0.60	1511.2	1	32/84	6.92E9
	126.80	-.NDIEGLATLFSNHIPDYR.-	2076.26	3	3.86	0.52	1101.1	1	30/68	1.10E9
	126.74 - 127.83	-.NDIEGLATLFSNHIPDYR.-	2076.26	2	3.19	0.54	643.0	1	17/34	2.35E9
	71.44 - 72.34	-.NLM*TSYDTLTK.-	1303.46	2	2.97	0.42	658.6	1	14/20	1.75E9
	90.49	-.NNVLALGDLAK.-	1128.30	1	2.09	0.28	235.2	5	11/20	1.13E9
	90.51 - 91.37	-.PGNINAWDNEDFVK.-	1619.72	2	3.49	0.60	1071.0	1	17/26	2.43E9
	150.97 - 152.11	-.YFNLPTILTTSFETGPNGPLVPELK.-	2750.14	2	3.68	0.64	1530.4	1	23/48	1.82E9
#38	SODF_ECOLI (P09157					70.21			7 (7 0 0 0 0)	0.48
	84.93	-.DALAPHISAETIEYHYGK.-	2016.20	3	4.30	0.61	1007.4	1	30/68	1.88E9
	85.01	-.DALAPHISAETIEYHYGK.-	2016.20	2	3.87	0.68	699.3	1	17/34	1.44E9
	79.63	-.HHQTYVTNLNNLIK.-	1695.90	2	3.22	0.49	534.5	1	13/26	1.83E9
	71.71	-.HHQTYVTNLNNLIK.-	1695.90	2	2.74	0.45	883.3	1	15/26	1.29E9
	120.73	-.NFGSGWTWLVK.-	1295.47	2	3.02	0.34	546.7	1	15/20	1.08E9
	108.59	-.SFELPALPYAK.-	1236.44	2	2.84	0.17	894.3	1	16/20	1.10E9
	71.58 - 72.09	-.SLEEIIR.-	859.99	1	1.97	0.12	457.9	2	9/12	2.06E9
#39	RL3_ECO57 (P60440)					70.20			7 (7 0 0 0 0)	2.64
	10.59	-.AIQVTTGAK.-	889.03	1	1.99	0.39	710.3	1	12/16	4.17E9
	74.04 - 74.61	-.GAVPGATGSDLIVKPAVK.-	1680.97	1	2.95	0.39	644.8	1	15/34	1.99E9
	73.30 - 74.38	-.GAVPGATGSDLIVKPAVK.-	1680.97	2	3.72	0.59	959.2	1	25/34	1.13E10
	77.62	-.GAVPGATGSDLIVKPAVKA.-	1752.05	2	4.04	0.53	1210.1	1	23/36	1.67E9
	123.16 - 124.30	-.IFTEDGVSIPVTVIEVEANR.-	2189.45	2	4.02	0.56	355.1	1	18/38	1.95E10
	124.88 - 125.64	-.IFTEDGVSIPVTVIEVEANR.-	2189.45	2	3.73	0.54	232.2	1	15/38	1.40E10
	76.96 - 77.77	-.VTVQSLDVVR.-	1116.29	2	3.60	0.44	1592.4	1	17/18	5.89E9
#40	ALKH_ECOLI (P10177					60.23			6 (6 0 0 0 0)	1.02
	138.36 - 138.63	-.AATEGTIPLIPGISTVSELM*LGM*DYGLK.-	2911.38	2	2.97	0.53	90.4	5	13/54	1.74E9
	138.11 - 138.68	-.AATEGTIPLIPGISTVSELM*LGM*DYGLK.-	2911.38	3	3.67	0.46	191.4	41	22/108	4.27E9
	93.28 - 93.99	-.ALQAIAGPFSQVR.-	1358.57	2	3.08	0.44	739.5	1	16/24	2.58E9
	75.80	-.FCPTGGISPANYR.-	1440.58	2	2.78	0.51	1256.7	1	20/24	4.30E9
	117.81	-.TSAESILTTGPVVPVIVVK.-	1911.27	3	3.64	0.51	867.3	1	27/72	1.46E9
	116.84 - 118.03	-.TSAESILTTGPVVPVIVVK.-	1911.27	2	4.51	0.59	703.8	1	19/36	8.34E9
#41	ADHE_ECOLI (P17547					60.22			6 (6 0 0 0 0)	0.39
	61.43 - 61.79	-.EYASFTQEQVDK.-	1445.51	1	2.23	0.37	263.9	1	14/22	5.29E8
	63.81	-.FATHGGYLLQGK.-	1292.47	2	2.99	0.36	945.0	1	17/22	6.57E8
	127.05 - 127.60	-.FLFNNGYADQITSVLK.-	1831.06	2	4.43	0.51	2375.3	1	24/30	2.27E9
	104.98	-.ILIGEVTVVDESEPFAHEK.-	2113.35	2	2.58	0.30	479.3	2	14/36	1.55E9
	112.04 - 113.37	-.ILINTPASQGGIGDLYNFK.-	2022.29	2	3.99	0.61	523.7	1	18/36	2.99E9
	59.49	-.NAIIFSPHPR.-	1152.33	2	2.73	0.54	1226.5	1	16/18	7.09E8
#42	GMHA_ECO57 (P6322					50.30			5 (5 0 0 0 0)	0.39
	97.32 - 98.03	-.EGDVLLGISTSGNSANVIK.-	1875.07	2	4.44	0.73	961.8	1	20/36	2.81E9
	99.63 - 100.52	-.EGDVLLGISTSGNSANVIK.-	1875.07	2	3.55	0.62	803.8	1	18/36	1.73E9
	35.07 - 35.80	-.M*AGTADIEIR.-	1093.24	2	2.80	0.46	1194.0	1	15/18	2.55E8
	145.01	-.NELNEAAETLANFLK.-	1677.84	2	4.20	0.40	1180.5	1	18/28	1.11E9
	140.16	-.NELNEAAETLANFLKDDANIHAIQR.-	2812.05	3	6.01	0.66	1865.3	1	38/96	2.77E9
#43	LRP_ECOLI (P19494)					50.23			5 (5 0 0 0 0)	0.48
	101.59	-.GAPDVFEQFNTAVQK.-	1651.80	1	2.86	0.27	334.8	2	13/28	1.23E9
	101.36 - 101.50	-.GAPDVFEQFNTAVQK.-	1651.80	2	4.33	0.55	1345.5	1	20/28	5.13E9
	115.62	-.LEEIQECHLVSGDFDYLLK.-	2309.55	2	4.57	0.54	1215.9	1	19/36	2.10E9
	79.19	-.NILNELQK.-	972.12	1	2.06	0.19	351.6	1	10/14	1.43E9
	61.97 - 62.30	-.RVGLSPTPCLER.-	1385.59	2	2.56	0.25	574.2	2	13/22	7.56E8
#44	Q8XCI0 (Q8XCI0) FKB					50.21			5 (5 0 0 0 0)	0.44
	68.33	-.EGVNSTESGLQFR.-	1424.50	1	2.05	0.37	196.7	1	14/24	6.61E8
	155.90 - 156.48	-.GEPAEFPVNGVIPGWIEALTLM*PVGSK.-	2826.26	3	3.72	0.50	1136.0	1	34/104	2.81E9
	156.11 - 156.61	-.GEPAEFPVNGVIPGWIEALTLM*PVGSK.-	2826.26	2	4.23	0.61	322.1	1	18/52	1.58E9
	159.80 - 160.60	-.GEPAEFPVNGVIPGWIEALTLM*PVGSK.-	2826.26	2	3.39	0.62	373.7	1	19/52	6.33E8
	86.61 - 87.62	-.LIDGTVFDSSVAR.-	1380.53	2	3.30	0.45	984.2	1	18/24	4.16E9
#45	UPP_ECOLI (P25532)					50.19			5 (5 0 0 0 0)	0.74
	79.40 - 80.62	-.AGLGM*M*DGVLENVPSAR.-	1749.99	2	3.61	0.49	687.2	1	16/32	5.08E9
	118.61 - 119.21	-.AHPDVELYTASIDQGLNEHGYIIPGLGDAGDK	3367.62	3	3.73	0.53	320.1	4	28/124	3.04E9
	91.06	-.NEETLEPVPYFQK.-	1594.75	1	1.82	0.28	145.2	7	10/24	8.28E8
	90.10 - 91.10	-.NEETLEPVPYFQK.-	1594.75	2	3.85	0.52	848.0	1	17/24	4.39E9
	109.38 - 110.38	-.VTIEGWNGPVEIDQIK.-	1799.02	2	3.73	0.56	1179.7	1	20/30	3.11E9
#46	YFBU_ECO57 (Q8XCV					50.17			5 (5 0 0 0 0)	0.34
	75.94	-.ELDREFGELKEETCR.-	1912.04	2	3.10	0.46	396.9	1	14/28	1.59E9
	53.56 - 54.10	-.LILSNQYK.-	979.16	1	1.94	0.21	441.9	1	11/14	7.00E8
	61.56 - 62.03	-.M*LNVWHACPR.-	1300.51	2	2.72	0.38	900.0	1	14/18	7.73E8

	92.14 - 93.49	-.RVTFLGFDAATEAR.-	1554.73	2	2.71	0.35	628.7	1	14/26	3.33E9
	115.31	-.TIIDIM*EM*YHALHVSWSNLQDQQSIDER.-	3406.75	3	3.45	0.41	626.2	2	26/108	1.25E9
#47	EFG_ECOLI (P02996)					40.27			4 (4 0 0 0 0)	0.44
	109.97 - 110.60	-.AKPVLLEPIM*KVEVETPEENTGDVIGDLSR.-	3296.74	3	5.41	0.59	1233.7	1	36/116	2.14E9
	150.91 - 152.19	-.LGANPVPLQLAIGAEEHFTGVVDLVK.-	2689.10	3	4.63	0.57	534.4	1	31/100	1.13E9
	95.44 - 96.01	-.VEVETPEENTGDVIGDLSR.-	2060.16	2	5.10	0.53	579.1	1	20/36	3.54E9
	95.33	-.YDEAPSNVAQAVIEAR.-	1733.86	2	2.68	0.53	410.3	1	15/30	2.91E9
#48	Q8XE62 (Q8XE62) Yaj					40.27			4 (4 0 0 0 0)	0.28
	68.46	-.DNQIVTLTASR.-	1218.34	1	1.88	0.42	185.3	1	11/20	9.83E8
	111.52	-.FLLQEVLEK.-	1119.34	2	2.71	0.17	786.8	1	14/16	1.04E9
	162.48 - 163.05	-.GYM*VGPNGPVNLQIIVSQLYADVSQGNVR.-	3106.50	3	5.35	0.60	2144.3	1	40/112	1.60E9
	138.72	-.NIADAVNSVLTDTIADM*SQDTSIHEFIK.-	3066.34	3	3.96	0.33	559.7	1	32/108	2.67E9
#49	Q8X5N4 (Q8X5N4) Hyp					40.24			4 (4 0 0 0 0)	0.31
	128.63 - 129.24	-.M*ILVTSLGCGDSWPFLSER.-	2185.48	2	4.75	0.64	1349.8	1	21/36	1.72E9
	118.78	-.RADVAAHIHELANAPALNQQVYSLIEPDLKPA	3466.89	3	3.79	0.54	614.6	1	30/124	1.59E9
	104.40	-.TLELALAEQRPVVAVIR.-	1879.24	3	3.67	0.53	1018.2	1	29/64	1.56E9
	104.38	-.TLELALAEQRPVVAVIR.-	1879.24	2	3.16	0.45	333.7	11	12/32	1.94E9
#50	YNCE_ECO57 (Q8X9X					40.22			4 (4 0 0 0 0)	0.29
	86.11	-.AAEVLVVDTRNGNILAK.-	1784.05	2	3.33	0.49	737.2	1	16/32	1.35E9
	93.80 - 94.40	-.ELVADDATNTVYISGIGK.-	1867.05	2	4.12	0.72	1213.5	1	21/34	1.84E9
	94.03	-.LYTTNADGELITIDTADNK.-	2069.21	2	3.44	0.59	653.0	1	15/36	1.10E9
	89.33 - 89.89	-.LYTTNADGELITIDTADNK.-	2069.21	2	4.30	0.53	891.0	1	18/36	2.08E9
#51	DKSA_ECOLI (P18274					40.21			4 (4 0 0 0 0)	0.35
	52.28 - 52.88	-.PTADLCIDCK.-	1193.32	2	3.70	0.57	1324.8	1	16/18	5.37E8
	62.88 - 63.40	-.RLEARPTADLCIDCK.-	1819.04	2	3.15	0.50	380.1	1	14/28	1.04E9
	70.31 - 71.40	-.TVTHM*QDEAANFPDPVDR.-	2060.19	2	4.21	0.28	754.6	1	16/34	4.93E9
	80.21	-.TVTHMQDEAANFPDPVDR.-	2044.19	2	3.92	0.43	1086.8	1	18/34	1.27E9
#52	ATPD_ECOLI (P00831					40.21			4 (4 0 0 0 0)	0.38
	80.07	-.AAFDFAVEHQSVER.-	1606.72	2	4.05	0.56	1346.6	1	18/26	1.90E9
	11.18 - 11.29	-.AGDM*VIDGSVR.-	1136.26	2	2.81	0.35	717.5	1	15/20	2.98E9
	143.70	-.LNALPDVLEQFIHLR.-	1779.08	2	4.17	0.59	751.6	1	19/28	1.67E9
	143.59	-.LNALPDVLEQFIHLR.-	1779.08	3	3.64	0.52	629.1	1	24/56	1.87E9
#53	MTNN_ECOLI (P24247					40.21			4 (4 0 0 0 0)	0.12
	118.08	-.VAAALGATLLLEHCKPDVIINTGSAGGLAPTLI	3273.81	3	4.15	0.48	783.9	1	30/128	1.79E9
	38.25 - 39.27	-.VGDIVVSDEAR.-	1160.26	2	3.30	0.49	1129.4	1	18/20	2.23E8
	36.11 - 37.36	-.VGDIVVSDEAR.-	1160.26	2	3.28	0.43	864.3	1	16/20	2.85E8
	34.55 - 35.46	-.VGDIVVSDEAR.-	1160.26	2	3.55	0.47	1295.8	1	17/20	2.55E8
#54	GT_ECOLI (P39100) G					40.20			4 (4 0 0 0 0)	0.31
	110.92	-.LNLEGLEHIAAFM*QR.-	1759.02	2	3.33	0.57	686.2	1	14/28	1.90E9
	78.21	-.M*AERPEVQDALSAEGLK.-	1861.07	2	3.47	0.59	364.0	1	18/32	2.80E9
	72.48 - 73.74	-.RLENGDDYFAVNPK.-	1638.76	2	4.03	0.56	1277.6	1	20/26	1.80E9
	132.60	-.TIEWLNYIATELHK.-	1731.97	3	3.69	0.52	585.1	1	26/52	4.32E8
#55	SSPA_ECOLI (P05838					40.20			4 (4 0 0 0 0)	0.35
	108.30	-.DSFLASLTEAER.-	1339.43	2	3.26	0.44	1627.2	1	17/22	1.54E9
	106.33 - 107.72	-.IIM*EYLDERFPHPPLM*PVYPVAR.-	2817.32	3	3.44	0.37	681.3	1	29/88	2.53E9
	99.69 - 100.21	-.LPQLGIEFSGPGAK.-	1414.63	2	3.90	0.61	855.0	1	17/26	2.58E9
	96.19 - 96.74	-.SVM*TLFSGPTDIYSHQVR.-	2055.30	2	3.43	0.57	871.3	1	18/34	1.21E9
#56	Q8XBX3 (Q8XBX3) Ox					40.19			4 (4 0 0 0 0)	0.29
	102.25	-.GYTSLVVVPVGHHSVEDFNATLPK.-	2567.88	2	2.81	0.39	116.6	4	12/46	1.06E9
	80.50 - 81.68	-.KILDASHVVVFCAK.-	1587.88	2	3.85	0.67	1373.6	1	16/26	2.51E9
	61.89	-.SAAGNYVFNER.-	1228.30	2	2.99	0.47	1177.3	1	16/20	8.09E8
	89.91	-.SRLPQNITLTEV.-	1371.57	2	3.06	0.50	884.9	1	15/22	2.12E9
#57	GREA_ECOLI (P21346					40.19			4 (4 0 0 0 0)	0.24
	81.66	-.GLIGKEEDDVVVIK.-	1514.75	2	3.70	0.54	1177.5	1	17/26	2.01E9
	98.55	-.IVGDDEADFKQNLISVNSPIAR.-	2402.65	3	3.24	0.38	1148.6	1	29/84	2.25E9
	60.76	-.LSNAQVIDVTK.-	1188.36	1	1.83	0.26	650.4	3	13/20	3.34E8
	60.45 - 60.64	-.LSNAQVIDVTK.-	1188.36	2	3.84	0.50	1875.8	1	18/20	6.90E8
#58	Q8XDF1 (Q8XDF1) Ou					40.18			4 (4 0 0 0 0)	0.20
	69.51	-.FTNISGFANK.-	1099.22	1	2.36	0.34	447.6	1	12/18	9.32E8
	23.26	-.KAEQWATGLK.-	1132.29	2	2.83	0.28	855.2	1	14/18	6.65E7
	159.69	-.NSNFFGLVDGLNFAVQYLGK.-	2204.47	2	3.22	0.43	371.3	1	13/38	5.41E8
	86.65 - 88.06	-.YDANNIYLAANYGETR.-	1848.95	2	3.64	0.57	1166.6	1	19/30	2.82E9
#59	TPIS_ECOLI (P04790)					30.28			3 (3 0 0 0 0)	0.34
	85.57	-.DIGAQYIIIGHSER.-	1572.75	2	3.79	0.40	1207.4	1	17/26	2.04E9
	111.69 - 112.39	-.EAEGSHIM*LGAQNVDLNLSGAFTGETSAAM	3296.63	3	5.58	0.64	1144.2	1	31/124	2.70E9
	105.96	-.EQGLTPVLCIGETEAENEAGKTEEVCAR.-	3092.30	3	3.53	0.50	261.9	3	28/108	2.76E9
#60	RS7_ECO57 (P66607)					30.24			3 (3 0 0 0 0)	0.20
	146.84	-.STAESIVYSALETLAQR.-	1840.03	3	4.83	0.46	2566.1	1	35/64	8.11E8
	146.86	-.STAESIVYSALETLAQR.-	1840.03	2	4.05	0.45	1083.6	1	17/32	9.96E8
	75.44 - 76.48	-.VGGSTYQVPVEVRPVR.-	1743.99	2	2.67	0.04	455.8	1	14/30	2.53E9
#61	PPIA_ECOLI (P20752)					30.24			3 (3 0 0 0 0)	0.28
	131.05 - 132.03	-.APVSVQNFVDYVNSGFYNNTTFHR.-	2777.99	3	4.81	0.60	772.2	1	30/92	2.36E9
	121.29	-.TADKDSATSQFFINVADNAFLDHGQR.-	2870.04	3	4.79	0.58	1108.6	1	29/100	2.21E9
	96.26	-.VIPGFM*IQGGGFTEQM*QQK.-	2129.45	2	2.79	0.46	337.8	1	17/36	1.54E9

#62	GCH1_ECOLI (P27511					30.23			3 (3 0 0 0 0)	0.21
	83.22	-.DITLTSTCEHHFVTIDGK.-	2075.26	2	3.96	0.63	968.2	1	20/34	1.42E9
	69.70	-.EAALVHEALVAR.-	1279.47	2	3.16	0.53	735.6	1	16/22	1.07E9
	127.58 - 128.23	-.M*YVDEIFSGLDYANFPK.-	2026.25	2	4.57	0.55	1132.7	1	20/32	2.21E9
#63	Q9LAP1 (Q9LAP1) lha					30.23			3 (3 0 0 0 0)	0.22
	68.17 - 68.83	-.FTQNYSSLSAVQK.-	1473.61	2	3.65	0.29	1018.9	1	17/24	2.39E9
	112.00	-.KLTNAAASVSVISQEELQSSQYHDLAEALR.-	3260.56	3	4.56	0.50	512.6	3	30/116	1.44E9
	149.33	-.LNWQITEEVASWLGAR.-	1874.09	2	2.78	0.50	565.3	1	15/30	1.06E9
#64	MPRA_ECOLI (P24201					30.23			3 (3 0 0 0 0)	0.26
	62.53	-.CLHLQLTEK.-	1142.32	1	2.19	0.23	393.4	2	11/16	5.20E8
	105.73 - 106.25	-.HEDFPYQEILLTR.-	1661.84	2	4.55	0.61	1349.4	1	20/24	2.49E9
	84.76 - 85.88	-.M*DSSFTPIEQM*LK.-	1559.79	2	3.42	0.51	684.5	1	17/24	2.86E9
#65	RS3_ECOLI (P02352)					30.22			3 (3 0 0 0 0)	0.31
	62.36	-.GEILGGM*AAVEQPEKPAAQPK.-	2138.43	2	4.42	0.63	501.8	1	18/40	6.94E8
	100.33 - 101.06	-.KVVADIAGVPAQINIAEVR.-	1964.30	2	3.83	0.04	587.4	2	18/36	4.00E9
	84.89 - 86.21	-.LVADSITSQLER.-	1332.49	2	3.91	0.58	1374.3	1	19/22	2.08E9
#66	PYRE_ECO57 (Q8XD					30.22			3 (3 0 0 0 0)	0.27
	61.11	-.EAKDHGEGGNLVGSALQGR.-	1896.01	2	2.74	0.57	366.0	1	15/36	4.30E8
	63.23 - 64.06	-.GRGEISAIQEVER.-	1444.58	2	4.38	0.53	1815.5	1	19/24	1.15E9
	117.38	-.SPYFFNAGLFNTGR.-	1591.75	2	2.85	0.58	783.4	1	14/26	4.45E9
#67	ALF_ECOLI (P11604)					30.22			3 (3 0 0 0 0)	0.12
	67.24	-.ANEAYLQGQLGNPK.-	1503.64	1	3.05	0.50	446.2	2	13/26	5.40E8
	141.39	-.LLPWIDGLLDAGEK.-	1540.79	2	3.01	0.28	613.3	1	18/26	1.07E9
	102.54	-.VKAPVIVQFSNGGASFIAGK.-	1991.32	3	4.32	0.51	1796.6	1	34/76	1.06E9
#68	GNTY_ECO57 (P6302					30.20			3 (3 0 0 0 0)	0.13
	12.23	-.LLANQEEGTQIR.-	1372.51	2	3.97	0.43	1810.3	1	18/22	1.06E9
	78.57	-.VEYM*LQSQINPQLAGHGGR.-	2115.36	2	3.99	0.52	547.6	2	16/36	9.53E8
	108.82	-.VFVINPGTPNAECGVSYCPPDAVEATDTALK.	3294.60	2	2.71	0.46	159.9	2	13/60	8.47E8
#69	LOLA_ECO57 (P6131					30.20			3 (3 0 0 0 0)	0.19
	92.06	-.FTFTPPQGVTVDDQR.-	1708.85	2	2.91	0.52	545.5	1	17/28	1.54E9
	159.25 - 160.34	-.TLWFYNPFVEQATATWLK.-	2216.52	2	3.95	0.49	825.0	1	20/34	7.39E8
	80.70 - 80.85	-.VTDGSGAAVQEGQGDLWVK.-	1918.05	2	3.67	0.40	924.8	1	17/36	2.01E9
#70	ODP1_ECOLI (P06958					30.19			3 (3 0 0 0 0)	0.21
	119.81	-.DYGVGSDVYSVTSFTELAR.-	2067.20	2	3.83	0.67	722.0	1	17/36	2.01E9
	146.06 - 146.68	-.LELPSLQDFGALLEEQSK.-	2018.25	2	3.60	0.59	934.5	1	20/34	9.35E8
	90.90 - 91.48	-.VPYIAQVM*NDAPAVASTDYM*K.-	2317.63	2	3.42	0.08	675.2	1	16/40	1.70E9
#71	DEF_ECOLI (P27251)					30.19			3 (3 0 0 0 0)	0.28
	115.33 - 116.19	-.IVDDM*FETM*YAEEGIGLAATQVDIHQR.-	3086.40	3	3.82	0.56	743.6	1	26/104	3.96E9
	102.81	-.LFM*DYLSPLK.-	1243.50	2	3.45	0.42	1187.6	1	16/18	1.41E9
	65.19 - 65.81	-.VAKPVEEVNAEIQR.-	1582.78	2	2.57	0.27	837.8	1	17/26	8.47E8
#72	DCEB_ECOLI (P28302					30.19			3 (3 0 0 0 0)	0.28
	89.56	-.LKDGEDPGYTLYDLSER.-	1972.10	2	3.78	0.57	331.0	1	16/32	2.14E9
	91.15	-.LKDGEDPGYTLYDLSER.-	1972.10	2	3.46	0.50	299.5	3	14/32	1.69E9
	88.39 - 89.63	-.LKDGEDPGYTLYDLSER.-	1972.10	3	3.67	0.19	894.9	1	25/64	2.36E9
#73	OMPA_ECOLI (P0293					30.18			3 (3 0 0 0 0)	0.22
	12.59 - 15.20	-.IGSDAYNQGLSER.-	1410.47	2	3.20	0.47	890.1	1	17/24	1.51E9
	90.35	-.RAQSVVDYLISK.-	1379.59	2	3.60	0.37	1505.3	1	18/22	1.64E9
	91.56	-.SDVLFNFNK.-	1084.21	1	2.12	0.48	513.1	1	11/16	1.71E9
#74	FABA_ECOLI (P18391					30.18			3 (3 0 0 0 0)	0.12
	11.53	-.ALGVGEVK.-	772.91	1	2.15	0.30	655.3	1	10/14	1.27E9
	57.58 - 58.12	-.ESYTKEDLLASGR.-	1469.58	2	3.58	0.62	744.7	1	15/24	6.64E8
	68.21	-.LIYTASDLK.-	1024.19	2	2.58	0.44	534.6	1	14/16	8.34E8
#75	Q8X592 (Q8X592) Hyp					30.18			3 (3 0 0 0 0)	0.23
	68.52 - 68.56	-.LSDEVTDSPIVDK.-	1418.53	1	1.90	0.31	360.6	1	12/24	8.40E8
	68.40 - 68.98	-.LSDEVTDSPIVDK.-	1418.53	2	3.55	0.48	948.3	1	19/24	2.46E9
	96.21	-.STM*M*AGLSYAHFTQYGYLR.-	2230.51	2	2.66	0.39	368.8	1	15/36	1.78E9
#76	Q8XEB4 (Q8XEB4) Fo					30.17			3 (3 0 0 0 0)	0.18
	120.81	-.DAIPTQSVLTITSNVVYGK.-	2007.27	2	2.58	0.56	268.1	4	13/36	1.71E9
	93.22	-.VDDLAVDLVER.-	1244.38	2	3.45	0.49	1839.1	1	19/20	1.33E9
	57.81 - 58.37	-.VVGLQTEAPLKR.-	1311.56	2	2.51	0.46	752.0	1	14/22	8.45E8
#77	RPIA_ECOLI (P27252)					30.16			3 (3 0 0 0 0)	0.26
	65.92 - 66.44	-.FICIADASK.-	1025.17	1	2.30	0.40	890.6	1	12/16	9.05E8
	110.56 - 111.83	-.FPLPVEVIPM*AR.-	1385.70	2	2.68	0.49	506.6	1	14/22	2.44E9
	77.98 - 78.70	-.GADVALIGTPDGVK.-	1313.48	2	3.26	0.48	1073.1	1	17/26	2.51E9
#78	PTNA_ECOLI (P08186					30.16			3 (3 0 0 0 0)	0.21
	138.86 - 139.58	-.DDDPSFDELVALAVETGR.-	1950.05	2	3.12	0.42	575.7	1	15/34	1.81E9
	136.59	-.GVLFLVDTWGGSPFNAASR.-	1995.23	2	3.02	0.42	507.0	1	14/36	1.69E9
	99.78	-.TLLTQVAPPGVTAHVVDVAK.-	2017.36	2	2.89	0.46	166.4	6	13/38	1.21E9
#79	EFTS_ECOLI (P02997					30.15			3 (3 0 0 0 0)	0.26
	75.62 - 76.29	-.EYQVQLDIAM*QSGKPK.-	1852.10	2	3.09	0.53	456.8	1	15/30	2.15E9
	95.37 - 95.89	-.FTGEVSLTGQPFVM*EPSK.-	1971.22	2	2.96	0.42	379.2	2	14/34	1.56E9
	10.76	-.GADEELVK.-	860.93	1	1.82	0.10	578.5	2	10/14	2.08E9
#80	BCCP_ECOLI (P02905					30.15			3 (3 0 0 0 0)	0.13
	51.93 - 52.52	-.AFIEVGQK.-	892.03	2	2.65	0.09	732.5	1	13/14	3.62E8

	51.26 - 54.29	-.AFIEVGQK.-	892.03	1	2.01	0.10	799.4	1	11/14	1.11E9
	60.80 - 61.72	-.SPM*VGTFYR.-	1074.24	2	2.98	0.54	673.8	2	13/16	1.44E9
#81	ACP_ECOLI (P02901)					30.14			3 (3 0 0 0 0)	0.10
	62.65 - 63.17	-.IIGEQLGVK.-	957.15	2	2.85	0.17	347.7	1	13/16	1.03E9
	62.77	-.IIGEQLGVK.-	957.15	1	2.04	0.22	747.8	1	12/16	1.01E9
	33.81 - 34.73	-.KIIGEQLGVK.-	1085.32	2	2.64	0.25	571.9	1	13/18	2.55E8
#82	Q8X9J7 (Q8X9J7) Hyp					20.27			2 (2 0 0 0 0)	0.24
	90.22	-.QAYGQALAM*YHGQTYQIAPEQPLGDK.-	2896.18	3	5.32	0.63	795.8	1	29/100	1.54E9
	109.96 - 110.54	-.TIVDQELLPYVQVK.-	1645.92	2	3.30	0.47	568.8	1	16/26	3.74E9
#83	Q8X6U4 (Q8X6U4) Put					20.26			2 (2 0 0 0 0)	0.12
	152.98 - 153.35	-.VLLTLEELELPYEQILAGR.-	2200.56	2	5.17	0.49	430.5	1	16/36	1.37E9
	71.88	-.WYQQLTERPAVR.-	1547.74	2	2.82	0.53	560.2	1	12/22	1.30E9
#84	RCSB_ECOLI (P14374					20.26			2 (2 0 0 0 0)	0.16
	155.44 - 156.24	-.KSLEQIEWVNVVGEFEDSTALINNLPK.-	3074.43	3	5.11	0.54	2072.7	1	37/104	9.40E8
	119.83	-.LDAHVLITDLSM*PGDKYGDGITLIK.-	2703.11	3	4.18	0.52	1071.6	1	31/96	2.50E9
#85	RRAA_ECOLI (P32165					20.25			2 (2 0 0 0 0)	0.13
	64.41	-.ALVDAELAR.-	958.09	2	2.54	0.36	958.3	1	14/16	8.14E8
	142.85 - 143.00	-.M*KYDTSELCDIYQEDVNVVEPLFSNFGGR.-	3443.73	3	5.02	0.45	655.6	2	29/112	2.11E9
#86	Q8XCD3 (Q8XCD3) Hy					20.24			2 (2 0 0 0 0)	0.14
	132.73	-.LNEFPEQFEPLFGLR.-	1837.07	2	2.77	0.36	285.4	2	13/28	1.89E9
	148.66	-.TGPLNESELEWLDDILTK.-	2074.27	2	4.84	0.56	1084.0	1	18/34	1.24E9
#87	Q8XDF3 (Q8XDF3) As					20.24			2 (2 0 0 0 0)	0.10
	157.20 - 158.53	-.M*FENITAAPADPILGLADLFR.-	2292.64	2	4.81	0.61	605.7	1	20/40	1.20E9
	72.17	-.VGACTLVAADSETVDR.-	1664.79	2	2.95	0.60	1279.3	1	19/30	1.03E9
#88	Q8X5N7 (Q8X5N7) Put					20.23			2 (2 0 0 0 0)	0.15
	144.62	-.QLSSEGILSLRPDSVITWQDAGPQIVLDQLR.-	3436.86	3	4.68	0.60	965.7	1	30/120	1.45E9
	106.15	-.TLQVPEQGEALVTQISQR.-	1998.23	2	3.55	0.54	755.1	1	20/34	1.94E9
#89	YDCL_ECO57 (P64452					20.23			2 (2 0 0 0 0)	0.23
	141.21 - 142.51	-.EGLQFYEVVPVALVVAGTQM*ATGHR.-	2690.07	3	3.71	0.46	688.0	1	29/96	2.34E9
	106.80 - 107.58	-.LYFEGELIDAATNKPVIK.-	2022.33	2	4.64	0.61	973.9	1	18/34	2.78E9
#90	APT_ECO57 (Q8XD48					20.23			2 (2 0 0 0 0)	0.20
	150.72 - 152.02	-.GFLFGAPVALGLGVGFVPVR.-	1974.38	2	4.61	0.60	1492.4	1	24/38	2.29E9
	106.76	-.QGITSYSLVPFPGH.-	1503.68	2	3.91	0.59	845.1	1	16/26	2.16E9
#91	Q8XAA8 (Q8XAA8) Hy					20.23			2 (2 0 0 0 0)	0.19
	73.47 - 74.72	-.AAAQLQQGLADTSDENLK.-	1874.00	2	3.68	0.29	885.5	1	17/34	3.17E9
	91.60	-.SASLAYQNAVTAVSEGKPDSIPAAEK.-	2605.84	2	4.58	0.66	690.0	1	19/50	1.04E9
#92	YBEL_ECOLI (P46129)					20.23			2 (2 0 0 0 0)	0.15
	84.34	-.DIDALVEQAR.-	1130.23	2	3.03	0.38	1346.0	1	15/18	1.30E9
	136.36 - 136.88	-.DLEEFAM*SYEESLKEESDSVFM*R.-	2805.00	3	4.51	0.62	997.3	1	32/88	1.96E9
#93	IMDH_ECOLI (P06981)					20.22			2 (2 0 0 0 0)	0.13
	131.91	-.EALTFDDVLLVPAHSTVLPNTADLSTQLTK.-	3211.61	3	4.48	0.50	1024.4	1	36/116	1.47E9
	89.42	-.FVTDLNQPVSVYM*TPK.-	1856.13	2	2.55	0.39	385.6	1	14/30	1.44E9
#94	ATPA_ECOLI (P00822					20.21			2 (2 0 0 0 0)	0.12
	124.60	-.ELAAFSQFASDLDDATR.-	1857.96	2	4.27	0.66	1286.0	1	19/32	1.63E9
	80.47	-.GYLADVELSK.-	1095.23	1	1.93	0.10	539.6	2	12/18	1.02E9
#95	YBEY_ECO57 (Q8XBK					20.21			2 (2 0 0 0 0)	0.14
	145.62 - 146.22	-.DKPTNVLSFPFEVPPGM*EM*SLLGDLVICR.-	3295.81	3	4.23	0.55	650.2	1	35/112	1.78E9
	70.77	-.VVDTAESHSLNLTYR.-	1705.85	2	3.54	0.70	1216.3	1	16/28	1.40E9
#96	SSB_ECOLI (P02339)					20.21			2 (2 0 0 0 0)	0.24
	90.39	-.VILVGNLGQDPEVR.-	1509.73	1	2.49	0.40	219.0	6	12/26	8.02E8
	90.18 - 90.79	-.VILVGNLGQDPEVR.-	1509.73	2	4.20	0.56	1385.3	1	20/26	4.52E9
#97	Q7DBF7 (Q7DBF7) Ac					20.20			2 (2 0 0 0 0)	0.13
	148.00	-.QECGSDYALVFVDDVLAGK.-	2087.27	2	4.04	0.61	617.5	1	18/36	1.37E9
	101.79	-.VSESILLHGVEPITIK.-	1736.05	2	3.24	0.52	489.9	1	14/30	1.61E9
#98	GLR2_ECOLI (P39811					20.20			2 (2 0 0 0 0)	0.20
	107.12 - 108.14	-.NIPVELHVLLNDDAETPTR.-	2147.38	2	4.03	0.44	576.4	1	15/36	2.25E9
	78.82 - 79.01	-.SAFDEFSTPAAR.-	1299.37	2	2.88	0.41	1288.2	1	17/22	2.26E9
#99	Q8XD73 (Q8XD73) Gly					20.20			2 (2 0 0 0 0)	0.52
	104.50	-.LVFITDTVQPVINQGGTK.-	1931.22	2	4.00	0.56	600.8	1	17/34	2.54E9
	133.87 - 135.32	-.QSPFSFVLPFNPADVQPNAR.-	2232.48	2	2.62	0.40	293.3	1	12/38	9.09E9
#100	YIEF_ECOLI (P31465)					20.19			2 (2 0 0 0 0)	0.15
	118.18	-.LQVVTLLGSLR.-	1199.47	2	2.82	0.39	1304.8	1	15/20	5.91E8
	142.37 - 142.95	-.VDPQTGEVIDQGTLDHLTGQLTAFGEFIQR.-	3287.58	3	3.90	0.53	811.9	1	36/116	2.68E9
#101	Q7DBF8 (Q7DBF8) Glu					20.19			2 (2 0 0 0 0)	0.17
	113.04 - 114.05	-.ELSAEGFNFIGTGVSGGEEGALK.-	2270.44	2	3.86	0.25	583.8	1	18/44	1.76E9
	110.36	-.ELSAEGFNFIGTGVSGGEEGALKGPSIM*PGC	3239.56	3	3.50	0.44	727.7	1	31/128	1.92E9
#102	RUVA_ECOLI (P08576					20.19			2 (2 0 0 0 0)	0.12
	59.55 - 60.13	-.IARPDASSETLIR.-	1429.60	2	2.67	0.22	702.6	1	15/24	6.49E8
	148.70 - 150.11	-.LALAILSGM*SAQQFVNAVEREEVGALVK.-	2961.43	3	3.83	0.52	758.9	1	29/108	1.98E9
#103	YQJD_ECO57 (P6458:					20.19			2 (2 0 0 0 0)	0.23
	120.77	-.SLSDTLEEVLSSSGEK.-	1681.78	1	1.81	0.41	294.6	2	12/30	8.70E8
	120.59	-.SLSDTLEEVLSSSGEK.-	1681.78	2	3.81	0.65	780.6	1	16/30	4.26E9
#104	CH60_ECOLI (P06139					20.19			2 (2 0 0 0 0)	0.10
	162.20 - 163.57	-.AGKPLLIIAEDVEGEALATLVVNTM*R.-	2741.20	3	3.42	0.40	1233.5	1	29/100	6.69E8

	69.14	-.AIAQVGTISANSDETVGK.-	1761.91	2	3.79	0.58	987.6	1	21/34	1.51E9
#105	Q8X9J5 (Q8X9J5) Puta					20.18			2 (2 0 0 0 0)	0.20
	111.17 - 111.71	-.LAANWPLEQDELLTR.-	1769.98	2	3.67	0.49	260.7	1	14/28	2.41E9
	71.36 - 72.74	-.VEAHATTPFWR.-	1315.46	2	2.54	0.54	942.5	1	14/20	1.94E9
#106	YIAF_ECOLI (P37667)					20.18			2 (2 0 0 0 0)	0.27
	100.46 - 101.54	-.EM*NGSLGVLAQQLQNAK.-	1818.05	2	3.59	0.53	474.4	1	15/32	3.55E9
	70.03 - 70.59	-.VPQDYVTQSGPLR.-	1460.62	2	2.68	0.32	950.9	1	15/24	2.37E9
#107	YBGI_ECOLI (P75743)					20.18			2 (2 0 0 0 0)	0.30
	89.58 - 90.52	-.LNSAAISDYAPNGLQVEGK.-	1948.12	2	3.54	0.51	577.4	1	17/36	4.54E9
	92.35 - 93.13	-.VAWCTGGGQSFIDSAAR.-	1783.92	2	2.76	0.27	1137.4	1	20/32	2.14E9
#108	HIS4_ECO57 (Q9S5G4					20.17			2 (2 0 0 0 0)	0.11
	121.77	-.LQDYAAQGAEVLHLVDLTGAK.-	2213.48	3	3.39	0.45	485.0	1	27/80	1.22E9
	98.38	-.TLVAGVNVPVQVGGGVR.-	1622.89	2	2.98	0.57	868.9	1	19/32	1.16E9
#109	GRPE_ECO57 (Q7ABI					20.17			2 (2 0 0 0 0)	0.24
	95.27	-.ANPDM*SAM*VEGIELTLK.-	1852.12	2	3.32	0.60	224.0	1	14/32	2.61E9
	136.83 - 137.42	-.FINELLPVIDSLDR.-	1644.89	2	3.32	0.40	576.3	1	16/26	2.64E9
#110	Q8X5V9 (Q8X5V9) Vita					20.16			2 (2 0 0 0 0)	0.20
	127.98 - 129.09	-.APLYPDDILWNFEK.-	1721.93	2	2.95	0.44	302.7	2	13/26	2.53E9
	88.04 - 88.64	-.FSPDM*TPEDPIVM*ESIK.-	1969.22	2	3.23	0.50	221.2	1	12/32	1.82E9
#111	RS2_ECOLI (P02351)					20.16			2 (2 0 0 0 0)	0.03
	21.35	-.LKDLETQSQDGTFDK.-	1725.84	2	3.13	0.49	1094.0	1	18/28	9.72E7
	52.42 - 53.33	-.VHIINLEK.-	966.16	1	2.07	0.29	147.1	6	8/14	6.76E8
#112	OMPT_ECO57 (P5860					20.15			2 (2 0 0 0 0)	0.08
	51.72 - 53.05	-.LGLM*AGYQESR.-	1241.40	2	2.77	0.54	557.0	1	16/20	7.12E8
	93.47	-.YEDFELGGTFK.-	1306.40	2	3.01	0.46	1187.7	1	17/20	1.05E9
#113	Q8X938 (Q8X938) Puta					20.14			2 (2 0 0 0 0)	0.17
	97.48	-.LTGLEPGELFVHR.-	1468.68	2	2.87	0.51	566.8	1	17/24	1.42E9
	89.06	-.M*LVEEDPGFFEK.-	1457.63	2	2.68	0.50	789.7	1	14/22	2.32E9
#114	YCEI_ECOLI (P37904)					20.14			2 (2 0 0 0 0)	0.06
	60.15	-.IDKEGQHAFVNFR.-	1561.73	2	2.85	0.50	776.7	1	16/24	3.89E8
	64.14 - 65.08	-.YPQATFTSTSVK.-	1330.47	2	2.68	0.41	867.4	1	15/22	1.00E9
#115	Q8XBI0 (Q8XBI0) Hypo					10.29			1 (1 0 0 0 0)	0.08
	129.50 - 130.93	-.SQLQSALSWLEQQSASSHIESSTLPEVR.-	3100.34	3	5.77	0.48	1020.4	1	33/108	1.71E9
#116	EAE_ECO57 (P43261)					10.26			1 (1 0 0 0 0)	0.11
	134.26 - 135.64	-.LPFEYSALPLLGSAPLVAAGGVAGHTNK.-	2752.16	3	5.30	0.65	949.0	1	31/108	2.48E9
#117	Q8XBL3 (Q8XBL3) PEI					10.26			1 (1 0 0 0 0)	0.03
	163.42	-.IIDLSAIQDEVILVAADLTPSETAQLNLKK.-	3223.70	3	5.25	0.59	1335.3	1	43/116	7.22E8
#118	Q8XDP2 (Q8XDP2) Pu					10.26			1 (1 0 0 0 0)	0.04
	63.34 - 63.93	-.TQGLGPANPIASNSTAEGK.-	1813.95	2	5.22	0.73	1084.9	1	22/36	8.75E8
#119	RPE_ECOLI (P32661)					10.26			1 (1 0 0 0 0)	0.08
	130.34 - 131.23	-.VNNIGEIAAAGADM*FVAGSAIFDQPDYKK.-	3030.36	3	5.16	0.44	520.1	1	29/112	1.69E9
#120	YRBD_ECO57 (P6460					10.26			1 (1 0 0 0 0)	0.06
	160.84 - 161.57	-.SAM*VLEDLIGQFLYGSK.-	1888.17	2	5.13	0.70	1273.6	1	20/32	1.27E9
#121	MDH_ECO57 (P61891					10.25			1 (1 0 0 0 0)	0.10
	132.48 - 132.54	-.TQLPSGSELSLYDIAPVTPGVAVDLSHIPTAV	3377.83	3	4.95	0.55	1091.8	1	38/128	2.28E9
#122	YEAK_ECO57 (P6448					10.24			1 (1 0 0 0 0)	0.20
	144.22 - 145.49	-.ASLASPAEVDELTGCVFGAIPPFSFHPK.-	2946.30	3	4.89	0.55	392.5	3	28/108	4.51E9
#123	LOLB_ECO57 (P61321					10.24			1 (1 0 0 0 0)	0.04
	125.02	-.LLLTNPLGSTELELNAQPGNVQLVDNK.-	2892.25	2	4.81	0.58	354.7	1	16/52	8.56E8
#124	Q8XCY2 (Q8XCY2) Tra					10.23			1 (1 0 0 0 0)	0.05
	125.11	-.TPGHPEVGYTAGVETTTGPLGQGIANAVGM*	3412.77	3	4.69	0.60	971.8	1	33/136	1.21E9
#125	FABI_ECOLI (P29132)					10.23			1 (1 0 0 0 0)	0.07
	129.84	-.SM*LNPGSALLTLSYLGAER.-	2010.30	2	4.68	0.66	1345.2	1	22/36	1.50E9
#126	Q7DBF5 (Q7DBF5) GD					10.23			1 (1 0 0 0 0)	0.08
	97.34	-.YFRPAEVDTLLGDPSK.-	1809.01	2	4.55	0.54	850.7	1	20/30	1.87E9
#127	DLDH_ECOLI (P00391					10.22			1 (1 0 0 0 0)	0.10
	127.21 - 127.38	-.VIPSIAYTEPEVAWVGLTEK.-	2203.52	2	4.48	0.60	1076.4	1	22/38	2.22E9
#128	Q8X633 (Q8X633) Out					10.22			1 (1 0 0 0 0)	0.08
	123.99 - 125.31	-.GPM*SSLYGSDALGGVVNIITK.-	2096.39	2	4.40	0.60	1075.6	1	20/40	1.72E9
#129	YCFC_ECO57 (Q8X73					10.22			1 (1 0 0 0 0)	0.06
	79.65	-.IQVTGSPAVLQSPQVQAK.-	1852.13	2	4.39	0.62	983.9	1	20/34	1.41E9
#130	YFIO_ECOLI (P77146)					10.22			1 (1 0 0 0 0)	0.11
	124.62 - 125.93	-.GLTNM*ALDDSALQGFFGVDR.-	2144.35	2	4.38	0.62	1325.8	1	20/38	2.36E9
#131	Q8X5J6 (Q8X5J6) Nuc					10.22			1 (1 0 0 0 0)	0.05
	104.91	-.LLVDNNSEGEYAIIPASVADK.-	2219.43	2	4.38	0.53	728.6	1	18/40	1.15E9
#132	Q7DBI4 (Q7DBI4) Puta					10.22			1 (1 0 0 0 0)	0.04
	150.03 - 150.34	-.TIEDVFIHLLSDTYSAEK.-	2082.30	2	4.30	0.64	1202.7	1	19/34	9.16E8
#133	Q8X8L3 (Q8X8L3) Urid					10.21			1 (1 0 0 0 0)	0.09
	151.26 - 152.27	-.LDGASLHFAPLEFPAVADFECTTALVEAAK.-	3192.56	3	4.29	0.46	803.7	1	27/116	2.07E9
#134	DEAD_ECO57 (Q8XA8					10.21			1 (1 0 0 0 0)	0.08
	71.52	-.VQQQLESSDLDQYR.-	1709.80	2	4.29	0.63	1181.0	1	17/26	1.84E9
#135	YDJA_ECOLI (P24250)					10.21			1 (1 0 0 0 0)	0.17
	99.09 - 99.61	-.LAEPAPTGEQLQNILR.-	1750.98	2	4.18	0.60	597.8	1	19/30	3.76E9
#136	CLPX_ECOLI (P33138					10.21			1 (1 0 0 0 0)	0.05

	147.60	-.LLDVPFTM*ADATTLTEAGYVGEDVENIIQK.-	3271.64	3	4.10	0.57	344.2	1	26/116	1.05E9
#137	Q8XCU0 (Q8XCU0) Pe					10.20			1 (1 0 0 0 0)	0.14
	91.97 - 92.18	-.VINGFM*IQGGGFEPGM*K.-	1815.11	2	4.08	0.54	996.4	1	19/32	3.13E9
#138	PUR4_ECO57 (Q8XA4					10.20			1 (1 0 0 0 0)	0.09
	151.26 - 152.27	-.LGLALAEDEIDYLQDAFTK.-	2126.35	2	4.01	0.59	710.2	1	15/36	2.07E9
#139	YFHP_ECOLI (P77484					10.20			1 (1 0 0 0 0)	0.04
	144.48	-.QGISLSYLEQLFSR.-	1641.85	2	3.91	0.64	1057.9	1	17/26	9.25E8
#140	EFP_ECOLI (P33398)					10.19			1 (1 0 0 0 0)	0.19
	125.97 - 126.86	-.VPLFVQIGEVIK.-	1342.65	2	3.89	0.49	1845.7	1	20/22	4.27E9
#141	Q8XCM3 (Q8XCM3) H					10.19			1 (1 0 0 0 0)	0.08
	114.35	-.IEDVHAFDGHIACDAASNSEIVLPLVVK.-	3021.36	3	3.89	0.48	911.2	1	31/108	1.86E9
#142	MDAB_ECOLI (P40717					10.19			1 (1 0 0 0 0)	0.10
	129.62 - 130.85	-.ANQFLGM*EPLPTFIANDVIK.-	2235.59	2	3.88	0.57	305.9	1	13/38	2.21E9
#143	YRAP_ECO57 (P6459					10.19			1 (1 0 0 0 0)	0.10
	82.15 - 82.36	-.VLLVGQSPNAELSAR.-	1554.77	2	3.85	0.62	866.7	1	17/28	2.26E9
#144	Q8X966 (Q8X966) Pyr					10.19			1 (1 0 0 0 0)	0.14
	131.38	-.VPDIGADEVEITEILVK.-	1841.09	2	3.78	0.54	464.5	1	15/32	3.00E9
#145	KPY1_ECOLI (P14178)					10.19			1 (1 0 0 0 0)	0.06
	107.89	-.GAVETAEKLDAPLIVVATQGGK.-	2168.48	2	3.77	0.43	665.1	1	18/42	1.28E9
#146	DEGP_ECOLI (P0937					10.19			1 (1 0 0 0 0)	0.03
	161.55 - 163.07	-.VGDYTVAIGNPFGLGETVTSGIVSALGR.-	2752.07	2	3.74	0.53	272.9	1	15/54	7.69E8
#147	Q8X7C0 (Q8X7C0) Hy					10.19			1 (1 0 0 0 0)	0.08
	81.40	-.KGGVIVYPTDSGYALGCK.-	1886.13	2	3.73	0.50	438.3	1	16/34	1.69E9
#148	RL2_ECO57 (P60424)					10.18			1 (1 0 0 0 0)	0.04
	80.09	-.AGDQIQSGVDAAIKPGNTLPM*R.-	2256.53	2	3.68	0.56	301.6	1	17/42	9.18E8
#149	HIS2_ECO57 (Q9S5G					10.18			1 (1 0 0 0 0)	0.10
	88.89	-.VGEEGVETALAATVHDR.-	1754.88	3	3.67	0.50	1651.9	1	33/64	2.13E9
#150	PHNA_ECOLI (P16680					10.18			1 (1 0 0 0 0)	0.14
	98.48 - 99.53	-.DANGNLLADGDSVTIIK.-	1716.87	2	3.62	0.53	717.3	1	18/32	3.17E9
#151	GMHB_ECO57 (P6322					10.17			1 (1 0 0 0 0)	0.04
	155.50 - 156.03	-.TGKPITPEAENAADWVLNSLADLPQAIK.-	2964.32	3	3.45	0.34	1157.9	1	31/108	8.48E8
#152	Q8XAN4 (Q8XAN4) Hy					10.17			1 (1 0 0 0 0)	0.08
	94.33	-.VIYQAGFTDHQLKPQEAQHLLDYR.-	2872.19	3	3.43	0.53	969.2	1	27/92	1.78E9
#153	Q7DBI3 (Q7DBI3) Hyp					10.17			1 (1 0 0 0 0)	0.09
	110.90	-.M*AALGQSIGGIFPSDEIVK.-	1950.25	2	3.38	0.42	922.0	1	18/36	2.06E9
#154	YGGT_ECO57 (P6456					10.17			1 (1 0 0 0 0)	0.02
	160.73	-.SPIEYVLIQLADPLLRPIR.-	2207.64	3	3.38	0.59	956.7	1	26/72	3.92E8
#155	HIS5_ECO57 (P58237)					10.17			1 (1 0 0 0 0)	0.07
	72.52	-.VSRDPDVVLLADK.-	1427.63	2	3.32	0.51	1566.4	1	17/24	1.51E9
#156	Q8X522 (Q8X522) Hyp					10.17			1 (1 0 0 0 0)	0.21
	139.42 - 140.71	-.DYLAELNEQGQAYAQFVASTAECC#GEGGIK	3338.50	3	3.30	0.29	232.0	485	19/116	4.60E9
#157	HSLV_ECOLI (P31059					10.16			1 (1 0 0 0 0)	0.08
	71.86	-.ALLENTELSAR.-	1217.36	2	3.22	0.50	1643.9	1	16/20	1.72E9
#158	LEUD_ECO57 (Q8XA0					10.16			1 (1 0 0 0 0)	0.05
	133.91	-.LSDAEVDELFALVK.-	1549.75	2	3.21	0.43	600.4	1	15/26	1.02E9
#159	EXBB_ECOLI (P18783					10.16			1 (1 0 0 0 0)	0.06
	113.81	-.SLSLHLLNEAQNELELSEGSDDNEGIKER.-	3241.42	3	3.20	0.45	622.9	1	29/112	1.25E9
#160	RS1_ECOLI (P02349)					10.16			1 (1 0 0 0 0)	0.04
	145.98	-.M*TESFAQLFEESLKEIETRPGSIVR.-	2915.27	3	3.16	0.58	852.7	1	29/96	8.76E8
#161	Q8XCK2 (Q8XCK2) Hy					10.16			1 (1 0 0 0 0)	0.03
	66.81	-.AAHQDEPQFGSQSTPLDER.-	2114.18	2	3.15	0.55	151.1	17	10/36	5.78E8
#162	RRMJ_ECOLI (P28692					10.16			1 (1 0 0 0 0)	0.12
	125.85	-.LFKPGM*TVVDLGAAPGGWSQYVVTQIGGK.	2994.46	3	3.11	0.09	433.2	15	25/112	2.68E9
#163	Q8XEC3 (Q8XEC3) Ch					10.15			1 (1 0 0 0 0)	0.04
	156.68	-.LSGKPLLLTELFLPASPLY.-	2073.50	2	3.10	0.53	326.7	1	16/36	9.01E8
#164	CLPB_ECO57 (P6328					10.15			1 (1 0 0 0 0)	0.12
	79.73 - 80.81	-.LPQVEGTGGDVQPSQDLVR.-	1996.17	2	3.07	0.33	349.8	6	11/36	2.57E9
#165	Q7DBF3 (Q7DBF3) Pe					10.15			1 (1 0 0 0 0)	0.09
	132.07 - 132.63	-.YVGTFGDISTFSFFGNK.-	1888.07	2	3.05	0.63	461.1	2	14/32	2.06E9
#166	AROK_ECOLI (P24167					10.15			1 (1 0 0 0 0)	0.10
	116.00	-.NPLYEEIADVTIR.-	1533.71	2	2.84	0.40	1762.1	1	18/24	2.32E9
#167	Q8XDI6 (Q8XDI6) Puta					10.15			1 (1 0 0 0 0)	0.02
	53.52 - 54.12	-.EQARPAAQQSEDELLR.-	1841.96	2	2.96	0.42	284.2	3	13/30	4.68E8
#168	METQ_ECO57 (Q8X8					10.15			1 (1 0 0 0 0)	0.11
	107.87	-.LVAVGNTFVYPIAGYSK.-	1800.09	2	2.95	0.62	496.2	1	13/32	2.39E9
#169	HLPA_ECOLI (P11457					10.15			1 (1 0 0 0 0)	0.08
	81.08	-.TGVSNTLENEFK.-	1339.43	2	2.92	0.54	691.5	1	14/22	1.71E9
#170	ATDA_ECOLI (P37354					10.14			1 (1 0 0 0 0)	0.03
	49.82 - 50.88	-.TPGQTLLKPTAQ.-	1255.45	2	2.89	0.37	1008.4	1	15/22	6.37E8
#171	PTNC_ECOLI (P08187					10.14			1 (1 0 0 0 0)	0.02
	59.53	-.TITVAFQHAADK.-	1302.46	2	2.87	0.54	953.5	1	16/22	4.62E8
#172	Q8X5V7 (Q8X5V7) Put					10.14			1 (1 0 0 0 0)	0.03
	153.76	-.LM*SLQDGAISAYDLLDLLR.-	2124.44	2	2.85	0.47	474.4	1	14/36	6.77E8
#173	Q8X9G7 (Q8X9G7) Ne					10.14			1 (1 0 0 0 0)	0.02

	56.71	-.VASPAIVEAKPIK.-	1323.61	2	2.81	0.50	1254.1	1	18/24		3.77E8
#174	YGIM_ECOLI (P39202)					10.14			1 (1 0 0 0 0)		0.04
	66.48	-.VAQSDSVINGLKEENQK.-	1860.02	2	2.80	0.46	520.9	1	16/32		9.65E8
#175	EVGA_ECOLI (P30854					10.14			1 (1 0 0 0 0)		0.06
	104.52	-.M*NAIIIDDHPLAIAAIR.-	1864.20	2	2.75	0.39	649.0	1	16/32		1.23E9
#176	YAGU_ECOLI (P77262					10.14			1 (1 0 0 0 0)		0.05
	67.35 - 68.02	-.ITHEPDPEIPLGSNR.-	1675.82	2	2.74	0.50	561.0	1	17/28		1.22E9
#177	GSA_ECO57 (Q8X4V5					10.14			1 (1 0 0 0 0)		0.07
	108.12	-.VALAGAQDYYGVEPDLTCLGK.-	2241.48	2	2.73	0.44	545.5	1	16/40		1.62E9
#178	PURA_ECOLI (P12283					10.14			1 (1 0 0 0 0)		0.07
	108.49	-.RIEELTGVPIDIISTGPDR.-	2082.34	2	2.71	0.40	241.4	5	12/36		1.46E9
#179	MOAB_ECOLI (P3074(					10.13			1 (1 0 0 0 0)		0.07
	73.32	-.SQVSTEFIPTR.-	1265.40	2	2.68	0.23	686.5	1	13/20		1.64E9
#180	G6PI_ECOLI (P11537)					10.13			1 (1 0 0 0 0)		0.07
	75.19	-.VFEGNRPTNSILLR.-	1616.85	2	2.54	0.31	660.7	1	15/26		1.48E9
#181	PUR9_ECO57 (Q8X61					10.13			1 (1 0 0 0 0)		0.06
	109.03	-.EGCSLEDAVENIDIGGPTM*VR.-	2279.46	2	2.64	0.33	552.7	1	14/40		1.30E9
#182	CPXR_ECOLI (P16244					10.13			1 (1 0 0 0 0)		0.03
	158.62 - 159.29	-.QTHQTPVIM*LTARGSELDR.-	2170.44	2	2.63	0.32	728.9	1	17/36		7.75E8
#183	TOLQ_ECOLI (P05828					10.13			1 (1 0 0 0 0)		0.09
	105.98 - 107.37	-.DNLTGSEQIFYSGFK.-	1706.83	2	2.59	0.38	532.4	1	13/28		1.98E9
#184	Q8X4W3 (Q8X4W3) Cl					10.13			1 (1 0 0 0 0)		0.02
	61.15	-.HIDSDIDIVK.-	1155.28	2	2.59	0.44	764.3	1	14/18		5.20E8
#185	MSRA_ECO57 (Q8XC(					10.13			1 (1 0 0 0 0)		0.06
	66.85	-.HLVSPADALPGR.-	1233.40	2	2.58	0.48	928.9	1	16/22		1.33E9
#186	LOLD_ECO57 (Q8X8E					10.13			1 (1 0 0 0 0)		0.05
	128.25	-.ALVNNPWLVLADEPTGNLDAR.-	2279.54	2	2.53	0.46	599.6	1	16/40		1.15E9
#187	RvrsDB|00004199					10.13			1 (1 0 0 0 0)		0.22
	97.63 - 98.29	-.TSQEDTAEQRSIVESFLQR.-	2225.36	2	2.50	0.06	292.7	12	12/36		4.91E9
#188	Q8X5I7 (Q8X5I7) Tauri					10.12			1 (1 0 0 0 0)		0.01
	37.26 - 40.73	-.PEDLIGK.-	771.88	1	2.40	0.18	154.0	1	9/12		3.31E8
#189	RvrsDB|00001234					10.11			1 (1 0 0 0 0)		0.03
	67.82	-.YDLLNPNRVVGSK.-	1475.68	1	2.26	0.27	186.6	3	10/24		5.68E8
#190	RL13_ECOLI (P02410)					10.11			1 (1 0 0 0 0)		0.05
	84.30	-.DWYVVDATGK.-	1154.25	1	2.22	0.34	772.1	1	13/18		1.19E9
#191	Q8XCB6 (Q8XCB6) Pu					10.11			1 (1 0 0 0 0)		0.08
	77.92 - 77.96	-.EAGLSDLSLK.-	1033.16	1	2.04	0.28	270.7	1	13/18		1.76E9
#192	Q8X4E8 (Q8X4E8) Hyp					10.11			1 (1 0 0 0 0)		0.04
	26.17 - 30.24	-.ITXQXNK.-	830.01	1	1.80	0.05	327.9	2	9/12		7.93E8
#193	Q8X588 (Q8X588) Hyp					10.10			1 (1 0 0 0 0)		0.01
	13.50	-.DAALIAAAQK.-	972.12	1	1.93	0.21	675.8	1	12/18	1	2.93E8
#194	Q8X6L2 (Q8X6L2) Puta					10.09			1 (1 0 0 0 0)		0.07
	32.78 - 38.34	-.NSAETLR.-	790.85	1	1.82	0.11	204.7	1	8/12		1.44E9
