# Supplementary material for: Proteins other than the locus of enterocyte effacement-encoded proteins contribute to Escherichia coli O157:H7 adherence to bovine rectoanal junction stratified squamous epithelial cells
Source: BMC Microbiol. 2012 Jun 12;12:103. doi: 10.1186/1471-2180-12-103 (PMC3420319; doi:10.1186/1471-2180-12-103)
Supplement: Additional file 11 — http://www.biomedcentral.com/imedia/1326109329675419/supp11.pdf. DATA SHEETS: O157-DMEM MS/MS data sheet 7. [file 1471-2180-12-103-S11.pdf]

DMEM-07 SequestReport

| | Reference<br>Time(s) | Sequence | MH+ | Charge | XC | Score<br>Delta Cn | Accession<br>Sp | RSp | Peptides (Hits)<br>Ions | Count | Area<br>Peak Area |
|---|---|---|---|---|---|---|---|---|---|---|---|
| #1 | RL24_ECO57 (P60625 | | | | | 230.24 | | | 23 (23 0 0 0 0) | | 1.88 |
| | 74.25 - 74.82 | -.DDEVIVLTGK.- | 1089.22 | 1 | 2.17 | 0.17 | 743.6 | 1 | 12/18 | | 4.33E9 |
| | 60.15 - 61.53 | -.DDEVIVLTGKDK.- | 1332.48 | 2 | 3.09 | 0.42 | 1014.6 | 1 | 17/22 | | 1.43E9 |
| | 124.40 - 125.59 | -.EAAIQVSNVAIFNAATGK.- | 1805.03 | 2 | 3.10 | 0.51 | 699.4 | 1 | 15/34 | | 2.82E9 |
| | 107.08 - 107.97 | -.EAAIQVSNVAIFNAATGK.- | 1805.03 | 2 | 4.74 | 0.72 | 1136.3 | 1 | 19/34 | | 3.56E9 |
| | 105.40 - 106.46 | -.EAAIQVSNVAIFNAATGK.- | 1805.03 | 2 | 4.70 | 0.65 | 1230.0 | 1 | 21/34 | | 4.62E9 |
| | 203.79 - 205.32 | -.EAAIQVSNVAIFNAATGK.- | 1805.03 | 2 | 3.97 | 0.65 | 1104.1 | 1 | 19/34 | | 1.92E8 |
| | 55.47 - 56.86 | -.HQKPVPALNQPGGIVEK.- | 1813.09 | 2 | 2.53 | 0.55 | 159.0 | 5 | 13/32 | | 6.73E8 |
| | 30.40 - 30.96 | -.HQKPVPALNQPGGIVEK.- | 1813.09 | 2 | 3.78 | 0.57 | 636.1 | 1 | 18/32 | | 9.51E8 |
| | 27.76 - 29.18 | -.HQKPVPALNQPGGIVEK.- | 1813.09 | 2 | 3.81 | 0.57 | 654.6 | 1 | 19/32 | | 4.66E8 |
| | 20.37 - 21.34 | -.HQKPVPALNQPGGIVEK.- | 1813.09 | 3 | 3.48 | 0.23 | 657.5 | 2 | 26/64 | | 1.80E8 |
| | 17.31 - 18.92 | -.HQKPVPALNQPGGIVEK.- | 1813.09 | 2 | 3.33 | 0.61 | 903.9 | 1 | 20/32 | | 2.15E8 |
| | 25.19 - 26.85 | -.HQKPVPALNQPGGIVEK.- | 1813.09 | 2 | 3.55 | 0.56 | 559.3 | 1 | 18/32 | | 4.37E8 |
| | 26.50 - 28.06 | -.HQKPVPALNQPGGIVEK.- | 1813.09 | 3 | 3.90 | 0.45 | 747.3 | 1 | 30/64 | | 3.80E8 |
| | 13.09 - 14.00 | -.HQKPVPALNQPGGIVEK.- | 1813.09 | 2 | 2.99 | 0.57 | 438.1 | 1 | 15/32 | | 9.55E8 |
| | 13.11 - 14.28 | -.HQKPVPALNQPGGIVEK.- | 1813.09 | 3 | 3.34 | 0.31 | 457.0 | 2 | 26/64 | | 8.57E8 |
| | 20.65 - 22.39 | -.HQKPVPALNQPGGIVEK.- | 1813.09 | 2 | 3.53 | 0.59 | 701.4 | 1 | 19/32 | | 1.09E8 |
| | 14.95 - 16.32 | -.HQKPVPALNQPGGIVEK.- | 1813.09 | 2 | 3.79 | 0.67 | 866.3 | 1 | 20/32 | | 5.32E8 |
| | 15.14 - 16.05 | -.HQKPVPALNQPGGIVEK.- | 1813.09 | 3 | 4.42 | 0.56 | 1300.1 | 1 | 32/64 | | 4.25E8 |
| | 23.09 - 24.31 | -.HQKPVPALNQPGGIVEK.- | 1813.09 | 2 | 3.68 | 0.61 | 1013.6 | 1 | 21/32 | | 2.22E8 |
| | 57.51 - 58.59 | -.IRRDDEVIVLTGK.- | 1514.75 | 2 | 3.63 | 0.52 | 733.4 | 1 | 16/24 | | 4.35E9 |
| | 10.57 - 11.47 | -.KHQKPVPALNQPGGIVEK.- | 1941.27 | 2 | 3.56 | 0.60 | 596.7 | 1 | 17/34 | | 2.86E9 |
| | 89.01 - 89.57 | -.VIVEGINLVK.- | 1084.33 | 1 | 2.03 | 0.17 | 406.4 | 3 | 10/18 | | 6.71E9 |
| | 89.07 - 89.70 | -.VIVEGINLVK.- | 1084.33 | 2 | 3.50 | 0.32 | 786.4 | 1 | 16/18 | | 3.26E9 |
| #2 | OMPX_ECOLI (P36546 | | | | | 210.32 | | | 21 (21 0 0 0 0) | | 5.97 |
| | 59.85 - 60.43 | -.FQTTEYPTYK.- | 1278.39 | 1 | 2.43 | 0.23 | 658.8 | 2 | 12/18 | | 2.83E9 |
| | 60.22 - 60.78 | -.FQTTEYPTYK.- | 1278.39 | 2 | 2.75 | 0.55 | 595.9 | 1 | 14/18 | | 6.42E9 |
| | 58.52 - 59.64 | -.FQTTEYPTYK.- | 1278.39 | 2 | 2.52 | 0.36 | 445.8 | 3 | 12/18 | | 5.72E9 |
| | 124.09 - 125.47 | -.INDWASIYGVVGVGYGK.- | 1799.02 | 2 | 2.85 | 0.58 | 360.7 | 1 | 12/32 | | 3.75E9 |
| | 118.87 - 120.04 | -.INDWASIYGVVGVGYGK.- | 1799.02 | 2 | 5.33 | 0.70 | 1232.7 | 1 | 20/32 | | 4.33E9 |
| | 120.82 - 121.67 | -.INDWASIYGVVGVGYGK.- | 1799.02 | 2 | 3.47 | 0.63 | 783.4 | 1 | 17/32 | | 3.51E9 |
| | 122.94 - 123.97 | -.INDWASIYGVVGVGYGK.- | 1799.02 | 2 | 3.70 | 0.65 | 895.4 | 1 | 17/32 | | 2.93E9 |
| | 116.83 - 118.30 | -.INDWASIYGVVGVGYGK.- | 1799.02 | 2 | 4.35 | 0.58 | 856.8 | 1 | 18/32 | | 5.75E9 |
| | 76.37 | -.NQYYGITAGPAYR.- | 1474.60 | 1 | 2.19 | 0.48 | 151.3 | 1 | 15/24 | | 2.91E9 |
| | 76.17 - 76.77 | -.NQYYGITAGPAYR.- | 1474.60 | 2 | 3.57 | 0.57 | 1430.2 | 1 | 17/24 | | 4.61E9 |
| | 108.19 | -.SVDVGTWIAGVGYR.- | 1480.65 | 1 | 2.38 | 0.43 | 166.3 | 1 | 13/26 | | 2.49E9 |
| | 107.95 - 108.53 | -.SVDVGTWIAGVGYR.- | 1480.65 | 2 | 3.24 | 0.58 | 1712.3 | 1 | 19/26 | | 9.80E9 |
| | 108.23 | -.SVDVGTWIAGVGYR.- | 1480.65 | 1 | 2.03 | 0.41 | 126.3 | 2 | 11/26 | | 1.79E9 |
| | 126.72 - 127.42 | -.SVDVGTWIAGVGYRF.- | 1627.83 | 2 | 3.76 | 0.61 | 1160.4 | 1 | 18/28 | | 5.53E9 |
| | 74.50 - 76.86 | -.TASSGDYNKNQYYGITAGPAYR.- | 2398.53 | 2 | 4.16 | 0.57 | 558.3 | 1 | 17/42 | | 7.39E9 |
| | 120.23 - 121.39 | -.YEEDNSPLGVIGSFTYTEK.- | 2150.28 | 2 | 4.13 | 0.61 | 881.3 | 1 | 20/36 | | 6.28E9 |
| | 118.57 - 119.17 | -.YEEDNSPLGVIGSFTYTEK.- | 2150.28 | 2 | 2.75 | 0.48 | 360.9 | 1 | 13/36 | | 1.88E9 |
| | 112.84 - 114.15 | -.YRYEEDNSPLGVIGSFTYTEK.- | 2469.65 | 2 | 4.25 | 0.59 | 785.7 | 1 | 18/40 | | 9.56E9 |
| | 114.71 - 115.83 | -.YRYEEDNSPLGVIGSFTYTEK.- | 2469.65 | 2 | 6.41 | 0.62 | 1633.0 | 1 | 23/40 | | 1.23E10 |
| | 113.44 - 114.55 | -.YRYEEDNSPLGVIGSFTYTEK.- | 2469.65 | 3 | 4.93 | 0.57 | 1724.9 | 1 | 33/80 | | 1.69E10 |
| | 115.14 - 116.33 | -.YRYEEDNSPLGVIGSFTYTEK.- | 2469.65 | 3 | 5.12 | 0.46 | 1621.8 | 1 | 34/80 | | 1.24E10 |
| #3 | RS5_ECOLI (P02356) 3 | | | | | 210.28 | | | 21 (21 0 0 0 0) | | 5.06 |
| | 69.99 - 71.27 | -.ATIDGLENM*NSPEM*VAAK.- | 1924.14 | 2 | 4.90 | 0.64 | 1071.4 | 1 | 20/34 | | 1.26E10 |
| | 68.23 - 69.39 | -.ATIDGLENM*NSPEM*VAAK.- | 1924.14 | 2 | 4.03 | 0.54 | 625.5 | 1 | 17/34 | | 1.15E10 |
| | 59.45 - 60.57 | -.ATIDGLENM*NSPEM*VAAK.- | 1924.14 | 2 | 4.24 | 0.42 | 401.7 | 2 | 13/34 | | 1.82E9 |
| | 78.07 | -.ATIDGLENM*NSPEMVAAK.- | 1908.14 | 2 | 4.08 | 0.15 | 492.5 | 3 | 14/34 | | 1.92E9 |
| | 82.17 | -.ATIDGLENMNSPEM*VAAK.- | 1908.14 | 2 | 4.51 | 0.21 | 999.1 | 1 | 17/34 | | 1.87E9 |
| | 82.85 - 83.43 | -.AVLEVAGVHNVLAK.- | 1420.68 | 1 | 2.76 | 0.50 | 611.9 | 1 | 15/26 | | 4.06E9 |
| | 69.35 | -.AYGSTNPINVVR.- | 1291.44 | 1 | 3.02 | 0.54 | 340.1 | 1 | 13/22 | | 3.60E9 |
| | 69.21 - 70.57 | -.AYGSTNPINVVR.- | 1291.44 | 2 | 3.59 | 0.59 | 1038.6 | 1 | 19/22 | | 9.05E9 |
| | 69.29 - 71.76 | -.AYGSTNPINVVR.- | 1291.44 | 1 | 2.15 | 0.39 | 224.2 | 1 | 12/22 | | 5.62E9 |
| | 120.52 - 121.63 | -.IFSFTALTVVGDGNGR.- | 1654.85 | 2 | 4.16 | 0.62 | 2687.2 | 1 | 24/30 | | 5.50E9 |
| | 122.27 - 123.61 | -.IFSFTALTVVGDGNGR.- | 1654.85 | 2 | 3.03 | 0.53 | 659.8 | 1 | 14/30 | | 6.07E9 |
| | 75.16 - 75.73 | -.NM*INVALNNGTLQHPVK.- | 1880.16 | 2 | 4.60 | 0.53 | 1659.2 | 1 | 21/32 | | 4.61E9 |
| | 79.99 | -.NM*INVALNNGTLQHPVK.- | 1880.16 | 2 | 3.39 | 0.47 | 1001.5 | 1 | 19/32 | | 1.55E9 |
| | 76.59 - 77.63 | -.NM*INVALNNGTLQHPVK.- | 1880.16 | 2 | 4.52 | 0.46 | 1295.8 | 1 | 21/32 | | 6.61E9 |
| | 88.64 | -.NMINVALNNGTLQHPVK.- | 1864.16 | 2 | 4.90 | 0.57 | 2084.9 | 1 | 23/32 | | 1.08E9 |
| | 70.03 - 71.31 | -.RNM*INVALNNGTLQHPVK.- | 2036.35 | 2 | 2.96 | 0.46 | 495.8 | 1 | 17/34 | | 3.00E9 |
| | 61.14 - 61.74 | -.SVEEILGK.- | 875.00 | 1 | 2.14 | 0.19 | 585.9 | 1 | 10/14 | | 3.06E9 |
| | 76.08 - 76.63 | -.VFM*QPASEGTGIIAGGAM*R.- | 1926.21 | 3 | 5.42 | 0.54 | 1785.1 | 1 | 35/72 | | 2.87E9 |
| | 75.98 - 77.17 | -.VFM*QPASEGTGIIAGGAM*R.- | 1926.21 | 2 | 5.51 | 0.62 | 1115.7 | 1 | 21/36 | | 1.67E10 |
| | 85.38 - 86.65 | -.VFM*QPASEGTGIIAGGAMR.- | 1910.21 | 2 | 4.53 | 0.33 | 730.0 | 1 | 19/36 | | 4.72E9 |
| | 95.05 | -.VFMQPASEGTGIIAGGAMR.- | 1894.21 | 2 | 2.94 | 0.47 | 279.2 | 3 | 14/36 | | 1.57E9 |
| #4 | RL9_ECOLI (P02418) 5 | | | | | 200.24 | | | 20 (20 0 0 0 0) | | 4.01 |
| | 95.90 - 96.49 | -.DIADAVTAAGVEVAK.- | 1430.59 | 1 | 1.88 | 0.46 | 395.2 | 1 | 12/28 | | 9.50E9 |
| | 95.86 - 96.72 | -.DIADAVTAAGVEVAK.- | 1430.59 | 2 | 4.15 | 0.59 | 1462.2 | 1 | 19/28 | | 1.00E10 |
| | 84.94 | -.INALETVTIASK.- | 1260.46 | 2 | 3.40 | 0.53 | 700.0 | 1 | 14/22 | | 6.12E9 |
| | 83.17 - 84.37 | -.INALETVTIASK.- | 1260.46 | 2 | 3.99 | 0.61 | 1296.6 | 1 | 19/22 | | 4.71E9 |
| | 69.67 | -.KNIEFFEAR.- | 1154.30 | 2 | 2.93 | 0.29 | 923.6 | 1 | 13/16 | | 1.36E9 |
| | 64.23 | -.LAEVLAAANAR.- | 1099.27 | 1 | 2.30 | 0.46 | 390.5 | 1 | 13/20 | | 2.52E9 |
| | 68.39 | -.LAEVLAAANAR.- | 1099.27 | 1 | 2.30 | 0.43 | 432.1 | 1 | 12/20 | | 1.96E9 |
| | 68.53 | -.LAEVLAAANAR.- | 1099.27 | 2 | 2.84 | 0.57 | 1434.9 | 1 | 17/20 | | 1.16E9 |
| | 59.13 | -.LAEVLAAANAR.- | 1099.27 | 2 | 2.50 | 0.46 | 1291.9 | 1 | 16/20 | | 9.09E8 |
| | 64.02 - 65.16 | -.LAEVLAAANAR.- | 1099.27 | 1 | 2.26 | 0.49 | 291.0 | 1 | 11/20 | | 3.84E9 |
| | 64.04 - 65.22 | -.LAEVLAAANAR.- | 1099.27 | 2 | 4.78 | 0.54 | 2096.4 | 1 | 19/20 | | 3.67E9 |
| | 63.33 - 63.91 | -.LFGSIGTR.- | 850.99 | 2 | 2.57 | 0.43 | 459.1 | 4 | 12/14 | | 1.38E9 |

| | | | | | | | | | | |
|---|---|---|---|---|---|---|---|---|---|---|
| | 63.31 - 63.96 | -.LFGSIGTR.- | 850.99 | 1 | 2.13 | 0.19 | 227.6 | 1 | 11/14 | 2.01E9 |
| | 84.35 | -.MQVILLDK.- | 960.22 | 1 | 1.98 | 0.10 | 745.8 | 1 | 10/14 | 1.35E9 |
| | 57.47 - 58.61 | -.NFLVPQGK.- | 903.06 | 1 | 1.82 | 0.16 | 333.8 | 1 | 10/14 | 5.00E9 |
| | 87.13 - 87.69 | -.NIEFFEAR.- | 1026.13 | 1 | 1.89 | 0.06 | 151.8 | 3 | 10/14 | 5.22E9 |
| | 87.31 | -.NIEFFEAR.- | 1026.13 | 2 | 2.72 | 0.21 | 628.0 | 1 | 12/14 | 1.98E9 |
| | 77.25 | -.TTGEHEVSFQVHSEVFAK.- | 2033.19 | 2 | 3.90 | 0.46 | 871.0 | 1 | 18/34 | 1.33E9 |
| | 80.10 - 80.64 | -.VANLGSLGDQVNVK.- | 1414.59 | 2 | 4.63 | 0.55 | 1340.9 | 1 | 20/26 | 1.37E10 |
| | 80.18 - 80.76 | -.VANLGSLGDQVNVK.- | 1414.59 | 1 | 2.50 | 0.48 | 210.2 | 4 | 11/26 | 8.82E9 |
| #5 | RS7_ECO57 (P66607) | | | | | 180.32 | | | 18 (18 0 0 0 0) | 4.41 |
| | 64.00 - 64.61 | -.FGSELLAK.- | 865.01 | 2 | 2.53 | 0.22 | 1336.9 | 1 | 14/14 | 1.32E9 |
| | 93.06 | -.FVNILM*VDGK.- | 1152.39 | 2 | 2.86 | 0.44 | 1080.1 | 1 | 16/18 | 1.50E9 |
| | 80.57 - 81.95 | -.FVNILM*VDGKK.- | 1280.56 | 2 | 3.34 | 0.50 | 1198.9 | 1 | 17/20 | 3.40E9 |
| | 135.18 - 135.79 | -.KSTAESIVYSALETLAQR.- | 1968.20 | 2 | 4.99 | 0.56 | 1329.8 | 1 | 21/34 | 1.94E9 |
| | 135.16 - 135.83 | -.KSTAESIVYSALETLAQR.- | 1968.20 | 3 | 4.16 | 0.23 | 2226.3 | 1 | 33/68 | 2.73E9 |
| | 54.78 | -.LANELSDAAENK.- | 1275.35 | 2 | 2.58 | 0.21 | 1058.3 | 1 | 15/22 | 8.14E8 |
| | 12.17 | -.LANELSDAAENK.- | 1275.35 | 2 | 4.07 | 0.55 | 1432.0 | 1 | 20/22 | 3.37E9 |
| | 11.82 - 12.63 | -.LANELSDAAENKGTAVK.- | 1731.89 | 2 | 5.15 | 0.56 | 1104.2 | 1 | 21/32 | 1.59E9 |
| | 66.35 | -.RVGGSTYQVPVEVR.- | 1547.74 | 2 | 3.40 | 0.49 | 334.6 | 1 | 14/26 | 1.22E9 |
| | 69.06 - 69.62 | -.RVGGSTYQVPVEVRPVR.- | 1900.18 | 3 | 4.10 | 0.45 | 1279.4 | 1 | 28/64 | 2.40E9 |
| | 128.24 - 128.98 | -.SELEAFEVALENVRPTVEVK.- | 2260.53 | 3 | 4.35 | 0.52 | 1154.7 | 1 | 31/76 | 1.58E10 |
| | 128.28 - 128.84 | -.SELEAFEVALENVRPTVEVK.- | 2260.53 | 2 | 5.06 | 0.54 | 1228.4 | 1 | 20/38 | 1.79E10 |
| | 129.87 | -.SELEAFEVALENVRPTVEVK.- | 2260.53 | 2 | 3.37 | 0.54 | 565.8 | 1 | 15/38 | 8.09E9 |
| | 123.49 - 123.82 | -.SGKSELEAFEVALENVRPTVEVK.- | 2532.83 | 2 | 3.96 | 0.54 | 836.8 | 1 | 17/44 | 2.25E9 |
| | 123.45 - 124.17 | -.SGKSELEAFEVALENVRPTVEVK.- | 2532.83 | 3 | 6.35 | 0.66 | 1989.7 | 1 | 40/88 | 7.91E9 |
| | 146.59 - 147.26 | -.STAESIVYSALETLAQR.- | 1840.03 | 3 | 5.12 | 0.42 | 2258.4 | 1 | 32/64 | 7.18E9 |
| | 146.61 - 147.22 | -.STAESIVYSALETLAQR.- | 1840.03 | 2 | 5.51 | 0.43 | 1873.5 | 1 | 21/32 | 1.05E10 |
| | 74.69 - 75.45 | -.VGGSTYQVPVEVRPVR.- | 1743.99 | 2 | 3.51 | 0.49 | 697.2 | 1 | 17/30 | 5.28E9 |
| #6 | RL22_ECO57 (P61177 | | | | | 170.43 | | | 17 (17 0 0 0 0) | 1.92 |
| | 13.19 - 14.42 | -.IFVDEGPSM*K.- | 1139.30 | 2 | 2.69 | 0.45 | 728.2 | 1 | 14/18 | 1.37E9 |
| | 85.94 | -.KVLESAIANAEHNDGADIDDLK.- | 2339.50 | 3 | 6.46 | 0.55 | 1871.3 | 1 | 38/84 | 2.01E9 |
| | 84.16 | -.KVLESAIANAEHNDGADIDDLKVTK.- | 2667.91 | 3 | 6.72 | 0.64 | 2985.4 | 1 | 41/96 | 1.08E9 |
| | 86.53 - 86.69 | -.KVLESAIANAEHNDGADIDDLKVTK.- | 2667.91 | 3 | 8.57 | 0.69 | 3956.8 | 1 | 42/96 | 2.12E9 |
| | 86.59 | -.KVLESAIANAEHNDGADIDDLKVTK.- | 2667.91 | 2 | 5.21 | 0.67 | 1092.4 | 1 | 23/48 | 1.09E9 |
| | 90.47 - 91.06 | -.KVSQALDILTYTNK.- | 1594.83 | 2 | 4.21 | 0.62 | 1644.2 | 1 | 19/26 | 2.55E9 |
| | 82.52 | -.KVSQALDILTYTNKK.- | 1723.01 | 3 | 4.94 | 0.56 | 1709.9 | 1 | 30/56 | 1.29E9 |
| | 81.60 - 82.29 | -.KVSQALDILTYTNKK.- | 1723.01 | 2 | 4.41 | 0.56 | 1821.7 | 1 | 19/28 | 2.18E9 |
| | 11.69 | -.TSHITVVVSDR.- | 1214.35 | 2 | 3.23 | 0.31 | 1182.5 | 1 | 18/20 | 2.83E9 |
| | 11.11 - 11.65 | -.TSHITVVVSDR.- | 1214.35 | 1 | 1.96 | 0.38 | 437.4 | 1 | 12/20 | 4.09E9 |
| | 89.61 - 89.99 | -.VLESAIANAEHNDGADIDDLK.- | 2211.33 | 2 | 4.88 | 0.65 | 1242.8 | 1 | 22/40 | 3.67E9 |
| | 86.47 - 87.23 | -.VLESAIANAEHNDGADIDDLK.- | 2211.33 | 2 | 5.23 | 0.60 | 943.6 | 1 | 21/40 | 2.31E9 |
| | 86.67 - 87.57 | -.VLESAIANAEHNDGADIDDLKVTK.- | 2539.74 | 3 | 5.17 | 0.53 | 927.3 | 1 | 29/92 | 2.54E9 |
| | 90.12 | -.VLESAIANAEHNDGADIDDLKVTK.- | 2539.74 | 2 | 3.34 | 0.47 | 725.8 | 1 | 19/46 | 1.95E9 |
| | 89.82 - 90.38 | -.VLESAIANAEHNDGADIDDLKVTK.- | 2539.74 | 3 | 4.94 | 0.58 | 1028.6 | 1 | 31/92 | 2.62E9 |
| | 97.10 - 97.61 | -.VSQALDILTYTNK.- | 1466.66 | 2 | 3.42 | 0.46 | 1159.4 | 1 | 16/24 | 3.90E9 |
| | 87.88 - 88.49 | -.VSQALDILTYTNKK.- | 1594.83 | 2 | 3.93 | 0.56 | 1550.0 | 1 | 20/26 | 3.77E9 |
| #7 | HNS_ECOLI (P08936) | | | | | 150.34 | | | 15 (15 0 0 0 0) | 3.40 |
| | 148.46 - 149.64 | -.ECTLETLEEM*LEKLEVVVNER.- | 2580.89 | 3 | 6.84 | 0.58 | 2366.3 | 1 | 33/80 | 6.21E9 |
| | 148.07 - 149.52 | -.ECTLETLEEM*LEKLEVVVNER.- | 2580.89 | 2 | 3.43 | 0.52 | 131.6 | 6 | 10/40 | 2.01E9 |
| | 167.04 | -.ECTLETLEEMLEKLEVVVNER.- | 2564.89 | 3 | 4.75 | 0.50 | 1330.0 | 1 | 29/80 | 2.32E9 |
| | 140.22 - 141.68 | -.EM*LIADGIDPNELLNSLAAVK.- | 2243.56 | 2 | 5.57 | 0.53 | 1356.6 | 1 | 26/40 | 2.15E10 |
| | 120.16 | -.EM*LIADGIDPNELLNSLAAVK.- | 2243.56 | 2 | 4.60 | 0.52 | 1046.7 | 1 | 20/40 | 1.13E9 |
| | 121.71 | -.EM*LIADGIDPNELLNSLAAVK.- | 2243.56 | 2 | 4.96 | 0.54 | 1464.7 | 1 | 24/40 | 2.70E9 |
| | 138.70 - 140.14 | -.EM*LIADGIDPNELLNSLAAVK.- | 2243.56 | 2 | 5.35 | 0.57 | 1200.9 | 1 | 24/40 | 2.48E10 |
| | 147.32 - 148.76 | -.EMLIADGIDPNELLNSLAAVK.- | 2227.56 | 2 | 4.05 | 0.57 | 227.0 | 3 | 11/40 | 4.55E9 |
| | 129.10 | -.EMLIADGIDPNELLNSLAAVK.- | 2227.56 | 2 | 5.13 | 0.60 | 905.2 | 1 | 19/40 | 1.52E9 |
| | 19.03 - 20.68 | -.LEVVVNER.- | 958.09 | 2 | 3.10 | 0.34 | 767.8 | 1 | 12/14 | 2.32E8 |
| | 23.79 - 25.42 | -.LEVVVNER.- | 958.09 | 2 | 2.92 | 0.36 | 595.8 | 1 | 11/14 | 2.66E8 |
| | 21.25 - 22.98 | -.LEVVVNER.- | 958.09 | 2 | 3.06 | 0.34 | 725.0 | 1 | 12/14 | 2.56E8 |
| | 41.97 | -.REEESAAAAEVEER.- | 1576.61 | 2 | 3.21 | 0.48 | 796.2 | 1 | 15/26 | 2.23E8 |
| | 97.86 - 98.46 | -.SLDDFLIKQ.- | 1079.23 | 2 | 2.76 | 0.26 | 1031.6 | 1 | 14/16 | 2.78E9 |
| | 98.27 | -.SLDDFLIKQ.- | 1079.23 | 1 | 1.97 | 0.20 | 447.0 | 1 | 11/16 | 2.93E9 |
| #8 | RS13_ECOLI (P02369) | | | | | 140.25 | | | 14 (14 0 0 0 0) | 2.99 |
| | 190.48 | -.AILAAAGIAEDVK.- | 1242.45 | 2 | 3.00 | 0.55 | 1425.4 | 1 | 18/24 | 3.27E8 |
| | 88.15 - 88.83 | -.AILAAAGIAEDVK.- | 1242.45 | 2 | 4.95 | 0.63 | 2245.7 | 1 | 21/24 | 4.30E9 |
| | 87.94 - 89.38 | -.AILAAAGIAEDVK.- | 1242.45 | 1 | 2.52 | 0.51 | 648.8 | 1 | 14/24 | 5.83E9 |
| | 91.22 | -.HAVIALTSIYGVGK.- | 1429.69 | 1 | 2.14 | 0.36 | 743.1 | 1 | 16/26 | 2.73E9 |
| | 91.38 | -.HAVIALTSIYGVGK.- | 1429.69 | 1 | 3.82 | 0.60 | 833.6 | 1 | 18/26 | 2.07E9 |
| | 91.14 - 91.75 | -.HAVIALTSIYGVGK.- | 1429.69 | 2 | 4.96 | 0.70 | 2266.8 | 1 | 22/26 | 4.63E9 |
| | 64.80 - 67.28 | -.IAGINIPDHK.- | 1078.25 | 1 | 1.82 | 0.35 | 301.5 | 3 | 10/18 | 3.55E9 |
| | 64.88 - 66.18 | -.IAGINIPDHK.- | 1078.25 | 2 | 3.71 | 0.52 | 976.3 | 1 | 15/18 | 2.80E9 |
| | 65.39 | -.IAGINIPDHK.- | 1078.25 | 1 | 2.43 | 0.42 | 623.7 | 1 | 13/18 | 1.99E9 |
| | 81.45 | -.ISELSEGQIDTLR.- | 1461.60 | 2 | 3.84 | 0.52 | 1317.1 | 1 | 17/24 | 2.14E9 |
| | 89.34 | -.ISELSEGQIDTLRDEVAK.- | 2004.19 | 2 | 3.20 | 0.46 | 432.9 | 2 | 14/34 | 1.68E9 |
| | 93.21 - 94.52 | -.ISELSEGQIDTLRDEVAK.- | 2004.19 | 2 | 4.68 | 0.55 | 1570.1 | 1 | 21/34 | 2.03E10 |
| | 93.23 - 93.82 | -.ISELSEGQIDTLRDEVAK.- | 2004.19 | 3 | 3.38 | 0.36 | 928.4 | 1 | 30/68 | 1.15E10 |
| | 50.63 - 51.79 | -.LM*DLGCYR.- | 1044.20 | 2 | 2.52 | 0.35 | 535.3 | 1 | 11/14 | 6.46E8 |
| #9 | RS6_ECOL6 (Q8XDI1) | | | | | 130.34 | | | 13 (13 0 0 0 0) | 2.29 |
| | 142.95 - 143.51 | -.AHYVLM*NVEAPQEVIDELETTFR.- | 2722.02 | 2 | 5.31 | 0.56 | 856.6 | 1 | 22/44 | 6.24E9 |
| | 142.84 - 144.01 | -.AHYVLM*NVEAPQEVIDELETTFR.- | 2722.02 | 3 | 6.74 | 0.69 | 2620.9 | 1 | 38/88 | 2.10E10 |
| | 151.85 - 153.12 | -.AHYVLMNVEAPQEVIDELETTFR.- | 2706.02 | 3 | 5.64 | 0.50 | 1453.2 | 1 | 30/88 | 3.20E9 |
| | 152.05 | -.AHYVLMNVEAPQEVIDELETTFR.- | 2706.02 | 2 | 3.05 | 0.56 | 817.8 | 1 | 20/44 | 1.06E9 |
| | 15.39 - 16.65 | -.FNDAVIR.- | 834.94 | 2 | 2.51 | 0.30 | 728.3 | 1 | 11/12 | 3.90E8 |
| | 18.63 - 19.97 | -.FNDAVIR.- | 834.94 | 2 | 2.57 | 0.34 | 805.7 | 1 | 11/12 | 2.77E8 |
| | 89.67 - 90.22 | -.HYEIVFM*VHPDQSEQVPGM*IER.- | 2674.99 | 3 | 4.34 | 0.58 | 1488.1 | 1 | 33/84 | 4.76E9 |

| | | | | | | | | | | |
|---|---|---|---|---|---|---|---|---|---|---|
| | 96.83 - 97.67 | -.HYEIVFM*VHPDQSEQVPGMIER.- | 2658.99 | 3 | 3.93 | 0.22 | 2311.5 | 1 | 35/84 | 2.19E9 |
| | 85.19 | -.M*RHYEIVFM*VHPDQSEQVPGM*IER.- | 2978.38 | 3 | 4.25 | 0.59 | 792.6 | 1 | 29/92 | 1.41E9 |
| | 54.76 | -.RDDFANETADDAEAGDSEE.- | 2057.93 | 2 | 2.79 | 0.31 | 666.5 | 1 | 16/36 | 8.51E8 |
| | 52.88 - 53.69 | -.RDDFANETADDAEAGDSEE.- | 2057.93 | 2 | 5.47 | 0.58 | 2289.6 | 1 | 24/36 | 1.32E9 |
| | 51.02 - 52.31 | -.RDDFANETADDAEAGDSEE.- | 2057.93 | 2 | 4.18 | 0.53 | 1181.9 | 1 | 20/36 | 8.55E8 |
| | 9.81 - 11.30 | -.YTAAITGAEGK.- | 1082.19 | 1 | 1.99 | 0.34 | 468.3 | 1 | 12/20 | 6.02E9 |
| #10 | RL13_ECOLI (P02410) | | | | | 130.31 | | | 13 (13 0 0 0 0) | 2.21 |
| | 102.62 - 103.78 | -.AEYTPHVDTGDYIIVLNADK.- | 2235.44 | 3 | 3.50 | 0.25 | 1479.3 | 1 | 30/76 | 5.44E9 |
| | 102.53 - 103.66 | -.AEYTPHVDTGDYIIVLNADK.- | 2235.44 | 2 | 6.25 | 0.68 | 2675.9 | 1 | 27/38 | 6.24E9 |
| | 105.89 | -.AEYTPHVDTGDYIIVLNADK.- | 2235.44 | 2 | 3.79 | 0.43 | 962.6 | 1 | 18/38 | 1.71E9 |
| | 84.73 | -.DWYVVDATGK.- | 1154.25 | 2 | 3.06 | 0.28 | 1204.9 | 1 | 16/18 | 1.54E9 |
| | 84.47 - 85.71 | -.DWYVVDATGK.- | 1154.25 | 1 | 2.11 | 0.18 | 715.8 | 1 | 12/18 | 6.14E9 |
| | 83.67 - 84.71 | -.DWYVVDATGK.- | 1154.25 | 1 | 2.83 | 0.47 | 887.0 | 1 | 13/18 | 3.46E9 |
| | 89.80 - 90.34 | -.HKAEYTPHVDTGDYIIVLNADK.- | 2500.75 | 2 | 4.77 | 0.54 | 865.4 | 1 | 20/42 | 3.12E9 |
| | 89.84 - 90.45 | -.HKAEYTPHVDTGDYIIVLNADK.- | 2500.75 | 3 | 5.34 | 0.70 | 1640.9 | 1 | 36/84 | 3.80E9 |
| | 62.87 - 63.47 | -.QATFEEM*IAR.- | 1212.36 | 2 | 2.89 | 0.50 | 861.4 | 1 | 16/18 | 2.23E9 |
| | 30.70 | -.TDKVYYHHTGHIGGIK.- | 1827.04 | 2 | 2.82 | 0.59 | 604.5 | 1 | 13/30 | 4.12E8 |
| | 75.68 - 76.69 | -.VYAGNEHNHAAQQPQVLDI.- | 2105.26 | 2 | 2.80 | 0.51 | 225.7 | 1 | 13/36 | 6.01E9 |
| | 80.70 - 81.98 | -.VYAGNEHNHAAQQPQVLDI.- | 2105.26 | 2 | 3.36 | 0.53 | 575.9 | 1 | 16/36 | 4.94E9 |
| | 82.75 | -.VYAGNEHNHAAQQPQVLDI.- | 2105.26 | 2 | 2.78 | 0.58 | 324.3 | 1 | 15/36 | 2.62E9 |
| #11 | RL10_ECOLI (P02408) | | | | | 130.24 | | | 13 (13 0 0 0 0) | 5.34 |
| | 96.24 | -.AAAFEGELIPASQIDR.- | 1688.86 | 2 | 4.43 | 0.44 | 1036.9 | 1 | 21/30 | 2.55E10 |
| | 95.92 | -.AAAFEGELIPASQIDR.- | 1688.86 | 1 | 2.25 | 0.09 | 311.3 | 2 | 13/30 | 3.52E9 |
| | 95.80 | -.AAAFEGELIPASQIDR.- | 1688.86 | 1 | 2.52 | 0.30 | 215.1 | 7 | 10/30 | 5.27E9 |
| | 94.50 - 95.79 | -.AAAFEGELIPASQIDR.- | 1688.86 | 2 | 3.22 | 0.58 | 731.7 | 1 | 18/30 | 2.86E10 |
| | 130.68 - 131.79 | -.AVEGTPFECLKDAFVGPTLIAYSM*EHP( | 3423.83 | 3 | 3.13 | 0.27 | 320.2 | 26 | 21/124 | 3.32E9 |
| | 99.34 - 100.52 | -.DAFVGPTLIAYSM*EHPGAAAR.- | 2191.45 | 2 | 4.70 | 0.56 | 409.7 | 1 | 15/40 | 9.00E9 |
| | 115.39 - 115.97 | -.DAFVGPTLIAYSMEHPGAAAR.- | 2175.45 | 2 | 4.33 | 0.60 | 817.2 | 1 | 18/40 | 2.64E9 |
| | 62.58 | -.GALSAVVADSR.- | 1046.16 | 1 | 2.55 | 0.49 | 251.6 | 1 | 12/20 | 1.54E9 |
| | 61.70 - 62.85 | -.GALSAVVADSR.- | 1046.16 | 2 | 3.66 | 0.59 | 1550.4 | 1 | 16/20 | 3.23E9 |
| | 61.62 - 62.74 | -.GALSAVVADSR.- | 1046.16 | 1 | 2.39 | 0.48 | 198.4 | 2 | 11/20 | 3.09E9 |
| | 91.04 - 92.05 | -.LATLPTYEEAIAR.- | 1448.65 | 2 | 3.10 | 0.55 | 374.1 | 1 | 16/24 | 1.38E10 |
| | 79.84 - 81.14 | -.QAIVAEVSEVAK.- | 1244.42 | 2 | 3.50 | 0.52 | 1341.3 | 1 | 19/22 | 1.19E10 |
| | 69.46 - 70.24 | -.RAVEGTPFECLK.- | 1407.59 | 2 | 3.24 | 0.42 | 1008.9 | 1 | 15/22 | 3.83E9 |
| #12 | RL14_ECOLI (P02411) | | | | | 120.31 | | | 12 (12 0 0 0 0) | 2.27 |
| | 87.82 - 88.95 | -.FDGNACVLLNNNSEQPIGTR.- | 2220.38 | 2 | 5.21 | 0.53 | 1070.2 | 1 | 22/38 | 9.09E9 |
| | 89.55 - 90.61 | -.FDGNACVLLNNNSEQPIGTR.- | 2220.38 | 2 | 4.56 | 0.47 | 1076.0 | 1 | 21/38 | 9.81E9 |
| | 91.86 - 93.25 | -.FDGNACVLLNNNSEQPIGTR.- | 2220.38 | 2 | 4.51 | 0.55 | 1023.8 | 1 | 21/38 | 4.45E9 |
| | 94.32 - 94.87 | -.FDGNACVLLNNNSEQPIGTR.- | 2220.38 | 2 | 5.32 | 0.62 | 1122.8 | 1 | 21/38 | 2.61E9 |
| | 55.64 - 56.78 | -.M*IQEQTM*LNVADNSGAR.- | 1911.11 | 2 | 4.78 | 0.60 | 1135.5 | 1 | 20/32 | 2.01E9 |
| | 65.75 - 66.45 | -.M*IQEQTM*LNVADNSGAR.- | 1911.11 | 2 | 2.71 | 0.37 | 291.8 | 5 | 12/32 | 1.70E9 |
| | 53.87 - 55.06 | -.M*IQEQTM*LNVADNSGAR.- | 1911.11 | 2 | 5.62 | 0.56 | 2034.2 | 1 | 23/32 | 4.04E9 |
| | 52.90 - 54.12 | -.M*IQEQTM*LNVADNSGAR.- | 1911.11 | 3 | 3.12 | 0.36 | 1268.3 | 1 | 29/64 | 6.92E8 |
| | 52.04 - 53.29 | -.M*IQEQTM*LNVADNSGAR.- | 1911.11 | 2 | 6.15 | 0.63 | 2377.3 | 1 | 24/32 | 2.88E9 |
| | 65.54 - 66.22 | -.MIQEQTM*LNVADNSGAR.- | 1895.11 | 2 | 3.01 | 0.28 | 795.4 | 1 | 15/32 | 1.02E9 |
| | 63.60 - 64.13 | -.RYAGVGDIIK.- | 1092.27 | 1 | 1.98 | 0.17 | 381.8 | 1 | 14/18 | 1.03E9 |
| | 75.05 - 76.19 | -.YAGVGDIIK.- | 936.09 | 1 | 2.12 | 0.27 | 308.7 | 4 | 9/16 | 9.64E9 |
| #13 | CH10_ECOLI (P05380) | | | | | 120.24 | | | 12 (12 0 0 0 0) | 2.45 |
| | 12.34 | -.GEVLAVGNGR.- | 972.08 | 1 | 1.91 | 0.41 | 309.2 | 1 | 11/18 | 2.61E9 |
| | 72.86 - 73.60 | -.ILENGEVKPLDVK.- | 1454.69 | 2 | 3.72 | 0.31 | 1016.7 | 1 | 18/24 | 4.03E9 |
| | 70.79 - 72.42 | -.ILENGEVKPLDVK.- | 1454.69 | 1 | 2.77 | 0.45 | 564.4 | 1 | 15/24 | 4.47E9 |
| | 70.69 - 71.25 | -.ILENGEVKPLDVK.- | 1454.69 | 2 | 3.91 | 0.39 | 1328.4 | 1 | 19/24 | 9.45E9 |
| | 44.50 - 46.00 | -.SAGGIVLTGSAAAK.- | 1203.37 | 2 | 4.04 | 0.59 | 1086.9 | 1 | 21/26 | 1.66E9 |
| | 42.72 - 43.94 | -.SAGGIVLTGSAAAK.- | 1203.37 | 2 | 4.26 | 0.67 | 1205.2 | 1 | 21/26 | 1.75E9 |
| | 40.96 - 42.12 | -.SAGGIVLTGSAAAK.- | 1203.37 | 2 | 4.17 | 0.60 | 1217.5 | 1 | 22/26 | 1.79E9 |
| | 39.21 - 40.61 | -.SAGGIVLTGSAAAK.- | 1203.37 | 2 | 3.90 | 0.62 | 1025.2 | 1 | 20/26 | 9.02E8 |
| | 46.51 - 47.58 | -.SAGGIVLTGSAAAK.- | 1203.37 | 2 | 3.92 | 0.63 | 1104.5 | 1 | 21/26 | 7.80E8 |
| | 103.57 - 104.98 | -.VGDIVIFNDGYGVK.- | 1496.69 | 2 | 3.83 | 0.57 | 1032.8 | 1 | 19/26 | 8.56E9 |
| | 101.81 - 102.39 | -.VGDIVIFNDGYGVK.- | 1496.69 | 2 | 4.78 | 0.59 | 1097.7 | 1 | 20/26 | 1.40E10 |
| | 101.48 - 102.08 | -.VGDIVIFNDGYGVK.- | 1496.69 | 1 | 2.02 | 0.41 | 142.3 | 35 | 9/26 | 2.88E9 |
| #14 | RS11_ECOLI (P02366) | | | | | 110.29 | | | 11 (11 0 0 0 0) | 1.57 |
| | 73.33 - 74.34 | -.ITNITDVTPIPHNGCRPPK.- | 2131.42 | 2 | 3.30 | 0.65 | 536.4 | 1 | 15/36 | 5.61E9 |
| | 74.92 | -.ITNITDVTPIPHNGCRPPK.- | 2131.42 | 2 | 3.51 | 0.56 | 876.1 | 1 | 20/36 | 6.01E9 |
| | 93.97 - 94.56 | -.KQVSDGVAHIHASFNNTIVTITDR.- | 2624.89 | 3 | 5.89 | 0.49 | 1821.0 | 1 | 34/92 | 3.50E9 |
| | 44.73 - 45.75 | -.KSTPFAAQVAAER.- | 1376.54 | 2 | 3.44 | 0.32 | 792.1 | 1 | 16/24 | 1.92E9 |
| | 44.19 - 44.88 | -.KSTPFAAQVAAER.- | 1376.54 | 3 | 3.44 | 0.28 | 1232.9 | 1 | 27/48 | 4.86E8 |
| | 42.87 - 44.13 | -.KSTPFAAQVAAER.- | 1376.54 | 2 | 3.61 | 0.33 | 747.5 | 1 | 17/24 | 1.11E9 |
| | 40.92 - 42.24 | -.KSTPFAAQVAAER.- | 1376.54 | 2 | 2.78 | 0.34 | 751.2 | 1 | 15/24 | 8.29E8 |
| | 39.36 - 40.11 | -.KSTPFAAQVAAER.- | 1376.54 | 2 | 3.00 | 0.48 | 831.7 | 1 | 16/24 | 3.61E8 |
| | 12.23 | -.NLEVM*VK.- | 849.03 | 1 | 2.07 | 0.41 | 72.1 | 42 | 7/12 | 3.38E9 |
| | 90.91 - 92.17 | -.QGNALGWATAGGSGFR.- | 1550.66 | 2 | 3.54 | 0.62 | 943.7 | 1 | 19/30 | 5.58E9 |
| | 68.02 - 68.58 | -.STPFAAQVAAER.- | 1248.37 | 2 | 3.54 | 0.53 | 852.7 | 1 | 18/22 | 5.01E9 |
| #15 | RL19_ECOLI (P02420) | | | | | 110.24 | | | 11 (11 0 0 0 0) | 3.47 |
| | 13.84 - 15.24 | -.GLHSAFTVR.- | 988.13 | 2 | 2.72 | 0.43 | 763.2 | 1 | 15/16 | 7.95E8 |
| | 13.58 - 15.75 | -.GLHSAFTVR.- | 988.13 | 1 | 1.81 | 0.37 | 409.1 | 1 | 12/16 | 1.19E9 |
| | 112.98 | -.LQAFEGVVIAIR.- | 1316.57 | 2 | 2.62 | 0.44 | 1112.1 | 1 | 17/22 | 2.05E9 |
| | 74.84 - 75.43 | -.QDVPSFRPGDTVEVK.- | 1674.84 | 2 | 3.27 | 0.34 | 514.7 | 1 | 16/28 | 2.78E9 |
| | 74.11 - 74.71 | -.QLEQEQM*KQDVPSFRPGDTVEVK.- | 2705.98 | 3 | 3.25 | 0.26 | 391.0 | 10 | 22/88 | 4.56E9 |
| | 85.44 | -.QLEQEQMKQDVPSFRPGDTVEVK.- | 2689.98 | 2 | 2.68 | 0.44 | 218.7 | 1 | 14/44 | 2.61E9 |
| | 77.46 - 78.77 | -.VFQTHSPVVDSISVK.- | 1643.87 | 1 | 2.78 | 0.43 | 363.4 | 1 | 16/28 | 3.33E9 |
| | 76.81 - 78.03 | -.VFQTHSPVVDSISVK.- | 1643.87 | 2 | 4.81 | 0.60 | 1662.7 | 1 | 22/28 | 4.02E10 |
| | 77.15 - 78.35 | -.VFQTHSPVVDSISVK.- | 1643.87 | 3 | 3.58 | 0.47 | 1750.4 | 1 | 29/56 | 1.18E10 |
| | 74.46 - 75.77 | -.VFQTHSPVVDSISVK.- | 1643.87 | 2 | 3.49 | 0.58 | 783.4 | 1 | 15/28 | 3.77E9 |
| | 37.28 - 44.06 | -.VWVVEGSK.- | 904.05 | 1 | 2.75 | 0.51 | 807.5 | 1 | 11/14 | 1.94E9 |

| | | | | | | | | | | |
|---|---|---|---|---|---|---|---|---|---|---|
| #16 | ACP_ECOLI (P02901) | | | | | 100.22 | | | 10 (10 0 0 0 0) | 1.42 |
| | 61.38 - 62.51 | -.IIGEQLGVK.- | 957.15 | 2 | 2.76 | 0.05 | 396.4 | 1 | 14/16 | 2.12E9 |
| | 112.60 | -.ITTVQAAIDYINGHQA.- | 1715.89 | 2 | 3.99 | 0.55 | 1377.8 | 1 | 20/30 | 1.98E9 |
| | 103.32 | -.ITTVQAAIDYINGHQA.- | 1715.89 | 1 | 3.23 | 0.46 | 465.9 | 1 | 15/30 | 1.32E9 |
| | 103.23 | -.ITTVQAAIDYINGHQA.- | 1715.89 | 1 | 3.33 | 0.47 | 616.7 | 1 | 17/30 | 1.91E9 |
| | 103.24 | -.ITTVQAAIDYINGHQA.- | 1715.89 | 3 | 3.43 | 0.37 | 1852.6 | 1 | 34/60 | 2.30E9 |
| | 121.82 - 122.76 | -.ITTVQAAIDYINGHQA.- | 1715.89 | 2 | 4.37 | 0.53 | 1983.4 | 1 | 21/30 | 3.77E9 |
| | 103.55 - 104.18 | -.ITTVQAAIDYINGHQA.- | 1715.89 | 2 | 3.96 | 0.40 | 1053.2 | 1 | 16/30 | 1.25E10 |
| | 108.21 | -.ITTVQAAIDYINGHQA.- | 1715.89 | 2 | 3.10 | 0.52 | 811.6 | 1 | 14/30 | 3.17E9 |
| | 27.63 - 29.39 | -.KIIGEQLGVK.- | 1085.32 | 2 | 2.83 | 0.33 | 637.5 | 1 | 14/18 | 9.22E8 |
| | 27.65 - 30.54 | -.KIIGEQLGVK.- | 1085.32 | 1 | 2.12 | 0.20 | 529.1 | 1 | 12/18 | 6.11E8 |
| #17 | RS8_ECOLI (P02361) 3 | | | | | 90.36 | | | 9 (9 0 0 0 0) | 1.63 |
| | 113.07 - 113.70 | -.EEGFIEDFKVEGDTKPELELTLK.- | 2667.95 | 3 | 4.04 | 0.51 | 770.1 | 1 | 28/88 | 2.52E9 |
| | 67.36 - 68.47 | -.SM*QDPIADM*LTR.- | 1410.60 | 2 | 3.80 | 0.52 | 795.9 | 1 | 17/22 | 5.44E9 |
| | 125.82 - 127.01 | -.VAIANVLKEEGFIEDFKVEGDTKPELELT | 3476.96 | 3 | 7.12 | 0.59 | 3267.3 | 1 | 45/120 | 7.19E9 |
| | 127.71 | -.VAIANVLKEEGFIEDFKVEGDTKPELELT | 3476.96 | 3 | 3.72 | 0.48 | 931.6 | 1 | 30/120 | 2.06E9 |
| | 85.73 - 85.87 | -.VM*AGLGIAVVSTSK.- | 1349.62 | 2 | 2.57 | 0.49 | 979.9 | 1 | 17/26 | 1.60E9 |
| | 80.59 - 81.24 | -.VM*AGLGIAVVSTSK.- | 1349.62 | 1 | 2.56 | 0.42 | 113.9 | 1 | 14/26 | 3.85E9 |
| | 80.14 - 81.16 | -.VM*AGLGIAVVSTSK.- | 1349.62 | 2 | 3.97 | 0.58 | 1725.9 | 1 | 22/26 | 5.47E9 |
| | 83.56 - 84.14 | -.VM*AGLGIAVVSTSK.- | 1349.62 | 2 | 2.65 | 0.43 | 714.1 | 1 | 15/26 | 1.95E9 |
| | 81.74 - 82.64 | -.VM*AGLGIAVVSTSK.- | 1349.62 | 2 | 4.34 | 0.58 | 1572.4 | 1 | 20/26 | 5.06E9 |
| #18 | RL15_ECO57 (P66072 | | | | | 90.29 | | | 9 (9 0 0 0 0) | 2.59 |
| | 12.71 | -.AAIEAAGGKIEE.- | 1159.27 | 1 | 2.09 | 0.27 | 740.9 | 1 | 15/22 | 1.58E9 |
| | 111.56 - 112.62 | -.AANIIGIQIEFAK.- | 1388.64 | 2 | 2.61 | 0.46 | 908.8 | 1 | 15/24 | 4.56E9 |
| | 111.73 | -.AANIIGIQIEFAK.- | 1388.64 | 1 | 2.13 | 0.29 | 357.5 | 14 | 10/24 | 2.46E9 |
| | 58.63 - 59.74 | -.GFEGGQM*PLYR.- | 1271.43 | 2 | 3.41 | 0.51 | 947.7 | 1 | 15/20 | 3.26E9 |
| | 9.76 - 11.32 | -.LNTLSPAEGSK.- | 1117.24 | 1 | 2.25 | 0.45 | 461.9 | 1 | 13/20 | 3.17E9 |
| | 81.51 - 82.27 | -.VEGGVVDLNTLK.- | 1244.42 | 2 | 3.47 | 0.49 | 1011.8 | 1 | 18/22 | 8.86E9 |
| | 88.37 | -.VILAGEVTTPVTVR.- | 1455.73 | 2 | 5.84 | 0.63 | 2200.2 | 1 | 21/26 | 1.30E10 |
| | 87.84 - 88.60 | -.VILAGEVTTPVTVR.- | 1455.73 | 1 | 2.85 | 0.49 | 186.9 | 1 | 14/26 | 4.66E9 |
| | 86.55 - 87.80 | -.VILAGEVTTPVTVR.- | 1455.73 | 2 | 4.99 | 0.57 | 1816.4 | 1 | 21/26 | 1.44E10 |
| #19 | OSME_ECOLI (P23933 | | | | | 90.26 | | | 9 (9 0 0 0 0) | 0.50 |
| | 62.80 | -.AQVAQIAGKPSSEVSM*IHAR.- | 2097.39 | 2 | 3.13 | 0.52 | 267.6 | 1 | 14/38 | 1.31E9 |
| | 62.70 | -.AQVAQIAGKPSSEVSM*IHAR.- | 2097.39 | 3 | 5.14 | 0.68 | 1860.6 | 1 | 35/76 | 1.36E9 |
| | 64.38 | -.AQVAQIAGKPSSEVSM*IHAR.- | 2097.39 | 2 | 3.80 | 0.53 | 256.7 | 1 | 14/38 | 1.33E9 |
| | 70.47 | -.AQVAQIAGKPSSEVSMIHAR.- | 2081.39 | 2 | 4.12 | 0.53 | 421.2 | 1 | 18/38 | 1.11E9 |
| | 70.61 | -.AQVAQIAGKPSSEVSMIHAR.- | 2081.39 | 3 | 3.77 | 0.41 | 1494.4 | 1 | 32/76 | 1.21E9 |
| | 59.43 - 60.72 | -.DQFVQPVVK.- | 1060.23 | 1 | 2.31 | 0.24 | 358.2 | 1 | 11/16 | 1.39E9 |
| | 60.47 - 61.66 | -.GTCQTYILGQR.- | 1297.44 | 2 | 2.89 | 0.49 | 1218.8 | 1 | 14/20 | 1.68E9 |
| | 15.79 - 17.34 | -.TKDQFVQPVVK.- | 1289.51 | 2 | 3.13 | 0.48 | 1205.3 | 1 | 16/20 | 3.76E8 |
| | 13.75 - 15.12 | -.TKDQFVQPVVK.- | 1289.51 | 2 | 3.26 | 0.47 | 970.4 | 1 | 15/20 | 1.07E9 |
| #20 | RISB_ECO57 (P61717 | | | | | 90.25 | | | 9 (9 0 0 0 0) | 0.90 |
| | 147.74 | -.FNNFINDSLLEGAIDALK.- | 1995.22 | 2 | 4.59 | 0.56 | 951.2 | 1 | 19/34 | 1.42E9 |
| | 149.43 - 150.01 | -.FNNFINDSLLEGAIDALK.- | 1995.22 | 2 | 4.09 | 0.56 | 968.2 | 1 | 20/34 | 9.92E8 |
| | 143.11 | -.FNNFINDSLLEGAIDALKR.- | 2151.41 | 2 | 2.62 | 0.37 | 83.6 | 124 | 10/36 | 1.52E9 |
| | 140.24 - 140.83 | -.FNNFINDSLLEGAIDALKR.- | 2151.41 | 3 | 3.71 | 0.33 | 707.0 | 1 | 28/72 | 3.32E9 |
| | 140.33 - 141.35 | -.FNNFINDSLLEGAIDALKR.- | 2151.41 | 2 | 4.03 | 0.51 | 834.9 | 1 | 17/36 | 2.05E9 |
| | 142.86 | -.FNNFINDSLLEGAIDALKR.- | 2151.41 | 3 | 5.04 | 0.29 | 1216.8 | 1 | 34/72 | 1.46E9 |
| | 127.57 | -.GAEAALTALEM*INVLK.- | 1660.96 | 2 | 4.35 | 0.48 | 1296.9 | 1 | 18/30 | 2.90E9 |
| | 134.87 - 135.59 | -.IGQVKDENITVVWVPGAYELPLAAGALAI | 3024.50 | 2 | 3.38 | 0.46 | 187.9 | 1 | 13/56 | 1.59E9 |
| | 72.29 - 72.98 | -.M*NIIEANVATPDAR.- | 1531.72 | 2 | 2.80 | 0.37 | 279.1 | 1 | 16/26 | 4.21E9 |
| #21 | RL17_ECOLI (P02416) | | | | | 90.20 | | | 9 (9 0 0 0 0) | 2.43 |
| | 97.15 - 98.19 | -.AGDNAPM*AYIELVDR.- | 1651.82 | 2 | 3.90 | 0.49 | 587.6 | 1 | 18/28 | 1.48E10 |
| | 98.76 - 99.38 | -.AGDNAPM*AYIELVDR.- | 1651.82 | 2 | 3.02 | 0.44 | 519.2 | 1 | 17/28 | 9.64E9 |
| | 111.24 | -.AGDNAPMAYIELVDR.- | 1635.82 | 2 | 3.52 | 0.42 | 724.7 | 1 | 20/28 | 3.10E9 |
| | 71.21 - 71.88 | -.LFNELGPR.- | 946.09 | 2 | 2.62 | 0.42 | 737.1 | 2 | 13/14 | 2.13E9 |
| | 71.14 - 71.70 | -.LFNELGPR.- | 946.09 | 1 | 2.41 | 0.35 | 346.4 | 1 | 11/14 | 5.59E9 |
| | 80.01 - 80.53 | -.RVVEPLITLAK.- | 1239.53 | 2 | 3.41 | 0.43 | 1577.2 | 1 | 17/20 | 4.38E9 |
| | 91.03 - 92.25 | -.VVEPLITLAK.- | 1083.35 | 1 | 2.46 | 0.30 | 686.0 | 1 | 12/18 | 6.25E9 |
| | 91.20 - 91.90 | -.VVEPLITLAK.- | 1083.35 | 2 | 3.10 | 0.51 | 850.3 | 1 | 15/18 | 3.12E9 |
| | 91.37 - 92.01 | -.VVEPLITLAK.- | 1083.35 | 1 | 2.57 | 0.55 | 785.8 | 1 | 12/18 | 3.37E9 |
| #22 | Q8X5P5 (Q8X5P5) Out | | | | | 70.32 | | | 7 (7 0 0 0 0) | 2.64 |
| | 111.16 - 112.33 | -.NHFVTILGTIQGEQPGFINK.- | 2214.51 | 2 | 6.42 | 0.66 | 1803.6 | 1 | 25/38 | 4.90E9 |
| | 119.62 | -.NHFVTILGTIQGEQPGFINK.- | 2214.51 | 2 | 3.60 | 0.51 | 554.4 | 1 | 15/38 | 1.51E9 |
| | 76.06 - 77.10 | -.SFVAVHNQPGLYVGQQAR.- | 1972.20 | 2 | 5.26 | 0.56 | 1225.2 | 1 | 23/34 | 1.00E10 |
| | 135.87 - 137.30 | -.TDTLLEIAVLPLDSYAKPDIEANYQGR.- | 3007.34 | 3 | 4.61 | 0.64 | 909.4 | 1 | 34/104 | 1.59E10 |
| | 137.06 - 137.65 | -.TDTLLEIAVLPLDSYAKPDIEANYQGR.- | 3007.34 | 2 | 4.10 | 0.60 | 516.0 | 1 | 19/52 | 3.19E9 |
| | 137.42 - 138.61 | -.TDTLLEIAVLPLDSYAKPDIEANYQGR.- | 3007.34 | 3 | 3.82 | 0.45 | 783.1 | 1 | 30/104 | 1.29E10 |
| | 121.35 - 122.50 | -.VPYNFLEVNM*QGIQVWHLR.- | 2360.72 | 3 | 5.27 | 0.51 | 2385.0 | 1 | 35/72 | 8.56E9 |
| #23 | HLPA_ECOLI (P11457 | | | | | 70.28 | | | 7 (7 0 0 0 0) | 1.62 |
| | 71.72 - 73.18 | -.DITADVLK.- | 875.00 | 1 | 1.94 | 0.09 | 375.3 | 7 | 9/14 | 5.75E9 |
| | 100.75 - 101.39 | -.IAIVNM*GSLFQQVAQK.- | 1764.08 | 2 | 5.52 | 0.50 | 1541.6 | 1 | 22/30 | 6.26E9 |
| | 125.86 | -.IAIVNMGSLFQQVAQK.- | 1748.08 | 2 | 5.05 | 0.66 | 1750.0 | 1 | 21/30 | 3.06E9 |
| | 127.72 - 128.86 | -.SVANSQDIDLVVDANAVAYNSSDVKDIT/ | 3451.74 | 3 | 3.08 | 0.44 | 648.2 | 1 | 31/128 | 3.69E9 |
| | 129.49 - 130.96 | -.SVANSQDIDLVVDANAVAYNSSDVKDIT/ | 3451.74 | 3 | 4.64 | 0.60 | 1319.9 | 1 | 36/128 | 4.83E9 |
| | 131.55 - 132.93 | -.SVANSQDIDLVVDANAVAYNSSDVKDIT/ | 3451.74 | 3 | 4.24 | 0.36 | 653.7 | 1 | 32/128 | 5.97E9 |
| | 80.78 - 82.08 | -.TGVSNTLENEFK.- | 1339.43 | 2 | 3.25 | 0.58 | 899.2 | 1 | 16/22 | 5.42E9 |
| #24 | DPS_ECOLI (P27430) | | | | | 70.27 | | | 7 (7 0 0 0 0) | 0.51 |
| | 79.40 | -.AIGEAKDDDTADILTAASR.- | 1934.05 | 2 | 5.34 | 0.65 | 2038.9 | 1 | 25/36 | 2.27E9 |
| | 95.01 | -.AVQLGGVALGTTQVINSK.- | 1757.03 | 1 | 2.11 | 0.50 | 160.1 | 2 | 12/34 | 8.33E8 |
| | 163.06 | -.DLDKFLWFIESNIE.- | 1769.98 | 2 | 3.14 | 0.31 | 806.6 | 1 | 14/26 | 5.06E8 |
| | 92.77 - 93.86 | -.GANFIAVHEM*LDGFR.- | 1693.91 | 2 | 3.04 | 0.42 | 675.3 | 1 | 16/28 | 2.73E9 |
| | 71.06 | -.KAIGEAKDDDTADILTAASR.- | 2062.23 | 2 | 4.77 | 0.65 | 888.4 | 1 | 20/38 | 1.02E9 |
| | 81.85 - 82.79 | -.SYPLDIHNVQDHLK.- | 1679.86 | 2 | 3.00 | 0.11 | 469.8 | 1 | 13/26 | 2.42E9 |

| | | | | | | | | | | | |
|---|---|---|---|---|---|---|---|---|---|---|---|
| | 69.78 | -.TALIDHLDTM*AER.- | 1502.68 | 2 | 2.55 | 0.39 | 504.5 | 1 | 14/24 | | 1.33E9 |
| #25 | RS12_ECOLI (P02367) | | | | | 70.24 | | | 7 (7 0 0 0 0) | | 0.71 |
| | 117.81 - 118.72 | -.LTNGFEVTSYIGGEGHNLQEHSVILIR.- | 2985.30 | 3 | 4.86 | 0.57 | 902.7 | 1 | 31/104 | | 7.46E9 |
| | 119.77 - 120.59 | -.LTNGFEVTSYIGGEGHNLQEHSVILIR.- | 2985.30 | 3 | 3.47 | 0.31 | 479.1 | 1 | 25/104 | | 3.36E9 |
| | 15.77 - 17.24 | -.SNVPALEACPQK.- | 1314.46 | 2 | 2.92 | 0.51 | 777.7 | 1 | 16/22 | | 5.26E8 |
| | 17.84 - 19.32 | -.SNVPALEACPQK.- | 1314.46 | 2 | 3.16 | 0.46 | 468.6 | 1 | 14/22 | | 2.79E8 |
| | 18.11 - 19.49 | -.SNVPALEACPQK.- | 1314.46 | 2 | 2.55 | 0.46 | 480.1 | 1 | 15/22 | | 2.43E8 |
| | 21.32 - 23.16 | -.SNVPALEACPQK.- | 1314.46 | 2 | 2.56 | 0.42 | 431.8 | 1 | 14/22 | | 1.23E8 |
| | 11.53 | -.SNVPALEACPQKR.- | 1470.65 | 2 | 2.65 | 0.47 | 708.8 | 1 | 15/24 | | 3.44E9 |
| #26 | RL11_ECOLI (P02409) | | | | | 70.22 | | | 7 (7 0 0 0 0) | | 1.43 |
| | 12.13 | -.AQLQEIAQTK.- | 1130.28 | 1 | 2.55 | 0.44 | 442.1 | 2 | 11/18 | | 3.76E9 |
| | 12.15 - 13.20 | -.AQLQEIAQTK.- | 1130.28 | 2 | 3.55 | 0.41 | 994.7 | 1 | 15/18 | | 2.90E9 |
| | 12.24 | -.AQLQEIAQTK.- | 1130.28 | 1 | 2.52 | 0.44 | 391.1 | 1 | 11/18 | | 2.37E9 |
| | 123.53 - 124.72 | -.GLPIPVVITVYADR.- | 1513.81 | 2 | 4.46 | 0.72 | 1033.1 | 1 | 22/26 | | 1.03E10 |
| | 123.74 - 124.32 | -.GLPIPVVITVYADR.- | 1513.81 | 1 | 2.44 | 0.47 | 336.8 | 1 | 13/26 | | 3.47E9 |
| | 124.19 | -.GLPIPVVITVYADR.- | 1513.81 | 1 | 3.11 | 0.50 | 461.4 | 1 | 14/26 | | 2.70E9 |
| | 66.54 - 67.66 | -.TPPAAVLLK.- | 910.14 | 1 | 1.98 | 0.27 | 658.8 | 1 | 13/16 | | 5.28E9 |
| #27 | EFTU_ECOLI (P02990 | | | | | 70.20 | | | 7 (7 0 0 0 0) | | 0.60 |
| | 49.92 - 50.66 | -.AFDQIDNAPEEK.- | 1377.44 | 2 | 2.86 | 0.35 | 892.8 | 1 | 15/22 | | 7.89E8 |
| | 48.34 - 48.85 | -.AFDQIDNAPEEK.- | 1377.44 | 2 | 3.02 | 0.41 | 557.2 | 1 | 12/22 | | 1.21E9 |
| | 74.13 | -.AGENVGVLLR.- | 1028.19 | 1 | 1.90 | 0.19 | 248.4 | 2 | 11/18 | | 1.56E9 |
| | 108.32 | -.ELLSQYDFPGDDTPIVR.- | 1966.14 | 2 | 2.88 | 0.54 | 358.2 | 1 | 14/32 | | 2.02E9 |
| | 77.00 | -.GITINTSHVEYDTPTR.- | 1804.94 | 2 | 4.00 | 0.60 | 1459.2 | 1 | 21/30 | | 1.53E9 |
| | 70.98 - 72.18 | -.GITINTSHVEYDTPTR.- | 1804.94 | 2 | 3.03 | 0.56 | 886.9 | 1 | 17/30 | | 3.45E9 |
| | 115.60 | -.TTLTAAITTVLAK.- | 1304.56 | 2 | 3.91 | 0.55 | 1969.0 | 1 | 21/24 | | 2.38E9 |
| #28 | YEEX_ECOLI (P76367 | | | | | 60.33 | | | 6 (6 0 0 0 0) | | 0.99 |
| | 83.31 - 84.18 | -.GDYEDRVDDYIIK.- | 1601.70 | 2 | 2.89 | 0.17 | 640.8 | 1 | 15/24 | | 2.75E9 |
| | 143.32 | -.VLLLDNLSDYIKPGM*SVEAIQGIIASM*K. | 3052.60 | 3 | 5.83 | 0.66 | 860.5 | 1 | 29/108 | | 5.38E9 |
| | 141.43 - 142.72 | -.VLLLDNLSDYIKPGM*SVEAIQGIIASM*K. | 3052.60 | 3 | 5.67 | 0.50 | 1264.7 | 1 | 34/108 | | 7.11E9 |
| | 158.28 - 159.44 | -.VLLLDNLSDYIKPGM*SVEAIQGIIASMK.- | 3036.60 | 3 | 6.62 | 0.28 | 2884.4 | 1 | 46/108 | | 2.40E9 |
| | 148.44 - 149.11 | -.VLLLDNLSDYIKPGMSVEAIQGIIASM*K.- | 3036.60 | 3 | 5.58 | 0.58 | 1235.8 | 1 | 31/108 | | 2.36E9 |
| | 162.39 - 163.59 | -.VLLLDNLSDYIKPGMSVEAIQGIIASMK.- | 3020.60 | 3 | 5.74 | 0.64 | 2085.9 | 1 | 38/108 | | 1.45E9 |
| #29 | FABZ_ECOLI (P21774 | | | | | 60.31 | | | 6 (6 0 0 0 0) | | 0.93 |
| | 83.98 - 84.60 | -.FKRPVVPGDQM*IM*EVTFEK.- | 2284.68 | 2 | 3.16 | 0.55 | 302.5 | 1 | 17/36 | | 2.26E9 |
| | 84.04 | -.FKRPVVPGDQM*IM*EVTFEK.- | 2284.68 | 3 | 6.26 | 0.65 | 1649.5 | 1 | 35/72 | | 2.03E9 |
| | 112.50 | -.FPFLLVDR.- | 1007.21 | 2 | 2.75 | 0.18 | 973.4 | 1 | 12/14 | | 1.83E9 |
| | 115.87 - 117.11 | -.LEPGELYYFAGIDEAR.- | 1844.01 | 2 | 3.98 | 0.62 | 865.8 | 1 | 22/30 | | 7.99E9 |
| | 117.54 - 118.13 | -.LEPGELYYFAGIDEAR.- | 1844.01 | 2 | 2.73 | 0.49 | 345.5 | 1 | 15/30 | | 4.68E9 |
| | 82.77 - 83.33 | -.RPVVPGDQM*IM*EVTFEK.- | 2009.34 | 2 | 3.19 | 0.54 | 274.1 | 1 | 15/32 | | 1.19E9 |
| #30 | SECB_ECOLI (P15040 | | | | | 60.31 | | | 6 (6 0 0 0 0) | | 2.19 |
| | 90.74 | -.DISFEAPNAPHVFQK.- | 1700.88 | 1 | 3.21 | 0.45 | 515.0 | 1 | 15/28 | | 1.55E9 |
| | 90.57 - 91.16 | -.DISFEAPNAPHVFQK.- | 1700.88 | 2 | 4.13 | 0.56 | 563.1 | 1 | 18/28 | | 5.28E9 |
| | 151.83 - 152.91 | -.LDLDTASSQLADDVYEVVLR.- | 2223.42 | 2 | 6.16 | 0.63 | 1928.1 | 1 | 25/38 | | 1.70E10 |
| | 152.27 - 152.78 | -.LDLDTASSQLADDVYEVVLR.- | 2223.42 | 3 | 4.11 | 0.52 | 1632.5 | 1 | 31/76 | | 3.96E9 |
| | 153.44 - 154.89 | -.LDLDTASSQLADDVYEVVLR.- | 2223.42 | 2 | 3.74 | 0.59 | 311.8 | 2 | 11/38 | | 1.76E10 |
| | 117.85 | -.LDLDTASSQLADDVYEVVLR.- | 2223.42 | 2 | 4.28 | 0.63 | 748.3 | 1 | 18/38 | | 1.95E9 |
| #31 | Q8XAT8 (Q8XAT8) Os | | | | | 60.26 | | | 6 (6 0 0 0 0) | | 1.84 |
| | 79.27 - 79.89 | -.GKGTVSTESGVLNQQPYGFNTR.- | 2341.52 | 3 | 3.87 | 0.49 | 914.2 | 1 | 33/84 | | 3.60E9 |
| | 87.00 - 87.61 | -.GTVSTESGVLNQQPYGFNTR.- | 2156.30 | 2 | 3.77 | 0.60 | 520.2 | 1 | 16/38 | | 7.51E9 |
| | 85.15 - 86.41 | -.GTVSTESGVLNQQPYGFNTR.- | 2156.30 | 2 | 5.27 | 0.63 | 621.3 | 1 | 17/38 | | 7.59E9 |
| | 109.51 - 110.55 | -.SEVAVPGIDASTFDGIIQK.- | 1948.16 | 2 | 3.19 | 0.55 | 389.6 | 1 | 18/36 | | 2.29E9 |
| | 113.40 - 114.44 | -.SEVAVPGIDASTFDGIIQK.- | 1948.16 | 2 | 3.11 | 0.36 | 488.7 | 1 | 20/36 | | 1.58E10 |
| | 105.87 | -.SEVAVPGIDASTFDGIIQK.- | 1948.16 | 2 | 3.61 | 0.60 | 394.2 | 1 | 18/36 | | 2.96E9 |
| #32 | RL16_ECOLI (P02414) | | | | | 60.26 | | | 6 (6 0 0 0 0) | | 2.21 |
| | 110.57 - 111.30 | -.GKGNVEYWVALIQPGK.- | 1760.03 | 2 | 5.24 | 0.62 | 1425.0 | 1 | 20/30 | | 3.36E9 |
| | 111.47 | -.GKGNVEYWVALIQPGK.- | 1760.03 | 3 | 3.48 | 0.44 | 1314.1 | 1 | 27/60 | | 1.32E9 |
| | 107.03 | -.GLAQGTDVSFGSFGLK.- | 1584.76 | 1 | 3.10 | 0.41 | 584.4 | 1 | 16/30 | | 3.88E9 |
| | 106.87 - 108.07 | -.GLAQGTDVSFGSFGLK.- | 1584.76 | 2 | 5.03 | 0.56 | 1944.8 | 1 | 24/30 | | 1.90E10 |
| | 119.81 - 120.76 | -.GNVEYWVALIQPGK.- | 1574.81 | 2 | 2.62 | 0.39 | 863.1 | 1 | 14/26 | | 6.63E9 |
| | 84.33 - 85.04 | -.VLYEM*DGVPEELAR.- | 1637.84 | 2 | 4.39 | 0.56 | 686.7 | 1 | 19/26 | | 1.36E10 |
| #33 | OMPC_ECO57 (Q8XE | | | | | 60.25 | | | 6 (6 0 0 0 0) | | 0.90 |
| | 130.37 - 131.57 | -.AQNFEAVAQYQFDFGLRPSLAYLQSK.- | 2993.32 | 3 | 5.03 | 0.52 | 1224.6 | 1 | 34/100 | | 3.46E9 |
| | 78.29 - 79.53 | -.FQDVGSFDYGR.- | 1291.35 | 2 | 2.91 | 0.47 | 1098.4 | 1 | 16/20 | | 3.53E9 |
| | 93.59 - 94.17 | -.INLLDDNQFTR.- | 1349.48 | 2 | 3.86 | 0.42 | 1367.1 | 1 | 18/20 | | 3.17E9 |
| | 149.28 - 150.81 | -.NTDFFGLVDGLNFAVQYQGK.- | 2234.45 | 2 | 2.63 | 0.42 | 393.1 | 1 | 14/38 | | 2.79E9 |
| | 66.92 - 67.57 | -.NYDDEDILK.- | 1125.17 | 1 | 2.16 | 0.12 | 341.2 | 2 | 11/16 | | 3.48E9 |
| | 88.85 | -.YVDVGATYYFNK.- | 1440.58 | 2 | 2.87 | 0.28 | 1357.7 | 1 | 18/22 | | 3.06E9 |
| #34 | YFBU_ECO57 (Q8XCV | | | | | 60.18 | | | 6 (6 0 0 0 0) | | 0.67 |
| | 75.91 | -.ELDREFGELKEETCR.- | 1912.04 | 3 | 3.19 | 0.42 | 733.2 | 1 | 24/56 | | 1.78E9 |
| | 76.00 | -.ELDREFGELKEETCR.- | 1912.04 | 2 | 3.08 | 0.44 | 452.1 | 1 | 16/28 | | 3.41E9 |
| | 50.37 - 50.96 | -.FM*VNVEGR.- | 968.11 | 2 | 2.67 | 0.36 | 748.5 | 1 | 13/14 | | 7.73E8 |
| | 106.33 - 106.39 | -.QYHLSANEINQIINA.- | 1728.89 | 2 | 2.66 | 0.42 | 362.4 | 1 | 12/28 | | 3.15E9 |
| | 93.41 - 93.46 | -.RVTFLGFDAATEAR.- | 1554.73 | 2 | 3.59 | 0.57 | 930.1 | 1 | 19/26 | | 3.36E9 |
| | 114.40 - 115.78 | -.TIIDIM*EM*YHALHVSWSNLQDQQSIDEF | 3406.75 | 3 | 3.66 | 0.43 | 321.1 | 218 | 19/108 | | 2.00E9 |
| #35 | DCEA_ECO57 (P5822 | | | | | 50.28 | | | 5 (5 0 0 0 0) | | 0.57 |
| | 114.59 - 115.16 | -.LGPYEFICTGRPDEGIPAVCFK.- | 2527.84 | 3 | 3.51 | 0.33 | 957.0 | 3 | 26/84 | 1 | 3.38E9 |
| | 49.69 - 50.31 | -.LQGIAQQNSFK.- | 1234.39 | 2 | 2.93 | 0.55 | 742.6 | 1 | 15/20 | 1 | 5.17E8 |
| | 93.39 - 93.94 | -.NWIDKEEYPQSAAIDLR.- | 2049.23 | 2 | 3.12 | 0.37 | 472.7 | 1 | 18/32 | 1 | 2.99E9 |
| | 117.18 - 117.74 | -.PAGQVIAQYYEFLR.- | 1655.88 | 2 | 3.54 | 0.43 | 999.2 | 1 | 16/26 | 1 | 2.99E9 |
| | 120.71 - 121.37 | -.VQNASYQVAAYLADEIAK.- | 1955.16 | 2 | 5.51 | 0.65 | 1422.4 | 1 | 21/34 | 1 | 2.51E9 |
| #36 | PAL_ECOLI (P07176) I | | | | | 50.26 | | | 5 (5 0 0 0 0) | | 0.60 |
| | 114.17 | -.LQM*QQLQQNNIVYFDLDKYDIR.- | 2803.14 | 3 | 4.26 | 0.48 | 575.4 | 4 | 23/84 | | 2.40E9 |
| | 119.66 | -.LQMQQLQQNNIVYFDLDKYDIR.- | 2787.14 | 3 | 5.16 | 0.57 | 1327.3 | 1 | 31/84 | | 1.80E9 |
| | 102.47 | -.SDFAQM*LDAHANFLR.- | 1752.93 | 2 | 3.18 | 0.48 | 641.4 | 1 | 15/28 | | 3.89E9 |

| | | | | | | | | | | |
|---|---|---|---|---|---|---|---|---|---|---|
| | 102.45 | -.SDFAQM*LDAHANFLR.- | 1752.93 | 3 | 3.55 | 0.45 | 1024.4 | 1 | 28/56 | 1.25E9 |
| | 100.95 | -.SDFAQM*LDAHANFLR.- | 1752.93 | 2 | 2.93 | 0.42 | 606.2 | 1 | 14/28 | 3.55E9 |
| #37 | YFID_ECOLI (P33633) | | | | | 50.25 | | | 5 (5 0 0 0 0) | 0.58 |
| | 126.59 - 128.01 | -.AANDDLLNSFWLLDSEKGEAR.- | 2365.54 | 3 | 4.25 | 0.47 | 826.7 | 1 | 27/80 | 4.40E9 |
| | 65.97 - 66.52 | -.AGYAEDEVVAVSK.- | 1338.45 | 2 | 4.90 | 0.58 | 2241.5 | 1 | 21/24 | 2.65E9 |
| | 12.39 | -.EVPVEVKPEVR.- | 1281.48 | 2 | 2.60 | 0.52 | 477.4 | 1 | 13/20 | 1.74E9 |
| | 67.61 | -.VEGGQHLNVNVLR.- | 1435.61 | 1 | 2.34 | 0.40 | 231.4 | 3 | 10/24 | 9.00E8 |
| | 67.38 - 68.43 | -.VEGGQHLNVNVLR.- | 1435.61 | 2 | 3.69 | 0.52 | 1259.0 | 1 | 17/24 | 2.80E9 |
| #38 | CEST_ECO57 (P5823 | | | | | 50.24 | | | 5 (5 0 0 0 0) | 0.70 |
| | 128.30 - 129.64 | -.FPLDDATPEKLENEIEVVVK.- | 2286.56 | 3 | 4.84 | 0.60 | 1543.6 | 1 | 31/76 | 7.15E9 |
| | 93.44 | -.IGIGSISFNENR.- | 1307.44 | 1 | 2.05 | 0.25 | 267.8 | 1 | 15/22 | 2.37E9 |
| | 92.95 - 93.57 | -.IGIGSISFNENR.- | 1307.44 | 2 | 2.98 | 0.64 | 918.7 | 1 | 18/22 | 3.42E9 |
| | 51.80 | -.SELLLEK.- | 831.98 | 2 | 2.82 | 0.35 | 541.0 | 2 | 11/12 | 6.74E8 |
| | 99.19 | -.SM*ENLYLVLHNQGITLENEHM*K.- | 2646.98 | 3 | 4.03 | 0.54 | 1156.8 | 1 | 28/84 | 1.58E9 |
| #39 | HFQ_ECOLI (P25521) | | | | | 50.23 | | | 5 (5 0 0 0 0) | 0.59 |
| | 98.82 - 99.03 | -.AKGQSLQDPFLNALR.- | 1658.88 | 2 | 3.97 | 0.57 | 1418.1 | 1 | 19/28 | 3.89E9 |
| | 98.90 | -.AKGQSLQDPFLNALR.- | 1658.88 | 3 | 4.53 | 0.29 | 1936.9 | 1 | 30/56 | 9.32E8 |
| | 97.77 | -.ERVPVSIYLVNGIK.- | 1587.89 | 2 | 3.41 | 0.35 | 904.8 | 1 | 17/26 | 2.52E9 |
| | 109.18 - 109.74 | -.GQSLQDPFLNALR.- | 1459.63 | 2 | 2.54 | 0.46 | 467.7 | 1 | 13/24 | 3.79E9 |
| | 102.88 | -.VPVSIYLVNGIK.- | 1302.59 | 2 | 3.58 | 0.59 | 1344.9 | 1 | 18/22 | 1.55E9 |
| #40 | RL21_ECOLI (P02422) | | | | | 50.17 | | | 5 (5 0 0 0 0) | 0.62 |
| | 102.97 | -.IGVPFVDGGVIK.- | 1201.44 | 2 | 2.53 | 0.36 | 608.8 | 1 | 17/22 | 3.59E9 |
| | 104.40 - 104.94 | -.IGVPFVDGGVIK.- | 1201.44 | 2 | 3.32 | 0.37 | 612.8 | 1 | 18/22 | 7.90E9 |
| | 43.12 - 44.25 | -.M*YAVFQSGGK.- | 1104.26 | 2 | 2.61 | 0.50 | 1455.7 | 1 | 16/18 | 5.47E8 |
| | 41.24 - 42.48 | -.M*YAVFQSGGK.- | 1104.26 | 2 | 2.98 | 0.63 | 1246.9 | 1 | 15/18 | 7.01E8 |
| | 39.45 - 40.60 | -.M*YAVFQSGGK.- | 1104.26 | 2 | 2.80 | 0.53 | 1179.4 | 1 | 15/18 | 7.51E8 |
| #41 | TPX_ECOLI (P37901) | | | | | 40.26 | | | 4 (4 0 0 0 0) | 0.58 |
| | 119.98 - 120.27 | -.FCGAEGLNNVITLSTFR.- | 1900.12 | 2 | 5.18 | 0.69 | 2253.4 | 1 | 23/32 | 2.10E9 |
| | 123.72 | -.FCGAEGLNNVITLSTFR.- | 1900.12 | 2 | 3.17 | 0.46 | 861.7 | 1 | 17/32 | 3.83E9 |
| | 120.31 - 121.75 | -.NAEFLQAYGVAIADGPLK.- | 1878.12 | 2 | 4.45 | 0.60 | 994.6 | 1 | 17/34 | 2.93E9 |
| | 116.60 - 117.24 | -.VLNIFPSIDTGVCAASVR.- | 1920.19 | 2 | 3.83 | 0.52 | 386.1 | 1 | 17/34 | 3.69E9 |
| #42 | YFIA_ECOLI (P11285) | | | | | 40.25 | | | 4 (4 0 0 0 0) | 0.39 |
| | 100.93 | -.EPQGFVADATINTPNGVLVASGK.- | 2286.53 | 2 | 3.69 | 0.43 | 753.2 | 1 | 18/44 | 1.89E9 |
| | 103.90 | -.EPQGFVADATINTPNGVLVASGK.- | 2286.53 | 2 | 4.72 | 0.59 | 898.7 | 1 | 20/44 | 2.36E9 |
| | 99.36 - 99.97 | -.EPQGFVADATINTPNGVLVASGK.- | 2286.53 | 2 | 4.94 | 0.53 | 1278.5 | 1 | 22/44 | 2.33E9 |
| | 119.34 - 119.94 | -.HEDM*YTAINELINKLER.- | 2106.35 | 3 | 5.06 | 0.49 | 1487.4 | 1 | 31/64 | 1.78E9 |
| #43 | BCP_ECOLI (P23480) | | | | | 40.24 | | | 4 (4 0 0 0 0) | 0.71 |
| | 77.21 | -.AGVDVLGISTDKPEK.- | 1529.72 | 1 | 2.42 | 0.50 | 559.1 | 1 | 15/28 | 9.02E8 |
| | 76.88 - 77.44 | -.AGVDVLGISTDKPEK.- | 1529.72 | 2 | 3.56 | 0.61 | 868.6 | 1 | 18/28 | 2.95E9 |
| | 135.12 - 135.77 | -.ELLNFTLLSDEDHQVCEQFGVWGEK.- | 2995.24 | 3 | 4.09 | 0.52 | 1363.2 | 1 | 33/96 | 2.50E9 |
| | 105.65 - 106.21 | -.FSLPDQDGEQVNLTDFQGQR.- | 2295.41 | 2 | 4.85 | 0.60 | 782.4 | 1 | 18/38 | 9.06E9 |
| #44 | ENO_ECOLI (P08324) | | | | | 40.24 | | | 4 (4 0 0 0 0) | 0.71 |
| | 119.10 - 119.25 | -.FNQIGSLTETLAAIK.- | 1606.85 | 2 | 4.81 | 0.43 | 1205.7 | 1 | 22/28 | 2.76E9 |
| | 84.84 - 85.75 | -.GM*PLYEHIAELNGTPGK.- | 1844.08 | 2 | 3.60 | 0.60 | 1158.8 | 1 | 19/32 | 3.11E9 |
| | 107.05 - 107.76 | -.IQLVGDDLFVTNTK.- | 1563.78 | 2 | 3.80 | 0.47 | 1027.1 | 1 | 18/26 | 5.00E9 |
| | 108.65 - 109.26 | -.SGETEDATIADLAVGTAAGQIK.- | 2119.27 | 2 | 4.58 | 0.50 | 1183.5 | 1 | 21/42 | 4.39E9 |
| #45 | Q8X6N7 (Q8X6N7) Put | | | | | 40.23 | | | 4 (4 0 0 0 0) | 0.33 |
| | 86.18 - 87.19 | -.SNDITALRPYLSDK.- | 1593.76 | 2 | 3.43 | 0.55 | 926.4 | 1 | 15/26 | 4.24E9 |
| | 65.52 - 66.08 | -.TTLPDSAHVASASTIPNR.- | 1839.00 | 2 | 4.62 | 0.54 | 1555.2 | 1 | 21/34 | 1.73E9 |
| | 49.41 - 50.02 | -.YLGGSVHATAGTLR.- | 1403.57 | 2 | 3.08 | 0.48 | 1015.5 | 1 | 19/26 | 6.36E8 |
| | 47.30 - 48.76 | -.YLGGSVHATAGTLR.- | 1403.57 | 2 | 2.98 | 0.49 | 1514.4 | 1 | 21/26 | 5.67E8 |
| #46 | YIFE_ECOLI (P27827) | | | | | 40.21 | | | 4 (4 0 0 0 0) | 0.31 |
| | 84.96 | -.HGYAFNELDLGK.- | 1364.49 | 2 | 4.07 | 0.58 | 2127.9 | 1 | 20/22 | 2.51E9 |
| | 86.73 | -.REPVTEEEKLFVAVCR.- | 1963.22 | 2 | 3.27 | 0.53 | 611.5 | 1 | 14/30 | 1.51E9 |
| | 64.86 - 65.89 | -.RFHTLSGGKPQVEGAEDYTDSDD.- | 2525.58 | 3 | 4.19 | 0.62 | 1209.2 | 1 | 32/88 | 1.42E9 |
| | 64.70 - 65.29 | -.RFHTLSGGKPQVEGAEDYTDSDD.- | 2525.58 | 2 | 2.97 | 0.38 | 172.4 | 12 | 10/44 | 1.19E9 |
| #47 | USPG_ECO57 (Q8XBT | | | | | 40.21 | | | 4 (4 0 0 0 0) | 0.31 |
| | 153.70 | -.FGSVRDEVNELAEELGADVVVIGSR.- | 2661.91 | 2 | 4.17 | 0.46 | 581.2 | 1 | 20/48 | 6.83E8 |
| | 153.46 - 154.08 | -.FGSVRDEVNELAEELGADVVVIGSR.- | 2661.91 | 3 | 4.04 | 0.48 | 866.7 | 1 | 27/96 | 1.66E9 |
| | 86.20 - 87.15 | -.NPSISTHLLGSNASSVIR.- | 1854.06 | 2 | 3.88 | 0.54 | 897.7 | 1 | 17/34 | 1.80E9 |
| | 126.55 - 127.12 | -.TIIM*PVDVFEM*ELSDK.- | 1900.20 | 2 | 3.58 | 0.58 | 279.9 | 1 | 14/30 | 2.47E9 |
| #48 | RL20_ECOLI (P02421) | | | | | 40.17 | | | 4 (4 0 0 0 0) | 0.66 |
| | 101.20 - 101.74 | -.ILADIAVFDK.- | 1105.31 | 2 | 3.48 | 0.53 | 760.3 | 1 | 16/18 | 3.42E9 |
| | 82.14 - 82.73 | -.VAFTALVEK.- | 978.17 | 2 | 2.81 | 0.20 | 543.9 | 1 | 13/16 | 2.20E9 |
| | 82.10 | -.VAFTALVEK.- | 978.17 | 1 | 2.01 | 0.16 | 567.0 | 2 | 11/16 | 2.94E9 |
| | 82.02 - 83.71 | -.VAFTALVEK.- | 978.17 | 1 | 1.91 | 0.03 | 489.3 | 9 | 10/16 | 5.64E9 |
| #49 | SLYB_ECOLI (P55741) | | | | | 30.30 | | | 3 (3 0 0 0 0) | 0.50 |
| | 89.30 - 89.87 | -.SLATAAGAVAGGVAGQGVQSAM*NK.- | 2133.37 | 2 | 6.07 | 0.64 | 1703.6 | 1 | 25/46 | 4.59E9 |
| | 98.55 - 99.29 | -.SLATAAGAVAGGVAGQGVQSAMNK.- | 2117.37 | 2 | 5.98 | 0.62 | 1759.9 | 1 | 26/46 | 5.06E9 |
| | 68.74 | -.VVLASNGSQVTVSPR.- | 1514.71 | 1 | 2.38 | 0.58 | 192.5 | 1 | 14/28 | 1.26E9 |
| #50 | RL7_ECOLI (P02392) | | | | | 30.29 | | | 3 (3 0 0 0 0) | 0.18 |
| | 49.71 | -.ALEEAGAEVEVK.- | 1245.36 | 2 | 2.61 | 0.44 | 1209.8 | 1 | 18/22 | 3.42E8 |
| | 73.65 | -.DLVESAPAALK.- | 1114.27 | 1 | 2.00 | 0.49 | 522.7 | 1 | 12/20 | 1.71E9 |
| | 170.02 | -.DQIIEAVAAM*SVM*DVVELISAM*EEK.- | 2771.18 | 3 | 5.80 | 0.49 | 982.0 | 1 | 32/96 | 1.94E9 |
| #51 | RS14_ECOLI (P02370) | | | | | 30.26 | | | 3 (3 0 0 0 0) | 0.41 |
| | 100.34 | -.AIISDVNASDEDRWNAVLK.- | 2117.31 | 2 | 3.39 | 0.44 | 529.3 | 1 | 14/36 | 1.57E9 |
| | 102.05 | -.AIISDVNASDEDRWNAVLK.- | 2117.31 | 2 | 4.73 | 0.63 | 1339.8 | 1 | 22/36 | 2.28E9 |
| | 96.64 - 97.95 | -.AIISDVNASDEDRWNAVLK.- | 2117.31 | 2 | 5.25 | 0.59 | 1465.7 | 1 | 20/36 | 4.99E9 |
| #52 | Q8XE62 (Q8XE62) Yaj | | | | | 30.24 | | | 3 (3 0 0 0 0) | 0.26 |
| | 161.05 - 162.74 | -.GYM*VGPNGPVNLQIIVSQLYADVSQGN | 3106.50 | 3 | 4.50 | 0.53 | 2027.9 | 1 | 37/112 | 1.93E9 |
| | 162.88 - 163.50 | -.GYM*VGPNGPVNLQIIVSQLYADVSQGN | 3106.50 | 3 | 3.91 | 0.51 | 1528.2 | 1 | 35/112 | 1.08E9 |
| | 137.73 - 139.07 | -.NIADAVNSVLTDTIADM*SQDTSIHEFIK.- | 3066.34 | 3 | 4.79 | 0.65 | 656.5 | 1 | 30/108 | 2.62E9 |
| #53 | FTNA_ECOLI (P23887) | | | | | 30.24 | | | 3 (3 0 0 0 0) | 0.59 |
| | 105.79 - 106.44 | -.LFDYLTDTGNLPR.- | 1525.69 | 2 | 3.53 | 0.49 | 1394.6 | 1 | 18/24 | 5.28E9 |

| | | | | | | | | | | |
|---|---|---|---|---|---|---|---|---|---|---|
| | 104.68 - 105.81 | -.LFDYLTDTGNLPR.- | 1525.69 | 2 | 3.67 | 0.37 | 1889.1 | 1 | 20/24 | 4.60E9 |
| | 128.44 - 129.66 | -.SGEGLYFIDKELSTLDTQN.- | 2131.28 | 2 | 4.77 | 0.64 | 895.8 | 1 | 19/36 | 2.92E9 |
| #54 | USPA_ECOLI (P28242 | | | | | 30.23 | | | 3 (3 0 0 0 0) | 0.58 |
| | 88.07 - 88.68 | -.HILIAVDLSPESK.- | 1422.65 | 1 | 2.33 | 0.33 | 950.3 | 1 | 16/24 | 2.79E9 |
| | 88.18 | -.HILIAVDLSPESK.- | 1422.65 | 1 | 3.86 | 0.55 | 1076.6 | 1 | 18/24 | 1.71E9 |
| | 87.96 - 89.10 | -.HILIAVDLSPESK.- | 1422.65 | 2 | 4.57 | 0.61 | 1631.4 | 1 | 21/24 | 7.93E9 |
| #55 | Q52355 (Q52355) Puta | | | | | 30.23 | | | 3 (3 0 0 0 0) | 0.49 |
| | 129.72 - 129.75 | -.SSEELKVFDTNEVIEFFNK.- | 2276.48 | 2 | 4.44 | 0.49 | 780.2 | 1 | 19/36 | 1.99E9 |
| | 126.76 - 127.35 | -.VFDTNEVIEFFNK.- | 1602.77 | 2 | 4.04 | 0.62 | 1639.9 | 1 | 18/24 | 5.92E9 |
| | 99.60 - 100.89 | -.YILALKDQPEAAQLALR.- | 1914.24 | 2 | 4.56 | 0.61 | 942.4 | 1 | 19/32 | 2.60E9 |
| #56 | NDK_ECOLI (P24233) | | | | | 30.23 | | | 3 (3 0 0 0 0) | 0.25 |
| | 112.77 - 113.34 | -.DLLGATNPANALAGTLR.- | 1668.88 | 2 | 4.51 | 0.60 | 1071.2 | 1 | 21/32 | 4.35E9 |
| | 32.79 - 33.98 | -.M*LHLTVEQAR.- | 1214.42 | 2 | 2.77 | 0.43 | 1099.1 | 1 | 15/18 | 4.85E8 |
| | 30.78 - 31.47 | -.M*LHLTVEQAR.- | 1214.42 | 2 | 2.55 | 0.29 | 1494.2 | 1 | 17/18 | 5.76E8 |
| #57 | NIFU_ECOLI (P77310) | | | | | 30.21 | | | 3 (3 0 0 0 0) | 0.22 |
| | 73.09 | -.NVGSFDNNDENVGSGM*VGAPACGDVI | 2774.93 | 2 | 2.58 | 0.35 | 135.6 | 42 | 10/52 | 1.05E9 |
| | 112.83 | -.TYGCGSAIASSSLVTEWVK.- | 2017.22 | 2 | 4.12 | 0.53 | 807.6 | 1 | 18/36 | 1.74E9 |
| | 62.91 - 64.31 | -.VNDEGIIEDAR.- | 1231.30 | 2 | 3.65 | 0.44 | 1288.9 | 1 | 17/20 | 2.07E9 |
| #58 | G3P1_ECOLI (P06977 | | | | | 30.20 | | | 3 (3 0 0 0 0) | 0.41 |
| | 149.68 - 150.85 | -.SDIEIVAINDLLDADYM*AYM*LK.- | 2549.90 | 3 | 4.08 | 0.52 | 1399.8 | 1 | 31/84 | 1.16E9 |
| | 98.69 | -.VPTPNVSVVDLTVR.- | 1496.73 | 1 | 2.96 | 0.56 | 373.5 | 1 | 14/26 | 1.22E9 |
| | 98.25 - 99.50 | -.VPTPNVSVVDLTVR.- | 1496.73 | 2 | 3.45 | 0.59 | 1438.2 | 1 | 19/26 | 6.41E9 |
| #59 | RS9_ECOLI (P02363) | | | | | 30.15 | | | 3 (3 0 0 0 0) | 0.42 |
| | 60.55 - 61.24 | -.ALM*EYDESLR.- | 1243.37 | 2 | 2.94 | 0.18 | 796.3 | 2 | 14/18 | 4.92E9 |
| | 76.90 | -.ALMEYDESLR.- | 1227.37 | 2 | 2.51 | 0.35 | 1103.3 | 1 | 15/18 | 1.49E9 |
| | 81.83 - 82.45 | -.SLEQYFGR.- | 1000.09 | 2 | 2.54 | 0.45 | 949.1 | 1 | 12/14 | 2.65E9 |
| #60 | YAEH_ECO57 (P6276 | | | | | 30.14 | | | 3 (3 0 0 0 0) | 0.22 |
| | 71.33 | -.ISEIEADLEK.- | 1147.26 | 1 | 1.97 | 0.23 | 904.9 | 1 | 14/18 | 1.36E9 |
| | 76.42 | -.SLGITNPEEIDR.- | 1344.45 | 1 | 2.18 | 0.20 | 380.1 | 1 | 13/22 | 1.44E9 |
| | 76.40 | -.SLGITNPEEIDR.- | 1344.45 | 2 | 2.79 | 0.40 | 1008.2 | 1 | 17/22 | 1.85E9 |
| #61 | ATPE_ECO57 (P5864 | | | | | 20.28 | | | 2 (2 0 0 0 0) | 0.23 |
| | 109.72 - 110.28 | -.IQVTGSEGELGIYPGHAPLLTAIKPGM*IR | 3036.54 | 3 | 5.16 | 0.60 | 1501.3 | 1 | 35/112 | 4.17E9 |
| | 62.68 | -.KAEEHISSSHGDVDYAQASAELAK.- | 2544.67 | 2 | 5.69 | 0.66 | 1728.4 | 1 | 25/46 | 7.15E8 |
| #62 | PUR6_ECOLI (P09028 | | | | | 20.27 | | | 2 (2 0 0 0 0) | 0.29 |
| | 138.76 - 139.16 | -.LFSFAESAEENGYQVIIAGAGGAAHLPGI | 3308.71 | 3 | 3.25 | 0.39 | 696.8 | 1 | 29/128 | 1.87E9 |
| | 150.41 - 151.79 | -.TLVPVLGVPVQSAALSGVDSLYSIVQM*F | 3014.53 | 3 | 5.45 | 0.67 | 754.1 | 1 | 31/112 | 4.31E9 |
| #63 | ADHE_ECOLI (P17547 | | | | | 20.26 | | | 2 (2 0 0 0 0) | 0.20 |
| | 129.22 | -.EAGVQEADFLANVDKLSEDAFDDQCTG | 3384.52 | 3 | 5.18 | 0.54 | 570.2 | 1 | 28/120 | 1.87E9 |
| | 112.20 | -.ILINTPASQGGIGDLYNFK.- | 2022.29 | 2 | 3.90 | 0.56 | 393.2 | 1 | 16/36 | 2.50E9 |
| #64 | Q8XCU0 (Q8XCU0) Pe | | | | | 20.25 | | | 2 (2 0 0 0 0) | 0.22 |
| | 94.58 - 95.63 | -.VINGFM*IQGGGFEPGM*K.- | 1815.11 | 2 | 5.07 | 0.49 | 951.9 | 1 | 19/32 | 2.33E9 |
| | 92.48 - 92.93 | -.VINGFM*IQGGGFEPGM*K.- | 1815.11 | 2 | 3.69 | 0.43 | 579.9 | 1 | 15/32 | 2.33E9 |
| #65 | HDEA_ECOLI (P26604 | | | | | 20.25 | | | 2 (2 0 0 0 0) | 0.34 |
| | 117.57 | -.DKPEDAVLDVQGIATVTPAIVQACTQDK. | 2984.30 | 2 | 5.03 | 0.64 | 686.0 | 1 | 20/54 | 1.43E9 |
| | 116.10 - 117.39 | -.DKPEDAVLDVQGIATVTPAIVQACTQDK. | 2984.30 | 3 | 4.24 | 0.37 | 393.0 | 14 | 25/108 | 5.94E9 |
| #66 | STPA_ECOLI (P30017 | | | | | 20.23 | | | 2 (2 0 0 0 0) | 0.19 |
| | 100.07 - 101.03 | -.ADGINPEELLGNSSAAAPR.- | 1883.01 | 2 | 4.60 | 0.68 | 794.0 | 1 | 18/36 | 2.47E9 |
| | 61.51 | -.TPKPIAQALAEGK.- | 1324.55 | 2 | 2.72 | 0.54 | 662.9 | 1 | 18/24 | 1.74E9 |
| #67 | ATPB_ECOLI (P00824 | | | | | 20.23 | | | 2 (2 0 0 0 0) | 0.15 |
| | 106.81 | -.AAPSYEELSNSQELLETGIK.- | 2180.36 | 2 | 4.32 | 0.45 | 869.7 | 1 | 18/38 | 1.78E9 |
| | 136.26 | -.FLSQPFFVAEVFTGSPGK.- | 1959.23 | 2 | 4.52 | 0.69 | 478.9 | 1 | 18/34 | 1.48E9 |
| #68 | RL6_ECOLI (P02390) | | | | | 20.22 | | | 2 (2 0 0 0 0) | 0.18 |
| | 107.74 | -.ALLNSM*VIGVTEGFTK.- | 1696.99 | 2 | 4.38 | 0.66 | 700.8 | 1 | 17/30 | 2.61E9 |
| | 83.69 | -.GADKQVIGQVAADLR.- | 1541.74 | 2 | 2.67 | 0.28 | 561.9 | 1 | 15/28 | 1.36E9 |
| #69 | OMPA_ECOLI (P0293 | | | | | 20.22 | | | 2 (2 0 0 0 0) | 0.47 |
| | 77.91 | -.FGQGEAAPVVAPAPAPAPEVQTK.- | 2233.51 | 2 | 4.34 | 0.53 | 536.6 | 1 | 19/44 | 1.25E9 |
| | 111.05 - 112.04 | -.LGYPITDDLDIYTR.- | 1655.83 | 2 | 2.89 | 0.55 | 826.0 | 1 | 16/26 | 8.92E9 |
| #70 | Q8X828 (Q8X828) Hyp | | | | | 20.21 | | | 2 (2 0 0 0 0) | 0.49 |
| | 107.83 - 108.44 | -.VLPQGFGSGLVAM*PDGVLQTR.- | 2159.50 | 2 | 4.21 | 0.62 | 698.5 | 1 | 20/40 | 4.31E9 |
| | 105.90 - 107.26 | -.VLPQGFGSGLVAM*PDGVLQTR.- | 2159.50 | 2 | 3.97 | 0.57 | 528.6 | 1 | 17/40 | 6.32E9 |
| #71 | RBFA_ECOLI (P09170 | | | | | 20.20 | | | 2 (2 0 0 0 0) | 0.16 |
| | 132.21 - 132.38 | -.IVPELTFFYDNSLVEGM*R.- | 2147.44 | 2 | 3.99 | 0.54 | 717.7 | 1 | 18/34 | 2.12E9 |
| | 65.18 - 66.24 | -.M*SNLVTSVVK.- | 1094.31 | 2 | 2.85 | 0.41 | 1201.0 | 1 | 14/18 | 1.27E9 |
| #72 | YIBN_ECOLI (P37688) | | | | | 20.19 | | | 2 (2 0 0 0 0) | 0.28 |
| | 107.54 - 108.97 | -.EGVAGWAGENLPLVR.- | 1568.76 | 2 | 2.90 | 0.42 | 735.1 | 2 | 15/28 | 4.09E9 |
| | 77.95 - 78.49 | -.LINKEDAVVVDLR.- | 1484.72 | 2 | 3.85 | 0.52 | 1689.5 | 1 | 20/24 | 2.03E9 |
| #73 | Q8X5N5 (Q8X5N5) Hy | | | | | 20.19 | | | 2 (2 0 0 0 0) | 0.19 |
| | 99.89 | -.KFM*GM*DTASILFFNK.- | 1783.11 | 2 | 3.15 | 0.45 | 350.2 | 1 | 15/28 | 2.18E9 |
| | 119.23 | -.QLLSEQVSAFHTLAASLK.- | 1944.22 | 2 | 3.80 | 0.54 | 357.0 | 1 | 14/34 | 2.01E9 |
| #74 | MINE_ECOLI (P18198) | | | | | 20.19 | | | 2 (2 0 0 0 0) | 0.17 |
| | 134.06 | -.DGDISILELNVTLPEAEELK.- | 2199.44 | 2 | 3.54 | 0.56 | 761.7 | 1 | 18/38 | 1.28E9 |
| | 102.55 | -.YVQIDPEM*VTVQLEQK.- | 1937.20 | 2 | 3.79 | 0.54 | 912.1 | 1 | 20/30 | 2.49E9 |
| #75 | YNCE_ECO57 (Q8X9X | | | | | 20.18 | | | 2 (2 0 0 0 0) | 0.18 |
| | 94.09 | -.ELVADDATNTVYISGIGK.- | 1867.05 | 2 | 3.62 | 0.71 | 897.2 | 1 | 17/34 | 1.62E9 |
| | 118.11 - 118.68 | -.VAAPESLAVLFNPAR.- | 1555.80 | 2 | 2.54 | 0.54 | 926.7 | 1 | 15/28 | 2.22E9 |
| #76 | RL1_ECOLI (P02384) | | | | | 20.17 | | | 2 (2 0 0 0 0) | 0.16 |
| | 78.39 | -.KGEM*NFDVVIASPDAM*R.- | 1913.17 | 2 | 3.34 | 0.58 | 321.6 | 1 | 15/32 | 1.80E9 |
| | 80.38 - 80.43 | -.VVGQLGQVLGPR.- | 1223.45 | 2 | 3.12 | 0.51 | 1235.1 | 1 | 17/22 | 1.74E9 |
| #77 | YAJC_ECOLI (P19677) | | | | | 20.17 | | | 2 (2 0 0 0 0) | 0.14 |
| | 87.50 | -.LM*DSIAKGDEVLTNGGLVGR.- | 2062.33 | 2 | 3.31 | 0.53 | 528.3 | 1 | 16/38 | 1.39E9 |
| | 84.92 | -.LM*DSIAKGDEVLTNGGLVGR.- | 2062.33 | 2 | 2.97 | 0.42 | 783.1 | 1 | 19/38 | 1.74E9 |
| #78 | Q8XDF3 (Q8XDF3) As | | | | | 20.15 | | | 2 (2 0 0 0 0) | 0.11 |
| | 49.81 - 50.92 | -.KAEQYLLENETTK.- | 1567.72 | 2 | 2.88 | 0.57 | 353.5 | 1 | 13/24 | 4.66E8 |
| | 157.84 - 159.17 | -.M*FENITAAPADPILGLADLFR.- | 2292.64 | 2 | 3.02 | 0.17 | 202.4 | 4 | 13/40 | 1.94E9 |

| | | | | | | | | | | |
|---|---|---|---|---|---|---|---|---|---|---|
| #79 | PYRI_ECOLI (P00478) | | | | | 20.14 | | | 2 (2 0 0 0 0) | 0.19 |
| | 93.70 | -.GTVIDHIPAQIGFK.- | 1496.74 | 2 | 2.81 | 0.58 | 573.0 | 1 | 15/26 | 1.83E9 |
| | 121.44 | -.IENTFLSEDQVDQLALYAPQATVNR.- | 2837.09 | 2 | 2.74 | 0.43 | 332.7 | 1 | 14/48 | 2.19E9 |
| #80 | NUSB_ECOLI (P04381 | | | | | 20.14 | | | 2 (2 0 0 0 0) | 0.13 |
| | 78.43 | -.LLEELGQVEK.- | 1158.33 | 1 | 2.04 | 0.16 | 465.0 | 13 | 10/18 | 1.56E9 |
| | 78.41 | -.LLEELGQVEK.- | 1158.33 | 2 | 2.73 | 0.42 | 1005.8 | 1 | 15/18 | 1.32E9 |
| #81 | EAE_ECO57 (P43261) | | | | | 10.27 | | | 1 (1 0 0 0 0) | 0.08 |
| | 134.69 | -.LPFEYSALPLLGSAPLVAAGGVAGHTNK | 2752.16 | 3 | 5.43 | 0.61 | 1546.2 | 1 | 35/108 | 1.80E9 |
| #82 | RPOA_ECOLI (P00574 | | | | | 10.27 | | | 1 (1 0 0 0 0) | 0.02 |
| | 164.95 | -.EGVQEDILEILLNLK.- | 1726.99 | 2 | 5.42 | 0.57 | 1568.0 | 1 | 19/28 | 5.39E8 |
| #83 | AHPC_ECOLI (P26427 | | | | | 10.25 | | | 1 (1 0 0 0 0) | 0.09 |
| | 169.37 | -.ATFVVDPQGIIQAIEVTAEGIGR.- | 2385.70 | 3 | 5.07 | 0.49 | 2104.8 | 1 | 37/88 | 1.93E9 |
| #84 | Q8XCT1 (Q8XCT1) Hy | | | | | 10.25 | | | 1 (1 0 0 0 0) | 0.09 |
| | 152.11 - 152.67 | -.FPEGTSEEQIDKTVDDFINEVIEPNK.- | 2995.20 | 3 | 5.04 | 0.48 | 1186.5 | 1 | 30/100 | 1.84E9 |
| #85 | Q8X8U1 (Q8X8U1) Sb | | | | | 10.24 | | | 1 (1 0 0 0 0) | 0.07 |
| | 165.18 - 166.44 | -.VVGDDFAKPWYQFFNSLLQDSAYEM*LF | 3326.72 | 3 | 4.76 | 0.51 | 735.0 | 1 | 29/108 | 1.50E9 |
| #86 | ELAB_ECOLI (P52084) | | | | | 10.23 | | | 1 (1 0 0 0 0) | 0.11 |
| | 168.41 | -.IDDDLTLLSETLEEVLR.- | 1975.18 | 2 | 4.60 | 0.37 | 613.9 | 1 | 16/32 | 2.38E9 |
| #87 | Q8X6P6 (Q8X6P6) Hyp | | | | | 10.23 | | | 1 (1 0 0 0 0) | 0.08 |
| | 102.43 | -.AVIVIM*GDDPKEDLAVLAK.- | 2014.37 | 2 | 4.60 | 0.65 | 530.4 | 1 | 19/36 | 1.83E9 |
| #88 | MDH_ECO57 (P61891) | | | | | 10.23 | | | 1 (1 0 0 0 0) | 0.07 |
| | 132.98 | -.TQLPSGSELSLYDIAPVTPGVAVDLSHIP | 3377.83 | 3 | 4.60 | 0.57 | 1352.1 | 1 | 38/128 | 1.41E9 |
| #89 | RP5M_ECOLI (P31221 | | | | | 10.22 | | | 1 (1 0 0 0 0) | 0.11 |
| | 99.85 - 100.47 | -.M*QLNITGNNVEITEALR.- | 1933.18 | 2 | 4.43 | 0.50 | 655.6 | 1 | 17/32 | 2.36E9 |
| #90 | Q8X4A1 (Q8X4A1) Hyp | | | | | 10.22 | | | 1 (1 0 0 0 0) | 0.09 |
| | 161.05 - 162.74 | -.NNILDILNLYNVELAAIR.- | 2072.39 | 2 | 4.34 | 0.61 | 323.7 | 8 | 11/34 | 1.93E9 |
| #91 | SLYA_ECOLI (P55740) | | | | | 10.21 | | | 1 (1 0 0 0 0) | 0.03 |
| | 157.86 | -.AEILHGISAEELEQLITLIAK.- | 2292.66 | 3 | 4.21 | 0.46 | 1270.5 | 1 | 28/80 | 5.91E8 |
| #92 | FUR_ECOLI (P06975) | | | | | 10.21 | | | 1 (1 0 0 0 0) | 0.16 |
| | 98.11 - 99.00 | -.LIDM*GEEIGLATVYR.- | 1696.95 | 2 | 4.20 | 0.67 | 1267.7 | 1 | 18/28 | 3.45E9 |
| #93 | Q7DB82 (Q7DB82) Z5 | | | | | 10.21 | | | 1 (1 0 0 0 0) | 0.10 |
| | 117.93 | -.M*VDTFNDEVFNYYLEQK.- | 2172.36 | 2 | 4.17 | 0.64 | 896.0 | 1 | 16/32 | 2.13E9 |
| #94 | RL3_ECO57 (P60440) | | | | | 10.21 | | | 1 (1 0 0 0 0) | 0.14 |
| | 123.76 - 124.28 | -.IFTEDGVSIPVTVIEVEANR.- | 2189.45 | 2 | 4.15 | 0.54 | 439.5 | 1 | 19/38 | 3.05E9 |
| #95 | DLDH_ECOLI (P00391 | | | | | 10.21 | | | 1 (1 0 0 0 0) | 0.08 |
| | 127.61 - 127.69 | -.VIPSIAYTEPEVAWVGLTEK.- | 2203.52 | 2 | 4.10 | 0.49 | 587.8 | 1 | 19/38 | 1.69E9 |
| #96 | PGK_ECO57 (Q8XD03 | | | | | 10.19 | | | 1 (1 0 0 0 0) | 0.14 |
| | 146.01 - 147.28 | -.ADEQILDIGDASAQELAEILK.- | 2243.45 | 2 | 3.81 | 0.50 | 700.4 | 1 | 18/40 | 2.99E9 |
| #97 | SERA_ECOLI (P08328 | | | | | 10.18 | | | 1 (1 0 0 0 0) | 0.18 |
| | 112.65 - 113.19 | -.YSDNGSTLSAVNFPEVSLPLHGGR.- | 2518.72 | 3 | 3.67 | 0.50 | 965.4 | 1 | 29/92 | 3.97E9 |
| #98 | Q8X937 (Q8X937) Hyp | | | | | 10.18 | | | 1 (1 0 0 0 0) | 0.09 |
| | 82.06 - 82.66 | -.TGIPDADKVNIQIADGK.- | 1755.95 | 2 | 3.65 | 0.53 | 670.2 | 1 | 18/32 | 1.99E9 |
| #99 | YFHP_ECOLI (P77484) | | | | | 10.18 | | | 1 (1 0 0 0 0) | 0.06 |
| | 144.59 | -.QGISLSYLEQLFSR.- | 1641.85 | 2 | 3.64 | 0.63 | 652.0 | 1 | 16/26 | 1.19E9 |
| #100 | YFEU_ECO57 (Q8XBJ | | | | | 10.18 | | | 1 (1 0 0 0 0) | 0.44 |
| | 97.00 - 97.31 | -.M*QLEKMITEGSNAASAEIDR.- | 2211.46 | 3 | 3.61 | 0.00 | 1258.1 | 4 | 28/76 | 9.52E9 |
| #101 | Q8X6G9 (Q8X6G9) Z0 | | | | | 10.18 | | | 1 (1 0 0 0 0) | 0.09 |
| | 90.83 - 91.18 | -.ISSLDTATQILSK.- | 1377.57 | 2 | 3.58 | 0.36 | 1279.0 | 1 | 17/24 | 1.95E9 |
| #102 | Q8XCN7 (Q8XCN7) Hy | | | | | 10.18 | | | 1 (1 0 0 0 0) | 0.13 |
| | 84.81 - 85.06 | -.AEAEQTLAALTEK.- | 1375.51 | 2 | 3.57 | 0.56 | 1721.6 | 1 | 18/24 | 2.79E9 |
| #103 | Q8X5Y3 (Q8X5Y3) Put | | | | | 10.18 | | | 1 (1 0 0 0 0) | 0.23 |
| | 96.64 - 97.95 | -.DVVVVSGNDEDEETQLAM*M*GLHGAQI | 3175.45 | 3 | 3.56 | 0.23 | 502.9 | 7 | 25/112 | 4.99E9 |
| #104 | Q8XBU9 (Q8XBU9) Hy | | | | | 10.18 | | | 1 (1 0 0 0 0) | 0.12 |
| | 92.37 | -.LGDDVLEAEM*PVDIR.- | 1688.88 | 2 | 3.55 | 0.36 | 1511.7 | 1 | 18/28 | 2.49E9 |
| #105 | YQJD_ECO57 (P64583 | | | | | 10.18 | | | 1 (1 0 0 0 0) | 0.25 |
| | 121.09 - 122.36 | -.SLSDTLEEVLSSSGEK.- | 1681.78 | 2 | 3.53 | 0.62 | 976.4 | 1 | 17/30 | 5.34E9 |
| #106 | USPD_ECO57 (P6709 | | | | | 10.18 | | | 1 (1 0 0 0 0) | 0.08 |
| | 134.24 - 135.29 | -.LSADLLIVPFIDK.- | 1444.74 | 2 | 3.53 | 0.56 | 1010.3 | 1 | 18/24 | 1.69E9 |
| #107 | RvrsDB\|00000622 | | | | | 10.18 | | | 1 (1 0 0 0 0) | 0.12 |
| | 134.89 - 136.17 | -.YGVVM*PCKALMCELAATGSALLAADSA | 3390.93 | 3 | 3.53 | 0.15 | 514.7 | 27 | 24/124 | 2.62E9 |
| #108 | Q8XD27 (Q8XD27) Hy | | | | | 10.18 | | | 1 (1 0 0 0 0) | 0.08 |
| | 131.30 | -.LPTIIDAPAQEFATIYVSGGK.- | 2192.50 | 2 | 3.52 | 0.48 | 576.1 | 1 | 19/40 | 1.66E9 |
| #109 | CH60_ECOLI (P06139) | | | | | 10.17 | | | 1 (1 0 0 0 0) | 0.03 |
| | 163.02 | -.AGKPLLIIAEDVEGEALATLVVNTM*R.- | 2741.20 | 3 | 3.48 | 0.46 | 1060.6 | 1 | 30/100 | 5.66E8 |
| #110 | Q8X622 (Q8X622) Rib | | | | | 10.17 | | | 1 (1 0 0 0 0) | 0.14 |
| | 103.84 | -.VNIEIDPQTQAVVDTVER.- | 2027.22 | 2 | 3.42 | 0.57 | 831.0 | 1 | 19/34 | 3.06E9 |
| #111 | KPY1_ECOLI (P14178) | | | | | 10.17 | | | 1 (1 0 0 0 0) | 0.08 |
| | 108.42 | -.GAVETAEKLDAPLIVVATQGGK.- | 2168.48 | 2 | 3.40 | 0.51 | 354.7 | 3 | 13/42 | 1.79E9 |
| #112 | RvrsDB\|00004723 | | | | | 10.17 | | | 1 (1 0 0 0 0) | 0.11 |
| | 102.05 | -.QLQGGSLQPPRKDLVSDDLDVAHLMES | 3177.54 | 3 | 3.38 | 0.21 | 148.9 | 467 | 17/112 | 2.28E9 |
| #113 | PPIA_ECOLI (P20752) | | | | | 10.17 | | | 1 (1 0 0 0 0) | 0.13 |
| | 131.67 - 132.91 | -.APVSVQNFVDYVNSGFYNNTTFHR.- | 2777.99 | 3 | 3.34 | 0.42 | 534.9 | 3 | 25/92 | 2.88E9 |
| #114 | RS20_ECOLI (P02378) | | | | | 10.16 | | | 1 (1 0 0 0 0) | 0.04 |
| | 55.25 - 56.53 | -.AFNEM*QPIVDR.- | 1336.50 | 2 | 3.22 | 0.48 | 652.3 | 1 | 13/20 | 7.80E8 |
| #115 | Q8XDR0 (Q8XDR0) Hy | | | | | 10.16 | | | 1 (1 0 0 0 0) | 0.05 |
| | 83.59 | -.IVDEQPGAECQLIGTATGK.- | 1988.18 | 2 | 3.16 | 0.51 | 336.7 | 1 | 16/36 | 9.96E8 |
| #116 | EFG_ECOLI (P02996) | | | | | 10.16 | | | 1 (1 0 0 0 0) | 0.15 |
| | 93.99 - 95.38 | -.YDEAPSNVAQAVIEAR.- | 1733.86 | 2 | 3.16 | 0.51 | 413.1 | 1 | 15/30 | 3.32E9 |
| #117 | Q8X8W0 (Q8X8W0) R | | | | | 10.16 | | | 1 (1 0 0 0 0) | 0.07 |
| | 89.41 | -.IYTNAEELVGKPFR.- | 1637.86 | 2 | 3.15 | 0.58 | 793.5 | 1 | 15/26 | 1.60E9 |
| #118 | RvrsDB\|00004000 | | | | | 10.15 | | | 1 (1 0 0 0 0) | 0.11 |
| | 127.27 | -.FVTVDDCSEFIEKM*RHSIYVIGRGQSK.- | 3219.61 | 3 | 3.08 | 0.25 | 603.0 | 10 | 26/104 | 2.39E9 |
| #119 | Q8X966 (Q8X966) Pyr | | | | | 10.15 | | | 1 (1 0 0 0 0) | 0.08 |
| | 131.25 | -.YINIGVAVDTPNGLVVPVFK.- | 2116.49 | 2 | 3.06 | 0.55 | 721.6 | 1 | 16/38 | 1.70E9 |

| | | | | | | | | | | |
|---|---|---|---|---|---|---|---|---|---|---|
| #120 | RvrsDB\|00003615 | | | | | 10.15 | | | 1 (1 0 0 0 0) | 0.05 |
| | 63.67 | -.LDRQADELNTKYLK.- | 1707.91 | 3 | 3.05 | 0.24 | 807.7 | 5 | 23/52 | 1.18E9 |
| #121 | BLC_ECOLI (P39281) ( | | | | | 10.15 | | | 1 (1 0 0 0 0) | 0.15 |
| | 136.63 - 137.95 | -.VSFFGPFYGGYNVIALDR.- | 2023.28 | 2 | 3.01 | 0.59 | 628.6 | 1 | 16/34 | 3.21E9 |
| #122 | YRAP_ECO57 (P6459 | | | | | 10.15 | | | 1 (1 0 0 0 0) | 0.09 |
| | 82.62 - 83.37 | -.VLLVGQSPNAELSAR.- | 1554.77 | 2 | 2.98 | 0.62 | 837.0 | 1 | 18/28 | 2.03E9 |
| #123 | Q8X9N3 (Q8X9N3) Z1 | | | | | 10.15 | | | 1 (1 0 0 0 0) | 0.11 |
| | 136.38 - 136.94 | -.VYDFLLPAQDTFPGDIIR.- | 2081.36 | 2 | 2.95 | 0.49 | 279.4 | 1 | 14/34 | 2.44E9 |
| #124 | Q9LAN9 (Q9LAN9) Put | | | | | 10.15 | | | 1 (1 0 0 0 0) | 0.04 |
| | 65.07 | -.TGEGDGDDEQVKIDLTK.- | 1820.89 | 2 | 2.91 | 0.54 | 318.9 | 1 | 14/32 | 7.95E8 |
| #125 | DUT_ECO57 (P64007) | | | | | 10.15 | | | 1 (1 0 0 0 0) | 0.12 |
| | 110.34 - 110.87 | -.EFPLPTYATSGSAGLDLR.- | 1896.09 | 2 | 2.90 | 0.39 | 492.5 | 1 | 16/34 | 2.56E9 |
| #126 | RvrsDB\|00004199 | | | | | 10.14 | | | 1 (1 0 0 0 0) | 0.26 |
| | 97.97 - 98.53 | -.TSQEDTAEQRSIVESFLQR.- | 2225.36 | 2 | 2.90 | 0.00 | 443.5 | 3 | 15/36 | 5.64E9 |
| #127 | RvrsDB\|00004098 | | | | | 10.14 | | | 1 (1 0 0 0 0) | 0.11 |
| | 128.42 - 128.94 | -.DQLM*AANEVLNM*AFTTK.- | 1930.19 | 2 | 2.81 | 0.24 | 536.1 | 3 | 13/32 | 2.27E9 |
| #128 | ATPA_ECOLI (P00822 | | | | | 10.14 | | | 1 (1 0 0 0 0) | 0.06 |
| | 125.33 | -.ELAAFSQFASDLDDATR.- | 1857.96 | 2 | 2.81 | 0.54 | 623.1 | 1 | 15/32 | 1.26E9 |
| #129 | WBDQ_ECO57 (O853 | | | | | 10.14 | | | 1 (1 0 0 0 0) | 0.07 |
| | 90.28 | -.M*FLHSQDFATIVR.- | 1581.82 | 2 | 2.75 | 0.50 | 919.8 | 1 | 16/24 | 1.56E9 |
| #130 | IVY_ECOLI (P45502) Ir | | | | | 10.14 | | | 1 (1 0 0 0 0) | 0.07 |
| | 84.25 | -.SNQM*TGLFSTIDEK.- | 1587.73 | 2 | 2.74 | 0.41 | 833.7 | 1 | 16/26 | 1.56E9 |
| #131 | Q8XBL1 (Q8XBL1) PT | | | | | 10.14 | | | 1 (1 0 0 0 0) | 0.09 |
| | 117.89 - 118.45 | -.VGDTVIEFDLPQLEEK.- | 1833.03 | 2 | 2.74 | 0.51 | 661.5 | 1 | 14/30 | 1.88E9 |
| #132 | YHCB_ECOLI (P39436 | | | | | 10.14 | | | 1 (1 0 0 0 0) | 0.17 |
| | 116.25 - 117.48 | -.SSSSLLPELSAEANPFR.- | 1805.97 | 2 | 2.70 | 0.34 | 337.7 | 1 | 15/32 | 3.69E9 |
| #133 | Q8XE01 (Q8XE01) Hyp | | | | | 10.13 | | | 1 (1 0 0 0 0) | 0.11 |
| | 108.01 | -.LFGEYYYSPDSLSSGIK.- | 1927.10 | 2 | 2.70 | 0.48 | 613.9 | 1 | 15/32 | 2.34E9 |
| #134 | ECOT_ECO57 (Q8XE4 | | | | | 10.13 | | | 1 (1 0 0 0 0) | 0.10 |
| | 97.44 | -.LPIVVYTPDNVDVK.- | 1572.83 | 2 | 2.70 | 0.47 | 645.5 | 1 | 15/26 | 2.20E9 |
| #135 | ATPF_ECOLI (P00859 | | | | | 10.13 | | | 1 (1 0 0 0 0) | 0.04 |
| | 61.85 | -.AEAQVIIEQANK.- | 1314.47 | 2 | 2.68 | 0.41 | 818.7 | 1 | 14/22 | 9.12E8 |
| #136 | Q8X7N6 (Q8X7N6) Tra | | | | | 10.13 | | | 1 (1 0 0 0 0) | 0.05 |
| | 65.09 - 65.68 | -.LEPADDFRDEPVK.- | 1531.65 | 2 | 2.67 | 0.35 | 896.0 | 1 | 17/24 | 9.75E8 |
| #137 | RS19_ECOLI (P02375 | | | | | 10.13 | | | 1 (1 0 0 0 0) | 0.02 |
| | 14.90 - 16.16 | -.LGEFAPTR.- | 891.01 | 2 | 2.63 | 0.39 | 753.5 | 1 | 11/14 | 4.39E8 |
| #138 | Q8X9Z5 (Q8X9Z5) Ace | | | | | 10.13 | | | 1 (1 0 0 0 0) | 0.10 |
| | 100.99 | -.GYNIESLTVAPTDDPTLSR.- | 2050.21 | 2 | 2.57 | 0.39 | 305.5 | 1 | 14/36 | 2.24E9 |
| #139 | O82913 (O82913) Plas | | | | | 10.13 | | | 1 (1 0 0 0 0) | 0.08 |
| | 90.26 | -.FPAFNNPEVYR.- | 1354.50 | 2 | 2.56 | 0.28 | 751.7 | 1 | 13/20 | 1.71E9 |
| #140 | Q8XEC9 (Q8XEC9) NA | | | | | 10.10 | | | 1 (1 0 0 0 0) | 0.14 |
| | 104.72 | -.EDVDRAVK.- | 932.01 | 1 | 2.09 | 0.14 | 516.9 | 14 | 9/14 | 2.96E9 |
| #141 | YOAC_ECO57 (P6449 | | | | | 10.10 | | | 1 (1 0 0 0 0) | 0.12 |
| | 57.74 - 58.37 | -.NM*PAVIDK.- | 904.07 | 1 | 2.05 | 0.25 | 216.0 | 6 | 9/14 | 2.63E9 |
| #142 | Q8X7P6 (Q8X7P6) Hyp | | | | | 10.10 | | | 1 (1 0 0 0 0) | 0.59 |
| | 75.49 - 76.21 | -.KDVRGVFMAK.- | 1151.41 | 1 | 1.98 | 0.27 | 427.9 | 7 | 10/18 | 1.27E10 |
| #143 | Q8X497 (Q8X497) Hyp | | | | | 10.10 | | | 1 (1 0 0 0 0) | 0.15 |
| | 91.54 | -.MIDLSITTR.- | 1050.26 | 1 | 1.98 | 0.17 | 334.8 | 1 | 12/16 | 3.20E9 |
| #144 | RL18_ECOLI (P02419) | | | | | 10.09 | | | 1 (1 0 0 0 0) | 0.20 |
| | 10.25 - 11.19 | -.VQALADAAR.- | 915.03 | 1 | 1.82 | 0.43 | 311.0 | 2 | 9/16 | 4.24E9 |
