# Supplementary material for: Proteins other than the locus of enterocyte effacement-encoded proteins contribute to Escherichia coli O157:H7 adherence to bovine rectoanal junction stratified squamous epithelial cells
Source: BMC Microbiol. 2012 Jun 12;12:103. doi: 10.1186/1471-2180-12-103 (PMC3420319; doi:10.1186/1471-2180-12-103)
Supplement: Additional file 12 — http://www.biomedcentral.com/imedia/1285024576754199/supp12.pdf. DATA SHEETS: O157-DMEM MS/MS data sheet 8. [file 1471-2180-12-103-S12.pdf]

DMEM-08 SequestReport

	Reference					Score	Accession		Peptides (Hits)		Area
	Time(s)	Sequence	MH+	Charge	XC	Delta Cn	Sp	RSp	Ions	Count	Peak Area
#1	RL7_ECOLI (P02392) [illegible]					360.29			36 (36 0 0 0 0)		8.93
	53.23 - 54.48	-.ALEEAGAEVEVK.-	1245.36	2	4.12	0.42	1579.6	1	19/22		2.33E9
	54.26 - 55.79	-.ALEEAGAEVEVK.-	1245.36	1	2.95	0.43	979.4	1	14/22		2.39E9
	56.86 - 58.10	-.ALEEAGAEVEVK.-	1245.36	2	4.13	0.57	1881.1	1	19/22		3.00E9
	58.40 - 59.60	-.ALEEAGAEVEVK.-	1245.36	2	3.89	0.52	1474.3	1	19/22		2.78E9
	60.20 - 61.40	-.ALEEAGAEVEVK.-	1245.36	2	4.03	0.50	1347.0	1	19/22		1.98E9
	63.45 - 64.77	-.ALEEAGAEVEVK.-	1245.36	2	4.78	0.51	1416.3	1	19/22		6.77E9
	65.29 - 66.74	-.ALEEAGAEVEVK.-	1245.36	2	4.38	0.51	1501.2	1	19/22		5.47E9
	67.33 - 68.71	-.ALEEAGAEVEVK.-	1245.36	2	3.64	0.54	1380.1	1	19/22		1.98E9
	69.56	-.ALEEAGAEVEVK.-	1245.36	2	2.93	0.45	1585.8	1	17/22		1.22E9
	70.99	-.ALEEAGAEVEVK.-	1245.36	2	3.76	0.51	1389.7	1	19/22		1.93E9
	205.71 - 206.31	-.ALEEAGAEVEVK.-	1245.36	2	3.78	0.62	1399.3	1	19/22		4.51E7
	79.77	-.ALEEAGAEVEVK.-	1245.36	2	3.07	0.45	1172.5	1	15/22		1.53E9
	74.80 - 75.35	-.DLVESAPAALK.-	1114.27	1	2.79	0.47	441.0	4	10/20		1.56E10
	76.51	-.DLVESAPAALK.-	1114.27	2	2.73	0.49	1145.8	1	15/20		7.32E9
	74.67 - 76.37	-.DLVESAPAALK.-	1114.27	1	2.43	0.53	423.5	1	11/20		3.58E10
	74.77 - 75.93	-.DLVESAPAALK.-	1114.27	2	3.11	0.45	1124.5	1	16/20		1.48E10
	82.58	-.DLVESAPAALKEGVSK.-	1614.82	1	2.29	0.45	513.2	1	16/30		5.49E8
	170.63	-.DQIIEAVAAM*SVM*DVVELISAM*EEK.-	2771.18	3	5.85	0.52	1653.8	1	36/96		8.26E9
	71.32 - 71.96	-.EAKDLVESAPAALK.-	1442.64	2	2.88	0.36	981.6	1	17/26		2.16E9
	114.41 - 115.69	-.FGVSAAAAVAVAAGPVEAAEEK.-	2016.24	2	4.93	0.66	1210.4	1	26/42		4.05E9
	116.91 - 118.25	-.FGVSAAAAVAVAAGPVEAAEEK.-	2016.24	2	4.13	0.56	978.7	1	22/42		3.51E9
	119.83 - 120.15	-.FGVSAAAAVAVAAGPVEAAEEK.-	2016.24	2	3.11	0.49	713.1	1	17/42		2.03E9
	123.40 - 124.80	-.FGVSAAAAVAVAAGPVEAAEEK.-	2016.24	2	2.69	0.44	530.9	1	16/42		2.98E9
	196.46 - 198.34	-.FGVSAAAAVAVAAGPVEAAEEKTEFDVILK.-	2962.34	3	3.01	0.35	818.0	1	28/116		5.80E8
	158.86 - 160.16	-.FGVSAAAAVAVAAGPVEAAEEKTEFDVILK.-	2962.34	3	4.80	0.58	1073.4	1	34/116		2.15E9
	160.71 - 162.37	-.FGVSAAAAVAVAAGPVEAAEEKTEFDVILK.-	2962.34	3	3.57	0.37	953.4	1	33/116		2.43E9
	162.96 - 164.64	-.FGVSAAAAVAVAAGPVEAAEEKTEFDVILK.-	2962.34	3	4.87	0.54	1094.1	1	34/116		1.75E9
	165.22 - 166.92	-.FGVSAAAAVAVAAGPVEAAEEKTEFDVILK.-	2962.34	3	4.81	0.53	908.1	1	32/116		2.76E9
	167.47	-.FGVSAAAAVAVAAGPVEAAEEKTEFDVILK.-	2962.34	3	4.27	0.60	1091.1	1	36/116		2.98E9
	157.10 - 158.78	-.FGVSAAAAVAVAAGPVEAAEEKTEFDVILK.-	2962.34	3	4.55	0.49	965.6	1	30/116		4.86E9
	150.99 - 152.61	-.FGVSAAAAVAVAAGPVEAAEEKTEFDVILK.-	2962.34	3	3.57	0.54	1146.0	1	31/116		1.35E9
	187.48 - 189.29	-.FGVSAAAAVAVAAGPVEAAEEKTEFDVILK.-	2962.34	3	5.85	0.58	836.7	1	31/116		1.36E9
	189.84 - 191.60	-.FGVSAAAAVAVAAGPVEAAEEKTEFDVILK.-	2962.34	3	4.41	0.41	874.2	1	31/116		1.55E9
	192.16 - 193.97	-.FGVSAAAAVAVAAGPVEAAEEKTEFDVILK.-	2962.34	3	3.11	0.41	620.9	1	31/116		7.52E8
	105.63	-.GATGLGLKEAKDLVESAPAALK.-	2140.47	3	4.87	0.50	1614.9	1	34/84		1.05E9
	91.04	-.TEFDVILK.-	965.13	1	2.04	0.21	593.0	1	10/14		3.10E9
#2	MULI_ECOLI (P02937)					310.26			31 (31 0 0 0 0)		17.13
	70.55 - 71.16	-.IDQLSSDVQTLNAK.-	1532.68	2	2.88	0.54	1169.4	1	18/26		1.48E9
	76.86 - 78.02	-.IDQLSSDVQTLNAK.-	1532.68	1	3.58	0.49	758.8	1	16/26		1.98E10
	76.84 - 81.73	-.IDQLSSDVQTLNAK.-	1532.68	1	2.26	0.44	580.2	1	16/26		4.14E10
	76.02 - 77.96	-.IDQLSSDVQTLNAK.-	1532.68	2	4.99	0.61	1551.3	1	20/26		5.38E10
	75.24 - 76.39	-.IDQLSSDVQTLNAK.-	1532.68	2	4.72	0.54	1603.2	1	21/26		7.01E10
	74.50 - 75.16	-.IDQLSSDVQTLNAK.-	1532.68	1	3.28	0.47	458.7	1	14/26		6.51E9
	73.53 - 74.65	-.IDQLSSDVQTLNAK.-	1532.68	2	5.08	0.58	1941.6	1	21/26		9.38E9
	81.13 - 81.71	-.IDQLSSDVQTLNAK.-	1532.68	2	4.85	0.63	1558.2	1	21/26		8.70E9
	71.98 - 72.66	-.IDQLSSDVQTLNAK.-	1532.68	2	3.95	0.57	1056.1	1	17/26		2.15E9
	81.38	-.IDQLSSDVQTLNAK.-	1532.68	1	2.81	0.39	814.6	1	15/26		1.99E9
	78.47 - 79.62	-.IDQLSSDVQTLNAK.-	1532.68	2	5.25	0.61	1888.6	1	23/26		3.84E10
	83.11 - 83.17	-.IDQLSSDVQTLNAK.-	1532.68	2	2.95	0.33	1146.2	1	19/26		1.15E9
	10.88	-.SDVQAAKDDAAR.-	1247.30	2	3.10	0.43	1330.8	1	17/22		2.86E9
	52.37 - 53.94	-.VDQLSNDVNAM*R.-	1378.50	2	3.22	0.53	1108.2	1	16/22		1.87E9
	71.40 - 71.45	-.VDQLSNDVNAM*R.-	1378.50	2	3.48	0.54	1158.5	1	17/22		1.41E9
	13.59 - 14.32	-.VDQLSNDVNAM*R.-	1378.50	2	2.59	0.41	771.6	1	14/22		8.17E8
	11.35 - 11.95	-.VDQLSNDVNAM*R.-	1378.50	2	3.81	0.59	1001.9	1	17/22		1.78E10
	50.36 - 52.00	-.VDQLSNDVNAM*R.-	1378.50	2	4.51	0.56	1099.1	1	17/22		1.11E9
	48.16 - 49.73	-.VDQLSNDVNAM*R.-	1378.50	2	4.25	0.61	1500.7	1	19/22		6.39E8
	46.21 - 48.06	-.VDQLSNDVNAM*R.-	1378.50	2	4.52	0.59	1268.3	1	18/22		5.17E8
	44.02 - 45.60	-.VDQLSNDVNAM*R.-	1378.50	2	4.09	0.59	1007.0	1	17/22		5.69E8
	38.13	-.VDQLSNDVNAM*R.-	1378.50	2	3.11	0.39	810.1	1	16/22		3.60E8
	36.52 - 37.54	-.VDQLSNDVNAM*R.-	1378.50	2	4.00	0.52	1140.4	1	17/22		2.96E8
	34.02 - 35.47	-.VDQLSNDVNAM*R.-	1378.50	2	3.85	0.48	1221.8	1	18/22		5.55E8
	31.75 - 33.25	-.VDQLSNDVNAM*R.-	1378.50	2	3.63	0.48	1221.3	1	17/22		6.81E8
	57.61	-.VDQLSNDVNAM*RSDVQAAK.-	2078.25	2	3.28	0.59	446.7	1	17/36		3.45E8
	71.24 - 72.05	-.VDQLSNDVNAMR.-	1362.50	2	4.39	0.56	1202.6	1	18/22		7.59E9
	53.27	-.VDQLSNDVNAMR.-	1362.50	2	2.91	0.45	870.8	1	15/22		4.17E8
	51.14 - 52.66	-.VDQLSNDVNAMR.-	1362.50	2	3.03	0.53	1150.0	1	16/22		4.53E8
	71.50	-.VDQLSNDVNAMR.-	1362.50	1	2.11	0.26	279.0	1	13/22		2.13E9
	72.89	-.VDQLSNDVNAMR.-	1362.50	2	3.81	0.51	1248.7	1	18/22		2.55E9
#3	CSPC_ECOLI (P36996					250.27			25 (25 0 0 0 0)		6.23
	106.40	-.DVFVHFSAIQGNGFK.-	1666.86	1	3.49	0.60	833.0	1	19/28		1.03E9
	106.10 - 107.34	-.DVFVHFSAIQGNGFK.-	1666.86	2	4.24	0.57	1605.7	1	21/28		7.30E9
	107.94 - 109.21	-.DVFVHFSAIQGNGFK.-	1666.86	2	3.63	0.54	1821.8	1	21/28		3.07E9
	89.52 - 89.63	-.GFGFITPADGSK.-	1197.32	2	2.69	0.49	742.7	1	15/22		1.87E9
	86.92 - 87.97	-.GFGFITPADGSK.-	1197.32	2	3.40	0.55	1305.3	1	18/22		1.04E10
	86.98 - 88.23	-.GFGFITPADGSK.-	1197.32	1	2.35	0.38	564.7	1	13/22		3.74E9
	82.06	-.GPAAVNVTAI.-	913.05	2	2.60	0.58	948.3	1	15/18		1.25E9
	87.39 - 88.59	-.TLAEGQNVEFEIQDGQK.-	1907.03	2	5.16	0.56	1662.2	1	23/32		3.09E9
	89.29 - 89.56	-.TLAEGQNVEFEIQDGQK.-	1907.03	2	4.82	0.51	968.8	1	18/32		1.97E9
	85.68 - 86.81	-.TLAEGQNVEFEIQDGQK.-	1907.03	2	5.00	0.62	1088.4	1	19/32		1.05E10
	90.71 - 92.06	-.TLAEGQNVEFEIQDGQK.-	1907.03	2	4.11	0.54	1081.9	1	19/32		2.10E9

	84.27 - 84.34	-.TLAEGQNVEFEIQDGQK.-	1907.03	3	5.34	0.51	2794.2	1	34/64	1.60E9
	95.02 - 96.49	-.TLAEGQNVEFEIQDGQK.-	1907.03	2	4.89	0.60	827.0	1	17/32	2.12E9
	97.31 - 98.00	-.TLAEGQNVEFEIQDGQK.-	1907.03	2	3.15	0.41	331.5	9	11/32	2.41E9
	98.98 - 100.02	-.TLAEGQNVEFEIQDGQK.-	1907.03	2	4.84	0.47	728.3	1	17/32	1.57E9
	100.64 - 101.38	-.TLAEGQNVEFEIQDGQK.-	1907.03	2	4.23	0.62	941.2	1	17/32	1.12E9
	102.20 - 103.32	-.TLAEGQNVEFEIQDGQK.-	1907.03	2	3.11	0.47	571.8	1	13/32	1.83E9
	103.92 - 105.20	-.TLAEGQNVEFEIQDGQK.-	1907.03	2	2.81	0.33	664.4	1	16/32	2.16E9
	83.94 - 85.10	-.TLAEGQNVEFEIQDGQK.-	1907.03	2	5.12	0.60	1394.5	1	21/32	2.12E10
	82.18 - 83.36	-.TLAEGQNVEFEIQDGQK.-	1907.03	2	5.10	0.56	1246.6	1	20/32	2.13E10
	107.31 - 108.38	-.TLAEGQNVEFEIQDGQK.-	1907.03	2	2.78	0.40	364.5	3	11/32	1.68E9
	93.23 - 94.40	-.TLAEGQNVEFEIQDGQK.-	1907.03	2	4.90	0.60	1275.8	1	18/32	2.36E9
	114.15 - 115.09	-.TLAEGQNVEFEIQDGQK.-	1907.03	2	3.56	0.55	862.2	1	17/32	1.63E9
	133.74	-.TLAEGQNVEFEIQDGQK.-	1907.03	2	3.27	0.61	864.8	1	14/32	7.24E8
	203.94 - 205.87	-.TLAEGQNVEFEIQDGQK.-	1907.03	2	4.76	0.48	956.6	1	17/32	1.51E8
#4	RL29_ECOLI (P02429)					180.27			18 (18 0 0 0 0)	0.85
	130.92	-.EKSVEELNTELLNLLR.-	1901.15	3	4.22	0.48	1216.9	1	26/60	1.19E9
	131.03	-.EKSVEELNTELLNLLR.-	1901.15	2	4.02	0.53	1282.7	1	19/30	1.68E9
	29.68 - 31.31	-.M*QAASGQLQQSHLLK.-	1656.89	2	3.85	0.42	907.8	1	17/28	4.72E8
	51.51	-.M*QAASGQLQQSHLLK.-	1656.89	2	2.54	0.31	621.5	3	14/28	2.21E8
	20.53 - 22.37	-.M*QAASGQLQQSHLLK.-	1656.89	2	4.56	0.52	2504.3	1	24/28	2.43E8
	13.96 - 15.28	-.M*QAASGQLQQSHLLK.-	1656.89	2	3.84	0.45	1509.3	1	21/28	6.78E8
	27.43 - 28.96	-.M*QAASGQLQQSHLLK.-	1656.89	2	4.29	0.43	1382.6	1	21/28	5.42E8
	25.85 - 27.47	-.M*QAASGQLQQSHLLK.-	1656.89	3	3.04	0.38	813.9	1	26/56	3.31E8
	25.32 - 26.83	-.M*QAASGQLQQSHLLK.-	1656.89	2	4.44	0.52	1465.0	1	21/28	5.46E8
	14.85 - 15.55	-.M*QAASGQLQQSHLLK.-	1656.89	3	3.01	0.40	882.4	1	25/56	4.03E8
	18.26 - 19.89	-.M*QAASGQLQQSHLLK.-	1656.89	2	4.63	0.61	1797.4	1	22/28	2.74E8
	17.02 - 18.18	-.M*QAASGQLQQSHLLK.-	1656.89	3	3.44	0.45	964.8	1	27/56	2.52E8
	16.37 - 17.71	-.M*QAASGQLQQSHLLK.-	1656.89	2	4.88	0.57	1256.1	1	21/28	5.09E8
	23.03 - 24.74	-.M*QAASGQLQQSHLLK.-	1656.89	2	4.35	0.46	2174.8	1	24/28	2.50E8
	63.63	-.MQAASGQLQQSHLLK.-	1640.89	2	3.15	0.38	1239.3	1	19/28	6.14E8
	131.78	-.SVEELNTELLNLLR.-	1643.86	2	3.68	0.47	1143.3	1	20/26	9.18E8
	139.01 - 139.61	-.SVEELNTELLNLLR.-	1643.86	2	5.31	0.44	1790.6	1	20/26	5.10E8
	139.42	-.SVEELNTELLNLLR.-	1643.86	1	1.98	0.33	75.5	24	8/26	5.01E8
#5	YFID_ECOLI (P33633)					170.28			17 (17 0 0 0 0)	3.82
	137.88 - 138.95	-.AANDDLLNSFWLLDSEK.-	1952.11	2	5.51	0.63	1448.0	1	20/32	4.65E9
	139.50 - 140.57	-.AANDDLLNSFWLLDSEK.-	1952.11	2	5.00	0.64	1350.6	1	22/32	4.47E9
	130.03 - 130.54	-.AANDDLLNSFWLLDSEK.-	1952.11	2	4.60	0.59	817.2	1	20/32	1.63E9
	127.41 - 128.81	-.AANDDLLNSFWLLDSEKGEAR.-	2365.54	2	2.56	0.48	548.7	1	15/40	3.67E9
	127.24 - 128.44	-.AANDDLLNSFWLLDSEKGEAR.-	2365.54	3	4.59	0.52	1086.3	1	29/80	9.38E9
	68.11	-.AGYAEDEVVAVSK.-	1338.45	1	3.23	0.51	638.0	1	14/24	4.70E9
	67.90 - 69.02	-.AGYAEDEVVAVSK.-	1338.45	2	5.27	0.54	1700.7	1	19/24	8.69E9
	49.84 - 51.07	-.AGYAEDEVVAVSK.-	1338.45	2	3.89	0.53	1263.9	1	19/24	2.93E8
	69.67 - 70.39	-.AGYAEDEVVAVSK.-	1338.45	2	3.14	0.44	876.5	1	17/24	4.39E9
	46.91 - 48.45	-.AGYAEDEVVAVSK.-	1338.45	2	2.78	0.37	763.9	1	15/24	2.02E8
	12.67 - 13.80	-.EVPVEVKPEVR.-	1281.48	2	2.95	0.52	727.6	1	16/20	2.62E9
	20.09 - 32.75	-.LGDIEYR.-	865.95	1	2.00	0.14	292.0	1	9/12	1.65E9
	64.06 - 65.26	-.M*ITGIQITK.-	1021.26	2	2.70	0.36	1113.7	1	16/16	2.36E9
	63.92 - 66.00	-.M*ITGIQITK.-	1021.26	1	1.83	0.39	152.0	1	12/16	4.23E9
	69.48 - 70.66	-.VEGGQHLNVNVLR.-	1435.61	2	4.25	0.52	1390.8	1	19/24	8.41E9
	69.60 - 70.80	-.VEGGQHLNVNVLR.-	1435.61	1	2.73	0.37	321.5	1	12/24	2.90E9
	69.84	-.VEGGQHLNVNVLR.-	1435.61	1	2.98	0.41	391.4	1	13/24	2.10E9
#6	Q8XE69 (Q8XE69) 50S					160.26			16 (16 0 0 0 0)	2.70
	82.31 - 82.92	-.AANKFPAIIYGGK.-	1350.59	1	2.45	0.40	832.9	1	16/24	3.36E9
	82.12 - 83.25	-.AANKFPAIIYGGK.-	1350.59	2	3.06	0.38	828.9	1	17/24	5.10E9
	83.83 - 85.16	-.AANKFPAIIYGGK.-	1350.59	2	2.63	0.35	701.3	1	16/24	4.64E9
	82.79	-.AANKFPAIIYGGK.-	1350.59	1	2.99	0.49	905.4	1	15/24	2.04E9
	138.80	-.AEFYSEVLTIVVDGK.-	1670.88	2	3.16	0.62	1427.5	1	17/28	3.33E9
	136.53 - 137.72	-.AEFYSEVLTIVVDGK.-	1670.88	2	4.90	0.48	1955.3	1	21/28	3.12E9
	137.06 - 138.25	-.AEFYSEVLTIVVDGK.-	1670.88	2	5.18	0.61	2045.3	1	20/28	3.57E9
	75.51	-.EAPLAIELDHDK.-	1351.49	1	1.91	0.23	337.5	1	15/22	9.49E8
	82.67 - 83.21	-.EAPLAIELDHDKVM*NIQAK.-	2152.46	2	4.19	0.61	625.0	1	18/36	7.63E9
	93.83	-.EAPLAIELDHDKVMNIQAK.-	2136.46	2	3.45	0.56	476.9	1	14/36	8.35E8
	85.92 - 86.46	-.FPAIIYGGK.-	966.16	1	2.08	0.39	745.6	1	11/16	1.72E9
	86.61	-.FPAIIYGGK.-	966.16	1	2.13	0.18	431.9	1	11/16	1.47E9
	66.97	-.LQHIDFVR.-	1028.19	1	1.90	0.08	435.7	1	10/14	1.01E9
	71.84 - 72.36	-.LQHIDFVRA.-	1099.27	1	1.94	0.37	627.6	1	11/16	2.07E9
	73.98 - 74.88	-.M*FTINAEVR.-	1097.27	2	3.11	0.58	1184.4	1	15/16	3.16E9
	75.70 - 76.34	-.M*FTINAEVR.-	1097.27	2	2.59	0.61	1063.7	1	14/16	2.88E9
#7	RS10_ECOLI (P02364)					140.26			14 (14 0 0 0 0)	3.00
	79.13	-.FTVLISPHVNK.-	1255.49	1	2.32	0.14	661.4	2	13/20	1.55E9
	78.97 - 80.09	-.FTVLISPHVNK.-	1255.49	2	3.30	0.45	1677.8	1	18/20	4.75E9
	79.18 - 79.73	-.FTVLISPHVNK.-	1255.49	3	3.18	0.29	1170.1	1	23/40	1.30E9
	78.18	-.FTVLISPHVNKDAR.-	1597.84	2	3.02	0.49	700.3	1	14/26	1.13E9
	74.44	-.FTVLISPHVNKDAR.-	1597.84	2	2.58	0.45	736.1	1	16/26	8.89E8
	88.92 - 89.50	-.LIDQATAEIVETAK.-	1502.69	1	3.01	0.38	669.7	2	14/26	3.39E9
	89.31	-.LIDQATAEIVETAK.-	1502.69	1	2.93	0.37	476.4	2	14/26	1.38E9
	88.90 - 90.08	-.LIDQATAEIVETAK.-	1502.69	2	5.27	0.59	1821.9	1	20/26	1.32E10
	87.41	-.LIDQATAEIVETAK.-	1502.69	2	2.86	0.49	513.7	1	14/26	9.44E8
	86.25 - 86.83	-.LIDQATAEIVETAKR.-	1658.88	2	3.65	0.44	1095.8	1	18/28	2.83E9
	69.79 - 70.68	-.LVDIVEPTEK.-	1143.31	1	2.88	0.28	775.9	1	14/18	4.47E9
	69.63 - 70.76	-.LVDIVEPTEK.-	1143.31	2	2.91	0.32	1046.2	1	16/18	5.70E9
	69.62 - 70.74	-.LVDIVEPTEK.-	1143.31	1	2.76	0.24	732.1	1	13/18	9.56E9
	72.51	-.LVDIVEPTEK.-	1143.31	2	2.63	0.27	784.4	1	12/18	1.03E9
#8	PTHP_ECOLI (P07006					140.25			14 (14 0 0 0 0)	3.70

	73.20 - 74.48	-.GFTSEITVTSNGK.-	1341.45	2	4.42	0.60	1737.9	1	20/24	4.06E9
	73.58 - 80.11	-.GFTSEITVTSNGK.-	1341.45	1	2.35	0.51	706.6	1	17/24	7.54E9
	75.01 - 76.14	-.GFTSEITVTSNGK.-	1341.45	2	4.02	0.65	1627.9	1	20/24	5.81E9
	76.72 - 78.06	-.GFTSEITVTSNGK.-	1341.45	2	3.96	0.67	1935.9	1	20/24	4.89E9
	204.80 - 205.43	-.GFTSEITVTSNGK.-	1341.45	2	3.24	0.67	1585.7	1	19/24	5.44E7
	65.80 - 66.42	-.GFTSEITVTSNGKSASAK.-	1785.93	2	4.18	0.59	1432.8	1	23/34	1.31E9
	203.82 - 205.65	-.LQTLGLTQGTVVTISAEGEDEQK.-	2418.64	3	3.72	0.20	972.9	1	28/88	1.83E8
	118.35 - 119.53	-.LQTLGLTQGTVVTISAEGEDEQKAVEHLVK.-	3195.57	3	4.19	0.46	631.1	1	31/116	6.08E9
	80.92 - 81.60	-.M*FQQEVTITAPNGLHTR.-	1960.20	2	5.09	0.66	1575.1	1	25/32	4.79E9
	82.48 - 83.13	-.M*FQQEVTITAPNGLHTR.-	1960.20	2	3.97	0.54	1168.0	1	20/32	3.86E9
	80.94 - 81.26	-.M*FQQEVTITAPNGLHTR.-	1960.20	3	3.53	0.47	1084.5	1	32/64	3.97E9
	89.96 - 91.11	-.M*FQQEVTITAPNGLHTRPAAQFVK.-	2702.09	2	3.94	0.52	311.5	1	16/46	6.78E9
	89.87 - 91.44	-.M*FQQEVTITAPNGLHTRPAAQFVK.-	2702.09	3	4.59	0.47	1175.2	1	31/92	1.28E10
	92.28 - 92.88	-.MFQQEVTITAPNGLHTRPAAQFVK.-	2686.09	3	3.88	0.52	1359.9	1	31/92	2.24E9
#9	DBHA_ECOLI (P02342					130.26			13 (13 0 0 0 0)	4.52
	133.90	-.AALESTLAAITESLK.-	1518.74	3	4.10	0.37	2859.8	1	33/56	8.36E8
	133.94	-.AALESTLAAITESLK.-	1518.74	1	1.87	0.32	289.5	2	12/28	1.44E9
	133.78 - 134.65	-.AALESTLAAITESLK.-	1518.74	2	5.14	0.62	1218.3	1	18/28	1.07E10
	159.08 - 159.66	-.AALESTLAAITESLKEGDAVQLVGFGTFK.-	2968.35	2	4.99	0.66	1078.8	1	22/56	1.75E9
	161.23 - 162.92	-.AALESTLAAITESLKEGDAVQLVGFGTFK.-	2968.35	3	4.30	0.55	916.9	1	30/112	2.73E9
	108.65	-.EGDAVQLVGFGTFK.-	1468.64	1	2.82	0.52	367.2	1	16/26	1.84E9
	108.53 - 109.91	-.EGDAVQLVGFGTFK.-	1468.64	2	4.07	0.63	1610.5	1	20/26	7.33E9
	83.29 - 84.51	-.IAAANVPAFVSGK.-	1245.45	1	2.27	0.47	653.2	1	14/24	1.30E10
	83.23 - 84.38	-.IAAANVPAFVSGK.-	1245.45	2	3.62	0.47	1653.4	1	21/24	1.84E10
	87.87	-.M*NKTQLIDVIAEK.-	1519.79	2	2.85	0.53	924.0	1	17/24	1.14E9
	90.17 - 90.75	-.TQLIDVIAEK.-	1130.32	1	2.21	0.23	1008.9	2	13/18	9.02E9
	90.21 - 91.36	-.TQLIDVIAEK.-	1130.32	2	2.96	0.45	1040.4	1	15/18	6.28E9
	90.40 - 91.06	-.TQLIDVIAEK.-	1130.32	1	2.46	0.32	694.6	3	11/18	4.01E9
#10	HDEA_ECOLI (P26604					90.32			9 (9 0 0 0 0)	6.23
	110.97 - 112.37	-.DKPEDAVLDVQGIATVTPAIVQACTQDK.-	2984.30	3	4.10	0.46	727.6	1	29/108	2.98E9
	112.73	-.DKPEDAVLDVQGIATVTPAIVQACTQDK.-	2984.30	2	4.30	0.59	660.4	1	19/54	5.05E8
	112.96 - 113.74	-.DKPEDAVLDVQGIATVTPAIVQACTQDK.-	2984.30	3	4.17	0.45	1020.4	1	36/108	2.50E9
	115.39 - 116.78	-.DKPEDAVLDVQGIATVTPAIVQACTQDK.-	2984.30	3	5.49	0.57	862.9	1	33/108	1.64E10
	117.38 - 118.57	-.DKPEDAVLDVQGIATVTPAIVQACTQDK.-	2984.30	3	6.42	0.57	1339.0	1	38/108	3.56E10
	117.70 - 118.89	-.DKPEDAVLDVQGIATVTPAIVQACTQDK.-	2984.30	2	4.98	0.63	988.9	1	23/54	9.54E9
	119.04 - 120.28	-.DKPEDAVLDVQGIATVTPAIVQACTQDK.-	2984.30	3	5.78	0.55	1045.1	1	33/108	3.45E10
	120.87 - 122.05	-.DKPEDAVLDVQGIATVTPAIVQACTQDK.-	2984.30	3	5.74	0.50	983.2	1	33/108	3.59E9
	124.43 - 125.20	-.DKPEDAVLDVQGIATVTPAIVQACTQDK.-	2984.30	3	4.78	0.40	669.6	2	32/108	2.61E9
#11	IF1_ECOLI (P02998) T					90.27			9 (9 0 0 0 0)	0.67
	97.29 - 97.88	-.AKEDNIEM*QGTVLETLPNTM*FR.-	2570.88	2	3.71	0.56	784.8	1	20/42	1.13E9
	99.18 - 99.78	-.ILTGDKVTVELTPYDLSK.-	1993.29	2	5.39	0.63	1314.6	1	20/34	1.47E9
	99.20 - 99.80	-.ILTGDKVTVELTPYDLSK.-	1993.29	3	3.37	0.41	1133.3	1	29/68	1.50E9
	49.52 - 50.81	-.VELENGHVVTAHISGK.-	1690.88	2	2.77	0.59	601.8	1	16/30	2.46E8
	73.31	-.VELENGHVVTAHISGK.-	1690.88	1	3.51	0.53	557.6	1	18/30	6.32E8
	73.04 - 73.66	-.VELENGHVVTAHISGK.-	1690.88	2	4.06	0.58	1332.5	1	20/30	1.46E9
	64.94 - 65.64	-.VELENGHVVTAHISGK.-	1690.88	2	4.20	0.66	2042.2	1	21/30	8.51E8
	91.38 - 92.86	-.VTVELTPYDLSK.-	1365.55	2	3.38	0.56	1218.4	1	18/22	3.26E9
	93.00 - 93.12	-.VTVELTPYDLSK.-	1365.55	1	2.30	0.52	421.0	1	13/22	1.05E9
#12	CSPE_ECOLI (P36997					90.25			9 (9 0 0 0 0)	1.73
	107.03	-.DVFVHFSAIQTNGFK.-	1710.91	1	2.36	0.52	469.4	1	16/28	5.32E8
	105.80 - 107.10	-.DVFVHFSAIQTNGFK.-	1710.91	2	4.75	0.70	1660.1	1	21/28	3.36E9
	88.79	-.GFGFITPEDGSK.-	1255.36	1	2.34	0.40	712.1	1	13/22	2.97E9
	125.36 - 126.71	-.GFGFITPEDGSKDVFVHFSAIQTNGFK.-	2947.25	3	5.08	0.61	1758.6	1	39/104	2.87E9
	126.92 - 128.29	-.GFGFITPEDGSKDVFVHFSAIQTNGFK.-	2947.25	3	3.06	0.35	854.2	1	31/104	3.42E9
	93.21	-.GPSAANVIAL.-	913.05	2	3.18	0.53	995.5	1	15/18	7.39E8
	70.11 - 71.09	-.VEFEITNGAK.-	1108.23	2	2.90	0.36	627.7	1	14/18	4.11E9
	70.09 - 74.84	-.VEFEITNGAK.-	1108.23	1	2.41	0.31	431.7	1	12/18	1.01E10
	72.02	-.VEFEITNGAK.-	1108.23	2	2.65	0.34	741.4	1	15/18	2.09E9
#13	RS18_ECOLI (P02374)					90.25			9 (9 0 0 0 0)	1.41
	78.42 - 79.09	-.FTAEGVQEIDYK.-	1400.52	1	2.00	0.30	543.7	1	13/22	1.93E9
	78.36 - 79.16	-.FTAEGVQEIDYK.-	1400.52	2	4.93	0.58	1505.6	1	19/22	3.08E9
	104.99	-.FTAEGVQEIDYKDIATLK.-	2042.28	2	4.28	0.56	803.0	1	16/34	1.05E9
	104.73 - 105.74	-.FTAEGVQEIDYKDIATLK.-	2042.28	3	3.85	0.41	602.2	1	26/68	1.94E9
	102.62 - 103.20	-.FTAEGVQEIDYKDIATLK.-	2042.28	2	5.04	0.62	1018.3	1	18/34	2.79E9
	102.64 - 103.78	-.FTAEGVQEIDYKDIATLK.-	2042.28	3	4.40	0.52	727.0	1	30/68	3.52E9
	204.49 - 205.52	-.FTAEGVQEIDYKDIATLK.-	2042.28	2	4.33	0.55	870.9	1	17/34	7.88E7
	102.25 - 102.82	-.YLSLLPYTDR.-	1241.42	2	2.72	0.43	534.0	1	14/18	7.32E9
	102.37 - 103.01	-.YLSLLPYTDR.-	1241.42	1	2.02	0.33	281.2	1	11/18	2.75E9
#14	RPOZ_ECOLI (P08374					90.24			9 (9 0 0 0 0)	1.08
	109.57 - 110.85	-.EIEEGLINNQILDVR.-	1755.95	2	3.16	0.38	1043.8	1	16/28	3.89E9
	107.82 - 108.91	-.EIEEGLINNQILDVR.-	1755.95	2	4.80	0.44	1507.8	1	21/28	3.72E9
	109.97 - 110.55	-.ERQEQQEQEAAELQAVTAIAEGR.-	2585.73	3	4.30	0.44	1343.1	1	29/88	3.26E9
	110.22	-.ERQEQQEQEAAELQAVTAIAEGR.-	2585.73	2	4.20	0.51	580.2	1	19/44	1.10E9
	98.90 - 99.02	-.IGNRFDLVLVAAR.-	1444.71	2	2.69	0.16	436.7	2	12/24	1.04E9
	87.77	-.QM*QVGGKDPLVPEENDKTTVIALR.-	2656.01	3	3.15	0.37	1193.5	1	33/92	1.21E9
	85.14	-.QM*QVGGKDPLVPEENDKTTVIALR.-	2656.01	3	4.30	0.52	1075.5	1	31/92	9.42E8
	92.22 - 92.75	-.QMQVGGKDPLVPEENDKTTVIALR.-	2640.01	3	3.66	0.49	869.7	1	29/92	1.64E9
	94.28 - 95.46	-.QMQVGGKDPLVPEENDKTTVIALR.-	2640.01	3	3.52	0.36	844.2	1	29/92	2.03E9
#15	RS16_ECOLI (P02372)					90.21			9 (9 0 0 0 0)	1.83
	67.11 - 67.64	-.IAHWVGQGATISDR.-	1511.67	1	2.69	0.50	356.1	1	14/26	1.79E9
	67.39	-.IAHWVGQGATISDR.-	1511.67	2	3.63	0.38	1474.6	1	19/26	4.45E9
	66.99 - 67.51	-.IAHWVGQGATISDR.-	1511.67	3	4.06	0.50	2599.1	1	33/52	1.34E9
	72.28 - 73.43	-.KRPFYQVVVADSR.-	1565.80	2	3.58	0.52	490.1	1	17/24	4.94E9
	72.38 - 72.93	-.KRPFYQVVVADSR.-	1565.80	3	3.03	0.18	1259.1	2	25/48	2.32E9

	96.71 - 96.98	-.VGFFNPIASEK.-	1209.38	2	2.99	0.52	1218.6	1	17/20		2.23E9
	89.36 - 89.98	-.VGFFNPIASEKEEGTR.-	1781.95	3	4.14	0.47	1185.4	1	30/60		4.14E9
	89.27 - 89.83	-.VGFFNPIASEKEEGTR.-	1781.95	2	3.89	0.59	1036.4	1	21/30		4.44E9
	102.12	-.VGFFNPIASEKEEGTRLDLDR.-	2394.63	3	3.96	0.49	510.0	1	25/80		6.15E9
#16	RS20_ECOLI (P02378)					90.19			9 (9 0 0 0 0)		1.12
	57.84 - 59.00	-.AFNEM*QPIVDR.-	1336.50	2	3.74	0.53	1082.9	1	15/20		4.26E9
	61.41 - 62.35	-.AFNEM*QPIVDR.-	1336.50	2	3.30	0.46	655.8	1	14/20		2.38E9
	62.96 - 63.84	-.AFNEM*QPIVDR.-	1336.50	2	2.98	0.45	1203.4	1	16/20		1.41E9
	64.65 - 66.02	-.AFNEM*QPIVDR.-	1336.50	2	3.39	0.45	1075.9	1	15/20		1.29E9
	80.84 - 81.04	-.AFNEMQPIVDR.-	1320.50	2	3.28	0.47	1162.7	1	16/20		1.57E9
	11.61	-.ANLTAQINK.-	973.11	1	2.00	0.27	424.9	3	11/16		3.29E9
	52.15	-.VYAAIEAGDK.-	1037.15	1	1.86	0.26	257.9	1	13/18		2.37E8
	12.03	-.VYAAIEAGDK.-	1037.15	1	2.30	0.29	1329.1	1	14/18		3.24E9
	12.09	-.VYAAIEAGDK.-	1037.15	2	2.74	0.37	816.0	1	15/18		1.78E9
#17	ECNB_ECOLI (P56549					80.29			8 (8 0 0 0 0)		1.16
	64.09 - 65.52	-.GVGEDISDGGNAISGAATK.-	1719.79	2	4.59	0.67	829.4	1	21/36		1.47E9
	65.90 - 67.08	-.GVGEDISDGGNAISGAATK.-	1719.79	2	5.25	0.63	1165.6	1	23/36		2.83E9
	67.62	-.GVGEDISDGGNAISGAATK.-	1719.79	2	4.89	0.67	1347.2	1	24/36		1.70E9
	71.22	-.GVGEDISDGGNAISGAATK.-	1719.79	2	4.51	0.62	976.8	1	21/36		8.64E8
	74.00 - 74.92	-.GVGEDISDGGNAISGAATK.-	1719.79	2	4.51	0.61	896.9	1	21/36		4.64E9
	75.12 - 76.16	-.GVGEDISDGGNAISGAATK.-	1719.79	2	5.56	0.65	1320.8	1	24/36		6.85E9
	75.14	-.GVGEDISDGGNAISGAATK.-	1719.79	1	2.20	0.41	145.2	20	10/36		1.27E9
	60.43 - 61.03	-.GVGEDISDGGNAISGAATK.-	1719.79	2	5.71	0.67	950.9	1	21/36		5.41E8
#18	RL31_ECOLI (P02432)					80.21			8 (8 0 0 0 0)		0.64
	54.75 - 57.11	-.FNIPGSK.-	762.88	1	1.93	0.35	249.2	10	8/12		1.11E9
	69.14 - 69.71	-.STVGHDLNLDVCSK.-	1545.67	2	4.21	0.60	1789.2	1	20/26		3.86E9
	69.29	-.STVGHDLNLDVCSK.-	1545.67	1	2.52	0.39	543.2	1	15/26		1.21E9
	69.37	-.STVGHDLNLDVCSK.-	1545.67	3	3.28	0.44	452.3	5	18/52		8.64E8
	69.52	-.STVGHDLNLDVCSK.-	1545.67	1	3.69	0.62	720.8	1	16/26		1.00E9
	71.36	-.STVGHDLNLDVCSK.-	1545.67	2	3.12	0.62	920.2	1	16/26		9.23E8
	11.86	-.YEEITASCSCGNVM*K.-	1765.91	2	3.31	0.53	937.5	1	18/28		1.43E9
	67.59	-.YEEITASCSCGNVMK.-	1749.91	2	2.90	0.54	1198.1	1	19/28		7.65E8
#19	OMPA_ECOLI (P0293					80.21			8 (8 0 0 0 0)		0.83
	85.74	-.AALIDCLAPDR.-	1215.38	1	2.06	0.19	480.6	1	14/20		7.27E8
	85.77	-.AALIDCLAPDR.-	1215.38	2	2.86	0.21	1115.9	1	17/20		1.16E9
	81.85	-.DGSVVVLGYTDR.-	1281.40	2	2.95	0.52	1052.8	1	16/22		1.25E9
	50.50 - 52.07	-.GIKDVVTQPQA.-	1156.31	2	2.77	0.44	806.6	2	14/20		4.10E8
	48.28 - 49.95	-.GIKDVVTQPQA.-	1156.31	2	2.65	0.48	785.7	1	15/20		5.01E8
	46.04 - 47.41	-.GIKDVVTQPQA.-	1156.31	2	3.01	0.51	871.3	1	14/20		3.23E8
	112.43 - 112.94	-.LGYPITDDLDIYTR.-	1655.83	2	4.20	0.57	1234.8	1	19/26		5.64E9
	92.46 - 93.81	-.RAQSVVDYLISK.-	1379.59	2	2.52	0.12	719.4	2	15/22		4.36E9
#20	RS19_ECOLI (P02375)					80.18			8 (8 0 0 0 0)		0.55
	107.65	-.GPFIDLHLLK.-	1153.40	1	1.83	0.16	431.0	1	11/18		1.05E9
	96.96	-.KGPFIDLHLLK.-	1281.57	3	3.64	0.28	1429.2	2	25/40		8.36E8
	96.94 - 97.53	-.KGPFIDLHLLK.-	1281.57	2	3.31	0.33	1833.5	1	18/20		1.73E9
	19.58 - 20.88	-.LGEFAPTR.-	891.01	2	2.55	0.36	838.7	1	12/14		4.44E8
	15.68 - 16.84	-.LGEFAPTR.-	891.01	2	2.74	0.41	797.7	1	12/14		4.38E8
	15.98 - 26.93	-.LGEFAPTR.-	891.01	1	2.03	0.31	266.0	1	10/14		7.01E8
	66.32 - 67.41	-.QHVPVFVTDEM*VGHK.-	1739.98	2	3.08	0.52	315.3	1	13/28		2.62E9
	107.63 - 108.80	-.STIFPNM*IGLTIAVHNGR.-	1958.27	2	3.03	0.61	413.4	1	16/34		1.78E9
#21	Q8XCD0 (Q8XCD0) Hy					70.29			7 (7 0 0 0 0)		0.81
	38.06 - 39.34	-.AIVEAAGLK.-	872.05	2	2.51	0.18	638.5	1	14/16		2.56E8
	33.96 - 44.05	-.AIVEAAGLK.-	872.05	1	2.07	0.30	670.6	1	12/16		1.05E9
	134.42 - 135.64	-.DLNDFATVNATYEAFFTEHNATFPAR.-	2964.15	3	5.83	0.55	847.7	1	31/100		3.77E9
	134.97	-.DLNDFATVNATYEAFFTEHNATFPAR.-	2964.15	2	3.27	0.62	403.6	1	15/50		8.31E8
	136.27 - 137.47	-.DLNDFATVNATYEAFFTEHNATFPAR.-	2964.15	3	5.19	0.57	975.8	1	31/100		4.60E9
	138.19 - 139.46	-.DLNDFATVNATYEAFFTEHNATFPAR.-	2964.15	3	4.49	0.57	1090.1	1	31/100		2.16E9
	85.81 - 86.50	-.IEIEAIAVR.-	1014.20	2	3.50	0.39	1339.4	1	15/16		1.37E9
#22	Q9KXA4 (Q9KXA4) Hy					70.28			7 (7 0 0 0 0)		3.22
	123.95 - 125.18	-.ELKDDAWVILEGSIVK.-	1816.09	2	4.32	0.50	1382.5	1	19/30	1	4.98E9
	94.91 - 96.01	-.IEGEIDKDLSSVEVDVK.-	1876.05	3	5.54	0.55	1047.0	1	32/64	1	9.22E9
	96.47	-.IEGEIDKDLSSVEVDVK.-	1876.05	2	4.11	0.57	1282.5	1	20/32	1	8.38E9
	96.59	-.IEGEIDKDLSSVEVDVK.-	1876.05	3	3.92	0.60	805.1	1	29/64	1	2.58E9
	94.69 - 95.89	-.IEGEIDKDLSSVEVDVK.-	1876.05	2	4.92	0.64	1651.9	1	22/32	1	2.63E10
	89.42 - 90.02	-.IEGEIDKDLSSVEVDVK.-	1876.05	2	4.26	0.51	1457.1	1	22/32	1	1.51E9
	125.02 - 125.61	-.YEFRDNSGTIVTDIDDSIWAGQNVSPK.-	3029.22	3	4.84	0.59	758.9	1	30/104		3.06E9
#23	RL32_ECOLI (P02435)					70.24			7 (7 0 0 0 0)		0.96
	78.38 - 79.01	-.RSHDALTAVTSLSVDK.-	1700.88	2	4.75	0.60	1613.3	1	21/30		1.96E9
	76.80 - 77.83	-.RSHDALTAVTSLSVDK.-	1700.88	2	4.56	0.60	697.1	1	15/30		2.57E9
	85.12 - 86.48	-.SHDALTAVTSLSVDK.-	1544.69	2	4.65	0.60	1913.4	1	23/28		5.63E9
	85.19 - 85.83	-.SHDALTAVTSLSVDK.-	1544.69	1	2.64	0.41	977.3	1	17/28		2.41E9
	85.33	-.SHDALTAVTSLSVDK.-	1544.69	3	4.14	0.42	1491.6	1	29/56		1.01E9
	85.34	-.SHDALTAVTSLSVDK.-	1544.69	1	3.54	0.45	798.1	1	16/28		1.79E9
	79.47 - 80.15	-.SHDALTAVTSLSVDKTSGEK.-	2047.21	2	4.11	0.33	261.0	3	15/38		1.32E9
#24	Q7DH26 (Q7DH26) Sh					70.23			7 (7 0 0 0 0)		0.64
	91.75 - 92.63	-.TNACHNGGGFSEVIFR.-	1766.89	2	4.56	0.54	1260.1	1	22/30		1.82E9
	89.54	-.TNACHNGGGFSEVIFR.-	1766.89	2	4.29	0.52	1681.5	1	23/30		1.31E9
	92.53	-.TNACHNGGGFSEVIFR.-	1766.89	3	3.53	0.37	1764.0	1	28/60		6.61E8
	73.24 - 74.59	-.VEYTKYNDDDTFTVK.-	1838.95	2	4.14	0.42	1127.1	1	20/28		1.64E9
	12.86	-.VGDKELFTNR.-	1179.31	2	2.84	0.48	1015.0	1	15/18		1.03E9
	146.09 - 147.37	-.WNLQSLLLSAQITGM*TVTIK.-	2234.65	2	3.14	0.56	354.7	1	12/38		3.81E9
	56.76 - 57.37	-.YNDDDTFTVK.-	1218.25	2	2.87	0.44	848.5	1	16/18		8.77E8
#25	RL27_ECOLI (P02427)					70.20			7 (7 0 0 0 0)		0.61
	107.36 - 108.59	-.FGGESVLAGSIIVR.-	1405.62	2	2.67	0.43	796.9	1	15/26		2.33E9

	103.64 - 104.97	-.FGGESVLAGSIIVR.-	1405.62	2	4.00	0.56	1028.8	1	19/26		2.73E9
	104.55	-.FGGESVLAGSIIVR.-	1405.62	1	2.03	0.40	169.3	2	12/26		8.85E8
	105.57	-.FGGESVLAGSIIVR.-	1405.62	2	2.92	0.46	903.1	1	16/26		2.36E9
	205.00	-.FGGESVLAGSIIVR.-	1405.62	2	2.54	0.46	682.3	1	14/26		4.74E7
	73.06	-.KFISIEAE.-	937.07	1	2.60	0.40	458.0	1	10/14		1.66E9
	73.01	-.KFISIEAE.-	937.07	2	2.55	0.29	949.1	4	12/14		6.46E8
#26	RS21_ECOLI (P02379)					70.18			7 (7 0 0 0 0)		0.48
	34.06 - 35.45	-.AGVLAEVR.-	814.95	2	2.61	0.38	605.0	1	11/14		3.64E8
	36.09 - 37.37	-.AGVLAEVR.-	814.95	2	2.98	0.33	812.7	1	12/14		4.55E8
	39.23 - 39.94	-.AGVLAEVR.-	814.95	2	3.16	0.35	892.7	1	13/14		3.06E8
	33.44 - 42.13	-.AGVLAEVR.-	814.95	1	1.88	0.32	173.6	6	8/14		1.61E9
	80.81 - 81.40	-.VRENEPFDVALR.-	1445.61	2	3.62	0.45	674.7	1	16/22		3.11E9
	81.08	-.VRENEPFDVALR.-	1445.61	3	3.57	0.41	1629.1	1	25/44		9.43E8
	72.24 - 72.85	-.VRENEPFDVALRR.-	1601.79	2	2.66	0.29	282.6	10	11/24		1.54E9
#27	THIO_ECOLI (P00274)					60.26			6 (6 0 0 0 0)		1.21
	111.88 - 112.47	-.GIPTLLLFKNGEVAATK.-	1773.11	2	4.99	0.70	1490.0	1	22/32		2.24E9
	101.65 - 102.60	-.IIHLTDDSFDTDVLK.-	1732.91	2	2.71	0.29	397.9	4	13/28		7.04E9
	101.99	-.IIHLTDDSFDTDVLK.-	1732.91	1	2.41	0.32	866.1	1	16/28		5.66E8
	121.84 - 123.14	-.M*IAPILDEIADEYQGK.-	1823.06	2	2.89	0.40	524.1	1	16/30		9.09E9
	97.10	-.SDKIIHLTDDSFDTDVLK.-	2063.25	3	5.26	0.55	2256.0	1	33/68		1.14E9
	96.53 - 97.12	-.SDKIIHLTDDSFDTDVLK.-	2063.25	2	4.56	0.56	1387.1	1	20/34		9.05E8
#28	Q8X9M2 (Q8X9M2) 30					60.25			6 (6 0 0 0 0)		0.51
	118.05 - 119.33	-.DANDTGSTEVQVALLTAQINHLQGHFAEHK.-	3246.49	3	3.51	0.40	791.3	1	30/116		3.51E9
	120.02	-.DANDTGSTEVQVALLTAQINHLQGHFAEHK.-	3246.49	3	3.66	0.53	985.5	1	32/116		1.97E9
	115.05	-.DANDTGSTEVQVALLTAQINHLQGHFAEHKK	3374.67	3	3.82	0.24	353.4	14	24/120		8.38E8
	113.31	-.DANDTGSTEVQVALLTAQINHLQGHFAEHKK	3374.67	3	5.09	0.53	629.0	1	28/120		7.52E8
	79.43	-.KLLDYLK.-	893.11	2	2.82	0.17	899.5	1	12/12		7.34E8
	79.28	-.KLLDYLK.-	893.11	1	1.94	0.21	497.5	5	9/12		1.13E9
#29	EFTU_ECOLI (P02990)					60.22			6 (6 0 0 0 0)		0.77
	54.46 - 55.70	-.AFDQIDNAPEEK.-	1377.44	2	3.43	0.34	796.3	1	15/22		1.77E9
	109.17	-.ELLSQYDFPGDDTPIVR.-	1966.14	2	2.95	0.53	377.4	1	14/32		1.76E9
	73.33 - 73.85	-.GITINTSHVEYDTPTR.-	1804.94	2	4.35	0.58	754.9	1	16/30		3.45E9
	90.90	-.M*VVTLIHPIAM*DDGLR.-	1814.16	3	3.14	0.53	735.6	1	27/60		9.96E8
	90.19 - 91.46	-.M*VVTLIHPIAM*DDGLR.-	1814.16	2	3.61	0.39	402.5	1	15/30		2.37E9
	117.17	-.TTLTAAITTVLAK.-	1304.56	2	4.20	0.52	2106.1	1	21/24		3.10E9
#30	G3P1_ECOLI (P06977)					60.20			6 (6 0 0 0 0)		0.41
	85.94	-.AGIALNDNFVK.-	1162.32	2	2.90	0.48	931.1	1	14/20		1.15E9
	86.04	-.AGIALNDNFVK.-	1162.32	1	2.56	0.22	582.2	1	14/20		5.68E8
	83.15	-.LVSWYDNETGYSNK.-	1676.77	2	3.82	0.64	821.4	1	16/26		1.43E9
	150.55 - 151.14	-.SDIEIVAINDLLDADYM*AYM*LK.-	2549.90	3	3.96	0.49	1285.7	1	29/84		6.48E8
	88.48	-.VLDLIAHISK.-	1109.34	2	2.97	0.42	739.1	1	14/18		6.02E8
	98.84 - 100.13	-.VPTPNVSVVDLTVR.-	1496.73	2	3.61	0.68	1217.9	1	19/26		2.75E9
#31	OMPC_ECO57 (Q8XE4					60.19			6 (6 0 0 0 0)		0.64
	80.35	-.FQDVGSFDYGR.-	1291.35	2	2.80	0.42	924.3	1	15/20		1.52E9
	95.39 - 96.08	-.INLLDDNQFTR.-	1349.48	2	3.63	0.39	942.9	1	16/20		3.04E9
	69.39	-.NYDDEDILK.-	1125.17	1	2.39	0.10	362.9	2	12/16		1.71E9
	72.40	-.VDGLHYFSDDK.-	1296.37	2	3.15	0.49	1226.1	1	16/20		9.29E8
	78.16 - 79.54	-.VDGLHYFSDDKSVDGDQTYM*R.-	2465.60	3	3.71	0.41	1425.7	1	30/80		2.32E9
	78.12 - 78.82	-.VDGLHYFSDDKSVDGDQTYM*R.-	2465.60	2	3.33	0.48	778.4	1	18/40		1.56E9
#32	RS17_ECOLI (P02373)					50.33			5 (5 0 0 0 0)		0.81
	80.67 - 82.01	-.LHVHDENNECGIGDVVEIR.-	2206.35	3	4.73	0.43	2312.8	1	33/72		4.47E9
	80.79	-.LHVHDENNECGIGDVVEIR.-	2206.35	2	5.84	0.62	2323.3	1	25/36		2.66E9
	82.42 - 83.02	-.LHVHDENNECGIGDVVEIR.-	2206.35	2	6.27	0.67	2969.4	1	28/36		3.46E9
	83.98 - 84.86	-.LHVHDENNECGIGDVVEIR.-	2206.35	2	6.63	0.62	3521.0	1	28/36		2.06E9
	68.21 - 68.81	-.SIVVAIER.-	887.06	2	2.59	0.39	1081.0	1	13/14		1.51E9
#33	YIHD_ECOLI (P32126)					50.33			5 (5 0 0 0 0)		0.33
	100.85	-.DAVIPGLQKDYEEDFK.-	1868.03	2	3.23	0.50	341.5	1	15/30		9.68E8
	32.03 - 33.91	-.DYEEDFK.-	945.95	1	1.86	0.25	612.8	1	10/12		2.89E8
	101.06 - 101.80	-.DYEEDFKTALLR.-	1500.64	2	2.77	0.59	666.1	1	14/22		1.84E9
	146.51 - 146.86	-.ESGFDGELADLTDDILIYHLK.-	2365.58	3	3.35	0.50	472.4	6	23/80		1.77E9
	156.17 - 156.74	-.LNEVIELLQPAWQKEPDLNLLQFLQK.-	3121.62	3	6.54	0.57	1687.0	1	36/100		9.25E8
#34	DCEA_ECO57 (P5822					50.28			5 (5 0 0 0 0)		0.26
	143.54 - 144.37	-.GFEM*DFAELLLEDYK.-	1837.04	2	3.75	0.53	1572.4	1	18/28	1	1.29E9
	128.97 - 130.24	-.GWQVPAFTLGGEATDIVVM*R.-	2164.47	2	5.52	0.68	874.3	1	21/38	1	1.34E9
	116.19	-.LGPYEFICTGRPDEGIPAVCFK.-	2527.84	3	3.63	0.57	1189.4	1	30/84	1	1.27E9
	45.01 - 45.85	-.LM*DLSINK.-	950.14	2	2.52	0.16	1028.0	2	13/14	1	1.11E8
	54.67 - 55.86	-.LQGIAQQNSFK.-	1234.39	2	2.69	0.41	610.4	1	14/20	1	4.97E8
#35	ALF_ECOLI (P11604) F					50.25			5 (5 0 0 0 0)		0.31
	69.25	-.ANEAYLQGQLGNPK.-	1503.64	1	2.58	0.40	293.0	4	12/26		6.54E8
	69.06	-.ANEAYLQGQLGNPK.-	1503.64	2	4.72	0.61	1846.6	1	20/26		1.14E9
	107.14 - 107.74	-.APVIVQFSNGGASFIAGK.-	1764.02	2	4.95	0.69	2038.4	1	25/34		1.48E9
	102.72	-.IFDFVKPGVITGDDVQK.-	1879.15	2	3.50	0.60	731.3	1	21/32		1.25E9
	104.15	-.VKAPVIVQFSNGGASFIAGK.-	1991.32	2	4.38	0.60	960.4	1	18/38		7.89E8
#36	RL33_ECOLI (P02436)					50.22			5 (5 0 0 0 0)		0.33
	60.08	-.LVSSAGTGHFYTTTK.-	1570.73	2	3.70	0.62	1228.6	1	19/28		8.37E8
	12.48 - 13.68	-.LVSSAGTGHFYTTTK.-	1570.73	2	3.30	0.61	877.9	1	17/28		3.04E9
	14.30 - 15.60	-.LVSSAGTGHFYTTTK.-	1570.73	2	4.42	0.67	1487.4	1	19/28		1.50E9
	16.43	-.LVSSAGTGHFYTTTK.-	1570.73	2	2.51	0.58	459.2	1	15/28		1.76E8
	49.62 - 50.59	-.LVSSAGTGHFYTTTK.-	1570.73	2	3.16	0.49	455.8	1	16/28		2.65E8
#37	YCFF_ECOLI (P36950)					50.19			5 (5 0 0 0 0)		0.59
	119.11 - 120.26	-.EIPSDIVYQDDLVTAFR.-	1982.18	2	3.78	0.60	489.3	1	16/32		4.97E9
	120.83 - 121.51	-.EIPSDIVYQDDLVTAFR.-	1982.18	2	3.66	0.56	429.1	1	16/32		3.37E9
	53.55 - 54.07	-.IAEQEGIAEDGYR.-	1451.52	2	3.78	0.54	1288.1	1	19/24		8.32E8
	51.85 - 53.01	-.IAEQEGIAEDGYR.-	1451.52	2	3.52	0.53	1090.0	1	18/24		5.93E8

	49.88 - 51.33	-.IAEQEGIAEDGYR.-	1451.52	2	3.03	0.46	882.6	1	16/24		4.49E8
#38	R31C_ECO57 (Q8X9T					40.26			4 (4 0 0 0 0)		0.96
	118.77	-.VIELDGVTYPYVTIDVSSK.-	2099.37	2	4.10	0.65	591.2	1	18/36		2.75E9
	120.85 - 122.01	-.VIELDGVTYPYVTIDVSSK.-	2099.37	2	5.17	0.66	945.5	1	22/36		7.07E9
	122.68 - 123.77	-.VIELDGVTYPYVTIDVSSK.-	2099.37	2	3.05	0.50	490.8	1	17/36		3.56E9
	117.21 - 117.74	-.VIELDGVTYPYVTIDVSSK.-	2099.37	2	4.52	0.65	638.0	1	18/36		3.27E9
#39	RS5_ECOLI (P02356)					40.19			4 (4 0 0 0 0)		0.27
	70.88	-.ATIDGLENM*NSPEM*VAAK.-	1924.14	2	3.09	0.45	485.4	1	14/34		8.65E8
	84.69 - 85.60	-.AVLEVAGVHNVLAK.-	1420.68	2	2.51	0.47	856.3	1	16/26		1.67E9
	71.94 - 73.08	-.AYGSTNPINVVR.-	1291.44	2	2.58	0.51	683.5	1	15/22		1.17E9
	77.95	-.VFM*QPASEGTGIIAGGAM*R.-	1926.21	2	3.89	0.62	788.2	1	20/36		9.56E8
#40	RL28_ECOLI (P02428)					40.19			4 (4 0 0 0 0)		0.60
	21.15 - 22.95	-.FWVESEKR.-	1081.21	2	2.61	0.15	840.0	2	13/14		4.83E8
	110.15	-.GIDTVLAELR.-	1087.25	1	2.49	0.36	340.5	1	11/18		2.02E9
	109.49 - 110.32	-.GIDTVLAELR.-	1087.25	2	3.86	0.45	1262.7	1	16/18		7.23E9
	100.71	-.KGIDTVLAELR.-	1215.43	2	3.39	0.40	1211.6	1	15/20		6.74E8
#41	Q8XBL1 (Q8XBL1) PTS					40.19			4 (4 0 0 0 0)		0.33
	46.81 - 47.98	-.IVGDGIAIKPTGNK.-	1383.62	2	2.94	0.43	550.8	1	14/26		3.17E8
	78.99 - 79.75	-.LSGSVTVGETPVIR.-	1415.62	2	2.58	0.49	395.3	1	13/26		1.82E9
	111.81	-.STLTPVVISNM*DEIKELIK.-	2147.52	2	3.35	0.58	321.5	1	13/36		9.49E8
	118.83 - 119.40	-.VGDTVIEFDLPQLEEK.-	1833.03	2	3.74	0.47	915.5	2	16/30		2.64E9
#42	RL24_ECO57 (P60625					40.17			4 (4 0 0 0 0)		0.22
	76.32	-.DDEVIVLTGK.-	1089.22	1	2.43	0.27	985.0	1	14/18		1.09E9
	104.41 - 105.36	-.EAAIQVSNVAIFNAATGK.-	1805.03	2	2.92	0.55	587.7	1	14/34		1.63E9
	43.33 - 43.96	-.HQKPVPALNQPGGIVEK.-	1813.09	2	3.44	0.55	755.2	1	20/32		1.41E8
	90.94	-.VIVEGINLVK.-	1084.33	2	2.96	0.44	805.2	1	16/18		1.00E9
#43	IHFA_ECOLI (P06984)					40.17			4 (4 0 0 0 0)		0.18
	72.64	-.AEM*SEYLFDK.-	1249.37	2	3.04	0.33	1008.3	1	14/18		1.33E9
	100.35 - 100.37	-.LSGFGNFDLR.-	1126.25	2	3.42	0.53	1585.3	1	17/18		1.04E9
	45.18 - 46.50	-.TGEDIPITAR.-	1073.18	2	3.29	0.45	630.4	1	16/18		3.39E8
	42.69 - 44.44	-.TGEDIPITAR.-	1073.18	2	2.90	0.38	662.7	1	16/18		4.07E8
#44	YBAB_ECOLI (P17577					30.28			3 (3 0 0 0 0)		0.21
	98.30 - 99.00	-.M*QEEIAQLEVTGESGAGLVK.-	2106.34	2	5.53	0.60	2649.4	1	27/38		1.36E9
	156.44 - 157.74	-.RVEIDPSLLEDDKEM*LEDLVAAAFNDAAR.-	3263.58	3	4.81	0.53	1779.7	1	36/112		1.66E9
	162.52 - 163.05	-.VEIDPSLLEDDKEM*LEDLVAAAFNDAAR.-	3107.39	3	5.15	0.59	1103.7	1	31/108		7.11E8
#45	Q8X4Q9 (Q8X4Q9) Hy					30.27			3 (3 0 0 0 0)		0.25
	113.10 - 113.72	-.LGPADILESDENGIIPEQDR.-	2182.33	2	4.34	0.00	995.4	1	22/38	4	1.56E9
	114.66 - 115.23	-.LGPADILESDENGIIPEQDR.-	2182.33	2	5.38	0.00	1130.9	1	24/38	4	9.06E8
	111.17 - 112.49	-.LGPADILESDENGIIPEQDR.-	2182.33	2	5.07	0.00	1349.0	1	24/38	4	1.80E9
#46	Q8X512 (Q8X512) Hyp					30.27			3 (3 0 0 0 0)		0.25
	113.10 - 113.72	-.LGPADILESDENGIXPEQDR.-	2182.33	2	4.34	0.64	995.4	1	22/38		1.56E9
	114.66 - 115.23	-.LGPADILESDENGIXPEQDR.-	2182.33	2	5.38	0.68	1130.9	1	24/38		9.06E8
	111.17 - 112.49	-.LGPADILESDENGIXPEQDR.-	2182.33	2	5.07	0.65	1349.0	1	24/38		1.80E9
#47	Q8XE61 (Q8XE61) Pos					30.26			3 (3 0 0 0 0)		0.21
	133.80 - 134.44	-.AAFQPVFLEVVDESYR.-	1871.08	2	5.12	0.61	977.3	1	21/30		2.28E9
	109.01	-.EWEGLQDTVFASPPCR.-	1893.04	2	3.71	0.59	899.0	1	16/30		7.27E8
	150.57 - 151.28	-.M*IYSTLAEELSTTVHALALHTYTIK.-	2824.24	3	3.39	0.41	634.2	1	28/96		7.07E8
#48	Q8X5P5 (Q8X5P5) Out					30.24			3 (3 0 0 0 0)		0.26
	78.20	-.SFVAVHNQPGLYVGQQAR.-	1972.20	2	4.72	0.66	977.8	1	20/34		1.76E9
	137.60 - 138.10	-.TDTLLEIAVLPLDSYAKPDIEANYQGR.-	3007.34	3	4.84	0.58	1421.2	1	37/104		1.54E9
	122.75	-.VPYNFLEVNM*QGIQVWHLR.-	2360.72	3	3.11	0.43	1434.6	1	27/72		1.20E9
#49	YCHN_ECOLI (P39164					30.24			3 (3 0 0 0 0)		0.41
	117.23 - 118.39	-.GQKPGEGYNIQQM*LEILTAQNVPVK.-	2773.16	3	3.51	0.33	374.0	6	23/96		3.48E9
	120.36 - 121.54	-.IVIVANGAPYGSESLFNSLR.-	2108.38	2	4.81	0.66	923.2	1	24/38		2.63E9
	115.47	-.IVIVANGAPYGSESLFNSLR.-	2108.38	2	3.38	0.49	924.7	1	23/38		9.38E8
#50	ENO_ECOLI (P08324)					30.24			3 (3 0 0 0 0)		0.33
	31.70	-.DAGYTAVISHR.-	1190.29	2	2.74	0.50	909.6	1	15/20		3.06E8
	120.30	-.FNQIGSLTETLAAIK.-	1606.85	2	4.15	0.44	1093.4	1	22/28		2.75E9
	109.60 - 109.99	-.SGETEDATIADLAVGTAAGQIK.-	2119.27	2	4.79	0.58	956.2	1	19/42		2.70E9
#51	YQJD_ECO57 (P6458					30.24			3 (3 0 0 0 0)		0.54
	121.92 - 121.96	-.SLSDTLEEVLSSSGEK.-	1681.78	1	2.51	0.47	180.2	17	10/30		7.71E8
	121.94	-.SLSDTLEEVLSSSGEK.-	1681.78	3	4.15	0.54	1483.3	1	32/60		6.02E8
	121.66 - 123.10	-.SLSDTLEEVLSSSGEK.-	1681.78	2	4.78	0.66	1158.7	1	20/30		8.04E9
#52	Q8X620 (Q8X620) Hyp					30.24			3 (3 0 0 0 0)		0.24
	61.92	-.LGVEEVVAK.-	944.11	2	2.68	0.21	1137.9	1	15/16		4.18E8
	73.28 - 73.93	-.SALAYLEK.-	895.04	1	1.88	0.10	742.4	1	10/14		9.91E8
	95.77 - 96.38	-.VFDVNEPLSQINQAK.-	1702.89	2	4.78	0.48	1111.6	1	20/28		2.80E9
#53	YBED_ECOLI (P30977					30.23			3 (3 0 0 0 0)		0.36
	119.97 - 121.37	-.GNYHSVSITINATHIEQVETLYEELGK.-	3047.32	3	3.61	0.39	426.8	27	21/104		2.41E9
	129.31 - 130.17	-.LNELLEFPTPFTYK.-	1712.97	2	4.54	0.57	971.1	1	18/26		2.71E9
	143.03	-.VM*GQALPELVDQVVEVVQR.-	2126.46	2	4.65	0.53	823.9	1	23/36		1.08E9
#54	Q9KXD0 (Q9KXD0) Bo					30.23			3 (3 0 0 0 0)		0.50
	80.46 - 81.21	-.ETITHHFFVSGIGQK.-	1701.91	3	3.61	0.39	1353.2	1	28/56	1	2.66E9
	80.58	-.ETITHHFFVSGIGQK.-	1701.91	1	2.98	0.50	294.1	1	15/28	1	1.08E9
	80.33 - 80.90	-.ETITHHFFVSGIGQK.-	1701.91	2	4.57	0.61	1409.2	1	21/28	1	4.95E9
#55	ADHE_ECOLI (P17547					30.22			3 (3 0 0 0 0)		0.24
	66.28	-.FATHGGYLLQGK.-	1292.47	2	2.86	0.46	1039.8	1	17/22		5.53E8
	128.83	-.FLFNNGYADQITSVLK.-	1831.06	2	4.43	0.51	1590.3	1	22/30		1.26E9
	112.25 - 113.62	-.ILINTPASQGGIGDLYNFK.-	2022.29	2	4.35	0.60	403.5	1	15/36		2.29E9
#56	Q8XEB4 (Q8XEB4) Fo					30.22			3 (3 0 0 0 0)		0.18
	94.77	-.EM*LLDAM*ENPEKYPQLTIR.-	2324.66	2	3.40	0.60	342.8	1	17/36		7.66E8
	135.52	-.NYTPYEGDESFLAGATEATTTLWDK.-	2781.92	2	2.79	0.47	299.0	1	14/48		7.64E8
	102.48 - 103.84	-.THAPVDFDTAVASTITSHDAGYINK.-	2632.82	3	4.37	0.65	490.6	1	32/96		1.55E9
#57	YGGX_ECOLI (P52065					30.19			3 (3 0 0 0 0)		0.62

	95.68 - 96.91	-.EAEGQDFQLYPGELGK.-	1781.90	2	3.27	0.60	368.4	1	15/30	8.10E9
	113.70 - 114.87	-.LLEQEM*VNFLFEGK.-	1713.98	2	3.79	0.59	1339.7	1	17/26	1.77E9
	104.89	-.TIFCTFLQR.-	1186.38	2	2.78	0.47	640.4	1	13/16	9.31E8
#58	RL30_ECOLI (P02430)					30.17			3 (3 0 0 0 0)	0.40
	98.12 - 98.75	-.ATLLGLGLR.-	914.13	2	2.61	0.20	765.8	2	13/16	2.07E9
	80.44	-.GM*INAVSFM*VK.-	1229.50	2	3.47	0.40	971.3	1	15/20	4.11E9
	93.85	-.GM*INAVSFMVK.-	1213.50	2	3.24	0.32	740.0	1	14/20	7.82E8
#59	CSRA_ECOLI (P31803					30.17			3 (3 0 0 0 0)	0.34
	127.88 - 129.01	-.VGETLM*IGDEVTVTVLGVK.-	1977.31	2	3.31	0.52	391.3	1	13/36	2.10E9
	131.94 - 132.52	-.VGETLM*IGDEVTVTVLGVK.-	1977.31	2	2.68	0.44	557.7	1	15/36	1.34E9
	125.91 - 127.17	-.VGETLM*IGDEVTVTVLGVK.-	1977.31	2	3.31	0.46	547.7	1	18/36	2.52E9
#60	Q8XCC3 (Q8XCC3) Hy					30.13			3 (3 0 0 0 0)	0.06
	46.99	-.LQLLHDEGR.-	1081.21	2	2.57	0.32	1264.2	1	14/16	1.68E8
	20.23 - 25.34	-.LQLLHDEGR.-	1081.21	1	1.87	0.09	115.1	34	8/16	2.20E8
	25.61 - 26.79	-.LQLLHDEGR.-	1081.21	2	2.69	0.13	775.3	2	15/16	5.76E8
#61	RL21_ECOLI (P02422)					30.13			3 (3 0 0 0 0)	0.16
	104.31	-.IGVPFVDGGVIK.-	1201.44	2	2.63	0.45	721.0	1	18/22	7.13E8
	106.08	-.IGVPFVDGGVIK.-	1201.44	2	2.59	0.42	514.5	1	17/22	1.74E9
	50.69 - 51.63	-.M*YAVFQSGGK.-	1104.26	1	1.90	0.49	143.0	1	13/18	3.59E8
#62	Q8XCT1 (Q8XCT1) Hy|					20.31			2 (2 0 0 0 0)	0.24
	152.94 - 154.08	-.FPEGTSEEQIDKTVDDFINEVIEPNK.-	2995.20	3	6.22	0.54	1152.3	1	32/100	1.28E9
	115.70 - 116.74	-.TVDDFINEVIEPNK.-	1633.78	2	3.18	0.39	522.8	3	12/26	2.83E9
#63	ATPB_ECOLI (P00824					20.27			2 (2 0 0 0 0)	0.16
	137.02	-.FLSQPFFVAEVFTGSPGK.-	1959.23	2	3.70	0.59	511.6	1	18/34	1.34E9
	127.51 - 128.70	-.GIM*EGEYDHLPEQAFYM*VGSIEEAVEK.-	3105.40	3	5.45	0.50	1124.4	1	29/104	1.49E9
#64	DBHB_ECOLI (P02341					20.26			2 (2 0 0 0 0)	0.36
	136.29 - 136.87	-.ALDAIIASVTESLK.-	1431.66	2	5.24	0.61	1880.4	1	21/26	3.23E9
	11.12	-.SQLIDK.-	703.81	1	1.81	0.21	464.8	1	8/10	2.96E9
#65	PGK_ECO57 (Q8XD03					20.25			2 (2 0 0 0 0)	0.18
	147.66 - 149.19	-.ADEQILDIGDASAQELAEILK.-	2243.45	2	3.55	0.53	776.1	1	17/40	1.96E9
	132.71	-.ISYISTGGGAFLEFVEGK.-	1876.10	2	5.06	0.64	1205.7	1	21/34	1.22E9
#66	ELAB_ECOLI (P52084					20.25			2 (2 0 0 0 0)	0.71
	169.17	-.IDDDLTLLSETLEEVLR.-	1975.18	2	5.00	0.44	1133.0	1	20/32	1.14E10
	54.12 - 55.56	-.SSGDPADQKYVELK.-	1537.65	2	2.63	0.40	303.3	4	13/26	1.04E9
#67	Q9LAN9 (Q9LAN9) Put					20.23			2 (2 0 0 0 0)	0.16
	133.45 - 133.57	-.FDLSEDASTETAM*VFGELYR.-	2298.47	2	4.63	0.62	1089.9	1	19/38	1.27E9
	131.41 - 132.14	-.FDLSEDASTETAM*VFGELYR.-	2298.47	2	4.65	0.70	501.4	1	16/38	1.44E9
#68	CEST_ECO57 (P5823					20.23			2 (2 0 0 0 0)	0.10
	130.22 - 130.46	-.FPLDDATPEKLENEIEVVVK.-	2286.56	2	4.60	0.61	205.2	2	12/38	8.38E8
	129.69 - 130.30	-.FPLDDATPEKLENEIEVVVK.-	2286.56	3	3.67	0.51	1472.9	1	29/76	8.68E8
#69	SECB_ECOLI (P15040					20.22			2 (2 0 0 0 0)	0.15
	92.32	-.DISFEAPNAPHVFQK.-	1700.88	2	2.72	0.45	602.1	1	17/28	6.27E8
	152.63 - 153.94	-.LDLDTASSQLADDVYEVVLR.-	2223.42	2	4.35	0.59	867.1	1	17/38	1.96E9
#70	YDHD_ECOLI (P37010					20.22			2 (2 0 0 0 0)	0.23
	113.33 - 113.89	-.FAYVDILQNPDIR.-	1564.77	2	4.34	0.50	1814.8	1	19/24	3.29E9
	78.51	-.GELQQLIK.-	929.10	2	2.78	0.24	944.9	1	12/14	6.45E8
#71	Q8XD87 (Q8XD87) Z1					20.21			2 (2 0 0 0 0)	0.24
	119.06 - 119.67	-.TLLGQQGYGTLADVPEKVDM*VDVFR.-	2769.12	3	3.62	0.49	606.0	1	26/96	2.23E9
	95.25 - 96.42	-.YLLDQGYHVIPVSPK.-	1730.00	2	4.26	0.56	664.0	1	17/28	1.91E9
#72	GLR3_ECOLI (P37687					20.21			2 (2 0 0 0 0)	0.37
	126.74 - 126.86	-.TTVPQIFIDAQHIGGCDDLYALDAR.-	2791.06	3	3.62	0.49	414.6	1	28/96	5.52E9
	126.69 - 126.94	-.TTVPQIFIDAQHIGGCDDLYALDAR.-	2791.06	2	4.25	0.55	778.0	1	20/48	9.61E8
#73	TPX_ECOLI (P37901)					20.21			2 (2 0 0 0 0)	0.10
	99.37	-.DLSDVTLGQFAGK.-	1351.49	2	2.95	0.45	1240.6	1	17/24	9.47E8
	121.60	-.NAEFLQAYGVAIADGPLK.-	1878.12	2	4.14	0.56	1273.6	1	18/34	8.40E8
#74	Q8XDF1 (Q8XDF1) Ou					20.20			2 (2 0 0 0 0)	0.09
	66.12	-.AVGLHYFSK.-	1022.18	2	2.62	0.39	933.4	1	15/16	8.32E8
	127.74	-.NM*STYVDYIINQIDSDNK.-	2150.31	2	4.07	0.57	530.0	1	13/34	7.81E8
#75	RS13_ECOLI (P02369)					20.20			2 (2 0 0 0 0)	0.27
	94.81 - 95.44	-.ISELSEGQIDTLRDEVAK.-	2004.19	3	4.01	0.34	892.4	1	29/68	1.58E9
	94.61 - 95.83	-.ISELSEGQIDTLRDEVAK.-	2004.19	2	3.57	0.45	821.5	1	19/34	3.03E9
#76	RL3_ECO57 (P60440)					20.20			2 (2 0 0 0 0)	0.22
	76.06	-.GAVPGATGSDLIVKPAVK.-	1680.97	2	3.94	0.60	387.6	1	18/34	2.05E9
	124.70	-.IFTEDGVSIPVTVIEVEANR.-	2189.45	2	3.75	0.39	359.5	1	17/38	1.79E9
#77	Q8XCN7 (Q8XCN7) Hy					20.19			2 (2 0 0 0 0)	0.11
	86.40	-.AEAEQTLAALTEK.-	1375.51	1	1.82	0.17	501.4	1	14/24	4.71E8
	86.00	-.AEAEQTLAALTEK.-	1375.51	2	3.87	0.56	1953.2	1	18/24	1.41E9
#78	YJBJ_ECO57 (P68207					20.18			2 (2 0 0 0 0)	0.20
	75.98 - 77.36	-.DQAEKEVVDWETR.-	1605.69	2	3.69	0.43	1358.2	1	18/24	2.61E9
	58.54 - 59.93	-.LTDDDM*TIIEGK.-	1367.51	2	3.46	0.50	1156.6	1	17/22	9.38E8
#79	GLNB_ECOLI (P05826					20.18			2 (2 0 0 0 0)	0.12
	95.93	-.GAEYM*VDFLPK.-	1286.48	2	3.63	0.55	692.0	1	13/20	1.22E9
	132.02	-.IEIVVPDDIVDTCVDTIIR.-	2186.48	2	3.52	0.57	240.1	1	16/36	9.03E8
#80	YCGL_ECOLI (P76003					20.18			2 (2 0 0 0 0)	0.15
	68.02	-.DQTYLYVEK.-	1159.27	1	2.10	0.19	835.7	1	12/16	5.95E8
	116.87 - 117.47	-.GFGQPQLAM*ILPLDGR.-	1730.03	2	3.58	0.58	843.7	1	16/30	1.94E9
#81	EFG_ECOLI (P02996)					20.16			2 (2 0 0 0 0)	0.12
	103.42	-.DVTTGDTLCDPDAPIILER.-	2102.28	2	3.29	0.48	902.2	1	18/36	5.84E8
	128.12 - 128.77	-.IATDPFVGNLTFFR.-	1598.83	2	2.96	0.33	587.7	1	13/26	1.54E9
#82	IHFB_ECOLI (P08756)					20.16			2 (2 0 0 0 0)	0.26
	86.60 - 87.17	-.GFGSFSLHYR.-	1171.29	2	2.67	0.57	971.8	1	14/18	1.25E9
	106.71	-.TVEDAVKEM*LEHM*ASTLAQGER.-	2478.74	3	3.29	0.53	571.8	1	27/84	3.19E9
#83	MINE_ECOLI (P18198)					20.15			2 (2 0 0 0 0)	0.15
	134.28	-.DGDISILELNVTLPEAEELK.-	2199.44	2	2.95	0.57	372.6	1	14/38	8.45E8

	103.52 - 104.49	-.YVQIDPEM*VTVQLEQK.-	1937.20	2	2.87	0.15	369.7	4	14/30	1.82E9
#84	Q8X699 (Q8X699) Hyp					20.14			2 (2 0 0 0 0)	0.05
	51.89 - 53.45	-.AEFEKVESQYEK.-	1487.59	2	2.82	0.32	941.8	1	15/22	5.04E8
	49.58 - 51.22	-.AEFEKVESQYEK.-	1487.59	2	2.73	0.35	492.2	1	13/22	4.17E8
#85	ATPE_ECO57 (P58646					10.29			1 (1 0 0 0 0)	0.02
	65.54	-.KAEEHISSSHGDVDYAQASAELAK.-	2544.67	2	5.71	0.67	926.1	1	20/46	3.02E8
#86	YCIN_ECOLI (P46132)					10.28			1 (1 0 0 0 0)	0.06
	76.65	-.EHEDTLAGIEATGVTQR.-	1827.93	2	5.53	0.73	1862.0	1	23/32	1.08E9
#87	RL13_ECOLI (P02410)					10.28			1 (1 0 0 0 0)	0.05
	104.61	-.AEYTPHVDTGDYIIVLNADK.-	2235.44	2	5.51	0.67	1696.7	1	22/38	8.04E8
#88	MDH_ECO57 (P61891)					10.27			1 (1 0 0 0 0)	0.09
	133.14	-.TQLPSGSELSLYDIAPVTPGVAVDLSHIPTAVK	3377.83	3	5.44	0.54	764.4	1	34/128	1.55E9
#89	FABF_ECOLI (P39435)					10.26			1 (1 0 0 0 0)	0.05
	113.93	-.VVVTGLGM*LSPVGNTVESTWK.-	2191.53	2	5.19	0.64	953.1	1	21/40	8.37E8
#90	YCCJ_ECOLI (P46131)					10.26			1 (1 0 0 0 0)	0.11
	130.41 - 131.30	-.NTSPEIAEAIFEVAGYDEK.-	2084.23	2	5.14	0.66	1491.1	1	21/36	1.98E9
#91	Q8XCK0 (Q8XCK0) Hy					10.25			1 (1 0 0 0 0)	0.12
	117.99 - 118.01	-.IVGQADPVAWVSLQDIQGK.-	2025.29	2	5.10	0.67	2032.3	1	25/36	2.09E9
#92	Q8X807 (Q8X807) Moly					10.24			1 (1 0 0 0 0)	0.06
	119.81 - 120.47	-.ELVGTDATEVAADFPTVEALR.-	2205.41	2	4.85	0.65	1284.5	1	20/40	1.07E9
#93	CSPD_ECOLI (P24245					10.24			1 (1 0 0 0 0)	0.09
	122.21 - 122.71	-.GFGFICPEGGGEDIFAHYSTIQM*DGYR.-	3042.28	3	4.79	0.57	682.9	1	34/104	1.55E9
#94	Q8XCL2 (Q8XCL2) Hyp					10.24			1 (1 0 0 0 0)	0.08
	88.71	-.AQPISVIQIDDPNNPGEK.-	1936.11	2	4.78	0.57	1297.9	1	20/34	1.39E9
#95	Q7AK26 (Q7AK26) Shi					10.23			1 (1 0 0 0 0)	0.09
	129.36	-.WNLQPLLQSAQLTGM*TVTIK.-	2259.65	2	4.64	0.63	678.2	1	19/38	1.49E9
#96	HHA_ECOLI (P23870)					10.22			1 (1 0 0 0 0)	0.06
	105.61	-.NKYELSDNELAVFYSAADHR.-	2343.49	3	4.44	0.60	1373.3	1	33/76	1.09E9
#97	TRXB_ECOLI (P09625					10.22			1 (1 0 0 0 0)	0.08
	114.31	-.LLILGSGPAGYTAAVYAAR.-	1865.17	2	4.36	0.68	1300.2	1	21/36	1.45E9
#98	Q8XDR0 (Q8XDR0) Hy					10.21			1 (1 0 0 0 0)	0.20
	84.42 - 84.96	-.IVDEQPGAECQLIGTATGK.-	1988.18	2	4.27	0.69	604.2	1	21/36	3.52E9
#99	YDFZ_ECO57 (P64465					10.21			1 (1 0 0 0 0)	0.09
	100.62 - 101.88	-.ILSIDTEGLTAEQIR.-	1659.86	2	4.21	0.59	1753.7	1	21/28	1.54E9
#100	RS4_ECOLI (P02354) 3					10.21			1 (1 0 0 0 0)	0.06
	104.85	-.VVNIASYQVSPNDVVSIR.-	1961.21	2	4.17	0.55	609.0	1	17/34	1.10E9
#101	RP5M_ECOLI (P31221					10.21			1 (1 0 0 0 0)	0.12
	100.40 - 101.55	-.M*QLNITGNNVEITEALR.-	1933.18	2	4.13	0.42	896.1	1	19/32	2.11E9
#102	RRF_ECOLI (P16174)					10.20			1 (1 0 0 0 0)	0.07
	130.66	-.ASPSLLDGIVVEYYGTPTPLR.-	2249.55	2	4.03	0.35	1874.0	1	22/40	1.26E9
#103	IMDH_ECOLI (P06981)					10.20			1 (1 0 0 0 0)	0.06
	132.56	-.EALTFDDVLLVPAHSTVLPNTADLSTQLTK.-	3211.61	3	3.99	0.40	530.1	2	27/116	1.02E9
#104	Q8XBL3 (Q8XBL3) PEI					10.20			1 (1 0 0 0 0)	0.08
	112.63	-.VLAEQALAQPTTDELM*TLVNK.-	2302.63	2	3.95	0.55	498.3	1	20/40	1.42E9
#105	RS6_ECOL6 (Q8XDI1)					10.20			1 (1 0 0 0 0)	0.09
	144.13	-.AHYVLM*NVEAPQEVIDELETTFR.-	2722.02	3	3.93	0.53	1274.1	1	30/88	1.58E9
#106	YEBG_ECOLI (P33220					10.20			1 (1 0 0 0 0)	0.06
	79.20	-.LPSPQVVGAESEEEDASHAA.-	2024.09	2	3.91	0.50	1227.6	1	23/38	1.08E9
#107	PTNA_ECOLI (P08186					10.19			1 (1 0 0 0 0)	0.08
	137.92	-.GVLFLVDTWGGSPFNAASR.-	1995.23	2	3.89	0.62	808.2	1	17/36	1.42E9
#108	Q8XBZ4 (Q8XBZ4) Hyp					10.19			1 (1 0 0 0 0)	0.11
	109.68 - 111.05	-.LGVDTSTASCLLAEQLPK.-	1904.15	2	3.89	0.57	1335.9	1	21/34	1.94E9
#109	YAEP_ECOLI (P52099)					10.19			1 (1 0 0 0 0)	0.04
	127.49	-.YAEIASGDLGYVPDALGCVLK.-	2212.48	2	3.84	0.41	779.8	1	18/40	6.26E8
#110	RPOB_ECOLI (P00575					10.19			1 (1 0 0 0 0)	0.05
	105.42	-.FIEQDPEGQYGLEAAFR.-	1971.12	2	3.83	0.53	503.7	1	16/32	8.41E8
#111	RL35_ECOLI (P07085)					10.19			1 (1 0 0 0 0)	0.21
	134.54 - 135.17	-.GDLGLVIACLPYA.-	1362.59	2	3.80	0.53	1427.0	1	18/24	3.60E9
#112	Q7DB68 (Q7DB68) Typ					10.19			1 (1 0 0 0 0)	0.06
	94.89	-.M*EAANLSPSGAVM*PLATSLSGNNSVDEK.-	2824.09	2	3.79	0.60	205.2	4	13/54	1.02E9
#113	Q8X6G9 (Q8X6G9) Z03					10.19			1 (1 0 0 0 0)	0.17
	102.78 - 104.01	-.FIVEDGNTGFFYK.-	1537.70	2	3.78	0.49	1794.5	1	21/24	2.87E9
#114	Q8X7A3 (Q8X7A3) Hyp					10.19			1 (1 0 0 0 0)	0.07
	91.58 - 92.16	-.GCAIDIGTVIDNDNCTSK.-	1954.07	2	3.76	0.56	992.4	1	20/34	1.21E9
#115	DPS_ECOLI (P27430)					10.19			1 (1 0 0 0 0)	0.05
	81.30	-.AIGEAKDDDTADILTAASR.-	1934.05	2	3.72	0.59	954.9	1	21/36	8.01E8
#116	Q8XCL8 (Q8XCL8) Hyp					10.19			1 (1 0 0 0 0)	0.09
	87.89 - 88.63	-.IGAFEIDDGELHGESPGDR.-	2015.08	3	3.71	0.48	1241.2	1	28/72	1.61E9
#117	Q8XBA0 (Q8XBA0) Hyp					10.18			1 (1 0 0 0 0)	0.02
	156.76	-.EVSVDVDALLAAINEISESEVHR.-	2496.71	3	3.68	0.53	716.3	1	27/88	3.66E8
#118	PUR7_ECOLI (P21155					10.18			1 (1 0 0 0 0)	0.07
	122.85	-.TVYSTENPDLLVLEFR.-	1897.12	2	3.68	0.58	550.4	1	15/30	1.16E9
#119	RL16_ECOLI (P02414)					10.18			1 (1 0 0 0 0)	0.10
	108.15	-.GLAQGTDVSFGSFGLK.-	1584.76	2	3.66	0.62	597.6	1	15/30	1.71E9
#120	EFP_ECOLI (P33398) I					10.18			1 (1 0 0 0 0)	0.04
	127.81	-.VPLFVQIGEVIK.-	1342.65	2	3.64	0.58	2090.9	1	20/22	7.09E8
#121	YQFB_ECO57 (P67604					10.18			1 (1 0 0 0 0)	0.16
	126.14	-.VIADIYPGQTQFYVIEFK.-	2132.44	2	3.63	0.58	277.4	1	15/34	2.82E9
#122	FIS_ECOLI (P11028) D					10.18			1 (1 0 0 0 0)	0.09
	81.36	-.VNSDVLTVSTVNSQDQVTQKPLR.-	2529.79	2	3.63	0.57	431.9	1	16/44	1.64E9
#123	RS3_ECOLI (P02352) 3					10.18			1 (1 0 0 0 0)	0.03
	64.42	-.GEILGGM*AAVEQPEKPAAQPK.-	2138.43	2	3.54	0.56	384.7	1	17/40	4.53E8
#124	Q8XAF3 (Q8XAF3) Hyp					10.18			1 (1 0 0 0 0)	0.08
	85.81 - 86.50	-.LEIEAIAVR.-	1014.20	2	3.50	0.00	1339.4	1	15/16	1.37E9

#125	RS1_ECOLI (P02349)					10.17			1 (1 0 0 0 0)		0.05
	147.04	-.M*TESFAQLFEESLKEIETRPGSIVR.-	2915.27	3	3.43	0.41	764.0	1	28/96		8.14E8
#126	YGFE_ECOLI (P45580					10.17			1 (1 0 0 0 0)		0.06
	110.26	-.M*LQQTIEQALLEQGR.-	1775.02	2	3.41	0.47	814.7	1	15/28		9.77E8
#127	DNAK_ECOLI (P04475					10.17			1 (1 0 0 0 0)		0.03
	105.01	-.TTPSIIAYTQDGETLVGQPAK.-	2191.42	2	3.38	0.57	748.1	1	17/40		6.00E8
#128	Q8XDR2 (Q8XDR2) Hy					10.17			1 (1 0 0 0 0)		0.07
	96.83 - 98.08	-.AWDAWVVAGHAPVR.-	1535.73	2	3.36	0.46	1997.8	1	21/26		1.19E9
#129	YAJC_ECOLI (P19677)					10.17			1 (1 0 0 0 0)		0.06
	73.51	-.GDEVLTNGGLVGR.-	1287.41	2	3.36	0.44	1083.2	1	19/24		1.04E9
#130	YGAM_ECOLI (Q4741					10.16			1 (1 0 0 0 0)		0.06
	73.87 - 73.89	-.DAVGCADSFVR.-	1197.27	2	3.28	0.52	1191.1	1	18/20		1.07E9
#131	GPMA_ECO57 (P6270					10.16			1 (1 0 0 0 0)		0.08
	76.74	-.YYLGNADEIAAK.-	1328.45	2	3.28	0.53	1009.6	1	19/22		1.32E9
#132	YBJQ_ECOLI (P75819)					10.16			1 (1 0 0 0 0)		0.15
	87.06 - 88.21	-.EIAFEELGSQAR.-	1350.46	2	3.26	0.46	658.6	1	16/22		2.61E9
#133	TTDB_ECO57 (Q8XBK					10.16			1 (1 0 0 0 0)		0.15
	102.84 - 103.44	-.KNGDKWEM*VSVGPTTSMRM*ESFER.-	2836.18	3	3.10	0.04	388.9	17	23/92		2.56E9
#134	BCP_ECOLI (P23480)					10.15			1 (1 0 0 0 0)		0.04
	107.05	-.FSLPDQDGEQVNLTDFQGQR.-	2295.41	2	3.10	0.56	415.3	1	15/38		6.35E8
#135	CCDA_ECO57 (P6255					10.15			1 (1 0 0 0 0)		0.06
	100.87	-.AYDVNISGLVSTTM*QNEAR.-	2086.27	2	3.05	0.57	672.1	1	17/36		9.84E8
#136	USPA_ECOLI (P28242					10.15			1 (1 0 0 0 0)		0.10
	90.48	-.HILIAVDLSPESK.-	1422.65	2	3.03	0.52	1231.1	1	17/24		1.76E9
#137	YADR_ECOLI (P37026					10.15			1 (1 0 0 0 0)		0.05
	67.20	-.SLIADEDNPNLK.-	1329.44	2	3.02	0.45	808.9	1	15/22		8.88E8
#138	RS11_ECOLI (P02366)					10.15			1 (1 0 0 0 0)		0.05
	76.67	-.ITNITDVTPIPHNGCRPPK.-	2131.42	2	2.99	0.54	579.5	1	16/36		8.98E8
#139	RL9_ECOLI (P02418)					10.14			1 (1 0 0 0 0)		0.03
	97.61	-.DIADAVTAAGVEVAK.-	1430.59	1	2.86	0.50	561.9	1	15/28		6.07E8
#140	PTND_ECOLI (P08188					10.14			1 (1 0 0 0 0)		0.06
	113.00	-.M*QALGFCFSM*VPAIR.-	1761.10	2	2.85	0.60	528.9	1	14/28		9.72E8
#141	FEOA_ECOLI (P33649					10.14			1 (1 0 0 0 0)		0.16
	117.42	-.LLSLGM*LPGSSFNVVR.-	1707.03	2	2.85	0.51	577.0	1	18/30		2.71E9
#142	CH10_ECOLI (P05380)					10.14			1 (1 0 0 0 0)		0.01
	52.41 - 53.10	-.SAGGIVLTGSAAAK.-	1203.37	2	2.84	0.50	1272.3	1	20/26		2.33E8
#143	YNCE_ECO57 (Q8X9X					10.14			1 (1 0 0 0 0)		0.07
	75.87	-.TFDTPTHPNSLALSADGK.-	1873.01	2	2.84	0.48	270.1	1	13/34		1.18E9
#144	CYAY_ECO57 (Q8XAP					10.14			1 (1 0 0 0 0)		0.06
	101.61	-.DLLEQAATQQAGETVSFR.-	1965.11	2	2.83	0.54	693.6	1	15/34		9.82E8
#145	Q8XCY5 (Q8XCY5) Hy					10.14			1 (1 0 0 0 0)		0.04
	100.46	-.CKAM*GVDVNRVYSLDVLVR.-	2211.57	2	2.81	0.06	334.0	5	12/36		7.09E8
#146	DII1_ECO57 (Q9EYF1)					10.14			1 (1 0 0 0 0)		0.03
	67.54	-.RYDDVEVIVK.-	1236.40	2	2.80	0.50	1175.2	1	15/18	1	6.00E8
#147	ODP1_ECOLI (P06958					10.14			1 (1 0 0 0 0)		0.06
	60.14 - 61.26	-.FNIDADKVNPR.-	1289.42	2	2.79	0.32	986.2	1	15/20		1.12E9
#148	Q8XBL2 (Q8XBL2) Put					10.14			1 (1 0 0 0 0)		0.05
	114.49 - 114.57	-.VAEEVAELLLAR.-	1313.53	2	2.71	0.48	592.4	3	12/22		7.82E8
#149	SODM_ECO57 (P6682					10.13			1 (1 0 0 0 0)		0.04
	120.56	-.EFWNVVNWDEAAAR.-	1707.83	2	2.70	0.55	1025.5	1	17/26		7.23E8
#150	RvrsDB|00004288					10.13			1 (1 0 0 0 0)		0.04
	96.36	-.GTDEADAAFLASQRDELGFAK.-	2213.35	2	2.65	0.16	311.5	6	13/40		6.11E8
#151	YGIN_ECOLI (P40718)					10.13			1 (1 0 0 0 0)		0.04
	76.43	-.AYSEAVKGDVLEM*NIR.-	1812.04	2	2.64	0.46	232.7	4	11/30		7.72E8
#152	RvrsDB|00002431					10.13			1 (1 0 0 0 0)		0.07
	106.89	-.FWAALTRRLLGNNDM*AKLR.-	2263.65	2	2.63	0.04	730.0	1	16/36		1.25E9
#153	YFHJ_ECOLI (P37096)					10.13			1 (1 0 0 0 0)		0.08
	107.17 - 107.78	-.EIGEALYDAYPDLDPK.-	1809.95	2	2.61	0.39	504.2	1	13/30		1.37E9
#154	OMPX_ECOLI (P3654					10.13			1 (1 0 0 0 0)		0.05
	63.59 - 64.44	-.FQTTEYPTYK.-	1278.39	2	2.57	0.39	397.7	2	11/18		8.04E8
#155	RvrsDB|00001174					10.13			1 (1 0 0 0 0)		0.06
	124.76	-.NLDISLDRLDR.-	1330.47	2	2.54	0.17	360.2	8	11/20		1.09E9
#156	RvrsDB|00000390					10.13			1 (1 0 0 0 0)		0.10
	76.90 - 77.58	-.LIKIAALGESTVPIK.-	1553.91	2	2.51	0.19	511.1	3	14/28		1.77E9
#157	RvrsDB|00004199					10.13			1 (1 0 0 0 0)		0.16
	99.22 - 99.76	-.TSQEDTAEQRSIVESFLQR.-	2225.36	2	2.50	0.04	265.7	14	11/36		2.86E9
#158	PTCB_ECOLI (P17409					10.13			1 (1 0 0 0 0)		0.06
	133.96	-.YEVPVIIEAFPETLAGEK.-	2006.28	2	2.50	0.40	410.4	1	12/34		9.97E8
#159	RHLB_ECO57 (Q8XAT					10.12			1 (1 0 0 0 0)		0.08
	121.21 - 121.58	-.M*YDLGFIK.-	1003.20	1	2.10	0.22	466.9	1	10/14		1.38E9
#160	RvrsDB|00001234					10.12			1 (1 0 0 0 0)		0.05
	69.86 - 69.99	-.YDLLNPNRVVGSK.-	1475.68	1	2.39	0.20	140.6	21	9/24		8.67E8
#161	GLYA_ECO57 (Q8XA5					10.12			1 (1 0 0 0 0)		0.04
	63.90	-.SPFVTSGIR.-	964.10	1	2.16	0.35	157.2	2	10/16		6.43E8
#162	Q8X5P4 (Q8X5P4) AT					10.11			1 (1 0 0 0 0)		0.14
	109.53 - 110.73	-.DSLLAELR.-	917.04	1	2.14	0.09	275.2	1	10/14		2.47E9
#163	RvrsDB|00002686					10.10			1 (1 0 0 0 0)		0.01
	45.23 - 47.06	-.QKKERLLR.-	1071.30	1	1.97	0.06	156.6	11	8/14		2.21E8
#164	RvrsDB|00002250					10.09			1 (1 0 0 0 0)		0.07
	77.11	-.ELLKGMAENRGTAR.-	1546.78	1	1.87	0.03	149.2	17	11/26		1.23E9
#165	Q8X5R6 (Q8X5R6) Hy					10.09			1 (1 0 0 0 0)		0.16
	70.24	-.FQQSIQDDK.-	1109.17	1	1.82	0.01	364.3	2	11/16		2.78E9
#166	RL19_ECOLI (P02420)					10.09			1 (1 0 0 0 0)		0.01
	49.49 - 50.32	-.VWVVEGSK.-	904.05	1	1.81	0.34	676.4	1	11/14		1.55E8
